# Supplementary material for: Social distancing in America: Understanding long-term adherence to COVID-19 mitigation recommendations
Source: PLoS One. 2021 Sep 24;16(9):e0257945. doi: 10.1371/journal.pone.0257945 (PMC8462713; doi:10.1371/journal.pone.0257945)
Supplement: S1 Survey — (PDF) [file pone.0257945.s001.pdf]

Lead Researcher: Benjamin van Rooij, Professor of Law; School of Law, University of California, Irvine.

Contact: bvanrooij@law.uci.edu, (949) 824-0066.

We are asking you to take part in a study conducted by researchers at the University of California, Irvine. Participating in this study is optional. If you choose to be in the study, you will complete a survey. The survey includes questions about demographics as well as the measures to stop the spread of the Coronavirus. This survey will help us learn more about how and why people comply with measures taken to stop the spread of the Coronavirus. The survey will take about 20 minutes to complete. You can stop the survey at any time. The survey is anonymous, and no one will be able to link your answers back to you. Please do not include your name or other information that could be used to identify you in the survey responses. The data you provide may be collected and used by SurveyMonkey as per its privacy agreement. This survey contains a number of checks to make sure that participants are finishing the tasks honestly and completely.

This research is for residents of the United States over the age of 18; if you are not a resident of the United States and/or under the age of 18, please do not complete this survey. You will receive $3.00 for your participation in this study. If you have any comments, concerns, or questions regarding the conduct of this research, please contact the researchers listed at the top of this form. If you have questions or concerns about your rights as a research participant, you can contact the UCI Institutional Review Board by phone, (949) 824-6662, by e-mail at IRB@research.uci.edu, or at 141 Innovation, Suite 250, Irvine, CA 92697.

What is an IRB? An Institutional Review Board (IRB) is a committee made up of scientists and non-scientists. The IRB's role is to protect the rights and welfare of human subjects involved in research. The IRB also assures that the research complies with applicable regulations, laws, and institutional policies.

* 1. Please confirm that you agree to participate in this research project by clicking on the statement below:

- ◯ I agree to participate in this research project
- ◯ I do not agree to participate in this research project

Please answer the questions below:

* 2. What is your age?

* 3. What is your gender?

- Male
- Female
- Other

* 4. What country are you from?

* 5. What is your country of residence?

- United States
- Other

* 6. What state do you live in?

* 7. What was your employment status prior to the outbreak of the Coronavirus?

- Employed full time
- Employed part time
- Unemployed and currently looking for work
- Unemployed and not currently looking for work
- Student
- Retired
- Homemaker
- Self-employed
- Unable to work

* 8. What is the highest degree or level of education you have completed?

- Some high school, no diploma
- High school graduate, diploma
- Some college credit, no degree
- Associate degree
- Bachelor's degree
- Master's degree
- Professional degree
- Doctorate degree

* 9. Does your job require you to care for Coronavirus patients?

- Yes
- No, but I might be in the upcoming months
- No

* 10. Since the authorities took measures to stop the spread of the Coronavirus, **I keep a safe distance (6 feet or more):**

	Never	Almost never	Occasionally	Frequently	Usually	Almost always	Always
...from people outside of my direct household.	◯	◯	◯	◯	◯	◯	◯
...from my neighbors.	◯	◯	◯	◯	◯	◯	◯
...from colleagues at work.	◯	◯	◯	◯	◯	◯	◯
...from friends and family outside of my direct household.	◯	◯	◯	◯	◯	◯	◯
...from others when I go grocery shopping.	◯	◯	◯	◯	◯	◯	◯
...from others when going for a walk or exercising.	◯	◯	◯	◯	◯	◯	◯
...from others when commuting/traveling.	◯	◯	◯	◯	◯	◯	◯

* 11. I believe:

	Strongly disagree	Disagree	Slightly disagree	Neither agree nor disagree	Slightly agree	Agree	Strongly agree
...the Coronavirus is a major threat to my own health.	◯	◯	◯	◯	◯	◯	◯
...the Coronavirus is a major threat to the general health.	◯	◯	◯	◯	◯	◯	◯
...the Coronavirus is a major threat to the health of friends and family.	◯	◯	◯	◯	◯	◯	◯

* 12. I morally believe:

	Strongly disagree	Disagree	Slightly disagree	Neither agree nor disagree	Slightly agree	Agree	Strongly agree
...people should keep a safe distance from others (6 feet or more) to contain the Coronavirus.	◯	◯	◯	◯	◯	◯	◯

* 13. Please indicate your agreement with the following statements.

	Strongly disagree	Disagree	Slightly disagree	Neither agree nor disagree	Slightly agree	Agree	Strongly agree
Authorities and government officials have been consistent with their approach to containing the Coronavirus.							
Authorities and government officials have responded adequately with their approach to containing the Coronavirus.							
This question is to verify you are reading the questions, please respond "Strongly agree."							

* 14. Due to the measures to contain the Coronavirus, I will likely:

	Extremely unlikely	Unlikely	More or less unlikely	Neither unlikely nor likely	More or less likely	Likely	Extremely likely
...lose income.							
...lose my job.							
...not be able to work.							
...not be able to work as effectively as normal.							
...experience a negative impact on my social life.							

* 15. How ***probable*** is it that:

	Extremely improbable	Improbable	Somewhat improbable	Neither improbable nor probable	Somewhat probable	Probable	Extremely probable
...authorities will **find out** if you do not keep a safe distance (6 feet or more) from people outside of your direct household?							
...authorities **will punish you** if you do not keep a safe distance (6 feet or more) from people outside of your direct household?							

* 16. How much will you ***suffer*** if authorities punish you if you do not keep a safe distance (6 feet or more) from people outside of your own household?

- ◯ Extreme suffering
- ◯ High suffering
- ◯ Moderate suffering
- ◯ Somewhat suffering
- ◯ Low suffering
- ◯ No suffering at all

* 17. Please indicate your agreement with the following statements.

At this moment, **I am able to** keep a safe distance (6 feet or more) from:

	Strongly disagree	Disagree	Slightly disagree	Neither agree nor disagree	Slightly agree	Agree	Strongly agree
...people outside of my direct household.	○	○	○	○	○	○	○
...my neighbors.	○	○	○	○	○	○	○
...my colleagues at work.	○	○	○	○	○	○	○
...friends and family outside of my direct household.	○	○	○	○	○	○	○
...others when I go grocery shopping.	○	○	○	○	○	○	○
...others when going for a walk or exercising.	○	○	○	○	○	○	○
...others when commuting/traveling.	○	○	○	○	○	○	○

* 18. Please indicate your agreement with the following statements.

At this moment, ***it is still possible for me*** to come at ***an unsafe distance (closer than 6 feet) from:***

	Strongly disagree	Disagree	Slightly disagree	Neither agree nor disagree	Slightly agree	Agree	Strongly agree
...people outside of my direct household.	○	○	○	○	○	○	○
...my neighbors.	○	○	○	○	○	○	○
...my colleagues at work.	○	○	○	○	○	○	○
...friends and family outside of my direct household.	○	○	○	○	○	○	○
...others when I go grocery shopping.	○	○	○	○	○	○	○
...others when going for a walk or exercising.	○	○	○	○	○	○	○
...others when commuting/traveling.	○	○	○	○	○	○	○

* 19. ***Most people I know*** are keeping a safe distance (6 feet or more):

	Strongly disagree	Disagree	Slightly disagree	Neither agree nor disagree	Slightly agree	Agree	Strongly agree
...from people outside of their direct household.	◯	◯	◯	◯	◯	◯	◯
...from their neighbors.	◯	◯	◯	◯	◯	◯	◯
...from their colleagues at work.	◯	◯	◯	◯	◯	◯	◯
...from friends and family outside of their direct household.	◯	◯	◯	◯	◯	◯	◯
...from others when they go grocery shopping.	◯	◯	◯	◯	◯	◯	◯
...from others when they go for a walk or exercising.	◯	◯	◯	◯	◯	◯	◯
...from others when commuting/traveling.	◯	◯	◯	◯	◯	◯	◯

* 20. Please indicate your agreement with the following statements.

	False	More or less false	I'm not sure	More or less true	True
I should try harder to control myself when I'm having fun.	◯	◯	◯	◯	◯
I do things without giving them enough thought.	◯	◯	◯	◯	◯
When I'm doing something fun (like partying or acting silly), I tend to get carried away and go too far.	◯	◯	◯	◯	◯
I say the first thing that comes to my mind without thinking enough about it.	◯	◯	◯	◯	◯
I stop and think things through before I act.	◯	◯	◯	◯	◯

* 21. It is ***acceptable to break a legal rule*** if:

	Strongly disagree	Disagree	Slightly disagree	Neither agree nor disagree	Slightly agree	Agree	Strongly agree
...the legal rule is clearly against your moral principles.	◯	◯	◯	◯	◯	◯	◯
...this legal rule makes unreasonable demands of you.	◯	◯	◯	◯	◯	◯	◯
...obeying this legal rule is very expensive for you.	◯	◯	◯	◯	◯	◯	◯
...this legal rule is not enforced.	◯	◯	◯	◯	◯	◯	◯
...most of your direct colleagues and/or friends also break this legal rule.	◯	◯	◯	◯	◯	◯	◯
...you are in one way or another unable to do what this legal rule asks of you.	◯	◯	◯	◯	◯	◯	◯
...most of your direct colleagues and/or friends think breaking the legal rule is justified.	◯	◯	◯	◯	◯	◯	◯
...you do not know this legal rule.	◯	◯	◯	◯	◯	◯	◯
...you do not understand this legal rule.	◯	◯	◯	◯	◯	◯	◯
...this legal rule has not been published.	◯	◯	◯	◯	◯	◯	◯
...you feel that this legal rule was made without representing your interests.	◯	◯	◯	◯	◯	◯	◯
...you think this legal rule is enforced unfairly.	◯	◯	◯	◯	◯	◯	◯
This question is to verify you are reading the questions, please respond "Strongly agree."	◯	◯	◯	◯	◯	◯	◯

* 22. In **enforcing** the measures to reduce the spread of the Coronavirus, **I** expect that the authorities will:

	Strongly disagree	Disagree	Slightly disagree	Neither agree nor disagree	Slightly agree	Agree	Strongly agree
...treat people with respect.	◯	◯	◯	◯	◯	◯	◯
...give a person the chance to tell their side of the story if the person is accused of violating measures to contain the Coronavirus.	◯	◯	◯	◯	◯	◯	◯
...treat people fairly, despite gender, race, religion, or socio-economic background.	◯	◯	◯	◯	◯	◯	◯
...be honest in enforcing measures to contain the Coronavirus.	◯	◯	◯	◯	◯	◯	◯

* 23. The following statements are about ***the measures that the authorities have taken to contain the Coronavirus***.

	Strongly disagree	Disagree	Neither agree nor disagree	Agree	Strongly agree
I feel a moral obligation to obey the authorities handling the Coronavirus.	◯	◯	◯	◯	◯
I feel a moral duty to support the decisions of the authorities handling the Coronavirus, even if I disagree with them.	◯	◯	◯	◯	◯
I feel a moral duty to obey the instructions of the authorities handling the Coronavirus, even when I don't understand the reasons behind them.	◯	◯	◯	◯	◯

* 24. Please indicate your agreement with the following statements.

	Strongly disagree	Disagree	Neither agree nor disagree	Agree	Strongly agree
People like me have no choice but to obey the authorities handling the Coronavirus.	◯	◯	◯	◯	◯
If you don't do what the authorities handling the Coronavirus tell you they will treat you badly.	◯	◯	◯	◯	◯
I only obey the authorities handling the Coronavirus because I am afraid of them.	◯	◯	◯	◯	◯

* 25. How likely are you to:

	Extremely unlikely	Unlikely	More or less unlikely	Neither unlikely nor likely	More or less likely	Likely	Extremely likely
...**call the authorities** to report someone you witnessed violating measures created to contain the Coronavirus.	◯	◯	◯	◯	◯	◯	◯
...**give the authorities information** to help them find someone suspected of violating measures created to contain the Coronavirus?	◯	◯	◯	◯	◯	◯	◯

* 26. According to measures adopted by authorities to contain the Coronavirus, I am **currently** required to:

	Yes	No	Unsure
...keep a safe distance (6 feet or more) from people outside of my direct household.	◯	◯	◯

* 27. To me, the measures authorities have adopted to reduce the spread of the Coronavirus are…

- Extremely unclear
- Unclear
- Slightly unclear
- Neither unclear nor clear
- Slightly clear
- Clear
- Extremely clear

* 28. The Coronavirus makes me feel:

	Strongly disagree	Disagree	Slightly disagree	Neither agree nor disagree	Slightly agree	Agree	Strongly agree
...angry.							
...anxious.							
...powerless.							
...depressed.							
...stressed.							
...lonely.							

* 29. Please indicate how much you trust **scientists** to:

	Completely distrust	Partially distrust	Neither trust nor distrust	Partially trust	Completely trust
...create knowledge that is unbiased and accurate?					
...create knowledge that is useful?					
...advise government officials on policy?					
...inform the public on important issues?					

* 30. Please indicate how much you trust **traditional media** (e.g., newspapers, TV news, news apps) to be unbiased and accurate.

- Completely distrust
- Partially distrust
- Neither trust nor distrust
- Partially trust
- Completely trust

* 31. What is your ethnicity?

- White
- Hispanic or Latino
- Black or African American
- Native American or American Indian
- Asian or Pacific Islander
- Other

* 32. What is your current insurance status?

- Uninsured
- Private insurance
- Public insurance

* 33. How would you describe your political view?

- Very progressive
- Slightly progressive
- Slightly conservative
- Very conservative
- Prefer not to say

* 34.

Think of this ladder as showing where people stand in the United States.

At the top of the ladder are the people who are the best off - those who have the most money, the most education, and the most respected jobs. **At the bottom** are the people who are the worst off - who have the least money, least education, and the least respected jobs or no job. The higher up you are on the ladder, the closer you are to the people at the very top; the lower you are, the closer you are to the people at the very bottom.

Where would you place yourself on this ladder?

Please indicate on the slider below where you think you stood **before** the Coronavirus started, relative to other people in the United States.

1 10

* 35. Please indicate on the slider below where you think you will stand **after** the Coronavirus will be over and all current measures are lifted, relative to other people in the United States.

1 10

* 36. Do you have health issues that may put you at increased risk for severe illness from the Coronavirus?

- Yes
- No

* 37. Does anyone you know have health issues that may put them at increased risk for severe illness from the Coronavirus?

- Yes
- No

Thank you for taking the time to complete this survey. We truly value the information you have provided.

Please continue to the next page to be redirected back to SurveyMonkey for payment.
