# Supplementary material for: Social distancing in America: Understanding long-term adherence to COVID-19 mitigation recommendations
Source: PLoS One. 2021 Sep 24;16(9):e0257945. doi: 10.1371/journal.pone.0257945 (PMC8462713; doi:10.1371/journal.pone.0257945)
Supplement: S1 Dataset — (DOCX) [file pone.0257945.s002.docx]

**Dataset and syntax files**

All relevant datasets and syntax files can be accessed at the Figshare repository of the University of Amsterdam through the following [link](https://uvaauas.figshare.com/articles/dataset/Social_Distancing_in_America_Compliance_with_COVID-19_mitigation_measures_in_the_United_States/13125206). Stored here are the following files:

|  | **File name** | **Description** | **Program** |
| --- | --- | --- | --- |
| 1 | Corona Compliance survey US W1 May.sav | Dataset May survey (raw) | SPSS |
| 2 | Corona Compliance survey US W2 June.sav | Dataset June survey (raw) | SPSS |
| 3 | Corona Compliance survey US W3 July.sav | Dataset July survey (raw) | SPSS |
| 4 | Corona Compliance survey US W1-W3 May-July.sav | Combined dataset May, June and July (processed) | SPSS |
| 5 | Corona Compliance survey US W1 May Syntax data prep R1.sps | Syntax file for data processing, May survey. *Version R1 (manuscript revision)* | SPSS |
| 6 | Corona Compliance survey US W2 June Syntax data prep R1.sps | Syntax file for data processing, June survey. *Version R1 (manuscript revision)* | SPSS |
| 7 | Corona Compliance survey US W3 July Syntax data prep R1.sps | Syntax file for data processing, July survey. *Version R1 (manuscript revision)* | SPSS |
| 8 | Corona Compliance survey US W1-3 Comparison Syntax R1.sps | Syntax file for comparison of variables between waves. *Version R1 (manuscript revision)* | SPSS |
| 9 | Corona Compliance survey US W1 May.dta | Dataset May survey (processed) | STATA |
| 10 | Corona Compliance survey US W2 June.dta | Dataset June survey (processed) | STATA |
| 11 | Corona Compliance survey US W3 July.dta | Dataset July survey (processed) | STATA |
| 12 | Corona Compliance survey US W1-3 May-July.dta | Combined dataset May, June and July (processed) | STATA |
| 13 | Corona Compliance survey US W1-W3 SYNTAX R1.do | Syntax file for main analysis, May-July survey. *Version R1 (manuscript revision)* | STATA |
| 14 | Corona Compliance survey US W1 May SYNTAX R1.do | Syntax file for main analysis, separately for May survey. *Version R1 (manuscript revision)* | STATA |
| 15 | Corona Compliance survey US W2 June SYNTAX R1.do | Syntax file for main analysis, separately for June survey. *Version R1 (manuscript revision)* | STATA |
| 16 | Corona Compliance survey US W3 July SYNTAX R1.do | Syntax file for main analysis, separately for July survey. *Version R1 (manuscript revision)* | STATA |

**Reported analyses**

Raw data (SPSS .sav) were processed in using the syntax files (SPSS .sps). The resulting files were aggregated to produce the combined dataset.

Development of the dependent and independent variables across the three surveys was examined using the Corona Compliance survey US W1-3 Comparison Syntax R1 syntax file (SPSS .sps). The relevant sections are (1) *COMPLIANCE W1 W2 W3 with CONTROLS* (average adherence), (2) *NBREG* *ALWAYS COMPLY W1 W2 W3 with CONTROLS* (full adherence), (3) *LOG REG for binary DVs* (development of predictor variables, binary variables), and (4) *IVs across waves* (ANOVA with robust standard errors comparing independent variables (development of predictor variables, scale measures).

For the main analysis, the hierarchical regression model was estimated in STATA. For this, the combined processed dataset (Corona Compliance survey US W1-3 May-July.dta) and the Corona Compliance survey US W1-W3 SYNTAX R1.do syntax were used. The relevant sections are (1) *HIERARCHICAL MODEL* (hierarchical regression model), (2) *INTERACTIONS WITH SURVEY WAVE* (change in predictors across waves), and (3) *Mediation model* (mediation models).

**Supplementary analyses**

Finally, supplementary files have been provided to conduct these analyses separately for each survey wave, using the processed datafiles (STATA .dta) and syntax files (STATA .do). These separate analyses are not reported in the manuscript (where only the combined analysis is presented).
