# Supplementary material for: Social distancing in America: Understanding long-term adherence to COVID-19 mitigation recommendations
Source: PLoS One. 2021 Sep 24;16(9):e0257945. doi: 10.1371/journal.pone.0257945 (PMC8462713; doi:10.1371/journal.pone.0257945)
Supplement: S1 Table — May 8–18 (Survey 1. N = 1012). Note. *–Correlation is significant at the .05 level. **–Correlation is significant at the .01 level. Gender–Female as reference category. Political orientation–N = 866. (DOCX) [file pone.0257945.s003.docx]

|  | **Age** | **Gender** | **Minority** | **Education** | **Employed** | **COVID care** | **Insurance** | **SES pre-COVID-19** | **SES change** | **Health risk self** | **Health risk others** | **Political orientation** |
| --- | --- | --- | --- | --- | --- | --- | --- | --- | --- | --- | --- | --- |
| Age |  |  |  |  |  |  |  |  |  |  |  |  |
| Gender | -0.021 |  |  |  |  |  |  |  |  |  |  |  |
| Minority | -.076** | 0.019 |  |  |  |  |  |  |  |  |  |  |
| Education | 0.032 | -.074** | -0.043 |  |  |  |  |  |  |  |  |  |
| Employed | -0.045 | -.151** | -0.033 | .300** |  |  |  |  |  |  |  |  |
| COVID care | -.075** | -0.040 | 0.056 | .066* | .162** |  |  |  |  |  |  |  |
| Insurance | 0.012 | 0.019 | -0.039 | .257** | .224** | .078** |  |  |  |  |  |  |
| SES pre-COVID-19 | -.061** | -0.044 | -0.023 | .145** | .061* | .100** | .158** |  |  |  |  |  |
| SES change | 0.037 | -.078** | 0.033 | -0.011 | 0.050 | 0.028 | 0.014 | -.200** |  |  |  |  |
| Health risk self | .134** | 0.015 | -.065* | -0.029 | -0.059 | .168** | 0.013 | -0.032 | -0.003 |  |  |  |
| Health risk others | .062* | .088** | -0.047 | 0.009 | -0.013 | .064* | 0.024 | -0.037 | -0.028 | .343** |  |  |
| Political orientation | .097** | -0.029 | -.090** | -.084** | -0.007 | -0.059 | -0.015 | -0.017 | 0.023 | -.077* | -.095** |  |
| Adherence | .090** | .102** | 0.029 | -0.010 | -.083** | -.077** | 0.012 | 0.030 | -0.008 | 0.036 | .077** | -.073** |
