# Supplementary material for: Social distancing in America: Understanding long-term adherence to COVID-19 mitigation recommendations
Source: PLoS One. 2021 Sep 24;16(9):e0257945. doi: 10.1371/journal.pone.0257945 (PMC8462713; doi:10.1371/journal.pone.0257945)
Supplement: S2 Table — June 8–16 (Survey 2. N = 986). Note. *–Correlation is significant at the .05 level. **–Correlation is significant at the .01 level. Gender–Female as reference category. Political orientation–N = 880. (DOCX) [file pone.0257945.s004.docx]

|  | **Age** | **Gender** | **Minority** | **Education** | **Employed** | **COVID care** | **Insurance** | **SES pre-COVID-19** | **SES change** | **Health risk self** | **Health risk others** | **Political orientation** |
| --- | --- | --- | --- | --- | --- | --- | --- | --- | --- | --- | --- | --- |
| **Age** |  |  |  |  |  |  |  |  |  |  |  |  |
| **Gender** | -0.032 |  |  |  |  |  |  |  |  |  |  |  |
| **Minority** | -.056^*^ | .070^*^ |  |  |  |  |  |  |  |  |  |  |
| **Education** | 0.018 | -.079^**^ | -0.021 |  |  |  |  |  |  |  |  |  |
| **Employed** | -.055^*^ | -.116^**^ | -0.027 | .276^**^ |  |  |  |  |  |  |  |  |
| **COVID care** | -.124^**^ | -.083^**^ | .072^*^ | .076^**^ | .168^**^ |  |  |  |  |  |  |  |
| **Insurance** | 0.020 | -0.034 | -.106^**^ | .221^**^ | .252^**^ | 0.030 |  |  |  |  |  |  |
| **SES pre-COVID-19** | -0.038 | -.068^*^ | 0.041 | .133^**^ | .122^**^ | .137^**^ | .146^**^ |  |  |  |  |  |
| **SES change** | .069^**^ | -0.029 | .068^*^ | -0.015 | -0.012 | 0.015 | -0.005 | -.215^**^ |  |  |  |  |
| **Health risk self** | .147^**^ | 0.030 | -.067^*^ | -0.025 | -.096^**^ | .084^**^ | -0.021 | -0.005 | -0.040 |  |  |  |
| **Health risk others** | .052^*^ | .136^**^ | -.151^**^ | 0.003 | -0.050 | 0.039 | 0.033 | -0.045 | -.062^*^ | .400^**^ |  |  |
| **Political orientation** | .135^**^ | -0.031 | -.088^**^ | -0.022 | -0.022 | -.075^*^ | 0.020 | .081^**^ | -0.009 | -.061^*^ | -0.057 |  |
| **Adherence** | .089^**^ | .086^**^ | .086^**^ | -0.002 | -.064^*^ | -0.031 | -0.007 | 0.037 | -0.042 | .073^**^ | 0.018 | -.071^**^ |
