# Supplementary material for: Social distancing in America: Understanding long-term adherence to COVID-19 mitigation recommendations
Source: PLoS One. 2021 Sep 24;16(9):e0257945. doi: 10.1371/journal.pone.0257945 (PMC8462713; doi:10.1371/journal.pone.0257945)
Supplement: S3 Table — July 11–17 (Survey 3. N = 921). Note. *–Correlation is significant at the .05 level. **–Correlation is significant at the .01 level. Gender–Female as reference category. Political orientation–N = 803. (DOCX) [file pone.0257945.s005.docx]

|  | **Age** | **Gender** | **Minority** | **Education** | **Employed** | **COVID care** | **Insurance** | **SES pre-COVID-19** | **SES change** | **Health risk self** | **Health risk others** | **Political orientation** |
| --- | --- | --- | --- | --- | --- | --- | --- | --- | --- | --- | --- | --- |
| **Age** |  |  |  |  |  |  |  |  |  |  |  |  |
| **Gender** | 0.045 |  |  |  |  |  |  |  |  |  |  |  |
| **Minority** | -0.050 | 0.011 |  |  |  |  |  |  |  |  |  |  |
| **Education** | 0.015 | -.058^*^ | 0.037 |  |  |  |  |  |  |  |  |  |
| **Employed** | -0.044 | -.124^**^ | 0.030 | .342^**^ |  |  |  |  |  |  |  |  |
| **COVID care** | -.093^**^ | -.140^**^ | 0.063 | .135^**^ | .231^**^ |  |  |  |  |  |  |  |
| **Insurance** | -0.008 | -0.020 | -0.042 | .269^**^ | .319^**^ | 0.044 |  |  |  |  |  |  |
| **SES pre-COVID-19** | -0.043 | -.083^**^ | 0.014 | .185^**^ | .169^**^ | .152^**^ | .150^**^ |  |  |  |  |  |
| **SES change** | 0.014 | -0.016 | 0.032 | -0.035 | -0.036 | 0.022 | -0.035 | -.184^**^ |  |  |  |  |
| **Health risk self** | .191^**^ | 0.041 | -0.025 | 0.010 | -.109^**^ | .084^*^ | -0.052 | -.060^*^ | -0.037 |  |  |  |
| **Health risk others** | .104^**^ | .154^**^ | -.114^**^ | .060^*^ | -0.028 | 0.007 | 0.002 | -.070^*^ | -0.053 | .475^**^ |  |  |
| **Political orientation** | .118^**^ | 0.005 | -.112^**^ | -.066^*^ | -0.023 | -0.062 | -0.017 | -0.028 | 0.024 | 0.009 | -0.048 |  |
| **Adherence** | .105** | .092** | .076** | .073** | -0.033 | -0.029 | -0.009 | .057* | -0.017 | .124** | 0.041 | -.108** |
