# Supplementary material for: Social distancing in America: Understanding long-term adherence to COVID-19 mitigation recommendations
Source: PLoS One. 2021 Sep 24;16(9):e0257945. doi: 10.1371/journal.pone.0257945 (PMC8462713; doi:10.1371/journal.pone.0257945)
Supplement: S4 Table — May 8–18 (Survey 1. N = 1012). Note. *–Correlation is significant at the .05 level. **–Correlation is significant at the .01 level. (DOCX) [file pone.0257945.s006.docx]

|  | **Knowledge of measures** | **Clarity of measures** | **Perceived health threat** | **Personal costs** | **Punishment certainty** | **Punishment severity** | **Moral alignment** | **Authority response** | **Normative obligation to obey** | **Non-normative obligation to obey** | **Obligation to obey the law (general)** | **Procedural justice** | **Trust in science** | **Trust in media** | **Impulsivity** | **Negative emotions** | **Descriptive social norms** | **Practical capacity to adhere** | **Opportinty to violate** |
| --- | --- | --- | --- | --- | --- | --- | --- | --- | --- | --- | --- | --- | --- | --- | --- | --- | --- | --- | --- |
| **Knowledge of measures** |  |  |  |  |  |  |  |  |  |  |  |  |  |  |  |  |  |  |  |
| **Clarity of measures** | .182** |  |  |  |  |  |  |  |  |  |  |  |  |  |  |  |  |  |  |
| **Perceived health threat** | .150** | .247** |  |  |  |  |  |  |  |  |  |  |  |  |  |  |  |  |  |
| **Personal costs** | 0.026 | 0.035 | .199** |  |  |  |  |  |  |  |  |  |  |  |  |  |  |  |  |
| **Punishment certainty** | 0.027 | .099** | .136** | .195** |  |  |  |  |  |  |  |  |  |  |  |  |  |  |  |
| **Punishment severity** | -.059* | -.096** | -.108** | -.134** | -.228** |  |  |  |  |  |  |  |  |  |  |  |  |  |  |
| **Moral alignment** | .198** | .316** | .565** | .105** | 0.041 | -.059* |  |  |  |  |  |  |  |  |  |  |  |  |  |
| **Authority response** | .062* | .249** | .090** | 0.032 | .226** | -.129** | .096** |  |  |  |  |  |  |  |  |  |  |  |  |
| **Normative obligation to obey** | .154** | .360** | .364** | .097** | .089** | -.077** | .393** | .227** |  |  |  |  |  |  |  |  |  |  |  |
| **Non-normative obligation to obey** | 0.013 | 0.024 | .117** | .198** | .286** | -.233** | 0.010 | .160** | .108** |  |  |  |  |  |  |  |  |  |  |
| **Obligation to obey the law (general)** | .107** | .121** | .115** | -.099** | -.122** | .072** | .199** | -.080** | .183** | -.227** |  |  |  |  |  |  |  |  |  |
| **Procedural justice** | .080** | .272** | .161** | .057* | .084** | -.072** | .209** | .265** | .331** | .046* | -0.011 |  |  |  |  |  |  |  |  |
| **Trust in science** | .114** | .258** | .264** | .075** | .058* | -0.030 | .304** | .085** | .272** | 0.017 | .079** | .171** |  |  |  |  |  |  |  |
| **Trust in media** | 0.052 | .209** | .241** | .057* | .166** | -.070** | .192** | .099** | .219** | .090** | 0.013 | .130** | .367** |  |  |  |  |  |  |
| **Impulsivity** | -.077** | -0.018 | 0.018 | .118** | .179** | -.107** | -.056* | .134** | 0.011 | .241** | -.314** | 0.024 | 0.021 | .120** |  |  |  |  |  |
| **Negative emotions** | 0.047 | 0.007 | .204** | .290** | .077** | -.139** | .101** | -0.002 | .109** | .188** | -.069** | .070** | .105** | .053* | .178** |  |  |  |  |
| **Descriptive social norms** | .127** | .206** | .201** | .083** | .141** | -.135** | .247** | .214** | .277** | .147** | 0.028 | .219** | .166** | .150** | .045* | .087** |  |  |  |
| **Practical capacity to adhere** | .144** | .232** | .297** | .047* | .059* | -.054* | .404** | .115** | .319** | 0.021 | .170** | .220** | .190** | .117** | -.091** | .065** | .411** |  |  |
| **Opportunity to violate** | -0.007 | 0.039 | .070** | .117** | .126** | -.055* | 0.002 | .114** | .047* | .188** | -.171** | .080** | .059** | .078** | .162** | .077** | .110** | 0.014 |  |
| **Adherence** | .161** | .199** | .355** | .066** | .063** | -0.036 | .480** | .047* | .280** | -0.029 | .223** | .144** | .159** | .103** | -.136** | .068** | .283** | .506** | -.048* |
