# Supplementary material for: Social distancing in America: Understanding long-term adherence to COVID-19 mitigation recommendations
Source: PLoS One. 2021 Sep 24;16(9):e0257945. doi: 10.1371/journal.pone.0257945 (PMC8462713; doi:10.1371/journal.pone.0257945)
Supplement: S5 Table — June 8–16 (Survey 2. N = 986). Note. *–Correlation is significant at the .05 level. **–Correlation is significant at the .01 level. (DOCX) [file pone.0257945.s007.docx]

|  | **Knowledge of measures** | **Clarity of measures** | **Perceived health threat** | **Personal costs** | **Punishment certainty** | **Punishment severity** | **Moral alignment** | **Authority response** | **Normative obligation to obey** | **Non-normative obligation to obey** | **Obligation to obey the law (general)** | **Procedural justice** | **Trust in science** | **Trust in media** | **Impulsivity** | **Negative emotions** | **Descriptive social norms** | **Practical capacity to adhere** | **Opportinty to violate** |
| --- | --- | --- | --- | --- | --- | --- | --- | --- | --- | --- | --- | --- | --- | --- | --- | --- | --- | --- | --- |
| **Knowledge of measures** |  |  |  |  |  |  |  |  |  |  |  |  |  |  |  |  |  |  |  |
| **Clarity of measures** | .228** |  |  |  |  |  |  |  |  |  |  |  |  |  |  |  |  |  |  |
| **Perceived health threat** | .182** | .225** |  |  |  |  |  |  |  |  |  |  |  |  |  |  |  |  |  |
| **Personal costs** | 0.040 | -0.038 | .139** |  |  |  |  |  |  |  |  |  |  |  |  |  |  |  |  |
| **Punishment certainty** | 0.046 | .072** | .140** | .212** |  |  |  |  |  |  |  |  |  |  |  |  |  |  |  |
| **Punishment severity** | 0.014 | 0.005 | -.067** | -.206** | -.246** |  |  |  |  |  |  |  |  |  |  |  |  |  |  |
| **Moral alignment** | .213** | .264** | .550** | .067** | 0.023 | -0.007 |  |  |  |  |  |  |  |  |  |  |  |  |  |
| **Authority response** | 0.045 | .301** | .133** | 0.026 | .231** | -.059* | .089** |  |  |  |  |  |  |  |  |  |  |  |  |
| **Normative obligation to obey** | .184** | .313** | .302** | .060* | .096** | -0.029 | .296** | .225** |  |  |  |  |  |  |  |  |  |  |  |
| **Non-normative obligation to obey** | -0.020 | -0.008 | .095** | .199** | .362** | -.224** | 0.000 | .186** | .097** |  |  |  |  |  |  |  |  |  |  |
| **Obligation to obey the law (general)** | .053* | .099** | .060** | -.134** | -.155** | .105** | .144** | -.076** | .106** | -.229** |  |  |  |  |  |  |  |  |  |
| **Procedural justice** | .098** | .199** | .158** | .062** | .128** | -0.016 | .123** | .265** | .302** | .066** | -0.032 |  |  |  |  |  |  |  |  |
| **Trust in science** | .126** | .251** | .291** | .045* | 0.023 | -0.012 | .301** | .117** | .280** | 0.033 | 0.011 | .170** |  |  |  |  |  |  |  |
| **Trust in media** | .129** | .241** | .257** | 0.010 | .169** | -0.028 | .220** | .190** | .201** | .140** | -0.037 | .148** | .332** |  |  |  |  |  |  |
| **Impulsivity** | -0.045 | -.057* | 0.013 | .154** | .230** | -.137** | -.068** | .154** | 0.007 | .278** | -.351** | .101** | 0.028 | .122** |  |  |  |  |  |
| **Negative emotions** | 0.016 | -0.045 | .169** | .297** | .135** | -.178** | .100** | -0.001 | .102** | .197** | -.091** | .051* | .071** | 0.000 | .132** |  |  |  |  |
| **Descriptive social norms** | .114** | .190** | .204** | .094** | .182** | -0.016 | .185** | .242** | .239** | .176** | -0.026 | .209** | .179** | .188** | .097** | .109** |  |  |  |
| **Practical capacity to adhere** | .187** | .238** | .316** | 0.037 | 0.021 | 0.018 | .365** | .120** | .284** | 0.005 | .096** | .160** | .196** | .118** | -.081** | .062** | .362** |  |  |
| **Opportunity to violate** | -0.016 | .055* | .117** | .100** | .173** | -.079** | 0.033 | .146** | .112** | .163** | -.127** | .139** | .099** | .109** | .179** | .094** | .179** | .099** |  |
| **Adherence** | .190** | .203** | .412** | .067** | .048* | -0.017 | .456** | .088** | .225** | 0.023 | .128** | .117** | .198** | .138** | -.087** | .105** | .238** | .481** | 0.033 |
