# Supplementary material for: Social distancing in America: Understanding long-term adherence to COVID-19 mitigation recommendations
Source: PLoS One. 2021 Sep 24;16(9):e0257945. doi: 10.1371/journal.pone.0257945 (PMC8462713; doi:10.1371/journal.pone.0257945)
Supplement: S6 Table — July 11–17 (Survey 3. N = 921). Note. *–Correlation is significant at the .05 level. **–Correlation is significant at the .01 level. (DOCX) [file pone.0257945.s008.docx]

|  | **Knowledge of measures** | **Clarity of measures** | **Perceived health threat** | **Personal costs** | **Punishment certainty** | **Punishment severity** | **Moral alignment** | **Authority response** | **Normative obligation to obey** | **Non-normative obligation to obey** | **Obligation to obey the law**  **(general)** | **Procedural justice** | **Trust in science** | **Trust in media** | **Impulsivity** | **Negative emotions** | **Descriptive social norms** | **Practical capacity to adhere** | **Opportinty to violate** |
| --- | --- | --- | --- | --- | --- | --- | --- | --- | --- | --- | --- | --- | --- | --- | --- | --- | --- | --- | --- |
| **Knowledge of measures** |  |  |  |  |  |  |  |  |  |  |  |  |  |  |  |  |  |  |  |
| **Clarity of measures** | .209^**^ |  |  |  |  |  |  |  |  |  |  |  |  |  |  |  |  |  |  |
| **Perceived health threat** | .114^**^ | .212^**^ |  |  |  |  |  |  |  |  |  |  |  |  |  |  |  |  |  |
| **Personal costs** | 0.002 | 0.008 | .207^**^ |  |  |  |  |  |  |  |  |  |  |  |  |  |  |  |  |
| **Punishment certainty** | 0.036 | .094^**^ | .161^**^ | .227^**^ |  |  |  |  |  |  |  |  |  |  |  |  |  |  |  |
| **Punishment severity** | -.075^*^ | -.062^*^ | -.126^**^ | -.167^**^ | -.260^**^ |  |  |  |  |  |  |  |  |  |  |  |  |  |  |
| **Moral alignment** | .146^**^ | .244^**^ | .545^**^ | .094^**^ | .064^*^ | -0.018 |  |  |  |  |  |  |  |  |  |  |  |  |  |
| **Authority response** | 0.034 | .186^**^ | 0.038 | 0.029 | .336^**^ | -.144^**^ | -0.016 |  |  |  |  |  |  |  |  |  |  |  |  |
| **Normative obligation to obey** | .149^**^ | .325^**^ | .321^**^ | .087^**^ | .114^**^ | -.080^**^ | .362^**^ | .169^**^ |  |  |  |  |  |  |  |  |  |  |  |
| **Non-normative obligation to obey** | -0.019 | -0.019 | .101^**^ | .184^**^ | .266^**^ | -.186^**^ | 0.018 | .198^**^ | .091^**^ |  |  |  |  |  |  |  |  |  |  |
| **Obligation to obey the law (general)** | .071^**^ | .146^**^ | .084^**^ | -.131^**^ | -.147^**^ | .063^**^ | .148^**^ | -.110^**^ | .158^**^ | -.228^**^ |  |  |  |  |  |  |  |  |  |
| **Procedural justice** | .077^**^ | .230^**^ | .185^**^ | .054^*^ | .171^**^ | -.117^**^ | .129^**^ | .261^**^ | .340^**^ | .090^**^ | 0.020 |  |  |  |  |  |  |  |  |
| **Trust in science** | .119^**^ | .277^**^ | .309^**^ | .093^**^ | .062^*^ | -0.048 | .336^**^ | .049^*^ | .327^**^ | 0.013 | .058^*^ | .189^**^ |  |  |  |  |  |  |  |
| **Trust in media** | .060^*^ | .257^**^ | .267^**^ | .109^**^ | .251^**^ | -.101^**^ | .242^**^ | .178^**^ | .233^**^ | .142^**^ | 0.001 | .175^**^ | .354^**^ |  |  |  |  |  |  |
| **Impulsivity** | -.071^*^ | -0.022 | 0.024 | .140^**^ | .230^**^ | -.117^**^ | -0.038 | .202^**^ | -0.010 | .247^**^ | -.321^**^ | .063^**^ | 0.017 | .098^**^ |  |  |  |  |  |
| **Negative emotions** | 0.020 | 0.012 | .230^**^ | .274^**^ | .108^**^ | -.149^**^ | .169^**^ | 0.036 | .120^**^ | .194^**^ | -.093^**^ | .083^**^ | .166^**^ | .100^**^ | .122^**^ |  |  |  |  |
| **Descriptive social norms** | .114^**^ | .232^**^ | .186^**^ | 0.025 | .157^**^ | -0.035 | .208^**^ | .239^**^ | .257^**^ | .104^**^ | -0.001 | .232^**^ | .224^**^ | .206^**^ | 0.034 | 0.037 |  |  |  |
| **Practical capacity to adhere** | .149^**^ | .244^**^ | .300^**^ | .065^**^ | .066^**^ | -0.003 | .372^**^ | .078^**^ | .307^**^ | 0.004 | .102^**^ | .165^**^ | .247^**^ | .162^**^ | -.085^**^ | .077^**^ | .376^**^ |  |  |
| **Opportunity to violate** | 0.027 | 0.029 | .071^**^ | .046^*^ | .109^**^ | -.081^**^ | 0.011 | .143^**^ | .082^**^ | .161^**^ | -.129^**^ | .108^**^ | .066^**^ | .071^**^ | .122^**^ | .066^**^ | .194^**^ | .062^**^ |  |
| **Adherence** | .125^**^ | .211^**^ | .373^**^ | .093^**^ | .054^*^ | -0.014 | .433^**^ | 0.028 | .281^**^ | 0.040 | .131^**^ | .117^**^ | .251^**^ | .185^**^ | -.101^**^ | .098^**^ | .284^**^ | .509^**^ | 0.035 |
