# Supplementary material for: Social distancing in America: Understanding long-term adherence to COVID-19 mitigation recommendations
Source: PLoS One. 2021 Sep 24;16(9):e0257945. doi: 10.1371/journal.pone.0257945 (PMC8462713; doi:10.1371/journal.pone.0257945)
Supplement: S1 Output — (PDF) [file pone.0257945.s009.pdf]

```
      ___  ____  ____  ____  ____(R)
     /__    /   ____/   /   ____/
    ___/   /   /___/   /   /___/
      Statistics/Data Analysis

  1 .
        name:  <unnamed>
         log:  C:\Users\creinde\OneDrive - UvA\RESEARCH\2020\20 03 Coronavirus-measures compliance survey\Data\US\NWO US
    log type:  smcl
   opened on:  17 Jun 2021, 22:50:38

  2 . use "C:\Users\creinde\OneDrive - UvA\RESEARCH\2020\20 03 Coronavirus-measures compliance survey\Data\US\NWO US Summe

  3 .
  4 .
  5 .
  6 . ***************************************************************************************
  7 . ***************************************************************************************
  8 . *A. SELECTION CRITERION:
  9 .
 10 . * - Only if provided consent
 11 . * - No missing data
 12 . * - Both checks correct
 13 . * - Nonbinary gender excluded (insufficient number)
 14 .
 15 . gen chris_sample_reqs = 1 if Consent == 1 & N_Missing == 0 & NChecksRight == 2 & Gender < 3
   (440 missing values generated)

 16 .
 17 . *B. SELECTION CRITERION:
 18 .
 19 . * - Only if provided consent
 20 . * - Both checks correct
 21 .
 22 . *gen chris_sample_reqs = 1 if Consent == 1 & NChecksRight == 2
 23 .
 24 . *C. SELECTION CRITERION:
 25 .
 26 . * - Only if provided consent
 27 . * - No missing data
 28 .
 29 . *gen chris_sample_reqs = 1 if Consent == 1 & N_Missing == 0
 30 .
 31 .
 32 . tab chris_sample_reqs
```

chris_sampl e_reqs	Freq.	Percent	Cum.
1	**1,012**	**100.00**	**100.00**
Total	**1,012**	**100.00**	

```
 33 .
 34 .
 35 . *generate insurance dummies
 36 .
 37 . gen Insurance_Public = 0

 38 . replace Insurance_Public = 1 if (Insurance == 1)
   (412 real changes made)

 39 .
 40 . gen Insurance_Private = 0

 41 . replace Insurance_Private = 1 if (Insurance == 2)
   (826 real changes made)
```

```
. 
. 
. *generate Geographic region dummies
. 
. gen GeoCensus = 0

. replace GeoCensus = 1 if (Province == 8|Province == 22|Province == 24|Province == 32|Province == 44|Province == 50|P
(292 real changes made)

. replace GeoCensus = 2 if (Province == 16|Province == 17|Province == 25|Province == 39|Province == 55|Province == 18|
> 6)
(308 real changes made)

. replace GeoCensus = 3 if (Province == 9|Province == 11|Province == 12|Province == 23|Province == 36|Province == 45|P
> rovince == 47|Province == 5|Province == 21|Province == 40|Province == 48)
(646 real changes made)

. replace GeoCensus = 4 if (Province == 4|Province == 7|Province == 15|Province == 29|Province == 31|Province == 34|Pr
> vince == 53)
(206 real changes made)

. 
. gen GeoCensus_d1 = 0

. gen GeoCensus_d2 = 0

. gen GeoCensus_d3 = 0

. 
. replace GeoCensus_d1 = 1 if (GeoCensus == 2)
(308 real changes made)

. replace GeoCensus_d2 = 1 if (GeoCensus == 3)
(646 real changes made)

. replace GeoCensus_d3 = 1 if (GeoCensus == 4)
(206 real changes made)

. 
. 
. ***************************************************************************************
. ***************************************************************************************
. ***************************************************************************************
. ***************************************************************************************
. 
. *HIERARCHICAL MODEL
. 
. ***************************************************************************************
. ***************************************************************************************
. ***************************************************************************************
. ***************************************************************************************
. 
. *1. Step 1: covariates only
. 
. *1.a.1 Descriptive Statistics
. sum DV_Compliance_SC7  Age i.Gender_Female i.Minority Education i.Employed i.Corona_care i.Insurance_Public i.Insura
> vative_other i.GeoCensus_d1 i.GeoCensus_d2 i.GeoCensus_d3 if chris_sample_reqs == 1
```

Variable	Obs	Mean	Std. Dev.	Min	Max
DV_Complia~7	**1,012**	**6.00974**	**1.197677**	**1**	**7**
Age	**1,012**	**40.31917**	**12.87619**	**17**	**68**
Gender_Fem~e					
0	**1,012**	**.4347826**	**.4959736**	**0**	**1**
1	**1,012**	**.5652174**	**.4959736**	**0**	**1**
Minority					
0	**1,012**	**.6897233**	**.4628355**	**0**	**1**
1	**1,012**	**.3102767**	**.4628355**	**0**	**1**
Education	**1,012**	**3.924901**	**1.497209**	**1**	**8**

Employed					
0	1,012	.3428854	.4749083	0	1
1	1,012	.6571146	.4749083	0	1
Corona_care					
0	1,012	.9318182	.2521823	0	1
1	1,012	.0681818	.2521823	0	1
Insurance_~c					
0	1,012	.7262846	.4460851	0	1
1	1,012	.2737154	.4460851	0	1
Insurance_~e					
0	1,012	.4031621	.4907753	0	1
1	1,012	.5968379	.4907753	0	1
SES_before	1,012	6.049407	1.950305	1	10
SES_change	1,012	-.4397233	1.663396	-9	7
Health_self					
1	1,012	.68083	.4663855	0	1
2	1,012	.31917	.4663855	0	1
Health_other					
1	1,012	.4209486	.4939554	0	1
2	1,012	.5790514	.4939554	0	1
Conservat~01					
0	1,012	.5365613	.498908	0	1
1	1,012	.4634387	.498908	0	1
Conservati~r					
0	1,012	.8754941	.3303211	0	1
1	1,012	.1245059	.3303211	0	1
GeoCensus_d1					
0	1,012	.7865613	.4099374	0	1
1	1,012	.2134387	.4099374	0	1
GeoCensus_d2					
0	1,012	.5573123	.49695	0	1
1	1,012	.4426877	.49695	0	1
GeoCensus_d3					
0	1,012	.8577075	.3495226	0	1
1	1,012	.1422925	.3495226	0	1

```
77 .
78 . *1.a.2 Regression
79 . reg DV_Compliance_SC7  Age i.Gender_Female i.Minority Education i.Employed i.Corona_care i.Insurance_Public i.Insura
   > vative_other i.GeoCensus_d1 i.GeoCensus_d2 i.GeoCensus_d3 if chris_sample_reqs == 1
```

Source	SS	df	MS			
				Number of obs	=	1,012
				F(17, 994)	=	4.50
Model	103.635144	17	6.09618492	Prob > F	=	0.0000
Residual	1346.57497	994	1.35470319	R-squared	=	0.0715
				Adj R-squared	=	0.0556
Total	1450.21011	1,011	1.43443137	Root MSE	=	1.1639

DV_Compliance_SC7	Coef.	Std. Err.	t	P>|t|	[95% Conf.	Interval]
Age	.0096826	.0029675	3.26	0.001	.0038593	.0155059
1.Gender_Female	.2397574	.0757079	3.17	0.002	.0911918	.388323
1.Minority	.099633	.0810822	1.23	0.219	-.0594789	.258745
Education	.0287317	.0279154	1.03	0.304	-.0260482	.0835116
1.Employed	-.1846154	.0861782	-2.14	0.032	-.3537274	-.0155033
1.Corona_care	-.3222999	.152608	-2.11	0.035	-.6217706	-.0228291
1.Insurance_Public	.1072949	.1268594	0.85	0.398	-.141648	.3562379
1.Insurance_Private	.1051034	.1199634	0.88	0.381	-.1303072	.3405141
SES_before	.0459208	.0208296	2.20	0.028	.0050458	.0867958
SES_change	.0075274	.0236549	0.32	0.750	-.038892	.0539467
2.Health_self	.034226	.0864675	0.40	0.692	-.1354539	.2039058
2.Health_other	.2531445	.0798011	3.17	0.002	.0965466	.4097424
1.Conservative_01	-.1769804	.0807769	-2.19	0.029	-.3354932	-.0184675
1.Conservative_other	-.0203891	.1220071	-0.17	0.867	-.2598101	.2190319
1.GeoCensus_d1	-.2450175	.1163195	-2.11	0.035	-.4732775	-.0167575
1.GeoCensus_d2	-.308031	.1001262	-3.08	0.002	-.504514	-.111548
1.GeoCensus_d3	-.2158254	.1280066	-1.69	0.092	-.4670196	.0353688
_cons	5.263274	.2403952	21.89	0.000	4.791534	5.735015

```
. estimates store model_1

.
. *1.a.3 Check hettest: Run this right after your regression to apply the Breusch-Pagan / Cook-Weisberg test for heter
. *if significant, then you need to run the regression with vce(ro) at the end
. estat hettest

Breusch-Pagan / Cook-Weisberg test for heteroskedasticity
         Ho: Constant variance
         Variables: fitted values of DV_Compliance_SC7

         chi2(1)      =    51.78
         Prob > chi2  =   0.0000

.
. *1.a.4. check vif, to check for for multicollinearity (VIFs >10 are problematic)
. vif
```

Variable	VIF	1/VIF
Age	1.09	0.917773
1.Gender_F~e	1.05	0.950372
1.Minority	1.05	0.951456
Education	1.30	0.767079
1.Employed	1.25	0.799979
1.Corona_c~e	1.11	0.904710
1.Insuranc~c	2.39	0.418420
1.Insuran~te	2.59	0.386572
SES_before	1.23	0.811946
SES_change	1.16	0.865481
2.Health_s~f	1.21	0.823942
2.Health_o~r	1.16	0.862383
1.Conserv~01	1.21	0.825045
1.Conserva~r	1.21	0.824993
1.GeoCensu~1	1.70	0.589322
1.GeoCensu~2	1.85	0.541218
1.GeoCensu~3	1.49	0.669388
Mean VIF	1.41	

```
88 .
89 . *1.a.5. Effect size
90 . estat esize
```

Effect sizes for linear models

Source	Eta-Squared	df	[95% Conf.	Interval]
Model	.0714622	17	.0301533	.0880267
Age	.010597	1	.0016856	.0267014
Gender_Female	.0099888	1	.0014463	.025752
Minority	.0015167	1	.	.010108
Education	.0010646	1	.	.00889
Employed	.0045957	1	.	.0166288
Corona_care	.0044672	1	.	.0163876
Insurance_Public	.0007191	1	.	.0078301
Insurance_Private	.0007716	1	.	.008002
SES_before	.0048658	1	.	.0171301
SES_change	.0001019	1	.	.0047546
Health_self	.0001576	1	.	.0052773
Health_other	.0100221	1	.0014591	.0258042
Conservative_01	.0048061	1	.	.0170199
Conservative_other	.0000281	1	.	.0033715
GeoCensus_d1	.0044439	1	.	.0163438
GeoCensus_d2	.0094317	1	.0012373	.024872
GeoCensus_d3	.0028518	1	.	.013178

Note: Eta-Squared values for individual model terms are partial.

```
91 .
92 . *1.a.6 Regression with vce(ro)
93 . reg DV_Compliance_SC7  Age i.Gender_Female i.Minority Education i.Employed i.Corona_care i.Insurance_Public i.Insura
   > vative_other i.GeoCensus_d1 i.GeoCensus_d2 i.GeoCensus_d3 if chris_sample_reqs == 1, vce(ro)
```

```
Linear regression                               Number of obs     =      1,012
                                                F(17, 994)        =       4.87
                                                Prob > F          =     0.0000
                                                R-squared         =     0.0715
                                                Root MSE          =     1.1639
```

DV_Compliance_SC7	Coef.	Robust Std. Err.	t	P>|t|	[95% Conf.	Interval]
Age	.0096826	.0029307	3.30	0.001	.0039315	.0154337
1.Gender_Female	.2397574	.0768992	3.12	0.002	.0888539	.3906609
1.Minority	.099633	.079153	1.26	0.208	-.0556931	.2549592
Education	.0287317	.0282951	1.02	0.310	-.0267933	.0842567
1.Employed	-.1846154	.0853531	-2.16	0.031	-.3521083	-.0171224
1.Corona_care	-.3222999	.1500628	-2.15	0.032	-.6167761	-.0278236
1.Insurance_Public	.1072949	.1336612	0.80	0.422	-.1549956	.3695855
1.Insurance_Private	.1051034	.126575	0.83	0.407	-.1432814	.3534883
SES_before	.0459208	.0212762	2.16	0.031	.0041694	.0876722
SES_change	.0075274	.0243903	0.31	0.758	-.040335	.0553897
2.Health_self	.034226	.0776269	0.44	0.659	-.1181054	.1865573
2.Health_other	.2531445	.0798682	3.17	0.002	.096415	.4098741
1.Conservative_01	-.1769804	.0782946	-2.26	0.024	-.3306221	-.0233386
1.Conservative_other	-.0203891	.1270758	-0.16	0.873	-.2697567	.2289786
1.GeoCensus_d1	-.2450175	.1072221	-2.29	0.023	-.4554251	-.0346098
1.GeoCensus_d2	-.308031	.0927874	-3.32	0.001	-.4901127	-.1259492
1.GeoCensus_d3	-.2158254	.1162455	-1.86	0.064	-.44394	.0122893
_cons	5.263274	.2492547	21.12	0.000	4.774149	5.7524

```
. 
. 
. *****************
. 
. *2. Step 2: Add practical knowledge and understanding
. 
. *2.a.1 Descriptive Statistics
. sum DV_Compliance_SC7  Age i.Gender_Female i.Minority Education i.Employed i.Corona_care i.Insurance_Public i.Insura
> vative_other i.GeoCensus_d1 i.GeoCensus_d2 i.GeoCensus_d3 i.Current_measures Measures_clear if chris_sample_reqs ==
```

Variable	Obs	Mean	Std. Dev.	Min	Max
DV_Complia~7	1,012	6.00974	1.197677	1	7
Age	1,012	40.31917	12.87619	17	68
Gender_Fem~e					
0	1,012	.4347826	.4959736	0	1
1	1,012	.5652174	.4959736	0	1
Minority					
0	1,012	.6897233	.4628355	0	1
1	1,012	.3102767	.4628355	0	1
Education	1,012	3.924901	1.497209	1	8
Employed					
0	1,012	.3428854	.4749083	0	1
1	1,012	.6571146	.4749083	0	1
Corona_care					
0	1,012	.9318182	.2521823	0	1
1	1,012	.0681818	.2521823	0	1
Insurance_~c					
0	1,012	.7262846	.4460851	0	1
1	1,012	.2737154	.4460851	0	1
Insurance_~e					
0	1,012	.4031621	.4907753	0	1
1	1,012	.5968379	.4907753	0	1
SES_before	1,012	6.049407	1.950305	1	10
SES_change	1,012	-.4397233	1.663396	-9	7
Health_self					
1	1,012	.68083	.4663855	0	1
2	1,012	.31917	.4663855	0	1
Health_other					
1	1,012	.4209486	.4939554	0	1
2	1,012	.5790514	.4939554	0	1
Conservat~01					
0	1,012	.5365613	.498908	0	1
1	1,012	.4634387	.498908	0	1
Conservati~r					
0	1,012	.8754941	.3303211	0	1
1	1,012	.1245059	.3303211	0	1
GeoCensus_d1					
0	1,012	.7865613	.4099374	0	1
1	1,012	.2134387	.4099374	0	1
GeoCensus_d2					
0	1,012	.5573123	.49695	0	1
1	1,012	.4426877	.49695	0	1
GeoCensus_d3					

0	1,012	.8577075	.3495226	0	1
1	1,012	.1422925	.3495226	0	1
Current_me~s					
0	1,012	.0958498	.2945307	0	1
Yes	1,012	.9041502	.2945307	0	1
Measures_c~r	1,012	5.36166	1.623161	1	7

```
102 .
103 . *2.a.2 Regression
104 . reg DV_Compliance_SC7  Age i.Gender_Female i.Minority Education i.Employed i.Corona_care i.Insurance_Public i.Insura
    > vative_other i.GeoCensus_d1 i.GeoCensus_d2 i.GeoCensus_d3 i.Current_measures Measures_clear if chris_sample_reqs ==
```

Source	SS	df	MS		
				Number of obs =	1,012
				F(19, 992) =	8.36
Model	200.255414	19	10.5397586	Prob > F =	0.0000
Residual	1249.9547	992	1.26003498	R-squared =	0.1381
				Adj R-squared =	0.1216
Total	1450.21011	1,011	1.43443137	Root MSE =	1.1225

DV_Compliance_SC7	Coef.	Std. Err.	t	P>|t|	[95% Conf.	Interval]
Age	.0062525	.0028893	2.16	0.031	.0005827	.0119222
1.Gender_Female	.214839	.0732336	2.93	0.003	.0711284	.3585496
1.Minority	.0726183	.0782607	0.93	0.354	-.0809573	.226194
Education	.0486639	.0270255	1.80	0.072	-.0043698	.1016977
1.Employed	-.1882241	.0831366	-2.26	0.024	-.3513678	-.0250804
1.Corona_care	-.3341624	.147194	-2.27	0.023	-.6230097	-.0453151
1.Insurance_Public	.0789393	.1225834	0.64	0.520	-.1616133	.3194919
1.Insurance_Private	.1057804	.1160518	0.91	0.362	-.1219549	.3335157
SES_before	.0287187	.0201858	1.42	0.155	-.010893	.0683304
SES_change	.0021096	.0228219	0.09	0.926	-.0426752	.0468944
2.Health_self	.0595517	.0834534	0.71	0.476	-.1042137	.2233172
2.Health_other	.195308	.0772831	2.53	0.012	.0436507	.3469652
1.Conservative_01	-.153734	.0779509	-1.97	0.049	-.3067016	-.0007663
1.Conservative_other	.0271126	.1177972	0.23	0.818	-.2040477	.258273
1.GeoCensus_d1	-.1956876	.112345	-1.74	0.082	-.4161488	.0247736
1.GeoCensus_d2	-.2499394	.0968591	-2.58	0.010	-.4400116	-.0598673
1.GeoCensus_d3	-.152208	.1238267	-1.23	0.219	-.3952004	.0907844
Current_measures						
Yes	.7444765	.1252405	5.94	0.000	.4987098	.9902431
Measures_clear	.1154276	.0225309	5.12	0.000	.0712138	.1596414
_cons	4.129446	.2658322	15.53	0.000	3.607788	4.651104

```
105 . estimates store model_2

106 .
107 . *2.a.3 Check hettest: Run this right after your regression to apply the Breusch-Pagan / Cook-Weisberg test for heter
108 . *if significant, then you need to run the regression with vce(ro) at the end
109 . estat hettest

    Breusch-Pagan / Cook-Weisberg test for heteroskedasticity
             Ho: Constant variance
             Variables: fitted values of DV_Compliance_SC7

             chi2(1)      =    94.69
             Prob > chi2  =   0.0000
```

110 .
111 . *2.a.4. check vif, to check for for multicollinearity (VIFs >10 are problematic)
112 . vif

Variable	VIF	1/VIF
Age	1.11	0.900496
1.Gender_F~e	1.06	0.944699
1.Minority	1.05	0.949927
Education	1.31	0.761235
1.Employed	1.25	0.799517
1.Corona_c~e	1.11	0.904528
1.Insuranc~c	2.40	0.416805
1.Insuran~te	2.60	0.384204
SES_before	1.24	0.804146
SES_change	1.16	0.864839
2.Health_s~f	1.22	0.822722
2.Health_o~r	1.17	0.855237
1.Conserv~01	1.21	0.824039
1.Conserva~r	1.21	0.823168
1.GeoCensu~1	1.70	0.587609
1.GeoCensu~2	1.86	0.537930
1.GeoCensu~3	1.50	0.665353
1.Current_~s	1.09	0.915970
Measures_c~r	1.07	0.931858
Mean VIF	1.39	

113 .
114 . *2.a.5. Effect size
115 . estat esize

Effect sizes for linear models

Source	Eta-Squared	df	[95% Conf.	Interval]
Model	.1380872	19	.0858946	.1615885
Age	.0046986	1	.	.0168363
Gender_Female	.0086009	1	.0009414	.0235578
Minority	.0008672	1	.	.0083147
Education	.0032579	1	.	.0140366
Employed	.0051406	1	.0000174	.0176494
Corona_care	.0051686	1	.0000231	.0177003
Insurance_Public	.0004179	1	.	.0067226
Insurance_Private	.0008368	1	.	.0082202
SES_before	.0020363	1	.	.0113848
SES_change	8.61e-06	1	.	.0021749
Health_self	.0005131	1	.	.0071075
Health_other	.0063969	1	.0002982	.0198756
Conservative_01	.0039056	1	.	.0153278
Conservative_other	.0000534	1	.	.0040485
GeoCensus_d1	.0030492	1	.	.0136063
GeoCensus_d2	.0066676	1	.0003665	.0203415
GeoCensus_d3	.0015208	1	.	.0101307
Current_measures	.0343954	1	.0155743	.0593626
Measures_clear	.0257756	1	.009877	.0481976

Note: Eta-Squared values for individual model terms are partial.

116 .

```
117 . *2.a.6 Regression with vce(ro)
118 . reg DV_Compliance_SC7  Age i.Gender_Female i.Minority Education i.Employed i.Corona_care i.Insurance_Public i.Insura
    > vative_other i.GeoCensus_d1 i.GeoCensus_d2 i.GeoCensus_d3 i.Current_measures Measures_clear if chris_sample_reqs ==
```

Linear regression

Number of obs	=	1,012
F(19, 992)	=	7.31
Prob > F	=	0.0000
R-squared	=	0.1381
Root MSE	=	1.1225

DV_Compliance_SC7	Coef.	Robust Std. Err.	t	P>|t|	[95% Conf.	Interval]
Age	.0062525	.0029115	2.15	0.032	.000539	.0119659
1.Gender_Female	.214839	.0750247	2.86	0.004	.0676136	.3620644
1.Minority	.0726183	.0760379	0.96	0.340	-.0765952	.2218319
Education	.0486639	.0273437	1.78	0.075	-.0049942	.1023221
1.Employed	-.1882241	.0834843	-2.25	0.024	-.3520503	-.0243979
1.Corona_care	-.3341624	.1480642	-2.26	0.024	-.6247174	-.0436074
1.Insurance_Public	.0789393	.1293201	0.61	0.542	-.1748331	.3327117
1.Insurance_Private	.1057804	.1223902	0.86	0.388	-.134393	.3459538
SES_before	.0287187	.0203118	1.41	0.158	-.0111403	.0685777
SES_change	.0021096	.0234644	0.09	0.928	-.043936	.0481551
2.Health_self	.0595517	.0758261	0.79	0.432	-.0892462	.2083497
2.Health_other	.195308	.0771241	2.53	0.011	.0439628	.3466531
1.Conservative_01	-.153734	.0752984	-2.04	0.041	-.3014964	-.0059716
1.Conservative_other	.0271126	.1230213	0.22	0.826	-.2142992	.2685245
1.GeoCensus_d1	-.1956876	.1050137	-1.86	0.063	-.4017622	.010387
1.GeoCensus_d2	-.2499394	.0898264	-2.78	0.005	-.426211	-.0736679
1.GeoCensus_d3	-.152208	.1125099	-1.35	0.176	-.3729927	.0685768
Current_measures						
Yes	.7444765	.1694615	4.39	0.000	.4119323	1.077021
Measures_clear	.1154276	.0257697	4.48	0.000	.0648583	.1659969
_cons	4.129446	.2845111	14.51	0.000	3.571133	4.687759

```
119 .
120 .
121 . *****************
122 .
123 . *3. Step 3: Add costs + benefits
124 .
125 . *3.a.1 Descriptive Statistics
126 . sum DV_Compliance_SC7  Age i.Gender_Female i.Minority Education i.Employed i.Corona_care i.Insurance_Public i.Insura
    > vative_other i.GeoCensus_d1 i.GeoCensus_d2 i.GeoCensus_d3 i.Current_measures Measures_clear MA_Perc_Threat_SC3 Costs
```

Variable	Obs	Mean	Std. Dev.	Min	Max
DV_Complia~7	1,012	6.00974	1.197677	1	7
Age	1,012	40.31917	12.87619	17	68
Gender_Fem~e					
0	1,012	.4347826	.4959736	0	1
1	1,012	.5652174	.4959736	0	1
Minority					
0	1,012	.6897233	.4628355	0	1
1	1,012	.3102767	.4628355	0	1
Education	1,012	3.924901	1.497209	1	8
Employed					
0	1,012	.3428854	.4749083	0	1
1	1,012	.6571146	.4749083	0	1
Corona_care					
0	1,012	.9318182	.2521823	0	1
1	1,012	.0681818	.2521823	0	1
Insurance_~c					

```
          0 |      1,012    .7262846    .4460851          0          1
          1 |      1,012    .2737154    .4460851          0          1
            |
Insurance_~e |
          0 |      1,012    .4031621    .4907753          0          1
          1 |      1,012    .5968379    .4907753          0          1
-------------+---------------------------------------------------------
            |
  SES_before |      1,012    6.049407    1.950305          1         10
  SES_change |      1,012   -.4397233    1.663396         -9          7
            |
 Health_self |
          1 |      1,012      .68083    .4663855          0          1
          2 |      1,012      .31917    .4663855          0          1
            |
Health_other |
          1 |      1,012    .4209486    .4939554          0          1
-------------+---------------------------------------------------------
          2 |      1,012    .5790514    .4939554          0          1
            |
Conservat~01 |
          0 |      1,012    .5365613     .498908          0          1
          1 |      1,012    .4634387     .498908          0          1
            |
Conservati~r |
          0 |      1,012    .8754941    .3303211          0          1
          1 |      1,012    .1245059    .3303211          0          1
-------------+---------------------------------------------------------
            |
 GeoCensus_d1 |
          0 |      1,012    .7865613    .4099374          0          1
          1 |      1,012    .2134387    .4099374          0          1
            |
 GeoCensus_d2 |
          0 |      1,012    .5573123      .49695          0          1
          1 |      1,012    .4426877      .49695          0          1
            |
 GeoCensus_d3 |
          0 |      1,012    .8577075    .3495226          0          1
-------------+---------------------------------------------------------
          1 |      1,012    .1422925    .3495226          0          1
            |
Current_me~s |
          0 |      1,012    .0958498    .2945307          0          1
        Yes |      1,012    .9041502    .2945307          0          1
            |
Measures_c~r |      1,012     5.36166    1.623161          1          7
MA_Perc_Th~3 |      1,012    5.596509    1.470155          1          7
-------------+---------------------------------------------------------
   Costs_SC5 |      1,012    4.308696    1.616251          1          7
Deterr_SD_~2 |      1,012    3.340909    1.762026          1          7
Deterr_SD_~e |      1,012    3.800395    1.698804          1          6

127 .
128 . *3.a.2 Regression
129 . reg DV_Compliance_SC7  Age i.Gender_Female i.Minority Education i.Employed i.Corona_care i.Insurance_Public i.Insura
    > vative_other i.GeoCensus_d1 i.GeoCensus_d2 i.GeoCensus_d3 i.Current_measures Measures_clear MA_Perc_Threat_SC3 Costs

      Source |       SS           df       MS      Number of obs   =     1,012
-------------+----------------------------------   F(23, 988)      =     17.51
       Model |   419.88426        23  18.2558374   Prob > F        =    0.0000
    Residual |  1030.32585       988  1.04283993   R-squared       =    0.2895
-------------+----------------------------------   Adj R-squared   =    0.2730
       Total |  1450.21011     1,011  1.43443137   Root MSE        =    1.0212
```

DV_Compliance_SC7	Coef.	Std. Err.	t	P>|t|	[95% Conf.	Interval]
Age	.0067644	.0026809	2.52	0.012	.0015036	.0120252
1.Gender_Female	.1985525	.066866	2.97	0.003	.0673367	.3297683
1.Minority	-.0562001	.0724182	-0.78	0.438	-.1983113	.0859111
Education	.0613508	.024656	2.49	0.013	.0129667	.1097349
1.Employed	-.1728668	.0757203	-2.28	0.023	-.3214579	-.0242757
1.Corona_care	-.4539415	.1370192	-3.31	0.001	-.7228236	-.1850593
1.Insurance_Public	.0402011	.111898	0.36	0.719	-.1793841	.2597862
1.Insurance_Private	.0745686	.1063436	0.70	0.483	-.1341168	.283254
SES_before	.0322691	.0185996	1.73	0.083	-.0042302	.0687684
SES_change	.0374154	.0214772	1.74	0.082	-.0047308	.0795617
2.Health_self	-.1227606	.0770886	-1.59	0.112	-.2740369	.0285157
2.Health_other	.0932653	.071463	1.31	0.192	-.0469713	.2335019
1.Conservative_01	.0942191	.0731908	1.29	0.198	-.0494081	.2378464
1.Conservative_other	.164577	.1077201	1.53	0.127	-.0468095	.3759635
1.GeoCensus_d1	-.0984539	.1024594	-0.96	0.337	-.2995169	.1026091
1.GeoCensus_d2	-.2368721	.0882093	-2.69	0.007	-.4099711	-.063773
1.GeoCensus_d3	-.1408697	.1128178	-1.25	0.212	-.3622598	.0805204
Current_measures						
Yes	.5222445	.1150906	4.54	0.000	.2963944	.7480946
Measures_clear	.037729	.0212979	1.77	0.077	-.0040653	.0795234
MA_Perc_Threat_SC3	.3549622	.0256559	13.84	0.000	.304616	.4053084
Costs_SC5	.0166275	.0227318	0.73	0.465	-.0279806	.0612357
Deterr_SD_Likely_SC2	.0011627	.0207453	0.06	0.955	-.0395473	.0418726
Deterr_SD_Severe	-.0096188	.0203707	-0.47	0.637	-.0495936	.0303561
_cons	2.678641	.3045728	8.79	0.000	2.080957	3.276325

```
130 . estimates store model_3

131 .
132 . *3.a.3 Check hettest: Run this right after your regression to apply the Breusch-Pagan / Cook-Weisberg test for heter
133 . *if significant, then you need to run the regression with vce(ro) at the end
134 . estat hettest

    Breusch-Pagan / Cook-Weisberg test for heteroskedasticity
             Ho: Constant variance
             Variables: fitted values of DV_Compliance_SC7

             chi2(1)      =   123.77
             Prob > chi2  =   0.0000

135 .
136 . *3.a.4. check vif, to check for for multicollinearity (VIFs >10 are problematic)
137 . vif
```

Variable	VIF	1/VIF
Age	1.16	0.865654
1.Gender_F~e	1.07	0.937861
1.Minority	1.09	0.918158
Education	1.32	0.756934
1.Employed	1.25	0.797668
1.Corona_c~e	1.16	0.863922
1.Insuranc~c	2.42	0.413986
1.Insuran~te	2.64	0.378685
SES_before	1.28	0.783885
SES_change	1.24	0.808198
2.Health_s~f	1.25	0.797986
2.Health_o~r	1.21	0.827807
1.Conserv~01	1.29	0.773593
1.Conserva~r	1.23	0.814705
1.GeoCensu~1	1.71	0.584693
1.GeoCensu~2	1.86	0.536800
1.GeoCensu~3	1.51	0.663377
1.Current_~s	1.11	0.897688
Measures_c~r	1.16	0.863116
MA_Perc_Th~3	1.38	0.725049
Costs_SC5	1.31	0.764154
Deterr_SD_~2	1.30	0.771973
Deterr_SD_~e	1.16	0.861325

Mean VIF	1.40

138 .
139 . *3.a.5. Effect size
140 . estat esize

Effect sizes for linear models

Source	Eta-Squared	df	[95% Conf.	Interval]
Model	.2895334	23	.2285605	.3173307
Age	.0064027	1	.0002944	.0199199
Gender_Female	.0088455	1	.0010165	.0239905
Minority	.0006092	1	.	.0074842
Education	.0062277	1	.000252	.0196161
Employed	.0052475	1	.000035	.0178761
Corona_care	.0109871	1	.0018286	.0273639
Insurance_Public	.0001306	1	.	.0050699
Insurance_Private	.0004974	1	.	.0070672
SES_before	.0030373	1	.	.0136102
SES_change	.0030624	1	.	.0136624
Health_self	.0025602	1	.	.0125927
Health_other	.001721	1	.	.0106566
Conservative_01	.0016745	1	.	.0105419
Conservative_other	.002357	1	.	.0121436
GeoCensus_d1	.0009337	1	.	.0085401
GeoCensus_d2	.0072458	1	.0005164	.0213579
GeoCensus_d3	.0015756	1	.	.0102946
Current_measures	.0204152	1	.006609	.0410109
Measures_clear	.0031662	1	.	.0138774
MA_Perc_Threat_SC3	.1623012	1	.1229311	.2031122
Costs_SC5	.0005412	1	.	.0072354
Deterr_SD_Likely_SC2	3.18e-06	1	.	.001173
Deterr_SD_Severe	.0002256	1	.	.0057758

Note: Eta-Squared values for individual model terms are partial.

141 .
142 . *3.a.6 Regression with vce(ro)
143 . reg DV_Compliance_SC7 Age i.Gender_Female i.Minority Education i.Employed i.Corona_care i.Insurance_Public i.Insura
> vative_other i.GeoCensus_d1 i.GeoCensus_d2 i.GeoCensus_d3 i.Current_measures Measures_clear MA_Perc_Threat_SC3 Costs

Linear regression

Number of obs	=	1,012
F(23, 988)	=	14.82
Prob > F	=	0.0000
R-squared	=	0.2895
Root MSE	=	1.0212

DV_Compliance_SC7	Coef.	Robust Std. Err.	t	P>|t|	[95% Conf.	Interval]
Age	.0067644	.0026757	2.53	0.012	.0015137	.0120151
1.Gender_Female	.1985525	.068594	2.89	0.004	.0639459	.3331591
1.Minority	-.0562001	.0714716	-0.79	0.432	-.1964536	.0840535
Education	.0613508	.024044	2.55	0.011	.0141676	.108534
1.Employed	-.1728668	.0761627	-2.27	0.023	-.3223261	-.0234074
1.Corona_care	-.4539415	.1344191	-3.38	0.001	-.7177212	-.1901617
1.Insurance_Public	.0402011	.1205441	0.33	0.739	-.1963508	.2767529
1.Insurance_Private	.0745686	.1165764	0.64	0.523	-.1541972	.3033344
SES_before	.0322691	.0191623	1.68	0.093	-.0053344	.0698726
SES_change	.0374154	.0217734	1.72	0.086	-.0053119	.0801428
2.Health_self	-.1227606	.0699769	-1.75	0.080	-.260081	.0145598
2.Health_other	.0932653	.0687433	1.36	0.175	-.0416344	.228165
1.Conservative_01	.0942191	.0735832	1.28	0.201	-.0501781	.2386164
1.Conservative_other	.164577	.1135111	1.45	0.147	-.0581735	.3873275
1.GeoCensus_d1	-.0984539	.0985668	-1.00	0.318	-.2918783	.0949705
1.GeoCensus_d2	-.2368721	.0844311	-2.81	0.005	-.4025568	-.0711873
1.GeoCensus_d3	-.1408697	.1027999	-1.37	0.171	-.342601	.0608616
Current_measures						

Yes	.5222445	.1474208	3.54	0.000	.2329506	.8115384
Measures_clear	.037729	.0220512	1.71	0.087	-.0055435	.0810015
MA_Perc_Threat_SC3	.3549622	.029389	12.08	0.000	.2972902	.4126342
Costs_SC5	.0166275	.0253966	0.65	0.513	-.0332099	.066465
Deterr_SD_Likely_SC2	.0011627	.0206407	0.06	0.955	-.039342	.0416673
Deterr_SD_Severe	-.0096188	.0203046	-0.47	0.636	-.0494638	.0302262
_cons	2.678641	.3095504	8.65	0.000	2.071189	3.286093

```
144 .
145 .
146 . *****************
147 .
148 . *4. Step 4: Add legitimacy
149 .
150 . *4.a.1 Descriptive Statistics
151 . sum DV_Compliance_SC7  Age i.Gender_Female i.Minority Education i.Employed i.Corona_care i.Insurance_Public i.Insura
    > vative_other i.GeoCensus_d1 i.GeoCensus_d2 i.GeoCensus_d3 i.Current_measures Measures_clear MA_Perc_Threat_SC3 Costs
    > C3 OOL_SC12 PJE_SC4 if chris_sample_reqs == 1
```

Variable	Obs	Mean	Std. Dev.	Min	Max
DV_Complia~7	1,012	6.00974	1.197677	1	7
Age	1,012	40.31917	12.87619	17	68
Gender_Fem~e					
0	1,012	.4347826	.4959736	0	1
1	1,012	.5652174	.4959736	0	1
Minority					
0	1,012	.6897233	.4628355	0	1
1	1,012	.3102767	.4628355	0	1
Education	1,012	3.924901	1.497209	1	8
Employed					
0	1,012	.3428854	.4749083	0	1
1	1,012	.6571146	.4749083	0	1
Corona_care					
0	1,012	.9318182	.2521823	0	1
1	1,012	.0681818	.2521823	0	1
Insurance_~c					
0	1,012	.7262846	.4460851	0	1
1	1,012	.2737154	.4460851	0	1
Insurance_~e					
0	1,012	.4031621	.4907753	0	1
1	1,012	.5968379	.4907753	0	1
SES_before	1,012	6.049407	1.950305	1	10
SES_change	1,012	-.4397233	1.663396	-9	7
Health_self					
1	1,012	.68083	.4663855	0	1
2	1,012	.31917	.4663855	0	1
Health_other					
1	1,012	.4209486	.4939554	0	1
2	1,012	.5790514	.4939554	0	1
Conservat~01					
0	1,012	.5365613	.498908	0	1
1	1,012	.4634387	.498908	0	1
Conservati~r					
0	1,012	.8754941	.3303211	0	1
1	1,012	.1245059	.3303211	0	1

```
GeoCensus_d1
          0 |      1,012    .7865613    .4099374          0          1
          1 |      1,012    .2134387    .4099374          0          1
            |
GeoCensus_d2
          0 |      1,012    .5573123      .49695          0          1
          1 |      1,012    .4426877      .49695          0          1
            |
GeoCensus_d3
          0 |      1,012    .8577075    .3495226          0          1
------------+-----------------------------------------------------
          1 |      1,012    .1422925    .3495226          0          1
            |
Current_me~s
          0 |      1,012    .0958498    .2945307          0          1
        Yes |      1,012    .9041502    .2945307          0          1
            |
Measures_c~r |     1,012     5.36166    1.623161          1          7
MA_Perc_Th~3 |     1,012    5.596509    1.470155          1          7
------------+-----------------------------------------------------
   Costs_SC5 |     1,012    4.308696    1.616251          1          7
Deterr_SD_~2 |     1,012    3.340909    1.762026          1          7
Deterr_SD_~e |     1,012    3.800395    1.698804          1          6
MA_MoralBe~f |     1,012    6.211462    1.183779          1          7
MA_Authori~2 |     1,012     4.29002    1.849594          1          7
------------+-----------------------------------------------------
     NOO_SC3 |     1,012    3.969368    .8523516          1          5
    NNOO_SC3 |     1,012    2.953228     .989675          1          5
   OOL_SC12 |      1,012    4.397892    1.459976          1          7
     PJE_SC4 |     1,012    5.240613    1.513755          1          7

152 .
153 . *4.a.2 Regression
154 . reg DV_Compliance_SC7  Age i.Gender_Female i.Minority Education i.Employed i.Corona_care i.Insurance_Public i.Insura
    > vative_other i.GeoCensus_d1 i.GeoCensus_d2 i.GeoCensus_d3 i.Current_measures Measures_clear MA_Perc_Threat_SC3 Costs
    > C3 OOL_SC12 PJE_SC4 if chris_sample_reqs == 1

      Source |       SS           df       MS      Number of obs   =     1,012
-------------+----------------------------------   F(29, 982)      =     21.93
       Model |   570.02991        29  19.6562038   Prob > F        =    0.0000
    Residual |  880.180202       982  .896313851   R-squared       =    0.3931
-------------+----------------------------------   Adj R-squared   =    0.3751
       Total |  1450.21011     1,011  1.43443137   Root MSE        =    .94674

--------------------------------------------------------------------------------------
   DV_Compliance_SC7 |      Coef.   Std. Err.      t    P>|t|     [95% Conf. Interval]
---------------------+----------------------------------------------------------------
                 Age |   .0053622   .0025236     2.12   0.034       .00041    .0103144
     1.Gender_Female |   .1391204   .0625532     2.22   0.026     .0163671    .2618736
          1.Minority |  -.0704702   .0672637    -1.05   0.295    -.2024674    .0615269
           Education |   .0451958   .0231095     1.96   0.051     -.000154    .0905455
          1.Employed |   -.121994    .070497    -1.73   0.084    -.2603361    .0163481
       1.Corona_care |  -.3222549   .1284959    -2.51   0.012     -.574413   -.0700967
  1.Insurance_Public |   .0019397   .1039172     0.02   0.985    -.2019856    .2058649
 1.Insurance_Private |   .0516394   .0987455     0.52   0.601    -.1421371    .2454159
          SES_before |    .029451    .017709     1.66   0.097    -.0053009    .0642029
          SES_change |   .0358676   .0202725     1.77   0.077    -.0039148    .0756499
       2.Health_self |  -.0130794   .0720275    -0.18   0.856    -.1544249    .1282661
      2.Health_other |   .0764487   .0670133     1.14   0.254    -.0550572    .2079545
   1.Conservative_01 |   .1175005   .0703962     1.67   0.095    -.0206438    .2556448
1.Conservative_other |   .1885466   .1008198     1.87   0.062    -.0093004    .3863936
      1.GeoCensus_d1 |  -.0755154   .0955225    -0.79   0.429     -.262967    .1119363
      1.GeoCensus_d2 |  -.1744445   .0822127    -2.12   0.034    -.3357774   -.0131117
      1.GeoCensus_d3 |  -.0512545   .1052219    -0.49   0.626    -.2577401    .1552311
                     |
    Current_measures |
                 Yes |   .3467584   .1077436     3.22   0.001     .1353244    .5581925
      Measures_clear |  -.0106353   .0217059    -0.49   0.624    -.0532305    .0319599
  MA_Perc_Threat_SC3 |   .1150109   .0314388     3.66   0.000      .053316    .1767059
           Costs_SC5 |   .0380501   .0215276     1.77   0.077    -.0041952    .0802955
 Deterr_SD_Likely_SC2 |   .0334136   .0203441     1.64   0.101    -.0065095    .0733366
    Deterr_SD_Severe |  -.0290755   .0192045    -1.51   0.130    -.0667622    .0086111
      MA_MoralBelief |   .4124454   .0372133    11.08   0.000     .3394188    .4854721
```

MA_Authority_SC2	-.0120668	.0198845	-0.61	0.544	-.0510879	.0269543
NOO_SC3	.0415941	.0472862	0.88	0.379	-.0511996	.1343878
NNOO_SC3	-.0579989	.0375412	-1.54	0.123	-.1316691	.0156713
OOL_SC12	.07835	.0248487	3.15	0.002	.0295872	.1271127
PJE_SC4	.010141	.0230361	0.44	0.660	-.0350648	.0553467
_cons	1.483492	.3296998	4.50	0.000	.8364953	2.13049

```
. estimates store model_4

.
. *4.a.3 Check hettest: Run this right after your regression to apply the Breusch-Pagan / Cook-Weisberg test for heter
. *if significant, then you need to run the regression with vce(ro) at the end
. estat hettest
```

Breusch-Pagan / Cook-Weisberg test for heteroskedasticity
Ho: Constant variance
Variables: fitted values of DV_Compliance_SC7

chi2(**1**) = **176.04**
Prob > chi2 = **0.0000**

```
.
. *4.a.4. check vif, to check for for multicollinearity (VIFs >10 are problematic)
. vif
```

Variable	VIF	1/VIF
Age	1.19	0.839663
1.Gender_F~e	1.09	0.921071
1.Minority	1.09	0.914732
Education	1.35	0.740562
1.Employed	1.26	0.790949
1.Corona_c~e	1.18	0.844308
1.Insuranc~c	2.42	0.412571
1.Insuran~te	2.65	0.377493
SES_before	1.35	0.743214
SES_change	1.28	0.779656
2.Health_s~f	1.27	0.785638
2.Health_o~r	1.24	0.809116
1.Conserv~01	1.39	0.718736
1.Conserva~r	1.25	0.799365
1.GeoCensu~1	1.73	0.578179
1.GeoCensu~2	1.88	0.531135
1.GeoCensu~3	1.53	0.655460
1.Current_~s	1.14	0.880370
Measures_c~r	1.40	0.714218
MA_Perc_Th~3	2.41	0.415003
Costs_SC5	1.37	0.732319
Deterr_SD_~2	1.45	0.689931
Deterr_SD_~e	1.20	0.832942
MA_MoralBe~f	2.19	0.456848
MA_Authori~2	1.53	0.655428
NOO_SC3	1.83	0.545760
NNOO_SC3	1.56	0.642256
OOL_SC12	1.48	0.673611
PJE_SC4	1.37	0.729085
Mean VIF	1.52	

```
.
. *4.a.5. Effect size
```

```
165 . estat esize
```

Effect sizes for linear models

Source	Eta-Squared	df	[95% Conf.	Interval]
Model	.3930671	29	.3307226	.4175884
Age	.0045768	1	.	.0166881
Gender_Female	.0050118	1	.	.0174951
Minority	.0011165	1	.	.0091083
Education	.0038798	1	.	.0153537
Employed	.0030402	1	.	.0136596
Corona_care	.0063641	1	.0002772	.0199048
Insurance_Public	3.55e-07	1	.	.
Insurance_Private	.0002784	1	.	.0061073
SES_before	.0028085	1	.	.0131709
SES_change	.0031776	1	.	.0139446
Health_self	.0000336	1	.	.003586
Health_other	.0013235	1	.	.0096775
Conservative_01	.002829	1	.	.0132146
Conservative_other	.0035489	1	.	.0146988
GeoCensus_d1	.000636	1	.	.0076113
GeoCensus_d2	.0045639	1	.	.016664
GeoCensus_d3	.0002416	1	.	.0058989
Current_measures	.0104377	1	.0015914	.0265716
Measures_clear	.0002444	1	.	.0059157
MA_Perc_Threat_SC3	.0134449	1	.0028965	.0311421
Costs_SC5	.0031713	1	.	.0139315
Deterr_SD_Likely_SC2	.0027395	1	.	.0130231
Deterr_SD_Severe	.0023288	1	.	.0121212
MA_MoralBelief	.111183	1	.0770004	.1485198
MA_Authority_SC2	.0003749	1	.	.0065828
NOO_SC3	.0007873	1	.	.0081189
NNOO_SC3	.0024247	1	.	.0123357
OOL_SC12	.0100227	1	.0014304	.0259217
PJE_SC4	.0001973	1	.	.0056186

Note: Eta-Squared values for individual model terms are partial.

```
166 .
167 . *4.a.6 Regression with vce(ro)
168 . reg DV_Compliance_SC7  Age i.Gender_Female i.Minority Education i.Employed i.Corona_care i.Insurance_Public i.Insura
    > vative_other i.GeoCensus_d1 i.GeoCensus_d2 i.GeoCensus_d3 i.Current_measures Measures_clear MA_Perc_Threat_SC3 Costs
    > C3 OOL_SC12 PJE_SC4 if chris_sample_reqs == 1, vce(ro)
```

Linear regression

Number of obs	=	1,012
F(29, 982)	=	17.00
Prob > F	=	0.0000
R-squared	=	0.3931
Root MSE	=	.94674

DV_Compliance_SC7	Coef.	Robust Std. Err.	t	P>|t|	[95% Conf.	Interval]
Age	.0053622	.002615	2.05	0.041	.0002306	.0104939
1.Gender_Female	.1391204	.0657582	2.12	0.035	.0100776	.2681631
1.Minority	-.0704702	.065273	-1.08	0.281	-.1985609	.0576204
Education	.0451958	.0218602	2.07	0.039	.0022978	.0880938
1.Employed	-.121994	.0711853	-1.71	0.087	-.2616868	.0176988
1.Corona_care	-.3222549	.1381782	-2.33	0.020	-.5934134	-.0510963
1.Insurance_Public	.0019397	.1150418	0.02	0.987	-.2238164	.2276958
1.Insurance_Private	.0516394	.1120924	0.46	0.645	-.1683287	.2716075
SES_before	.029451	.0191336	1.54	0.124	-.0080965	.0669985
SES_change	.0358676	.0212382	1.69	0.092	-.0058099	.077545
2.Health_self	-.0130794	.0654126	-0.20	0.842	-.1414441	.1152852
2.Health_other	.0764487	.0646657	1.18	0.237	-.0504503	.2033476
1.Conservative_01	.1175005	.0758079	1.55	0.121	-.0312637	.2662646
1.Conservative_other	.1885466	.1016608	1.85	0.064	-.0109508	.3880439
1.GeoCensus_d1	-.0755154	.0875477	-0.86	0.389	-.2473175	.0962867
1.GeoCensus_d2	-.1744445	.072675	-2.40	0.017	-.3170606	-.0318284
1.GeoCensus_d3	-.0512545	.0903208	-0.57	0.571	-.2284984	.1259894

Current_measures						
Yes	.3467584	.1324219	2.62	0.009	.0868961	.6066208
Measures_clear	-.0106353	.0211312	-0.50	0.615	-.0521027	.0308321
MA_Perc_Threat_SC3	.1150109	.035986	3.20	0.001	.0443927	.1856292
Costs_SC5	.0380501	.0232824	1.63	0.103	-.0076388	.0837391
Deterr_SD_Likely_SC2	.0334136	.0199676	1.67	0.095	-.0057706	.0725977
Deterr_SD_Severe	-.0290755	.0193564	-1.50	0.133	-.0670601	.0089091
MA_MoralBelief	.4124454	.0461803	8.93	0.000	.3218221	.5030688
MA_Authority_SC2	-.0120668	.0191429	-0.63	0.529	-.0496324	.0254989
NOO_SC3	.0415941	.0483233	0.86	0.390	-.0532347	.1364229
NNOO_SC3	-.0579989	.0394203	-1.47	0.142	-.1353567	.0193589
OOL_SC12	.07835	.0268304	2.92	0.004	.0256984	.1310015
PJE_SC4	.010141	.0239387	0.42	0.672	-.0368359	.0571178
_cons	1.483492	.3375389	4.40	0.000	.8211121	2.145873

```
. 
. 
. *****************
. 
. *5. Step 5: Add personal factors
. 
. *5.a.1 Descriptive Statistics
. sum DV_Compliance_SC7  Age i.Gender_Female i.Minority Education i.Employed i.Corona_care i.Insurance_Public i.Insura
> vative_other i.GeoCensus_d1 i.GeoCensus_d2 i.GeoCensus_d3 i.Current_measures Measures_clear MA_Perc_Threat_SC3 Costs
> C3 OOL_SC12 PJE_SC4 Trust_Science_SC4 Trust_in_media Impulsivity_SC4 NegEmo_SC6 if chris_sample_reqs == 1
```

Variable	Obs	Mean	Std. Dev.	Min	Max
DV_Complia~7	1,012	6.00974	1.197677	1	7
Age	1,012	40.31917	12.87619	17	68
Gender_Fem~e					
0	1,012	.4347826	.4959736	0	1
1	1,012	.5652174	.4959736	0	1
Minority					
0	1,012	.6897233	.4628355	0	1
1	1,012	.3102767	.4628355	0	1
Education	1,012	3.924901	1.497209	1	8
Employed					
0	1,012	.3428854	.4749083	0	1
1	1,012	.6571146	.4749083	0	1
Corona_care					
0	1,012	.9318182	.2521823	0	1
1	1,012	.0681818	.2521823	0	1
Insurance_~c					
0	1,012	.7262846	.4460851	0	1
1	1,012	.2737154	.4460851	0	1
Insurance_~e					
0	1,012	.4031621	.4907753	0	1
1	1,012	.5968379	.4907753	0	1
SES_before	1,012	6.049407	1.950305	1	10
SES_change	1,012	-.4397233	1.663396	-9	7
Health_self					
1	1,012	.68083	.4663855	0	1
2	1,012	.31917	.4663855	0	1
Health_other					
1	1,012	.4209486	.4939554	0	1
2	1,012	.5790514	.4939554	0	1

```
Conservat~01 |
           0 |      1,012    .5365613     .498908          0          1
           1 |      1,012    .4634387     .498908          0          1
             |
Conservati~r |
           0 |      1,012    .8754941    .3303211          0          1
           1 |      1,012    .1245059    .3303211          0          1
-------------+---------------------------------------------------------
             |
GeoCensus_d1 |
           0 |      1,012    .7865613    .4099374          0          1
           1 |      1,012    .2134387    .4099374          0          1
             |
GeoCensus_d2 |
           0 |      1,012    .5573123      .49695          0          1
           1 |      1,012    .4426877      .49695          0          1
             |
GeoCensus_d3 |
           0 |      1,012    .8577075    .3495226          0          1
-------------+---------------------------------------------------------
           1 |      1,012    .1422925    .3495226          0          1
             |
Current_me~s |
           0 |      1,012    .0958498    .2945307          0          1
         Yes |      1,012    .9041502    .2945307          0          1
             |
Measures_c~r |      1,012     5.36166    1.623161          1          7
MA_Perc_Th~3 |      1,012    5.596509    1.470155          1          7
-------------+---------------------------------------------------------
   Costs_SC5 |      1,012    4.308696    1.616251          1          7
Deterr_SD_~2 |      1,012    3.340909    1.762026          1          7
Deterr_SD_~e |      1,012    3.800395    1.698804          1          6
MA_MoralBe~f |      1,012    6.211462    1.183779          1          7
MA_Authori~2 |      1,012     4.29002    1.849594          1          7
-------------+---------------------------------------------------------
     NOO_SC3 |      1,012    3.969368    .8523516          1          5
    NNOO_SC3 |      1,012    2.953228     .989675          1          5
   OOL_SC12 |      1,012    4.397892    1.459976          1          7
     PJE_SC4 |      1,012    5.240613    1.513755          1          7
Trust_Scie~4 |      1,012    3.890563    .9653709          1          5
-------------+---------------------------------------------------------
Trust_in_m~a |      1,012    2.917984    1.296039          1          5
Impulsivi~C4 |      1,012     2.39748    1.103923          1          5
  NegEmo_SC6 |      1,012    4.603096    1.528395          1          7

177 .
178 . *5.a.2 Regression
179 . reg DV_Compliance_SC7  Age i.Gender_Female i.Minority Education i.Employed i.Corona_care i.Insurance_Public i.Insura
    > vative_other i.GeoCensus_d1 i.GeoCensus_d2 i.GeoCensus_d3 i.Current_measures Measures_clear MA_Perc_Threat_SC3 Costs
    > C3 OOL_SC12 PJE_SC4 Trust_Science_SC4 Trust_in_media Impulsivity_SC4 NegEmo_SC6 if chris_sample_reqs == 1

      Source |       SS           df       MS      Number of obs   =     1,012
-------------+----------------------------------   F(33, 978)      =     20.14
       Model |  586.802154        33  17.7818834   Prob > F        =    0.0000
    Residual |  863.407958       978  .882830223   R-squared       =    0.4046
-------------+----------------------------------   Adj R-squared   =    0.3845
       Total |  1450.21011     1,011  1.43443137   Root MSE        =    .93959

------------------------------------------------------------------------------------
  DV_Compliance_SC7 |      Coef.   Std. Err.      t    P>|t|     [95% Conf. Interval]
--------------------+---------------------------------------------------------------
                Age |   .0038179   .0025984     1.47   0.142    -.0012813     .008917
    1.Gender_Female |   .1143239   .0627567     1.82   0.069    -.0088293    .2374771
         1.Minority |  -.0805974   .0687846    -1.17   0.242    -.2155797     .054385
          Education |   .0424963   .0229983     1.85   0.065    -.0026353    .0876279
         1.Employed |  -.1283552   .0701254    -1.83   0.067    -.2659688    .0092584
      1.Corona_care |  -.2322209     .12997    -1.79   0.074    -.4872731    .0228312
 1.Insurance_Public |   .0300588   .1033952     0.29   0.771    -.1728432    .2329608
1.Insurance_Private |   .0485601   .0981123     0.49   0.621    -.1439748    .2410949
         SES_before |    .029705   .0177142     1.68   0.094    -.0050573    .0644672
         SES_change |   .0398611   .0202847     1.97   0.050     .0000545    .0796677
      2.Health_self |  -.0034242    .071561    -0.05   0.962     -.143855    .1370065
     2.Health_other |   .0517207   .0674885     0.77   0.444    -.0807181    .1841596
  1.Conservative_01 |   .0792863   .0729411     1.09   0.277    -.0638529    .2224254
```

1.Conservative_other	.159508	.1017914	1.57	0.117	-.0402467	.3592628
1.GeoCensus_d1	-.0562819	.0949849	-0.59	0.554	-.2426796	.1301157
1.GeoCensus_d2	-.1490608	.0818325	-1.82	0.069	-.3096484	.0115267
1.GeoCensus_d3	-.0293599	.1046672	-0.28	0.779	-.234758	.1760382
Current_measures						
Yes	.316873	.1072843	2.95	0.003	.106339	.527407
Measures_clear	-.0088856	.0217666	-0.41	0.683	-.0516002	.0338289
MA_Perc_Threat_SC3	.1179087	.0320794	3.68	0.000	.0549564	.1808611
Costs_SC5	.0342598	.0222146	1.54	0.123	-.0093339	.0778535
Deterr_SD_Likely_SC2	.0420933	.0204294	2.06	0.040	.0020027	.0821838
Deterr_SD_Severe	-.0298948	.0192257	-1.55	0.120	-.0676231	.0078335
MA_MoralBelief	.4161319	.0375025	11.10	0.000	.3425373	.4897265
MA_Authority_SC2	-.0042351	.0198453	-0.21	0.831	-.0431794	.0347092
NOO_SC3	.0622466	.0473638	1.31	0.189	-.0306997	.1551929
NNOO_SC3	-.0417366	.0377325	-1.11	0.269	-.1157825	.0323093
OOL_SC12	.0401804	.02633	1.53	0.127	-.0114893	.0918501
PJE_SC4	.0093867	.0229878	0.41	0.683	-.0357244	.0544978
Trust_Science_SC4	-.0190474	.0395193	-0.48	0.630	-.0965998	.0585049
Trust_in_media	-.0131015	.0283856	-0.46	0.645	-.0688052	.0426022
Impulsivity_SC4	-.1457193	.0346136	-4.21	0.000	-.2136446	-.0777939
NegEmo_SC6	.0204552	.0234399	0.87	0.383	-.0255432	.0664535
_cons	1.924744	.3569053	5.39	0.000	1.224356	2.625132

```
180 . estimates store model_5

181 .
182 . *5.a.3 Check hettest: Run this right after your regression to apply the Breusch-Pagan / Cook-Weisberg test for heter
183 . *if significant, then you need to run the regression with vce(ro) at the end
184 . estat hettest

    Breusch-Pagan / Cook-Weisberg test for heteroskedasticity
             Ho: Constant variance
             Variables: fitted values of DV_Compliance_SC7

             chi2(1)      =   179.71
             Prob > chi2  =   0.0000

185 .
186 . *5.a.4. check vif, to check for for multicollinearity (VIFs >10 are problematic)
187 . vif
```

Variable	VIF	1/VIF
Age	1.28	0.780069
1.Gender_F~e	1.11	0.901341
1.Minority	1.16	0.861570
Education	1.36	0.736498
1.Employed	1.27	0.787328
1.Corona_c~e	1.23	0.812850
1.Insuranc~c	2.44	0.410478
1.Insuran~te	2.66	0.376629
SES_before	1.37	0.731605
SES_change	1.30	0.767001
2.Health_s~f	1.28	0.783941
2.Health_o~r	1.27	0.785763
1.Conserv~01	1.52	0.659386
1.Conserva~r	1.29	0.772380
1.GeoCensu~1	1.74	0.575946
1.GeoCensu~2	1.89	0.528018
1.GeoCensu~3	1.53	0.652461
1.Current_~s	1.14	0.874565
Measures_c~r	1.43	0.699557
MA_Perc_Th~3	2.55	0.392597
Costs_SC5	1.48	0.677381
Deterr_SD_~2	1.48	0.673891
Deterr_SD_~e	1.22	0.818611
MA_MoralBe~f	2.26	0.443062
MA_Authori~2	1.54	0.648122
NOO_SC3	1.87	0.535792
NNOO_SC3	1.60	0.626196
OOL_SC12	1.69	0.590928
PJE_SC4	1.39	0.721139

Trust_Scie~4	1.67	0.599956
Trust_in_m~a	1.55	0.645198
Impulsivi~C4	1.67	0.598076
NegEmo_SC6	1.47	0.680365
Mean VIF	1.57	

```
188 .
189 . *5.a.5. Effect size
190 . estat esize
```

Effect sizes for linear models

Source	Eta-Squared	df	[95% Conf.	Interval]
Model	.4046325	33	.3401634	.4267922
Age	.0022026	1	.	.0118624
Gender_Female	.0033818	1	.	.0143919
Minority	.0014019	1	.	.0099101
Education	.003479	1	.	.0145887
Employed	.0034139	1	.	.0144571
Corona_care	.0032536	1	.	.0141304
Insurance_Public	.0000864	1	.	.0046236
Insurance_Private	.0002504	1	.	.005969
SES_before	.002867	1	.	.013324
SES_change	.0039329	1	.	.0154882
Health_self	2.34e-06	1	.	.0008627
Health_other	.0006002	1	.	.0075053
Conservative_01	.0012067	1	.	.0093845
Conservative_other	.0025045	1	.	.0125401
GeoCensus_d1	.0003589	1	.	.0065288
GeoCensus_d2	.0033812	1	.	.0143907
GeoCensus_d3	.0000804	1	.	.0045427
Current_measures	.008841	1	.0009946	.0240778
Measures_clear	.0001704	1	.	.0054422
MA_Perc_Threat_SC3	.0136252	1	.0029675	.0314525
Costs_SC5	.0024261	1	.	.0123664
Deterr_SD_Likely_SC2	.0043221	1	.	.0162385
Deterr_SD_Severe	.0024661	1	.	.0124554
MA_MoralBelief	.1118164	1	.0774861	.1492911
MA_Authority_SC2	.0000466	1	.	.0039432
NOO_SC3	.0017629	1	.	.0108239
NNOO_SC3	.0012495	1	.	.009502
OOL_SC12	.0023755	1	.	.0122537
PJE_SC4	.0001705	1	.	.0054428
Trust_Science_SC4	.0002375	1	.	.0058926
Trust_in_media	.0002178	1	.	.0057708
Impulsivity_SC4	.0177993	1	.0050939	.0374825
NegEmo_SC6	.0007781	1	.	.0081116

Note: Eta-Squared values for individual model terms are partial.

```
191 .
192 . *5.a.6 Regression with vce(ro)
193 . reg DV_Compliance_SC7  Age i.Gender_Female i.Minority Education i.Employed i.Corona_care i.Insurance_Public i.Insura
    > vative_other i.GeoCensus_d1 i.GeoCensus_d2 i.GeoCensus_d3 i.Current_measures Measures_clear MA_Perc_Threat_SC3 Costs
    > C3 OOL_SC12 PJE_SC4 Trust_Science_SC4 Trust_in_media Impulsivity_SC4 NegEmo_SC6 if chris_sample_reqs == 1, vce(ro)
```

```
Linear regression                               Number of obs     =      1,012
                                                F(33, 978)        =      16.13
                                                Prob > F          =     0.0000
                                                R-squared         =     0.4046
                                                Root MSE          =     .93959
```

DV_Compliance_SC7	Coef.	Robust Std. Err.	t	P>|t|	[95% Conf.	Interval]
Age	.0038179	.0026568	1.44	0.151	-.0013958	.0090315
1.Gender_Female	.1143239	.0664449	1.72	0.086	-.0160671	.2447149
1.Minority	-.0805974	.0674723	-1.19	0.233	-.2130045	.0518097
Education	.0424963	.0220906	1.92	0.055	-.0008541	.0858467
1.Employed	-.1283552	.0704518	-1.82	0.069	-.2666093	.0098988
1.Corona_care	-.2322209	.1393025	-1.67	0.096	-.5055871	.0411452
1.Insurance_Public	.0300588	.113691	0.26	0.792	-.1930477	.2531652
1.Insurance_Private	.0485601	.1100586	0.44	0.659	-.167418	.2645381
SES_before	.029705	.0193338	1.54	0.125	-.0082355	.0676454
SES_change	.0398611	.0216385	1.84	0.066	-.0026022	.0823244
2.Health_self	-.0034242	.0651988	-0.05	0.958	-.1313698	.1245214
2.Health_other	.0517207	.0654776	0.79	0.430	-.076772	.1802135
1.Conservative_01	.0792863	.0780458	1.02	0.310	-.0738703	.2324428
1.Conservative_other	.159508	.1025622	1.56	0.120	-.0417593	.3607753
1.GeoCensus_d1	-.0562819	.0872809	-0.64	0.519	-.2275613	.1149975
1.GeoCensus_d2	-.1490608	.072449	-2.06	0.040	-.2912343	-.0068874
1.GeoCensus_d3	-.0293599	.0901676	-0.33	0.745	-.2063041	.1475843
Current_measures						
Yes	.316873	.1310485	2.42	0.016	.0597044	.5740416
Measures_clear	-.0088856	.0209406	-0.42	0.671	-.0499792	.0322079
MA_Perc_Threat_SC3	.1179087	.0377249	3.13	0.002	.0438776	.1919398
Costs_SC5	.0342598	.0226903	1.51	0.131	-.0102676	.0787872
Deterr_SD_Likely_SC2	.0420933	.0196873	2.14	0.033	.0034591	.0807275
Deterr_SD_Severe	-.0298948	.0197657	-1.51	0.131	-.0686828	.0088932
MA_MoralBelief	.4161319	.0472588	8.81	0.000	.3233916	.5088722
MA_Authority_SC2	-.0042351	.018829	-0.22	0.822	-.0411849	.0327147
NOO_SC3	.0622466	.0474449	1.31	0.190	-.030859	.1553522
NNOO_SC3	-.0417366	.0387915	-1.08	0.282	-.1178608	.0343877
OOL_SC12	.0401804	.0281246	1.43	0.153	-.0150112	.095372
PJE_SC4	.0093867	.0235132	0.40	0.690	-.0367554	.0555288
Trust_Science_SC4	-.0190474	.0377017	-0.51	0.614	-.0930329	.0549381
Trust_in_media	-.0131015	.0268424	-0.49	0.626	-.0657769	.0395739
Impulsivity_SC4	-.1457193	.0332025	-4.39	0.000	-.2108756	-.0805629
NegEmo_SC6	.0204552	.02225	0.92	0.358	-.023208	.0641183
_cons	1.924744	.3664993	5.25	0.000	1.205529	2.64396

```
194 .
195 .
196 . *****************
197 .
198 . *6. Step 6: Add social environment
199 .
200 . *6.a.1 Descriptive Statistics
201 . sum DV_Compliance_SC7  Age i.Gender_Female i.Minority Education i.Employed i.Corona_care i.Insurance_Public i.Insura
    > vative_other i.GeoCensus_d1 i.GeoCensus_d2 i.GeoCensus_d3 i.Current_measures Measures_clear MA_Perc_Threat_SC3 Costs
    > C3 OOL_SC12 PJE_SC4 Trust_Science_SC4 Trust_in_media Impulsivity_SC4 NegEmo_SC6 SN_SC7 if chris_sample_reqs == 1
```

Variable	Obs	Mean	Std. Dev.	Min	Max
DV_Complia~7	1,012	6.00974	1.197677	1	7
Age	1,012	40.31917	12.87619	17	68
Gender_Fem~e					
0	1,012	.4347826	.4959736	0	1
1	1,012	.5652174	.4959736	0	1
Minority					
0	1,012	.6897233	.4628355	0	1
1	1,012	.3102767	.4628355	0	1
Education	1,012	3.924901	1.497209	1	8
Employed					
0	1,012	.3428854	.4749083	0	1
1	1,012	.6571146	.4749083	0	1
Corona_care					

0	1,012	.9318182	.2521823	0	1
1	1,012	.0681818	.2521823	0	1
Insurance_~c					
0	1,012	.7262846	.4460851	0	1
1	1,012	.2737154	.4460851	0	1
Insurance_~e					
0	1,012	.4031621	.4907753	0	1
1	1,012	.5968379	.4907753	0	1
SES_before	1,012	6.049407	1.950305	1	10
SES_change	1,012	-.4397233	1.663396	-9	7
Health_self					
1	1,012	.68083	.4663855	0	1
2	1,012	.31917	.4663855	0	1
Health_other					
1	1,012	.4209486	.4939554	0	1
2	1,012	.5790514	.4939554	0	1
Conservat~01					
0	1,012	.5365613	.498908	0	1
1	1,012	.4634387	.498908	0	1
Conservati~r					
0	1,012	.8754941	.3303211	0	1
1	1,012	.1245059	.3303211	0	1
GeoCensus_d1					
0	1,012	.7865613	.4099374	0	1
1	1,012	.2134387	.4099374	0	1
GeoCensus_d2					
0	1,012	.5573123	.49695	0	1
1	1,012	.4426877	.49695	0	1
GeoCensus_d3					
0	1,012	.8577075	.3495226	0	1
1	1,012	.1422925	.3495226	0	1
Current_me~s					
0	1,012	.0958498	.2945307	0	1
Yes	1,012	.9041502	.2945307	0	1
Measures_c~r	1,012	5.36166	1.623161	1	7
MA_Perc_Th~3	1,012	5.596509	1.470155	1	7
Costs_SC5	1,012	4.308696	1.616251	1	7
Deterr_SD_~2	1,012	3.340909	1.762026	1	7
Deterr_SD_~e	1,012	3.800395	1.698804	1	6
MA_MoralBe~f	1,012	6.211462	1.183779	1	7
MA_Authori~2	1,012	4.29002	1.849594	1	7
NOO_SC3	1,012	3.969368	.8523516	1	5
NNOO_SC3	1,012	2.953228	.989675	1	5
OOL_SC12	1,012	4.397892	1.459976	1	7
PJE_SC4	1,012	5.240613	1.513755	1	7
Trust_Scie~4	1,012	3.890563	.9653709	1	5
Trust_in_m~a	1,012	2.917984	1.296039	1	5
Impulsivi~C4	1,012	2.39748	1.103923	1	5
NegEmo_SC6	1,012	4.603096	1.528395	1	7
SN_SC7	1,012	5.457369	1.296999	1	7

```
202 .
203 . *6.a.2 Regression
204 . reg DV_Compliance_SC7  Age i.Gender_Female i.Minority Education i.Employed i.Corona_care i.Insurance_Public i.Insura
    > vative_other i.GeoCensus_d1 i.GeoCensus_d2 i.GeoCensus_d3 i.Current_measures Measures_clear MA_Perc_Threat_SC3 Costs
    > C3 OOL_SC12 PJE_SC4 Trust_Science_SC4 Trust_in_media Impulsivity_SC4 NegEmo_SC6 SN_SC7 if chris_sample_reqs == 1
```

Source	SS	df	MS
Model	617.505641	34	18.1619306
Residual	832.70447	977	.852307544
Total	1450.21011	1,011	1.43443137

Number of obs	=	1,012
F(34, 977)	=	21.31
Prob > F	=	0.0000
R-squared	=	0.4258
Adj R-squared	=	0.4058
Root MSE	=	.92321

DV_Compliance_SC7	Coef.	Std. Err.	t	P>|t|	[95% Conf.	Interval]
Age	.0036374	.0025533	1.42	0.155	-.0013731	.008648
1.Gender_Female	.1181395	.0616655	1.92	0.056	-.0028726	.2391517
1.Minority	-.0753828	.0675906	-1.12	0.265	-.2080224	.0572567
Education	.0347571	.022634	1.54	0.125	-.0096597	.0791738
1.Employed	-.1315832	.0689046	-1.91	0.056	-.2668013	.0036348
1.Corona_care	-.191268	.1278856	-1.50	0.135	-.4422301	.0596942
1.Insurance_Public	.0525202	.101661	0.52	0.606	-.1469789	.2520192
1.Insurance_Private	.0518846	.0964029	0.54	0.591	-.137296	.2410652
SES_before	.027135	.0174106	1.56	0.119	-.0070314	.0613014
SES_change	.0363071	.0199398	1.82	0.069	-.0028226	.0754368
2.Health_self	-.0093177	.0703199	-0.13	0.895	-.1473131	.1286777
2.Health_other	.0616114	.066332	0.93	0.353	-.0685582	.191781
1.Conservative_01	.0692483	.0716886	0.97	0.334	-.0714332	.2099297
1.Conservative_other	.1725091	.1000397	1.72	0.085	-.0238085	.3688266
1.GeoCensus_d1	-.0315486	.0934194	-0.34	0.736	-.2148744	.1517772
1.GeoCensus_d2	-.1056657	.0807299	-1.31	0.191	-.2640897	.0527582
1.GeoCensus_d3	-.0007503	.1029523	-0.01	0.994	-.2027833	.2012828
Current_measures						
Yes	.2773393	.105619	2.63	0.009	.070073	.4846055
Measures_clear	-.0142531	.0214057	-0.67	0.506	-.0562595	.0277533
MA_Perc_Threat_SC3	.1255644	.0315458	3.98	0.000	.0636591	.1874696
Costs_SC5	.0357303	.0218285	1.64	0.102	-.007106	.0785665
Deterr_SD_Likely_SC2	.0337321	.0201214	1.68	0.094	-.0057541	.0732183
Deterr_SD_Severe	-.0218474	.0189379	-1.15	0.249	-.0590111	.0153162
MA_MoralBelief	.3874032	.0371581	10.43	0.000	.3144843	.460322
MA_Authority_SC2	-.0163153	.0196028	-0.83	0.405	-.0547838	.0221532
NOO_SC3	.0375159	.0467198	0.80	0.422	-.0541669	.1291986
NNOO_SC3	-.06401	.0372597	-1.72	0.086	-.1371283	.0091083
OOL_SC12	.036619	.0258776	1.42	0.157	-.0141631	.087401
PJE_SC4	.0010739	.0226294	0.05	0.962	-.0433338	.0454817
Trust_Science_SC4	-.0136869	.0388404	-0.35	0.725	-.089907	.0625333
Trust_in_media	-.023523	.0279446	-0.84	0.400	-.0783613	.0313154
Impulsivity_SC4	-.1500895	.0340177	-4.41	0.000	-.2168457	-.0833333
NegEmo_SC6	.0148468	.0230501	0.64	0.520	-.0303866	.0600802
SN_SC7	.1513872	.0252228	6.00	0.000	.1018901	.2008843
_cons	1.620236	.3543323	4.57	0.000	.9248964	2.315576

```
205 . estimates store model_6

206 .
207 . *6.a.3 Check hettest: Run this right after your regression to apply the Breusch-Pagan / Cook-Weisberg test for heter
208 . *if significant, then you need to run the regression with vce(ro) at the end
209 . estat hettest

    Breusch-Pagan / Cook-Weisberg test for heteroskedasticity
             Ho: Constant variance
             Variables: fitted values of DV_Compliance_SC7

             chi2(1)      =   161.96
             Prob > chi2  =   0.0000
```

210 .
211 . *6.a.4. check vif, to check for for multicollinearity (VIFs >10 are problematic)
212 . vif

Variable	VIF	1/VIF
Age	1.28	0.779961
1.Gender_F~e	1.11	0.901246
1.Minority	1.16	0.861427
Education	1.36	0.734107
1.Employed	1.27	0.787280
1.Corona_c~e	1.23	0.810537
1.Insuranc~c	2.44	0.409922
1.Insuran~te	2.66	0.376616
SES_before	1.37	0.731163
SES_change	1.30	0.766325
2.Health_s~f	1.28	0.783788
2.Health_o~r	1.27	0.785278
1.Conserv~01	1.52	0.659027
1.Conserva~r	1.30	0.772018
1.GeoCensu~1	1.74	0.574825
1.GeoCensu~2	1.91	0.523783
1.GeoCensu~3	1.54	0.651062
1.Current_~s	1.15	0.871164
Measures_c~r	1.43	0.698336
MA_Perc_Th~3	2.55	0.391956
Costs_SC5	1.48	0.677295
Deterr_SD_~2	1.49	0.670661
Deterr_SD_~e	1.23	0.814508
MA_MoralBe~f	2.30	0.435710
MA_Authori~2	1.56	0.641290
NOO_SC3	1.88	0.531624
NNOO_SC3	1.61	0.619984
OOL_SC12	1.69	0.590617
PJE_SC4	1.39	0.718438
Trust_Scie~4	1.67	0.599639
Trust_in_m~a	1.56	0.642707
Impulsivi~C4	1.67	0.597802
NegEmo_SC6	1.47	0.679247
SN_SC7	1.27	0.787732
Mean VIF	1.56	

213 .
214 . *6.a.5. Effect size
215 . estat esize

Effect sizes for linear models

Source	Eta-Squared	df	[95% Conf.	Interval]
Model	.4258043	34	.3619418	.4472992
Age	.002073	1	.	.01157
Gender_Female	.0037427	1	.	.0151223
Minority	.0012715	1	.	.0095682
Education	.0024078	1	.	.0123328
Employed	.0037187	1	.	.0150749
Corona_care	.0022843	1	.	.0120553
Insurance_Public	.0002731	1	.	.0061016
Insurance_Private	.0002964	1	.	.0062262
SES_before	.0024801	1	.	.0124932
SES_change	.003382	1	.	.0143999
Health_self	.000018	1	.	.0029478
Health_other	.0008823	1	.	.0084465
Conservative_01	.0009541	1	.	.0086651
Conservative_other	.0030343	1	.	.0136838
GeoCensus_d1	.0001167	1	.	.0049772
GeoCensus_d2	.0017504	1	.	.0107999
GeoCensus_d3	5.44e-08	1	.	.
Current_measures	.0070079	1	.0004347	.021054
Measures_clear	.0004536	1	.	.0069478
MA_Perc_Threat_SC3	.0159577	1	.0041158	.0348689

Costs_SC5	.0027349	1	.	.0130489
Deterr_SD_Likely_SC2	.0028683	1	.	.0133341
Deterr_SD_Severe	.0013604	1	.	.0098066
MA_MoralBelief	.1001178	1	.0673709	.13644
MA_Authority_SC2	.0007085	1	.	.0078877
NOO_SC3	.0006595	1	.	.0077206
NNOO_SC3	.0030117	1	.	.0136364
OOL_SC12	.0020454	1	.	.0115057
PJE_SC4	2.31e-06	1	.	.0008467
Trust_Science_SC4	.0001271	1	.	.0050796
Trust_in_media	.0007247	1	.	.007942
Impulsivity_SC4	.0195356	1	.0060508	.039926
NegEmo_SC6	.0004245	1	.	.0068258
SN_SC7	.0355608	1	.0162592	.0610512

Note: Eta-Squared values for individual model terms are partial.

```
216 .
217 . *6.a.6 Regression with vce(ro)
218 . reg DV_Compliance_SC7  Age i.Gender_Female i.Minority Education i.Employed i.Corona_care i.Insurance_Public i.Insura
    > vative_other i.GeoCensus_d1 i.GeoCensus_d2 i.GeoCensus_d3 i.Current_measures Measures_clear MA_Perc_Threat_SC3 Costs
    > C3 OOL_SC12 PJE_SC4 Trust_Science_SC4 Trust_in_media Impulsivity_SC4 NegEmo_SC6 SN_SC7 if chris_sample_reqs == 1, vc
```

Linear regression

Number of obs	=	1,012
F(34, 977)	=	18.49
Prob > F	=	0.0000
R-squared	=	0.4258
Root MSE	=	.92321

DV_Compliance_SC7	Coef.	Robust Std. Err.	t	P>|t|	[95% Conf.	Interval]
Age	.0036374	.0026196	1.39	0.165	-.0015033	.0087781
1.Gender_Female	.1181395	.065456	1.80	0.071	-.0103111	.2465901
1.Minority	-.0753828	.0664895	-1.13	0.257	-.2058614	.0550958
Education	.0347571	.0215121	1.62	0.106	-.0074582	.0769723
1.Employed	-.1315832	.0689706	-1.91	0.057	-.2669309	.0037644
1.Corona_care	-.191268	.1390607	-1.38	0.169	-.46416	.0816241
1.Insurance_Public	.0525202	.1108524	0.47	0.636	-.165016	.2700564
1.Insurance_Private	.0518846	.1077734	0.48	0.630	-.1596094	.2633785
SES_before	.027135	.0191963	1.41	0.158	-.0105358	.0648058
SES_change	.0363071	.0212092	1.71	0.087	-.0053138	.077928
2.Health_self	-.0093177	.0644686	-0.14	0.885	-.1358305	.1171951
2.Health_other	.0616114	.0639855	0.96	0.336	-.0639534	.1871762
1.Conservative_01	.0692483	.0766993	0.90	0.367	-.0812661	.2197626
1.Conservative_other	.1725091	.1025871	1.68	0.093	-.0288073	.3738255
1.GeoCensus_d1	-.0315486	.0862326	-0.37	0.715	-.2007709	.1376737
1.GeoCensus_d2	-.1056657	.0714879	-1.48	0.140	-.2459533	.0346218
1.GeoCensus_d3	-.0007503	.0876813	-0.01	0.993	-.1728156	.1713151
Current_measures						
Yes	.2773393	.1287523	2.15	0.031	.0246764	.5300021
Measures_clear	-.0142531	.0206845	-0.69	0.491	-.0548442	.026338
MA_Perc_Threat_SC3	.1255644	.0369373	3.40	0.001	.0530788	.1980499
Costs_SC5	.0357303	.0222836	1.60	0.109	-.0079989	.0794594
Deterr_SD_Likely_SC2	.0337321	.0194032	1.74	0.082	-.0043446	.0718089
Deterr_SD_Severe	-.0218474	.0196297	-1.11	0.266	-.0603687	.0166738
MA_MoralBelief	.3874032	.0466337	8.31	0.000	.2958894	.4789169
MA_Authority_SC2	-.0163153	.0182192	-0.90	0.371	-.0520686	.019438
NOO_SC3	.0375159	.0465399	0.81	0.420	-.0538138	.1288455
NNOO_SC3	-.06401	.038745	-1.65	0.099	-.1400431	.0120231
OOL_SC12	.036619	.027864	1.31	0.189	-.0180612	.0912992
PJE_SC4	.0010739	.0229037	0.05	0.963	-.0438721	.0460199
Trust_Science_SC4	-.0136869	.0373033	-0.37	0.714	-.0868907	.059517
Trust_in_media	-.023523	.0269316	-0.87	0.383	-.0763735	.0293276
Impulsivity_SC4	-.1500895	.0322337	-4.66	0.000	-.2133448	-.0868343
NegEmo_SC6	.0148468	.0219901	0.68	0.500	-.0283064	.0580001
SN_SC7	.1513872	.0258496	5.86	0.000	.10066	.2021144
_cons	1.620236	.3621014	4.47	0.000	.9096503	2.330822

```
. 
. 
. *****************
. 
. *7. Step 7: Add practical circumstances
. 
. *7.a.1 Descriptive Statistics
. sum DV_Compliance_SC7  Age i.Gender_Female i.Minority Education i.Employed i.Corona_care i.Insurance_Public i.Insura
> vative_other i.GeoCensus_d1 i.GeoCensus_d2 i.GeoCensus_d3 i.Current_measures Measures_clear MA_Perc_Threat_SC3 Costs
> C3 OOL_SC12 PJE_SC4 Trust_Science_SC4 Trust_in_media Impulsivity_SC4 NegEmo_SC6 SN_SC7 CTC_SC7 OTC_SC7 if chris_samp
```

Variable	Obs	Mean	Std. Dev.	Min	Max
DV_Complia~7	1,012	6.00974	1.197677	1	7
Age	1,012	40.31917	12.87619	17	68
Gender_Fem~e					
0	1,012	.4347826	.4959736	0	1
1	1,012	.5652174	.4959736	0	1
Minority					
0	1,012	.6897233	.4628355	0	1
1	1,012	.3102767	.4628355	0	1
Education	1,012	3.924901	1.497209	1	8
Employed					
0	1,012	.3428854	.4749083	0	1
1	1,012	.6571146	.4749083	0	1
Corona_care					
0	1,012	.9318182	.2521823	0	1
1	1,012	.0681818	.2521823	0	1
Insurance_~c					
0	1,012	.7262846	.4460851	0	1
1	1,012	.2737154	.4460851	0	1
Insurance_~e					
0	1,012	.4031621	.4907753	0	1
1	1,012	.5968379	.4907753	0	1
SES_before	1,012	6.049407	1.950305	1	10
SES_change	1,012	-.4397233	1.663396	-9	7
Health_self					
1	1,012	.68083	.4663855	0	1
2	1,012	.31917	.4663855	0	1
Health_other					
1	1,012	.4209486	.4939554	0	1
2	1,012	.5790514	.4939554	0	1
Conservat~01					
0	1,012	.5365613	.498908	0	1
1	1,012	.4634387	.498908	0	1
Conservati~r					
0	1,012	.8754941	.3303211	0	1
1	1,012	.1245059	.3303211	0	1
GeoCensus_d1					
0	1,012	.7865613	.4099374	0	1
1	1,012	.2134387	.4099374	0	1
GeoCensus_d2					
0	1,012	.5573123	.49695	0	1
1	1,012	.4426877	.49695	0	1

GeoCensus_d3					
0	1,012	.8577075	.3495226	0	1
1	1,012	.1422925	.3495226	0	1
Current_me~s					
0	1,012	.0958498	.2945307	0	1
Yes	1,012	.9041502	.2945307	0	1
Measures_c~r	1,012	5.36166	1.623161	1	7
MA_Perc_Th~3	1,012	5.596509	1.470155	1	7
Costs_SC5	1,012	4.308696	1.616251	1	7
Deterr_SD_~2	1,012	3.340909	1.762026	1	7
Deterr_SD_~e	1,012	3.800395	1.698804	1	6
MA_MoralBe~f	1,012	6.211462	1.183779	1	7
MA_Authori~2	1,012	4.29002	1.849594	1	7
NOO_SC3	1,012	3.969368	.8523516	1	5
NNOO_SC3	1,012	2.953228	.989675	1	5
OOL_SC12	1,012	4.397892	1.459976	1	7
PJE_SC4	1,012	5.240613	1.513755	1	7
Trust_Scie~4	1,012	3.890563	.9653709	1	5
Trust_in_m~a	1,012	2.917984	1.296039	1	5
Impulsivi~C4	1,012	2.39748	1.103923	1	5
NegEmo_SC6	1,012	4.603096	1.528395	1	7
SN_SC7	1,012	5.457369	1.296999	1	7
CTC_SC7	1,012	6.062253	.9418468	1	7
OTC_SC7	1,012	4.460898	1.781575	1	7

```
227 .
228 . *7.a.2 Regression
229 . reg DV_Compliance_SC7  Age i.Gender_Female i.Minority Education i.Employed i.Corona_care i.Insurance_Public i.Insura
    > vative_other i.GeoCensus_d1 i.GeoCensus_d2 i.GeoCensus_d3 i.Current_measures Measures_clear MA_Perc_Threat_SC3 Costs
    > C3 OOL_SC12 PJE_SC4 Trust_Science_SC4 Trust_in_media Impulsivity_SC4 NegEmo_SC6 SN_SC7 CTC_SC7 OTC_SC7 if chris_samp
```

Source	SS	df	MS			
				Number of obs	=	1,012
				F(36, 975)	=	29.94
Model	761.447096	36	21.1513082	Prob > F	=	0.0000
Residual	688.763015	975	.706423606	R-squared	=	0.5251
				Adj R-squared	=	0.5075
Total	1450.21011	1,011	1.43443137	Root MSE	=	.84049

DV_Compliance_SC7	Coef.	Std. Err.	t	P>|t|	[95% Conf.	Interval]
Age	.00213	.0023278	0.92	0.360	-.002438	.0066981
1.Gender_Female	.118856	.0561576	2.12	0.035	.0086524	.2290597
1.Minority	-.0722763	.0615361	-1.17	0.240	-.1930348	.0484821
Education	.0213801	.0206276	1.04	0.300	-.0190995	.0618596
1.Employed	-.0743246	.0629215	-1.18	0.238	-.1978018	.0491526
1.Corona_care	-.0541879	.1169328	-0.46	0.643	-.2836568	.175281
1.Insurance_Public	.0986836	.0926234	1.07	0.287	-.0830805	.2804477
1.Insurance_Private	.0575465	.0877677	0.66	0.512	-.1146888	.2297818
SES_before	.0232587	.0158652	1.47	0.143	-.0078751	.0543925
SES_change	.018444	.0182043	1.01	0.311	-.0172802	.0541682
2.Health_self	.0082054	.064033	0.13	0.898	-.117453	.1338638
2.Health_other	.0808748	.060417	1.34	0.181	-.0376875	.199437
1.Conservative_01	.0745618	.0653489	1.14	0.254	-.0536788	.2028024
1.Conservative_other	.1270175	.0912087	1.39	0.164	-.0519704	.3060054
1.GeoCensus_d1	-.0444295	.0850702	-0.52	0.602	-.2113714	.1225123
1.GeoCensus_d2	-.1084421	.0735071	-1.48	0.140	-.2526925	.0358083
1.GeoCensus_d3	.0077576	.0937973	0.08	0.934	-.1763102	.1918254
Current_measures						
Yes	.203247	.0963003	2.11	0.035	.0142672	.3922268
Measures_clear	-.0248332	.0195025	-1.27	0.203	-.0631049	.0134384
MA_Perc_Threat_SC3	.1238045	.0287261	4.31	0.000	.0674325	.1801765
Costs_SC5	.034696	.019925	1.74	0.082	-.0044048	.0737968
Deterr_SD_Likely_SC2	.0252867	.0183392	1.38	0.168	-.0107021	.0612755
Deterr_SD_Severe	-.0161186	.0172661	-0.93	0.351	-.0500016	.0177644
MA_MoralBelief	.2693976	.0348292	7.73	0.000	.2010488	.3377464

MA_Authority_SC2	-.0109672	.0178664	-0.61	0.539	-.0460283	.024094
NOO_SC3	-.021856	.042778	-0.51	0.610	-.1058036	.0620916
NNOO_SC3	-.057248	.0341825	-1.67	0.094	-.1243276	.0098317
OOL_SC12	.020743	.0236526	0.88	0.381	-.0256729	.0671588
PJE_SC4	-.0261986	.0207214	-1.26	0.206	-.0668623	.0144652
Trust_Science_SC4	-.0365378	.035409	-1.03	0.302	-.1060245	.0329489
Trust_in_media	-.016535	.0254456	-0.65	0.516	-.0664695	.0333995
Impulsivity_SC4	-.1069513	.0312231	-3.43	0.001	-.1682236	-.045679
NegEmo_SC6	.0023783	.0210034	0.11	0.910	-.0388389	.0435954
SN_SC7	.0259997	.0246417	1.06	0.292	-.0223572	.0743567
CTC_SC7	.5269948	.0370229	14.23	0.000	.4543411	.5996484
OTC_SC7	-.0112358	.0161133	-0.70	0.486	-.0428565	.020385
_cons	.5190459	.3352705	1.55	0.122	-.1388889	1.176981

```
230 . estimates store model_7

231 .
232 . *7.a.3 Check hettest: Run this right after your regression to apply the Breusch-Pagan / Cook-Weisberg test for heter
233 . *if significant, then you need to run the regression with vce(ro) at the end
234 . estat hettest

    Breusch-Pagan / Cook-Weisberg test for heteroskedasticity
             Ho: Constant variance
             Variables: fitted values of DV_Compliance_SC7

             chi2(1)      =   184.69
             Prob > chi2  =   0.0000

235 .
236 . *7.a.4. check vif, to check for for multicollinearity (VIFs >10 are problematic)
237 . vif
```

Variable	VIF	1/VIF
Age	1.29	0.777782
1.Gender_F~e	1.11	0.900699
1.Minority	1.16	0.861391
Education	1.37	0.732575
1.Employed	1.28	0.782521
1.Corona_c~e	1.24	0.803549
1.Insuranc~c	2.44	0.409296
1.Insuran~te	2.66	0.376599
SES_before	1.37	0.729827
SES_change	1.31	0.762031
2.Health_s~f	1.28	0.783459
2.Health_o~r	1.27	0.784550
1.Conserv~01	1.52	0.657350
1.Conserva~r	1.30	0.769784
1.GeoCensu~1	1.74	0.574544
1.GeoCensu~2	1.91	0.523636
1.GeoCensu~3	1.54	0.650104
1.Current_~s	1.15	0.868555
Measures_c~r	1.43	0.697286
MA_Perc_Th~3	2.55	0.391774
Costs_SC5	1.48	0.673751
Deterr_SD_~2	1.49	0.669160
Deterr_SD_~e	1.23	0.812156
MA_MoralBe~f	2.43	0.411042
MA_Authori~2	1.56	0.639860
NOO_SC3	1.90	0.525575
NNOO_SC3	1.64	0.610552
OOL_SC12	1.71	0.585955
PJE_SC4	1.41	0.710170
Trust_Scie~4	1.67	0.597994
Trust_in_m~a	1.56	0.642469
Impulsivi~C4	1.70	0.588144
NegEmo_SC6	1.47	0.678051
SN_SC7	1.46	0.684056
CTC_SC7	1.74	0.574663
OTC_SC7	1.18	0.847885
Mean VIF	1.57	

```
238 .
239 . *7.a.5. Effect size
240 . estat esize
```

Effect sizes for linear models

Source	Eta-Squared	df	[95% Conf.	Interval]
Model	.5250598	36	.4676558	.5441459
Age	.0008581	1	.	.0083829
Gender_Female	.0045733	1	.	.0167379
Minority	.0014129	1	.	.0099578
Education	.0011006	1	.	.0091051
Employed	.001429	1	.	.0100001
Corona_care	.0002202	1	.	.0057996
Insurance_Public	.0011629	1	.	.0092811
Insurance_Private	.0004407	1	.	.0069046
SES_before	.0021995	1	.	.0118758
SES_change	.0010517	1	.	.0089645
Health_self	.0000168	1	.	.0028846
Health_other	.0018345	1	.	.0110176
Conservative_01	.0013334	1	.	.0097474
Conservative_other	.0019851	1	.	.0113775
GeoCensus_d1	.0002797	1	.	.0061468
GeoCensus_d2	.0022272	1	.	.0119393
GeoCensus_d3	7.02e-06	1	.	.0019847
Current_measures	.0045479	1	.	.01669
Measures_clear	.0016602	1	.	.0105899
MA_Perc_Threat_SC3	.0186948	1	.0055707	.038777
Costs_SC5	.0031003	1	.	.0138361
Deterr_SD_Likely_SC2	.0019461	1	.	.0112852
Deterr_SD_Severe	.000893	1	.	.0084913
MA_MoralBelief	.057814	1	.0326572	.0882203
MA_Authority_SC2	.0003863	1	.	.0066692
NOO_SC3	.0002677	1	.	.0060808
NNOO_SC3	.0028685	1	.	.013349
OOL_SC12	.0007882	1	.	.0081613
PJE_SC4	.0016368	1	.	.0105314
Trust_Science_SC4	.0010909	1	.	.0090773
Trust_in_media	.0004329	1	.	.0068717
Impulsivity_SC4	.011891	1	.002173	.0288811
NegEmo_SC6	.0000132	1	.	.0026295
SN_SC7	.0011405	1	.	.0092182
CTC_SC7	.1720556	1	.1316721	.2136008
OTC_SC7	.0004984	1	.	.0071386

Note: Eta-Squared values for individual model terms are partial.

```
241 .
242 . *7.a.6 Regression with vce(ro)
243 . reg DV_Compliance_SC7  Age i.Gender_Female i.Minority Education i.Employed i.Corona_care i.Insurance_Public i.Insura
    > vative_other i.GeoCensus_d1 i.GeoCensus_d2 i.GeoCensus_d3 i.Current_measures Measures_clear MA_Perc_Threat_SC3 Costs
    > C3 OOL_SC12 PJE_SC4 Trust_Science_SC4 Trust_in_media Impulsivity_SC4 NegEmo_SC6 SN_SC7 CTC_SC7 OTC_SC7 if chris_samp
```

Linear regression

Number of obs	=	1,012
F(36, 975)	=	24.74
Prob > F	=	0.0000
R-squared	=	0.5251
Root MSE	=	.84049

DV_Compliance_SC7	Coef.	Robust Std. Err.	t	P>|t|	[95% Conf.	Interval]
Age	.00213	.0023377	0.91	0.362	-.0024576	.0067176
1.Gender_Female	.118856	.0593906	2.00	0.046	.002308	.2354041
1.Minority	-.0722763	.060426	-1.20	0.232	-.1908563	.0463036
Education	.0213801	.0197074	1.08	0.278	-.0172938	.0600539
1.Employed	-.0743246	.0618056	-1.20	0.229	-.1956118	.0469626
1.Corona_care	-.0541879	.1426205	-0.38	0.704	-.3340664	.2256906
1.Insurance_Public	.0986836	.0952508	1.04	0.300	-.0882365	.2856037
1.Insurance_Private	.0575465	.094001	0.61	0.541	-.126921	.242014

SES_before	.0232587	.0178479	1.30	0.193	-.0117661	.0582834
SES_change	.018444	.0189969	0.97	0.332	-.0188355	.0557236
2.Health_self	.0082054	.0569617	0.14	0.885	-.1035763	.1199871
2.Health_other	.0808748	.0572126	1.41	0.158	-.0313992	.1931487
1.Conservative_01	.0745618	.0684028	1.09	0.276	-.0596718	.2087954
1.Conservative_other	.1270175	.0944319	1.35	0.179	-.0582957	.3123307
1.GeoCensus_d1	-.0444295	.0770129	-0.58	0.564	-.1955597	.1067006
1.GeoCensus_d2	-.1084421	.0653223	-1.66	0.097	-.2366307	.0197465
1.GeoCensus_d3	.0077576	.0792879	0.10	0.922	-.147837	.1633521
Current_measures						
Yes	.203247	.1221055	1.66	0.096	-.0363729	.4428668
Measures_clear	-.0248332	.0182706	-1.36	0.174	-.0606875	.011021
MA_Perc_Threat_SC3	.1238045	.0325847	3.80	0.000	.0598604	.1877487
Costs_SC5	.034696	.0201697	1.72	0.086	-.0048851	.0742771
Deterr_SD_Likely_SC2	.0252867	.0177484	1.42	0.155	-.0095427	.0601162
Deterr_SD_Severe	-.0161186	.0185841	-0.87	0.386	-.0525881	.0203509
MA_MoralBelief	.2693976	.0424186	6.35	0.000	.1861554	.3526398
MA_Authority_SC2	-.0109672	.015793	-0.69	0.488	-.0419594	.0200251
NOO_SC3	-.021856	.0412939	-0.53	0.597	-.1028912	.0591792
NNOO_SC3	-.057248	.0366067	-1.56	0.118	-.1290849	.014589
OOL_SC12	.020743	.0262067	0.79	0.429	-.0306851	.072171
PJE_SC4	-.0261986	.01989	-1.32	0.188	-.0652308	.0128336
Trust_Science_SC4	-.0365378	.032917	-1.11	0.267	-.1011341	.0280586
Trust_in_media	-.016535	.0240309	-0.69	0.492	-.0636933	.0306233
Impulsivity_SC4	-.1069513	.0294385	-3.63	0.000	-.1647214	-.0491812
NegEmo_SC6	.0023783	.0202291	0.12	0.906	-.0373193	.0420759
SN_SC7	.0259997	.0228977	1.14	0.256	-.0189348	.0709343
CTC_SC7	.5269948	.0488106	10.80	0.000	.4312088	.6227807
OTC_SC7	-.0112358	.0160817	-0.70	0.485	-.0427945	.0203229
_cons	.5190459	.3529136	1.47	0.142	-.1735118	1.211604

```
244 .
245 .
246 . *****************
247 .
248 . *8. Compare Rsq models
249 .
250 . *8.a.1. model 1 vs model 2
251 . lrtest model_1 model_2

    Likelihood-ratio test                              LR chi2(2)  =      75.35
    (Assumption: model_1 nested in model_2)            Prob > chi2 =     0.0000

252 .
253 . *8.a.2. model 2 vs model 3
254 . lrtest model_2 model_3

    Likelihood-ratio test                              LR chi2(4)  =     195.55
    (Assumption: model_2 nested in model_3)            Prob > chi2 =     0.0000

255 .
256 . *8.a.3. model 3 vs model 4
257 . lrtest model_3 model_4

    Likelihood-ratio test                              LR chi2(6)  =     159.39
    (Assumption: model_3 nested in model_4)            Prob > chi2 =     0.0000

258 .
259 . *8.a.4. model 4 vs model 5
260 . lrtest model_4 model_5

    Likelihood-ratio test                              LR chi2(4)  =      19.47
    (Assumption: model_4 nested in model_5)            Prob > chi2 =     0.0006
```

```
261 .
262 . *8.a.5. model 5 vs model 6
263 . lrtest model_5 model_6

    Likelihood-ratio test                                 LR chi2(1)  =     36.64
    (Assumption: model_5 nested in model_6)               Prob > chi2 =    0.0000

264 .
265 . *8.a.6. model 6 vs model 7
266 . lrtest model_6 model_7

    Likelihood-ratio test                                 LR chi2(2)  =    192.06
    (Assumption: model_6 nested in model_7)               Prob > chi2 =    0.0000

267 .
268 .
269 . ****************
270 .
271 . log close
          name:  <unnamed>
           log:  C:\Users\creinde\OneDrive - UvA\RESEARCH\2020\20 03 Coronavirus-measures compliance survey\Data\US\NWO US
      log type:  smcl
     closed on:  17 Jun 2021, 22:50:46
```
