# Supplementary material for: Social distancing in America: Understanding long-term adherence to COVID-19 mitigation recommendations
Source: PLoS One. 2021 Sep 24;16(9):e0257945. doi: 10.1371/journal.pone.0257945 (PMC8462713; doi:10.1371/journal.pone.0257945)
Supplement: S2 Output — (PDF) [file pone.0257945.s010.pdf]

```
  ___  ____  ____  ____  ____(R)
 /__    /   ____/   /   ____/
___/   /   /___/   /   /___/
  Statistics/Data Analysis
```

```
      name:  <unnamed>
       log:  C:\Users\creinde\OneDrive - UvA\RESEARCH\2020\20 03 Coronavirus-measures compliance survey\Data\US\NWO US
  log type:  smcl
 opened on:  17 Jun 2021, 22:51:46

 1 . use "C:\Users\creinde\OneDrive - UvA\RESEARCH\2020\20 03 Coronavirus-measures compliance survey\Data\US\NWO US Summe

 2 .
 3 .
 4 .
 5 . ************************************************************************************
 6 . ************************************************************************************
 7 . *A. SELECTION CRITERION:
 8 .
 9 . * - Only if provided consent
10 . * - No missing data
11 . * - Both checks correct
12 . * - Nonbinary gender excluded (insufficient number)
13 .
14 . gen chris_sample_reqs = 1 if Consent == 1 & N_Missing == 0 & NChecksRight == 2 & Gender < 3
   (725 missing values generated)

15 .
16 . *B. SELECTION CRITERION:
17 .
18 . * - Only if provided consent
19 . * - Both checks correct
20 .
21 . *gen chris_sample_reqs = 1 if Consent == 1 & NChecksRight == 2
22 .
23 . *C. SELECTION CRITERION:
24 .
25 . * - Only if provided consent
26 . * - No missing data
27 .
28 . *gen chris_sample_reqs = 1 if Consent == 1 & N_Missing == 0
29 .
30 .
31 . tab chris_sample_reqs
```

chris_sampl e_reqs	Freq.	Percent	Cum.
1	**986**	**100.00**	**100.00**
Total	**986**	**100.00**	

```
32 .
33 .
34 . *generate insurance dummies
35 .
36 . gen Insurance_Public = 0

37 . replace Insurance_Public = 1 if (Insurance == 1)
   (415 real changes made)

38 .
39 . gen Insurance_Private = 0

40 . replace Insurance_Private = 1 if (Insurance == 2)
   (803 real changes made)
```

```
41 .
42 .
43 . *generate Geographic region dummies
44 .
45 . gen GeoCensus = 0

46 . replace GeoCensus = 1 if (Province == 8|Province == 22|Province == 24|Province == 32|Province == 44|Province == 50|P
   (329 real changes made)

47 . replace GeoCensus = 2 if (Province == 16|Province == 17|Province == 25|Province == 39|Province == 55|Province == 18|
   > 6)
   (283 real changes made)

48 . replace GeoCensus = 3 if (Province == 9|Province == 11|Province == 12|Province == 23|Province == 36|Province == 45|P
   > rovince == 47|Province == 5|Province == 21|Province == 40|Province == 48)
   (666 real changes made)

49 . replace GeoCensus = 4 if (Province == 4|Province == 7|Province == 15|Province == 29|Province == 31|Province == 34|Pr
   > vince == 53)
   (265 real changes made)

50 .
51 . gen GeoCensus_d1 = 0

52 . gen GeoCensus_d2 = 0

53 . gen GeoCensus_d3 = 0

54 .
55 . replace GeoCensus_d1 = 1 if (GeoCensus == 2)
   (283 real changes made)

56 . replace GeoCensus_d2 = 1 if (GeoCensus == 3)
   (666 real changes made)

57 . replace GeoCensus_d3 = 1 if (GeoCensus == 4)
   (265 real changes made)

58 .
59 .
60 . ********************************************************************************
61 . ********************************************************************************
62 . ********************************************************************************
63 . ********************************************************************************
64 .
65 . *HIERARCHICAL MODEL
66 .
67 . ********************************************************************************
68 . ********************************************************************************
69 . ********************************************************************************
70 . ********************************************************************************
71 .
72 . *1. Step 1: covariates only
73 .
74 . *1.a.1 Descriptive Statistics
75 . sum DV_Compliance_SC7  Age i.Gender_Female i.Minority Education i.Employed i.Corona_care i.Insurance_Public i.Insura
   > vative_other i.GeoCensus_d1 i.GeoCensus_d2 i.GeoCensus_d3 if chris_sample_reqs == 1
```

Variable	Obs	Mean	Std. Dev.	Min	Max
DV_Complia~7	**986**	**5.788757**	**1.286931**	**1**	**7**
Age	**986**	**40.17039**	**13.41275**	**18**	**79**
Gender_Fem~e					
0	**986**	**.4574037**	**.4984351**	**0**	**1**
1	**986**	**.5425963**	**.4984351**	**0**	**1**
Minority					
0	**986**	**.6146045**	**.4869356**	**0**	**1**
1	**986**	**.3853955**	**.4869356**	**0**	**1**
Education	**986**	**3.883367**	**1.59045**	**1**	**8**

Employed					
0	986	.3600406	.4802554	0	1
1	986	.6399594	.4802554	0	1
Corona_care					
0	986	.8985801	.3020371	0	1
1	986	.1014199	.3020371	0	1
Insurance_~c					
0	986	.7271805	.4456348	0	1
1	986	.2728195	.4456348	0	1
Insurance_~e					
0	986	.4219067	.4941144	0	1
1	986	.5780933	.4941144	0	1
SES_before	986	5.995943	2.102448	1	10
SES_change	986	-.1977688	1.591358	-9	9
Health_self					
1	986	.6764706	.4680602	0	1
2	986	.3235294	.4680602	0	1
Health_other					
1	986	.4472617	.4974632	0	1
2	986	.5527383	.4974632	0	1
Conservat~01					
0	986	.5628803	.496282	0	1
1	986	.4371197	.496282	0	1
Conservati~r					
0	986	.8924949	.3099115	0	1
1	986	.1075051	.3099115	0	1
GeoCensus_d1					
0	986	.8032454	.397747	0	1
1	986	.1967546	.397747	0	1
GeoCensus_d2					
0	986	.5750507	.4945862	0	1
1	986	.4249493	.4945862	0	1
GeoCensus_d3					
0	986	.8275862	.3779314	0	1
1	986	.1724138	.3779314	0	1

```
76 .
77 . *1.a.2 Regression
78 . reg DV_Compliance_SC7  Age i.Gender_Female i.Minority Education i.Employed i.Corona_care i.Insurance_Public i.Insura
   > vative_other i.GeoCensus_d1 i.GeoCensus_d2 i.GeoCensus_d3 if chris_sample_reqs == 1
```

Source	SS	df	MS
Model	121.66882	17	7.15698943
Residual	1509.67919	968	1.55958594
Total	1631.34802	985	1.65619088

Number of obs	=	986
F(17, 968)	=	4.59
Prob > F	=	0.0000
R-squared	=	0.0746
Adj R-squared	=	0.0583
Root MSE	=	1.2488

DV_Compliance_SC7	Coef.	Std. Err.	t	P>|t|	[95% Conf.	Interval]
Age	.012339	.0031212	3.95	0.000	.006214	.018464
1.Gender_Female	.2722874	.0833456	3.27	0.001	.1087285	.4358464
1.Minority	.2293174	.0872207	2.63	0.009	.058154	.4004808
Education	.0176458	.0273492	0.65	0.519	-.0360248	.0713163
1.Employed	.0051881	.0916031	0.06	0.955	-.1745755	.1849516
1.Corona_care	-.203348	.1385087	-1.47	0.142	-.47516	.0684639
1.Insurance_Public	.202807	.1322939	1.53	0.126	-.0568089	.4624229
1.Insurance_Private	.1828516	.1229904	1.49	0.137	-.0585069	.4242101
SES_before	.0336078	.0211148	1.59	0.112	-.0078283	.0750439
SES_change	-.030163	.0268709	-1.12	0.262	-.0828951	.022569
2.Health_self	.1502476	.0960509	1.56	0.118	-.0382444	.3387395
2.Health_other	.0578276	.0895038	0.65	0.518	-.1178163	.2334716
1.Conservative_01	-.2854025	.0869615	-3.28	0.001	-.4560573	-.1147477
1.Conservative_other	-.2155051	.1384781	-1.56	0.120	-.4872569	.0562467
1.GeoCensus_d1	-.4206096	.12726	-3.31	0.001	-.6703469	-.1708723
1.GeoCensus_d2	-.3080147	.1085164	-2.84	0.005	-.5209691	-.0950602
1.GeoCensus_d3	-.0983258	.1317751	-0.75	0.456	-.3569237	.1602721
_cons	4.935191	.2379619	20.74	0.000	4.46821	5.402171

```
79 . estimates store model_1

80 .
81 . *1.a.3 Check hettest: Run this right after your regression to apply the Breusch-Pagan / Cook-Weisberg test for heter
82 . *if significant, then you need to run the regression with vce(ro) at the end
83 . estat hettest

Breusch-Pagan / Cook-Weisberg test for heteroskedasticity
         Ho: Constant variance
         Variables: fitted values of DV_Compliance_SC7

         chi2(1)      =    52.71
         Prob > chi2  =   0.0000

84 .
85 . *1.a.4. check vif, to check for for multicollinearity (VIFs >10 are problematic)
86 . vif
```

Variable	VIF	1/VIF
Age	1.11	0.903457
1.Gender_F~e	1.09	0.917466
1.Minority	1.14	0.877791
Education	1.19	0.836843
1.Employed	1.22	0.818103
1.Corona_c~e	1.11	0.904687
1.Insuranc~c	2.20	0.455549
1.Insuran~te	2.33	0.428722
SES_before	1.24	0.803430
SES_change	1.15	0.865907
2.Health_s~f	1.28	0.783370
2.Health_o~r	1.25	0.798671
1.Conserv~01	1.18	0.850084
1.Conserva~r	1.16	0.859678
1.GeoCensu~1	1.62	0.617981
1.GeoCensu~2	1.82	0.549666
1.GeoCensu~3	1.57	0.638381
Mean VIF	1.39	

```
87 .
88 . *1.a.5. Effect size
89 . estat esize
```

Effect sizes for linear models

Source	Eta-Squared	df	[95% Conf.	Interval]
Model	.0745818	17	.0319906	.0917406
Age	.0158892	1	.0040436	.0348727
Gender_Female	.0109057	1	.0017412	.0274406
Minority	.0070904	1	.0004435	.0212756
Education	.0004299	1	.	.0068948
Employed	3.31e-06	1	.	.0012188
Corona_care	.0022217	1	.	.0119751
Insurance_Public	.0024219	1	.	.0124275
Insurance_Private	.0022782	1	.	.0121039
SES_before	.0026103	1	.	.0128435
SES_change	.0013	1	.	.0097013
Health_self	.0025214	1	.	.0126482
Health_other	.000431	1	.	.0068999
Conservative_01	.0110048	1	.0017813	.0275941
Conservative_other	.0024957	1	.	.0125915
GeoCensus_d1	.011159	1	.0018443	.0278324
GeoCensus_d2	.0082542	1	.0007829	.0232178
GeoCensus_d3	.0005748	1	.	.007467

Note: Eta-Squared values for individual model terms are partial.

```
90 .
91 . *1.a.6 Regression with vce(ro)
92 . reg DV_Compliance_SC7  Age i.Gender_Female i.Minority Education i.Employed i.Corona_care i.Insurance_Public i.Insura
> vative_other i.GeoCensus_d1 i.GeoCensus_d2 i.GeoCensus_d3 if chris_sample_reqs == 1, vce(ro)
```

Linear regression

Number of obs	=	986
F(17, 968)	=	4.78
Prob > F	=	0.0000
R-squared	=	0.0746
Root MSE	=	1.2488

DV_Compliance_SC7	Coef.	Robust Std. Err.	t	P>|t|	[95% Conf.	Interval]
Age	.012339	.0030129	4.10	0.000	.0064264	.0182517
1.Gender_Female	.2722874	.0822318	3.31	0.001	.1109143	.4336605
1.Minority	.2293174	.0868365	2.64	0.008	.058908	.3997269
Education	.0176458	.0262229	0.67	0.501	-.0338145	.0691061
1.Employed	.0051881	.091361	0.06	0.955	-.1741003	.1844764
1.Corona_care	-.203348	.1462587	-1.39	0.165	-.4903687	.0836726
1.Insurance_Public	.202807	.1493917	1.36	0.175	-.0903619	.495976
1.Insurance_Private	.1828516	.1305828	1.40	0.162	-.0734065	.4391097
SES_before	.0336078	.0242823	1.38	0.167	-.0140443	.0812599
SES_change	-.030163	.0295316	-1.02	0.307	-.0881164	.0277903
2.Health_self	.1502476	.0952304	1.58	0.115	-.0366343	.3371294
2.Health_other	.0578276	.0926929	0.62	0.533	-.1240745	.2397297
1.Conservative_01	-.2854025	.0861687	-3.31	0.001	-.4545015	-.1163035
1.Conservative_other	-.2155051	.1472206	-1.46	0.144	-.5044134	.0734033
1.GeoCensus_d1	-.4206096	.135282	-3.11	0.002	-.6860894	-.1551297
1.GeoCensus_d2	-.3080147	.1062304	-2.90	0.004	-.5164831	-.0995462
1.GeoCensus_d3	-.0983258	.116431	-0.84	0.399	-.3268121	.1301605
_cons	4.935191	.248628	19.85	0.000	4.447279	5.423103

```
 93 .
 94 .
 95 . *****************
 96 .
 97 . *2. Step 2: Add practical knowledge and understanding
 98 .
 99 . *2.a.1 Descriptive Statistics
100 . sum DV_Compliance_SC7  Age i.Gender_Female i.Minority Education i.Employed i.Corona_care i.Insurance_Public i.Insura
    > vative_other i.GeoCensus_d1 i.GeoCensus_d2 i.GeoCensus_d3 i.Current_measures Measures_clear if chris_sample_reqs ==
```

Variable	Obs	Mean	Std. Dev.	Min	Max
DV_Complia~7	986	5.788757	1.286931	1	7
Age	986	40.17039	13.41275	18	79
Gender_Fem~e					
0	986	.4574037	.4984351	0	1
1	986	.5425963	.4984351	0	1
Minority					
0	986	.6146045	.4869356	0	1
1	986	.3853955	.4869356	0	1
Education	986	3.883367	1.59045	1	8
Employed					
0	986	.3600406	.4802554	0	1
1	986	.6399594	.4802554	0	1
Corona_care					
0	986	.8985801	.3020371	0	1
1	986	.1014199	.3020371	0	1
Insurance_~c					
0	986	.7271805	.4456348	0	1
1	986	.2728195	.4456348	0	1
Insurance_~e					
0	986	.4219067	.4941144	0	1
1	986	.5780933	.4941144	0	1
SES_before	986	5.995943	2.102448	1	10
SES_change	986	-.1977688	1.591358	-9	9
Health_self					
1	986	.6764706	.4680602	0	1
2	986	.3235294	.4680602	0	1
Health_other					
1	986	.4472617	.4974632	0	1
2	986	.5527383	.4974632	0	1
Conservat~01					
0	986	.5628803	.496282	0	1
1	986	.4371197	.496282	0	1
Conservati~r					
0	986	.8924949	.3099115	0	1
1	986	.1075051	.3099115	0	1
GeoCensus_d1					
0	986	.8032454	.397747	0	1
1	986	.1967546	.397747	0	1
GeoCensus_d2					
0	986	.5750507	.4945862	0	1
1	986	.4249493	.4945862	0	1
GeoCensus_d3					

0	986	.8275862	.3779314	0	1
1	986	.1724138	.3779314	0	1
Current_me~s					
0	986	.1713996	.377049	0	1
Yes	986	.8286004	.377049	0	1
Measures_c~r	986	5.148073	1.736553	1	7

```
101 .
102 . *2.a.2 Regression
103 . reg DV_Compliance_SC7  Age i.Gender_Female i.Minority Education i.Employed i.Corona_care i.Insurance_Public i.Insura
    > vative_other i.GeoCensus_d1 i.GeoCensus_d2 i.GeoCensus_d3 i.Current_measures Measures_clear if chris_sample_reqs ==
```

Source	SS	df	MS
Model	262.482326	19	13.8148593
Residual	1368.86569	966	1.41704523
Total	1631.34802	985	1.65619088

Number of obs	=	986
F(19, 966)	=	9.75
Prob > F	=	0.0000
R-squared	=	0.1609
Adj R-squared	=	0.1444
Root MSE	=	1.1904

DV_Compliance_SC7	Coef.	Std. Err.	t	P>|t|	[95% Conf.	Interval]
Age	.0068922	.0030287	2.28	0.023	.0009486	.0128358
1.Gender_Female	.2379153	.0795435	2.99	0.003	.0818173	.3940134
1.Minority	.1551547	.0834952	1.86	0.063	-.0086981	.3190075
Education	.0427565	.0261916	1.63	0.103	-.0086426	.0941556
1.Employed	.00237	.0873624	0.03	0.978	-.1690719	.1738119
1.Corona_care	-.3074384	.1326191	-2.32	0.021	-.5676931	-.0471837
1.Insurance_Public	.1387906	.1262689	1.10	0.272	-.1090024	.3865836
1.Insurance_Private	.1234371	.1174163	1.05	0.293	-.1069833	.3538574
SES_before	.0179847	.020259	0.89	0.375	-.0217721	.0577415
SES_change	-.0254801	.0256343	-0.99	0.320	-.0757854	.0248252
2.Health_self	.1996953	.0917143	2.18	0.030	.0197131	.3796775
2.Health_other	.0156448	.0857037	0.18	0.855	-.1525421	.1838317
1.Conservative_01	-.260824	.0829519	-3.14	0.002	-.4236107	-.0980373
1.Conservative_other	-.1443381	.1322552	-1.09	0.275	-.4038787	.1152025
1.GeoCensus_d1	-.3616552	.1214513	-2.98	0.003	-.5999941	-.1233164
1.GeoCensus_d2	-.2081454	.1039285	-2.00	0.045	-.412097	-.0041938
1.GeoCensus_d3	-.0187351	.1258627	-0.15	0.882	-.2657308	.2282607
Current_measures						
Yes	.7185127	.1054595	6.81	0.000	.5115565	.9254689
Measures_clear	.129586	.0231211	5.60	0.000	.0842127	.1749594
_cons	3.921167	.249563	15.71	0.000	3.431419	4.410916

```
104 . estimates store model_2

105 .
106 . *2.a.3 Check hettest: Run this right after your regression to apply the Breusch-Pagan / Cook-Weisberg test for heter
107 . *if significant, then you need to run the regression with vce(ro) at the end
108 . estat hettest

    Breusch-Pagan / Cook-Weisberg test for heteroskedasticity
             Ho: Constant variance
             Variables: fitted values of DV_Compliance_SC7

             chi2(1)      =    93.83
             Prob > chi2  =   0.0000
```

109 .
110 . *2.a.4. check vif, to check for for multicollinearity (VIFs >10 are problematic)
111 . vif

Variable	VIF	1/VIF
Age	1.15	0.871757
1.Gender_F~e	1.09	0.915209
1.Minority	1.15	0.870326
Education	1.21	0.829052
1.Employed	1.22	0.817249
1.Corona_c~e	1.12	0.896633
1.Insuranc~c	2.20	0.454356
1.Insuran~te	2.34	0.427401
SES_before	1.26	0.792976
SES_change	1.16	0.864508
2.Health_s~f	1.28	0.780675
2.Health_o~r	1.26	0.791455
1.Conserv~01	1.18	0.848864
1.Conserva~r	1.17	0.856341
1.GeoCensu~1	1.62	0.616495
1.GeoCensu~2	1.84	0.544496
1.GeoCensu~3	1.57	0.635811
1.Current_~s	1.10	0.909872
Measures_c~r	1.12	0.892391
Mean VIF	1.37	

112 .
113 . *2.a.5. Effect size
114 . estat esize

Effect sizes for linear models

Source	Eta-Squared	df	[95% Conf.	Interval]
Model	.160899	19	.1059389	.1864617
Age	.0053321	1	.0000287	.0182132
Gender_Female	.009176	1	.0010846	.0247337
Minority	.0035619	1	.	.0148469
Education	.0027511	1	.	.0131633
Employed	7.62e-07	1	.	.
Corona_care	.0055325	1	.0000702	.018575
Insurance_Public	.0012491	1	.	.0095754
Insurance_Private	.0011428	1	.	.0092796
SES_before	.0008152	1	.	.0082995
SES_change	.0010217	1	.	.0089311
Health_self	.0048838	1	.	.0173913
Health_other	.0000345	1	.	.0036562
Conservative_01	.0101308	1	.0014324	.0262512
Conservative_other	.0012315	1	.	.0095269
GeoCensus_d1	.0090958	1	.0010568	.0246047
GeoCensus_d2	.0041351	1	.	.0159755
GeoCensus_d3	.0000229	1	.	.0032248
Current_measures	.0458498	1	.0235146	.0739931
Measures_clear	.0314938	1	.0134108	.0560171

Note: Eta-Squared values for individual model terms are partial.

115 .

```
116 . *2.a.6 Regression with vce(ro)
117 . reg DV_Compliance_SC7  Age i.Gender_Female i.Minority Education i.Employed i.Corona_care i.Insurance_Public i.Insura
    > vative_other i.GeoCensus_d1 i.GeoCensus_d2 i.GeoCensus_d3 i.Current_measures Measures_clear if chris_sample_reqs ==

    Linear regression                               Number of obs     =        986
                                                    F(19, 966)        =       8.29
                                                    Prob > F          =     0.0000
                                                    R-squared         =     0.1609
                                                    Root MSE          =     1.1904

    ------------------------------------------------------------------------------------
                          |               Robust
       DV_Compliance_SC7  |      Coef.   Std. Err.      t    P>|t|     [95% Conf. Interval]
    ----------------------+-------------------------------------------------------------
                      Age |   .0068922   .0030017     2.30   0.022     .0010017    .0127827
          1.Gender_Female |   .2379153   .0795656     2.99   0.003     .0817739    .3940568
               1.Minority |   .1551547   .0835431     1.86   0.064    -.0087921    .3191015
                Education |   .0427565   .0253326     1.69   0.092    -.0069568    .0924698
               1.Employed |     .00237   .0882783     0.03   0.979    -.1708693    .1756093
            1.Corona_care |  -.3074384   .1392725    -2.21   0.028    -.5807498   -.034127
       1.Insurance_Public |   .1387906   .1413153     0.98   0.326    -.1385299     .416111
      1.Insurance_Private |   .1234371   .1243212     0.99   0.321    -.1205338    .3674079
               SES_before |   .0179847   .0223689     0.80   0.422    -.0259125    .0618819
               SES_change |  -.0254801   .0276426    -0.92   0.357    -.0797267    .0287664
            2.Health_self |   .1996953   .0898681     2.22   0.027      .023336    .3760545
           2.Health_other |   .0156448   .0859678     0.18   0.856    -.1530603    .1843499
        1.Conservative_01 |   -.260824   .0815657    -3.20   0.001    -.4208903   -.1007577
     1.Conservative_other |  -.1443381   .1357239    -1.06   0.288    -.4106859    .1220096
           1.GeoCensus_d1 |  -.3616552   .1314955    -2.75   0.006    -.6197051   -.1036054
           1.GeoCensus_d2 |  -.2081454   .1015943    -2.05   0.041    -.4075163   -.0087745
           1.GeoCensus_d3 |  -.0187351   .1144475    -0.16   0.870    -.2433295    .2058593
                          |
         Current_measures |
                     Yes  |   .7185127   .1281867     5.61   0.000     .4669562    .9700692
           Measures_clear |    .129586   .0250382     5.18   0.000     .0804506    .1787215
                    _cons |   3.921167   .2673481    14.67   0.000     3.396517    4.445817
    ------------------------------------------------------------------------------------

118 .
119 .
120 . *****************
121 .
122 . *3. Step 3: Add costs + benefits
123 .
124 . *3.a.1 Descriptive Statistics
125 . sum DV_Compliance_SC7  Age i.Gender_Female i.Minority Education i.Employed i.Corona_care i.Insurance_Public i.Insura
    > vative_other i.GeoCensus_d1 i.GeoCensus_d2 i.GeoCensus_d3 i.Current_measures Measures_clear MA_Perc_Threat_SC3 Costs

        Variable |        Obs        Mean    Std. Dev.       Min        Max
    -------------+---------------------------------------------------------
    DV_Complia~7 |        986    5.788757    1.286931          1          7
             Age |        986    40.17039    13.41275         18         79
                 |
    Gender_Fem~e |
               0 |        986    .4574037    .4984351          0          1
               1 |        986    .5425963    .4984351          0          1
                 |
        Minority |
               0 |        986    .6146045    .4869356          0          1
    -------------+---------------------------------------------------------
               1 |        986    .3853955    .4869356          0          1
                 |
       Education |        986    3.883367     1.59045          1          8
                 |
        Employed |
               0 |        986    .3600406    .4802554          0          1
               1 |        986    .6399594    .4802554          0          1
                 |
     Corona_care |
               0 |        986    .8985801    .3020371          0          1
    -------------+---------------------------------------------------------
               1 |        986    .1014199    .3020371          0          1
                 |
    Insurance_~c |
```

```
          0 |        986    .7271805    .4456348          0          1
          1 |        986    .2728195    .4456348          0          1
            |
Insurance_~e |
          0 |        986    .4219067    .4941144          0          1
          1 |        986    .5780933    .4941144          0          1
-------------+---------------------------------------------------------
            |
  SES_before |        986    5.995943    2.102448          1         10
  SES_change |        986   -.1977688    1.591358         -9          9
            |
 Health_self |
          1 |        986    .6764706    .4680602          0          1
          2 |        986    .3235294    .4680602          0          1
            |
Health_other |
          1 |        986    .4472617    .4974632          0          1
-------------+---------------------------------------------------------
          2 |        986    .5527383    .4974632          0          1
            |
Conservat~01 |
          0 |        986    .5628803     .496282          0          1
          1 |        986    .4371197     .496282          0          1
            |
Conservati~r |
          0 |        986    .8924949    .3099115          0          1
          1 |        986    .1075051    .3099115          0          1
-------------+---------------------------------------------------------
            |
GeoCensus_d1 |
          0 |        986    .8032454     .397747          0          1
          1 |        986    .1967546     .397747          0          1
            |
GeoCensus_d2 |
          0 |        986    .5750507    .4945862          0          1
          1 |        986    .4249493    .4945862          0          1
            |
GeoCensus_d3 |
          0 |        986    .8275862    .3779314          0          1
-------------+---------------------------------------------------------
          1 |        986    .1724138    .3779314          0          1
            |
Current_me~s |
          0 |        986    .1713996     .377049          0          1
        Yes |        986    .8286004     .377049          0          1
            |
Measures_c~r |        986    5.148073    1.736553          1          7
MA_Perc_Th~3 |        986    5.532454    1.555847          1          7
-------------+---------------------------------------------------------
   Costs_SC5 |        986    4.088641    1.658638          1          7
Deterr_SD_~2 |        986    3.188641    1.782006          1          7
Deterr_SD_~e |        986     3.80426    1.728008          1          6

126 .
127 . *3.a.2 Regression
128 . reg DV_Compliance_SC7  Age i.Gender_Female i.Minority Education i.Employed i.Corona_care i.Insurance_Public i.Insura
    > vative_other i.GeoCensus_d1 i.GeoCensus_d2 i.GeoCensus_d3 i.Current_measures Measures_clear MA_Perc_Threat_SC3 Costs

      Source |       SS           df       MS      Number of obs   =       986
-------------+----------------------------------   F(23, 962)      =     21.15
       Model |  547.89011        23  23.8213091   Prob > F        =    0.0000
    Residual |  1083.45791       962  1.12625562   R-squared       =    0.3359
-------------+----------------------------------   Adj R-squared   =    0.3200
       Total |  1631.34802       985  1.65619088   Root MSE        =    1.0613
```

DV_Compliance_SC7	Coef.	Std. Err.	t	P>|t|	[95% Conf.	Interval]
Age	.0053769	.0027778	1.94	0.053	-.0000744	.0108283
1.Gender_Female	.2085115	.0712381	2.93	0.004	.0687114	.3483115
1.Minority	.0394854	.0752948	0.52	0.600	-.1082755	.1872463
Education	.0389176	.023377	1.66	0.096	-.0069581	.0847934
1.Employed	.0112094	.0779869	0.14	0.886	-.1418347	.1642535
1.Corona_care	-.3492803	.1199066	-2.91	0.004	-.584589	-.1139715
1.Insurance_Public	.0950507	.1129133	0.84	0.400	-.1265341	.3166354
1.Insurance_Private	.1543112	.1056177	1.46	0.144	-.0529565	.3615789
SES_before	.0075088	.0183244	0.41	0.682	-.0284516	.0434692
SES_change	-.0073458	.0232111	-0.32	0.752	-.052896	.0382044
2.Health_self	-.0376796	.0834712	-0.45	0.652	-.2014863	.1261272
2.Health_other	-.0626331	.0774889	-0.81	0.419	-.2146999	.0894338
1.Conservative_01	-.0588368	.0752966	-0.78	0.435	-.2066013	.0889276
1.Conservative_other	.0027931	.1183874	0.02	0.981	-.2295343	.2351205
1.GeoCensus_d1	-.2513708	.1085585	-2.32	0.021	-.4644096	-.0383321
1.GeoCensus_d2	-.1710111	.0927527	-1.84	0.066	-.353032	.0110099
1.GeoCensus_d3	-.0666652	.1123769	-0.59	0.553	-.2871973	.153867
Current_measures						
Yes	.4558885	.0956791	4.76	0.000	.2681246	.6436523
Measures_clear	.0630335	.0211881	2.97	0.003	.0214533	.1046138
MA_Perc_Threat_SC3	.3876513	.0249468	15.54	0.000	.3386949	.4366077
Costs_SC5	.0249756	.0232655	1.07	0.283	-.0206812	.0706325
Deterr_SD_Likely_SC2	-.0228317	.0223156	-1.02	0.307	-.0666246	.0209613
Deterr_SD_Severe	.0013997	.0212129	0.07	0.947	-.0402291	.0430285
_cons	2.484213	.2809221	8.84	0.000	1.932922	3.035504

```
129 . estimates store model_3

130 .
131 . *3.a.3 Check hettest: Run this right after your regression to apply the Breusch-Pagan / Cook-Weisberg test for heter
132 . *if significant, then you need to run the regression with vce(ro) at the end
133 . estat hettest

    Breusch-Pagan / Cook-Weisberg test for heteroskedasticity
             Ho: Constant variance
             Variables: fitted values of DV_Compliance_SC7

             chi2(1)      =   196.35
             Prob > chi2  =   0.0000

134 .
135 . *3.a.4. check vif, to check for for multicollinearity (VIFs >10 are problematic)
136 . vif
```

Variable	VIF	1/VIF
Age	1.21	0.823661
1.Gender_F~e	1.10	0.906898
1.Minority	1.18	0.850606
Education	1.21	0.827151
1.Employed	1.23	0.815103
1.Corona_c~e	1.15	0.871754
1.Insuranc~c	2.21	0.451599
1.Insuran~te	2.38	0.419829
SES_before	1.30	0.770355
SES_change	1.19	0.838058
2.Health_s~f	1.33	0.749072
2.Health_o~r	1.30	0.769484
1.Conserv~01	1.22	0.818829
1.Conserva~r	1.18	0.849404
1.GeoCensu~1	1.63	0.613281
1.GeoCensu~2	1.84	0.543330
1.GeoCensu~3	1.58	0.633900
1.Current_~s	1.14	0.878559
Measures_c~r	1.18	0.844579
MA_Perc_Th~3	1.32	0.758991
Costs_SC5	1.30	0.767847
Deterr_SD_~2	1.38	0.723042
Deterr_SD_~e	1.18	0.850963

Mean VIF	1.38

```
137 .
138 . *3.a.5. Effect size
139 . estat esize
```

Effect sizes for linear models

Source	Eta-Squared	df	[95% Conf.	Interval]
Model	.3358512	23	.2745941	.3642734
Age	.0038796	1	.	.0155096
Gender_Female	.0088269	1	.0009571	.0242098
Minority	.0002858	1	.	.0062417
Education	.0028727	1	.	.0134534
Employed	.0000215	1	.	.0031644
Corona_care	.0087432	1	.0009292	.0240741
Insurance_Public	.0007361	1	.	.0080648
Insurance_Private	.002214	1	.	.0119995
SES_before	.0001745	1	.	.0055419
SES_change	.0001041	1	.	.0048987
Health_self	.0002118	1	.	.0058037
Health_other	.0006787	1	.	.0078704
Conservative_01	.0006343	1	.	.0077153
Conservative_other	5.79e-07	1	.	.
GeoCensus_d1	.0055426	1	.0000678	.0186278
GeoCensus_d2	.0035212	1	.	.0147961
GeoCensus_d3	.0003657	1	.	.0066405
Current_measures	.0230557	1	.0080218	.04494
Measures_clear	.009116	1	.0010552	.0246767
MA_Perc_Threat_SC3	.2006408	1	.1581298	.2434948
Costs_SC5	.0011965	1	.	.0094551
Deterr_SD_Likely_SC2	.0010869	1	.	.0091451
Deterr_SD_Severe	4.53e-06	1	.	.001543

Note: Eta-Squared values for individual model terms are partial.

```
140 .
141 . *3.a.6 Regression with vce(ro)
142 . reg DV_Compliance_SC7  Age i.Gender_Female i.Minority Education i.Employed i.Corona_care i.Insurance_Public i.Insura
    > vative_other i.GeoCensus_d1 i.GeoCensus_d2 i.GeoCensus_d3 i.Current_measures Measures_clear MA_Perc_Threat_SC3 Costs
```

Linear regression

Number of obs	=	986
F(23, 962)	=	13.56
Prob > F	=	0.0000
R-squared	=	0.3359
Root MSE	=	1.0613

DV_Compliance_SC7	Coef.	Robust Std. Err.	t	P>|t|	[95% Conf.	Interval]
Age	.0053769	.0026805	2.01	0.045	.0001166	.0106373
1.Gender_Female	.2085115	.0711944	2.93	0.003	.0687973	.3482256
1.Minority	.0394854	.075544	0.52	0.601	-.1087647	.1877354
Education	.0389176	.0228924	1.70	0.089	-.0060072	.0838425
1.Employed	.0112094	.0790882	0.14	0.887	-.1439958	.1664147
1.Corona_care	-.3492803	.1240593	-2.82	0.005	-.5927383	-.1058223
1.Insurance_Public	.0950507	.1227688	0.77	0.439	-.145875	.3359763
1.Insurance_Private	.1543112	.1108891	1.39	0.164	-.0633013	.3719237
SES_before	.0075088	.0206106	0.36	0.716	-.0329382	.0479557
SES_change	-.0073458	.0250462	-0.29	0.769	-.0564972	.0418056
2.Health_self	-.0376796	.0761012	-0.50	0.621	-.187023	.1116639
2.Health_other	-.0626331	.0757878	-0.83	0.409	-.2113615	.0860954
1.Conservative_01	-.0588368	.0732048	-0.80	0.422	-.2024964	.0848227
1.Conservative_other	.0027931	.1284709	0.02	0.983	-.2493225	.2549086
1.GeoCensus_d1	-.2513708	.1159645	-2.17	0.030	-.4789435	-.0237982
1.GeoCensus_d2	-.1710111	.0907315	-1.88	0.060	-.3490656	.0070435
1.GeoCensus_d3	-.0666652	.0991265	-0.67	0.501	-.2611944	.127864
Current_measures						

Yes	.4558885	.1099432	4.15	0.000	.2401323	.6716446
Measures_clear	.0630335	.0218201	2.89	0.004	.0202131	.105854
MA_Perc_Threat_SC3	.3876513	.0332655	11.65	0.000	.32237	.4529326
Costs_SC5	.0249756	.0225047	1.11	0.267	-.0191884	.0691397
Deterr_SD_Likely_SC2	-.0228317	.0209501	-1.09	0.276	-.0639449	.0182816
Deterr_SD_Severe	.0013997	.0217459	0.06	0.949	-.0412751	.0440746
_cons	2.484213	.303909	8.17	0.000	1.887812	3.080614

```
143 .
144 .
145 . *****************
146 .
147 . *4. Step 4: Add legitimacy
148 .
149 . *4.a.1 Descriptive Statistics
150 . sum DV_Compliance_SC7  Age i.Gender_Female i.Minority Education i.Employed i.Corona_care i.Insurance_Public i.Insura
    > vative_other i.GeoCensus_d1 i.GeoCensus_d2 i.GeoCensus_d3 i.Current_measures Measures_clear MA_Perc_Threat_SC3 Costs
    > C3 OOL_SC12 PJE_SC4 if chris_sample_reqs == 1
```

Variable	Obs	Mean	Std. Dev.	Min	Max
DV_Complia~7	986	5.788757	1.286931	1	7
Age	986	40.17039	13.41275	18	79
Gender_Fem~e					
0	986	.4574037	.4984351	0	1
1	986	.5425963	.4984351	0	1
Minority					
0	986	.6146045	.4869356	0	1
1	986	.3853955	.4869356	0	1
Education	986	3.883367	1.59045	1	8
Employed					
0	986	.3600406	.4802554	0	1
1	986	.6399594	.4802554	0	1
Corona_care					
0	986	.8985801	.3020371	0	1
1	986	.1014199	.3020371	0	1
Insurance_~c					
0	986	.7271805	.4456348	0	1
1	986	.2728195	.4456348	0	1
Insurance_~e					
0	986	.4219067	.4941144	0	1
1	986	.5780933	.4941144	0	1
SES_before	986	5.995943	2.102448	1	10
SES_change	986	-.1977688	1.591358	-9	9
Health_self					
1	986	.6764706	.4680602	0	1
2	986	.3235294	.4680602	0	1
Health_other					
1	986	.4472617	.4974632	0	1
2	986	.5527383	.4974632	0	1
Conservat~01					
0	986	.5628803	.496282	0	1
1	986	.4371197	.496282	0	1
Conservati~r					
0	986	.8924949	.3099115	0	1
1	986	.1075051	.3099115	0	1

GeoCensus_d1					
0	986	.8032454	.397747	0	1
1	986	.1967546	.397747	0	1
GeoCensus_d2					
0	986	.5750507	.4945862	0	1
1	986	.4249493	.4945862	0	1
GeoCensus_d3					
0	986	.8275862	.3779314	0	1
1	986	.1724138	.3779314	0	1
Current_me~s					
0	986	.1713996	.377049	0	1
Yes	986	.8286004	.377049	0	1
Measures_c~r	986	5.148073	1.736553	1	7
MA_Perc_Th~3	986	5.532454	1.555847	1	7
Costs_SC5	986	4.088641	1.658638	1	7
Deterr_SD_~2	986	3.188641	1.782006	1	7
Deterr_SD_~e	986	3.80426	1.728008	1	6
MA_MoralBe~f	986	6.10142	1.342722	1	7
MA_Authori~2	986	4.356491	1.837274	1	7
NOO_SC3	986	3.838404	.9147702	1	5
NNOO_SC3	986	2.970926	1.020185	1	5
OOL_SC12	986	4.293949	1.500592	1	7
PJE_SC4	986	5.057049	1.675777	1	7

```
151 .
152 . *4.a.2 Regression
153 . reg DV_Compliance_SC7  Age i.Gender_Female i.Minority Education i.Employed i.Corona_care i.Insurance_Public i.Insura
    > vative_other i.GeoCensus_d1 i.GeoCensus_d2 i.GeoCensus_d3 i.Current_measures Measures_clear MA_Perc_Threat_SC3 Costs
    > C3 OOL_SC12 PJE_SC4 if chris_sample_reqs == 1
```

Source	SS	df	MS			
				Number of obs	=	986
				F(29, 956)	=	22.82
Model	667.311621	29	23.0107455	Prob > F	=	0.0000
Residual	964.036394	956	1.00840627	R-squared	=	0.4091
				Adj R-squared	=	0.3911
Total	1631.34802	985	1.65619088	Root MSE	=	1.0042

DV_Compliance_SC7	Coef.	Std. Err.	t	P>|t|	[95% Conf.	Interval]
Age	.0052475	.0026759	1.96	0.050	-3.78e-06	.0104987
1.Gender_Female	.1372805	.0680981	2.02	0.044	.0036415	.2709195
1.Minority	.0467001	.0715317	0.65	0.514	-.0936772	.1870774
Education	.0287874	.0222126	1.30	0.195	-.0148036	.0723784
1.Employed	.05022	.0740841	0.68	0.498	-.0951663	.1956063
1.Corona_care	-.2260752	.115658	-1.95	0.051	-.4530481	.0008978
1.Insurance_Public	.077979	.1070154	0.73	0.466	-.1320331	.2879912
1.Insurance_Private	.143932	.100156	1.44	0.151	-.052619	.3404829
SES_before	-.0092416	.0178317	-0.52	0.604	-.0442354	.0257523
SES_change	-.021513	.0222354	-0.97	0.334	-.0651489	.0221229
2.Health_self	.0280132	.0795982	0.35	0.725	-.1281941	.1842206
2.Health_other	-.0535001	.0737504	-0.73	0.468	-.1982314	.0912313
1.Conservative_01	.0083061	.0739462	0.11	0.911	-.1368096	.1534217
1.Conservative_other	-.0024842	.1128646	-0.02	0.982	-.2239751	.2190066
1.GeoCensus_d1	-.1546497	.1036706	-1.49	0.136	-.358098	.0487987
1.GeoCensus_d2	-.1820024	.0883501	-2.06	0.040	-.3553849	-.0086198
1.GeoCensus_d3	-.0674906	.107038	-0.63	0.528	-.2775472	.142566
Current_measures						
Yes	.3416244	.0916803	3.73	0.000	.1617066	.5215423
Measures_clear	.0129824	.0218924	0.59	0.553	-.0299803	.055945
MA_Perc_Threat_SC3	.1795089	.0305752	5.87	0.000	.1195067	.2395112
Costs_SC5	.0255495	.0223085	1.15	0.252	-.0182297	.0693288
Deterr_SD_Likely_SC2	-.0065304	.0230509	-0.28	0.777	-.0517666	.0387058
Deterr_SD_Severe	.0015601	.0202603	0.08	0.939	-.0381997	.0413199
MA_MoralBelief	.3340053	.0351266	9.51	0.000	.2650712	.4029394

```
    MA_Authority_SC2 |     -.00088    .021718    -0.04   0.968    -.0435005    .0417405
             NOO_SC3 |    .0873527   .0444429     1.97   0.050     .0001358    .1745697
            NNOO_SC3 |    .0309378   .0396221     0.78   0.435    -.0468185    .1086941
            OOL_SC12 |    .0488995   .0244607     2.00   0.046     .0008966    .0969024
             PJE_SC4 |     .017787   .0220036     0.81   0.419     -.025394    .0609679
               _cons |    1.253896   .3129653     4.01   0.000     .6397175    1.868074
------------------------------------------------------------------------------------------

154 . estimates store model_4

155 .
156 . *4.a.3 Check hettest: Run this right after your regression to apply the Breusch-Pagan / Cook-Weisberg test for heter
157 . *if significant, then you need to run the regression with vce(ro) at the end
158 . estat hettest

Breusch-Pagan / Cook-Weisberg test for heteroskedasticity
         Ho: Constant variance
         Variables: fitted values of DV_Compliance_SC7

         chi2(1)      =   165.65
         Prob > chi2  =   0.0000

159 .
160 . *4.a.4. check vif, to check for for multicollinearity (VIFs >10 are problematic)
161 . vif
```

Variable	VIF	1/VIF
Age	1.26	0.794756
1.Gender_F~e	1.13	0.888611
1.Minority	1.19	0.843838
Education	1.22	0.820280
1.Employed	1.24	0.808731
1.Corona_c~e	1.19	0.838933
1.Insuranc~c	2.22	0.450141
1.Insuran~te	2.39	0.418014
SES_before	1.37	0.728385
SES_change	1.22	0.817659
2.Health_s~f	1.36	0.737547
2.Health_o~r	1.31	0.760586
1.Conserv~01	1.32	0.760169
1.Conserva~r	1.20	0.836775
1.GeoCensu~1	1.66	0.602107
1.GeoCensu~2	1.87	0.536168
1.GeoCensu~3	1.60	0.625601
1.Current_~s	1.17	0.856745
Measures_c~r	1.41	0.708332
MA_Perc_Th~3	2.21	0.452405
Costs_SC5	1.34	0.747750
Deterr_SD_~2	1.65	0.606744
Deterr_SD_~e	1.20	0.835250
MA_MoralBe~f	2.17	0.460209
MA_Authori~2	1.56	0.643001
NOO_SC3	1.61	0.619398
NNOO_SC3	1.60	0.626566
OOL_SC12	1.32	0.759863
PJE_SC4	1.33	0.752973
Mean VIF	1.49	

```
162 .
163 . *4.a.5. Effect size
```

```
164 . estat esize

    Effect sizes for linear models
```

Source	Eta-Squared	df	[95% Conf.	Interval]
Model	.4090553	29	.3464346	.4335717
Age	.0040065	1	.	.0158063
Gender_Female	.004233	1	.	.0162453
Minority	.0004456	1	.	.0070245
Education	.0017538	1	.	.0109481
Employed	.0004804	1	.	.0071679
Corona_care	.0039807	1	.	.0157559
Insurance_Public	.0005551	1	.	.0074592
Insurance_Private	.0021556	1	.	.0119071
SES_before	.0002809	1	.	.0062442
SES_change	.0009782	1	.	.0088628
Health_self	.0001295	1	.	.0051871
Health_other	.0005502	1	.	.0074405
Conservative_01	.0000132	1	.	.0026646
Conservative_other	5.07e-07	1	.	.
GeoCensus_d1	.0023223	1	.	.0122891
GeoCensus_d2	.0044194	1	.	.0166021
GeoCensus_d3	.0004157	1	.	.0068964
Current_measures	.0143161	1	.0032165	.0327169
Measures_clear	.0003677	1	.	.0066805
MA_Perc_Threat_SC3	.0348011	1	.0155657	.0603947
Costs_SC5	.0013702	1	.	.0099662
Deterr_SD_Likely_SC2	.0000839	1	.	.0046694
Deterr_SD_Severe	6.20e-06	1	.	.0018747
MA_MoralBelief	.0864035	1	.0554664	.1215249
MA_Authority_SC2	1.72e-06	1	.	.0005357
NOO_SC3	.0040247	1	.	.0158419
NNOO_SC3	.0006373	1	.	.0077599
OOL_SC12	.0041629	1	.	.0161102
PJE_SC4	.0006831	1	.	.0079197

Note: Eta-Squared values for individual model terms are partial.

```
165 .
166 . *4.a.6 Regression with vce(ro)
167 . reg DV_Compliance_SC7  Age i.Gender_Female i.Minority Education i.Employed i.Corona_care i.Insurance_Public i.Insura
    > vative_other i.GeoCensus_d1 i.GeoCensus_d2 i.GeoCensus_d3 i.Current_measures Measures_clear MA_Perc_Threat_SC3 Costs
    > C3 OOL_SC12 PJE_SC4 if chris_sample_reqs == 1, vce(ro)

    Linear regression                               Number of obs     =        986
                                                    F(29, 956)        =      15.04
                                                    Prob > F          =     0.0000
                                                    R-squared         =     0.4091
                                                    Root MSE          =     1.0042
```

DV_Compliance_SC7	Coef.	Robust Std. Err.	t	P>|t|	[95% Conf.	Interval]
Age	.0052475	.002643	1.99	0.047	.0000607	.0104342
1.Gender_Female	.1372805	.0671853	2.04	0.041	.0054327	.2691283
1.Minority	.0467001	.0723141	0.65	0.519	-.0952125	.1886128
Education	.0287874	.0211475	1.36	0.174	-.0127134	.0702883
1.Employed	.05022	.0752229	0.67	0.505	-.0974011	.1978411
1.Corona_care	-.2260752	.1240159	-1.82	0.069	-.46945	.0172996
1.Insurance_Public	.077979	.1136978	0.69	0.493	-.1451471	.3011052
1.Insurance_Private	.143932	.1057742	1.36	0.174	-.0636445	.3515084
SES_before	-.0092416	.0194087	-0.48	0.634	-.0473302	.0288471
SES_change	-.021513	.0236699	-0.91	0.364	-.0679638	.0249379
2.Health_self	.0280132	.0741143	0.38	0.706	-.1174322	.1734587
2.Health_other	-.0535001	.0725993	-0.74	0.461	-.1959724	.0889723
1.Conservative_01	.0083061	.0733709	0.11	0.910	-.1356805	.1522927
1.Conservative_other	-.0024842	.1192766	-0.02	0.983	-.2365584	.2315899
1.GeoCensus_d1	-.1546497	.108637	-1.42	0.155	-.3678442	.0585449
1.GeoCensus_d2	-.1820024	.0859638	-2.12	0.035	-.3507019	-.0133029
1.GeoCensus_d3	-.0674906	.0970473	-0.70	0.487	-.257941	.1229598

Current_measures						
Yes	.3416244	.1048658	3.26	0.001	.1358308	.5474181
Measures_clear	.0129824	.0224526	0.58	0.563	-.0310798	.0570445
MA_Perc_Threat_SC3	.1795089	.0363968	4.93	0.000	.1080822	.2509357
Costs_SC5	.0255495	.0220589	1.16	0.247	-.0177398	.0688389
Deterr_SD_Likely_SC2	-.0065304	.0215844	-0.30	0.762	-.0488886	.0358279
Deterr_SD_Severe	.0015601	.0205581	0.08	0.940	-.0387841	.0419042
MA_MoralBelief	.3340053	.0476212	7.01	0.000	.2405513	.4274594
MA_Authority_SC2	-.00088	.0211709	-0.04	0.967	-.0424268	.0406668
NOO_SC3	.0873527	.0465579	1.88	0.061	-.0040148	.1787202
NNOO_SC3	.0309378	.0377832	0.82	0.413	-.0432098	.1050853
OOL_SC12	.0488995	.0254558	1.92	0.055	-.0010563	.0988553
PJE_SC4	.017787	.0226204	0.79	0.432	-.0266045	.0621785
_cons	1.253896	.3419857	3.67	0.000	.5827666	1.925025

```
168 .
169 .
170 . *****************
171 .
172 . *5. Step 5: Add personal factors
173 .
174 . *5.a.1 Descriptive Statistics
175 . sum DV_Compliance_SC7  Age i.Gender_Female i.Minority Education i.Employed i.Corona_care i.Insurance_Public i.Insura
    > vative_other i.GeoCensus_d1 i.GeoCensus_d2 i.GeoCensus_d3 i.Current_measures Measures_clear MA_Perc_Threat_SC3 Costs
    > C3 OOL_SC12 PJE_SC4 Trust_Science_SC4 Trust_in_media Impulsivity_SC4 NegEmo_SC6 if chris_sample_reqs == 1
```

Variable	Obs	Mean	Std. Dev.	Min	Max
DV_Complia~7	986	5.788757	1.286931	1	7
Age	986	40.17039	13.41275	18	79
Gender_Fem~e					
0	986	.4574037	.4984351	0	1
1	986	.5425963	.4984351	0	1
Minority					
0	986	.6146045	.4869356	0	1
1	986	.3853955	.4869356	0	1
Education	986	3.883367	1.59045	1	8
Employed					
0	986	.3600406	.4802554	0	1
1	986	.6399594	.4802554	0	1
Corona_care					
0	986	.8985801	.3020371	0	1
1	986	.1014199	.3020371	0	1
Insurance_~c					
0	986	.7271805	.4456348	0	1
1	986	.2728195	.4456348	0	1
Insurance_~e					
0	986	.4219067	.4941144	0	1
1	986	.5780933	.4941144	0	1
SES_before	986	5.995943	2.102448	1	10
SES_change	986	-.1977688	1.591358	-9	9
Health_self					
1	986	.6764706	.4680602	0	1
2	986	.3235294	.4680602	0	1
Health_other					
1	986	.4472617	.4974632	0	1
2	986	.5527383	.4974632	0	1

Conservat~01					
0	986	.5628803	.496282	0	1
1	986	.4371197	.496282	0	1
Conservati~r					
0	986	.8924949	.3099115	0	1
1	986	.1075051	.3099115	0	1
GeoCensus_d1					
0	986	.8032454	.397747	0	1
1	986	.1967546	.397747	0	1
GeoCensus_d2					
0	986	.5750507	.4945862	0	1
1	986	.4249493	.4945862	0	1
GeoCensus_d3					
0	986	.8275862	.3779314	0	1
1	986	.1724138	.3779314	0	1
Current_me~s					
0	986	.1713996	.377049	0	1
Yes	986	.8286004	.377049	0	1
Measures_c~r	986	5.148073	1.736553	1	7
MA_Perc_Th~3	986	5.532454	1.555847	1	7
Costs_SC5	986	4.088641	1.658638	1	7
Deterr_SD_~2	986	3.188641	1.782006	1	7
Deterr_SD_~e	986	3.80426	1.728008	1	6
MA_MoralBe~f	986	6.10142	1.342722	1	7
MA_Authori~2	986	4.356491	1.837274	1	7
NOO_SC3	986	3.838404	.9147702	1	5
NNOO_SC3	986	2.970926	1.020185	1	5
OOL_SC12	986	4.293949	1.500592	1	7
PJE_SC4	986	5.057049	1.675777	1	7
Trust_Scie~4	986	3.833671	.9938757	1	5
Trust_in_m~a	986	2.936105	1.297	1	5
Impulsivi~C4	986	2.517241	1.138374	1	5
NegEmo_SC6	986	4.526707	1.607009	1	7

```
176 .
177 . *5.a.2 Regression
178 . reg DV_Compliance_SC7  Age i.Gender_Female i.Minority Education i.Employed i.Corona_care i.Insurance_Public i.Insura
    > vative_other i.GeoCensus_d1 i.GeoCensus_d2 i.GeoCensus_d3 i.Current_measures Measures_clear MA_Perc_Threat_SC3 Costs
    > C3 OOL_SC12 PJE_SC4 Trust_Science_SC4 Trust_in_media Impulsivity_SC4 NegEmo_SC6 if chris_sample_reqs == 1
```

Source	SS	df	MS			
				Number of obs	=	986
				F(33, 952)	=	20.59
Model	679.363401	33	20.5867697	Prob > F	=	0.0000
Residual	951.984614	952	.999983838	R-squared	=	0.4164
				Adj R-squared	=	0.3962
Total	1631.34802	985	1.65619088	Root MSE	=	.99999

DV_Compliance_SC7	Coef.	Std. Err.	t	P>|t|	[95% Conf.	Interval]
Age	.0054999	.0027313	2.01	0.044	.0001397	.01086
1.Gender_Female	.1178268	.0685568	1.72	0.086	-.0167131	.2523667
1.Minority	.0611048	.0721409	0.85	0.397	-.0804688	.2026784
Education	.025045	.0222511	1.13	0.261	-.0186219	.0687118
1.Employed	.0417997	.0742156	0.56	0.573	-.1038453	.1874447
1.Corona_care	-.1864778	.1159633	-1.61	0.108	-.414051	.0410954
1.Insurance_Public	.08826	.1068474	0.83	0.409	-.1214236	.2979437
1.Insurance_Private	.1559849	.0999696	1.56	0.119	-.0402014	.3521711
SES_before	-.0029182	.0179564	-0.16	0.871	-.0381569	.0323205
SES_change	-.0184261	.022228	-0.83	0.407	-.0620478	.0251955
2.Health_self	.0240442	.0794081	0.30	0.762	-.1317911	.1798794
2.Health_other	-.0607897	.0736325	-0.83	0.409	-.2052906	.0837112
1.Conservative_01	.022825	.0766573	0.30	0.766	-.1276118	.1732618

```
1.Conservative_other |    .027014   .1148754     0.24   0.814    -.1984243    .2524522
       1.GeoCensus_d1 |  -.1393906   .1034502    -1.35   0.178    -.3424074    .0636261
       1.GeoCensus_d2 |  -.1624056   .0885481    -1.83   0.067    -.3361775    .0113664
       1.GeoCensus_d3 |  -.0679587    .106724    -0.64   0.524    -.2774002    .1414827
                      |
     Current_measures |
                 Yes  |    .336997   .0913966     3.69   0.000     .1576349    .5163592
       Measures_clear |   .0110545   .0222047     0.50   0.619    -.0325213    .0546302
    MA_Perc_Threat_SC3 |    .170483   .0312603     5.45   0.000      .109136    .2318301
            Costs_SC5 |    .014951   .0231053     0.65   0.518    -.0303923    .0602943
 Deterr_SD_Likely_SC2 |   .0008911   .0232003     0.04   0.969    -.0446386    .0464208
     Deterr_SD_Severe |   .0049145   .0202852     0.24   0.809    -.0348944    .0447233
       MA_MoralBelief |   .3274326   .0353267     9.27   0.000     .2581055    .3967597
     MA_Authority_SC2 |   .0060353   .0217707     0.28   0.782    -.0366889    .0487595
              NOO_SC3 |   .0721731   .0448868     1.61   0.108    -.0159154    .1602615
             NNOO_SC3 |    .036263   .0403929     0.90   0.370    -.0430064    .1155324
             OOL_SC12 |   .0269772   .0262764     1.03   0.305    -.0245892    .0785435
              PJE_SC4 |   .0190094   .0221371     0.86   0.391    -.0244337    .0624525
    Trust_Science_SC4 |   .0498738    .042172     1.18   0.237    -.0328871    .1326347
       Trust_in_media |  -.0099748   .0301547    -0.33   0.741    -.0691522    .0492026
      Impulsivity_SC4 |  -.0923994    .036157    -2.56   0.011     -.163356   -.0214428
           NegEmo_SC6 |   .0495496   .0235791     2.10   0.036     .0032766    .0958226
                _cons |   1.259495   .3397361     3.71   0.000     .5927773    1.926214
------------------------------------------------------------------------------------------

179 . estimates store model_5

180 . 
181 . *5.a.3 Check hettest: Run this right after your regression to apply the Breusch-Pagan / Cook-Weisberg test for heter
182 . *if significant, then you need to run the regression with vce(ro) at the end
183 . estat hettest

Breusch-Pagan / Cook-Weisberg test for heteroskedasticity 
         Ho: Constant variance
         Variables: fitted values of DV_Compliance_SC7

         chi2(1)      =   158.55
         Prob > chi2  =   0.0000

184 . 
185 . *5.a.4. check vif, to check for for multicollinearity (VIFs >10 are problematic)
186 . vif

    Variable |       VIF       1/VIF  
-------------+----------------------
         Age |      1.32    0.756430
1.Gender_F~e |      1.15    0.869438
  1.Minority |      1.22    0.822717
   Education |      1.23    0.810615
  1.Employed |      1.25    0.799139
1.Corona_c~e |      1.21    0.827552
1.Insuranc~c |      2.23    0.447786
1.Insuran~te |      2.40    0.416069
  SES_before |      1.40    0.712307
  SES_change |      1.23    0.811370
2.Health_s~f |      1.36    0.734892
2.Health_o~r |      1.32    0.756650
1.Conserv~01 |      1.43    0.701443
1.Conserva~r |      1.25    0.800990
1.GeoCensu~1 |      1.67    0.599626
1.GeoCensu~2 |      1.89    0.529316
1.GeoCensu~3 |      1.60    0.624032
1.Current_~s |      1.17    0.854871
Measures_c~r |      1.46    0.682799
MA_Perc_Th~3 |      2.33    0.429177
   Costs_SC5 |      1.45    0.691240
Deterr_SD_~2 |      1.68    0.593950
Deterr_SD_~e |      1.21    0.826241
MA_MoralBe~f |      2.22    0.451211
MA_Authori~2 |      1.58    0.634545
     NOO_SC3 |      1.66    0.602138
    NNOO_SC3 |      1.67    0.597845
    OOL_SC12 |      1.53    0.652981
     PJE_SC4 |      1.36    0.737706
```

Trust_Scie~4	1.73	0.577887
Trust_in_m~a	1.51	0.663692
Impulsivi~C4	1.67	0.599243
NegEmo_SC6	1.41	0.707075
Mean VIF	1.54	

```
187 .
188 . *5.a.5. Effect size
189 . estat esize
```

Effect sizes for linear models

Source	Eta-Squared	df	[95% Conf.	Interval]
Model	.416443	33	.3514903	.4385926
Age	.004241	1	.	.0162937
Gender_Female	.0030932	1	.	.0139945
Minority	.000753	1	.	.0081794
Education	.001329	1	.	.0098818
Employed	.0003331	1	.	.006535
Corona_care	.0027089	1	.	.0131756
Insurance_Public	.0007162	1	.	.0080561
Insurance_Private	.0025508	1	.	.0128294
SES_before	.0000277	1	.	.0034596
SES_change	.0007213	1	.	.0080732
Health_self	.0000963	1	.	.0048446
Health_other	.0007154	1	.	.0080534
Conservative_01	.0000931	1	.	.0048051
Conservative_other	.0000581	1	.	.0042654
GeoCensus_d1	.0019034	1	.	.01134
GeoCensus_d2	.0035211	1	.	.0148743
GeoCensus_d3	.0004257	1	.	.0069611
Current_measures	.0140798	1	.0030889	.0324136
Measures_clear	.0002603	1	.	.0061481
MA_Perc_Threat_SC3	.0302955	1	.0125141	.054666
Costs_SC5	.0004396	1	.	.0070205
Deterr_SD_Likely_SC2	1.55e-06	1	.	.0004258
Deterr_SD_Severe	.0000616	1	.	.004332
MA_MoralBelief	.0827713	1	.0523933	.1174955
MA_Authority_SC2	.0000807	1	.	.0046386
NOO_SC3	.0027083	1	.	.0131743
NNOO_SC3	.0008459	1	.	.00848
OOL_SC12	.001106	1	.	.009262
PJE_SC4	.000774	1	.	.0082483
Trust_Science_SC4	.001467	1	.	.0102478
Trust_in_media	.0001149	1	.	.0050565
Impulsivity_SC4	.0068132	1	.0003477	.0209498
NegEmo_SC6	.0046172	1	.	.0170105

Note: Eta-Squared values for individual model terms are partial.

```
190 .
191 . *5.a.6 Regression with vce(ro)
192 . reg DV_Compliance_SC7  Age i.Gender_Female i.Minority Education i.Employed i.Corona_care i.Insurance_Public i.Insura
    > vative_other i.GeoCensus_d1 i.GeoCensus_d2 i.GeoCensus_d3 i.Current_measures Measures_clear MA_Perc_Threat_SC3 Costs
    > C3 OOL_SC12 PJE_SC4 Trust_Science_SC4 Trust_in_media Impulsivity_SC4 NegEmo_SC6 if chris_sample_reqs == 1, vce(ro)
```

Linear regression

Number of obs	=	986
F(33, 952)	=	14.25
Prob > F	=	0.0000
R-squared	=	0.4164
Root MSE	=	.99999

DV_Compliance_SC7	Coef.	Robust Std. Err.	t	P>|t|	[95% Conf.	Interval]
Age	.0054999	.0027189	2.02	0.043	.0001642	.0108355
1.Gender_Female	.1178268	.0670366	1.76	0.079	-.0137298	.2493834
1.Minority	.0611048	.0728242	0.84	0.402	-.0818097	.2040193
Education	.025045	.0211162	1.19	0.236	-.0163946	.0664846
1.Employed	.0417997	.0752788	0.56	0.579	-.1059318	.1895313
1.Corona_care	-.1864778	.1244819	-1.50	0.134	-.4307684	.0578127
1.Insurance_Public	.08826	.1135686	0.78	0.437	-.1346136	.3111337
1.Insurance_Private	.1559849	.1057804	1.47	0.141	-.0516049	.3635746
SES_before	-.0029182	.01908	-0.15	0.878	-.0403618	.0345254
SES_change	-.0184261	.0239026	-0.77	0.441	-.0653341	.0284818
2.Health_self	.0240442	.0742388	0.32	0.746	-.1216464	.1697347
2.Health_other	-.0607897	.073495	-0.83	0.408	-.2050206	.0834412
1.Conservative_01	.022825	.0750998	0.30	0.761	-.1245553	.1702053
1.Conservative_other	.027014	.1262459	0.21	0.831	-.2207385	.2747664
1.GeoCensus_d1	-.1393906	.1084634	-1.29	0.199	-.3522456	.0734644
1.GeoCensus_d2	-.1624056	.0848938	-1.91	0.056	-.3290062	.0041951
1.GeoCensus_d3	-.0679587	.0964307	-0.70	0.481	-.2572	.1212826
Current_measures						
Yes	.336997	.104612	3.22	0.001	.1317003	.5422937
Measures_clear	.0110545	.0227352	0.49	0.627	-.0335625	.0556714
MA_Perc_Threat_SC3	.170483	.0376467	4.53	0.000	.0966029	.2443632
Costs_SC5	.014951	.0229615	0.65	0.515	-.0301101	.060012
Deterr_SD_Likely_SC2	.0008911	.0216869	0.04	0.967	-.0416687	.0434508
Deterr_SD_Severe	.0049145	.0209885	0.23	0.815	-.0362747	.0461036
MA_MoralBelief	.3274326	.0480144	6.82	0.000	.2332063	.421659
MA_Authority_SC2	.0060353	.0210492	0.29	0.774	-.0352729	.0473435
NOO_SC3	.0721731	.0464445	1.55	0.121	-.0189723	.1633184
NNOO_SC3	.036263	.0394339	0.92	0.358	-.0411243	.1136503
OOL_SC12	.0269772	.0279668	0.96	0.335	-.0279066	.081861
PJE_SC4	.0190094	.0222604	0.85	0.393	-.0246756	.0626944
Trust_Science_SC4	.0498738	.0450008	1.11	0.268	-.0384383	.1381859
Trust_in_media	-.0099748	.0286792	-0.35	0.728	-.0662566	.0463069
Impulsivity_SC4	-.0923994	.0362727	-2.55	0.011	-.1635831	-.0212157
NegEmo_SC6	.0495496	.0253726	1.95	0.051	-.0002431	.0993423
_cons	1.259495	.3638363	3.46	0.001	.5454816	1.973509

```
193 .
194 .
195 . *****************
196 .
197 . *6. Step 6: Add social environment
198 .
199 . *6.a.1 Descriptive Statistics
200 . sum DV_Compliance_SC7  Age i.Gender_Female i.Minority Education i.Employed i.Corona_care i.Insurance_Public i.Insura
    > vative_other i.GeoCensus_d1 i.GeoCensus_d2 i.GeoCensus_d3 i.Current_measures Measures_clear MA_Perc_Threat_SC3 Costs
    > C3 OOL_SC12 PJE_SC4 Trust_Science_SC4 Trust_in_media Impulsivity_SC4 NegEmo_SC6 SN_SC7 if chris_sample_reqs == 1
```

Variable	Obs	Mean	Std. Dev.	Min	Max
DV_Complia~7	986	5.788757	1.286931	1	7
Age	986	40.17039	13.41275	18	79
Gender_Fem~e					
0	986	.4574037	.4984351	0	1
1	986	.5425963	.4984351	0	1
Minority					
0	986	.6146045	.4869356	0	1
1	986	.3853955	.4869356	0	1
Education	986	3.883367	1.59045	1	8
Employed					
0	986	.3600406	.4802554	0	1
1	986	.6399594	.4802554	0	1
Corona_care					

0	986	.8985801	.3020371	0	1
1	986	.1014199	.3020371	0	1
Insurance_~c					
0	986	.7271805	.4456348	0	1
1	986	.2728195	.4456348	0	1
Insurance_~e					
0	986	.4219067	.4941144	0	1
1	986	.5780933	.4941144	0	1
SES_before	986	5.995943	2.102448	1	10
SES_change	986	-.1977688	1.591358	-9	9
Health_self					
1	986	.6764706	.4680602	0	1
2	986	.3235294	.4680602	0	1
Health_other					
1	986	.4472617	.4974632	0	1
2	986	.5527383	.4974632	0	1
Conservat~01					
0	986	.5628803	.496282	0	1
1	986	.4371197	.496282	0	1
Conservati~r					
0	986	.8924949	.3099115	0	1
1	986	.1075051	.3099115	0	1
GeoCensus_d1					
0	986	.8032454	.397747	0	1
1	986	.1967546	.397747	0	1
GeoCensus_d2					
0	986	.5750507	.4945862	0	1
1	986	.4249493	.4945862	0	1
GeoCensus_d3					
0	986	.8275862	.3779314	0	1
1	986	.1724138	.3779314	0	1
Current_me~s					
0	986	.1713996	.377049	0	1
Yes	986	.8286004	.377049	0	1
Measures_c~r	986	5.148073	1.736553	1	7
MA_Perc_Th~3	986	5.532454	1.555847	1	7
Costs_SC5	986	4.088641	1.658638	1	7
Deterr_SD_~2	986	3.188641	1.782006	1	7
Deterr_SD_~e	986	3.80426	1.728008	1	6
MA_MoralBe~f	986	6.10142	1.342722	1	7
MA_Authori~2	986	4.356491	1.837274	1	7
NOO_SC3	986	3.838404	.9147702	1	5
NNOO_SC3	986	2.970926	1.020185	1	5
OOL_SC12	986	4.293949	1.500592	1	7
PJE_SC4	986	5.057049	1.675777	1	7
Trust_Scie~4	986	3.833671	.9938757	1	5
Trust_in_m~a	986	2.936105	1.297	1	5
Impulsivi~C4	986	2.517241	1.138374	1	5
NegEmo_SC6	986	4.526707	1.607009	1	7
SN_SC7	986	5.213272	1.400333	1	7

```
201 .
202 . *6.a.2 Regression
203 . reg DV_Compliance_SC7  Age i.Gender_Female i.Minority Education i.Employed i.Corona_care i.Insurance_Public i.Insura
    > vative_other i.GeoCensus_d1 i.GeoCensus_d2 i.GeoCensus_d3 i.Current_measures Measures_clear MA_Perc_Threat_SC3 Costs
    > C3 OOL_SC12 PJE_SC4 Trust_Science_SC4 Trust_in_media Impulsivity_SC4 NegEmo_SC6 SN_SC7 if chris_sample_reqs == 1
```

Source	SS	df	MS			
				Number of obs	=	986
				F(34, 951)	=	21.21
Model	703.492321	34	20.6909506	Prob > F	=	0.0000
Residual	927.855694	951	.97566319	R-squared	=	0.4312
				Adj R-squared	=	0.4109
Total	1631.34802	985	1.65619088	Root MSE	=	.98776

DV_Compliance_SC7	Coef.	Std. Err.	t	P>|t|	[95% Conf.	Interval]
Age	.0044332	.0027064	1.64	0.102	-.0008781	.0097444
1.Gender_Female	.1178056	.067718	1.74	0.082	-.0150884	.2506995
1.Minority	.0525713	.0712789	0.74	0.461	-.0873108	.1924534
Education	.021863	.0219881	0.99	0.320	-.0212879	.0650139
1.Employed	.0476216	.0733169	0.65	0.516	-.09626	.1915031
1.Corona_care	-.1642564	.1146316	-1.43	0.152	-.3892165	.0607036
1.Insurance_Public	.0923077	.1055432	0.87	0.382	-.1148168	.2994322
1.Insurance_Private	.1622896	.0987546	1.64	0.101	-.0315124	.3560917
SES_before	-.0071682	.0177573	-0.40	0.687	-.0420162	.0276798
SES_change	-.0184695	.0219561	-0.84	0.400	-.0615574	.0246184
2.Health_self	.0431701	.0785308	0.55	0.583	-.1109436	.1972837
2.Health_other	-.0670784	.0727426	-0.92	0.357	-.209833	.0756762
1.Conservative_01	.033355	.075749	0.44	0.660	-.1152994	.1820095
1.Conservative_other	.0529432	.1135896	0.47	0.641	-.1699719	.2758584
1.GeoCensus_d1	-.1351474	.102188	-1.32	0.186	-.3356874	.0653927
1.GeoCensus_d2	-.1412743	.0875678	-1.61	0.107	-.3131227	.0305742
1.GeoCensus_d3	-.0431328	.1055363	-0.41	0.683	-.2502438	.1639782
Current_measures						
Yes	.3121893	.0904161	3.45	0.001	.1347512	.4896274
Measures_clear	.0069214	.0219487	0.32	0.753	-.0361521	.0499949
MA_Perc_Threat_SC3	.1748902	.0308905	5.66	0.000	.1142687	.2355118
Costs_SC5	.0104421	.0228406	0.46	0.648	-.0343818	.055266
Deterr_SD_Likely_SC2	-.010443	.0230295	-0.45	0.650	-.0556376	.0347515
Deterr_SD_Severe	-.0051744	.0201394	-0.26	0.797	-.0446973	.0343485
MA_MoralBelief	.3108386	.0350536	8.87	0.000	.2420472	.3796299
MA_Authority_SC2	-.0068517	.0216599	-0.32	0.752	-.0493585	.035655
NOO_SC3	.0624162	.0443809	1.41	0.160	-.0246797	.1495121
NNOO_SC3	.0103939	.0402364	0.26	0.796	-.0685684	.0893562
OOL_SC12	.0208462	.0259842	0.80	0.423	-.0301467	.0718391
PJE_SC4	.0083276	.0219715	0.38	0.705	-.0347905	.0514458
Trust_Science_SC4	.0458528	.0416639	1.10	0.271	-.035911	.1276166
Trust_in_media	-.0151485	.0298039	-0.51	0.611	-.0736375	.0433406
Impulsivity_SC4	-.104732	.0358006	-2.93	0.004	-.1749892	-.0344747
NegEmo_SC6	.0431717	.0233259	1.85	0.065	-.0026044	.0889479
SN_SC7	.1275747	.0256534	4.97	0.000	.0772308	.1779186
_cons	1.196436	.3358188	3.56	0.000	.5374043	1.855467

```
204 . estimates store model_6

205 .
206 . *6.a.3 Check hettest: Run this right after your regression to apply the Breusch-Pagan / Cook-Weisberg test for heter
207 . *if significant, then you need to run the regression with vce(ro) at the end
208 . estat hettest

    Breusch-Pagan / Cook-Weisberg test for heteroskedasticity
             Ho: Constant variance
             Variables: fitted values of DV_Compliance_SC7

             chi2(1)      =   146.45
             Prob > chi2  =   0.0000
```

```
209 .
210 . *6.a.4. check vif, to check for for multicollinearity (VIFs >10 are problematic)
211 . vif
```

Variable	VIF	1/VIF
Age	1.33	0.751679
1.Gender_F~e	1.15	0.869438
1.Minority	1.22	0.822240
Education	1.23	0.809928
1.Employed	1.25	0.798935
1.Corona_c~e	1.21	0.826294
1.Insuranc~c	2.23	0.447759
1.Insuran~te	2.40	0.416001
SES_before	1.41	0.710657
SES_change	1.23	0.811369
2.Health_s~f	1.36	0.733129
2.Health_o~r	1.32	0.756422
1.Conserv~01	1.43	0.700895
1.Conserva~r	1.25	0.799303
1.GeoCensu~1	1.67	0.599584
1.GeoCensu~2	1.89	0.528069
1.GeoCensu~3	1.61	0.622635
1.Current_~s	1.17	0.852269
Measures_c~r	1.47	0.681820
MA_Perc_Th~3	2.33	0.428824
Costs_SC5	1.45	0.690151
Deterr_SD_~2	1.70	0.588133
Deterr_SD_~e	1.22	0.817857
MA_MoralBe~f	2.24	0.447122
MA_Authori~2	1.60	0.625463
NOO_SC3	1.66	0.600961
NNOO_SC3	1.70	0.587852
OOL_SC12	1.53	0.651511
PJE_SC4	1.37	0.730656
Trust_Scie~4	1.73	0.577670
Trust_in_m~a	1.51	0.662883
Impulsivi~C4	1.68	0.596367
NegEmo_SC6	1.42	0.704938
SN_SC7	1.30	0.767555
Mean VIF	1.54	

```
212 .
213 . *6.a.5. Effect size
214 . estat esize
```

Effect sizes for linear models

Source	Eta-Squared	df	[95% Conf.	Interval]
Model	.4312337	34	.366545	.4527218
Age	.0028134	1	.	.0134088
Gender_Female	.0031722	1	.	.0141671
Minority	.0005717	1	.	.0075493
Education	.0010385	1	.	.0090724
Employed	.0004434	1	.	.0070419
Corona_care	.0021544	1	.	.0119398
Insurance_Public	.0008037	1	.	.008351
Insurance_Private	.0028318	1	.	.0134482
SES_before	.0001713	1	.	.0055657
SES_change	.0007435	1	.	.0081536
Health_self	.0003177	1	.	.0064627
Health_other	.0008933	1	.	.0086347
Conservative_01	.0002038	1	.	.005801
Conservative_other	.0002284	1	.	.0059613
GeoCensus_d1	.0018358	1	.	.0111833
GeoCensus_d2	.0027294	1	.	.0132276
GeoCensus_d3	.0001756	1	.	.0055986
Current_measures	.0123809	1	.0023111	.029882
Measures_clear	.0001046	1	.	.0049464
MA_Perc_Threat_SC3	.0326064	1	.0140393	.0576643

Costs_SC5	.0002197	1	.	.0059062
Deterr_SD_Likely_SC2	.0002162	1	.	.0058831
Deterr_SD_Severe	.0000694	1	.	.0044693
MA_MoralBelief	.0763699	1	.0471251	.1102236
MA_Authority_SC2	.0001052	1	.	.0049539
NOO_SC3	.0020755	1	.	.0117557
NNOO_SC3	.0000702	1	.	.0044816
OOL_SC12	.0006763	1	.	.0079253
PJE_SC4	.000151	1	.	.0054016
Trust_Science_SC4	.001272	1	.	.0097334
Trust_in_media	.0002716	1	.	.0062171
Impulsivity_SC4	.0089188	1	.0009649	.0244678
NegEmo_SC6	.0035891	1	.	.0150194
SN_SC7	.0253459	1	.0093497	.0481654

Note: Eta-Squared values for individual model terms are partial.

```
215 .
216 . *6.a.6 Regression with vce(ro)
217 . reg DV_Compliance_SC7  Age i.Gender_Female i.Minority Education i.Employed i.Corona_care i.Insurance_Public i.Insura
    > vative_other i.GeoCensus_d1 i.GeoCensus_d2 i.GeoCensus_d3 i.Current_measures Measures_clear MA_Perc_Threat_SC3 Costs
    > C3 OOL_SC12 PJE_SC4 Trust_Science_SC4 Trust_in_media Impulsivity_SC4 NegEmo_SC6 SN_SC7 if chris_sample_reqs == 1, vc
```

Linear regression

Number of obs	=	986
F(34, 951)	=	15.56
Prob > F	=	0.0000
R-squared	=	0.4312
Root MSE	=	.98776

DV_Compliance_SC7	Coef.	Robust Std. Err.	t	P>|t|	[95% Conf.	Interval]
Age	.0044332	.002656	1.67	0.095	-.0007791	.0096454
1.Gender_Female	.1178056	.0661969	1.78	0.075	-.0121033	.2477144
1.Minority	.0525713	.0714635	0.74	0.462	-.0876731	.1928157
Education	.021863	.0209745	1.04	0.298	-.0192987	.0630247
1.Employed	.0476216	.0741709	0.64	0.521	-.097936	.1931791
1.Corona_care	-.1642564	.1200635	-1.37	0.172	-.3998765	.0713636
1.Insurance_Public	.0923077	.1131726	0.82	0.415	-.1297892	.3144047
1.Insurance_Private	.1622896	.1055185	1.54	0.124	-.0447864	.3693657
SES_before	-.0071682	.0188553	-0.38	0.704	-.0441709	.0298345
SES_change	-.0184695	.0235632	-0.78	0.433	-.0647113	.0277723
2.Health_self	.0431701	.0735946	0.59	0.558	-.1012566	.1875967
2.Health_other	-.0670784	.0729254	-0.92	0.358	-.2101916	.0760348
1.Conservative_01	.033355	.0741363	0.45	0.653	-.1121346	.1788447
1.Conservative_other	.0529432	.1243581	0.43	0.670	-.1911048	.2969913
1.GeoCensus_d1	-.1351474	.1050259	-1.29	0.198	-.3412567	.070962
1.GeoCensus_d2	-.1412743	.0826328	-1.71	0.088	-.3034379	.0208894
1.GeoCensus_d3	-.0431328	.0949422	-0.45	0.650	-.2294532	.1431876
Current_measures						
Yes	.3121893	.1039775	3.00	0.003	.1081375	.5162412
Measures_clear	.0069214	.0225553	0.31	0.759	-.0373426	.0511853
MA_Perc_Threat_SC3	.1748902	.0372252	4.70	0.000	.1018372	.2479433
Costs_SC5	.0104421	.0227003	0.46	0.646	-.0341064	.0549906
Deterr_SD_Likely_SC2	-.010443	.0215745	-0.48	0.628	-.0527822	.0318962
Deterr_SD_Severe	-.0051744	.0206707	-0.25	0.802	-.0457398	.035391
MA_MoralBelief	.3108386	.0478545	6.50	0.000	.216926	.4047512
MA_Authority_SC2	-.0068517	.0208425	-0.33	0.742	-.0477543	.0340508
NOO_SC3	.0624162	.0451733	1.38	0.167	-.0262348	.1510671
NNOO_SC3	.0103939	.0405805	0.26	0.798	-.0692437	.0900316
OOL_SC12	.0208462	.0274685	0.76	0.448	-.0330597	.074752
PJE_SC4	.0083276	.0218365	0.38	0.703	-.0345256	.0511809
Trust_Science_SC4	.0458528	.044054	1.04	0.298	-.0406015	.1323072
Trust_in_media	-.0151485	.0284497	-0.53	0.595	-.07098	.040683
Impulsivity_SC4	-.104732	.0356947	-2.93	0.003	-.1747815	-.0346824
NegEmo_SC6	.0431717	.0248747	1.74	0.083	-.0056439	.0919873
SN_SC7	.1275747	.0272722	4.68	0.000	.0740541	.1810953
_cons	1.196436	.3528073	3.39	0.001	.504065	1.888807

```
218 .
219 .
220 . *****************
221 .
222 . *7. Step 7: Add practical circumstances
223 .
224 . *7.a.1 Descriptive Statistics
225 . sum DV_Compliance_SC7  Age i.Gender_Female i.Minority Education i.Employed i.Corona_care i.Insurance_Public i.Insura
    > vative_other i.GeoCensus_d1 i.GeoCensus_d2 i.GeoCensus_d3 i.Current_measures Measures_clear MA_Perc_Threat_SC3 Costs
    > C3 OOL_SC12 PJE_SC4 Trust_Science_SC4 Trust_in_media Impulsivity_SC4 NegEmo_SC6 SN_SC7 CTC_SC7 OTC_SC7 if chris_samp
```

Variable	Obs	Mean	Std. Dev.	Min	Max
DV_Complia~7	986	5.788757	1.286931	1	7
Age	986	40.17039	13.41275	18	79
Gender_Fem~e					
0	986	.4574037	.4984351	0	1
1	986	.5425963	.4984351	0	1
Minority					
0	986	.6146045	.4869356	0	1
1	986	.3853955	.4869356	0	1
Education	986	3.883367	1.59045	1	8
Employed					
0	986	.3600406	.4802554	0	1
1	986	.6399594	.4802554	0	1
Corona_care					
0	986	.8985801	.3020371	0	1
1	986	.1014199	.3020371	0	1
Insurance_~c					
0	986	.7271805	.4456348	0	1
1	986	.2728195	.4456348	0	1
Insurance_~e					
0	986	.4219067	.4941144	0	1
1	986	.5780933	.4941144	0	1
SES_before	986	5.995943	2.102448	1	10
SES_change	986	-.1977688	1.591358	-9	9
Health_self					
1	986	.6764706	.4680602	0	1
2	986	.3235294	.4680602	0	1
Health_other					
1	986	.4472617	.4974632	0	1
2	986	.5527383	.4974632	0	1
Conservat~01					
0	986	.5628803	.496282	0	1
1	986	.4371197	.496282	0	1
Conservati~r					
0	986	.8924949	.3099115	0	1
1	986	.1075051	.3099115	0	1
GeoCensus_d1					
0	986	.8032454	.397747	0	1
1	986	.1967546	.397747	0	1
GeoCensus_d2					
0	986	.5750507	.4945862	0	1
1	986	.4249493	.4945862	0	1

GeoCensus_d3					
0	986	.8275862	.3779314	0	1
1	986	.1724138	.3779314	0	1
Current_me~s					
0	986	.1713996	.377049	0	1
Yes	986	.8286004	.377049	0	1
Measures_c~r	986	5.148073	1.736553	1	7
MA_Perc_Th~3	986	5.532454	1.555847	1	7
Costs_SC5	986	4.088641	1.658638	1	7
Deterr_SD_~2	986	3.188641	1.782006	1	7
Deterr_SD_~e	986	3.80426	1.728008	1	6
MA_MoralBe~f	986	6.10142	1.342722	1	7
MA_Authori~2	986	4.356491	1.837274	1	7
NOO_SC3	986	3.838404	.9147702	1	5
NNOO_SC3	986	2.970926	1.020185	1	5
OOL_SC12	986	4.293949	1.500592	1	7
PJE_SC4	986	5.057049	1.675777	1	7
Trust_Scie~4	986	3.833671	.9938757	1	5
Trust_in_m~a	986	2.936105	1.297	1	5
Impulsivi~C4	986	2.517241	1.138374	1	5
NegEmo_SC6	986	4.526707	1.607009	1	7
SN_SC7	986	5.213272	1.400333	1	7
CTC_SC7	986	5.972327	.9444972	1	7
OTC_SC7	986	4.697769	1.752303	1	7

```
226 .
227 . *7.a.2 Regression
228 . reg DV_Compliance_SC7  Age i.Gender_Female i.Minority Education i.Employed i.Corona_care i.Insurance_Public i.Insura
    > vative_other i.GeoCensus_d1 i.GeoCensus_d2 i.GeoCensus_d3 i.Current_measures Measures_clear MA_Perc_Threat_SC3 Costs
    > C3 OOL_SC12 PJE_SC4 Trust_Science_SC4 Trust_in_media Impulsivity_SC4 NegEmo_SC6 SN_SC7 CTC_SC7 OTC_SC7 if chris_samp
```

Source	SS	df	MS
Model	845.885156	36	23.4968099
Residual	785.462859	949	.827674246
Total	1631.34802	985	1.65619088

Number of obs	=	986
F(36, 949)	=	28.39
Prob > F	=	0.0000
R-squared	=	0.5185
Adj R-squared	=	0.5003
Root MSE	=	.90977

DV_Compliance_SC7	Coef.	Std. Err.	t	P>|t|	[95% Conf.	Interval]
Age	.0032928	.0024946	1.32	0.187	-.0016028	.0081883
1.Gender_Female	.1091297	.0624723	1.75	0.081	-.0134701	.2317294
1.Minority	.0399118	.0656582	0.61	0.543	-.0889402	.1687639
Education	.018884	.0202602	0.93	0.352	-.020876	.058644
1.Employed	.0798883	.0676192	1.18	0.238	-.0528121	.2125888
1.Corona_care	-.1060859	.1056806	-1.00	0.316	-.3134805	.1013087
1.Insurance_Public	.1877856	.0974988	1.93	0.054	-.0035526	.3791237
1.Insurance_Private	.2415216	.0911646	2.65	0.008	.062614	.4204291
SES_before	-.0258673	.0164204	-1.58	0.116	-.0580918	.0063573
SES_change	-.0220101	.0202704	-1.09	0.278	-.06179	.0177698
2.Health_self	.0236853	.0723455	0.33	0.743	-.1182903	.1656609
2.Health_other	-.0479964	.0670192	-0.72	0.474	-.1795193	.0835265
1.Conservative_01	.0587501	.0697964	0.84	0.400	-.078223	.1957232
1.Conservative_other	-.0285331	.1048323	-0.27	0.786	-.2342631	.1771969
1.GeoCensus_d1	-.1705297	.0943711	-1.81	0.071	-.3557298	.0146704
1.GeoCensus_d2	-.1712955	.0806869	-2.12	0.034	-.3296408	-.0129501
1.GeoCensus_d3	-.0661839	.0972247	-0.68	0.496	-.2569842	.1246164
Current_measures						
Yes	.1989929	.083837	2.37	0.018	.0344655	.3635203
Measures_clear	-.00844	.0202497	-0.42	0.677	-.0481794	.0312995
MA_Perc_Threat_SC3	.1397056	.0286239	4.88	0.000	.0835321	.195879
Costs_SC5	.0091335	.0210452	0.43	0.664	-.032167	.050434
Deterr_SD_Likely_SC2	.0048211	.0213024	0.23	0.821	-.0369841	.0466263
Deterr_SD_Severe	-.0064567	.0185502	-0.35	0.728	-.0428609	.0299475
MA_MoralBelief	.2447013	.0326845	7.49	0.000	.180559	.3088435

MA_Authority_SC2	.0003729	.0199582	0.02	0.985	-.0387944	.0395403
NOO_SC3	.0007294	.0411667	0.02	0.986	-.0800588	.0815176
NNOO_SC3	.024595	.0371092	0.66	0.508	-.0482306	.0974206
OOL_SC12	.0161155	.0239798	0.67	0.502	-.030944	.063175
PJE_SC4	.0097119	.0203033	0.48	0.633	-.0301327	.0495564
Trust_Science_SC4	.0373747	.0383934	0.97	0.331	-.037971	.1127204
Trust_in_media	.0012014	.027483	0.04	0.965	-.052733	.0551358
Impulsivity_SC4	-.0628616	.0332365	-1.89	0.059	-.1280873	.002364
NegEmo_SC6	.0410751	.0214859	1.91	0.056	-.0010903	.0832405
SN_SC7	.0116405	.0253257	0.46	0.646	-.0380603	.0613413
CTC_SC7	.5054916	.0388347	13.02	0.000	.4292798	.5817034
OTC_SC7	-.0330591	.0179433	-1.84	0.066	-.0682723	.0021541
_cons	-.1680937	.3331737	-0.50	0.614	-.8219361	.4857486

```
229 . estimates store model_7

230 .
231 . *7.a.3 Check hettest: Run this right after your regression to apply the Breusch-Pagan / Cook-Weisberg test for heter
232 . *if significant, then you need to run the regression with vce(ro) at the end
233 . estat hettest

    Breusch-Pagan / Cook-Weisberg test for heteroskedasticity
             Ho: Constant variance
             Variables: fitted values of DV_Compliance_SC7

             chi2(1)      =   145.02
             Prob > chi2  =   0.0000

234 .
235 . *7.a.4. check vif, to check for for multicollinearity (VIFs >10 are problematic)
236 . vif
```

Variable	VIF	1/VIF
Age	1.33	0.750565
1.Gender_F~e	1.15	0.866626
1.Minority	1.22	0.822057
Education	1.24	0.809273
1.Employed	1.26	0.796780
1.Corona_c~e	1.21	0.824731
1.Insuranc~c	2.25	0.445109
1.Insuran~te	2.41	0.414110
SES_before	1.42	0.705022
SES_change	1.24	0.807540
2.Health_s~f	1.36	0.732820
2.Health_o~r	1.32	0.755967
1.Conserv~01	1.43	0.700326
1.Conserva~r	1.26	0.796081
1.GeoCensu~1	1.68	0.596392
1.GeoCensu~2	1.90	0.527635
1.GeoCensu~3	1.61	0.622363
1.Current_~s	1.19	0.840923
Measures_c~r	1.47	0.679530
MA_Perc_Th~3	2.36	0.423674
Costs_SC5	1.45	0.689627
Deterr_SD_~2	1.71	0.583109
Deterr_SD_~e	1.22	0.817774
MA_MoralBe~f	2.29	0.436282
MA_Authori~2	1.60	0.624930
NOO_SC3	1.69	0.592527
NNOO_SC3	1.71	0.586275
OOL_SC12	1.54	0.648946
PJE_SC4	1.38	0.725868
Trust_Scie~4	1.73	0.577094
Trust_in_m~a	1.51	0.661327
Impulsivi~C4	1.70	0.586978
NegEmo_SC6	1.42	0.704821
SN_SC7	1.50	0.668096
CTC_SC7	1.60	0.624571
OTC_SC7	1.18	0.849960
Mean VIF	1.54	

237 .
238 . *7.a.5. Effect size
239 . estat esize

Effect sizes for linear models

Source	Eta-Squared	df	[95% Conf.	Interval]
Model	.5185191	36	.459445	.5377648
Age	.0018326	1	.	.0111891
Gender_Female	.0032052	1	.	.0142511
Minority	.0003892	1	.	.0068157
Education	.0009146	1	.	.0087126
Employed	.0014687	1	.	.0102721
Corona_care	.0010607	1	.	.0091499
Insurance_Public	.0038937	1	.	.015642
Insurance_Private	.0073416	1	.000482	.0218788
SES_before	.0026081	1	.	.0129779
SES_change	.0012408	1	.	.0096607
Health_self	.0001129	1	.	.0050472
Health_other	.0005402	1	.	.0074413
Conservative_01	.000746	1	.	.0081738
Conservative_other	.0000781	1	.	.0046108
GeoCensus_d1	.003429	1	.	.0147112
GeoCensus_d2	.0047267	1	.	.0172419
GeoCensus_d3	.0004881	1	.	.0072367
Current_measures	.0059016	1	.0001293	.0193847
Measures_clear	.000183	1	.	.0056628
MA_Perc_Threat_SC3	.0244871	1	.0088092	.0470422
Costs_SC5	.0001984	1	.	.005773
Deterr_SD_Likely_SC2	.000054	1	.	.0041932
Deterr_SD_Severe	.0001276	1	.	.0051976
MA_MoralBelief	.05577	1	.0307864	.086234
MA_Authority_SC2	3.68e-07	1	.	.
NOO_SC3	3.31e-07	1	.	.
NNOO_SC3	.0004627	1	.	.0071331
OOL_SC12	.0004757	1	.	.0071866
PJE_SC4	.000241	1	.	.0060489
Trust_Science_SC4	.0009976	1	.	.0089638
Trust_in_media	2.01e-06	1	.	.0006991
Impulsivity_SC4	.0037553	1	.	.0153679
NegEmo_SC6	.0038363	1	.	.0155286
SN_SC7	.0002226	1	.	.0059338
CTC_SC7	.1514885	1	.112287	.1925419
OTC_SC7	.0035642	1	.	.0149852

Note: Eta-Squared values for individual model terms are partial.

240 .
241 . *7.a.6 Regression with vce(ro)
242 . reg DV_Compliance_SC7 Age i.Gender_Female i.Minority Education i.Employed i.Corona_care i.Insurance_Public i.Insura
> vative_other i.GeoCensus_d1 i.GeoCensus_d2 i.GeoCensus_d3 i.Current_measures Measures_clear MA_Perc_Threat_SC3 Costs
> C3 OOL_SC12 PJE_SC4 Trust_Science_SC4 Trust_in_media Impulsivity_SC4 NegEmo_SC6 SN_SC7 CTC_SC7 OTC_SC7 if chris_samp

Linear regression

Number of obs	=	986
F(36, 949)	=	25.69
Prob > F	=	0.0000
R-squared	=	0.5185
Root MSE	=	.90977

DV_Compliance_SC7	Coef.	Robust Std. Err.	t	P>|t|	[95% Conf.	Interval]
Age	.0032928	.0024591	1.34	0.181	-.0015331	.0081186
1.Gender_Female	.1091297	.0621393	1.76	0.079	-.0128167	.2310761
1.Minority	.0399118	.0660955	0.60	0.546	-.0897985	.1696221
Education	.018884	.0193176	0.98	0.329	-.0190261	.0567942
1.Employed	.0798883	.0685647	1.17	0.244	-.0546677	.2144443
1.Corona_care	-.1060859	.1163515	-0.91	0.362	-.3344218	.12225
1.Insurance_Public	.1877856	.1114757	1.68	0.092	-.0309818	.4065529
1.Insurance_Private	.2415216	.1019339	2.37	0.018	.0414797	.4415634

SES_before	-.0258673	.0173226	-1.49	0.136	-.0598623	.0081278
SES_change	-.0220101	.0220877	-1.00	0.319	-.0653565	.0213363
2.Health_self	.0236853	.0670976	0.35	0.724	-.1079916	.1553622
2.Health_other	-.0479964	.0667251	-0.72	0.472	-.1789423	.0829495
1.Conservative_01	.0587501	.0685596	0.86	0.392	-.0757959	.1932962
1.Conservative_other	-.0285331	.1134344	-0.25	0.801	-.2511443	.1940782
1.GeoCensus_d1	-.1705297	.096699	-1.76	0.078	-.3602982	.0192388
1.GeoCensus_d2	-.1712955	.0771437	-2.22	0.027	-.3226875	-.0199035
1.GeoCensus_d3	-.0661839	.0834762	-0.79	0.428	-.2300032	.0976353
Current_measures						
Yes	.1989929	.0954935	2.08	0.037	.0115901	.3863956
Measures_clear	-.00844	.0205267	-0.41	0.681	-.0487229	.031843
MA_Perc_Threat_SC3	.1397056	.0337632	4.14	0.000	.0734465	.2059647
Costs_SC5	.0091335	.0207203	0.44	0.659	-.0315294	.0497964
Deterr_SD_Likely_SC2	.0048211	.0204883	0.24	0.814	-.0353865	.0450287
Deterr_SD_Severe	-.0064567	.0187357	-0.34	0.730	-.0432248	.0303114
MA_MoralBelief	.2447013	.0462736	5.29	0.000	.1538908	.3355117
MA_Authority_SC2	.0003729	.0196142	0.02	0.985	-.0381193	.0388652
NOO_SC3	.0007294	.0419576	0.02	0.986	-.081611	.0830697
NNOO_SC3	.024595	.0388826	0.63	0.527	-.0517108	.1009007
OOL_SC12	.0161155	.0238057	0.68	0.499	-.0306023	.0628333
PJE_SC4	.0097119	.0197999	0.49	0.624	-.0291448	.0485685
Trust_Science_SC4	.0373747	.0415407	0.90	0.369	-.0441476	.118897
Trust_in_media	.0012014	.0263927	0.05	0.964	-.0505933	.0529961
Impulsivity_SC4	-.0628616	.0339454	-1.85	0.064	-.1294783	.003755
NegEmo_SC6	.0410751	.0215557	1.91	0.057	-.0012273	.0833774
SN_SC7	.0116405	.0245527	0.47	0.636	-.0365433	.0598244
CTC_SC7	.5054916	.0526998	9.59	0.000	.40207	.6089132
OTC_SC7	-.0330591	.0167831	-1.97	0.049	-.0659954	-.0001229
_cons	-.1680937	.3440883	-0.49	0.625	-.8433557	.5071682

```
243 .
244 .
245 .
246 . *****************
247 .
248 . *8. Compare Rsq models
249 .
250 . *8.a.1. model 1 vs model 2
251 . lrtest model_1 model_2

    Likelihood-ratio test                             LR chi2(2)  =     96.54
    (Assumption: model_1 nested in model_2)           Prob > chi2 =    0.0000

252 .
253 . *8.a.2. model 2 vs model 3
254 . lrtest model_2 model_3

    Likelihood-ratio test                             LR chi2(4)  =    230.55
    (Assumption: model_2 nested in model_3)           Prob > chi2 =    0.0000

255 .
256 . *8.a.3. model 3 vs model 4
257 . lrtest model_3 model_4

    Likelihood-ratio test                             LR chi2(6)  =    115.15
    (Assumption: model_3 nested in model_4)           Prob > chi2 =    0.0000

258 .
259 . *8.a.4. model 4 vs model 5
```

```
260 . lrtest model_4 model_5

    Likelihood-ratio test                                 LR chi2(4)  =     12.40
    (Assumption: model_4 nested in model_5)               Prob > chi2 =    0.0146

261 .
262 . *8.a.5. model 5 vs model 6
263 . lrtest model_5 model_6

    Likelihood-ratio test                                 LR chi2(1)  =     25.31
    (Assumption: model_5 nested in model_6)               Prob > chi2 =    0.0000

264 .
265 . *8.a.6. model 6 vs model 7
266 . lrtest model_6 model_7

    Likelihood-ratio test                                 LR chi2(2)  =    164.27
    (Assumption: model_6 nested in model_7)               Prob > chi2 =    0.0000

267 .
268 .
269 . ****************
270 .
271 . log close
          name:  <unnamed>
           log:  C:\Users\creinde\OneDrive - UvA\RESEARCH\2020\20 03 Coronavirus-measures compliance survey\Data\US\NWO US
      log type:  smcl
     closed on:  17 Jun 2021, 22:51:55
```
