# Supplementary material for: Social distancing in America: Understanding long-term adherence to COVID-19 mitigation recommendations
Source: PLoS One. 2021 Sep 24;16(9):e0257945. doi: 10.1371/journal.pone.0257945 (PMC8462713; doi:10.1371/journal.pone.0257945)
Supplement: S3 Output — (PDF) [file pone.0257945.s011.pdf]

```
  ___  ____  ____  ____  ____(R)
 /__    /   ____/   /   ____/
___/   /   /___/   /   /___/
  Statistics/Data Analysis
```

```
      name:  <unnamed>
       log:  C:\Users\creinde\OneDrive - UvA\RESEARCH\2020\20 03 Coronavirus-measures compliance survey\Data\US\NWO US
  log type:  smcl
 opened on:  17 Jun 2021, 22:52:58

. use "C:\Users\creinde\OneDrive - UvA\RESEARCH\2020\20 03 Coronavirus-measures compliance survey\Data\US\NWO US Summe

.
.
.
. ************************************************************************************
. ************************************************************************************
. *A. SELECTION CRITERION:
.
. * - Only if provided consent
. * - No missing data
. * - Both checks correct
. * - Nonbinary gender excluded (insufficient number)
.
. gen chris_sample_reqs = 1 if Consent == 1 & N_Missing == 0 & NChecksRight == 2 & Gender < 3
(837 missing values generated)

.
. *B. SELECTION CRITERION:
.
. * - Only if provided consent
. * - Both checks correct
.
. *gen chris_sample_reqs = 1 if Consent == 1 & NChecksRight == 2
.
. *C. SELECTION CRITERION:
.
. * - Only if provided consent
. * - No missing data
.
. *gen chris_sample_reqs = 1 if Consent == 1 & N_Missing == 0
.
.
. tab chris_sample_reqs
```

| chris_sample_reqs | Freq. | Percent | Cum. |
|---|---|---|---|
| 1 | **921** | **100.00** | **100.00** |
| Total | **921** | **100.00** | |

```
.
.
. *generate insurance dummies
.
. gen Insurance_Public = 0

. replace Insurance_Public = 1 if (Insurance == 1)
(470 real changes made)

.
. gen Insurance_Private = 0

. replace Insurance_Private = 1 if (Insurance == 2)
(734 real changes made)
```

```
41 .
42 .
43 . *generate Geographic region dummies
44 .
45 . gen GeoCensus = 0

46 . replace GeoCensus = 1 if (Province == 8|Province == 22|Province == 24|Province == 32|Province == 44|Province == 50|P
   (299 real changes made)

47 . replace GeoCensus = 2 if (Province == 16|Province == 17|Province == 25|Province == 39|Province == 55|Province == 18|
   > 6)
   (317 real changes made)

48 . replace GeoCensus = 3 if (Province == 9|Province == 11|Province == 12|Province == 23|Province == 36|Province == 45|P
   > rovince == 47|Province == 5|Province == 21|Province == 40|Province == 48)
   (671 real changes made)

49 . replace GeoCensus = 4 if (Province == 4|Province == 7|Province == 15|Province == 29|Province == 31|Province == 34|Pr
   > vince == 53)
   (266 real changes made)

50 .
51 . gen GeoCensus_d1 = 0

52 . gen GeoCensus_d2 = 0

53 . gen GeoCensus_d3 = 0

54 .
55 . replace GeoCensus_d1 = 1 if (GeoCensus == 2)
   (317 real changes made)

56 . replace GeoCensus_d2 = 1 if (GeoCensus == 3)
   (671 real changes made)

57 . replace GeoCensus_d3 = 1 if (GeoCensus == 4)
   (266 real changes made)

58 .
59 .
60 . ***********************************************************************************
61 . ***********************************************************************************
62 . ***********************************************************************************
63 . ***********************************************************************************
64 .
65 . *HIERARCHICAL MODEL
66 .
67 . ***********************************************************************************
68 . ***********************************************************************************
69 . ***********************************************************************************
70 . ***********************************************************************************
71 .
72 . *1. Step 1: covariates only
73 .
74 . *1.a.1 Descriptive Statistics
75 . sum DV_Compliance_SC7  Age i.Gender_Female i.Minority Education i.Employed i.Corona_care i.Insurance_Public i.Insura
   > vative_other i.GeoCensus_d1 i.GeoCensus_d2 i.GeoCensus_d3 if chris_sample_reqs == 1
```

| Variable | Obs | Mean | Std. Dev. | Min | Max |
|---|---|---|---|---|---|
| DV_Complia~7 | 921 | 5.76206 | 1.391929 | 1 | 7 |
| Age | 921 | 40.17155 | 12.8724 | 17 | 72 |
| Gender_Fem~e | | | | | |
| 0 | 921 | .4733985 | .4995631 | 0 | 1 |
| 1 | 921 | .5266015 | .4995631 | 0 | 1 |
| Minority | | | | | |
| 0 | 921 | .6666667 | .4716606 | 0 | 1 |
| 1 | 921 | .3333333 | .4716606 | 0 | 1 |
| Education | 921 | 3.756786 | 1.53705 | 1 | 8 |

| | | | | | |
|---|---|---|---|---|---|
| Employed | | | | | |
| 0 | 921 | .3821933 | .4861874 | 0 | 1 |
| 1 | 921 | .6178067 | .4861874 | 0 | 1 |
| Corona_care | | | | | |
| 0 | 921 | .9055375 | .2926301 | 0 | 1 |
| 1 | 921 | .0944625 | .2926301 | 0 | 1 |
| Insurance_~c | | | | | |
| 0 | 921 | .6612378 | .473546 | 0 | 1 |
| 1 | 921 | .3387622 | .473546 | 0 | 1 |
| Insurance_~e | | | | | |
| 0 | 921 | .4744843 | .4996198 | 0 | 1 |
| 1 | 921 | .5255157 | .4996198 | 0 | 1 |
| SES_before | 921 | 5.856678 | 2.096861 | 1 | 10 |
| SES_change | 921 | -.2290988 | 1.700595 | -9 | 8 |
| Health_self | | | | | |
| 1 | 921 | .6210641 | .4853857 | 0 | 1 |
| 2 | 921 | .3789359 | .4853857 | 0 | 1 |
| Health_other | | | | | |
| 1 | 921 | .3778502 | .4851133 | 0 | 1 |
| 2 | 921 | .6221498 | .4851133 | 0 | 1 |
| Conservat~01 | | | | | |
| 0 | 921 | .5439739 | .4983332 | 0 | 1 |
| 1 | 921 | .4560261 | .4983332 | 0 | 1 |
| Conservati~r | | | | | |
| 0 | 921 | .8718784 | .3344068 | 0 | 1 |
| 1 | 921 | .1281216 | .3344068 | 0 | 1 |
| GeoCensus_d1 | | | | | |
| 0 | 921 | .7871878 | .4095183 | 0 | 1 |
| 1 | 921 | .2128122 | .4095183 | 0 | 1 |
| GeoCensus_d2 | | | | | |
| 0 | 921 | .5852334 | .4929494 | 0 | 1 |
| 1 | 921 | .4147666 | .4929494 | 0 | 1 |
| GeoCensus_d3 | | | | | |
| 0 | 921 | .8327904 | .3733656 | 0 | 1 |
| 1 | 921 | .1672096 | .3733656 | 0 | 1 |

```
76 .
77 . *1.a.2 Regression
78 . reg DV_Compliance_SC7  Age i.Gender_Female i.Minority Education i.Employed i.Corona_care i.Insurance_Public i.Insura
   > vative_other i.GeoCensus_d1 i.GeoCensus_d2 i.GeoCensus_d3 if chris_sample_reqs == 1
```

| Source | SS | df | MS |
|---|---|---|---|
| Model | 159.545687 | 17 | 9.38504044 |
| Residual | 1622.92368 | 903 | 1.79725767 |
| Total | 1782.46937 | 920 | 1.9374667 |

| | | |
|---|---|---|
| Number of obs | = | 921 |
| F(17, 903) | = | 5.22 |
| Prob > F | = | 0.0000 |
| R-squared | = | 0.0895 |
| Adj R-squared | = | 0.0724 |
| Root MSE | = | 1.3406 |

| DV_Compliance_SC7 | Coef. | Std. Err. | t | P>\|t\| | [95% Conf. | Interval] |
|---|---|---|---|---|---|---|
| Age | .0114124 | .0036215 | 3.15 | 0.002 | .004305 | .0185199 |
| 1.Gender_Female | .2634238 | .0919597 | 2.86 | 0.004 | .0829442 | .4439034 |
| 1.Minority | .1625946 | .0964824 | 1.69 | 0.092 | -.0267613 | .3519504 |
| Education | .1081568 | .0328651 | 3.29 | 0.001 | .043656 | .1726576 |
| 1.Employed | -.0118966 | .1054161 | -0.11 | 0.910 | -.2187856 | .1949925 |
| 1.Corona_care | -.1804984 | .1622678 | -1.11 | 0.266 | -.4989643 | .1379676 |
| 1.Insurance_Public | .0513097 | .1448771 | 0.35 | 0.723 | -.2330253 | .3356446 |
| 1.Insurance_Private | -.0172581 | .1429419 | -0.12 | 0.904 | -.2977951 | .263279 |
| SES_before | .0447848 | .0235449 | 1.90 | 0.057 | -.0014244 | .090994 |
| SES_change | .0028123 | .0276285 | 0.10 | 0.919 | -.0514113 | .057036 |
| 2.Health_self | .3109036 | .1092228 | 2.85 | 0.005 | .0965436 | .5252637 |
| 2.Health_other | .0405762 | .1071251 | 0.38 | 0.705 | -.1696669 | .2508194 |
| 1.Conservative_01 | -.3113392 | .0972281 | -3.20 | 0.001 | -.5021585 | -.12052 |
| 1.Conservative_other | -.1551149 | .1459877 | -1.06 | 0.288 | -.4416295 | .1313998 |
| 1.GeoCensus_d1 | -.2209107 | .1380567 | -1.60 | 0.110 | -.4918599 | .0500386 |
| 1.GeoCensus_d2 | -.0589445 | .1217732 | -0.48 | 0.628 | -.2979358 | .1800469 |
| 1.GeoCensus_d3 | .184477 | .1476619 | 1.25 | 0.212 | -.1053234 | .4742774 |
| _cons | 4.518217 | .271447 | 16.64 | 0.000 | 3.985476 | 5.050957 |

```
79 . estimates store model_1

80 .
81 . *1.a.3 Check hettest: Run this right after your regression to apply the Breusch-Pagan / Cook-Weisberg test for heter
82 . *if significant, then you need to run the regression with vce(ro) at the end
83 . estat hettest

   Breusch-Pagan / Cook-Weisberg test for heteroskedasticity
            Ho: Constant variance
            Variables: fitted values of DV_Compliance_SC7

            chi2(1)      =    59.29
            Prob > chi2  =   0.0000

84 .
85 . *1.a.4. check vif, to check for for multicollinearity (VIFs >10 are problematic)
86 . vif
```

| Variable | VIF | 1/VIF |
|---|---|---|
| Age | 1.11 | 0.898951 |
| 1.Gender_F~e | 1.08 | 0.925650 |
| 1.Minority | 1.06 | 0.943337 |
| Education | 1.31 | 0.765557 |
| 1.Employed | 1.34 | 0.743706 |
| 1.Corona_c~e | 1.15 | 0.866403 |
| 1.Insuranc~c | 2.41 | 0.415048 |
| 1.Insuran~te | 2.61 | 0.383022 |
| SES_before | 1.25 | 0.801471 |
| SES_change | 1.13 | 0.884922 |
| 2.Health_s~f | 1.44 | 0.695060 |
| 2.Health_o~r | 1.38 | 0.723359 |
| 1.Conserv~01 | 1.20 | 0.832146 |
| 1.Conserva~r | 1.22 | 0.819672 |
| 1.GeoCensu~1 | 1.64 | 0.611169 |
| 1.GeoCensu~2 | 1.84 | 0.542144 |
| 1.GeoCensu~3 | 1.56 | 0.642713 |
| Mean VIF | 1.46 | |

```
87 .
88 . *1.a.5. Effect size
89 . estat esize
```

Effect sizes for linear models

| Source | Eta-Squared | df | [95% Conf. | Interval] |
|---|---|---|---|---|
| Model | .0895082 | 17 | .0423336 | .1092884 |
| Age | .010878 | 1 | .0015492 | .028106 |
| Gender_Female | .0090053 | 1 | .00089 | .0251115 |
| Minority | .0031352 | 1 | . | .0144782 |
| Education | .0118515 | 1 | .0019337 | .0296197 |
| Employed | .0000141 | 1 | . | .0028306 |
| Corona_care | .0013684 | 1 | . | .0103231 |
| Insurance_Public | .0001389 | 1 | . | .0055057 |
| Insurance_Private | .0000161 | 1 | . | .0029807 |
| SES_before | .0039906 | 1 | . | .0162259 |
| SES_change | .0000115 | 1 | . | .0026017 |
| Health_self | .0088932 | 1 | .0008543 | .0249283 |
| Health_other | .0001589 | 1 | . | .0056846 |
| Conservative_01 | .0112278 | 1 | .0016843 | .0286528 |
| Conservative_other | .0012487 | 1 | . | .0099931 |
| GeoCensus_d1 | .0028275 | 1 | . | .0138186 |
| GeoCensus_d2 | .0002594 | 1 | . | .006391 |
| GeoCensus_d3 | .0017255 | 1 | . | .0112556 |

Note: Eta-Squared values for individual model terms are partial.

```
90 .
91 . *1.a.6 Regression with vce(ro)
92 . reg DV_Compliance_SC7  Age i.Gender_Female i.Minority Education i.Employed i.Corona_care i.Insurance_Public i.Insura
> vative_other i.GeoCensus_d1 i.GeoCensus_d2 i.GeoCensus_d3 if chris_sample_reqs == 1, vce(ro)
```

Linear regression

| | | |
|---|---|---|
| Number of obs | = | 921 |
| F(17, 903) | = | 5.24 |
| Prob > F | = | 0.0000 |
| R-squared | = | 0.0895 |
| Root MSE | = | 1.3406 |

| DV_Compliance_SC7 | Coef. | Robust Std. Err. | t | P>\|t\| | [95% Conf. | Interval] |
|---|---|---|---|---|---|---|
| Age | .0114124 | .0036676 | 3.11 | 0.002 | .0042144 | .0186104 |
| 1.Gender_Female | .2634238 | .092252 | 2.86 | 0.004 | .0823706 | .444477 |
| 1.Minority | .1625946 | .0944731 | 1.72 | 0.086 | -.0228177 | .3480069 |
| Education | .1081568 | .0319585 | 3.38 | 0.001 | .0454353 | .1708783 |
| 1.Employed | -.0118966 | .1110354 | -0.11 | 0.915 | -.229814 | .2060208 |
| 1.Corona_care | -.1804984 | .1544246 | -1.17 | 0.243 | -.4835712 | .1225745 |
| 1.Insurance_Public | .0513097 | .1515447 | 0.34 | 0.735 | -.2461111 | .3487305 |
| 1.Insurance_Private | -.0172581 | .1486537 | -0.12 | 0.908 | -.309005 | .2744889 |
| SES_before | .0447848 | .0239629 | 1.87 | 0.062 | -.0022448 | .0918143 |
| SES_change | .0028123 | .0289906 | 0.10 | 0.923 | -.0540844 | .0597091 |
| 2.Health_self | .3109036 | .0970575 | 3.20 | 0.001 | .1204191 | .5013882 |
| 2.Health_other | .0405762 | .1059151 | 0.38 | 0.702 | -.1672921 | .2484446 |
| 1.Conservative_01 | -.3113392 | .0939646 | -3.31 | 0.001 | -.4957537 | -.1269247 |
| 1.Conservative_other | -.1551149 | .146802 | -1.06 | 0.291 | -.4432276 | .1329979 |
| 1.GeoCensus_d1 | -.2209107 | .1426265 | -1.55 | 0.122 | -.5008286 | .0590072 |
| 1.GeoCensus_d2 | -.0589445 | .1199386 | -0.49 | 0.623 | -.2943354 | .1764465 |
| 1.GeoCensus_d3 | .184477 | .1326273 | 1.39 | 0.165 | -.0758166 | .4447706 |
| _cons | 4.518217 | .3107949 | 14.54 | 0.000 | 3.908253 | 5.128181 |

```
 93 .
 94 .
 95 . *****************
 96 .
 97 . *2. Step 2: Add practical knowledge and understanding
 98 .
 99 . *2.a.1 Descriptive Statistics
100 . sum DV_Compliance_SC7  Age i.Gender_Female i.Minority Education i.Employed i.Corona_care i.Insurance_Public i.Insura
    > vative_other i.GeoCensus_d1 i.GeoCensus_d2 i.GeoCensus_d3 i.Current_measures Measures_clear if chris_sample_reqs ==
```

| Variable | Obs | Mean | Std. Dev. | Min | Max |
|---|---|---|---|---|---|
| DV_Complia~7 | 921 | 5.76206 | 1.391929 | 1 | 7 |
| Age | 921 | 40.17155 | 12.8724 | 17 | 72 |
| Gender_Fem~e | | | | | |
| 0 | 921 | .4733985 | .4995631 | 0 | 1 |
| 1 | 921 | .5266015 | .4995631 | 0 | 1 |
| Minority | | | | | |
| 0 | 921 | .6666667 | .4716606 | 0 | 1 |
| 1 | 921 | .3333333 | .4716606 | 0 | 1 |
| Education | 921 | 3.756786 | 1.53705 | 1 | 8 |
| Employed | | | | | |
| 0 | 921 | .3821933 | .4861874 | 0 | 1 |
| 1 | 921 | .6178067 | .4861874 | 0 | 1 |
| Corona_care | | | | | |
| 0 | 921 | .9055375 | .2926301 | 0 | 1 |
| 1 | 921 | .0944625 | .2926301 | 0 | 1 |
| Insurance_~c | | | | | |
| 0 | 921 | .6612378 | .473546 | 0 | 1 |
| 1 | 921 | .3387622 | .473546 | 0 | 1 |
| Insurance_~e | | | | | |
| 0 | 921 | .4744843 | .4996198 | 0 | 1 |
| 1 | 921 | .5255157 | .4996198 | 0 | 1 |
| SES_before | 921 | 5.856678 | 2.096861 | 1 | 10 |
| SES_change | 921 | -.2290988 | 1.700595 | -9 | 8 |
| Health_self | | | | | |
| 1 | 921 | .6210641 | .4853857 | 0 | 1 |
| 2 | 921 | .3789359 | .4853857 | 0 | 1 |
| Health_other | | | | | |
| 1 | 921 | .3778502 | .4851133 | 0 | 1 |
| 2 | 921 | .6221498 | .4851133 | 0 | 1 |
| Conservat~01 | | | | | |
| 0 | 921 | .5439739 | .4983332 | 0 | 1 |
| 1 | 921 | .4560261 | .4983332 | 0 | 1 |
| Conservati~r | | | | | |
| 0 | 921 | .8718784 | .3344068 | 0 | 1 |
| 1 | 921 | .1281216 | .3344068 | 0 | 1 |
| GeoCensus_d1 | | | | | |
| 0 | 921 | .7871878 | .4095183 | 0 | 1 |
| 1 | 921 | .2128122 | .4095183 | 0 | 1 |
| GeoCensus_d2 | | | | | |
| 0 | 921 | .5852334 | .4929494 | 0 | 1 |
| 1 | 921 | .4147666 | .4929494 | 0 | 1 |
| GeoCensus_d3 | | | | | |

```
           0 |        921    .8327904    .3733656          0          1
-------------+---------------------------------------------------------
           1 |        921    .1672096    .3733656          0          1
             |
Current_me~s |
           0 |        921    .1368078    .3438311          0          1
         Yes |        921    .8631922    .3438311          0          1
             |
Measures_c~r |        921    5.033659    1.812384          1          7
```

```
101 .
102 . *2.a.2 Regression
103 . reg DV_Compliance_SC7  Age i.Gender_Female i.Minority Education i.Employed i.Corona_care i.Insurance_Public i.Insura
    > vative_other i.GeoCensus_d1 i.GeoCensus_d2 i.GeoCensus_d3 i.Current_measures Measures_clear if chris_sample_reqs ==
```

```
      Source |       SS           df       MS      Number of obs   =       921
-------------+----------------------------------   F(19, 901)      =      7.69
       Model |  248.760316        19  13.0926482   Prob > F        =    0.0000
    Residual |  1533.70905       901   1.7022298   R-squared       =    0.1396
-------------+----------------------------------   Adj R-squared   =    0.1214
       Total |  1782.46937       920   1.9374667   Root MSE        =    1.3047
```

```
------------------------------------------------------------------------------------
   DV_Compliance_SC7 |      Coef.   Std. Err.      t    P>|t|     [95% Conf. Interval]
---------------------+--------------------------------------------------------------
                 Age |   .0096207   .0035359     2.72   0.007     .0026812    .0165602
     1.Gender_Female |   .2405078    .089552     2.69   0.007      .064753    .4162626
          1.Minority |   .0952047   .0943709     1.01   0.313    -.0900078    .2804171
           Education |   .1108338   .0319869     3.46   0.001     .0480563    .1736114
          1.Employed |    .018375    .102829     0.18   0.858    -.1834372    .2201872
       1.Corona_care |  -.1930157   .1579312    -1.22   0.222    -.5029716    .1169401
  1.Insurance_Public |   -.004434   .1412113    -0.03   0.975    -.2815753    .2727072
 1.Insurance_Private |  -.0770336    .139375    -0.55   0.581     -.350571    .1965039
          SES_before |   .0321454   .0229805     1.40   0.162    -.0129561    .0772469
          SES_change |   .0011362   .0269008     0.04   0.966    -.0516592    .0539317
       2.Health_self |   .2634922   .1065313     2.47   0.014     .0544138    .4725705
      2.Health_other |   .0152049   .1043802     0.15   0.884    -.1896518    .2200616
   1.Conservative_01 |  -.2842093   .0947277    -3.00   0.003    -.4701219   -.0982966
1.Conservative_other |  -.0939172   .1423357    -0.66   0.510    -.3732654    .1854309
     1.GeoCensus_d1 |  -.1402172   .1348195    -1.04   0.299    -.4048141    .1243797
     1.GeoCensus_d2 |   -.02409   .1186091    -0.20   0.839    -.2568723    .2086922
     1.GeoCensus_d3 |   .1819387   .1437401     1.27   0.206    -.1001656     .464043
                     |
    Current_measures |
                Yes  |   .4790372   .1302292     3.68   0.000     .2234493    .7346251
      Measures_clear |   .1319466   .0248341     5.31   0.000     .0832072    .1806859
               _cons |   3.625742   .2915235    12.44   0.000     3.053598    4.197886
------------------------------------------------------------------------------------
```

```
104 . estimates store model_2

105 .
106 . *2.a.3 Check hettest: Run this right after your regression to apply the Breusch-Pagan / Cook-Weisberg test for heter
107 . *if significant, then you need to run the regression with vce(ro) at the end
108 . estat hettest

Breusch-Pagan / Cook-Weisberg test for heteroskedasticity
         Ho: Constant variance
         Variables: fitted values of DV_Compliance_SC7

         chi2(1)      =    81.89
         Prob > chi2  =   0.0000
```

```
109 .
110 . *2.a.4. check vif, to check for for multicollinearity (VIFs >10 are problematic)
111 . vif
```

| Variable | VIF | 1/VIF |
|---|---|---|
| Age | 1.12 | 0.893137 |
| 1.Gender_F~e | 1.08 | 0.924483 |
| 1.Minority | 1.07 | 0.933887 |
| Education | 1.31 | 0.765437 |
| 1.Employed | 1.35 | 0.740273 |
| 1.Corona_c~e | 1.15 | 0.866277 |
| 1.Insuranc~c | 2.42 | 0.413778 |
| 1.Insuran~te | 2.62 | 0.381576 |
| SES_before | 1.25 | 0.796842 |
| SES_change | 1.13 | 0.884096 |
| 2.Health_s~f | 1.45 | 0.691993 |
| 2.Health_o~r | 1.39 | 0.721618 |
| 1.Conserv~01 | 1.20 | 0.830303 |
| 1.Conserva~r | 1.22 | 0.816681 |
| 1.GeoCensu~1 | 1.65 | 0.606985 |
| 1.GeoCensu~2 | 1.85 | 0.541240 |
| 1.GeoCensu~3 | 1.56 | 0.642401 |
| 1.Current_~s | 1.08 | 0.922834 |
| Measures_c~r | 1.09 | 0.913344 |
| Mean VIF | 1.42 | |

```
112 .
113 . *2.a.5. Effect size
114 . estat esize
```

Effect sizes for linear models

| Source | Eta-Squared | df | [95% Conf. | Interval] |
|---|---|---|---|---|
| Model | .1395594 | 19 | .0840018 | .1635749 |
| Age | .0081498 | 1 | .0006252 | .0237216 |
| Gender_Female | .0079418 | 1 | .000566 | .0233736 |
| Minority | .0011283 | 1 | . | .0096641 |
| Education | .01315 | 1 | .0024773 | .0316253 |
| Employed | .0000354 | 1 | . | .0038697 |
| Corona_care | .001655 | 1 | . | .0110919 |
| Insurance_Public | 1.09e-06 | 1 | . | 3.77e-06 |
| Insurance_Private | .0003389 | 1 | . | .0068366 |
| SES_before | .002167 | 1 | . | .0123438 |
| SES_change | 1.98e-06 | 1 | . | .0006602 |
| Health_self | .006744 | 1 | .0002565 | .021327 |
| Health_other | .0000236 | 1 | . | .0034075 |
| Conservative_01 | .0098919 | 1 | .0011828 | .0265665 |
| Conservative_other | .000483 | 1 | . | .0074915 |
| GeoCensus_d1 | .0011991 | 1 | . | .0098674 |
| GeoCensus_d2 | .0000458 | 1 | . | .0041634 |
| GeoCensus_d3 | .001775 | 1 | . | .0113949 |
| Current_measures | .0147953 | 1 | .0032281 | .0340828 |
| Measures_clear | .0303793 | 1 | .0121774 | .0555374 |

Note: Eta-Squared values for individual model terms are partial.

```
115 .
```

```
116 . *2.a.6 Regression with vce(ro)
117 . reg DV_Compliance_SC7  Age i.Gender_Female i.Minority Education i.Employed i.Corona_care i.Insurance_Public i.Insura
    > vative_other i.GeoCensus_d1 i.GeoCensus_d2 i.GeoCensus_d3 i.Current_measures Measures_clear if chris_sample_reqs ==

    Linear regression                               Number of obs     =        921
                                                    F(19, 901)        =       6.85
                                                    Prob > F          =     0.0000
                                                    R-squared         =     0.1396
                                                    Root MSE          =     1.3047

    --------------------------------------------------------------------------------
                        |               Robust
       DV_Compliance_SC7 |      Coef.   Std. Err.      t    P>|t|     [95% Conf. Interval]
    --------------------+-----------------------------------------------------------
                    Age |   .0096207   .0035172     2.74   0.006     .0027179    .0165236
        1.Gender_Female |   .2405078   .0894952     2.69   0.007     .0648646    .4161511
             1.Minority |   .0952047   .0939121     1.01   0.311    -.0891074    .2795167
              Education |   .1108338   .0317352     3.49   0.001     .0485503    .1731173
             1.Employed |    .018375   .1082593     0.17   0.865    -.1940947    .2308447
          1.Corona_care |  -.1930157    .156932    -1.23   0.219    -.5010105     .114979
     1.Insurance_Public |   -.004434   .1464344    -0.03   0.976    -.2918263    .2829582
    1.Insurance_Private |  -.0770336   .1432106    -0.54   0.591    -.3580987    .2040315
             SES_before |   .0321454    .023259     1.38   0.167    -.0135027    .0777935
             SES_change |   .0011362   .0284901     0.04   0.968    -.0547785     .057051
          2.Health_self |   .2634922   .0966757     2.73   0.007     .0737565    .4532278
         2.Health_other |   .0152049   .1038979     0.15   0.884    -.1887052    .2191149
      1.Conservative_01 |  -.2842093   .0918009    -3.10   0.002    -.4643778   -.1040407
   1.Conservative_other |  -.0939172    .146215    -0.64   0.521    -.3808789    .1930444
         1.GeoCensus_d1 |  -.1402172   .1371054    -1.02   0.307    -.4093002    .1288658
         1.GeoCensus_d2 |    -.02409   .1165442    -0.21   0.836    -.2528198    .2046397
         1.GeoCensus_d3 |   .1819387   .1293714     1.41   0.160    -.0719658    .4358431
                        |
       Current_measures |
                   Yes  |   .4790372   .1633096     2.93   0.003     .1585258    .7995486
         Measures_clear |   .1319466   .0274719     4.80   0.000     .0780302     .185863
                  _cons |   3.625742   .3412806    10.62   0.000     2.955944    4.295539
    --------------------------------------------------------------------------------

118 .
119 .
120 . *****************
121 .
122 . *3. Step 3: Add costs + benefits
123 .
124 . *3.a.1 Descriptive Statistics
125 . sum DV_Compliance_SC7  Age i.Gender_Female i.Minority Education i.Employed i.Corona_care i.Insurance_Public i.Insura
    > vative_other i.GeoCensus_d1 i.GeoCensus_d2 i.GeoCensus_d3 i.Current_measures Measures_clear MA_Perc_Threat_SC3 Costs

        Variable |        Obs        Mean    Std. Dev.       Min        Max
    -------------+---------------------------------------------------------
    DV_Complia~7 |        921     5.76206    1.391929          1          7
             Age |        921    40.17155     12.8724         17         72
                 |
    Gender_Fem~e |
               0 |        921    .4733985    .4995631          0          1
               1 |        921    .5266015    .4995631          0          1
                 |
        Minority |
               0 |        921    .6666667    .4716606          0          1
    -------------+---------------------------------------------------------
               1 |        921    .3333333    .4716606          0          1
                 |
       Education |        921    3.756786     1.53705          1          8
                 |
        Employed |
               0 |        921    .3821933    .4861874          0          1
               1 |        921    .6178067    .4861874          0          1
                 |
     Corona_care |
               0 |        921    .9055375    .2926301          0          1
    -------------+---------------------------------------------------------
               1 |        921    .0944625    .2926301          0          1
                 |
    Insurance_~c |
```

```
           0 |        921    .6612378     .473546          0          1
           1 |        921    .3387622     .473546          0          1
             |
Insurance_~e |
           0 |        921    .4744843    .4996198          0          1
           1 |        921    .5255157    .4996198          0          1
-------------+--------------------------------------------------------
             |
  SES_before |        921    5.856678    2.096861          1         10
  SES_change |        921   -.2290988    1.700595         -9          8
             |
 Health_self |
           1 |        921    .6210641    .4853857          0          1
           2 |        921    .3789359    .4853857          0          1
             |
Health_other |
           1 |        921    .3778502    .4851133          0          1
-------------+--------------------------------------------------------
           2 |        921    .6221498    .4851133          0          1
             |
Conservat~01 |
           0 |        921    .5439739    .4983332          0          1
           1 |        921    .4560261    .4983332          0          1
             |
Conservati~r |
           0 |        921    .8718784    .3344068          0          1
           1 |        921    .1281216    .3344068          0          1
-------------+--------------------------------------------------------
             |
GeoCensus_d1 |
           0 |        921    .7871878    .4095183          0          1
           1 |        921    .2128122    .4095183          0          1
             |
GeoCensus_d2 |
           0 |        921    .5852334    .4929494          0          1
           1 |        921    .4147666    .4929494          0          1
             |
GeoCensus_d3 |
           0 |        921    .8327904    .3733656          0          1
-------------+--------------------------------------------------------
           1 |        921    .1672096    .3733656          0          1
             |
Current_me~s |
           0 |        921    .1368078    .3438311          0          1
         Yes |        921    .8631922    .3438311          0          1
             |
Measures_c~r |        921    5.033659    1.812384          1          7
MA_Perc_Th~3 |        921    5.740861    1.486305          1          7
-------------+--------------------------------------------------------
    Costs_SC5 |        921    4.148534    1.638717          1          7
Deterr_SD_~2 |        921    3.235613    1.741087          1          7
Deterr_SD_~e |        921    3.890337    1.729515          1          6

126 .
127 . *3.a.2 Regression
128 . reg DV_Compliance_SC7  Age i.Gender_Female i.Minority Education i.Employed i.Corona_care i.Insurance_Public i.Insura
    > vative_other i.GeoCensus_d1 i.GeoCensus_d2 i.GeoCensus_d3 i.Current_measures Measures_clear MA_Perc_Threat_SC3 Costs

      Source |       SS           df       MS      Number of obs   =       921
-------------+----------------------------------   F(23, 897)      =     17.70
       Model |  556.525175        23  24.1967468   Prob > F        =    0.0000
    Residual |  1225.94419       897  1.36671593   R-squared       =    0.3122
-------------+----------------------------------   Adj R-squared   =    0.2946
       Total |  1782.46937       920   1.9374667   Root MSE        =    1.1691
```

| DV_Compliance_SC7 | Coef. | Std. Err. | t | P>\|t\| | [95% Conf. | Interval] |
|---|---|---|---|---|---|---|
| Age | .0093907 | .0031956 | 2.94 | 0.003 | .0031191 | .0156624 |
| 1.Gender_Female | .1880656 | .0805433 | 2.33 | 0.020 | .0299903 | .3461409 |
| 1.Minority | -.0336013 | .085465 | -0.39 | 0.694 | -.201336 | .1341335 |
| Education | .0728063 | .028832 | 2.53 | 0.012 | .0162203 | .1293922 |
| 1.Employed | .0155931 | .0921975 | 0.17 | 0.866 | -.1653549 | .196541 |
| 1.Corona_care | -.2484824 | .144572 | -1.72 | 0.086 | -.5322211 | .0352563 |
| 1.Insurance_Public | .0784772 | .126835 | 0.62 | 0.536 | -.1704507 | .327405 |
| 1.Insurance_Private | .0632067 | .125935 | 0.50 | 0.616 | -.183955 | .3103683 |
| SES_before | .0471745 | .0209654 | 2.25 | 0.025 | .0060276 | .0883214 |
| SES_change | .0155995 | .0245294 | 0.64 | 0.525 | -.0325422 | .0637411 |
| 2.Health_self | .0223047 | .0974287 | 0.23 | 0.819 | -.16891 | .2135194 |
| 2.Health_other | -.0956695 | .0948938 | -1.01 | 0.314 | -.2819093 | .0905703 |
| 1.Conservative_01 | -.0417557 | .0867 | -0.48 | 0.630 | -.2119142 | .1284028 |
| 1.Conservative_other | .1283317 | .1285155 | 1.00 | 0.318 | -.1238943 | .3805578 |
| 1.GeoCensus_d1 | -.0047522 | .1212152 | -0.04 | 0.969 | -.2426506 | .2331462 |
| 1.GeoCensus_d2 | .0325588 | .1065627 | 0.31 | 0.760 | -.1765824 | .2417001 |
| 1.GeoCensus_d3 | .2310005 | .1290839 | 1.79 | 0.074 | -.022341 | .4843421 |
| Current_measures | | | | | | |
| Yes | .3422393 | .1173869 | 2.92 | 0.004 | .1118544 | .5726241 |
| Measures_clear | .0681767 | .0228659 | 2.98 | 0.003 | .0232998 | .1130536 |
| MA_Perc_Threat_SC3 | .4244787 | .0299034 | 14.20 | 0.000 | .36579 | .4831674 |
| Costs_SC5 | .0486656 | .0272013 | 1.79 | 0.074 | -.0047199 | .1020511 |
| Deterr_SD_Likely_SC2 | -.0132962 | .0264578 | -0.50 | 0.615 | -.0652226 | .0386301 |
| Deterr_SD_Severe | .0353471 | .0241041 | 1.47 | 0.143 | -.0119599 | .0826542 |
| _cons | 1.335491 | .3363094 | 3.97 | 0.000 | .6754457 | 1.995536 |

```
129 . estimates store model_3

130 .
131 . *3.a.3 Check hettest: Run this right after your regression to apply the Breusch-Pagan / Cook-Weisberg test for heter
132 . *if significant, then you need to run the regression with vce(ro) at the end
133 . estat hettest

    Breusch-Pagan / Cook-Weisberg test for heteroskedasticity
             Ho: Constant variance
             Variables: fitted values of DV_Compliance_SC7

             chi2(1)      =    71.95
             Prob > chi2  =   0.0000

134 .
135 . *3.a.4. check vif, to check for for multicollinearity (VIFs >10 are problematic)
136 . vif
```

| Variable | VIF | 1/VIF |
|---|---|---|
| Age | 1.14 | 0.877956 |
| 1.Gender_F~e | 1.09 | 0.917594 |
| 1.Minority | 1.09 | 0.914227 |
| Education | 1.32 | 0.756426 |
| 1.Employed | 1.35 | 0.739341 |
| 1.Corona_c~e | 1.20 | 0.830012 |
| 1.Insuranc~c | 2.43 | 0.411802 |
| 1.Insuran~te | 2.66 | 0.375248 |
| SES_before | 1.30 | 0.768681 |
| SES_change | 1.17 | 0.853720 |
| 2.Health_s~f | 1.51 | 0.664267 |
| 2.Health_o~r | 1.43 | 0.701016 |
| 1.Conserv~01 | 1.26 | 0.795816 |
| 1.Conserva~r | 1.24 | 0.804321 |
| 1.GeoCensu~1 | 1.66 | 0.602878 |
| 1.GeoCensu~2 | 1.86 | 0.538363 |
| 1.GeoCensu~3 | 1.56 | 0.639555 |
| 1.Current_~s | 1.10 | 0.911930 |
| Measures_c~r | 1.16 | 0.864994 |
| MA_Perc_Th~3 | 1.33 | 0.752029 |
| Costs_SC5 | 1.34 | 0.747661 |
| Deterr_SD_~2 | 1.43 | 0.700073 |
| Deterr_SD_~e | 1.17 | 0.854790 |

| | |
|---|---|
| Mean VIF | 1.43 |

```
137 .
138 . *3.a.5. Effect size
139 . estat esize
```

Effect sizes for linear models

| Source | Eta-Squared | df | [95% Conf. | Interval] |
|---|---|---|---|---|
| Model | .3122215 | 23 | .2476333 | .3409124 |
| Age | .0095356 | 1 | .0010506 | .0260389 |
| Gender_Female | .0060414 | 1 | .0000934 | .0201276 |
| Minority | .0001723 | 1 | . | .0058246 |
| Education | .0070586 | 1 | .0003264 | .0219135 |
| Employed | .0000319 | 1 | . | .0037612 |
| Corona_care | .0032825 | 1 | . | .0148391 |
| Insurance_Public | .0004266 | 1 | . | .0072736 |
| Insurance_Private | .0002807 | 1 | . | .0065476 |
| SES_before | .0056127 | 1 | 4.91e-06 | .0193542 |
| SES_change | .0004507 | 1 | . | .0073788 |
| Health_self | .0000584 | 1 | . | .0044617 |
| Health_other | .0011318 | 1 | . | .0097024 |
| Conservative_01 | .0002585 | 1 | . | .0064177 |
| Conservative_other | .0011104 | 1 | . | .0096399 |
| GeoCensus_d1 | 1.71e-06 | 1 | . | .0004974 |
| GeoCensus_d2 | .0001041 | 1 | . | .0051621 |
| GeoCensus_d3 | .0035575 | 1 | . | .0154076 |
| Current_measures | .0093871 | 1 | .0010009 | .0257988 |
| Measures_clear | .0098134 | 1 | .0011455 | .0264859 |
| MA_Perc_Threat_SC3 | .1834308 | 1 | .1405355 | .2272245 |
| Costs_SC5 | .0035557 | 1 | . | .0154039 |
| Deterr_SD_Likely_SC2 | .0002815 | 1 | . | .0065517 |
| Deterr_SD_Severe | .0023916 | 1 | . | .0128965 |

Note: Eta-Squared values for individual model terms are partial.

```
140 .
141 . *3.a.6 Regression with vce(ro)
142 . reg DV_Compliance_SC7  Age i.Gender_Female i.Minority Education i.Employed i.Corona_care i.Insurance_Public i.Insura
    > vative_other i.GeoCensus_d1 i.GeoCensus_d2 i.GeoCensus_d3 i.Current_measures Measures_clear MA_Perc_Threat_SC3 Costs
```

Linear regression

| | | |
|---|---|---|
| Number of obs | = | 921 |
| F(23, 897) | = | 14.57 |
| Prob > F | = | 0.0000 |
| R-squared | = | 0.3122 |
| Root MSE | = | 1.1691 |

| DV_Compliance_SC7 | Coef. | Robust Std. Err. | t | P>\|t\| | [95% Conf. | Interval] |
|---|---|---|---|---|---|---|
| Age | .0093907 | .0032504 | 2.89 | 0.004 | .0030115 | .01577 |
| 1.Gender_Female | .1880656 | .0811472 | 2.32 | 0.021 | .0288051 | .3473261 |
| 1.Minority | -.0336013 | .0893661 | -0.38 | 0.707 | -.2089923 | .1417897 |
| Education | .0728063 | .0287595 | 2.53 | 0.012 | .0163625 | .1292501 |
| 1.Employed | .0155931 | .0972067 | 0.16 | 0.873 | -.1751859 | .206372 |
| 1.Corona_care | -.2484824 | .1556245 | -1.60 | 0.111 | -.553913 | .0569481 |
| 1.Insurance_Public | .0784772 | .1362171 | 0.58 | 0.565 | -.1888643 | .3458186 |
| 1.Insurance_Private | .0632067 | .1358127 | 0.47 | 0.642 | -.203341 | .3297544 |
| SES_before | .0471745 | .0214462 | 2.20 | 0.028 | .0050839 | .0892651 |
| SES_change | .0155995 | .0271489 | 0.57 | 0.566 | -.0376833 | .0688823 |
| 2.Health_self | .0223047 | .0866634 | 0.26 | 0.797 | -.147782 | .1923913 |
| 2.Health_other | -.0956695 | .0925328 | -1.03 | 0.301 | -.2772754 | .0859365 |
| 1.Conservative_01 | -.0417557 | .0831671 | -0.50 | 0.616 | -.2049805 | .121469 |
| 1.Conservative_other | .1283317 | .1370703 | 0.94 | 0.349 | -.1406842 | .3973476 |
| 1.GeoCensus_d1 | -.0047522 | .1232026 | -0.04 | 0.969 | -.2465512 | .2370468 |
| 1.GeoCensus_d2 | .0325588 | .109333 | 0.30 | 0.766 | -.1820194 | .247137 |
| 1.GeoCensus_d3 | .2310005 | .1275132 | 1.81 | 0.070 | -.0192584 | .4812594 |
| Current_measures | | | | | | |

| | | | | | | |
|---|---|---|---|---|---|---|
| Yes | .3422393 | .1401501 | 2.44 | 0.015 | .0671791 | .6172995 |
| Measures_clear | .0681767 | .0239784 | 2.84 | 0.005 | .0211165 | .1152369 |
| MA_Perc_Threat_SC3 | .4244787 | .0369608 | 11.48 | 0.000 | .351939 | .4970183 |
| Costs_SC5 | .0486656 | .0279392 | 1.74 | 0.082 | -.0061682 | .1034994 |
| Deterr_SD_Likely_SC2 | -.0132962 | .0291689 | -0.46 | 0.649 | -.0705434 | .043951 |
| Deterr_SD_Severe | .0353471 | .025047 | 1.41 | 0.159 | -.0138104 | .0845047 |
| _cons | 1.335491 | .3855025 | 3.46 | 0.001 | .5788988 | 2.092083 |

```
143 .
144 .
145 . *****************
146 .
147 . *4. Step 4: Add legitimacy
148 .
149 . *4.a.1 Descriptive Statistics
150 . sum DV_Compliance_SC7  Age i.Gender_Female i.Minority Education i.Employed i.Corona_care i.Insurance_Public i.Insura
    > vative_other i.GeoCensus_d1 i.GeoCensus_d2 i.GeoCensus_d3 i.Current_measures Measures_clear MA_Perc_Threat_SC3 Costs
    > C3 OOL_SC12 PJE_SC4 if chris_sample_reqs == 1
```

| Variable | Obs | Mean | Std. Dev. | Min | Max |
|---|---|---|---|---|---|
| DV_Complia~7 | 921 | 5.76206 | 1.391929 | 1 | 7 |
| Age | 921 | 40.17155 | 12.8724 | 17 | 72 |
| Gender_Fem~e | | | | | |
| 0 | 921 | .4733985 | .4995631 | 0 | 1 |
| 1 | 921 | .5266015 | .4995631 | 0 | 1 |
| Minority | | | | | |
| 0 | 921 | .6666667 | .4716606 | 0 | 1 |
| 1 | 921 | .3333333 | .4716606 | 0 | 1 |
| Education | 921 | 3.756786 | 1.53705 | 1 | 8 |
| Employed | | | | | |
| 0 | 921 | .3821933 | .4861874 | 0 | 1 |
| 1 | 921 | .6178067 | .4861874 | 0 | 1 |
| Corona_care | | | | | |
| 0 | 921 | .9055375 | .2926301 | 0 | 1 |
| 1 | 921 | .0944625 | .2926301 | 0 | 1 |
| Insurance_~c | | | | | |
| 0 | 921 | .6612378 | .473546 | 0 | 1 |
| 1 | 921 | .3387622 | .473546 | 0 | 1 |
| Insurance_~e | | | | | |
| 0 | 921 | .4744843 | .4996198 | 0 | 1 |
| 1 | 921 | .5255157 | .4996198 | 0 | 1 |
| SES_before | 921 | 5.856678 | 2.096861 | 1 | 10 |
| SES_change | 921 | -.2290988 | 1.700595 | -9 | 8 |
| Health_self | | | | | |
| 1 | 921 | .6210641 | .4853857 | 0 | 1 |
| 2 | 921 | .3789359 | .4853857 | 0 | 1 |
| Health_other | | | | | |
| 1 | 921 | .3778502 | .4851133 | 0 | 1 |
| 2 | 921 | .6221498 | .4851133 | 0 | 1 |
| Conservat~01 | | | | | |
| 0 | 921 | .5439739 | .4983332 | 0 | 1 |
| 1 | 921 | .4560261 | .4983332 | 0 | 1 |
| Conservati~r | | | | | |
| 0 | 921 | .8718784 | .3344068 | 0 | 1 |
| 1 | 921 | .1281216 | .3344068 | 0 | 1 |

| | | | | | |
|---|---|---|---|---|---|
| GeoCensus_d1 | | | | | |
| 0 | 921 | .7871878 | .4095183 | 0 | 1 |
| 1 | 921 | .2128122 | .4095183 | 0 | 1 |
| GeoCensus_d2 | | | | | |
| 0 | 921 | .5852334 | .4929494 | 0 | 1 |
| 1 | 921 | .4147666 | .4929494 | 0 | 1 |
| GeoCensus_d3 | | | | | |
| 0 | 921 | .8327904 | .3733656 | 0 | 1 |
| 1 | 921 | .1672096 | .3733656 | 0 | 1 |
| Current_me~s | | | | | |
| 0 | 921 | .1368078 | .3438311 | 0 | 1 |
| Yes | 921 | .8631922 | .3438311 | 0 | 1 |
| Measures_c~r | 921 | 5.033659 | 1.812384 | 1 | 7 |
| MA_Perc_Th~3 | 921 | 5.740861 | 1.486305 | 1 | 7 |
| Costs_SC5 | 921 | 4.148534 | 1.638717 | 1 | 7 |
| Deterr_SD_~2 | 921 | 3.235613 | 1.741087 | 1 | 7 |
| Deterr_SD_~e | 921 | 3.890337 | 1.729515 | 1 | 6 |
| MA_MoralBe~f | 921 | 6.150923 | 1.359356 | 1 | 7 |
| MA_Authori~2 | 921 | 3.814332 | 1.945522 | 1 | 7 |
| NOO_SC3 | 921 | 3.899023 | .9339596 | 1 | 5 |
| NNOO_SC3 | 921 | 2.938835 | .9835599 | 1 | 5 |
| OOL_SC12 | 921 | 4.376945 | 1.487538 | 1 | 7 |
| PJE_SC4 | 921 | 5.079262 | 1.64679 | 1 | 7 |

```
151 .
152 . *4.a.2 Regression
153 . reg DV_Compliance_SC7  Age i.Gender_Female i.Minority Education i.Employed i.Corona_care i.Insurance_Public i.Insura
    > vative_other i.GeoCensus_d1 i.GeoCensus_d2 i.GeoCensus_d3 i.Current_measures Measures_clear MA_Perc_Threat_SC3 Costs
    > C3 OOL_SC12 PJE_SC4 if chris_sample_reqs == 1
```

| Source | SS | df | MS | | | |
|---|---|---|---|---|---|---|
| | | | | Number of obs | = | 921 |
| | | | | F(29, 891) | = | 20.22 |
| Model | 707.557423 | 29 | 24.3985318 | Prob > F | = | 0.0000 |
| Residual | 1074.91194 | 891 | 1.20641071 | R-squared | = | 0.3970 |
| | | | | Adj R-squared | = | 0.3773 |
| Total | 1782.46937 | 920 | 1.9374667 | Root MSE | = | 1.0984 |

| DV_Compliance_SC7 | Coef. | Std. Err. | t | P>\|t\| | [95% Conf. | Interval] |
|---|---|---|---|---|---|---|
| Age | .0067611 | .0030767 | 2.20 | 0.028 | .0007226 | .0127995 |
| 1.Gender_Female | .157395 | .0762931 | 2.06 | 0.039 | .0076599 | .3071301 |
| 1.Minority | -.029901 | .0803576 | -0.37 | 0.710 | -.1876132 | .1278112 |
| Education | .0671261 | .0271715 | 2.47 | 0.014 | .0137985 | .1204538 |
| 1.Employed | .0316137 | .0870012 | 0.36 | 0.716 | -.1391375 | .2023648 |
| 1.Corona_care | -.2269601 | .137923 | -1.65 | 0.100 | -.497652 | .0437318 |
| 1.Insurance_Public | .1007447 | .1196259 | 0.84 | 0.400 | -.1340368 | .3355261 |
| 1.Insurance_Private | .0308161 | .1186602 | 0.26 | 0.795 | -.2020699 | .2637021 |
| SES_before | .0339202 | .0204413 | 1.66 | 0.097 | -.0061986 | .0740389 |
| SES_change | .0063316 | .0234207 | 0.27 | 0.787 | -.0396346 | .0522978 |
| 2.Health_self | .0350109 | .0924871 | 0.38 | 0.705 | -.1465071 | .2165289 |
| 2.Health_other | -.1139342 | .0901565 | -1.26 | 0.207 | -.2908781 | .0630096 |
| 1.Conservative_01 | .0384402 | .084271 | 0.46 | 0.648 | -.1269525 | .203833 |
| 1.Conservative_other | .2051169 | .122145 | 1.68 | 0.093 | -.0346086 | .4448424 |
| 1.GeoCensus_d1 | -.0186564 | .1144051 | -0.16 | 0.870 | -.2431914 | .2058785 |
| 1.GeoCensus_d2 | -.0091827 | .1005541 | -0.09 | 0.927 | -.2065331 | .1881677 |
| 1.GeoCensus_d3 | .1564985 | .1218174 | 1.28 | 0.199 | -.0825839 | .3955809 |
| Current_measures | | | | | | |
| Yes | .271433 | .1106396 | 2.45 | 0.014 | .0542884 | .4885776 |
| Measures_clear | .0213603 | .0231207 | 0.92 | 0.356 | -.024017 | .0667376 |
| MA_Perc_Threat_SC3 | .1535816 | .0374958 | 4.10 | 0.000 | .0799912 | .2271721 |
| Costs_SC5 | .0549763 | .026193 | 2.10 | 0.036 | .0035692 | .1063834 |
| Deterr_SD_Likely_SC2 | -.0155043 | .0273572 | -0.57 | 0.571 | -.0691963 | .0381877 |
| Deterr_SD_Severe | .0128621 | .022978 | 0.56 | 0.576 | -.0322351 | .0579593 |
| MA_MoralBelief | .3608382 | .0393285 | 9.17 | 0.000 | .2836509 | .4380255 |

| | | | | | | |
|---|---|---|---|---|---|---|
| MA_Authority_SC2 | -.0060891 | .0245952 | -0.25 | 0.805 | -.0543604 | .0421821 |
| NOO_SC3 | .1936145 | .0510176 | 3.80 | 0.000 | .0934858 | .2937432 |
| NNOO_SC3 | .0644588 | .0441676 | 1.46 | 0.145 | -.022226 | .1511435 |
| OOL_SC12 | .0414625 | .0289468 | 1.43 | 0.152 | -.0153493 | .0982744 |
| PJE_SC4 | -.0198905 | .0263517 | -0.75 | 0.451 | -.0716092 | .0318282 |
| _cons | .2426497 | .366441 | 0.66 | 0.508 | -.4765384 | .9618379 |

```
. estimates store model_4

.
. *4.a.3 Check hettest: Run this right after your regression to apply the Breusch-Pagan / Cook-Weisberg test for heter
. *if significant, then you need to run the regression with vce(ro) at the end
. estat hettest
```

```
Breusch-Pagan / Cook-Weisberg test for heteroskedasticity
         Ho: Constant variance
         Variables: fitted values of DV_Compliance_SC7

         chi2(1)      =    47.14
         Prob > chi2  =   0.0000
```

```
.
. *4.a.4. check vif, to check for for multicollinearity (VIFs >10 are problematic)
. vif
```

| Variable | VIF | 1/VIF |
|---|---|---|
| Age | 1.20 | 0.836008 |
| 1.Gender_F~e | 1.11 | 0.902726 |
| 1.Minority | 1.10 | 0.912839 |
| Education | 1.33 | 0.751802 |
| 1.Employed | 1.36 | 0.732908 |
| 1.Corona_c~e | 1.24 | 0.805000 |
| 1.Insuranc~c | 2.45 | 0.408632 |
| 1.Insuran~te | 2.68 | 0.373094 |
| SES_before | 1.40 | 0.713756 |
| SES_change | 1.21 | 0.826619 |
| 2.Health_s~f | 1.54 | 0.650685 |
| 2.Health_o~r | 1.46 | 0.685530 |
| 1.Conserv~01 | 1.34 | 0.743553 |
| 1.Conserva~r | 1.27 | 0.785969 |
| 1.GeoCensu~1 | 1.67 | 0.597406 |
| 1.GeoCensu~2 | 1.87 | 0.533708 |
| 1.GeoCensu~3 | 1.58 | 0.633899 |
| 1.Current_~s | 1.10 | 0.906142 |
| Measures_c~r | 1.34 | 0.746803 |
| MA_Perc_Th~3 | 2.37 | 0.422207 |
| Costs_SC5 | 1.40 | 0.711754 |
| Deterr_SD_~2 | 1.73 | 0.577995 |
| Deterr_SD_~e | 1.20 | 0.830301 |
| MA_MoralBe~f | 2.18 | 0.458803 |
| MA_Authori~2 | 1.75 | 0.572710 |
| NOO_SC3 | 1.73 | 0.577578 |
| NNOO_SC3 | 1.44 | 0.694860 |
| OOL_SC12 | 1.41 | 0.707245 |
| PJE_SC4 | 1.44 | 0.696326 |
| Mean VIF | 1.55 | |

```
.
. *4.a.5. Effect size
```

```
164 . estat esize

    Effect sizes for linear models
```

| Source | Eta-Squared | df | [95% Conf. | Interval] |
|---|---|---|---|---|
| Model | .3969535 | 29 | .330649 | .4217786 |
| Age | .0053905 | 1 | . | .0190062 |
| Gender_Female | .0047541 | 1 | . | .0178164 |
| Minority | .0001554 | 1 | . | .0057121 |
| Education | .0068032 | 1 | .0002555 | .0215331 |
| Employed | .0001482 | 1 | . | .005648 |
| Corona_care | .0030299 | 1 | . | .0143566 |
| Insurance_Public | .0007954 | 1 | . | .0087028 |
| Insurance_Private | .0000757 | 1 | . | .0047942 |
| SES_before | .0030809 | 1 | . | .0144655 |
| SES_change | .000082 | 1 | . | .0048921 |
| Health_self | .0001608 | 1 | . | .005759 |
| Health_other | .0017892 | 1 | . | .0115068 |
| Conservative_01 | .0002335 | 1 | . | .0062937 |
| Conservative_other | .003155 | 1 | . | .0146229 |
| GeoCensus_d1 | .0000298 | 1 | . | .0037028 |
| GeoCensus_d2 | 9.36e-06 | 1 | . | .002393 |
| GeoCensus_d3 | .0018489 | 1 | . | .0116554 |
| Current_measures | .0067097 | 1 | .0002336 | .02137 |
| Measures_clear | .000957 | 1 | . | .0092207 |
| MA_Perc_Threat_SC3 | .0184813 | 1 | .0050344 | .0395495 |
| Costs_SC5 | .00492 | 1 | . | .0181302 |
| Deterr_SD_Likely_SC2 | .0003604 | 1 | . | .0070002 |
| Deterr_SD_Severe | .0003515 | 1 | . | .0069567 |
| MA_MoralBelief | .0863228 | 1 | .0543892 | .1227491 |
| MA_Authority_SC2 | .0000688 | 1 | . | .0046786 |
| NOO_SC3 | .0159072 | 1 | .0037204 | .0358444 |
| NNOO_SC3 | .0023847 | 1 | . | .0129295 |
| OOL_SC12 | .0022974 | 1 | . | .0127281 |
| PJE_SC4 | .000639 | 1 | . | .0081591 |

```
    Note: Eta-Squared values for individual model terms are partial.

165 .
166 . *4.a.6 Regression with vce(ro)
167 . reg DV_Compliance_SC7  Age i.Gender_Female i.Minority Education i.Employed i.Corona_care i.Insurance_Public i.Insura
    > vative_other i.GeoCensus_d1 i.GeoCensus_d2 i.GeoCensus_d3 i.Current_measures Measures_clear MA_Perc_Threat_SC3 Costs
    > C3 OOL_SC12 PJE_SC4 if chris_sample_reqs == 1, vce(ro)

    Linear regression                               Number of obs     =        921
                                                    F(29, 891)        =      21.16
                                                    Prob > F          =     0.0000
                                                    R-squared         =     0.3970
                                                    Root MSE          =     1.0984
```

| DV_Compliance_SC7 | Coef. | Robust Std. Err. | t | P>\|t\| | [95% Conf. | Interval] |
|---|---|---|---|---|---|---|
| Age | .0067611 | .0031132 | 2.17 | 0.030 | .0006511 | .012871 |
| 1.Gender_Female | .157395 | .0762577 | 2.06 | 0.039 | .0077293 | .3070606 |
| 1.Minority | -.029901 | .0852637 | -0.35 | 0.726 | -.1972421 | .1374402 |
| Education | .0671261 | .0267782 | 2.51 | 0.012 | .0145705 | .1196818 |
| 1.Employed | .0316137 | .0904971 | 0.35 | 0.727 | -.1459987 | .2092261 |
| 1.Corona_care | -.2269601 | .161245 | -1.41 | 0.160 | -.5434244 | .0895042 |
| 1.Insurance_Public | .1007447 | .1293969 | 0.78 | 0.436 | -.1532135 | .3547028 |
| 1.Insurance_Private | .0308161 | .1270688 | 0.24 | 0.808 | -.2185729 | .2802051 |
| SES_before | .0339202 | .0207319 | 1.64 | 0.102 | -.006769 | .0746093 |
| SES_change | .0063316 | .0261351 | 0.24 | 0.809 | -.0449619 | .0576251 |
| 2.Health_self | .0350109 | .0813079 | 0.43 | 0.667 | -.1245663 | .1945882 |
| 2.Health_other | -.1139342 | .08736 | -1.30 | 0.193 | -.2853896 | .0575211 |
| 1.Conservative_01 | .0384402 | .085559 | 0.45 | 0.653 | -.1294805 | .2063609 |
| 1.Conservative_other | .2051169 | .1335356 | 1.54 | 0.125 | -.056964 | .4671979 |
| 1.GeoCensus_d1 | -.0186564 | .1168606 | -0.16 | 0.873 | -.2480105 | .2106977 |
| 1.GeoCensus_d2 | -.0091827 | .103856 | -0.09 | 0.930 | -.2130137 | .1946483 |
| 1.GeoCensus_d3 | .1564985 | .1226423 | 1.28 | 0.202 | -.0842029 | .3971999 |

| | | | | | | |
|---|---|---|---|---|---|---|
| Current_measures | | | | | | |
| Yes | .271433 | .129263 | 2.10 | 0.036 | .0177375 | .5251285 |
| Measures_clear | .0213603 | .0246353 | 0.87 | 0.386 | -.0269897 | .0697103 |
| MA_Perc_Threat_SC3 | .1535816 | .043174 | 3.56 | 0.000 | .068847 | .2383162 |
| Costs_SC5 | .0549763 | .0269847 | 2.04 | 0.042 | .0020154 | .1079372 |
| Deterr_SD_Likely_SC2 | -.0155043 | .0306232 | -0.51 | 0.613 | -.0756062 | .0445977 |
| Deterr_SD_Severe | .0128621 | .0239155 | 0.54 | 0.591 | -.0340751 | .0597993 |
| MA_MoralBelief | .3608382 | .0502297 | 7.18 | 0.000 | .2622559 | .4594206 |
| MA_Authority_SC2 | -.0060891 | .0252138 | -0.24 | 0.809 | -.0555745 | .0433963 |
| NOO_SC3 | .1936145 | .0547683 | 3.54 | 0.000 | .0861247 | .3011043 |
| NNOO_SC3 | .0644588 | .0402739 | 1.60 | 0.110 | -.014584 | .1435015 |
| OOL_SC12 | .0414625 | .0277121 | 1.50 | 0.135 | -.0129261 | .0958511 |
| PJE_SC4 | -.0198905 | .025842 | -0.77 | 0.442 | -.0706087 | .0308278 |
| _cons | .2426497 | .4011932 | 0.60 | 0.545 | -.5447442 | 1.030044 |

```
168 .
169 .
170 . *****************
171 .
172 . *5. Step 5: Add personal factors
173 .
174 . *5.a.1 Descriptive Statistics
175 . sum DV_Compliance_SC7  Age i.Gender_Female i.Minority Education i.Employed i.Corona_care i.Insurance_Public i.Insura
    > vative_other i.GeoCensus_d1 i.GeoCensus_d2 i.GeoCensus_d3 i.Current_measures Measures_clear MA_Perc_Threat_SC3 Costs
    > C3 OOL_SC12 PJE_SC4 Trust_Science_SC4 Trust_in_media Impulsivity_SC4 NegEmo_SC6 if chris_sample_reqs == 1
```

| Variable | Obs | Mean | Std. Dev. | Min | Max |
|---|---|---|---|---|---|
| DV_Complia~7 | 921 | 5.76206 | 1.391929 | 1 | 7 |
| Age | 921 | 40.17155 | 12.8724 | 17 | 72 |
| Gender_Fem~e | | | | | |
| 0 | 921 | .4733985 | .4995631 | 0 | 1 |
| 1 | 921 | .5266015 | .4995631 | 0 | 1 |
| Minority | | | | | |
| 0 | 921 | .6666667 | .4716606 | 0 | 1 |
| 1 | 921 | .3333333 | .4716606 | 0 | 1 |
| Education | 921 | 3.756786 | 1.53705 | 1 | 8 |
| Employed | | | | | |
| 0 | 921 | .3821933 | .4861874 | 0 | 1 |
| 1 | 921 | .6178067 | .4861874 | 0 | 1 |
| Corona_care | | | | | |
| 0 | 921 | .9055375 | .2926301 | 0 | 1 |
| 1 | 921 | .0944625 | .2926301 | 0 | 1 |
| Insurance_~c | | | | | |
| 0 | 921 | .6612378 | .473546 | 0 | 1 |
| 1 | 921 | .3387622 | .473546 | 0 | 1 |
| Insurance_~e | | | | | |
| 0 | 921 | .4744843 | .4996198 | 0 | 1 |
| 1 | 921 | .5255157 | .4996198 | 0 | 1 |
| SES_before | 921 | 5.856678 | 2.096861 | 1 | 10 |
| SES_change | 921 | -.2290988 | 1.700595 | -9 | 8 |
| Health_self | | | | | |
| 1 | 921 | .6210641 | .4853857 | 0 | 1 |
| 2 | 921 | .3789359 | .4853857 | 0 | 1 |
| Health_other | | | | | |
| 1 | 921 | .3778502 | .4851133 | 0 | 1 |
| 2 | 921 | .6221498 | .4851133 | 0 | 1 |

| | | | | | |
|---|---|---|---|---|---|
| Conservat~01 | | | | | |
| 0 | 921 | .5439739 | .4983332 | 0 | 1 |
| 1 | 921 | .4560261 | .4983332 | 0 | 1 |
| Conservati~r | | | | | |
| 0 | 921 | .8718784 | .3344068 | 0 | 1 |
| 1 | 921 | .1281216 | .3344068 | 0 | 1 |
| GeoCensus_d1 | | | | | |
| 0 | 921 | .7871878 | .4095183 | 0 | 1 |
| 1 | 921 | .2128122 | .4095183 | 0 | 1 |
| GeoCensus_d2 | | | | | |
| 0 | 921 | .5852334 | .4929494 | 0 | 1 |
| 1 | 921 | .4147666 | .4929494 | 0 | 1 |
| GeoCensus_d3 | | | | | |
| 0 | 921 | .8327904 | .3733656 | 0 | 1 |
| 1 | 921 | .1672096 | .3733656 | 0 | 1 |
| Current_me~s | | | | | |
| 0 | 921 | .1368078 | .3438311 | 0 | 1 |
| Yes | 921 | .8631922 | .3438311 | 0 | 1 |
| Measures_c~r | 921 | 5.033659 | 1.812384 | 1 | 7 |
| MA_Perc_Th~3 | 921 | 5.740861 | 1.486305 | 1 | 7 |
| Costs_SC5 | 921 | 4.148534 | 1.638717 | 1 | 7 |
| Deterr_SD_~2 | 921 | 3.235613 | 1.741087 | 1 | 7 |
| Deterr_SD_~e | 921 | 3.890337 | 1.729515 | 1 | 6 |
| MA_MoralBe~f | 921 | 6.150923 | 1.359356 | 1 | 7 |
| MA_Authori~2 | 921 | 3.814332 | 1.945522 | 1 | 7 |
| NOO_SC3 | 921 | 3.899023 | .9339596 | 1 | 5 |
| NNOO_SC3 | 921 | 2.938835 | .9835599 | 1 | 5 |
| OOL_SC12 | 921 | 4.376945 | 1.487538 | 1 | 7 |
| PJE_SC4 | 921 | 5.079262 | 1.64679 | 1 | 7 |
| Trust_Scie~4 | 921 | 3.827633 | 1.001534 | 1 | 5 |
| Trust_in_m~a | 921 | 2.829533 | 1.342547 | 1 | 5 |
| Impulsivi~C4 | 921 | 2.45874 | 1.128404 | 1 | 5 |
| NegEmo_SC6 | 921 | 4.626131 | 1.569654 | 1 | 7 |

```
176 .
177 . *5.a.2 Regression
178 . reg DV_Compliance_SC7  Age i.Gender_Female i.Minority Education i.Employed i.Corona_care i.Insurance_Public i.Insura
    > vative_other i.GeoCensus_d1 i.GeoCensus_d2 i.GeoCensus_d3 i.Current_measures Measures_clear MA_Perc_Threat_SC3 Costs
    > C3 OOL_SC12 PJE_SC4 Trust_Science_SC4 Trust_in_media Impulsivity_SC4 NegEmo_SC6 if chris_sample_reqs == 1
```

| Source | SS | df | MS |
|---|---|---|---|
| Model | 739.173014 | 33 | 22.3991822 |
| Residual | 1043.29635 | 887 | 1.17620784 |
| Total | 1782.46937 | 920 | 1.9374667 |

| | | |
|---|---|---|
| Number of obs | = | 921 |
| F(33, 887) | = | 19.04 |
| Prob > F | = | 0.0000 |
| R-squared | = | 0.4147 |
| Adj R-squared | = | 0.3929 |
| Root MSE | = | 1.0845 |

| DV_Compliance_SC7 | Coef. | Std. Err. | t | P>\|t\| | [95% Conf. | Interval] |
|---|---|---|---|---|---|---|
| Age | .0057579 | .0030972 | 1.86 | 0.063 | -.0003208 | .0118366 |
| 1.Gender_Female | .1654323 | .0766232 | 2.16 | 0.031 | .0150484 | .3158163 |
| 1.Minority | -.0227417 | .0807127 | -0.28 | 0.778 | -.1811518 | .1356684 |
| Education | .0685656 | .0268947 | 2.55 | 0.011 | .015781 | .1213502 |
| 1.Employed | .0245453 | .0861104 | 0.29 | 0.776 | -.1444586 | .1935491 |
| 1.Corona_care | -.2121829 | .1377521 | -1.54 | 0.124 | -.482541 | .0581752 |
| 1.Insurance_Public | .074512 | .1183199 | 0.63 | 0.529 | -.1577077 | .3067316 |
| 1.Insurance_Private | .0095698 | .1176936 | 0.08 | 0.935 | -.2214206 | .2405603 |
| SES_before | .0289327 | .020382 | 1.42 | 0.156 | -.0110699 | .0689354 |
| SES_change | .0014415 | .0234481 | 0.06 | 0.951 | -.0445788 | .0474617 |
| 2.Health_self | .0717076 | .0916334 | 0.78 | 0.434 | -.108136 | .2515512 |
| 2.Health_other | -.153099 | .090283 | -1.70 | 0.090 | -.3302923 | .0240943 |
| 1.Conservative_01 | .1045296 | .087343 | 1.20 | 0.232 | -.0668935 | .2759527 |

| | | | | | | |
|---|---|---|---|---|---|---|
| 1.Conservative_other | .2515537 | .1221752 | 2.06 | 0.040 | .0117675 | .4913399 |
| 1.GeoCensus_d1 | -.0231716 | .1130095 | -0.21 | 0.838 | -.2449688 | .1986255 |
| 1.GeoCensus_d2 | -.0079432 | .0994831 | -0.08 | 0.936 | -.203193 | .1873066 |
| 1.GeoCensus_d3 | .1605265 | .1205487 | 1.33 | 0.183 | -.0760674 | .3971204 |
| | | | | | | |
| Current_measures | | | | | | |
| Yes | .2441804 | .1095834 | 2.23 | 0.026 | .0291074 | .4592534 |
| Measures_clear | .0069135 | .0234371 | 0.29 | 0.768 | -.0390852 | .0529122 |
| MA_Perc_Threat_SC3 | .1350257 | .0378669 | 3.57 | 0.000 | .0607066 | .2093448 |
| Costs_SC5 | .0524544 | .0266557 | 1.97 | 0.049 | .0001388 | .10477 |
| Deterr_SD_Likely_SC2 | .0005327 | .027617 | 0.02 | 0.985 | -.0536696 | .054735 |
| Deterr_SD_Severe | .0149425 | .0228615 | 0.65 | 0.514 | -.0299264 | .0598114 |
| MA_MoralBelief | .3416627 | .0392052 | 8.71 | 0.000 | .2647169 | .4186085 |
| MA_Authority_SC2 | -.0002603 | .0244899 | -0.01 | 0.992 | -.0483253 | .0478047 |
| NOO_SC3 | .1580391 | .0511155 | 3.09 | 0.002 | .0577176 | .2583606 |
| NNOO_SC3 | .0924185 | .044598 | 2.07 | 0.039 | .0048886 | .1799484 |
| OOL_SC12 | .0091396 | .0304823 | 0.30 | 0.764 | -.0506863 | .0689655 |
| PJE_SC4 | -.0221823 | .0260583 | -0.85 | 0.395 | -.0733255 | .0289609 |
| Trust_Science_SC4 | .2019897 | .0475592 | 4.25 | 0.000 | .108648 | .2953313 |
| Trust_in_media | -.025334 | .0337079 | -0.75 | 0.453 | -.0914904 | .0408225 |
| Impulsivity_SC4 | -.1194408 | .0403781 | -2.96 | 0.003 | -.1986886 | -.040193 |
| NegEmo_SC6 | -.0041776 | .0274041 | -0.15 | 0.879 | -.0579621 | .0496069 |
| _cons | .3663416 | .399148 | 0.92 | 0.359 | -.4170431 | 1.149726 |

```
179 . estimates store model_5

180 .
181 . *5.a.3 Check hettest: Run this right after your regression to apply the Breusch-Pagan / Cook-Weisberg test for heter
182 . *if significant, then you need to run the regression with vce(ro) at the end
183 . estat hettest

    Breusch-Pagan / Cook-Weisberg test for heteroskedasticity
             Ho: Constant variance
             Variables: fitted values of DV_Compliance_SC7

             chi2(1)      =    46.42
             Prob > chi2  =   0.0000

184 .
185 . *5.a.4. check vif, to check for for multicollinearity (VIFs >10 are problematic)
186 . vif
```

| Variable | VIF | 1/VIF |
|---|---|---|
| Age | 1.24 | 0.804334 |
| 1.Gender_F~e | 1.15 | 0.872559 |
| 1.Minority | 1.13 | 0.882172 |
| Education | 1.34 | 0.748149 |
| 1.Employed | 1.37 | 0.729420 |
| 1.Corona_c~e | 1.27 | 0.786796 |
| 1.Insuranc~c | 2.46 | 0.407245 |
| 1.Insuran~te | 2.70 | 0.369752 |
| SES_before | 1.43 | 0.699941 |
| SES_change | 1.24 | 0.804042 |
| 2.Health_s~f | 1.55 | 0.646270 |
| 2.Health_o~r | 1.50 | 0.666496 |
| 1.Conserv~01 | 1.48 | 0.674839 |
| 1.Conserva~r | 1.31 | 0.765914 |
| 1.GeoCensu~1 | 1.68 | 0.596925 |
| 1.GeoCensu~2 | 1.88 | 0.531609 |
| 1.GeoCensu~3 | 1.58 | 0.631106 |
| 1.Current_~s | 1.11 | 0.900568 |
| Measures_c~r | 1.41 | 0.708577 |
| MA_Perc_Th~3 | 2.48 | 0.403610 |
| Costs_SC5 | 1.49 | 0.670052 |
| Deterr_SD_~2 | 1.81 | 0.552971 |
| Deterr_SD_~e | 1.22 | 0.817785 |
| MA_MoralBe~f | 2.22 | 0.450135 |
| MA_Authori~2 | 1.78 | 0.563181 |
| NOO_SC3 | 1.78 | 0.560963 |
| NNOO_SC3 | 1.50 | 0.664452 |
| OOL_SC12 | 1.61 | 0.621819 |
| PJE_SC4 | 1.44 | 0.694267 |

| | | |
|---|---|---|
| Trust_Scie~4 | 1.77 | 0.563503 |
| Trust_in_m~a | 1.60 | 0.624274 |
| Impulsivi~C4 | 1.62 | 0.615849 |
| NegEmo_SC6 | 1.45 | 0.690966 |
| Mean VIF | 1.59 | |

```
187 .
188 . *5.a.5. Effect size
189 . estat esize
```

Effect sizes for linear models

| Source | Eta-Squared | df | [95% Conf. | Interval] |
|---|---|---|---|---|
| Model | .4146904 | 33 | .3464224 | .4369236 |
| Age | .0038813 | 1 | . | .0161528 |
| Gender_Female | .0052278 | 1 | . | .0187447 |
| Minority | .0000895 | 1 | . | .0050163 |
| Education | .0072742 | 1 | .000364 | .0223898 |
| Employed | .0000916 | 1 | . | .0050452 |
| Corona_care | .0026677 | 1 | . | .0136011 |
| Insurance_Public | .0004469 | 1 | . | .0074229 |
| Insurance_Private | 7.45e-06 | 1 | . | .0021424 |
| SES_before | .0022666 | 1 | . | .0126892 |
| SES_change | 4.26e-06 | 1 | . | .0015138 |
| Health_self | .0006899 | 1 | . | .008368 |
| Health_other | .0032315 | 1 | . | .0148193 |
| Conservative_01 | .0016121 | 1 | . | .0110873 |
| Conservative_other | .0047566 | 1 | . | .0178596 |
| GeoCensus_d1 | .0000474 | 1 | . | .0042509 |
| GeoCensus_d2 | 7.19e-06 | 1 | . | .0021014 |
| GeoCensus_d3 | .0019952 | 1 | . | .0120448 |
| Current_measures | .0055665 | 1 | . | .0193688 |
| Measures_clear | .0000981 | 1 | . | .005131 |
| MA_Perc_Threat_SC3 | .0141322 | 1 | .0028636 | .0332759 |
| Costs_SC5 | .0043468 | 1 | . | .0170715 |
| Deterr_SD_Likely_SC2 | 4.20e-07 | 1 | . | . |
| Deterr_SD_Severe | .0004814 | 1 | . | .0075702 |
| MA_MoralBelief | .0788689 | 1 | .0482168 | .1143533 |
| MA_Authority_SC2 | 1.27e-07 | 1 | . | . |
| NOO_SC3 | .0106621 | 1 | .0014228 | .027953 |
| NNOO_SC3 | .004818 | 1 | . | .017976 |
| OOL_SC12 | .0001013 | 1 | . | .0051721 |
| PJE_SC4 | .0008163 | 1 | . | .008799 |
| Trust_Science_SC4 | .0199307 | 1 | .0057934 | .0416506 |
| Trust_in_media | .0006364 | 1 | . | .0081754 |
| Impulsivity_SC4 | .0097685 | 1 | .0011051 | .0265282 |
| NegEmo_SC6 | .0000262 | 1 | . | .0035648 |

Note: Eta-Squared values for individual model terms are partial.

```
190 .
191 . *5.a.6 Regression with vce(ro)
192 . reg DV_Compliance_SC7  Age i.Gender_Female i.Minority Education i.Employed i.Corona_care i.Insurance_Public i.Insura
    > vative_other i.GeoCensus_d1 i.GeoCensus_d2 i.GeoCensus_d3 i.Current_measures Measures_clear MA_Perc_Threat_SC3 Costs
    > C3 OOL_SC12 PJE_SC4 Trust_Science_SC4 Trust_in_media Impulsivity_SC4 NegEmo_SC6 if chris_sample_reqs == 1, vce(ro)
```

Linear regression

| | | |
|---|---|---|
| Number of obs | = | 921 |
| F(33, 887) | = | 23.09 |
| Prob > F | = | 0.0000 |
| R-squared | = | 0.4147 |
| Root MSE | = | 1.0845 |

| DV_Compliance_SC7 | Coef. | Robust Std. Err. | t | P>\|t\| | [95% Conf. | Interval] |
|---|---|---|---|---|---|---|
| Age | .0057579 | .0030801 | 1.87 | 0.062 | -.0002872 | .011803 |
| 1.Gender_Female | .1654323 | .07678 | 2.15 | 0.031 | .0147407 | .316124 |
| 1.Minority | -.0227417 | .0849388 | -0.27 | 0.789 | -.1894462 | .1439628 |
| Education | .0685656 | .0264844 | 2.59 | 0.010 | .0165861 | .1205451 |
| 1.Employed | .0245453 | .0896472 | 0.27 | 0.784 | -.1514 | .2004905 |
| 1.Corona_care | -.2121829 | .1598259 | -1.33 | 0.185 | -.525864 | .1014982 |
| 1.Insurance_Public | .074512 | .1289557 | 0.58 | 0.564 | -.1785819 | .3276059 |
| 1.Insurance_Private | .0095698 | .1272224 | 0.08 | 0.940 | -.2401221 | .2592618 |
| SES_before | .0289327 | .0208207 | 1.39 | 0.165 | -.0119309 | .0697963 |
| SES_change | .0014415 | .0258502 | 0.06 | 0.956 | -.0492932 | .0521761 |
| 2.Health_self | .0717076 | .081009 | 0.89 | 0.376 | -.0872841 | .2306993 |
| 2.Health_other | -.153099 | .0847324 | -1.81 | 0.071 | -.3193984 | .0132003 |
| 1.Conservative_01 | .1045296 | .0876785 | 1.19 | 0.234 | -.0675519 | .2766111 |
| 1.Conservative_other | .2515537 | .1304274 | 1.93 | 0.054 | -.0044286 | .5075361 |
| 1.GeoCensus_d1 | -.0231716 | .1155824 | -0.20 | 0.841 | -.2500185 | .2036753 |
| 1.GeoCensus_d2 | -.0079432 | .1029331 | -0.08 | 0.939 | -.209964 | .1940776 |
| 1.GeoCensus_d3 | .1605265 | .1210758 | 1.33 | 0.185 | -.077102 | .398155 |
| Current_measures | | | | | | |
| Yes | .2441804 | .129015 | 1.89 | 0.059 | -.0090298 | .4973906 |
| Measures_clear | .0069135 | .0253471 | 0.27 | 0.785 | -.0428338 | .0566608 |
| MA_Perc_Threat_SC3 | .1350257 | .0431323 | 3.13 | 0.002 | .0503725 | .219679 |
| Costs_SC5 | .0524544 | .0279755 | 1.88 | 0.061 | -.0024516 | .1073604 |
| Deterr_SD_Likely_SC2 | .0005327 | .0315568 | 0.02 | 0.987 | -.0614019 | .0624674 |
| Deterr_SD_Severe | .0149425 | .0235406 | 0.63 | 0.526 | -.0312593 | .0611442 |
| MA_MoralBelief | .3416627 | .0491323 | 6.95 | 0.000 | .2452335 | .4380919 |
| MA_Authority_SC2 | -.0002603 | .0246886 | -0.01 | 0.992 | -.0487152 | .0481947 |
| NOO_SC3 | .1580391 | .0539246 | 2.93 | 0.003 | .0522044 | .2638737 |
| NNOO_SC3 | .0924185 | .0405939 | 2.28 | 0.023 | .0127473 | .1720897 |
| OOL_SC12 | .0091396 | .0299331 | 0.31 | 0.760 | -.0496082 | .0678875 |
| PJE_SC4 | -.0221823 | .025804 | -0.86 | 0.390 | -.0728262 | .0284617 |
| Trust_Science_SC4 | .2019897 | .0563579 | 3.58 | 0.000 | .0913793 | .3126 |
| Trust_in_media | -.025334 | .0328827 | -0.77 | 0.441 | -.0898709 | .039203 |
| Impulsivity_SC4 | -.1194408 | .0387302 | -3.08 | 0.002 | -.1954543 | -.0434274 |
| NegEmo_SC6 | -.0041776 | .0278944 | -0.15 | 0.881 | -.0589244 | .0505692 |
| _cons | .3663416 | .4323731 | 0.85 | 0.397 | -.4822522 | 1.214935 |

```
193 .
194 .
195 . *****************
196 .
197 . *6. Step 6: Add social environment
198 .
199 . *6.a.1 Descriptive Statistics
200 . sum DV_Compliance_SC7  Age i.Gender_Female i.Minority Education i.Employed i.Corona_care i.Insurance_Public i.Insura
    > vative_other i.GeoCensus_d1 i.GeoCensus_d2 i.GeoCensus_d3 i.Current_measures Measures_clear MA_Perc_Threat_SC3 Costs
    > C3 OOL_SC12 PJE_SC4 Trust_Science_SC4 Trust_in_media Impulsivity_SC4 NegEmo_SC6 SN_SC7 if chris_sample_reqs == 1
```

| Variable | Obs | Mean | Std. Dev. | Min | Max |
|---|---|---|---|---|---|
| DV_Complia~7 | 921 | 5.76206 | 1.391929 | 1 | 7 |
| Age | 921 | 40.17155 | 12.8724 | 17 | 72 |
| Gender_Fem~e | | | | | |
| 0 | 921 | .4733985 | .4995631 | 0 | 1 |
| 1 | 921 | .5266015 | .4995631 | 0 | 1 |
| Minority | | | | | |
| 0 | 921 | .6666667 | .4716606 | 0 | 1 |
| 1 | 921 | .3333333 | .4716606 | 0 | 1 |
| Education | 921 | 3.756786 | 1.53705 | 1 | 8 |
| Employed | | | | | |
| 0 | 921 | .3821933 | .4861874 | 0 | 1 |
| 1 | 921 | .6178067 | .4861874 | 0 | 1 |
| Corona_care | | | | | |

| | | | | | |
|---|---|---|---|---|---|
| 0 | 921 | .9055375 | .2926301 | 0 | 1 |
| 1 | 921 | .0944625 | .2926301 | 0 | 1 |
| Insurance_~c | | | | | |
| 0 | 921 | .6612378 | .473546 | 0 | 1 |
| 1 | 921 | .3387622 | .473546 | 0 | 1 |
| Insurance_~e | | | | | |
| 0 | 921 | .4744843 | .4996198 | 0 | 1 |
| 1 | 921 | .5255157 | .4996198 | 0 | 1 |
| SES_before | 921 | 5.856678 | 2.096861 | 1 | 10 |
| SES_change | 921 | -.2290988 | 1.700595 | -9 | 8 |
| Health_self | | | | | |
| 1 | 921 | .6210641 | .4853857 | 0 | 1 |
| 2 | 921 | .3789359 | .4853857 | 0 | 1 |
| Health_other | | | | | |
| 1 | 921 | .3778502 | .4851133 | 0 | 1 |
| 2 | 921 | .6221498 | .4851133 | 0 | 1 |
| Conservat~01 | | | | | |
| 0 | 921 | .5439739 | .4983332 | 0 | 1 |
| 1 | 921 | .4560261 | .4983332 | 0 | 1 |
| Conservati~r | | | | | |
| 0 | 921 | .8718784 | .3344068 | 0 | 1 |
| 1 | 921 | .1281216 | .3344068 | 0 | 1 |
| GeoCensus_d1 | | | | | |
| 0 | 921 | .7871878 | .4095183 | 0 | 1 |
| 1 | 921 | .2128122 | .4095183 | 0 | 1 |
| GeoCensus_d2 | | | | | |
| 0 | 921 | .5852334 | .4929494 | 0 | 1 |
| 1 | 921 | .4147666 | .4929494 | 0 | 1 |
| GeoCensus_d3 | | | | | |
| 0 | 921 | .8327904 | .3733656 | 0 | 1 |
| 1 | 921 | .1672096 | .3733656 | 0 | 1 |
| Current_me~s | | | | | |
| 0 | 921 | .1368078 | .3438311 | 0 | 1 |
| Yes | 921 | .8631922 | .3438311 | 0 | 1 |
| Measures_c~r | 921 | 5.033659 | 1.812384 | 1 | 7 |
| MA_Perc_Th~3 | 921 | 5.740861 | 1.486305 | 1 | 7 |
| Costs_SC5 | 921 | 4.148534 | 1.638717 | 1 | 7 |
| Deterr_SD_~2 | 921 | 3.235613 | 1.741087 | 1 | 7 |
| Deterr_SD_~e | 921 | 3.890337 | 1.729515 | 1 | 6 |
| MA_MoralBe~f | 921 | 6.150923 | 1.359356 | 1 | 7 |
| MA_Authori~2 | 921 | 3.814332 | 1.945522 | 1 | 7 |
| NOO_SC3 | 921 | 3.899023 | .9339596 | 1 | 5 |
| NNOO_SC3 | 921 | 2.938835 | .9835599 | 1 | 5 |
| OOL_SC12 | 921 | 4.376945 | 1.487538 | 1 | 7 |
| PJE_SC4 | 921 | 5.079262 | 1.64679 | 1 | 7 |
| Trust_Scie~4 | 921 | 3.827633 | 1.001534 | 1 | 5 |
| Trust_in_m~a | 921 | 2.829533 | 1.342547 | 1 | 5 |
| Impulsivi~C4 | 921 | 2.45874 | 1.128404 | 1 | 5 |
| NegEmo_SC6 | 921 | 4.626131 | 1.569654 | 1 | 7 |
| SN_SC7 | 921 | 5.077245 | 1.467881 | 1 | 7 |

```
201 .
202 . *6.a.2 Regression
203 . reg DV_Compliance_SC7  Age i.Gender_Female i.Minority Education i.Employed i.Corona_care i.Insurance_Public i.Insura
    > vative_other i.GeoCensus_d1 i.GeoCensus_d2 i.GeoCensus_d3 i.Current_measures Measures_clear MA_Perc_Threat_SC3 Costs
    > C3 OOL_SC12 PJE_SC4 Trust_Science_SC4 Trust_in_media Impulsivity_SC4 NegEmo_SC6 SN_SC7 if chris_sample_reqs == 1

      Source |       SS           df       MS      Number of obs   =       921
-------------+----------------------------------   F(34, 886)      =     20.25
       Model |  779.483742        34  22.9259924   Prob > F        =    0.0000
    Residual |  1002.98562       886  1.13203795   R-squared       =    0.4373
-------------+----------------------------------   Adj R-squared   =    0.4157
       Total |  1782.46937       920   1.9374667   Root MSE        =     1.064

-------------------------------------------------------------------------------------
   DV_Compliance_SC7 |      Coef.   Std. Err.      t    P>|t|     [95% Conf. Interval]
---------------------+---------------------------------------------------------------
                 Age |   .0037245   .0030575     1.22   0.224    -.0022764    .0097253
     1.Gender_Female |   .1517881   .0752055     2.02   0.044     .0041864    .2993898
          1.Minority |  -.0403135   .0792374    -0.51   0.611    -.1958285    .1152014
           Education |    .067435   .0263855     2.56   0.011     .0156496    .1192205
          1.Employed |   .0091519   .0845174     0.11   0.914    -.1567258    .1750297
       1.Corona_care |  -.2026489   .1351503    -1.50   0.134     -.467901    .0626032
  1.Insurance_Public |   .0779253   .1160784     0.67   0.502    -.1498955    .3057461
 1.Insurance_Private |  -.0188625   .1155609    -0.16   0.870    -.2456675    .2079425
          SES_before |   .0209066   .0200409     1.04   0.297    -.0184265    .0602397
          SES_change |  -.0023232   .0230123    -0.10   0.920    -.0474881    .0428417
       2.Health_self |   .0579507    .089926     0.64   0.519    -.1185421    .2344434
      2.Health_other |  -.1106802   .0888564    -1.25   0.213    -.2850738    .0637134
    1.Conservative_01 |   .0724631   .0858557     0.84   0.399    -.0960412    .2409673
1.Conservative_other |   .1973271   .1202032     1.64   0.101    -.0385892    .4332433
     1.GeoCensus_d1 |  -.0023022   .1109224    -0.02   0.983    -.2200035    .2153991
     1.GeoCensus_d2 |   .0004082   .0976074     0.00   0.997    -.1911604    .1919768
     1.GeoCensus_d3 |   .1037304   .1186459     0.87   0.382    -.1291295    .3365903
                     |
    Current_measures |
                 Yes |   .2191233   .1075881     2.04   0.042      .007966    .4302805
      Measures_clear |  -.0007849    .023029    -0.03   0.973    -.0459827    .0444129
  MA_Perc_Threat_SC3 |   .1348554   .0371491     3.63   0.000     .0619449    .2077658
           Costs_SC5 |     .05778   .0261656     2.21   0.027     .0064261    .1091338
Deterr_SD_Likely_SC2 |  -.0081291   .0271323    -0.30   0.765    -.0613803    .0451221
    Deterr_SD_Severe |   .0076835   .0224611     0.34   0.732    -.0363996    .0517666
      MA_MoralBelief |   .3210207   .0386173     8.31   0.000     .2452287    .3968126
    MA_Authority_SC2 |  -.0199963   .0242523    -0.82   0.410    -.0675949    .0276024
             NOO_SC3 |   .1491184   .0501689     2.97   0.003     .0506547     .247582
            NNOO_SC3 |   .0770683   .0438282     1.76   0.079    -.0089508    .1630874
            OOL_SC12 |   .0145152   .0299181     0.49   0.628    -.0442033    .0732337
             PJE_SC4 |  -.0349353   .0256535    -1.36   0.174    -.0852842    .0154135
   Trust_Science_SC4 |   .1586476   .0472196     3.36   0.001     .0659723    .2513229
      Trust_in_media |  -.0333384   .0330961    -1.01   0.314    -.0982943    .0316175
     Impulsivity_SC4 |  -.1204341   .0396131    -3.04   0.002    -.1981805   -.0426877
          NegEmo_SC6 |   .0005548   .0268963     0.02   0.984    -.0522331    .0533428
              SN_SC7 |   .1668663   .0279633     5.97   0.000     .1119842    .2217484
               _cons |   .2766351   .3918702     0.71   0.480     -.492467    1.045737
-------------------------------------------------------------------------------------

204 . estimates store model_6

205 .
206 . *6.a.3 Check hettest: Run this right after your regression to apply the Breusch-Pagan / Cook-Weisberg test for heter
207 . *if significant, then you need to run the regression with vce(ro) at the end
208 . estat hettest

    Breusch-Pagan / Cook-Weisberg test for heteroskedasticity
             Ho: Constant variance
             Variables: fitted values of DV_Compliance_SC7

             chi2(1)      =    45.44
             Prob > chi2  =   0.0000
```

```
209 .
210 . *6.a.4. check vif, to check for for multicollinearity (VIFs >10 are problematic)
211 . vif
```

| Variable | VIF | 1/VIF |
|---|---|---|
| Age | 1.26 | 0.794344 |
| 1.Gender_F~e | 1.15 | 0.871752 |
| 1.Minority | 1.14 | 0.880954 |
| Education | 1.34 | 0.748110 |
| 1.Employed | 1.37 | 0.728741 |
| 1.Corona_c~e | 1.27 | 0.786686 |
| 1.Insuranc~c | 2.46 | 0.407236 |
| 1.Insuran~te | 2.71 | 0.369124 |
| SES_before | 1.44 | 0.696788 |
| SES_change | 1.24 | 0.803438 |
| 2.Health_s~f | 1.55 | 0.645846 |
| 2.Health_o~r | 1.51 | 0.662230 |
| 1.Conserv~01 | 1.49 | 0.672195 |
| 1.Conserva~r | 1.31 | 0.761537 |
| 1.GeoCensu~1 | 1.68 | 0.596332 |
| 1.GeoCensu~2 | 1.88 | 0.531500 |
| 1.GeoCensu~3 | 1.59 | 0.627045 |
| 1.Current_~s | 1.11 | 0.899196 |
| Measures_c~r | 1.42 | 0.706353 |
| MA_Perc_Th~3 | 2.48 | 0.403609 |
| Costs_SC5 | 1.49 | 0.669273 |
| Deterr_SD_~2 | 1.81 | 0.551388 |
| Deterr_SD_~e | 1.23 | 0.815386 |
| MA_MoralBe~f | 2.24 | 0.446523 |
| MA_Authori~2 | 1.81 | 0.552708 |
| NOO_SC3 | 1.78 | 0.560465 |
| NNOO_SC3 | 1.51 | 0.662164 |
| OOL_SC12 | 1.61 | 0.621255 |
| PJE_SC4 | 1.45 | 0.689448 |
| Trust_Scie~4 | 1.82 | 0.550170 |
| Trust_in_m~a | 1.60 | 0.623248 |
| Impulsivi~C4 | 1.62 | 0.615838 |
| NegEmo_SC6 | 1.45 | 0.690365 |
| SN_SC7 | 1.37 | 0.730321 |
| Mean VIF | 1.59 | |

```
212 .
213 . *6.a.5. Effect size
214 . estat esize
```

Effect sizes for linear models

| Source | Eta-Squared | df | [95% Conf. | Interval] |
|---|---|---|---|---|
| Model | .4373055 | 34 | .3698367 | .458802 |
| Age | .001672 | 1 | . | .0112489 |
| Gender_Female | .0045767 | 1 | . | .0175253 |
| Minority | .0002921 | 1 | . | .0066723 |
| Education | .0073184 | 1 | .0003735 | .0224763 |
| Employed | .0000132 | 1 | . | .0027911 |
| Corona_care | .0025312 | 1 | . | .0133038 |
| Insurance_Public | .0005084 | 1 | . | .007688 |
| Insurance_Private | .0000301 | 1 | . | .0037257 |
| SES_before | .0012268 | 1 | . | .0100535 |
| SES_change | .0000115 | 1 | . | .0026327 |
| Health_self | .0004685 | 1 | . | .0075219 |
| Health_other | .0017481 | 1 | . | .0114424 |
| Conservative_01 | .0008034 | 1 | . | .008763 |
| Conservative_other | .0030324 | 1 | . | .0144052 |
| GeoCensus_d1 | 4.86e-07 | 1 | . | . |
| GeoCensus_d2 | 1.97e-08 | 1 | . | . |
| GeoCensus_d3 | .000862 | 1 | . | .0089549 |
| Current_measures | .00466 | 1 | . | .0176849 |
| Measures_clear | 1.31e-06 | 1 | . | .0001885 |
| MA_Perc_Threat_SC3 | .0146553 | 1 | .0031005 | .0340676 |

| | | | | |
|---|---|---|---|---|
| Costs_SC5 | .0054736 | 1 | . | .0192085 |
| Deterr_SD_Likely_SC2 | .0001013 | 1 | . | .005176 |
| Deterr_SD_Severe | .0001321 | 1 | . | .0055183 |
| MA_MoralBelief | .0723523 | 1 | .0429504 | .1068767 |
| MA_Authority_SC2 | .0007667 | 1 | . | .0086401 |
| NOO_SC3 | .009873 | 1 | .0011384 | .0267079 |
| NNOO_SC3 | .0034778 | 1 | . | .0153414 |
| OOL_SC12 | .0002656 | 1 | . | .0065198 |
| PJE_SC4 | .0020888 | 1 | . | .0122779 |
| Trust_Science_SC4 | .0125803 | 1 | .0021793 | .0309443 |
| Trust_in_media | .0011439 | 1 | . | .0098158 |
| Impulsivity_SC4 | .0103248 | 1 | .0012972 | .02743 |
| NegEmo_SC6 | 4.80e-07 | 1 | . | . |
| SN_SC7 | .0386378 | 1 | .0175787 | .0663704 |

Note: Eta-Squared values for individual model terms are partial.

```
215 .
216 . *6.a.6 Regression with vce(ro)
217 . reg DV_Compliance_SC7  Age i.Gender_Female i.Minority Education i.Employed i.Corona_care i.Insurance_Public i.Insura
    > vative_other i.GeoCensus_d1 i.GeoCensus_d2 i.GeoCensus_d3 i.Current_measures Measures_clear MA_Perc_Threat_SC3 Costs
    > C3 OOL_SC12 PJE_SC4 Trust_Science_SC4 Trust_in_media Impulsivity_SC4 NegEmo_SC6 SN_SC7 if chris_sample_reqs == 1, vc
```

Linear regression

| | | |
|---|---|---|
| Number of obs | = | 921 |
| F(34, 886) | = | 25.03 |
| Prob > F | = | 0.0000 |
| R-squared | = | 0.4373 |
| Root MSE | = | 1.064 |

| DV_Compliance_SC7 | Coef. | Robust Std. Err. | t | P>\|t\| | [95% Conf. | Interval] |
|---|---|---|---|---|---|---|
| Age | .0037245 | .0030493 | 1.22 | 0.222 | -.0022602 | .0097092 |
| 1.Gender_Female | .1517881 | .0747747 | 2.03 | 0.043 | .0050318 | .2985444 |
| 1.Minority | -.0403135 | .0835133 | -0.48 | 0.629 | -.2042204 | .1235933 |
| Education | .067435 | .0259892 | 2.59 | 0.010 | .0164274 | .1184426 |
| 1.Employed | .0091519 | .0882282 | 0.10 | 0.917 | -.1640087 | .1823125 |
| 1.Corona_care | -.2026489 | .1525492 | -1.33 | 0.184 | -.5020488 | .0967511 |
| 1.Insurance_Public | .0779253 | .1273488 | 0.61 | 0.541 | -.1720152 | .3278657 |
| 1.Insurance_Private | -.0188625 | .1246861 | -0.15 | 0.880 | -.263577 | .225852 |
| SES_before | .0209066 | .0199913 | 1.05 | 0.296 | -.0183292 | .0601424 |
| SES_change | -.0023232 | .0261884 | -0.09 | 0.929 | -.0537217 | .0490753 |
| 2.Health_self | .0579507 | .078932 | 0.73 | 0.463 | -.0969649 | .2128662 |
| 2.Health_other | -.1106802 | .0839941 | -1.32 | 0.188 | -.2755309 | .0541705 |
| 1.Conservative_01 | .0724631 | .0870004 | 0.83 | 0.405 | -.0982879 | .243214 |
| 1.Conservative_other | .1973271 | .1276536 | 1.55 | 0.123 | -.0532117 | .4478658 |
| 1.GeoCensus_d1 | -.0023022 | .1117183 | -0.02 | 0.984 | -.2215657 | .2169612 |
| 1.GeoCensus_d2 | .0004082 | .0996822 | 0.00 | 0.997 | -.1952325 | .196049 |
| 1.GeoCensus_d3 | .1037304 | .1197529 | 0.87 | 0.387 | -.131302 | .3387629 |
| Current_measures | | | | | | |
| Yes | .2191233 | .1266496 | 1.73 | 0.084 | -.0294449 | .4676915 |
| Measures_clear | -.0007849 | .025331 | -0.03 | 0.975 | -.0505007 | .0489309 |
| MA_Perc_Threat_SC3 | .1348554 | .0429692 | 3.14 | 0.002 | .0505221 | .2191887 |
| Costs_SC5 | .05778 | .0270202 | 2.14 | 0.033 | .0047488 | .1108111 |
| Deterr_SD_Likely_SC2 | -.0081291 | .0300762 | -0.27 | 0.787 | -.067158 | .0508998 |
| Deterr_SD_Severe | .0076835 | .0228626 | 0.34 | 0.737 | -.0371876 | .0525546 |
| MA_MoralBelief | .3210207 | .0476196 | 6.74 | 0.000 | .2275602 | .4144811 |
| MA_Authority_SC2 | -.0199963 | .0241677 | -0.83 | 0.408 | -.0674289 | .0274364 |
| NOO_SC3 | .1491184 | .0534869 | 2.79 | 0.005 | .0441425 | .2540942 |
| NNOO_SC3 | .0770683 | .0393527 | 1.96 | 0.050 | -.0001671 | .1543038 |
| OOL_SC12 | .0145152 | .0290583 | 0.50 | 0.618 | -.0425159 | .0715464 |
| PJE_SC4 | -.0349353 | .0253792 | -1.38 | 0.169 | -.0847457 | .014875 |
| Trust_Science_SC4 | .1586476 | .0548529 | 2.89 | 0.004 | .0509909 | .2663043 |
| Trust_in_media | -.0333384 | .0323153 | -1.03 | 0.303 | -.0967619 | .0300851 |
| Impulsivity_SC4 | -.1204341 | .0369454 | -3.26 | 0.001 | -.1929448 | -.0479234 |
| NegEmo_SC6 | .0005548 | .0277304 | 0.02 | 0.984 | -.0538701 | .0549797 |
| SN_SC7 | .1668663 | .0301483 | 5.53 | 0.000 | .1076959 | .2260367 |
| _cons | .2766351 | .422301 | 0.66 | 0.513 | -.5521918 | 1.105462 |

```
218 .
219 .
220 . *****************
221 .
222 . *7. Step 7: Add practical circumstances
223 .
224 . *7.a.1 Descriptive Statistics
225 . sum DV_Compliance_SC7  Age i.Gender_Female i.Minority Education i.Employed i.Corona_care i.Insurance_Public i.Insura
    > vative_other i.GeoCensus_d1 i.GeoCensus_d2 i.GeoCensus_d3 i.Current_measures Measures_clear MA_Perc_Threat_SC3 Costs
    > C3 OOL_SC12 PJE_SC4 Trust_Science_SC4 Trust_in_media Impulsivity_SC4 NegEmo_SC6 SN_SC7 CTC_SC7 OTC_SC7 if chris_samp
```

| Variable | Obs | Mean | Std. Dev. | Min | Max |
|---|---|---|---|---|---|
| DV_Complia~7 | 921 | 5.76206 | 1.391929 | 1 | 7 |
| Age | 921 | 40.17155 | 12.8724 | 17 | 72 |
| Gender_Fem~e | | | | | |
| 0 | 921 | .4733985 | .4995631 | 0 | 1 |
| 1 | 921 | .5266015 | .4995631 | 0 | 1 |
| Minority | | | | | |
| 0 | 921 | .6666667 | .4716606 | 0 | 1 |
| 1 | 921 | .3333333 | .4716606 | 0 | 1 |
| Education | 921 | 3.756786 | 1.53705 | 1 | 8 |
| Employed | | | | | |
| 0 | 921 | .3821933 | .4861874 | 0 | 1 |
| 1 | 921 | .6178067 | .4861874 | 0 | 1 |
| Corona_care | | | | | |
| 0 | 921 | .9055375 | .2926301 | 0 | 1 |
| 1 | 921 | .0944625 | .2926301 | 0 | 1 |
| Insurance_~c | | | | | |
| 0 | 921 | .6612378 | .473546 | 0 | 1 |
| 1 | 921 | .3387622 | .473546 | 0 | 1 |
| Insurance_~e | | | | | |
| 0 | 921 | .4744843 | .4996198 | 0 | 1 |
| 1 | 921 | .5255157 | .4996198 | 0 | 1 |
| SES_before | 921 | 5.856678 | 2.096861 | 1 | 10 |
| SES_change | 921 | -.2290988 | 1.700595 | -9 | 8 |
| Health_self | | | | | |
| 1 | 921 | .6210641 | .4853857 | 0 | 1 |
| 2 | 921 | .3789359 | .4853857 | 0 | 1 |
| Health_other | | | | | |
| 1 | 921 | .3778502 | .4851133 | 0 | 1 |
| 2 | 921 | .6221498 | .4851133 | 0 | 1 |
| Conservat~01 | | | | | |
| 0 | 921 | .5439739 | .4983332 | 0 | 1 |
| 1 | 921 | .4560261 | .4983332 | 0 | 1 |
| Conservati~r | | | | | |
| 0 | 921 | .8718784 | .3344068 | 0 | 1 |
| 1 | 921 | .1281216 | .3344068 | 0 | 1 |
| GeoCensus_d1 | | | | | |
| 0 | 921 | .7871878 | .4095183 | 0 | 1 |
| 1 | 921 | .2128122 | .4095183 | 0 | 1 |
| GeoCensus_d2 | | | | | |
| 0 | 921 | .5852334 | .4929494 | 0 | 1 |
| 1 | 921 | .4147666 | .4929494 | 0 | 1 |

| | | | | | |
|---|---|---|---|---|---|
| GeoCensus_d3 | | | | | |
| 0 | 921 | .8327904 | .3733656 | 0 | 1 |
| 1 | 921 | .1672096 | .3733656 | 0 | 1 |
| Current_me~s | | | | | |
| 0 | 921 | .1368078 | .3438311 | 0 | 1 |
| Yes | 921 | .8631922 | .3438311 | 0 | 1 |
| Measures_c~r | 921 | 5.033659 | 1.812384 | 1 | 7 |
| MA_Perc_Th~3 | 921 | 5.740861 | 1.486305 | 1 | 7 |
| Costs_SC5 | 921 | 4.148534 | 1.638717 | 1 | 7 |
| Deterr_SD_~2 | 921 | 3.235613 | 1.741087 | 1 | 7 |
| Deterr_SD_~e | 921 | 3.890337 | 1.729515 | 1 | 6 |
| MA_MoralBe~f | 921 | 6.150923 | 1.359356 | 1 | 7 |
| MA_Authori~2 | 921 | 3.814332 | 1.945522 | 1 | 7 |
| NOO_SC3 | 921 | 3.899023 | .9339596 | 1 | 5 |
| NNOO_SC3 | 921 | 2.938835 | .9835599 | 1 | 5 |
| OOL_SC12 | 921 | 4.376945 | 1.487538 | 1 | 7 |
| PJE_SC4 | 921 | 5.079262 | 1.64679 | 1 | 7 |
| Trust_Scie~4 | 921 | 3.827633 | 1.001534 | 1 | 5 |
| Trust_in_m~a | 921 | 2.829533 | 1.342547 | 1 | 5 |
| Impulsivi~C4 | 921 | 2.45874 | 1.128404 | 1 | 5 |
| NegEmo_SC6 | 921 | 4.626131 | 1.569654 | 1 | 7 |
| SN_SC7 | 921 | 5.077245 | 1.467881 | 1 | 7 |
| CTC_SC7 | 921 | 5.90895 | 1.079297 | 1 | 7 |
| OTC_SC7 | 921 | 4.611137 | 1.714485 | 1 | 7 |

```
226 .
227 . *7.a.2 Regression
228 . reg DV_Compliance_SC7  Age i.Gender_Female i.Minority Education i.Employed i.Corona_care i.Insurance_Public i.Insura
    > vative_other i.GeoCensus_d1 i.GeoCensus_d2 i.GeoCensus_d3 i.Current_measures Measures_clear MA_Perc_Threat_SC3 Costs
    > C3 OOL_SC12 PJE_SC4 Trust_Science_SC4 Trust_in_media Impulsivity_SC4 NegEmo_SC6 SN_SC7 CTC_SC7 OTC_SC7 if chris_samp
```

| Source | SS | df | MS | | | |
|---|---|---|---|---|---|---|
| | | | | Number of obs | = | 921 |
| | | | | F(36, 884) | = | 28.84 |
| Model | 962.816101 | 36 | 26.7448917 | Prob > F | = | 0.0000 |
| Residual | 819.653264 | 884 | .927209575 | R-squared | = | 0.5402 |
| | | | | Adj R-squared | = | 0.5214 |
| Total | 1782.46937 | 920 | 1.9374667 | Root MSE | = | .96292 |

| DV_Compliance_SC7 | Coef. | Std. Err. | t | P>\|t\| | [95% Conf. | Interval] |
|---|---|---|---|---|---|---|
| Age | .0043226 | .002768 | 1.56 | 0.119 | -.00111 | .0097552 |
| 1.Gender_Female | .1409138 | .068072 | 2.07 | 0.039 | .0073121 | .2745155 |
| 1.Minority | -.0066207 | .0717521 | -0.09 | 0.927 | -.147445 | .1342036 |
| Education | .0714067 | .0239022 | 2.99 | 0.003 | .024495 | .1183184 |
| 1.Employed | .0355793 | .076524 | 0.46 | 0.642 | -.1146106 | .1857692 |
| 1.Corona_care | -.0422826 | .1229352 | -0.34 | 0.731 | -.2835615 | .1989963 |
| 1.Insurance_Public | .0896369 | .1050613 | 0.85 | 0.394 | -.1165618 | .2958356 |
| 1.Insurance_Private | -.0330541 | .1047273 | -0.32 | 0.752 | -.2385973 | .1724892 |
| SES_before | .015131 | .0181432 | 0.83 | 0.405 | -.0204777 | .0507397 |
| SES_change | .0061254 | .0208356 | 0.29 | 0.769 | -.0347676 | .0470185 |
| 2.Health_self | .0730318 | .0814056 | 0.90 | 0.370 | -.0867389 | .2328025 |
| 2.Health_other | -.1035513 | .0804273 | -1.29 | 0.198 | -.2614019 | .0542994 |
| 1.Conservative_01 | .0374592 | .077784 | 0.48 | 0.630 | -.1152035 | .190122 |
| 1.Conservative_other | .1987363 | .1087868 | 1.83 | 0.068 | -.0147743 | .4122469 |
| 1.GeoCensus_d1 | -.0614745 | .1004755 | -0.61 | 0.541 | -.258673 | .1357239 |
| 1.GeoCensus_d2 | -.0799914 | .0885223 | -0.90 | 0.366 | -.2537299 | .0937471 |
| 1.GeoCensus_d3 | .041121 | .1074706 | 0.38 | 0.702 | -.1698063 | .2520482 |
| Current_measures | | | | | | |
| Yes | .1016512 | .0977554 | 1.04 | 0.299 | -.0902085 | .2935108 |
| Measures_clear | -.0207444 | .0208943 | -0.99 | 0.321 | -.0617527 | .0202638 |
| MA_Perc_Threat_SC3 | .114746 | .0337009 | 3.40 | 0.001 | .0486028 | .1808892 |
| Costs_SC5 | .0406876 | .0237136 | 1.72 | 0.087 | -.0058539 | .087229 |
| Deterr_SD_Likely_SC2 | -.0155964 | .0245613 | -0.63 | 0.526 | -.0638017 | .0326089 |
| Deterr_SD_Severe | .0009119 | .0203342 | 0.04 | 0.964 | -.038997 | .0408208 |
| MA_MoralBelief | .224306 | .0356402 | 6.29 | 0.000 | .1543567 | .2942553 |

| | | | | | | |
|---|---|---|---|---|---|---|
| MA_Authority_SC2 | -.0172224 | .0219754 | -0.78 | 0.433 | -.0603523 | .0259076 |
| NOO_SC3 | .061616 | .0458324 | 1.34 | 0.179 | -.028337 | .151569 |
| NNOO_SC3 | .1072172 | .0399543 | 2.68 | 0.007 | .0288007 | .1856336 |
| OOL_SC12 | .0165874 | .0272196 | 0.61 | 0.542 | -.0368352 | .07001 |
| PJE_SC4 | -.0185382 | .0232596 | -0.80 | 0.426 | -.0641887 | .0271123 |
| Trust_Science_SC4 | .1064422 | .0429049 | 2.48 | 0.013 | .0222348 | .1906496 |
| Trust_in_media | -.0160922 | .0300018 | -0.54 | 0.592 | -.0749754 | .0427909 |
| Impulsivity_SC4 | -.0764564 | .0360047 | -2.12 | 0.034 | -.1471212 | -.0057917 |
| NegEmo_SC6 | .0006676 | .0243444 | 0.03 | 0.978 | -.047112 | .0484472 |
| SN_SC7 | .0395316 | .0272453 | 1.45 | 0.147 | -.0139415 | .0930046 |
| CTC_SC7 | .5300955 | .0380319 | 13.94 | 0.000 | .4554521 | .6047388 |
| OTC_SC7 | -.0310681 | .0197299 | -1.57 | 0.116 | -.069791 | .0076548 |
| _cons | -.8149162 | .3720661 | -2.19 | 0.029 | -1.545152 | -.0846803 |

```
229 . estimates store model_7

230 .
231 . *7.a.3 Check hettest: Run this right after your regression to apply the Breusch-Pagan / Cook-Weisberg test for heter
232 . *if significant, then you need to run the regression with vce(ro) at the end
233 . estat hettest

Breusch-Pagan / Cook-Weisberg test for heteroskedasticity
         Ho: Constant variance
         Variables: fitted values of DV_Compliance_SC7

         chi2(1)      =    75.66
         Prob > chi2  =   0.0000

234 .
235 . *7.a.4. check vif, to check for for multicollinearity (VIFs >10 are problematic)
236 . vif
```

| Variable | VIF | 1/VIF |
|---|---|---|
| Age | 1.26 | 0.793859 |
| 1.Gender_F~e | 1.15 | 0.871509 |
| 1.Minority | 1.14 | 0.879958 |
| Education | 1.34 | 0.746687 |
| 1.Employed | 1.37 | 0.728094 |
| 1.Corona_c~e | 1.28 | 0.778753 |
| 1.Insuranc~c | 2.46 | 0.407174 |
| 1.Insuran~te | 2.72 | 0.368121 |
| SES_before | 1.44 | 0.696345 |
| SES_change | 1.25 | 0.802742 |
| 2.Health_s~f | 1.55 | 0.645517 |
| 2.Health_o~r | 1.51 | 0.662060 |
| 1.Conserv~01 | 1.49 | 0.670765 |
| 1.Conserva~r | 1.31 | 0.761530 |
| 1.GeoCensu~1 | 1.68 | 0.595282 |
| 1.GeoCensu~2 | 1.89 | 0.529273 |
| 1.GeoCensu~3 | 1.60 | 0.625954 |
| 1.Current_~s | 1.12 | 0.892110 |
| Measures_c~r | 1.42 | 0.702803 |
| MA_Perc_Th~3 | 2.49 | 0.401689 |
| Costs_SC5 | 1.50 | 0.667404 |
| Deterr_SD_~2 | 1.81 | 0.551120 |
| Deterr_SD_~e | 1.23 | 0.814871 |
| MA_MoralBe~f | 2.33 | 0.429381 |
| MA_Authori~2 | 1.81 | 0.551373 |
| NOO_SC3 | 1.82 | 0.550033 |
| NNOO_SC3 | 1.53 | 0.652620 |
| OOL_SC12 | 1.63 | 0.614738 |
| PJE_SC4 | 1.46 | 0.686924 |
| Trust_Scie~4 | 1.83 | 0.545814 |
| Trust_in_m~a | 1.61 | 0.621206 |
| Impulsivi~C4 | 1.64 | 0.610579 |
| NegEmo_SC6 | 1.45 | 0.690213 |
| SN_SC7 | 1.59 | 0.630122 |
| CTC_SC7 | 1.67 | 0.598153 |
| OTC_SC7 | 1.14 | 0.880792 |
| Mean VIF | 1.60 | |

```
237 .
238 . *7.a.5. Effect size
239 . estat esize
```

Effect sizes for linear models

| Source | Eta-Squared | df | [95% Conf. | Interval] |
|---|---|---|---|---|
| Model | .5401586 | 36 | .4802157 | .558994 |
| Age | .0027511 | 1 | . | .0138112 |
| Gender_Female | .0048241 | 1 | . | .0180166 |
| Minority | 9.63e-06 | 1 | . | .0024352 |
| Education | .0099951 | 1 | .0011756 | .026927 |
| Employed | .0002445 | 1 | . | .0064016 |
| Corona_care | .0001338 | 1 | . | .0055451 |
| Insurance_Public | .0008228 | 1 | . | .0088408 |
| Insurance_Private | .0001127 | 1 | . | .0053206 |
| SES_before | .0007862 | 1 | . | .0087192 |
| SES_change | .0000978 | 1 | . | .0051398 |
| Health_self | .0009096 | 1 | . | .0091209 |
| Health_other | .0018717 | 1 | . | .0117667 |
| Conservative_01 | .0002623 | 1 | . | .006511 |
| Conservative_other | .0037611 | 1 | . | .0159382 |
| GeoCensus_d1 | .0004233 | 1 | . | .0073367 |
| GeoCensus_d2 | .0009228 | 1 | . | .0091626 |
| GeoCensus_d3 | .0001656 | 1 | . | .0058338 |
| Current_measures | .0012217 | 1 | . | .0100536 |
| Measures_clear | .0011138 | 1 | . | .0097422 |
| MA_Perc_Threat_SC3 | .0129444 | 1 | .0023268 | .0315242 |
| Costs_SC5 | .0033192 | 1 | . | .0150298 |
| Deterr_SD_Likely_SC2 | .0004559 | 1 | . | .0074804 |
| Deterr_SD_Severe | 2.27e-06 | 1 | . | .0008079 |
| MA_MoralBelief | .0428857 | 1 | .0205365 | .0717202 |
| MA_Authority_SC2 | .0006943 | 1 | . | .0084033 |
| NOO_SC3 | .0020403 | 1 | . | .0121778 |
| NNOO_SC3 | .0080803 | 1 | .000573 | .0237901 |
| OOL_SC12 | .0004199 | 1 | . | .0073215 |
| PJE_SC4 | .0007181 | 1 | . | .0084866 |
| Trust_Science_SC4 | .0069143 | 1 | .0002712 | .0217999 |
| Trust_in_media | .0003253 | 1 | . | .0068636 |
| Impulsivity_SC4 | .0050751 | 1 | . | .0184895 |
| NegEmo_SC6 | 8.51e-07 | 1 | . | . |
| SN_SC7 | .0023759 | 1 | . | .0129667 |
| CTC_SC7 | .1801703 | 1 | .1372023 | .2241555 |
| OTC_SC7 | .0027971 | 1 | . | .0139123 |

Note: Eta-Squared values for individual model terms are partial.

```
240 .
241 . *7.a.6 Regression with vce(ro)
242 . reg DV_Compliance_SC7  Age i.Gender_Female i.Minority Education i.Employed i.Corona_care i.Insurance_Public i.Insura
    > vative_other i.GeoCensus_d1 i.GeoCensus_d2 i.GeoCensus_d3 i.Current_measures Measures_clear MA_Perc_Threat_SC3 Costs
    > C3 OOL_SC12 PJE_SC4 Trust_Science_SC4 Trust_in_media Impulsivity_SC4 NegEmo_SC6 SN_SC7 CTC_SC7 OTC_SC7 if chris_samp
```

Linear regression

| | | |
|---|---|---|
| Number of obs | = | 921 |
| F(36, 884) | = | 31.85 |
| Prob > F | = | 0.0000 |
| R-squared | = | 0.5402 |
| Root MSE | = | .96292 |

| DV_Compliance_SC7 | Coef. | Robust Std. Err. | t | P>\|t\| | [95% Conf. | Interval] |
|---|---|---|---|---|---|---|
| Age | .0043226 | .0029114 | 1.48 | 0.138 | -.0013915 | .0100367 |
| 1.Gender_Female | .1409138 | .0659942 | 2.14 | 0.033 | .0113902 | .2704373 |
| 1.Minority | -.0066207 | .0755274 | -0.09 | 0.930 | -.1548546 | .1416131 |
| Education | .0714067 | .0233638 | 3.06 | 0.002 | .0255516 | .1172618 |
| 1.Employed | .0355793 | .082068 | 0.43 | 0.665 | -.1254916 | .1966502 |
| 1.Corona_care | -.0422826 | .11736 | -0.36 | 0.719 | -.2726193 | .1880541 |
| 1.Insurance_Public | .0896369 | .1147313 | 0.78 | 0.435 | -.1355407 | .3148144 |
| 1.Insurance_Private | -.0330541 | .1160419 | -0.28 | 0.776 | -.2608038 | .1946957 |

```
             SES_before     .015131   .0183486     0.82   0.410    -.0208809    .0511429
             SES_change    .0061254   .0231947     0.26   0.792    -.0393977    .0516486
          2.Health_self    .0730318   .0704489     1.04   0.300    -.0652348    .2112984
         2.Health_other   -.1035513   .0755346    -1.37   0.171    -.2517994    .0446968
      1.Conservative_01    .0374592   .0782727     0.48   0.632    -.1161628    .1910813
   1.Conservative_other    .1987363   .1102846     1.80   0.072    -.0177139    .4151865
        1.GeoCensus_d1    -.0614745   .1013502    -0.61   0.544    -.2603896    .1374405
        1.GeoCensus_d2    -.0799914   .0900819    -0.89   0.375    -.2567907    .0968079
        1.GeoCensus_d3      .041121   .1112726     0.37   0.712    -.1772683    .2595103

       Current_measures
                    Yes    .1016512   .1206455     0.84   0.400    -.1351338    .3384362
         Measures_clear   -.0207444   .0243944    -0.85   0.395    -.0686222    .0271333
      MA_Perc_Threat_SC3    .114746   .0386599     2.97   0.003       .03887     .190622
              Costs_SC5    .0406876   .0247241     1.65   0.100    -.0078373    .0892124
    Deterr_SD_Likely_SC2  -.0155964   .0267747    -0.58   0.560    -.0681458     .036953
       Deterr_SD_Severe    .0009119   .0203815     0.04   0.964    -.0390898    .0409136
         MA_MoralBelief     .224306   .0421437     5.32   0.000     .1415925    .3070194
       MA_Authority_SC2   -.0172224   .0218583    -0.79   0.431    -.0601226    .0256779
                NOO_SC3     .061616   .0487738     1.26   0.207      -.03411     .157342
               NNOO_SC3    .1072172   .0357985     3.00   0.003     .0369572    .1774772
               OOL_SC12    .0165874   .0273166     0.61   0.544    -.0370256    .0702004
                PJE_SC4   -.0185382   .0222787    -0.83   0.406    -.0622636    .0251872
      Trust_Science_SC4    .1064422   .0469832     2.27   0.024     .0142305    .1986538
         Trust_in_media   -.0160922   .0291645    -0.55   0.581    -.0733321    .0411476
        Impulsivity_SC4   -.0764564   .0337794    -2.26   0.024    -.1427537   -.0101592
             NegEmo_SC6    .0006676   .0260866     0.03   0.980    -.0505312    .0518665
                 SN_SC7    .0395316   .0278917     1.42   0.157    -.0152101    .0942733
                CTC_SC7    .5300955   .0579914     9.14   0.000     .4162786    .6439123
                OTC_SC7   -.0310681   .0178835    -1.74   0.083    -.0661671    .0040309
                  _cons   -.8149162   .4099407    -1.99   0.047    -1.619487   -.0103456

243 .
244 .
245 .
246 . *****************
247 .
248 . *8. Compare Rsq models
249 .
250 . *8.a.1. model 1 vs model 2
251 . lrtest model_1 model_2

    Likelihood-ratio test                                 LR chi2(2)  =     52.07
    (Assumption: model_1 nested in model_2)               Prob > chi2 =    0.0000

252 .
253 . *8.a.2. model 2 vs model 3
254 . lrtest model_2 model_3

    Likelihood-ratio test                                 LR chi2(4)  =    206.28
    (Assumption: model_2 nested in model_3)               Prob > chi2 =    0.0000

255 .
256 . *8.a.3. model 3 vs model 4
257 . lrtest model_3 model_4

    Likelihood-ratio test                                 LR chi2(6)  =    121.09
    (Assumption: model_3 nested in model_4)               Prob > chi2 =    0.0000

258 .
259 . *8.a.4. model 4 vs model 5
```

```
260 . lrtest model_4 model_5

    Likelihood-ratio test                                 LR chi2(4)  =     27.50
    (Assumption: model_4 nested in model_5)               Prob > chi2 =    0.0000

261 .
262 . *8.a.5. model 5 vs model 6
263 . lrtest model_5 model_6

    Likelihood-ratio test                                 LR chi2(1)  =     36.29
    (Assumption: model_5 nested in model_6)               Prob > chi2 =    0.0000

264 .
265 . *8.a.6. model 6 vs model 7
266 . lrtest model_6 model_7

    Likelihood-ratio test                                 LR chi2(2)  =    185.91
    (Assumption: model_6 nested in model_7)               Prob > chi2 =    0.0000

267 .
268 .
269 . ****************
270 .
271 . log close
          name:  <unnamed>
           log:  C:\Users\creinde\OneDrive - UvA\RESEARCH\2020\20 03 Coronavirus-measures compliance survey\Data\US\NWO US
      log type:  smcl
     closed on:  17 Jun 2021, 22:53:08
```
