# Supplementary material for: Social distancing in America: Understanding long-term adherence to COVID-19 mitigation recommendations
Source: PLoS One. 2021 Sep 24;16(9):e0257945. doi: 10.1371/journal.pone.0257945 (PMC8462713; doi:10.1371/journal.pone.0257945)
Supplement: S4 Output — (PDF) [file pone.0257945.s012.pdf]

```
      ___  ____  ____  ____  ____(R)
     /__    /   ____/   /   ____/
    ___/   /   /___/   /   /___/
      Statistics/Data Analysis

 1 .
        name:  <unnamed>
         log:  C:\Users\creinde\OneDrive - UvA\RESEARCH\2020\20 03 Coronavirus-measures compliance survey\Data\US\NWO US
    log type:  smcl
   opened on:  17 Jun 2021, 22:31:33

 2 . use "C:\Users\creinde\OneDrive - UvA\RESEARCH\2020\20 03 Coronavirus-measures compliance survey\Data\US\NWO US Summe

 3 .
 4 .
 5 . ***************************************************************************************
 6 . ***************************************************************************************
 7 . *A. SELECTION CRITERION:
 8 .
 9 . * - Only if provided consent
10 . * - No missing data
11 . * - Both checks correct
12 . * - Nonbinary gender excluded (insufficient number)
13 .
14 . gen chris_sample_reqs = 1 if Consent == 1 & N_Missing == 0 & NChecksRight == 2 & Gender < 3
   (2,002 missing values generated)

15 .
16 . *B. SELECTION CRITERION:
17 .
18 . * - Only if provided consent
19 . * - Both checks correct
20 .
21 . *gen chris_sample_reqs = 1 if Consent == 1 & NChecksRight == 2
22 .
23 . *C. SELECTION CRITERION:
24 .
25 . * - Only if provided consent
26 . * - No missing data
27 .
28 . *gen chris_sample_reqs = 1 if Consent == 1 & N_Missing == 0
29 .
30 .
31 . tab chris_sample_reqs
```

chris_sample_reqs	Freq.	Percent	Cum.
1	**2,919**	**100.00**	**100.00**
Total	**2,919**	**100.00**	

```
32 .
33 . *generate wave dummies
34 .
35 . gen wave_d1 = 0

36 . replace wave_d1 = 1 if (WAVE == 2)
   (1,711 real changes made)

37 .
38 . gen wave_d2 = 0

39 . replace wave_d2 = 1 if (WAVE == 3)
   (1,758 real changes made)

40 .
```

```
.
. ********************************************************************************
. ********************************************************************************
. ********************************************************************************
. ********************************************************************************
.
. *HIERARCHICAL MODEL
.
. ********************************************************************************
. ********************************************************************************
. ********************************************************************************
. ********************************************************************************
.
. *1. Step 1: covariates only
.
. *1.a.1 Descriptive Statistics
. sum DV_Compliance_SC7 i.wave_d1 i.wave_d2 Age i.Gender_Female i.Minority Education i.Employed i.Corona_care i.Insura
> rvative_01 i.Conservative_other i.GeoCensus_d1 i.GeoCensus_d2 i.GeoCensus_d3 if chris_sample_reqs == 1
```

Variable	Obs	Mean	Std. Dev.	Min	Max
DV_Complia~7	2,919	5.856947	1.295922	1	7
wave_d1					
0	2,919	.6622131	.4730365	0	1
1	2,919	.3377869	.4730365	0	1
wave_d2					
0	2,919	.684481	.4648019	0	1
1	2,919	.315519	.4648019	0	1
Age	2,919	40.22234	13.05444	17	79
Gender_Fem~e					
0	2,919	.4546077	.4980206	0	1
1	2,919	.5453923	.4980206	0	1
Minority					
0	2,919	.6570743	.4747682	0	1
1	2,919	.3429257	.4747682	0	1
Education	2,919	3.857828	1.542854	1	8
Employed					
0	2,919	.3610826	.4803967	0	1
1	2,919	.6389174	.4803967	0	1
Corona_care					
0	2,919	.9122987	.2829084	0	1
1	2,919	.0877013	.2829084	0	1
Insurance_~c					
0	2,919	.4319973	.4954389	0	1
1	2,919	.5680027	.4954389	0	1
Insurance_~e					
0	2,919	.7060637	.4556412	0	1
1	2,919	.2939363	.4556412	0	1
SES_before	2,919	5.970538	2.050048	1	10
SES_change	2,919	-.2915382	1.654423	-9	9
Health_self					
1	2,919	.6605002	.4736207	0	1
2	2,919	.3394998	.4736207	0	1
Health_other					
1	2,919	.4162384	.4930185	0	1
2	2,919	.5837616	.4930185	0	1

Conservat~01					
0	2,919	.5477903	.4977961	0	1
1	2,919	.4522097	.4977961	0	1
Conservati~r					
0	2,919	.8800959	.324905	0	1
1	2,919	.1199041	.324905	0	1
GeoCensus_d1					
0	2,919	.7923947	.4056621	0	1
1	2,919	.2076053	.4056621	0	1
GeoCensus_d2					
0	2,919	.5721137	.4948571	0	1
1	2,919	.4278863	.4948571	0	1
GeoCensus_d3					
0	2,919	.8396711	.3669737	0	1
1	2,919	.1603289	.3669737	0	1

```
58 .
59 . *1.a.2 Regression
60 . reg DV_Compliance_SC7 i.wave_d1 i.wave_d2 Age i.Gender_Female i.Minority Education i.Employed i.Corona_care i.Insura
   > rvative_01 i.Conservative_other i.GeoCensus_d1 i.GeoCensus_d2 i.GeoCensus_d3 if chris_sample_reqs == 1
```

Source	SS	df	MS			
				Number of obs	=	2,919
				F(19, 2899)	=	12.48
Model	370.568928	19	19.5036278	Prob > F	=	0.0000
Residual	4529.96157	2,899	1.56259454	R-squared	=	0.0756
				Adj R-squared	=	0.0696
Total	4900.5305	2,918	1.67941415	Root MSE	=	1.25

DV_Compliance_SC7	Coef.	Std. Err.	t	P>|t|	[95% Conf.	Interval]
1.wave_d1	-.2274275	.0563164	-4.04	0.000	-.3378518	-.1170032
1.wave_d2	-.2428764	.0573423	-4.24	0.000	-.3553121	-.1304406
Age	.0112442	.0018566	6.06	0.000	.0076039	.0148845
1.Gender_Female	.2576536	.0478101	5.39	0.000	.1639083	.3513989
1.Minority	.176003	.0504718	3.49	0.000	.0770389	.2749672
Education	.0484999	.0168074	2.89	0.004	.0155443	.0814555
1.Employed	-.0634503	.0538989	-1.18	0.239	-.1691343	.0422338
1.Corona_care	-.2157033	.0862949	-2.50	0.012	-.384909	-.0464977
1.Insurance_Public	.0889272	.073665	1.21	0.227	-.0555138	.2333683
1.Insurance_Private	.1216517	.0772273	1.58	0.115	-.0297743	.2730777
SES_before	.0446428	.0124966	3.57	0.000	.0201397	.069146
SES_change	-.0051756	.0149421	-0.35	0.729	-.0344739	.0241226
2.Health_self	.1603787	.055392	2.90	0.004	.051767	.2689905
2.Health_other	.1262558	.0523501	2.41	0.016	.0236086	.228903
1.Conservative_01	-.2575815	.0504894	-5.10	0.000	-.3565802	-.1585828
1.Conservative_other	-.1238407	.0775217	-1.60	0.110	-.2758439	.0281626
1.GeoCensus_d1	-.3009583	.0728044	-4.13	0.000	-.4437119	-.1582047
1.GeoCensus_d2	-.2325394	.0631521	-3.68	0.000	-.356367	-.1087119
1.GeoCensus_d3	-.0389466	.077883	-0.50	0.617	-.1916583	.1137651
_cons	5.046692	.1477009	34.17	0.000	4.757083	5.336302

```
61 . estimates store model_1

62 .
```

```
63 . *1.a.3 Check hettest: Run this right after your regression to apply the Breusch-Pagan / Cook-Weisberg test for heter
64 . *if significant, then you need to run the regression with vce(ro) at the end
65 . estat hettest
```

```
Breusch-Pagan / Cook-Weisberg test for heteroskedasticity
         Ho: Constant variance
         Variables: fitted values of DV_Compliance_SC7

         chi2(1)      =   148.42
         Prob > chi2  =   0.0000
```

```
66 .
67 . *1.a.4. check vif, to check for for multicollinearity (VIFs >10 are problematic)
68 . vif
```

Variable	VIF	1/VIF
1.wave_d1	1.33	0.754573
1.wave_d2	1.33	0.753833
Age	1.10	0.911644
1.Gender_F~e	1.06	0.944553
1.Minority	1.07	0.932611
Education	1.26	0.796366
1.Employed	1.25	0.798732
1.Corona_c~e	1.11	0.898460
1.Insuranc~c	2.49	0.402030
1.Insuran~te	2.31	0.432487
SES_before	1.23	0.815920
SES_change	1.14	0.876283
2.Health_s~f	1.29	0.778045
2.Health_o~r	1.24	0.803895
1.Conserv~01	1.18	0.847731
1.Conserva~r	1.18	0.844114
1.GeoCensu~1	1.63	0.613927
1.GeoCensu~2	1.82	0.548309
1.GeoCensu~3	1.53	0.655550
Mean VIF	1.40	

```
69 .
70 . *1.a.5. Effect size
71 . estat esize
```

Effect sizes for linear models

Source	Eta-Squared	df	[95% Conf.	Interval]
Model	.0756181	19	.0527964	.0885519
wave_d1	.0055941	1	.0014824	.0122605
wave_d2	.0061503	1	.0017765	.0130698
Age	.0124947	1	.0057326	.0217019
Gender_Female	.0099187	1	.0040245	.0182987
Minority	.0041771	1	.0008008	.0101308
Education	.0028641	1	.0002935	.0080325
Employed	.0004778	1	.	.0033811
Corona_care	.0021506	1	.0000917	.0068114
Insurance_Public	.0005024	1	.	.0034459
Insurance_Private	.0008552	1	.	.0042903
SES_before	.0043829	1	.0008927	.0104472
SES_change	.0000414	1	.	.001706
Health_self	.0028833	1	.0002997	.0080645
Health_other	.0020024	1	.0000572	.0065472
Conservative_01	.0088982	1	.0033805	.0169177
Conservative_other	.0008795	1	.	.0043442
GeoCensus_d1	.00586	1	.0016214	.012649
GeoCensus_d2	.0046552	1	.0010183	.0108619
GeoCensus_d3	.0000863	1	.	.0020347

Note: Eta-Squared values for individual model terms are partial.

```
72 .
73 . *1.a.6 Regression with vce(ro)
74 . reg DV_Compliance_SC7 i.wave_d1 i.wave_d2 Age i.Gender_Female i.Minority Education i.Employed i.Corona_care i.Insura
   > rvative_01 i.Conservative_other i.GeoCensus_d1 i.GeoCensus_d2 i.GeoCensus_d3 if chris_sample_reqs == 1, vce(ro)
```

Linear regression

Number of obs	=	2,919
F(19, 2899)	=	12.08
Prob > F	=	0.0000
R-squared	=	0.0756
Root MSE	=	1.25

DV_Compliance_SC7	Coef.	Robust Std. Err.	t	P>|t|	[95% Conf.	Interval]
1.wave_d1	-.2274275	.0543894	-4.18	0.000	-.3340733	-.1207816
1.wave_d2	-.2428764	.0576431	-4.21	0.000	-.355902	-.1298507
Age	.0112442	.0018266	6.16	0.000	.0076626	.0148257
1.Gender_Female	.2576536	.0481512	5.35	0.000	.1632396	.3520676
1.Minority	.176003	.0493382	3.57	0.000	.0792615	.2727446
Education	.0484999	.0165431	2.93	0.003	.0160624	.0809374
1.Employed	-.0634503	.0546362	-1.16	0.246	-.17058	.0436795
1.Corona_care	-.2157033	.0873258	-2.47	0.014	-.3869302	-.0444765
1.Insurance_Public	.0889272	.0773388	1.15	0.250	-.0627174	.2405718
1.Insurance_Private	.1216517	.0835488	1.46	0.145	-.0421693	.2854728
SES_before	.0446428	.0132922	3.36	0.001	.0185797	.070706
SES_change	-.0051756	.0158948	-0.33	0.745	-.0363419	.0259907
2.Health_self	.1603787	.051409	3.12	0.002	.0595769	.2611806
2.Health_other	.1262558	.0526493	2.40	0.017	.0230219	.2294897
1.Conservative_01	-.2575815	.0493644	-5.22	0.000	-.3543744	-.1607886
1.Conservative_other	-.1238407	.0804342	-1.54	0.124	-.2815547	.0338734
1.GeoCensus_d1	-.3009583	.0736419	-4.09	0.000	-.4453541	-.1565625
1.GeoCensus_d2	-.2325394	.0613129	-3.79	0.000	-.3527606	-.1123182
1.GeoCensus_d3	-.0389466	.0702255	-0.55	0.579	-.1766435	.0987502
_cons	5.046692	.1568917	32.17	0.000	4.739062	5.354323

```
75 .
76 .
77 . *****************
78 .
79 . *2. Step 2: Add practical knowledge and understanding
80 .
81 . *2.a.1 Descriptive Statistics
82 . sum DV_Compliance_SC7 i.wave_d1 i.wave_d2 Age i.Gender_Female i.Minority Education i.Employed i.Corona_care i.Insura
   > rvative_01 i.Conservative_other i.GeoCensus_d1 i.GeoCensus_d2 i.GeoCensus_d3 i.Current_measures Measures_clear if ch
```

Variable	Obs	Mean	Std. Dev.	Min	Max
DV_Complia~7	2,919	5.856947	1.295922	1	7
wave_d1					
0	2,919	.6622131	.4730365	0	1
1	2,919	.3377869	.4730365	0	1
wave_d2					
0	2,919	.684481	.4648019	0	1
1	2,919	.315519	.4648019	0	1
Age	2,919	40.22234	13.05444	17	79
Gender_Fem~e					
0	2,919	.4546077	.4980206	0	1
1	2,919	.5453923	.4980206	0	1
Minority					
0	2,919	.6570743	.4747682	0	1
1	2,919	.3429257	.4747682	0	1
Education	2,919	3.857828	1.542854	1	8
Employed					

```
           0 |      2,919    .3610826    .4803967          0          1
           1 |      2,919    .6389174    .4803967          0          1
             |
 Corona_care |
           0 |      2,919    .9122987    .2829084          0          1
           1 |      2,919    .0877013    .2829084          0          1
-------------+---------------------------------------------------------
             |
Insurance_~c |
           0 |      2,919    .4319973    .4954389          0          1
           1 |      2,919    .5680027    .4954389          0          1
             |
Insurance_~e |
           0 |      2,919    .7060637    .4556412          0          1
           1 |      2,919    .2939363    .4556412          0          1
             |
  SES_before |      2,919    5.970538    2.050048          1         10
-------------+---------------------------------------------------------
  SES_change |      2,919   -.2915382    1.654423         -9          9
             |
 Health_self |
           1 |      2,919    .6605002    .4736207          0          1
           2 |      2,919    .3394998    .4736207          0          1
             |
Health_other |
           1 |      2,919    .4162384    .4930185          0          1
           2 |      2,919    .5837616    .4930185          0          1
-------------+---------------------------------------------------------
             |
Conservat~01 |
           0 |      2,919    .5477903    .4977961          0          1
           1 |      2,919    .4522097    .4977961          0          1
             |
Conservati~r |
           0 |      2,919    .8800959     .324905          0          1
           1 |      2,919    .1199041     .324905          0          1
             |
GeoCensus_d1 |
           0 |      2,919    .7923947    .4056621          0          1
-------------+---------------------------------------------------------
           1 |      2,919    .2076053    .4056621          0          1
             |
GeoCensus_d2 |
           0 |      2,919    .5721137    .4948571          0          1
           1 |      2,919    .4278863    .4948571          0          1
             |
GeoCensus_d3 |
           0 |      2,919    .8396711    .3669737          0          1
           1 |      2,919    .1603289    .3669737          0          1
-------------+---------------------------------------------------------
             |
Current_me~s |
           0 |      2,919    .1342926    .3410248          0          1
         Yes |      2,919    .8657074    .3410248          0          1
             |
Measures_c~r |      2,919    5.186023    1.727691          1          7

 83 .
 84 . *2.a.2 Regression
 85 . reg DV_Compliance_SC7 i.wave_d1 i.wave_d2 Age i.Gender_Female i.Minority Education i.Employed i.Corona_care i.Insura
    > rvative_01 i.Conservative_other i.GeoCensus_d1 i.GeoCensus_d2 i.GeoCensus_d3 i.Current_measures Measures_clear if ch

      Source |       SS           df       MS      Number of obs   =     2,919
-------------+----------------------------------   F(21, 2897)     =     23.18
       Model |  705.023913        21  33.5725673   Prob > F        =    0.0000
    Residual |  4195.50659     2,897  1.44822457   R-squared       =    0.1439
-------------+----------------------------------   Adj R-squared   =    0.1377
       Total |   4900.5305     2,918  1.67941415   Root MSE        =    1.2034
```

DV_Compliance_SC7	Coef.	Std. Err.	t	P>|t|	[95% Conf.	Interval]
1.wave_d1	-.1451837	.054508	-2.66	0.008	-.2520619	-.0383054
1.wave_d2	-.1708034	.0554236	-3.08	0.002	-.279477	-.0621298
Age	.0076057	.0018033	4.22	0.000	.0040698	.0111416
1.Gender_Female	.2316096	.0460641	5.03	0.000	.141288	.3219313
1.Minority	.119028	.048734	2.44	0.015	.0234711	.2145849
Education	.0643467	.0162166	3.97	0.000	.0325494	.0961439
1.Employed	-.0582667	.0519329	-1.12	0.262	-.1600957	.0435624
1.Corona_care	-.2617609	.0831394	-3.15	0.002	-.4247793	-.0987426
1.Insurance_Public	.0474593	.0709753	0.67	0.504	-.0917079	.1866265
1.Insurance_Private	.0687309	.0744509	0.92	0.356	-.0772511	.2147129
SES_before	.0289393	.0120819	2.40	0.017	.0052493	.0526293
SES_change	-.0061142	.0143891	-0.42	0.671	-.034328	.0220996
2.Health_self	.1690781	.053338	3.17	0.002	.0644938	.2736624
2.Health_other	.0830741	.0505348	1.64	0.100	-.0160136	.1821619
1.Conservative_01	-.233866	.0486316	-4.81	0.000	-.329222	-.13851
1.Conservative_other	-.0646267	.0747413	-0.86	0.387	-.2111782	.0819248
1.GeoCensus_d1	-.2380195	.0702133	-3.39	0.001	-.3756925	-.1003465
1.GeoCensus_d2	-.1712318	.060939	-2.81	0.005	-.29072	-.0517436
1.GeoCensus_d3	.0035682	.075043	0.05	0.962	-.1435749	.1507112
Current_measures						
Yes	.6605429	.0681974	9.69	0.000	.5268226	.7942631
Measures_clear	.1265811	.0134555	9.41	0.000	.1001977	.1529645
_cons	3.978475	.1588091	25.05	0.000	3.667085	4.289865

```
86 . estimates store model_2

87 .
88 . *2.a.3 Check hettest: Run this right after your regression to apply the Breusch-Pagan / Cook-Weisberg test for heter
89 . *if significant, then you need to run the regression with vce(ro) at the end
90 . estat hettest

   Breusch-Pagan / Cook-Weisberg test for heteroskedasticity
            Ho: Constant variance
            Variables: fitted values of DV_Compliance_SC7

            chi2(1)      =   259.90
            Prob > chi2  =   0.0000

91 .
92 . *2.a.4. check vif, to check for for multicollinearity (VIFs >10 are problematic)
93 . vif
```

Variable	VIF	1/VIF
1.wave_d1	1.34	0.746520
1.wave_d2	1.34	0.747870
Age	1.12	0.895558
1.Gender_F~e	1.06	0.943043
1.Minority	1.08	0.927091
Education	1.26	0.792832
1.Employed	1.25	0.797382
1.Corona_c~e	1.11	0.897108
1.Insuranc~c	2.49	0.401380
1.Insuran~te	2.32	0.431286
SES_before	1.24	0.809008
SES_change	1.14	0.875775
2.Health_s~f	1.29	0.777706
2.Health_o~r	1.25	0.799546
1.Conserv~01	1.18	0.846859
1.Conserva~r	1.19	0.841621
1.GeoCensu~1	1.63	0.611763
1.GeoCensu~2	1.83	0.545758
1.GeoCensu~3	1.53	0.654426
1.Current_~s	1.09	0.917579
Measures_c~r	1.09	0.918366
Mean VIF	1.37	

```
94 .
95 . *2.a.5. Effect size
96 . estat esize
```

Effect sizes for linear models

Source	Eta-Squared	df	[95% Conf.	Interval]
Model	.1438669	21	.1156543	.1609929
wave_d1	.0024429	1	.0001668	.0073229
wave_d2	.0032676	1	.0004323	.0086966
Age	.0061028	1	.0017499	.013004
Gender_Female	.008651	1	.0032265	.0165831
Minority	.0020549	1	.0000689	.0066433
Education	.0054054	1	.0013847	.0119856
Employed	.0004343	1	.	.0032655
Corona_care	.0034101	1	.0004853	.0089263
Insurance_Public	.0001543	1	.	.0023625
Insurance_Private	.0002941	1	.	.0028569
SES_before	.0019765	1	.0000512	.0065027
SES_change	.0000623	1	.	.0018821
Health_self	.0034566	1	.000503	.0090009
Health_other	.000932	1	.	.0044609
Conservative_01	.0079195	1	.0027835	.0155747
Conservative_other	.000258	1	.	.002741
GeoCensus_d1	.0039511	1	.0007027	.0097824
GeoCensus_d2	.002718	1	.0002469	.0077905
GeoCensus_d3	7.80e-07	1	.	.0002876
Current_measures	.0313673	1	.0200888	.0448017
Measures_clear	.0296427	1	.0186952	.0427721

Note: Eta-Squared values for individual model terms are partial.

```
97 .
98 . *2.a.6 Regression with vce(ro)
99 . reg DV_Compliance_SC7 i.wave_d1 i.wave_d2 Age i.Gender_Female i.Minority Education i.Employed i.Corona_care i.Insura
   > rvative_01 i.Conservative_other i.GeoCensus_d1 i.GeoCensus_d2 i.GeoCensus_d3 i.Current_measures Measures_clear if ch
```

Linear regression

Number of obs	=	2,919
F(21, 2897)	=	18.87
Prob > F	=	0.0000
R-squared	=	0.1439
Root MSE	=	1.2034

DV_Compliance_SC7	Coef.	Robust Std. Err.	t	P>|t|	[95% Conf.	Interval]
1.wave_d1	-.1451837	.0523512	-2.77	0.006	-.247833	-.0425343
1.wave_d2	-.1708034	.0557823	-3.06	0.002	-.2801803	-.0614265
Age	.0076057	.0017815	4.27	0.000	.0041126	.0110988
1.Gender_Female	.2316096	.0464549	4.99	0.000	.1405217	.3226976
1.Minority	.119028	.0480521	2.48	0.013	.0248082	.2132477
Education	.0643467	.0161462	3.99	0.000	.0326875	.0960058
1.Employed	-.0582667	.0529948	-1.10	0.272	-.162178	.0456447
1.Corona_care	-.2617609	.0856433	-3.06	0.002	-.4296889	-.093833
1.Insurance_Public	.0474593	.074261	0.64	0.523	-.0981505	.1930691
1.Insurance_Private	.0687309	.0799522	0.86	0.390	-.0880381	.2254999
SES_before	.0289393	.0126347	2.29	0.022	.0041654	.0537132
SES_change	-.0061142	.015335	-0.40	0.690	-.0361827	.0239544
2.Health_self	.1690781	.0497282	3.40	0.001	.071572	.2665843
2.Health_other	.0830741	.0502787	1.65	0.099	-.0155116	.1816599
1.Conservative_01	-.233866	.0476366	-4.91	0.000	-.327271	-.1404609
1.Conservative_other	-.0646267	.0776894	-0.83	0.406	-.2169588	.0877054
1.GeoCensus_d1	-.2380195	.0712952	-3.34	0.001	-.3778139	-.098225
1.GeoCensus_d2	-.1712318	.0592208	-2.89	0.004	-.287351	-.0551126
1.GeoCensus_d3	.0035682	.0680992	0.05	0.958	-.1299596	.137096
Current_measures						
Yes	.6605429	.0871969	7.58	0.000	.4895687	.831517
Measures_clear	.1265811	.0150405	8.42	0.000	.0970899	.1560722
_cons	3.978475	.1742723	22.83	0.000	3.636765	4.320185

```
100 .
101 .
102 . *****************
103 .
104 . *3. Step 3: Add costs + benefits
105 .
106 . *3.a.1 Descriptive Statistics
107 . sum DV_Compliance_SC7 i.wave_d1 i.wave_d2 Age i.Gender_Female i.Minority Education i.Employed i.Corona_care i.Insura
    > rvative_01 i.Conservative_other i.GeoCensus_d1 i.GeoCensus_d2 i.GeoCensus_d3 i.Current_measures Measures_clear MA_Pe
```

Variable	Obs	Mean	Std. Dev.	Min	Max
DV_Complia~7	2,919	5.856947	1.295922	1	7
wave_d1					
0	2,919	.6622131	.4730365	0	1
1	2,919	.3377869	.4730365	0	1
wave_d2					
0	2,919	.684481	.4648019	0	1
1	2,919	.315519	.4648019	0	1
Age	2,919	40.22234	13.05444	17	79
Gender_Fem~e					
0	2,919	.4546077	.4980206	0	1
1	2,919	.5453923	.4980206	0	1
Minority					
0	2,919	.6570743	.4747682	0	1
1	2,919	.3429257	.4747682	0	1
Education	2,919	3.857828	1.542854	1	8
Employed					
0	2,919	.3610826	.4803967	0	1
1	2,919	.6389174	.4803967	0	1
Corona_care					
0	2,919	.9122987	.2829084	0	1
1	2,919	.0877013	.2829084	0	1
Insurance_~c					
0	2,919	.4319973	.4954389	0	1
1	2,919	.5680027	.4954389	0	1
Insurance_~e					
0	2,919	.7060637	.4556412	0	1
1	2,919	.2939363	.4556412	0	1
SES_before	2,919	5.970538	2.050048	1	10
SES_change	2,919	-.2915382	1.654423	-9	9
Health_self					
1	2,919	.6605002	.4736207	0	1
2	2,919	.3394998	.4736207	0	1
Health_other					
1	2,919	.4162384	.4930185	0	1
2	2,919	.5837616	.4930185	0	1
Conservat~01					
0	2,919	.5477903	.4977961	0	1
1	2,919	.4522097	.4977961	0	1
Conservati~r					
0	2,919	.8800959	.324905	0	1

1	2,919	.1199041	.324905	0	1
GeoCensus_d1					
0	2,919	.7923947	.4056621	0	1
1	2,919	.2076053	.4056621	0	1
GeoCensus_d2					
0	2,919	.5721137	.4948571	0	1
1	2,919	.4278863	.4948571	0	1
GeoCensus_d3					
0	2,919	.8396711	.3669737	0	1
1	2,919	.1603289	.3669737	0	1
Current_me~s					
0	2,919	.1342926	.3410248	0	1
Yes	2,919	.8657074	.3410248	0	1
Measures_c~r	2,919	5.186023	1.727691	1	7
MA_Perc_Th~3	2,919	5.620418	1.506602	1	7
Costs_SC5	2,919	4.18383	1.639894	1	7
Deterr_SD_~2	2,919	3.256252	1.762824	1	7
Deterr_SD_~e	2,919	3.830079	1.718316	1	6

```
108 .
109 . *3.a.2 Regression
110 . reg DV_Compliance_SC7 i.wave_d1 i.wave_d2 Age i.Gender_Female i.Minority Education i.Employed i.Corona_care i.Insura
    > rvative_01 i.Conservative_other i.GeoCensus_d1 i.GeoCensus_d2 i.GeoCensus_d3 i.Current_measures Measures_clear MA_Pe
```

Source	SS	df	MS
Model	1511.07475	25	60.4429898
Residual	3389.45575	2,893	1.17160586
Total	4900.5305	2,918	1.67941415

Number of obs	=	2,919
F(25, 2893)	=	51.59
Prob > F	=	0.0000
R-squared	=	0.3083
Adj R-squared	=	0.3024
Root MSE	=	1.0824

DV_Compliance_SC7	Coef.	Std. Err.	t	P>|t|	[95% Conf.	Interval]
1.wave_d1	-.1303741	.0491378	-2.65	0.008	-.2267227	-.0340254
1.wave_d2	-.2343954	.0500215	-4.69	0.000	-.3324767	-.136314
Age	.0072007	.0016497	4.36	0.000	.003966	.0104354
1.Gender_Female	.2041719	.0415518	4.91	0.000	.1226977	.285646
1.Minority	-.0112874	.0443726	-0.25	0.799	-.0982924	.0757176
Education	.058065	.0146001	3.98	0.000	.0294374	.0866926
1.Employed	-.045634	.0467424	-0.98	0.329	-.1372857	.0460177
1.Corona_care	-.3334566	.0761442	-4.38	0.000	-.4827589	-.1841542
1.Insurance_Public	.0841396	.0642827	1.31	0.191	-.041905	.2101842
1.Insurance_Private	.0652998	.0671258	0.97	0.331	-.0663195	.1969191
SES_before	.0296033	.0110078	2.69	0.007	.0080195	.0511872
SES_change	.0171519	.0132235	1.30	0.195	-.0087766	.0430805
2.Health_self	-.0471238	.0488629	-0.96	0.335	-.1429334	.0486858
2.Health_other	-.0185089	.0460982	-0.40	0.688	-.1088976	.0718798
1.Conservative_01	-.0087877	.0447245	-0.20	0.844	-.0964828	.0789074
1.Conservative_other	.0998301	.0675477	1.48	0.140	-.0326163	.2322765
1.GeoCensus_d1	-.1330702	.0633014	-2.10	0.036	-.2571905	-.0089499
1.GeoCensus_d2	-.1356816	.054831	-2.47	0.013	-.2431933	-.0281699
1.GeoCensus_d3	.0067938	.0675415	0.10	0.920	-.1256404	.1392281
Current_measures						
Yes	.4494543	.0618795	7.26	0.000	.3281219	.5707866
Measures_clear	.0570116	.0124584	4.58	0.000	.0325834	.0814399
MA_Perc_Threat_SC3	.3856428	.0153286	25.16	0.000	.3555868	.4156988
Costs_SC5	.0295498	.0139305	2.12	0.034	.0022352	.0568645
Deterr_SD_Likely_SC2	-.0089444	.0132237	-0.68	0.499	-.0348731	.0169844
Deterr_SD_Severe	.0097617	.0125009	0.78	0.435	-.0147498	.0342731
_cons	2.292767	.1786861	12.83	0.000	1.942402	2.643132

```
111 . estimates store model_3

112 .
113 . *3.a.3 Check hettest: Run this right after your regression to apply the Breusch-Pagan / Cook-Weisberg test for heter
114 . *if significant, then you need to run the regression with vce(ro) at the end
115 . estat hettest

    Breusch-Pagan / Cook-Weisberg test for heteroskedasticity
             Ho: Constant variance
             Variables: fitted values of DV_Compliance_SC7

             chi2(1)      =   381.09
             Prob > chi2  =   0.0000

116 .
117 . *3.a.4. check vif, to check for for multicollinearity (VIFs >10 are problematic)
118 . vif
```

Variable	VIF	1/VIF
1.wave_d1	1.35	0.743148
1.wave_d2	1.35	0.742758
Age	1.16	0.865718
1.Gender_F~e	1.07	0.937608
1.Minority	1.11	0.904699
Education	1.26	0.791293
1.Employed	1.26	0.796296
1.Corona_c~e	1.16	0.865228
1.Insuranc~c	2.53	0.395847
1.Insuran~te	2.33	0.429211
SES_before	1.27	0.788445
SES_change	1.19	0.838895
2.Health_s~f	1.33	0.749681
2.Health_o~r	1.29	0.777322
1.Conserv~01	1.23	0.810032
1.Conserva~r	1.20	0.833610
1.GeoCensu~1	1.64	0.608894
1.GeoCensu~2	1.83	0.545362
1.GeoCensu~3	1.53	0.653560
1.Current_~s	1.11	0.901635
Measures_c~r	1.15	0.866643
MA_Perc_Th~3	1.33	0.752827
Costs_SC5	1.30	0.769368
Deterr_SD_~2	1.35	0.738882
Deterr_SD_~e	1.15	0.870183
Mean VIF	1.38	

```
119 .
120 . *3.a.5. Effect size
121 . estat esize

    Effect sizes for linear models
```

Source	Eta-Squared	df	[95% Conf.	Interval]
Model	.3083492	25	.2764366	.3280919
wave_d1	.0024274	1	.0001618	.0073005
wave_d2	.0075327	1	.0025525	.0150419
Age	.0065425	1	.0019879	.0136419
Gender_Female	.0082766	1	.0029954	.0160749
Minority	.0000224	1	.	.0014683
Education	.0054375	1	.0013992	.0120384
Employed	.0003294	1	.	.0029678
Corona_care	.0065855	1	.0020117	.0137033
Insurance_Public	.0005918	1	.	.0036777
Insurance_Private	.000327	1	.	.0029607
SES_before	.0024937	1	.0001802	.0074144
SES_change	.0005812	1	.	.0036513
Health_self	.0003214	1	.	.0029438
Health_other	.0000557	1	.	.0018344
Conservative_01	.0000133	1	.	.0012785

Conservative_other	.0007544	1	.	.0040665
GeoCensus_d1	.0015252	1	.	.0056691
GeoCensus_d2	.0021121	1	.0000816	.0067494
GeoCensus_d3	3.50e-06	1	.	.0008066
Current_measures	.0179094	1	.0095877	.0285962
Measures_clear	.0071866	1	.0023516	.014556
MA_Perc_Threat_SC3	.179511	1	.1555443	.2037896
Costs_SC5	.0015529	1	.	.0057221
Deterr_SD_Likely_SC2	.0001581	1	.	.0023805
Deterr_SD_Severe	.0002107	1	.	.0025813

Note: Eta-Squared values for individual model terms are partial.

```
122 .
123 . *3.a.6 Regression with vce(ro)
124 . reg DV_Compliance_SC7 i.wave_d1 i.wave_d2 Age i.Gender_Female i.Minority Education i.Employed i.Corona_care i.Insura
    > rvative_01 i.Conservative_other i.GeoCensus_d1 i.GeoCensus_d2 i.GeoCensus_d3 i.Current_measures Measures_clear MA_Pe
    > vce(ro)
```

Linear regression

Number of obs	=	2,919
F(25, 2893)	=	36.52
Prob > F	=	0.0000
R-squared	=	0.3083
Root MSE	=	1.0824

DV_Compliance_SC7	Coef.	Robust Std. Err.	t	P>|t|	[95% Conf.	Interval]
1.wave_d1	-.1303741	.04767	-2.73	0.006	-.2238447	-.0369035
1.wave_d2	-.2343954	.0506847	-4.62	0.000	-.3337772	-.1350135
Age	.0072007	.0016257	4.43	0.000	.004013	.0103884
1.Gender_Female	.2041719	.0419878	4.86	0.000	.1218428	.2865009
1.Minority	-.0112874	.0447806	-0.25	0.801	-.0990925	.0765176
Education	.058065	.0144669	4.01	0.000	.0296985	.0864315
1.Employed	-.045634	.0479641	-0.95	0.341	-.1396813	.0484132
1.Corona_care	-.3334566	.0789612	-4.22	0.000	-.4882825	-.1786306
1.Insurance_Public	.0841396	.06951	1.21	0.226	-.0521545	.2204337
1.Insurance_Private	.0652998	.0728184	0.90	0.370	-.0774814	.208081
SES_before	.0296033	.0117823	2.51	0.012	.0065008	.0527059
SES_change	.0171519	.0142411	1.20	0.229	-.0107718	.0450756
2.Health_self	-.0471238	.0439725	-1.07	0.284	-.1333444	.0390968
2.Health_other	-.0185089	.0447113	-0.41	0.679	-.1061782	.0691603
1.Conservative_01	-.0087877	.0434422	-0.20	0.840	-.0939684	.076393
1.Conservative_other	.0998301	.0724358	1.38	0.168	-.0422009	.241861
1.GeoCensus_d1	-.1330702	.0644739	-2.06	0.039	-.2594896	-.0066507
1.GeoCensus_d2	-.1356816	.0544624	-2.49	0.013	-.2424706	-.0288926
1.GeoCensus_d3	.0067938	.0626235	0.11	0.914	-.1159973	.129585
Current_measures						
Yes	.4494543	.0751151	5.98	0.000	.3021697	.5967388
Measures_clear	.0570116	.0130068	4.38	0.000	.0315081	.0825152
MA_Perc_Threat_SC3	.3856428	.0191999	20.09	0.000	.347996	.4232896
Costs_SC5	.0295498	.0145442	2.03	0.042	.0010318	.0580678
Deterr_SD_Likely_SC2	-.0089444	.0133225	-0.67	0.502	-.0350669	.0171781
Deterr_SD_Severe	.0097617	.0129295	0.75	0.450	-.0155903	.0351136
_cons	2.292767	.1939067	11.82	0.000	1.912557	2.672976

```
125 .
126 .
127 . *****************
```

```
128 .
129 . *4. Step 4: Add legitimacy
130 .
131 . *4.a.1 Descriptive Statistics
132 . sum DV_Compliance_SC7 i.wave_d1 i.wave_d2 Age i.Gender_Female i.Minority Education i.Employed i.Corona_care i.Insura
    > rvative_01 i.Conservative_other i.GeoCensus_d1 i.GeoCensus_d2 i.GeoCensus_d3 i.Current_measures Measures_clear MA_Pe
    > _SC2 NOO_SC3 NNOO_SC3 OOL_SC12 PJE_SC4 if chris_sample_reqs == 1
```

Variable	Obs	Mean	Std. Dev.	Min	Max
DV_Complia~7	2,919	5.856947	1.295922	1	7
wave_d1					
0	2,919	.6622131	.4730365	0	1
1	2,919	.3377869	.4730365	0	1
wave_d2					
0	2,919	.684481	.4648019	0	1
1	2,919	.315519	.4648019	0	1
Age	2,919	40.22234	13.05444	17	79
Gender_Fem~e					
0	2,919	.4546077	.4980206	0	1
1	2,919	.5453923	.4980206	0	1
Minority					
0	2,919	.6570743	.4747682	0	1
1	2,919	.3429257	.4747682	0	1
Education	2,919	3.857828	1.542854	1	8
Employed					
0	2,919	.3610826	.4803967	0	1
1	2,919	.6389174	.4803967	0	1
Corona_care					
0	2,919	.9122987	.2829084	0	1
1	2,919	.0877013	.2829084	0	1
Insurance_~c					
0	2,919	.4319973	.4954389	0	1
1	2,919	.5680027	.4954389	0	1
Insurance_~e					
0	2,919	.7060637	.4556412	0	1
1	2,919	.2939363	.4556412	0	1
SES_before	2,919	5.970538	2.050048	1	10
SES_change	2,919	-.2915382	1.654423	-9	9
Health_self					
1	2,919	.6605002	.4736207	0	1
2	2,919	.3394998	.4736207	0	1
Health_other					
1	2,919	.4162384	.4930185	0	1
2	2,919	.5837616	.4930185	0	1
Conservat~01					
0	2,919	.5477903	.4977961	0	1
1	2,919	.4522097	.4977961	0	1
Conservati~r					
0	2,919	.8800959	.324905	0	1
1	2,919	.1199041	.324905	0	1
GeoCensus_d1					
0	2,919	.7923947	.4056621	0	1

```
             1 |      2,919    .2076053    .4056621          0          1
               |
  GeoCensus_d2 |
             0 |      2,919    .5721137    .4948571          0          1
             1 |      2,919    .4278863    .4948571          0          1
               |
  GeoCensus_d3 |
             0 |      2,919    .8396711    .3669737          0          1
             1 |      2,919    .1603289    .3669737          0          1
---------------+---------------------------------------------------------
               |
  Current_me~s |
             0 |      2,919    .1342926    .3410248          0          1
           Yes |      2,919    .8657074    .3410248          0          1
               |
  Measures_c~r |      2,919    5.186023    1.727691          1          7
  MA_Perc_Th~3 |      2,919    5.620418    1.506602          1          7
     Costs_SC5 |      2,919     4.18383    1.639894          1          7
---------------+---------------------------------------------------------
  Deterr_SD_~2 |      2,919    3.256252    1.762824          1          7
  Deterr_SD_~e |      2,919    3.830079    1.718316          1          6
  MA_MoralBe~f |      2,919     6.15519     1.29568          1          7
  MA_Authori~2 |      2,919    4.162384    1.890693          1          7
       NOO_SC3 |      2,919    3.902935    .9011894          1          5
---------------+---------------------------------------------------------
      NNOO_SC3 |      2,919    2.954665    .9979227          1          5
      OOL_SC12 |      2,919    4.356172    1.482673          1          7
       PJE_SC4 |      2,919    5.127698    1.613611          1          7

133 .
134 . *4.a.2 Regression
135 . reg DV_Compliance_SC7 i.wave_d1 i.wave_d2 Age i.Gender_Female i.Minority Education i.Employed i.Corona_care i.Insura
    > rvative_01 i.Conservative_other i.GeoCensus_d1 i.GeoCensus_d2 i.GeoCensus_d3 i.Current_measures Measures_clear MA_Pe
    > _SC2 NOO_SC3 NNOO_SC3 OOL_SC12 PJE_SC4 if chris_sample_reqs == 1

      Source |       SS           df       MS      Number of obs   =     2,919
-------------+----------------------------------   F(31, 2887)     =     60.41
       Model |  1928.05243        31  62.1952398   Prob > F        =    0.0000
    Residual |  2972.47807     2,887  1.02960792   R-squared       =    0.3934
-------------+----------------------------------   Adj R-squared   =    0.3869
       Total |   4900.5305     2,918  1.67941415   Root MSE        =    1.0147

------------------------------------------------------------------------------------
 DV_Compliance_SC7 |      Coef.   Std. Err.      t    P>|t|     [95% Conf. Interval]
-------------------+----------------------------------------------------------------
         1.wave_d1 |  -.1004747   .0461668    -2.18   0.030    -.1909979   -.0099514
         1.wave_d2 |  -.1984235   .0471712    -4.21   0.000     -.290916   -.1059309
               Age |   .0058884    .001572     3.75   0.000      .002806    .0089708
   1.Gender_Female |   .1478955   .0392475     3.77   0.000     .0709396    .2248514
        1.Minority |  -.0119121   .0416364    -0.29   0.775    -.0935522     .069728
         Education |    .046387   .0137462     3.37   0.001     .0194336    .0733404
        1.Employed |  -.0158987   .0438911    -0.36   0.717    -.1019598    .0701623
     1.Corona_care |  -.2296211   .0724567    -3.17   0.002    -.3716932    -.087549
1.Insurance_Public |   .0719235   .0602993     1.19   0.233    -.0463105    .1901576
1.Insurance_Private|   .0638583   .0629927     1.01   0.311     -.059657    .1873736
        SES_before |   .0189879   .0106236     1.79   0.074    -.0018427    .0398185
        SES_change |   .0076474   .0125418     0.61   0.542    -.0169443    .0322392
     2.Health_self |   .0206625   .0461005     0.45   0.654    -.0697307    .1110558
    2.Health_other |  -.0281265   .0435325    -0.65   0.518    -.1134844    .0572314
 1.Conservative_01 |     .04557   .0433498     1.05   0.293    -.0394297    .1305696
1.Conservative_other|  .1312042   .0638924     2.05   0.040     .0059249    .2564835
    1.GeoCensus_d1 |  -.0966241   .0595027    -1.62   0.105    -.2132962    .0200479
    1.GeoCensus_d2 |  -.1336461   .0514938    -2.60   0.009    -.2346144   -.0326778
    1.GeoCensus_d3 |   .0129772   .0634259     0.20   0.838    -.1113874    .1373418
                   |
  Current_measures |
               Yes |   .3320284   .0583814     5.69   0.000     .2175551    .4465018
    Measures_clear |   .0071877   .0126765     0.57   0.571    -.0176682    .0320436
 MA_Perc_Threat_SC3|   .1452602   .0188071     7.72   0.000     .1083836    .1821368
         Costs_SC5 |   .0375573   .0132958     2.82   0.005     .0114871    .0636274
Deterr_SD_Likely_SC2|  .0072218   .0133107     0.54   0.587    -.0188777    .0333213
  Deterr_SD_Severe |  -.0018351   .0118448    -0.15   0.877    -.0250601      .02139
    MA_MoralBelief |   .3698516   .0211068    17.52   0.000     .3284656    .4112375
```

MA_Authority_SC2	-.0075125	.0124893	-0.60	0.548	-.0320013	.0169763
NOO_SC3	.1105221	.027182	4.07	0.000	.0572239	.1638202
NNOO_SC3	.007539	.0230482	0.33	0.744	-.0376535	.0527315
OOL_SC12	.0550423	.0148587	3.70	0.000	.0259075	.084177
PJE_SC4	.0041027	.0135231	0.30	0.762	-.0224132	.0306186
_cons	1.094442	.194686	5.62	0.000	.7127039	1.476179

```
. estimates store model_4

.
. *4.a.3 Check hettest: Run this right after your regression to apply the Breusch-Pagan / Cook-Weisberg test for heter
. *if significant, then you need to run the regression with vce(ro) at the end
. estat hettest

Breusch-Pagan / Cook-Weisberg test for heteroskedasticity
         Ho: Constant variance
         Variables: fitted values of DV_Compliance_SC7

         chi2(1)      =   360.27
         Prob > chi2  =   0.0000

.
. *4.a.4. check vif, to check for for multicollinearity (VIFs >10 are problematic)
. vif
```

Variable	VIF	1/VIF
1.wave_d1	1.35	0.739839
1.wave_d2	1.36	0.734003
Age	1.19	0.837820
1.Gender_F~e	1.08	0.923567
1.Minority	1.11	0.902977
Education	1.27	0.784459
1.Employed	1.26	0.793658
1.Corona_c~e	1.19	0.839726
1.Insuranc~c	2.53	0.395350
1.Insuran~te	2.33	0.428311
SES_before	1.34	0.743904
SES_change	1.22	0.819547
2.Health_s~f	1.35	0.740140
2.Health_o~r	1.31	0.766008
1.Conserv~01	1.32	0.757722
1.Conserva~r	1.22	0.818796
1.GeoCensu~1	1.65	0.605598
1.GeoCensu~2	1.84	0.543397
1.GeoCensu~3	1.54	0.651304
1.Current_~s	1.12	0.890156
Measures_c~r	1.36	0.735624
MA_Perc_Th~3	2.28	0.439488
Costs_SC5	1.35	0.742212
Deterr_SD_~2	1.56	0.640863
Deterr_SD_~e	1.17	0.851778
MA_MoralBe~f	2.12	0.471787
MA_Authori~2	1.58	0.632806
NOO_SC3	1.70	0.588018
NNOO_SC3	1.50	0.666992
OOL_SC12	1.38	0.727000
PJE_SC4	1.35	0.741030
Mean VIF	1.48	

```
144 .
145 . *4.a.5. Effect size
146 . estat esize
```

Effect sizes for linear models

Source	Eta-Squared	df	[95% Conf.	Interval]
Model	.3934375	31	.3615286	.4116176
wave_d1	.0016379	1	.	.005889
wave_d2	.0060916	1	.0017386	.0130019
Age	.0048363	1	.0010993	.0111509
Gender_Female	.0048945	1	.0011272	.0112384
Minority	.0000284	1	.	.0015609
Education	.0039289	1	.00069	.0097599
Employed	.0000454	1	.	.0017501
Corona_care	.0034667	1	.000504	.0090288
Insurance_Public	.0004926	1	.	.0034288
Insurance_Private	.0003558	1	.	.0030506
SES_before	.0011053	1	.	.0048381
SES_change	.0001288	1	.	.0022575
Health_self	.0000696	1	.	.0019375
Health_other	.0001446	1	.	.0023273
Conservative_01	.0003826	1	.	.003128
Conservative_other	.0014585	1	.	.0055462
GeoCensus_d1	.0009125	1	.	.0044268
GeoCensus_d2	.0023278	1	.0001342	.0071345
GeoCensus_d3	.0000145	1	.	.0013105
Current_measures	.0110794	1	.0047705	.019866
Measures_clear	.0001114	1	.	.0021749
MA_Perc_Threat_SC3	.0202452	1	.0113264	.0314977
Costs_SC5	.0027562	1	.0002567	.0078657
Deterr_SD_Likely_SC2	.000102	1	.	.0021271
Deterr_SD_Severe	8.31e-06	1	.	.0011112
MA_MoralBelief	.096132	1	.0768672	.1166571
MA_Authority_SC2	.0001253	1	.	.0022416
NOO_SC3	.0056939	1	.0015283	.0124231
NNOO_SC3	.0000371	1	.	.0016665
OOL_SC12	.0047307	1	.001049	.0109916
PJE_SC4	.0000319	1	.	.0016066

Note: Eta-Squared values for individual model terms are partial.

```
147 .
148 . *4.a.6 Regression with vce(ro)
149 . reg DV_Compliance_SC7 i.wave_d1 i.wave_d2 Age i.Gender_Female i.Minority Education i.Employed i.Corona_care i.Insura
    > rvative_01 i.Conservative_other i.GeoCensus_d1 i.GeoCensus_d2 i.GeoCensus_d3 i.Current_measures Measures_clear MA_Pe
    > _SC2 NOO_SC3 NNOO_SC3 OOL_SC12 PJE_SC4 if chris_sample_reqs == 1, vce(ro)
```

Linear regression

Number of obs	=	2,919
F(31, 2887)	=	43.79
Prob > F	=	0.0000
R-squared	=	0.3934
Root MSE	=	1.0147

DV_Compliance_SC7	Coef.	Robust Std. Err.	t	P>|t|	[95% Conf.	Interval]
1.wave_d1	-.1004747	.0446969	-2.25	0.025	-.1881157	-.0128336
1.wave_d2	-.1984235	.0472718	-4.20	0.000	-.2911134	-.1057335
Age	.0058884	.0015769	3.73	0.000	.0027964	.0089804
1.Gender_Female	.1478955	.0395603	3.74	0.000	.0703263	.2254647
1.Minority	-.0119121	.0425278	-0.28	0.779	-.0953001	.0714759
Education	.046387	.0133987	3.46	0.001	.0201151	.072659
1.Employed	-.0158987	.0451103	-0.35	0.725	-.1043504	.0725529
1.Corona_care	-.2296211	.0802928	-2.86	0.004	-.3870582	-.072184
1.Insurance_Public	.0719235	.0658474	1.09	0.275	-.0571892	.2010363
1.Insurance_Private	.0638583	.0687333	0.93	0.353	-.070913	.1986295
SES_before	.0189879	.0113804	1.67	0.095	-.0033266	.0413024
SES_change	.0076474	.0136357	0.56	0.575	-.0190892	.0343841
2.Health_self	.0206625	.0418824	0.49	0.622	-.0614599	.1027849

2.Health_other	-.0281265	.0423049	-0.66	0.506	-.1110774	.0548244
1.Conservative_01	.04557	.044295	1.03	0.304	-.041283	.132423
1.Conservative_other	.1312042	.0684315	1.92	0.055	-.0029754	.2653838
1.GeoCensus_d1	-.0966241	.0591066	-1.63	0.102	-.2125196	.0192714
1.GeoCensus_d2	-.1336461	.0503618	-2.65	0.008	-.2323949	-.0348973
1.GeoCensus_d3	.0129772	.0585979	0.22	0.825	-.1019208	.1278753
Current_measures						
Yes	.3320284	.0696236	4.77	0.000	.1955114	.4685454
Measures_clear	.0071877	.013092	0.55	0.583	-.0184828	.0328583
MA_Perc_Threat_SC3	.1452602	.0218933	6.63	0.000	.1023322	.1881883
Costs_SC5	.0375573	.0138979	2.70	0.007	.0103064	.0648082
Deterr_SD_Likely_SC2	.0072218	.0135002	0.53	0.593	-.0192493	.0336929
Deterr_SD_Severe	-.0018351	.0123066	-0.15	0.881	-.0259658	.0222956
MA_MoralBelief	.3698516	.0281589	13.13	0.000	.3146381	.4250651
MA_Authority_SC2	-.0075125	.0123239	-0.61	0.542	-.031677	.016652
NOO_SC3	.1105221	.0292244	3.78	0.000	.0532193	.1678249
NNOO_SC3	.007539	.0228549	0.33	0.742	-.0372746	.0523526
OOL_SC12	.0550423	.0153698	3.58	0.000	.0249054	.0851791
PJE_SC4	.0041027	.0138221	0.30	0.767	-.0229995	.0312048
_cons	1.094442	.2086266	5.25	0.000	.6853695	1.503514

```
150 .
151 .
152 . *****************
153 .
154 . *5. Step 5: Add personal factors
155 .
156 . *5.a.1 Descriptive Statistics
157 . sum DV_Compliance_SC7 i.wave_d1 i.wave_d2 Age i.Gender_Female i.Minority Education i.Employed i.Corona_care i.Insura
    > rvative_01 i.Conservative_other i.GeoCensus_d1 i.GeoCensus_d2 i.GeoCensus_d3 i.Current_measures Measures_clear MA_Pe
    > _SC2 NOO_SC3 NNOO_SC3 OOL_SC12 PJE_SC4 Trust_Science_SC4 Trust_in_media Impulsivity_SC4 NegEmo_SC6 if chris_sample_r
```

Variable	Obs	Mean	Std. Dev.	Min	Max
DV_Complia~7	2,919	5.856947	1.295922	1	7
wave_d1					
0	2,919	.6622131	.4730365	0	1
1	2,919	.3377869	.4730365	0	1
wave_d2					
0	2,919	.684481	.4648019	0	1
1	2,919	.315519	.4648019	0	1
Age	2,919	40.22234	13.05444	17	79
Gender_Fem~e					
0	2,919	.4546077	.4980206	0	1
1	2,919	.5453923	.4980206	0	1
Minority					
0	2,919	.6570743	.4747682	0	1
1	2,919	.3429257	.4747682	0	1
Education	2,919	3.857828	1.542854	1	8
Employed					
0	2,919	.3610826	.4803967	0	1
1	2,919	.6389174	.4803967	0	1
Corona_care					
0	2,919	.9122987	.2829084	0	1
1	2,919	.0877013	.2829084	0	1
Insurance_~c					
0	2,919	.4319973	.4954389	0	1
1	2,919	.5680027	.4954389	0	1
Insurance_~e					

0	2,919	.7060637	.4556412	0	1
1	2,919	.2939363	.4556412	0	1
SES_before	2,919	5.970538	2.050048	1	10
SES_change	2,919	-.2915382	1.654423	-9	9
Health_self					
1	2,919	.6605002	.4736207	0	1
2	2,919	.3394998	.4736207	0	1
Health_other					
1	2,919	.4162384	.4930185	0	1
2	2,919	.5837616	.4930185	0	1
Conservat~01					
0	2,919	.5477903	.4977961	0	1
1	2,919	.4522097	.4977961	0	1
Conservati~r					
0	2,919	.8800959	.324905	0	1
1	2,919	.1199041	.324905	0	1
GeoCensus_d1					
0	2,919	.7923947	.4056621	0	1
1	2,919	.2076053	.4056621	0	1
GeoCensus_d2					
0	2,919	.5721137	.4948571	0	1
1	2,919	.4278863	.4948571	0	1
GeoCensus_d3					
0	2,919	.8396711	.3669737	0	1
1	2,919	.1603289	.3669737	0	1
Current_me~s					
0	2,919	.1342926	.3410248	0	1
Yes	2,919	.8657074	.3410248	0	1
Measures_c~r	2,919	5.186023	1.727691	1	7
MA_Perc_Th~3	2,919	5.620418	1.506602	1	7
Costs_SC5	2,919	4.18383	1.639894	1	7
Deterr_SD_~2	2,919	3.256252	1.762824	1	7
Deterr_SD_~e	2,919	3.830079	1.718316	1	6
MA_MoralBe~f	2,919	6.15519	1.29568	1	7
MA_Authori~2	2,919	4.162384	1.890693	1	7
NOO_SC3	2,919	3.902935	.9011894	1	5
NNOO_SC3	2,919	2.954665	.9979227	1	5
OOL_SC12	2,919	4.356172	1.482673	1	7
PJE_SC4	2,919	5.127698	1.613611	1	7
Trust_Scie~4	2,919	3.85149	.9866086	1	5
Trust_in_m~a	2,919	2.896197	1.311564	1	5
Impulsivi~C4	2,919	2.457263	1.124088	1	5
NegEmo_SC6	2,919	4.584561	1.56834	1	7

158 .

```
159 . *5.a.2 Regression
160 . reg DV_Compliance_SC7 i.wave_d1 i.wave_d2 Age i.Gender_Female i.Minority Education i.Employed i.Corona_care i.Insura
    > rvative_01 i.Conservative_other i.GeoCensus_d1 i.GeoCensus_d2 i.GeoCensus_d3 i.Current_measures Measures_clear MA_Pe
    > _SC2 NOO_SC3 NNOO_SC3 OOL_SC12 PJE_SC4 Trust_Science_SC4 Trust_in_media Impulsivity_SC4 NegEmo_SC6 if chris_sample_r

      Source |       SS           df       MS      Number of obs   =     2,919
-------------+----------------------------------   F(35, 2883)     =     55.60
       Model |  1974.89491        35  56.4255689   Prob > F        =    0.0000
    Residual |  2925.63559     2,883  1.01478862   R-squared       =    0.4030
-------------+----------------------------------   Adj R-squared   =    0.3957
       Total |   4900.5305     2,918  1.67941415   Root MSE        =    1.0074

-------------------------------------------------------------------------------------
   DV_Compliance_SC7 |      Coef.   Std. Err.      t    P>|t|     [95% Conf. Interval]
---------------------+---------------------------------------------------------------
           1.wave_d1 |  -.0903378   .0458707    -1.97   0.049    -.1802804   -.0003952
           1.wave_d2 |   -.190852   .0468668    -4.07   0.000    -.2827479   -.0989562
                 Age |   .0054045   .0016017     3.37   0.001     .0022639    .0085451
     1.Gender_Female |   .1332199   .0394216     3.38   0.001     .0559225    .2105174
          1.Minority |  -.0009368   .0421226    -0.02   0.982    -.0835302    .0816566
           Education |   .0433359   .0136776     3.17   0.002      .016517    .0701548
          1.Employed |  -.0185973   .0436463    -0.43   0.670    -.1041784    .0669839
       1.Corona_care |  -.1806292   .0725666    -2.49   0.013    -.3229169   -.0383414
  1.Insurance_Public |   .0689312   .0599186     1.15   0.250    -.0485565    .1864188
 1.Insurance_Private |   .0733063   .0626132     1.17   0.242    -.0494648    .1960774
          SES_before |   .0198299   .0106276     1.87   0.062    -.0010085    .0406682
          SES_change |   .0076457   .0125148     0.61   0.541    -.0168932    .0321846
       2.Health_self |   .0306101   .0458204     0.67   0.504     -.059234    .1204541
      2.Health_other |  -.0454986    .043523    -1.05   0.296    -.1308379    .0398407
   1.Conservative_01 |   .0582815   .0449542     1.30   0.195    -.0298642    .1464271
1.Conservative_other |   .1530482    .064488     2.37   0.018      .026601    .2794954
      1.GeoCensus_d1 |  -.0854074   .0591158    -1.44   0.149    -.2013209     .030506
      1.GeoCensus_d2 |  -.1194536   .0512382    -2.33   0.020    -.2199209   -.0189864
      1.GeoCensus_d3 |   .0185366   .0630089     0.29   0.769    -.1050105    .1420837
                     |
    Current_measures |
                Yes  |   .3128024   .0580388     5.39   0.000     .1990006    .4266042
      Measures_clear |   .0031099   .0128009     0.24   0.808      -.02199    .0282097
    MA_Perc_Threat_SC3 |   .1392348   .0191119     7.29   0.000     .1017604    .1767091
           Costs_SC5 |   .0322592   .0136577     2.36   0.018     .0054793     .059039
  Deterr_SD_Likely_SC2 |   .0174867   .0133924     1.31   0.192     -.008773    .0437464
    Deterr_SD_Severe |   -.000135   .0118406    -0.01   0.991     -.023352    .0230819
      MA_MoralBelief |   .3602649   .0211585    17.03   0.000     .3187776    .4017522
     MA_Authority_SC2 |  -.0001475    .012471    -0.01   0.991    -.0246004    .0243054
             NOO_SC3 |   .1016395   .0272666     3.73   0.000     .0481755    .1551034
            NNOO_SC3 |   .0222116   .0232834     0.95   0.340    -.0234422    .0678655
            OOL_SC12 |   .0235922   .0157748     1.50   0.135    -.0073388    .0545232
             PJE_SC4 |   .0037502   .0134853     0.28   0.781    -.0226916    .0301921
   Trust_Science_SC4 |   .0767429   .0244945     3.13   0.002     .0287144    .1247715
      Trust_in_media |  -.0190764   .0175194    -1.09   0.276    -.0534282    .0152754
     Impulsivity_SC4 |  -.1216553   .0210854    -5.77   0.000    -.1629994   -.0803112
          NegEmo_SC6 |   .0265849   .0140743     1.89   0.059    -.0010117    .0541815
               _cons |   1.253626   .2105591     5.95   0.000      .840764    1.666487
-------------------------------------------------------------------------------------

161 . estimates store model_5

162 .
163 . *5.a.3 Check hettest: Run this right after your regression to apply the Breusch-Pagan / Cook-Weisberg test for heter
164 . *if significant, then you need to run the regression with vce(ro) at the end
165 . estat hettest

    Breusch-Pagan / Cook-Weisberg test for heteroskedasticity
             Ho: Constant variance
             Variables: fitted values of DV_Compliance_SC7

             chi2(1)      =   339.15
             Prob > chi2  =   0.0000
```

```
166 .
167 . *5.a.4. check vif, to check for for multicollinearity (VIFs >10 are problematic)
168 . vif
```

Variable	VIF	1/VIF
1.wave_d1	1.35	0.738637
1.wave_d2	1.36	0.732865
Age	1.26	0.795453
1.Gender_F~e	1.11	0.902249
1.Minority	1.15	0.869555
Education	1.28	0.780946
1.Employed	1.26	0.791034
1.Corona_c~e	1.21	0.825134
1.Insuranc~c	2.53	0.394627
1.Insuran~te	2.34	0.427280
SES_before	1.36	0.732647
SES_change	1.23	0.811236
2.Health_s~f	1.35	0.738434
2.Health_o~r	1.32	0.755312
1.Conserv~01	1.44	0.694459
1.Conserva~r	1.26	0.792173
1.GeoCensu~1	1.65	0.604720
1.GeoCensu~2	1.85	0.540932
1.GeoCensu~3	1.54	0.650453
1.Current_~s	1.13	0.887730
Measures_c~r	1.41	0.711013
MA_Perc_Th~3	2.38	0.419455
Costs_SC5	1.44	0.693272
Deterr_SD_~2	1.60	0.623957
Deterr_SD_~e	1.19	0.840106
MA_MoralBe~f	2.16	0.462727
MA_Authori~2	1.60	0.625530
NOO_SC3	1.74	0.575966
NNOO_SC3	1.55	0.644173
OOL_SC12	1.57	0.635729
PJE_SC4	1.36	0.734463
Trust_Scie~4	1.68	0.595473
Trust_in_m~a	1.52	0.658679
Impulsivi~C4	1.62	0.619048
NegEmo_SC6	1.40	0.713771
Mean VIF	1.52	

```
169 .
170 . *5.a.5. Effect size
171 . estat esize
```

Effect sizes for linear models

Source	Eta-Squared	df	[95% Conf.	Interval]
Model	.4029961	35	.3704557	.4202962
wave_d1	.0013435	1	.	.0053246
wave_d2	.0057191	1	.0015395	.0124655
Age	.0039336	1	.0006906	.0097722
Gender_Female	.0039456	1	.0006956	.0097909
Minority	1.72e-07	1	.	.
Education	.0034699	1	.0005042	.0090388
Employed	.000063	1	.	.0018937
Corona_care	.0021445	1	.0000879	.006817
Insurance_Public	.0004588	1	.	.003342
Insurance_Private	.0004752	1	.	.0033859
SES_before	.0012062	1	.	.0050491
SES_change	.0001294	1	.	.0022629
Health_self	.0001548	1	.	.0023727
Health_other	.0003789	1	.	.0031203
Conservative_01	.0005827	1	.	.0036625
Conservative_other	.0019499	1	.0000434	.0064686
GeoCensus_d1	.0007235	1	.	.0040025
GeoCensus_d2	.0018817	1	.0000288	.0063447
GeoCensus_d3	.00003	1	.	.0015847

Current_measures	.0099748	1	.0040477	.0184006
Measures_clear	.0000205	1	.	.0014388
MA_Perc_Threat_SC3	.0180768	1	.0096992	.0288251
Costs_SC5	.0019314	1	.0000394	.0064351
Deterr_SD_Likely_SC2	.000591	1	.	.0036833
Deterr_SD_Severe	4.51e-08	1	.	.
MA_MoralBelief	.0913725	1	.0724987	.1115696
MA_Authority_SC2	4.85e-08	1	.	.
NOO_SC3	.0047966	1	.0010786	.0110962
NNOO_SC3	.0003156	1	.	.0029328
OOL_SC12	.0007752	1	.	.0041224
PJE_SC4	.0000268	1	.	.0015412
Trust_Science_SC4	.0033933	1	.0004751	.0089157
Trust_in_media	.0004111	1	.	.0032112
Impulsivity_SC4	.0114147	1	.0049895	.0203157
NegEmo_SC6	.0012361	1	.	.0051097

Note: Eta-Squared values for individual model terms are partial.

```
172 .
173 . *5.a.6 Regression with vce(ro)
174 . reg DV_Compliance_SC7 i.wave_d1 i.wave_d2 Age i.Gender_Female i.Minority Education i.Employed i.Corona_care i.Insura
    > rvative_01 i.Conservative_other i.GeoCensus_d1 i.GeoCensus_d2 i.GeoCensus_d3 i.Current_measures Measures_clear MA_Pe
    > _SC2 NOO_SC3 NNOO_SC3 OOL_SC12 PJE_SC4 Trust_Science_SC4 Trust_in_media Impulsivity_SC4 NegEmo_SC6 if chris_sample_r
```

Linear regression

Number of obs	=	2,919
F(35, 2883)	=	42.79
Prob > F	=	0.0000
R-squared	=	0.4030
Root MSE	=	1.0074

DV_Compliance_SC7	Coef.	Robust Std. Err.	t	P>|t|	[95% Conf.	Interval]
1.wave_d1	-.0903378	.0444647	-2.03	0.042	-.1775236	-.0031519
1.wave_d2	-.190852	.0468894	-4.07	0.000	-.2827921	-.098912
Age	.0054045	.0015966	3.39	0.001	.002274	.008535
1.Gender_Female	.1332199	.0397764	3.35	0.001	.0552269	.211213
1.Minority	-.0009368	.0431078	-0.02	0.983	-.0854621	.0835885
Education	.0433359	.0133833	3.24	0.001	.0170941	.0695777
1.Employed	-.0185973	.0449264	-0.41	0.679	-.1066883	.0694938
1.Corona_care	-.1806292	.0806832	-2.24	0.025	-.3388317	-.0224267
1.Insurance_Public	.0689312	.0654636	1.05	0.292	-.059429	.1972913
1.Insurance_Private	.0733063	.0684309	1.07	0.284	-.0608722	.2074848
SES_before	.0198299	.0113164	1.75	0.080	-.0023592	.0420189
SES_change	.0076457	.0137059	0.56	0.577	-.0192287	.03452
2.Health_self	.0306101	.0417601	0.73	0.464	-.0512727	.1124928
2.Health_other	-.0454986	.0424648	-1.07	0.284	-.1287631	.0377658
1.Conservative_01	.0582815	.045706	1.28	0.202	-.0313382	.1479011
1.Conservative_other	.1530482	.0697101	2.20	0.028	.0163615	.2897349
1.GeoCensus_d1	-.0854074	.0589577	-1.45	0.148	-.2010108	.030196
1.GeoCensus_d2	-.1194536	.0500508	-2.39	0.017	-.2175926	-.0213147
1.GeoCensus_d3	.0185366	.0580817	0.32	0.750	-.0953492	.1324224
Current_measures						
Yes	.3128024	.0690018	4.53	0.000	.1775045	.4481003
Measures_clear	.0031099	.0132171	0.24	0.814	-.022806	.0290257
MA_Perc_Threat_SC3	.1392348	.0223001	6.24	0.000	.0955091	.1829605
Costs_SC5	.0322592	.014131	2.28	0.023	.0045512	.0599671
Deterr_SD_Likely_SC2	.0174867	.0136111	1.28	0.199	-.0092017	.0441751
Deterr_SD_Severe	-.000135	.0124711	-0.01	0.991	-.0245882	.0243182
MA_MoralBelief	.3602649	.0280059	12.86	0.000	.3053513	.4151786
MA_Authority_SC2	-.0001475	.012141	-0.01	0.990	-.0239534	.0236583
NOO_SC3	.1016395	.0288044	3.53	0.000	.0451602	.1581187
NNOO_SC3	.0222116	.0229331	0.97	0.333	-.0227552	.0671785
OOL_SC12	.0235922	.0164071	1.44	0.151	-.0085787	.055763
PJE_SC4	.0037502	.0136956	0.27	0.784	-.023104	.0306045
Trust_Science_SC4	.0767429	.0267404	2.87	0.004	.0243106	.1291753
Trust_in_media	-.0190764	.0167195	-1.14	0.254	-.0518598	.013707
Impulsivity_SC4	-.1216553	.0208654	-5.83	0.000	-.1625679	-.0807426
NegEmo_SC6	.0265849	.0144658	1.84	0.066	-.0017795	.0549493
_cons	1.253626	.2224696	5.64	0.000	.8174101	1.689841

```
175 .
176 .
177 . *****************
178 .
179 . *6. Step 6: Add social environment
180 .
181 . *6.a.1 Descriptive Statistics
182 . sum DV_Compliance_SC7 i.wave_d1 i.wave_d2 Age i.Gender_Female i.Minority Education i.Employed i.Corona_care i.Insura
    > rvative_01 i.Conservative_other i.GeoCensus_d1 i.GeoCensus_d2 i.GeoCensus_d3 i.Current_measures Measures_clear MA_Pe
    > _SC2 NOO_SC3 NNOO_SC3 OOL_SC12 PJE_SC4 Trust_Science_SC4 Trust_in_media Impulsivity_SC4 NegEmo_SC6 SN_SC7 if chris_s
```

Variable	Obs	Mean	Std. Dev.	Min	Max
DV_Complia~7	2,919	5.856947	1.295922	1	7
wave_d1					
0	2,919	.6622131	.4730365	0	1
1	2,919	.3377869	.4730365	0	1
wave_d2					
0	2,919	.684481	.4648019	0	1
1	2,919	.315519	.4648019	0	1
Age	2,919	40.22234	13.05444	17	79
Gender_Fem~e					
0	2,919	.4546077	.4980206	0	1
1	2,919	.5453923	.4980206	0	1
Minority					
0	2,919	.6570743	.4747682	0	1
1	2,919	.3429257	.4747682	0	1
Education	2,919	3.857828	1.542854	1	8
Employed					
0	2,919	.3610826	.4803967	0	1
1	2,919	.6389174	.4803967	0	1
Corona_care					
0	2,919	.9122987	.2829084	0	1
1	2,919	.0877013	.2829084	0	1
Insurance_~c					
0	2,919	.4319973	.4954389	0	1
1	2,919	.5680027	.4954389	0	1
Insurance_~e					
0	2,919	.7060637	.4556412	0	1
1	2,919	.2939363	.4556412	0	1
SES_before	2,919	5.970538	2.050048	1	10
SES_change	2,919	-.2915382	1.654423	-9	9
Health_self					
1	2,919	.6605002	.4736207	0	1
2	2,919	.3394998	.4736207	0	1
Health_other					
1	2,919	.4162384	.4930185	0	1
2	2,919	.5837616	.4930185	0	1
Conservat~01					
0	2,919	.5477903	.4977961	0	1
1	2,919	.4522097	.4977961	0	1
Conservati~r					
0	2,919	.8800959	.324905	0	1

1	2,919	.1199041	.324905	0	1
GeoCensus_d1					
0	2,919	.7923947	.4056621	0	1
1	2,919	.2076053	.4056621	0	1
GeoCensus_d2					
0	2,919	.5721137	.4948571	0	1
1	2,919	.4278863	.4948571	0	1
GeoCensus_d3					
0	2,919	.8396711	.3669737	0	1
1	2,919	.1603289	.3669737	0	1
Current_me~s					
0	2,919	.1342926	.3410248	0	1
Yes	2,919	.8657074	.3410248	0	1
Measures_c~r	2,919	5.186023	1.727691	1	7
MA_Perc_Th~3	2,919	5.620418	1.506602	1	7
Costs_SC5	2,919	4.18383	1.639894	1	7
Deterr_SD_~2	2,919	3.256252	1.762824	1	7
Deterr_SD_~e	2,919	3.830079	1.718316	1	6
MA_MoralBe~f	2,919	6.15519	1.29568	1	7
MA_Authori~2	2,919	4.162384	1.890693	1	7
NOO_SC3	2,919	3.902935	.9011894	1	5
NNOO_SC3	2,919	2.954665	.9979227	1	5
OOL_SC12	2,919	4.356172	1.482673	1	7
PJE_SC4	2,919	5.127698	1.613611	1	7
Trust_Scie~4	2,919	3.85149	.9866086	1	5
Trust_in_m~a	2,919	2.896197	1.311564	1	5
Impulsivi~C4	2,919	2.457263	1.124088	1	5
NegEmo_SC6	2,919	4.584561	1.56834	1	7
SN_SC7	2,919	5.25498	1.396018	1	7

```
183 .
184 . *6.a.2 Regression
185 . reg DV_Compliance_SC7 i.wave_d1 i.wave_d2 Age i.Gender_Female i.Minority Education i.Employed i.Corona_care i.Insura
    > rvative_01 i.Conservative_other i.GeoCensus_d1 i.GeoCensus_d2 i.GeoCensus_d3 i.Current_measures Measures_clear MA_Pe
    > _SC2 NOO_SC3 NNOO_SC3 OOL_SC12 PJE_SC4 Trust_Science_SC4 Trust_in_media Impulsivity_SC4 NegEmo_SC6 SN_SC7 if chris_s
```

Source	SS	df	MS
Model	2076.92725	36	57.6924236
Residual	2823.60325	2,882	.979737422
Total	4900.5305	2,918	1.67941415

Number of obs	=	2,919
F(36, 2882)	=	58.89
Prob > F	=	0.0000
R-squared	=	0.4238
Adj R-squared	=	0.4166
Root MSE	=	.98982

DV_Compliance_SC7	Coef.	Std. Err.	t	P>|t|	[95% Conf.	Interval]
1.wave_d1	-.0632841	.0451494	-1.40	0.161	-.1518124	.0252443
1.wave_d2	-.1532637	.0461974	-3.32	0.001	-.2438469	-.0626805
Age	.0043437	.0015772	2.75	0.006	.0012511	.0074363
1.Gender_Female	.1300492	.0387361	3.36	0.001	.054096	.2060024
1.Minority	-.0082106	.0413949	-0.20	0.843	-.0893771	.072956
Education	.0391633	.0134455	2.91	0.004	.0127995	.0655272
1.Employed	-.0226208	.0428877	-0.53	0.598	-.1067145	.0614729
1.Corona_care	-.1595952	.0713322	-2.24	0.025	-.2994624	-.0197279
1.Insurance_Public	.0668858	.058875	1.14	0.256	-.0485556	.1823273
1.Insurance_Private	.0834548	.0615304	1.36	0.175	-.0371931	.2041028
SES_before	.0142649	.0104566	1.36	0.173	-.0062384	.0347681
SES_change	.00437	.012301	0.36	0.722	-.0197496	.0284896
2.Health_self	.0313729	.0450222	0.70	0.486	-.056906	.1196518
2.Health_other	-.0315578	.0427865	-0.74	0.461	-.1154531	.0523375
1.Conservative_01	.0505884	.0441775	1.15	0.252	-.0360342	.137211
1.Conservative_other	.1518583	.0633646	2.40	0.017	.0276138	.2761028
1.GeoCensus_d1	-.067419	.0581126	-1.16	0.246	-.1813654	.0465275
1.GeoCensus_d2	-.0928101	.0504132	-1.84	0.066	-.1916597	.0060395

1.GeoCensus_d3	.0225062	.0619124	0.36	0.716	-.0988909	.1439034
Current_measures						
Yes	.2793733	.0571217	4.89	0.000	.1673698	.3913768
Measures_clear	-.0031614	.0125929	-0.25	0.802	-.0278533	.0215306
MA_Perc_Threat_SC3	.1441801	.0187852	7.68	0.000	.1073464	.1810139
Costs_SC5	.0318496	.0134198	2.37	0.018	.0055362	.058163
Deterr_SD_Likely_SC2	.0070683	.0131986	0.54	0.592	-.0188115	.032948
Deterr_SD_Severe	-.0032394	.0116383	-0.28	0.781	-.0260596	.0195809
MA_MoralBelief	.3384828	.0208992	16.20	0.000	.297504	.3794616
MA_Authority_SC2	-.0145861	.0123351	-1.18	0.237	-.0387726	.0096004
NOO_SC3	.0862004	.0268342	3.21	0.001	.0335842	.1388167
NNOO_SC3	-.0005102	.0229859	-0.02	0.982	-.0455807	.0445602
OOL_SC12	.0201419	.0155037	1.30	0.194	-.0102575	.0505413
PJE_SC4	-.0073118	.0132947	-0.55	0.582	-.0333798	.0187562
Trust_Science_SC4	.0649723	.0240954	2.70	0.007	.0177263	.1122183
Trust_in_media	-.0262524	.0172285	-1.52	0.128	-.0600338	.0075291
Impulsivity_SC4	-.128795	.0207299	-6.21	0.000	-.1694419	-.088148
NegEmo_SC6	.0229652	.0138336	1.66	0.097	-.0041595	.05009
SN_SC7	.1517372	.0148689	10.21	0.000	.1225825	.1808918
_cons	1.083639	.2075602	5.22	0.000	.6766573	1.49062

```
186 . estimates store model_6

187 .
188 . *6.a.3 Check hettest: Run this right after your regression to apply the Breusch-Pagan / Cook-Weisberg test for heter
189 . *if significant, then you need to run the regression with vce(ro) at the end
190 . estat hettest

    Breusch-Pagan / Cook-Weisberg test for heteroskedasticity
             Ho: Constant variance
             Variables: fitted values of DV_Compliance_SC7

             chi2(1)      =   313.81
             Prob > chi2  =   0.0000

191 .
192 . *6.a.4. check vif, to check for for multicollinearity (VIFs >10 are problematic)
193 . vif
```

Variable	VIF	1/VIF
1.wave_d1	1.36	0.736090
1.wave_d2	1.37	0.728206
Age	1.26	0.791998
1.Gender_F~e	1.11	0.902191
1.Minority	1.15	0.869297
Education	1.28	0.780224
1.Employed	1.26	0.790967
1.Corona_c~e	1.21	0.824445
1.Insuranc~c	2.53	0.394622
1.Insuran~te	2.34	0.427169
SES_before	1.37	0.730655
SES_change	1.23	0.810684
2.Health_s~f	1.35	0.738432
2.Health_o~r	1.33	0.754542
1.Conserv~01	1.44	0.694257
1.Conserva~r	1.26	0.792170
1.GeoCensu~1	1.66	0.604164
1.GeoCensu~2	1.85	0.539481
1.GeoCensu~3	1.54	0.650428
1.Current_~s	1.13	0.884811
Measures_c~r	1.41	0.709319
MA_Perc_Th~3	2.39	0.419176
Costs_SC5	1.44	0.693266
Deterr_SD_~2	1.61	0.620224
Deterr_SD_~e	1.19	0.839532
MA_MoralBe~f	2.18	0.457901
MA_Authori~2	1.62	0.617301
NOO_SC3	1.74	0.574135
NNOO_SC3	1.57	0.638129
OOL_SC12	1.57	0.635427
PJE_SC4	1.37	0.729580

Trust_Scie~4	1.68	0.594109
Trust_in_m~a	1.52	0.657581
Impulsivi~C4	1.62	0.618343
NegEmo_SC6	1.40	0.713301
SN_SC7	1.28	0.779267
Mean VIF	1.52	

```
194 .
195 . *6.a.5. Effect size
196 . estat esize
```

Effect sizes for linear models

Source	Eta-Squared	df	[95% Conf.	Interval]
Model	.4238168	36	.3916787	.4407293
wave_d1	.0006812	1	.	.0039035
wave_d2	.0038045	1	.0006366	.0095709
Age	.0026248	1	.0002159	.0076496
Gender_Female	.0038958	1	.0006744	.0097143
Minority	.0000137	1	.	.0012902
Education	.0029352	1	.0003129	.0081695
Employed	.0000965	1	.	.0021011
Corona_care	.0017339	1	.	.0060735
Insurance_Public	.0004476	1	.	.0033125
Insurance_Private	.0006379	1	.	.0037993
SES_before	.0006453	1	.	.0038173
SES_change	.0000438	1	.	.0017369
Health_self	.0001685	1	.	.0024288
Health_other	.0001887	1	.	.0025073
Conservative_01	.0004548	1	.	.0033318
Conservative_other	.001989	1	.0000519	.0065402
GeoCensus_d1	.0004668	1	.	.0033641
GeoCensus_d2	.0011746	1	.	.0049856
GeoCensus_d3	.0000458	1	.	.0017561
Current_measures	.0082316	1	.0029606	.0160299
Measures_clear	.0000219	1	.	.0014639
MA_Perc_Threat_SC3	.0200308	1	.0111579	.0312448
Costs_SC5	.0019506	1	.0000435	.006471
Deterr_SD_Likely_SC2	.0000995	1	.	.002117
Deterr_SD_Severe	.0000269	1	.	.0015424
MA_MoralBelief	.0834238	1	.065267	.1030088
MA_Authority_SC2	.0004849	1	.	.0034124
NOO_SC3	.0035678	1	.0005417	.0091962
NNOO_SC3	1.71e-07	1	.	.
OOL_SC12	.0005853	1	.	.0036698
PJE_SC4	.0001049	1	.	.0021454
Trust_Science_SC4	.0025165	1	.0001846	.0074652
Trust_in_media	.000805	1	.	.0041912
Impulsivity_SC4	.0132169	1	.0062122	.0226696
NegEmo_SC6	.0009553	1	.	.0045243
SN_SC7	.0348753	1	.0229281	.0489365

Note: Eta-Squared values for individual model terms are partial.

```
197 .
198 . *6.a.6 Regression with vce(ro)
199 . reg DV_Compliance_SC7 i.wave_d1 i.wave_d2 Age i.Gender_Female i.Minority Education i.Employed i.Corona_care i.Insura
> rvative_01 i.Conservative_other i.GeoCensus_d1 i.GeoCensus_d2 i.GeoCensus_d3 i.Current_measures Measures_clear MA_Pe
> _SC2 NOO_SC3 NNOO_SC3 OOL_SC12 PJE_SC4 Trust_Science_SC4 Trust_in_media Impulsivity_SC4 NegEmo_SC6 SN_SC7 if chris_s
```

Linear regression

Number of obs	=	2,919
F(36, 2882)	=	48.54
Prob > F	=	0.0000
R-squared	=	0.4238
Root MSE	=	.98982

DV_Compliance_SC7	Coef.	Robust Std. Err.	t	P>|t|	[95% Conf.	Interval]
1.wave_d1	-.0632841	.0439219	-1.44	0.150	-.1494055	.0228374
1.wave_d2	-.1532637	.0460731	-3.33	0.001	-.2436032	-.0629242
Age	.0043437	.0015603	2.78	0.005	.0012842	.0074032
1.Gender_Female	.1300492	.0390161	3.33	0.001	.0535469	.2065514
1.Minority	-.0082106	.042338	-0.19	0.846	-.0912264	.0748053
Education	.0391633	.0131544	2.98	0.003	.0133704	.0649562
1.Employed	-.0226208	.0440715	-0.51	0.608	-.1090356	.063794
1.Corona_care	-.1595952	.0774796	-2.06	0.040	-.3115162	-.0076741
1.Insurance_Public	.0668858	.0646886	1.03	0.301	-.0599548	.1937265
1.Insurance_Private	.0834548	.0677095	1.23	0.218	-.0493091	.2162187
SES_before	.0142649	.011099	1.29	0.199	-.0074979	.0360276
SES_change	.00437	.013553	0.32	0.747	-.0222046	.0309446
2.Health_self	.0313729	.0411181	0.76	0.446	-.049251	.1119969
2.Health_other	-.0315578	.0417346	-0.76	0.450	-.1133905	.0502749
1.Conservative_01	.0505884	.0449646	1.13	0.261	-.0375777	.1387545
1.Conservative_other	.1518583	.0684632	2.22	0.027	.0176165	.2861001
1.GeoCensus_d1	-.067419	.0572395	-1.18	0.239	-.1796534	.0448155
1.GeoCensus_d2	-.0928101	.0487482	-1.90	0.057	-.1883951	.0027748
1.GeoCensus_d3	.0225062	.0570345	0.39	0.693	-.0893264	.1343389
Current_measures						
Yes	.2793733	.0679525	4.11	0.000	.1461329	.4126136
Measures_clear	-.0031614	.0130628	-0.24	0.809	-.0287747	.022452
MA_Perc_Threat_SC3	.1441801	.0220136	6.55	0.000	.1010162	.187344
Costs_SC5	.0318496	.0137914	2.31	0.021	.0048077	.0588915
Deterr_SD_Likely_SC2	.0070683	.0132628	0.53	0.594	-.0189372	.0330738
Deterr_SD_Severe	-.0032394	.0121665	-0.27	0.790	-.0270953	.0206165
MA_MoralBelief	.3384828	.0276056	12.26	0.000	.2843541	.3926115
MA_Authority_SC2	-.0145861	.0118807	-1.23	0.220	-.0378816	.0087094
NOO_SC3	.0862004	.0282665	3.05	0.002	.0307759	.141625
NNOO_SC3	-.0005102	.0228925	-0.02	0.982	-.0453976	.0443772
OOL_SC12	.0201419	.0160652	1.25	0.210	-.0113585	.0516423
PJE_SC4	-.0073118	.0133928	-0.55	0.585	-.0335722	.0189486
Trust_Science_SC4	.0649723	.0261401	2.49	0.013	.0137171	.1162275
Trust_in_media	-.0262524	.0165421	-1.59	0.113	-.058688	.0061833
Impulsivity_SC4	-.128795	.0201558	-6.39	0.000	-.1683162	-.0892737
NegEmo_SC6	.0229652	.0142416	1.61	0.107	-.0049595	.05089
SN_SC7	.1517372	.0159017	9.54	0.000	.1205573	.182917
_cons	1.083639	.216112	5.01	0.000	.6598891	1.507388

```
200 .
201 .
202 . *****************
203 .
204 . *7. Step 7: Add practical circumstances
205 .
206 . *7.a.1 Descriptive Statistics
207 . sum DV_Compliance_SC7 i.wave_d1 i.wave_d2 Age i.Gender_Female i.Minority Education i.Employed i.Corona_care i.Insura
    > rvative_01 i.Conservative_other i.GeoCensus_d1 i.GeoCensus_d2 i.GeoCensus_d3 i.Current_measures Measures_clear MA_Pe
    > _SC2 NOO_SC3 NNOO_SC3 OOL_SC12 PJE_SC4 Trust_Science_SC4 Trust_in_media Impulsivity_SC4 NegEmo_SC6 SN_SC7 CTC_SC7 OT
```

Variable	Obs	Mean	Std. Dev.	Min	Max
DV_Complia~7	2,919	5.856947	1.295922	1	7
wave_d1					
0	2,919	.6622131	.4730365	0	1
1	2,919	.3377869	.4730365	0	1
wave_d2					
0	2,919	.684481	.4648019	0	1
1	2,919	.315519	.4648019	0	1
Age	2,919	40.22234	13.05444	17	79
Gender_Fem~e					
0	2,919	.4546077	.4980206	0	1
1	2,919	.5453923	.4980206	0	1

Minority					
0	2,919	.6570743	.4747682	0	1
1	2,919	.3429257	.4747682	0	1
Education	2,919	3.857828	1.542854	1	8
Employed					
0	2,919	.3610826	.4803967	0	1
1	2,919	.6389174	.4803967	0	1
Corona_care					
0	2,919	.9122987	.2829084	0	1
1	2,919	.0877013	.2829084	0	1
Insurance_~c					
0	2,919	.4319973	.4954389	0	1
1	2,919	.5680027	.4954389	0	1
Insurance_~e					
0	2,919	.7060637	.4556412	0	1
1	2,919	.2939363	.4556412	0	1
SES_before	2,919	5.970538	2.050048	1	10
SES_change	2,919	-.2915382	1.654423	-9	9
Health_self					
1	2,919	.6605002	.4736207	0	1
2	2,919	.3394998	.4736207	0	1
Health_other					
1	2,919	.4162384	.4930185	0	1
2	2,919	.5837616	.4930185	0	1
Conservat~01					
0	2,919	.5477903	.4977961	0	1
1	2,919	.4522097	.4977961	0	1
Conservati~r					
0	2,919	.8800959	.324905	0	1
1	2,919	.1199041	.324905	0	1
GeoCensus_d1					
0	2,919	.7923947	.4056621	0	1
1	2,919	.2076053	.4056621	0	1
GeoCensus_d2					
0	2,919	.5721137	.4948571	0	1
1	2,919	.4278863	.4948571	0	1
GeoCensus_d3					
0	2,919	.8396711	.3669737	0	1
1	2,919	.1603289	.3669737	0	1
Current_me~s					
0	2,919	.1342926	.3410248	0	1
Yes	2,919	.8657074	.3410248	0	1
Measures_c~r	2,919	5.186023	1.727691	1	7
MA_Perc_Th~3	2,919	5.620418	1.506602	1	7
Costs_SC5	2,919	4.18383	1.639894	1	7
Deterr_SD_~2	2,919	3.256252	1.762824	1	7
Deterr_SD_~e	2,919	3.830079	1.718316	1	6
MA_MoralBe~f	2,919	6.15519	1.29568	1	7
MA_Authori~2	2,919	4.162384	1.890693	1	7
NOO_SC3	2,919	3.902935	.9011894	1	5

```
     NNOO_SC3 |      2,919    2.954665    .9979227          1          5
     OOL_SC12 |      2,919    4.356172    1.482673          1          7
      PJE_SC4 |      2,919    5.127698    1.613611          1          7
Trust_Scie~4 |      2,919     3.85149    .9866086          1          5
Trust_in_m~a |      2,919    2.896197    1.311564          1          5
-------------+---------------------------------------------------------
Impulsivi~C4 |      2,919    2.457263    1.124088          1          5
   NegEmo_SC6 |      2,919    4.584561     1.56834          1          7
       SN_SC7 |      2,919     5.25498    1.396018          1          7
      CTC_SC7 |      2,919    5.983507    .9897931          1          7
      OTC_SC7 |      2,919    4.588313    1.752943          1          7

208 .
209 . *7.a.2 Regression
210 . reg DV_Compliance_SC7 i.wave_d1 i.wave_d2 Age i.Gender_Female i.Minority Education i.Employed i.Corona_care i.Insura
    > rvative_01 i.Conservative_other i.GeoCensus_d1 i.GeoCensus_d2 i.GeoCensus_d3 i.Current_measures Measures_clear MA_Pe
    > _SC2 NOO_SC3 NNOO_SC3 OOL_SC12 PJE_SC4 Trust_Science_SC4 Trust_in_media Impulsivity_SC4 NegEmo_SC6 SN_SC7 CTC_SC7 OT

      Source |       SS           df       MS      Number of obs   =     2,919
-------------+----------------------------------   F(38, 2880)     =     82.08
       Model |  2547.85944        38  67.0489326   Prob > F        =    0.0000
    Residual |  2352.67106     2,880  .816899674   R-squared       =    0.5199
-------------+----------------------------------   Adj R-squared   =    0.5136
       Total |   4900.5305     2,918  1.67941415   Root MSE        =    .90383

------------------------------------------------------------------------------------
 DV_Compliance_SC7 |      Coef.   Std. Err.      t    P>|t|     [95% Conf. Interval]
-------------------+----------------------------------------------------------------
         1.wave_d1 |  -.0862688   .0413068    -2.09   0.037    -.1672627   -.0052749
         1.wave_d2 |  -.1414935   .0422287    -3.35   0.001    -.2242951   -.0586919
               Age |   .0034977   .0014407     2.43   0.015     .0006727    .0063226
   1.Gender_Female |   .1197573   .0353811     3.38   0.001     .0503825    .1891322
        1.Minority |  -.0049472   .0377992    -0.13   0.896    -.0790633     .069169
         Education |   .0348256   .0122788     2.84   0.005     .0107495    .0589017
        1.Employed |   .0139114   .0392159     0.35   0.723    -.0629826    .0908054
     1.Corona_care |  -.0463747   .0653273    -0.71   0.478    -.1744676    .0817182
1.Insurance_Public |   .0907822   .0537792     1.69   0.092    -.0146673    .1962317
1.Insurance_Private |  .1336903   .0562248     2.38   0.017     .0234454    .2439352
        SES_before |   .0050847   .0095566     0.53   0.595    -.0136537    .0238231
        SES_change |    .000065   .0112391     0.01   0.995    -.0219726    .0221025
     2.Health_self |   .0345024    .041112     0.84   0.401    -.0461095    .1151143
    2.Health_other |  -.0190751   .0390754    -0.49   0.625    -.0956936    .0575435
  1.Conservative_01 |    .049631   .0403531     1.23   0.219    -.0294928    .1287548
1.Conservative_other |    .10716   .0578927     1.85   0.064    -.0063552    .2206753
    1.GeoCensus_d1 |  -.0985249   .0530868    -1.86   0.064     -.202617    .0055671
    1.GeoCensus_d2 |  -.1254039   .0460539    -2.72   0.007    -.2157058    -.035102
    1.GeoCensus_d3 |   .0009032   .0565422     0.02   0.987     -.109964    .1117705
                   |
  Current_measures |
               Yes |   .1796554    .052325     3.43   0.001     .0770572    .2822537
    Measures_clear |   -.019975   .0115206    -1.73   0.083    -.0425645    .0026145
  MA_Perc_Threat_SC3 |  .1257663   .0171879     7.32   0.000     .0920645     .159468
         Costs_SC5 |   .0263656   .0122599     2.15   0.032     .0023264    .0504047
Deterr_SD_Likely_SC2 |  .0058539   .0120596     0.49   0.627    -.0177924    .0295001
  Deterr_SD_Severe |  -.0035242   .0106277    -0.33   0.740    -.0243628    .0173144
    MA_MoralBelief |    .246358   .0194719    12.65   0.000     .2081779    .2845382
   MA_Authority_SC2 |  -.0083059   .0112708    -0.74   0.461    -.0304054    .0137937
           NOO_SC3 |   .0172211   .0246708     0.70   0.485    -.0311532    .0655954
          NNOO_SC3 |   .0200771    .021095     0.95   0.341    -.0212858      .06144
          OOL_SC12 |   .0153333   .0142058     1.08   0.281    -.0125213    .0431878
           PJE_SC4 |  -.0097955   .0121594    -0.81   0.421    -.0336375    .0140465
 Trust_Science_SC4 |   .0379242   .0220408     1.72   0.085    -.0052931    .0811414
    Trust_in_media |  -.0123097   .0157435    -0.78   0.434    -.0431794    .0185601
   Impulsivity_SC4 |  -.0855071   .0190515    -4.49   0.000    -.1228631   -.0481512
        NegEmo_SC6 |   .0176301   .0126337     1.40   0.163     -.007142    .0424022
            SN_SC7 |   .0295245   .0145911     2.02   0.043     .0009144    .0581346
           CTC_SC7 |   .5159331   .0216451    23.84   0.000     .4734916    .5583746
           OTC_SC7 |  -.0263086   .0102054    -2.58   0.010    -.0463192    -.006298
             _cons |  -.0980783    .199224    -0.49   0.623    -.4887143    .2925578
------------------------------------------------------------------------------------
```

```
211 . estimates store model_7

212 . 
213 . *7.a.3 Check hettest: Run this right after your regression to apply the Breusch-Pagan / Cook-Weisberg test for heter
214 . *if significant, then you need to run the regression with vce(ro) at the end
215 . estat hettest

    Breusch-Pagan / Cook-Weisberg test for heteroskedasticity 
             Ho: Constant variance
             Variables: fitted values of DV_Compliance_SC7

             chi2(1)      =   367.84
             Prob > chi2  =   0.0000

216 . 
217 . *7.a.4. check vif, to check for for multicollinearity (VIFs >10 are problematic)
218 . vif
```

Variable	VIF	1/VIF
1.wave_d1	1.36	0.733248
1.wave_d2	1.38	0.726660
Age	1.26	0.791415
1.Gender_F~e	1.11	0.901667
1.Minority	1.15	0.869272
Education	1.28	0.780049
1.Employed	1.27	0.788787
1.Corona_c~e	1.22	0.819601
1.Insuranc~c	2.54	0.394344
1.Insuran~te	2.34	0.426562
SES_before	1.37	0.729378
SES_change	1.24	0.809700
2.Health_s~f	1.35	0.738389
2.Health_o~r	1.33	0.754311
1.Conserv~01	1.44	0.693789
1.Conserva~r	1.26	0.791268
1.GeoCensu~1	1.66	0.603644
1.GeoCensu~2	1.86	0.539003
1.GeoCensu~3	1.54	0.650232
1.Current_~s	1.14	0.879211
Measures_c~r	1.42	0.706643
MA_Perc_Th~3	2.40	0.417487
Costs_SC5	1.44	0.692588
Deterr_SD_~2	1.61	0.619445
Deterr_SD_~e	1.19	0.839462
MA_MoralBe~f	2.27	0.439818
MA_Authori~2	1.62	0.616503
NOO_SC3	1.77	0.566349
NNOO_SC3	1.58	0.631727
OOL_SC12	1.58	0.631047
PJE_SC4	1.38	0.727212
Trust_Scie~4	1.69	0.592025
Trust_in_m~a	1.52	0.656597
Impulsivi~C4	1.64	0.610415
NegEmo_SC6	1.40	0.713081
SN_SC7	1.48	0.674721
CTC_SC7	1.64	0.609922
OTC_SC7	1.14	0.874760
Mean VIF	1.52	

```
219 . 
```

```
220 . *7.a.5. Effect size
221 . estat esize
```

Effect sizes for linear models

Source	Eta-Squared	df	[95% Conf.	Interval]
Model	.519915	38	.4910424	.5350256
wave_d1	.0015122	1	.	.0056564
wave_d2	.0038831	1	.0006685	.0096968
Age	.0020423	1	.0000636	.006638
Gender_Female	.0039623	1	.0007017	.0098207
Minority	5.95e-06	1	.	.0009947
Education	.0027854	1	.0002643	.0079223
Employed	.0000437	1	.	.0017369
Corona_care	.0001749	1	.	.0024556
Insurance_Public	.0009884	1	.	.0045973
Insurance_Private	.0019593	1	.0000451	.0064887
SES_before	.0000983	1	.	.0021116
SES_change	1.16e-08	1	.	.
Health_self	.0002445	1	.	.0027069
Health_other	.0000827	1	.	.0020241
Conservative_01	.000525	1	.	.0035184
Conservative_other	.0011883	1	.	.0050152
GeoCensus_d1	.0011946	1	.	.0050281
GeoCensus_d2	.0025679	1	.0001989	.0075551
GeoCensus_d3	8.86e-08	1	.	.
Current_measures	.0040766	1	.0007504	.0099987
Measures_clear	.0010427	1	.	.0047129
MA_Perc_Threat_SC3	.0182513	1	.0098246	.0290484
Costs_SC5	.0016033	1	.	.0058302
Deterr_SD_Likely_SC2	.0000818	1	.	.0020185
Deterr_SD_Severe	.0000382	1	.	.0016816
MA_MoralBelief	.0526544	1	.0379606	.0692024
MA_Authority_SC2	.0001885	1	.	.0025078
NOO_SC3	.0001692	1	.	.0024328
NNOO_SC3	.0003144	1	.	.0029313
OOL_SC12	.0004044	1	.	.0031945
PJE_SC4	.0002253	1	.	.002641
Trust_Science_SC4	.0010269	1	.	.0046794
Trust_in_media	.0002122	1	.	.0025948
Impulsivity_SC4	.0069459	1	.0022064	.0142351
NegEmo_SC6	.0006757	1	.	.0038919
SN_SC7	.0014196	1	.	.0054771
CTC_SC7	.1647705	1	.141331	.1886744
OTC_SC7	.0023022	1	.0001265	.0070973

Note: Eta-Squared values for individual model terms are partial.

```
222 .
223 . *7.a.6 Regression with vce(ro)
224 . reg DV_Compliance_SC7 i.wave_d1 i.wave_d2 Age i.Gender_Female i.Minority Education i.Employed i.Corona_care i.Insura
    > rvative_01 i.Conservative_other i.GeoCensus_d1 i.GeoCensus_d2 i.GeoCensus_d3 i.Current_measures Measures_clear MA_Pe
    > _SC2 NOO_SC3 NNOO_SC3 OOL_SC12 PJE_SC4 Trust_Science_SC4 Trust_in_media Impulsivity_SC4 NegEmo_SC6 SN_SC7 CTC_SC7 OT
```

Linear regression

	Number of obs	=	2,919
	F(38, 2880)	=	70.72
	Prob > F	=	0.0000
	R-squared	=	0.5199
	Root MSE	=	.90383

DV_Compliance_SC7	Coef.	Robust Std. Err.	t	P>|t|	[95% Conf.	Interval]
1.wave_d1	-.0862688	.0401253	-2.15	0.032	-.164946	-.0075916
1.wave_d2	-.1414935	.0420582	-3.36	0.001	-.2239607	-.0590264
Age	.0034977	.0014364	2.44	0.015	.0006813	.006314
1.Gender_Female	.1197573	.0354362	3.38	0.001	.0502744	.1892402
1.Minority	-.0049472	.038885	-0.13	0.899	-.0811924	.071298
Education	.0348256	.0120955	2.88	0.004	.0111089	.0585422
1.Employed	.0139114	.040399	0.34	0.731	-.0653024	.0931253
1.Corona_care	-.0463747	.0702339	-0.66	0.509	-.1840886	.0913391
1.Insurance_Public	.0907822	.0601155	1.51	0.131	-.0270915	.2086559
1.Insurance_Private	.1336903	.0625858	2.14	0.033	.0109728	.2564077
SES_before	.0050847	.010365	0.49	0.624	-.0152389	.0254083
SES_change	.000065	.0124175	0.01	0.996	-.0242832	.0244131
2.Health_self	.0345024	.0369302	0.93	0.350	-.03791	.1069148
2.Health_other	-.0190751	.0377804	-0.50	0.614	-.0931544	.0550043
1.Conservative_01	.049631	.0408158	1.22	0.224	-.0304	.129662
1.Conservative_other	.10716	.0616002	1.74	0.082	-.013625	.227945
1.GeoCensus_d1	-.0985249	.051845	-1.90	0.057	-.2001819	.0031321
1.GeoCensus_d2	-.1254039	.0441907	-2.84	0.005	-.2120524	-.0387554
1.GeoCensus_d3	.0009032	.0513811	0.02	0.986	-.0998441	.1016506
Current_measures						
Yes	.1796554	.063872	2.81	0.005	.0544161	.3048948
Measures_clear	-.019975	.0121872	-1.64	0.101	-.0438715	.0039214
MA_Perc_Threat_SC3	.1257663	.019691	6.39	0.000	.0871563	.1643762
Costs_SC5	.0263656	.0126345	2.09	0.037	.0015921	.0511391
Deterr_SD_Likely_SC2	.0058539	.0122768	0.48	0.634	-.0182184	.0299262
Deterr_SD_Severe	-.0035242	.01114	-0.32	0.752	-.0253673	.0183188
MA_MoralBelief	.246358	.0259189	9.50	0.000	.1955366	.2971795
MA_Authority_SC2	-.0083059	.0108809	-0.76	0.445	-.0296409	.0130292
NOO_SC3	.0172211	.0257454	0.67	0.504	-.0332601	.0677023
NNOO_SC3	.0200771	.0215234	0.93	0.351	-.0221256	.0622799
OOL_SC12	.0153333	.0147361	1.04	0.298	-.0135611	.0442276
PJE_SC4	-.0097955	.0120113	-0.82	0.415	-.0333472	.0137562
Trust_Science_SC4	.0379242	.0234925	1.61	0.107	-.0081397	.0839881
Trust_in_media	-.0123097	.0150382	-0.82	0.413	-.0417965	.0171772
Impulsivity_SC4	-.0855071	.0187594	-4.56	0.000	-.1222902	-.048724
NegEmo_SC6	.0176301	.0129022	1.37	0.172	-.0076684	.0429286
SN_SC7	.0295245	.0146569	2.01	0.044	.0007854	.0582637
CTC_SC7	.5159331	.0319965	16.12	0.000	.4531947	.5786714
OTC_SC7	-.0263086	.009764	-2.69	0.007	-.0454538	-.0071634
_cons	-.0980783	.2118318	-0.46	0.643	-.5134355	.317279

```
. 
. 
. 
. *****************
. 
. *8. Compare Rsq models
. 
. *8.a.1. model 1 vs model 2
. lrtest model_1 model_2

Likelihood-ratio test                                 LR chi2(2)  =    223.89
(Assumption: model_1 nested in model_2)               Prob > chi2 =    0.0000

. 
. *8.a.2. model 2 vs model 3
```

```
236 . lrtest model_2 model_3

    Likelihood-ratio test                              LR chi2(4)  =    622.75
    (Assumption: model_2 nested in model_3)            Prob > chi2 =    0.0000

237 .
238 . *8.a.3. model 3 vs model 4
239 . lrtest model_3 model_4

    Likelihood-ratio test                              LR chi2(6)  =    383.19
    (Assumption: model_3 nested in model_4)            Prob > chi2 =    0.0000

240 .
241 . *8.a.4. model 4 vs model 5
242 . lrtest model_4 model_5

    Likelihood-ratio test                              LR chi2(4)  =     46.37
    (Assumption: model_4 nested in model_5)            Prob > chi2 =    0.0000

243 .
244 . *8.a.5. model 5 vs model 6
245 . lrtest model_5 model_6

    Likelihood-ratio test                              LR chi2(1)  =    103.62
    (Assumption: model_5 nested in model_6)            Prob > chi2 =    0.0000

246 .
247 . *8.a.6. model 6 vs model 7
248 . lrtest model_6 model_7

    Likelihood-ratio test                              LR chi2(2)  =    532.61
    (Assumption: model_6 nested in model_7)            Prob > chi2 =    0.0000

249 .
250 .
251 . ***********************************************************************************
252 . ***********************************************************************************
253 . ***********************************************************************************
254 . ***********************************************************************************
255 .
256 . *INTERACTIONS WITH SURVEY WAVE
257 .
258 . ***********************************************************************************
259 . ***********************************************************************************
260 . ***********************************************************************************
261 . ***********************************************************************************
262 .
263 .
264 . *9. Model 9: Knowledge x Survey Wave
265 .
266 . *9.a.2 Regression
267 . reg DV_Compliance_SC7 i.wave_d1 i.wave_d2 Age i.Gender_Female i.Minority Education i.Employed i.Corona_care i.Insura
    > rvative_01 i.Conservative_other i.GeoCensus_d1 i.GeoCensus_d2 i.GeoCensus_d3 i.Current_measures Measures_clear MA_Pe
    > _SC2 NOO_SC3 NNOO_SC3 OOL_SC12 PJE_SC4 Trust_Science_SC4 Trust_in_media Impulsivity_SC4 NegEmo_SC6 SN_SC7 CTC_SC7 OT
```

Source	SS	df	MS			
				Number of obs	=	2,919
				F(40, 2878)	=	77.96
Model	2548.56195	40	63.7140487	Prob > F	=	0.0000
Residual	2351.96855	2,878	.817223263	R-squared	=	0.5201
				Adj R-squared	=	0.5134
Total	4900.5305	2,918	1.67941415	Root MSE	=	.904

DV_Compliance_SC7	Coef.	Std. Err.	t	P>|t|	[95% Conf.	Interval]
1.wave_d1	-.1123373	.1159555	-0.97	0.333	-.3397014	.1150268
1.wave_d2	-.0767031	.1231258	-0.62	0.533	-.3181268	.1647205
Age	.0034763	.0014414	2.41	0.016	.0006499	.0063026
1.Gender_Female	.1199388	.0353935	3.39	0.001	.0505396	.1893379
1.Minority	-.0051104	.0378105	-0.14	0.892	-.0792488	.069028
Education	.0352293	.01229	2.87	0.004	.0111312	.0593274
1.Employed	.0140676	.0392252	0.36	0.720	-.0628447	.0909798
1.Corona_care	-.0469441	.0653432	-0.72	0.473	-.1750683	.0811802
1.Insurance_Public	.0907821	.0538397	1.69	0.092	-.0147862	.1963504

```
     1.Insurance_Private    .1335921   .0562512     2.37   0.018     .0232954    .2438888
              SES_before    .0052048   .0095622     0.54   0.586    -.0135447    .0239542
              SES_change    .0000139   .0112444     0.00   0.999     -.022034    .0220619
           2.Health_self    .0360859   .0411563     0.88   0.381    -.0446128    .1167846
          2.Health_other   -.0194522   .0390908    -0.50   0.619     -.096101    .0571967
      1.Conservative_01     .0495815   .0403627     1.23   0.219    -.0295613    .1287243
    1.Conservative_other    .1064242    .057912     1.84   0.066    -.0071289    .2199773
         1.GeoCensus_d1    -.0989581   .0531025    -1.86   0.062    -.2030808    .0051647
         1.GeoCensus_d2    -.1243625   .0460785    -2.70   0.007    -.2147127   -.0340122
         1.GeoCensus_d3     .0024126   .0565768     0.04   0.966    -.1085226    .1133478

        Current_measures
                     Yes    .1904789   .0987624     1.93   0.054    -.0031732    .3841311
          Measures_clear   -.0197765    .011525    -1.72   0.086    -.0423746    .0028217
       MA_Perc_Threat_SC3   .1254717   .0171942     7.30   0.000     .0917575    .1591859
               Costs_SC5    .0261132   .0122655     2.13   0.033     .0020632    .0501631
     Deterr_SD_Likely_SC2   .0058555   .0120635     0.49   0.627    -.0177985    .0295095
        Deterr_SD_Severe   -.0039604   .0106445    -0.37   0.710     -.024832    .0169112
           MA_MoralBelief   .2460098    .019481    12.63   0.000     .2078116    .2842079
        MA_Authority_SC2   -.0083234   .0112754    -0.74   0.460    -.0304321    .0137854
                 NOO_SC3    .0170776   .0246782     0.69   0.489    -.0313112    .0654664
                NNOO_SC3    .0199461   .0211074     0.94   0.345     -.021441    .0613333
                OOL_SC12    .0154577   .0142158     1.09   0.277    -.0124165     .043332
                 PJE_SC4   -.0098812   .0121623    -0.81   0.417    -.0337288    .0139665
       Trust_Science_SC4    .0381696    .022047     1.73   0.084    -.0050599     .081399
          Trust_in_media   -.0126209   .0157551    -0.80   0.423    -.0435134    .0182715
        Impulsivity_SC4    -.0856332   .0190567    -4.49   0.000    -.1229992   -.0482671
              NegEmo_SC6    .0176999   .0126379     1.40   0.161    -.0070803    .0424801
                  SN_SC7    .0294877   .0145944     2.02   0.043     .0008711    .0581043
                 CTC_SC7    .5162768   .0216527    23.84   0.000     .4738204    .5587332
                 OTC_SC7   -.0260288    .010212    -2.55   0.011    -.0460524   -.0060052

Current_measures#wave_d1
                   Yes#1    .0323446   .1236839     0.26   0.794    -.2101735    .2748626

Current_measures#wave_d2
                   Yes#1   -.0744336   .1304392    -0.57   0.568    -.3301973    .1813302

                   _cons   -.1069567   .2123213    -0.50   0.614    -.5232738    .3093605

268 .
269 . *9.a.3 Check hettest: Run this right after your regression to apply the Breusch-Pagan / Cook-Weisberg test for heter
270 . *if significant, then you need to run the regression with vce(ro) at the end
271 . estat hettest

Breusch-Pagan / Cook-Weisberg test for heteroskedasticity
         Ho: Constant variance
         Variables: fitted values of DV_Compliance_SC7

         chi2(1)      =   366.91
         Prob > chi2  =   0.0000

272 .
273 . *9.a.4. check vif, to check for for multicollinearity (VIFs >10 are problematic)
274 . vif

    Variable         VIF       1/VIF

    1.wave_d1       10.74    0.093086
    1.wave_d2       11.69    0.085511
          Age        1.26    0.790941
1.Gender_F~e         1.11    0.901394
  1.Minority         1.15    0.869096
   Education         1.28    0.778936
  1.Employed         1.27    0.788725
1.Corona_c~e         1.22    0.819525
1.Insuranc~c         2.54    0.393613
1.Insuran~te         2.35    0.426330
  SES_before         1.37    0.728806
  SES_change         1.24    0.809258
2.Health_s~f         1.36    0.737093
2.Health_o~r         1.33    0.754014
1.Conserv~01         1.44    0.693731
```

```
1.Conserva~r |      1.26    0.791054
1.GeoCensu~1 |      1.66    0.603527
1.GeoCensu~2 |      1.86    0.538640
1.GeoCensu~3 |      1.54    0.649693
1.Current_~s |      4.05    0.246888
Measures_c~r |      1.42    0.706380
MA_Perc_Th~3 |      2.40    0.417344
   Costs_SC5 |      1.44    0.692240
Deterr_SD_~2 |      1.61    0.619285
Deterr_SD_~e |      1.19    0.837143
MA_MoralBe~f |      2.27    0.439578
MA_Authori~2 |      1.62    0.616235
     NOO_SC3 |      1.77    0.566232
    NNOO_SC3 |      1.58    0.631236
    OOL_SC12 |      1.59    0.630405
     PJE_SC4 |      1.38    0.727158
Trust_Scie~4 |      1.69    0.591927
Trust_in_m~a |      1.52    0.655893
Impulsivi~C4 |      1.64    0.610326
  NegEmo_SC6 |      1.40    0.712898
      SN_SC7 |      1.48    0.674682
     CTC_SC7 |      1.64    0.609736
     OTC_SC7 |      1.14    0.873969
Current_me~s#|
     wave_d1 |
        1 1  |     11.01    0.090802
Current_me~s#|
     wave_d2 |
        1 1  |     12.04    0.083030
-------------+----------------------
    Mean VIF |      2.59
```

```
275 .
276 . *9.a.5. Effect size
277 . estat esize
```

Effect sizes for linear models

Source	Eta-Squared	df	[95% Conf.	Interval]
Model	.5200584	40	.4908332	.5348485
wave_d1	.0008289	1	.	.0042485
wave_d2	.0010405	1	.	.0047099
Age	.0020168	1	.0000576	.0065944
Gender_Female	.0039742	1	.0007061	.0098418
Minority	6.35e-06	1	.	.0010181
Education	.0028469	1	.0002833	.0080274
Employed	.0000447	1	.	.0017472
Corona_care	.0001793	1	.	.0024738
Insurance_Public	.0009869	1	.	.0045957
Insurance_Private	.0019559	1	.0000441	.0064847
SES_before	.0001029	1	.	.0021372
SES_change	5.35e-10	1	.	.
Health_self	.0002671	1	.	.0027831
Health_other	.000086	1	.	.0020445
Conservative_01	.000524	1	.	.0035175
Conservative_other	.001172	1	.	.0049838
GeoCensus_d1	.0012052	1	.	.0050516
GeoCensus_d2	.0025246	1	.0001862	.0074834
GeoCensus_d3	6.32e-07	1	.	.0002138
Current_measures	.002738	1	.0002492	.0078451
Measures_clear	.0010221	1	.	.0046708
MA_Perc_Threat_SC3	.0181666	1	.0097595	.0289473
Costs_SC5	.0015725	1	.	.0057736
Deterr_SD_Likely_SC2	.0000819	1	.	.0020199
Deterr_SD_Severe	.0000481	1	.	.0017782
MA_MoralBelief	.0525012	1	.0378234	.0690367
MA_Authority_SC2	.0001893	1	.	.0025119
NOO_SC3	.0001664	1	.	.0024229
NNOO_SC3	.0003102	1	.	.0029197
OOL_SC12	.0004107	1	.	.0032136
PJE_SC4	.0002293	1	.	.0026562

Trust_Science_SC4	.0010404	1	.	.0047096
Trust_in_media	.0002229	1	.	.002634
Impulsivity_SC4	.0069673	1	.0022173	.0142684
NegEmo_SC6	.0006811	1	.	.0039063
SN_SC7	.0014164	1	.	.0054727
CTC_SC7	.1649531	1	.1414978	.1888712
OTC_SC7	.0022522	1	.0001135	.007012
Current_measures#wave_d1	.0000238	1	.	.0014968
Current_measures#wave_d2	.0001131	1	.	.0021887

Note: Eta-Squared values for individual model terms are partial.

```
278 .
279 . *9.a.6 Regression with vce(ro)
280 . reg DV_Compliance_SC7 i.wave_d1 i.wave_d2 Age i.Gender_Female i.Minority Education i.Employed i.Corona_care i.Insura
    > rvative_01 i.Conservative_other i.GeoCensus_d1 i.GeoCensus_d2 i.GeoCensus_d3 i.Current_measures Measures_clear MA_Pe
    > _SC2 NOO_SC3 NNOO_SC3 OOL_SC12 PJE_SC4 Trust_Science_SC4 Trust_in_media Impulsivity_SC4 NegEmo_SC6 SN_SC7 CTC_SC7 OT
    >  1, vce(ro)
```

Linear regression

Number of obs	=	2,919
F(40, 2878)	=	67.24
Prob > F	=	0.0000
R-squared	=	0.5201
Root MSE	=	.904

DV_Compliance_SC7	Coef.	Robust Std. Err.	t	P>|t|	[95% Conf.	Interval]
1.wave_d1	-.1123373	.1405605	-0.80	0.424	-.3879467	.1632722
1.wave_d2	-.0767031	.1537416	-0.50	0.618	-.378158	.2247518
Age	.0034763	.0014387	2.42	0.016	.0006553	.0062972
1.Gender_Female	.1199388	.0354941	3.38	0.001	.0503424	.1895351
1.Minority	-.0051104	.0388526	-0.13	0.895	-.0812922	.0710714
Education	.0352293	.0120767	2.92	0.004	.0115495	.0589092
1.Employed	.0140676	.0404492	0.35	0.728	-.0652447	.0933798
1.Corona_care	-.0469441	.0701766	-0.67	0.504	-.1845456	.0906575
1.Insurance_Public	.0907821	.0600377	1.51	0.131	-.0269391	.2085033
1.Insurance_Private	.1335921	.0626037	2.13	0.033	.0108394	.2563448
SES_before	.0052048	.0103487	0.50	0.615	-.0150868	.0254964
SES_change	.0000139	.0124215	0.00	0.999	-.0243419	.0243698
2.Health_self	.0360859	.0369331	0.98	0.329	-.0363321	.1085039
2.Health_other	-.0194522	.0378691	-0.51	0.608	-.0937054	.0548011
1.Conservative_01	.0495815	.0408131	1.21	0.225	-.0304443	.1296072
1.Conservative_other	.1064242	.0616055	1.73	0.084	-.0143712	.2272196
1.GeoCensus_d1	-.0989581	.0518845	-1.91	0.057	-.2006925	.0027764
1.GeoCensus_d2	-.1243625	.0441085	-2.82	0.005	-.2108499	-.037875
1.GeoCensus_d3	.0024126	.0515658	0.05	0.963	-.098697	.1035222
Current_measures						
Yes	.1904789	.1189391	1.60	0.109	-.0427355	.4236934
Measures_clear	-.0197765	.0121978	-1.62	0.105	-.0436938	.0041409
MA_Perc_Threat_SC3	.1254717	.0196801	6.38	0.000	.0868832	.1640603
Costs_SC5	.0261132	.0126397	2.07	0.039	.0013295	.0508969
Deterr_SD_Likely_SC2	.0058555	.0122534	0.48	0.633	-.0181707	.0298818
Deterr_SD_Severe	-.0039604	.0111042	-0.36	0.721	-.0257335	.0178127
MA_MoralBelief	.2460098	.0259039	9.50	0.000	.1952176	.2968019
MA_Authority_SC2	-.0083234	.0108889	-0.76	0.445	-.0296743	.0130275
NOO_SC3	.0170776	.0257152	0.66	0.507	-.0333444	.0674996
NNOO_SC3	.0199461	.0215383	0.93	0.354	-.0222859	.0621781
OOL_SC12	.0154577	.0146829	1.05	0.293	-.0133322	.0442477
PJE_SC4	-.0098812	.0120041	-0.82	0.410	-.0334186	.0136563
Trust_Science_SC4	.0381696	.0235011	1.62	0.104	-.0079111	.0842503
Trust_in_media	-.0126209	.0151009	-0.84	0.403	-.0422305	.0169886
Impulsivity_SC4	-.0856332	.0187547	-4.57	0.000	-.1224072	-.0488592
NegEmo_SC6	.0176999	.0129135	1.37	0.171	-.0076207	.0430205
SN_SC7	.0294877	.014656	2.01	0.044	.0007503	.058225
CTC_SC7	.5162768	.0319951	16.14	0.000	.453541	.5790125
OTC_SC7	-.0260288	.0097699	-2.66	0.008	-.0451856	-.006872
Current_measures#wave_d1						
Yes#1	.0323446	.1466875	0.22	0.825	-.2552786	.3199678
Current_measures#wave_d2						

	Coef.	Std. Err.	t	P>|t|	[95% Conf. Interval]	
Yes#1	-.0744336	.1599549	-0.47	0.642	-.3880713	.2392042
_cons	-.1069567	.2222435	-0.48	0.630	-.5427292	.3288159

```
281 .
282 .
283 . *****************
284 .
285 . *10. Model 10: Clarity x Survey Wave
286 .
287 . *10.a.2 Regression
288 . reg DV_Compliance_SC7 i.wave_d1 i.wave_d2 Age i.Gender_Female i.Minority Education i.Employed i.Corona_care i.Insura
    > rvative_01 i.Conservative_other i.GeoCensus_d1 i.GeoCensus_d2 i.GeoCensus_d3 i.Current_measures Measures_clear MA_Pe
    > _SC2 NOO_SC3 NNOO_SC3 OOL_SC12 PJE_SC4 Trust_Science_SC4 Trust_in_media Impulsivity_SC4 NegEmo_SC6 SN_SC7 CTC_SC7 OT
    note: Measures_clear omitted because of collinearity
    note: Measures_clear omitted because of collinearity
```

Source	SS	df	MS			
				Number of obs	=	2,919
				F(40, 2878)	=	78.05
Model	2549.92621	40	63.7481554	Prob > F	=	0.0000
Residual	2350.60429	2,878	.816749231	R-squared	=	0.5203
				Adj R-squared	=	0.5137
Total	4900.5305	2,918	1.67941415	Root MSE	=	.90374

DV_Compliance_SC7	Coef.	Std. Err.	t	P>|t|	[95% Conf.	Interval]
1.wave_d1	-.2830091	.1341096	-2.11	0.035	-.5459696	-.0200486
1.wave_d2	-.2897053	.1326875	-2.18	0.029	-.5498774	-.0295332
Age	.0034851	.0014424	2.42	0.016	.000657	.0063133
1.Gender_Female	.118353	.0353895	3.34	0.001	.0489616	.1877444
1.Minority	-.0061541	.0378106	-0.16	0.871	-.0802927	.0679844
Education	.0349213	.0122806	2.84	0.004	.0108417	.0590009
1.Employed	.0138563	.0392132	0.35	0.724	-.0630324	.090745
1.Corona_care	-.0494658	.0653766	-0.76	0.449	-.1776554	.0787238
1.Insurance_Public	.0921238	.0537808	1.71	0.087	-.013329	.1975766
1.Insurance_Private	.1342208	.0562208	2.39	0.017	.0239836	.244458
SES_before	.0049111	.0095577	0.51	0.607	-.0138295	.0236517
SES_change	.0001864	.0112385	0.02	0.987	-.0218499	.0222226
2.Health_self	.0324131	.0411797	0.79	0.431	-.0483315	.1131578
2.Health_other	-.0171484	.0390908	-0.44	0.661	-.0937973	.0595005
1.Conservative_01	.0484979	.0403571	1.20	0.230	-.030634	.1276297
1.Conservative_other	.10732	.0578874	1.85	0.064	-.006185	.2208251
1.GeoCensus_d1	-.0989635	.0530884	-1.86	0.062	-.2030586	.0051316
1.GeoCensus_d2	-.124625	.0460569	-2.71	0.007	-.2149329	-.034317
1.GeoCensus_d3	-.0011503	.0565691	-0.02	0.984	-.1120704	.1097698
Current_measures						
Yes	.1769751	.0523502	3.38	0.001	.0743273	.2796228
Measures_clear	-.0426031	.0186936	-2.28	0.023	-.0792573	-.0059489
MA_Perc_Threat_SC3	.1260254	.0171936	7.33	0.000	.0923124	.1597383
Costs_SC5	.0267555	.0122613	2.18	0.029	.0027137	.0507973
Deterr_SD_Likely_SC2	.0058913	.0120622	0.49	0.625	-.0177602	.0295428
Deterr_SD_Severe	-.0042312	.0106394	-0.40	0.691	-.0250929	.0166305
MA_MoralBelief	.2461499	.0194734	12.64	0.000	.2079666	.2843331
MA_Authority_SC2	-.0082445	.011289	-0.73	0.465	-.0303798	.0138909
NOO_SC3	.0175807	.0246708	0.71	0.476	-.0307935	.065955
NNOO_SC3	.0206639	.0211016	0.98	0.328	-.0207118	.0620396
OOL_SC12	.015487	.0142105	1.09	0.276	-.0123769	.0433509
PJE_SC4	-.0090301	.0121714	-0.74	0.458	-.0328956	.0148353
Trust_Science_SC4	.0379403	.0220399	1.72	0.085	-.0052753	.0811558
Trust_in_media	-.0132158	.0157527	-0.84	0.402	-.0441035	.017672
Impulsivity_SC4	-.0853015	.0190542	-4.48	0.000	-.1226629	-.0479402
NegEmo_SC6	.0175669	.0126334	1.39	0.164	-.0072046	.0423384
SN_SC7	.0293652	.0145938	2.01	0.044	.0007498	.0579806
CTC_SC7	.5163847	.0216494	23.85	0.000	.4739347	.5588347
OTC_SC7	-.0264225	.0102048	-2.59	0.010	-.046432	-.0064131
Measures_clear	0	(omitted)				
wave_d1#c.Measures_clear						
1	.0373439	.0242978	1.54	0.124	-.0102989	.0849867
Measures_clear	0	(omitted)				

```
wave_d2#c.Measures_clear │
                       1 │   .0280233    .024184     1.16   0.247    -.0193964     .075443
                         │
                   _cons │   .0211011   .2136291     0.10   0.921    -.3977804    .4399826
─────────────────────────┴────────────────────────────────────────────────────────────────

289 .
290 . *10.a.3 Check hettest: Run this right after your regression to apply the Breusch-Pagan / Cook-Weisberg test for hete
291 . *if significant, then you need to run the regression with vce(ro) at the end
292 . estat hettest

Breusch-Pagan / Cook-Weisberg test for heteroskedasticity
         Ho: Constant variance
         Variables: fitted values of DV_Compliance_SC7

         chi2(1)      =   369.82
         Prob > chi2  =   0.0000

293 .
294 . *10.a.4. check vif, to check for for multicollinearity (VIFs >10 are problematic)
295 . vif
```

Variable	VIF	1/VIF
1.wave_d1	14.38	0.069550
1.wave_d2	13.59	0.073588
Age	1.27	0.789479
1.Gender_F~e	1.11	0.901071
1.Minority	1.15	0.868588
Education	1.28	0.779682
1.Employed	1.27	0.788750
1.Corona_c~e	1.22	0.818215
1.Insuranc~c	2.54	0.394247
1.Insuran~te	2.34	0.426543
SES_before	1.37	0.729070
SES_change	1.24	0.809650
2.Health_s~f	1.36	0.735828
2.Health_o~r	1.33	0.753575
1.Conserv~01	1.44	0.693521
1.Conserva~r	1.26	0.791265
1.GeoCensu~1	1.66	0.603497
1.GeoCensu~2	1.86	0.538832
1.GeoCensu~3	1.54	0.649493
1.Current_~s	1.14	0.878202
Measures_c~r	3.73	0.268340
MA_Perc_Th~3	2.40	0.417133
Costs_SC5	1.44	0.692309
Deterr_SD_~2	1.62	0.619058
Deterr_SD_~e	1.19	0.837452
MA_MoralBe~f	2.27	0.439668
MA_Authori~2	1.63	0.614398
NOO_SC3	1.77	0.566245
NNOO_SC3	1.58	0.631219
OOL_SC12	1.59	0.630509
PJE_SC4	1.38	0.725650
Trust_Scie~4	1.69	0.591964
Trust_in_m~a	1.53	0.655712
Impulsivi~C4	1.64	0.610126
NegEmo_SC6	1.40	0.712986
SN_SC7	1.48	0.674347
CTC_SC7	1.64	0.609566
OTC_SC7	1.14	0.874698
wave_d1# c. Measures_c~r 1	14.66	0.068233
wave_d2# c. Measures_c~r 1	13.60	0.073518
Mean VIF	2.84	

```
296 .
297 . *10.a.5. Effect size
298 . estat esize

    Effect sizes for linear models
```

Source	Eta-Squared	df	[95% Conf.	Interval]
Model	.5203368	40	.491124	.5351217
wave_d1	.001545	1	.	.0057211
wave_d2	.0016536	1	.	.0059273
Age	.0020245	1	.0000593	.0066083
Gender_Female	.0038711	1	.0006628	.0096805
Minority	9.20e-06	1	.	.0011498
Education	.0028018	1	.000269	.007952
Employed	.0000434	1	.	.0017349
Corona_care	.0001989	1	.	.0025476
Insurance_Public	.0010185	1	.	.0046632
Insurance_Private	.0019765	1	.0000486	.0065218
SES_before	.0000917	1	.	.002077
SES_change	9.55e-08	1	.	.
Health_self	.0002152	1	.	.0026068
Health_other	.0000669	1	.	.0019236
Conservative_01	.0005015	1	.	.003459
Conservative_other	.0011928	1	.	.0050264
GeoCensus_d1	.001206	1	.	.0050531
GeoCensus_d2	.0025376	1	.0001898	.0075056
GeoCensus_d3	1.44e-07	1	.	.
Current_measures	.0039553	1	.0006981	.0098122
Measures_clear	.0001959	1	.	.0025366
MA_Perc_Threat_SC3	.0183257	1	.0098773	.0291451
Costs_SC5	.0016518	1	.	.0059237
Deterr_SD_Likely_SC2	.0000829	1	.	.002026
Deterr_SD_Severe	.000055	1	.	.0018356
MA_MoralBelief	.0525969	1	.0379061	.069144
MA_Authority_SC2	.0001853	1	.	.0024967
NOO_SC3	.0001764	1	.	.0024626
NNOO_SC3	.0003331	1	.	.0029893
OOL_SC12	.0004125	1	.	.0032188
PJE_SC4	.0001912	1	.	.0025191
Trust_Science_SC4	.0010286	1	.	.0046847
Trust_in_media	.0002445	1	.	.0027082
Impulsivity_SC4	.0069155	1	.002188	.0141951
NegEmo_SC6	.0006714	1	.	.0038831
SN_SC7	.0014048	1	.	.00545
CTC_SC7	.1650523	1	.1415928	.1889735
OTC_SC7	.002324	1	.0001318	.0071374
Measures_clear	.0001959	1	.	.0025366
wave_d1#c.Measures_clear	.0008201	1	.	.0042286
Measures_clear	.0001959	1	.	.0025366
wave_d2#c.Measures_clear	.0004663	1	.	.0033658

```
    Note: Eta-Squared values for individual model terms are partial.

299 .
300 . *10.a.6 Regression with vce(ro)
301 . reg DV_Compliance_SC7 i.wave_d1 i.wave_d2 Age i.Gender_Female i.Minority Education i.Employed i.Corona_care i.Insura
    > rvative_01 i.Conservative_other i.GeoCensus_d1 i.GeoCensus_d2 i.GeoCensus_d3 i.Current_measures Measures_clear MA_Pe
    > _SC2 NOO_SC3 NNOO_SC3 OOL_SC12 PJE_SC4 Trust_Science_SC4 Trust_in_media Impulsivity_SC4 NegEmo_SC6 SN_SC7 CTC_SC7 OT
    > vce(ro)
    note: Measures_clear omitted because of collinearity
    note: Measures_clear omitted because of collinearity

    Linear regression                               Number of obs     =      2,919
                                                    F(40, 2878)       =      67.25
                                                    Prob > F          =     0.0000
                                                    R-squared         =     0.5203
                                                    Root MSE          =     .90374
```

DV_Compliance_SC7	Coef.	Robust Std. Err.	t	P>|t|	[95% Conf.	Interval]
1.wave_d1	-.2830091	.1363008	-2.08	0.038	-.5502662	-.015752
1.wave_d2	-.2897053	.1382359	-2.10	0.036	-.5607566	-.0186539
Age	.0034851	.001442	2.42	0.016	.0006578	.0063125
1.Gender_Female	.118353	.03547	3.34	0.001	.0488038	.1879021
1.Minority	-.0061541	.0388344	-0.16	0.874	-.0823002	.069992
Education	.0349213	.0121172	2.88	0.004	.011162	.0586805
1.Employed	.0138563	.040419	0.34	0.732	-.0653968	.0931094
1.Corona_care	-.0494658	.0703775	-0.70	0.482	-.1874612	.0885296
1.Insurance_Public	.0921238	.0601054	1.53	0.125	-.0257302	.2099778
1.Insurance_Private	.1342208	.0626147	2.14	0.032	.0114466	.256995
SES_before	.0049111	.0103502	0.47	0.635	-.0153834	.0252056
SES_change	.0001864	.0124257	0.01	0.988	-.0241778	.0245505
2.Health_self	.0324131	.0371546	0.87	0.383	-.0404392	.1052654
2.Health_other	-.0171484	.0377876	-0.45	0.650	-.0912419	.0569451
1.Conservative_01	.0484979	.040831	1.19	0.235	-.0315631	.1285588
1.Conservative_other	.10732	.0615514	1.74	0.081	-.0133692	.2280093
1.GeoCensus_d1	-.0989635	.0518503	-1.91	0.056	-.2006309	.0027039
1.GeoCensus_d2	-.124625	.0441401	-2.82	0.005	-.2111743	-.0380756
1.GeoCensus_d3	-.0011503	.0514175	-0.02	0.982	-.1019692	.0996686
Current_measures						
Yes	.1769751	.0636957	2.78	0.005	.0520813	.3018688
Measures_clear	-.0426031	.0177624	-2.40	0.017	-.0774313	-.0077748
MA_Perc_Threat_SC3	.1260254	.019741	6.38	0.000	.0873175	.1647333
Costs_SC5	.0267555	.0126487	2.12	0.034	.0019541	.0515569
Deterr_SD_Likely_SC2	.0058913	.0122829	0.48	0.632	-.0181928	.0299755
Deterr_SD_Severe	-.0042312	.0111516	-0.38	0.704	-.0260971	.0176348
MA_MoralBelief	.2461499	.0258713	9.51	0.000	.1954217	.296878
MA_Authority_SC2	-.0082445	.0108723	-0.76	0.448	-.0295627	.0130738
NOO_SC3	.0175807	.0257193	0.68	0.494	-.0328493	.0680108
NNOO_SC3	.0206639	.021459	0.96	0.336	-.0214126	.0627404
OOL_SC12	.015487	.0146545	1.06	0.291	-.0132474	.0442214
PJE_SC4	-.0090301	.0120353	-0.75	0.453	-.0326289	.0145686
Trust_Science_SC4	.0379403	.0234886	1.62	0.106	-.0081159	.0839964
Trust_in_media	-.0132158	.0151105	-0.87	0.382	-.0428443	.0164127
Impulsivity_SC4	-.0853015	.0187278	-4.55	0.000	-.1220228	-.0485803
NegEmo_SC6	.0175669	.0128981	1.36	0.173	-.0077236	.0428574
SN_SC7	.0293652	.0146376	2.01	0.045	.000664	.0580664
CTC_SC7	.5163847	.0319602	16.16	0.000	.4537175	.5790519
OTC_SC7	-.0264225	.0097549	-2.71	0.007	-.0455498	-.0072952
Measures_clear	0	(omitted)				
wave_d1#c.Measures_clear						
1	.0373439	.024407	1.53	0.126	-.0105131	.0852009
Measures_clear	0	(omitted)				
wave_d2#c.Measures_clear						
1	.0280233	.0249934	1.12	0.262	-.0209836	.0770301
_cons	.0211011	.2275887	0.09	0.926	-.4251523	.4673545

```
302 .
303 .
304 . *****************
305 .
306 . *11. Model 11: Perceived threat x Survey Wave
```

```
307 .
308 . *11.a.2 Regression
309 . reg DV_Compliance_SC7 i.wave_d1 i.wave_d2 Age i.Gender_Female i.Minority Education i.Employed i.Corona_care i.Insura
    > rvative_01 i.Conservative_other i.GeoCensus_d1 i.GeoCensus_d2 i.GeoCensus_d3 i.Current_measures Measures_clear MA_Pe
    > _SC2 NOO_SC3 NNOO_SC3 OOL_SC12 PJE_SC4 Trust_Science_SC4 Trust_in_media Impulsivity_SC4 NegEmo_SC6 SN_SC7 CTC_SC7 OT
    > s == 1
    note: MA_Perc_Threat_SC3 omitted because of collinearity
    note: MA_Perc_Threat_SC3 omitted because of collinearity

          Source |       SS           df       MS      Number of obs   =     2,919
    -------------+----------------------------------   F(40, 2878)     =     78.08
           Model |  2550.33933        40  63.7584831   Prob > F        =    0.0000
        Residual |  2350.19117     2,878  .81660569   R-squared       =    0.5204
    -------------+----------------------------------   Adj R-squared   =    0.5138
           Total |   4900.5305     2,918  1.67941415   Root MSE        =    .90366

    ------------------------------------------------------------------------------------------
                  DV_Compliance_SC7 |      Coef.   Std. Err.      t    P>|t|     [95% Conf. Interval]
    --------------------------------+---------------------------------------------------------
                          1.wave_d1 |   -.326159   .1554219    -2.10   0.036    -.6309085   -.0214095
                          1.wave_d2 |   -.363486   .1648022    -2.21   0.027    -.6866282   -.0403438
                                Age |   .0034731   .0014407     2.41   0.016     .0006482     .006298
                    1.Gender_Female |   .1188399   .0353795     3.36   0.001     .0494682    .1882116
                         1.Minority |  -.0053578   .0377932    -0.14   0.887    -.0794622    .0687466
                          Education |   .0342646   .0122919     2.79   0.005     .0101629    .0583663
                         1.Employed |   .0125997    .039219     0.32   0.748    -.0643004    .0894998
                      1.Corona_care |  -.0463198   .0653203    -0.71   0.478    -.1743992    .0817595
                 1.Insurance_Public |   .0939741   .0538089     1.75   0.081    -.0115338    .1994819
                1.Insurance_Private |   .1347582   .0562254     2.40   0.017      .024512    .2450043
                         SES_before |   .0050984   .0095605     0.53   0.594    -.0136477    .0238445
                         SES_change |  -.0002058   .0112385    -0.02   0.985    -.0222421    .0218305
                      2.Health_self |   .0325502   .0411276     0.79   0.429    -.0480924    .1131928
                     2.Health_other |  -.0167881   .0390913    -0.43   0.668    -.0934379    .0598618
                  1.Conservative_01 |   .0463954    .040391     1.15   0.251    -.0328029    .1255937
               1.Conservative_other |   .1063929   .0578862     1.84   0.066    -.0071097    .2198956
                     1.GeoCensus_d1 |   -.097855   .0530797    -1.84   0.065     -.201933    .0062229
                     1.GeoCensus_d2 |  -.1246265   .0460478    -2.71   0.007    -.2149165   -.0343366
                     1.GeoCensus_d3 |   .0000615   .0565361     0.00   0.999    -.1107938    .1109168
                                    |
                   Current_measures |
                               Yes  |   .1778511   .0523744     3.40   0.001     .0751559    .2805462
                     Measures_clear |  -.0197559   .0115195    -1.71   0.086    -.0423433    .0028315
                 MA_Perc_Threat_SC3 |   .0987843   .0231355     4.27   0.000     .0534204    .1441481
                          Costs_SC5 |   .0269373   .0122669     2.20   0.028     .0028846      .05099
               Deterr_SD_Likely_SC2 |   .0054607   .0120601     0.45   0.651    -.0181865     .029108
                   Deterr_SD_Severe |  -.0035773   .0106281    -0.34   0.736    -.0244168    .0172622
                     MA_MoralBelief |    .244952    .019494    12.57   0.000     .2067283    .2831757
                   MA_Authority_SC2 |  -.0079993   .0112743    -0.71   0.478    -.0301058    .0141072
                            NOO_SC3 |   .0185529    .024684     0.75   0.452    -.0298473     .066953
                           NNOO_SC3 |   .0209294   .0210987     0.99   0.321    -.0204407    .0622995
                           OOL_SC12 |   .0161116   .0142104     1.13   0.257     -.011752    .0439752
                            PJE_SC4 |  -.0099866   .0121599    -0.82   0.412    -.0338295    .0138563
                  Trust_Science_SC4 |   .0361757   .0220599     1.64   0.101    -.0070791    .0794306
                     Trust_in_media |  -.0122489    .015746    -0.78   0.437    -.0431234    .0186256
                    Impulsivity_SC4 |  -.0852266    .019049    -4.47   0.000    -.1225776   -.0478756
                         NegEmo_SC6 |   .0175314   .0126326     1.39   0.165    -.0072384    .0423012
                             SN_SC7 |   .0295274   .0145887     2.02   0.043     .0009221    .0581327
                            CTC_SC7 |     .51577   .0216449    23.83   0.000      .473329     .558211
                            OTC_SC7 |  -.0264865   .0102054    -2.60   0.009    -.0464972   -.0064758
                 MA_Perc_Threat_SC3 |          0  (omitted)
                                    |
    wave_d1#c.MA_Perc_Threat_SC3    |
                                 1  |   .0430481   .0269378     1.60   0.110    -.0097712    .0958674
                                    |
                 MA_Perc_Threat_SC3 |          0  (omitted)
                                    |
    wave_d2#c.MA_Perc_Threat_SC3    |
                                 1  |   .0393768   .0280692     1.40   0.161     -.015661    .0944145
                                    |
                              _cons |   .0606249   .2192515     0.28   0.782     -.369281    .4905308
    ------------------------------------------------------------------------------------------
```

```
310 .
311 . *11.a.3 Check hettest: Run this right after your regression to apply the Breusch-Pagan / Cook-Weisberg test for hete
312 . *if significant, then you need to run the regression with vce(ro) at the end
313 . estat hettest

    Breusch-Pagan / Cook-Weisberg test for heteroskedasticity
             Ho: Constant variance
             Variables: fitted values of DV_Compliance_SC7

             chi2(1)      =   371.19
             Prob > chi2  =   0.0000

314 .
315 . *11.a.4. check vif, to check for for multicollinearity (VIFs >10 are problematic)
316 . vif
```

Variable	VIF	1/VIF
1.wave_d1	19.31	0.051774
1.wave_d2	20.97	0.047694
Age	1.26	0.791189
1.Gender_F~e	1.11	0.901425
1.Minority	1.15	0.869235
Education	1.29	0.778113
1.Employed	1.27	0.788378
1.Corona_c~e	1.22	0.819481
1.Insuranc~c	2.54	0.393767
1.Insuran~te	2.35	0.426399
SES_before	1.37	0.728514
SES_change	1.24	0.809502
2.Health_s~f	1.36	0.737562
2.Health_o~r	1.33	0.753424
1.Conserv~01	1.44	0.692236
1.Conserva~r	1.26	0.791159
1.GeoCensu~1	1.66	0.603590
1.GeoCensu~2	1.86	0.538952
1.GeoCensu~3	1.54	0.650139
1.Current_~s	1.14	0.877237
Measures_c~r	1.42	0.706520
MA_Perc_Th~3	4.34	0.230341
Costs_SC5	1.45	0.691559
Deterr_SD_~2	1.62	0.619170
Deterr_SD_~e	1.19	0.839089
MA_MoralBe~f	2.28	0.438660
MA_Authori~2	1.62	0.615897
NOO_SC3	1.77	0.565539
NNOO_SC3	1.58	0.631280
OOL_SC12	1.59	0.630408
PJE_SC4	1.38	0.726896
Trust_Scie~4	1.69	0.590785
Trust_in_m~a	1.52	0.656160
Impulsivi~C4	1.64	0.610356
NegEmo_SC6	1.40	0.712958
SN_SC7	1.48	0.674703
CTC_SC7	1.64	0.609716
OTC_SC7	1.14	0.874439
wave_d1# c. MA_Perc_Th~3 1	19.88	0.050307
wave_d2# c. MA_Perc_Th~3 1	22.01	0.045441
Mean VIF	3.48	

```
317 .
318 . *11.a.5. Effect size
319 . estat esize
```

Effect sizes for linear models

Source	Eta-Squared	df	[95% Conf.	Interval]
Model	.5204211	40	.4912121	.5352044
wave_d1	.0015278	1	.	.0056883
wave_d2	.0016874	1	.	.0059907
Age	.0020153	1	.0000572	.0065917
Gender_Female	.0039051	1	.000677	.0097338
Minority	6.98e-06	1	.	.0010518
Education	.0026927	1	.0002354	.0077689
Employed	.0000359	1	.	.0016572
Corona_care	.0001747	1	.	.0024558
Insurance_Public	.0010587	1	.	.0047482
Insurance_Private	.001992	1	.000052	.0065498
SES_before	.0000988	1	.	.0021155
SES_change	1.17e-07	1	.	.
Health_self	.0002176	1	.	.0026152
Health_other	.0000641	1	.	.0019042
Conservative_01	.0004582	1	.	.0033441
Conservative_other	.0011724	1	.	.0049846
GeoCensus_d1	.0011795	1	.	.0049992
GeoCensus_d2	.0025387	1	.0001901	.0075074
GeoCensus_d3	4.11e-10	1	.	.
Current_measures	.0039907	1	.000713	.0098675
Measures_clear	.0010209	1	.	.0046683
MA_Perc_Threat_SC3	.0181556	1	.0097513	.0289336
Costs_SC5	.0016727	1	.	.0059631
Deterr_SD_Likely_SC2	.0000712	1	.	.0019531
Deterr_SD_Severe	.0000394	1	.	.0016949
MA_MoralBelief	.0520082	1	.0373974	.0684838
MA_Authority_SC2	.0001749	1	.	.0024566
NOO_SC3	.0001963	1	.	.0025379
NNOO_SC3	.0003418	1	.	.0030153
OOL_SC12	.0004465	1	.	.0033122
PJE_SC4	.0002343	1	.	.0026735
Trust_Science_SC4	.0009335	1	.	.0044802
Trust_in_media	.0002102	1	.	.0025889
Impulsivity_SC4	.0069073	1	.0021833	.0141833
NegEmo_SC6	.0006688	1	.	.0038768
SN_SC7	.0014214	1	.	.0054823
CTC_SC7	.1647823	1	.1413343	.1886949
OTC_SC7	.002335	1	.0001346	.0071564
MA_Perc_Threat_SC3	.0181556	1	.0097513	.0289336
wave_d1#c.MA_Perc_Threat_SC3	.0008866	1	.	.0043771
MA_Perc_Threat_SC3	.0181556	1	.0097513	.0289336
wave_d2#c.MA_Perc_Threat_SC3	.0006833	1	.	.0039117

Note: Eta-Squared values for individual model terms are partial.

```
320 .
321 . *11.a.6 Regression with vce(ro)
322 . reg DV_Compliance_SC7 i.wave_d1 i.wave_d2 Age i.Gender_Female i.Minority Education i.Employed i.Corona_care i.Insura
    > rvative_01 i.Conservative_other i.GeoCensus_d1 i.GeoCensus_d2 i.GeoCensus_d3 i.Current_measures Measures_clear MA_Pe
    > _SC2 NOO_SC3 NNOO_SC3 OOL_SC12 PJE_SC4 Trust_Science_SC4 Trust_in_media Impulsivity_SC4 NegEmo_SC6 SN_SC7 CTC_SC7 OT
    > s == 1, vce(ro)
    note: MA_Perc_Threat_SC3 omitted because of collinearity
    note: MA_Perc_Threat_SC3 omitted because of collinearity

    Linear regression                               Number of obs     =      2,919
                                                    F(40, 2878)       =      67.65
                                                    Prob > F          =     0.0000
                                                    R-squared         =     0.5204
                                                    Root MSE          =     .90366
```

DV_Compliance_SC7	Coef.	Robust Std. Err.	t	P>|t|	[95% Conf.	Interval]
1.wave_d1	-.326159	.1934029	-1.69	0.092	-.7053813	.0530633
1.wave_d2	-.363486	.1950464	-1.86	0.062	-.7459307	.0189587
Age	.0034731	.0014355	2.42	0.016	.0006583	.0062879
1.Gender_Female	.1188399	.0353928	3.36	0.001	.0494421	.1882377
1.Minority	-.0053578	.0389243	-0.14	0.891	-.0816801	.0709645
Education	.0342646	.012147	2.82	0.005	.0104469	.0580823
1.Employed	.0125997	.0404127	0.31	0.755	-.0666411	.0918404
1.Corona_care	-.0463198	.0702312	-0.66	0.510	-.1840284	.0913888
1.Insurance_Public	.0939741	.0600291	1.57	0.118	-.0237304	.2116785
1.Insurance_Private	.1347582	.0625226	2.16	0.031	.0121646	.2573518
SES_before	.0050984	.0103758	0.49	0.623	-.0152464	.0254432
SES_change	-.0002058	.0123979	-0.02	0.987	-.0245155	.0241039
2.Health_self	.0325502	.0369591	0.88	0.379	-.0399189	.1050192
2.Health_other	-.0167881	.0377522	-0.44	0.657	-.0908122	.057236
1.Conservative_01	.0463954	.0410255	1.13	0.258	-.0340469	.1268377
1.Conservative_other	.1063929	.0616556	1.73	0.085	-.0145006	.2272865
1.GeoCensus_d1	-.097855	.0518255	-1.89	0.059	-.1994739	.0037638
1.GeoCensus_d2	-.1246265	.044125	-2.82	0.005	-.2111462	-.0381068
1.GeoCensus_d3	.0000615	.0514087	0.00	0.999	-.10074	.1008631
Current_measures						
Yes	.1778511	.0637886	2.79	0.005	.0527752	.302927
Measures_clear	-.0197559	.0121798	-1.62	0.105	-.0436379	.0041261
MA_Perc_Threat_SC3	.0987843	.0259622	3.80	0.000	.0478779	.1496906
Costs_SC5	.0269373	.0126058	2.14	0.033	.0022201	.0516546
Deterr_SD_Likely_SC2	.0054607	.0122525	0.45	0.656	-.0185639	.0294854
Deterr_SD_Severe	-.0035773	.0111309	-0.32	0.748	-.0254026	.018248
MA_MoralBelief	.244952	.0259225	9.45	0.000	.1941234	.2957806
MA_Authority_SC2	-.0079993	.0108542	-0.74	0.461	-.0292821	.0132835
NOO_SC3	.0185529	.0258182	0.72	0.472	-.0320712	.0691769
NNOO_SC3	.0209294	.0214338	0.98	0.329	-.0210977	.0629566
OOL_SC12	.0161116	.0146956	1.10	0.273	-.0127033	.0449265
PJE_SC4	-.0099866	.0119871	-0.83	0.405	-.0334907	.0135176
Trust_Science_SC4	.0361757	.0236	1.53	0.125	-.010099	.0824504
Trust_in_media	-.0122489	.0150123	-0.82	0.415	-.0416847	.017187
Impulsivity_SC4	-.0852266	.0187566	-4.54	0.000	-.1220043	-.0484489
NegEmo_SC6	.0175314	.0129161	1.36	0.175	-.0077944	.0428572
SN_SC7	.0295274	.0146563	2.01	0.044	.0007895	.0582654
CTC_SC7	.51577	.0320415	16.10	0.000	.4529434	.5785966
OTC_SC7	-.0264865	.0097616	-2.71	0.007	-.0456269	-.0073461
MA_Perc_Threat_SC3	0	(omitted)				
wave_d1#c.MA_Perc_Threat_SC3						
1	.0430481	.0322641	1.33	0.182	-.0202149	.1063111
MA_Perc_Threat_SC3	0	(omitted)				
wave_d2#c.MA_Perc_Threat_SC3						
1	.0393768	.0323676	1.22	0.224	-.0240892	.1028428
_cons	.0606249	.2426224	0.25	0.803	-.4151064	.5363562

```
323 .
324 .
325 . *****************
326 .
327 . *12. Model 12: Costs x Survey Wave
```

```
328 .
329 . *12.a.2 Regression
330 . reg DV_Compliance_SC7 i.wave_d1 i.wave_d2 Age i.Gender_Female i.Minority Education i.Employed i.Corona_care i.Insura
    > rvative_01 i.Conservative_other i.GeoCensus_d1 i.GeoCensus_d2 i.GeoCensus_d3 i.Current_measures Measures_clear MA_Pe
    > _SC2 NOO_SC3 NNOO_SC3 OOL_SC12 PJE_SC4 Trust_Science_SC4 Trust_in_media Impulsivity_SC4 NegEmo_SC6 SN_SC7 CTC_SC7 OT
    note: Costs_SC5 omitted because of collinearity
    note: Costs_SC5 omitted because of collinearity
```

Source	SS	df	MS
Model	2549.43688	40	63.735922
Residual	2351.09362	2,878	.816919256
Total	4900.5305	2,918	1.67941415

Number of obs	=	2,919
F(40, 2878)	=	78.02
Prob > F	=	0.0000
R-squared	=	0.5202
Adj R-squared	=	0.5136
Root MSE	=	.90384

DV_Compliance_SC7	Coef.	Std. Err.	t	P>|t|	[95% Conf.	Interval]
1.wave_d1	-.1040376	.1124475	-0.93	0.355	-.3245234	.1164482
1.wave_d2	-.2781679	.1155317	-2.41	0.016	-.5047011	-.0516346
Age	.0034226	.0014418	2.37	0.018	.0005956	.0062497
1.Gender_Female	.1199283	.0353817	3.39	0.001	.0505522	.1893044
1.Minority	-.0061128	.0378333	-0.16	0.872	-.0802959	.0680703
Education	.0342892	.0122882	2.79	0.005	.0101946	.0583838
1.Employed	.0137097	.0392168	0.35	0.727	-.0631862	.0906056
1.Corona_care	-.0448786	.0653552	-0.69	0.492	-.1730264	.0832691
1.Insurance_Public	.0901805	.0537933	1.68	0.094	-.0152969	.1956578
1.Insurance_Private	.1326146	.056231	2.36	0.018	.0223574	.2428717
SES_before	.0053195	.0095645	0.56	0.578	-.0134345	.0240734
SES_change	-7.70e-06	.0112444	-0.00	0.999	-.0220557	.0220403
2.Health_self	.0339325	.0411173	0.83	0.409	-.0466897	.1145548
2.Health_other	-.0197246	.0390793	-0.50	0.614	-.0963507	.0569016
1.Conservative_01	.0486348	.0403607	1.21	0.228	-.030504	.1277735
1.Conservative_other	.1060277	.0578991	1.83	0.067	-.0075002	.2195556
1.GeoCensus_d1	-.0974832	.053098	-1.84	0.066	-.2015972	.0066308
1.GeoCensus_d2	-.1257921	.0460614	-2.73	0.006	-.2161088	-.0354755
1.GeoCensus_d3	-.0009223	.0565601	-0.02	0.987	-.1118246	.1099801
Current_measures						
Yes	.1812307	.0523548	3.46	0.001	.078574	.2838873
Measures_clear	-.0198403	.0115287	-1.72	0.085	-.0424457	.0027651
MA_Perc_Threat_SC3	.1260082	.0171987	7.33	0.000	.0922851	.1597313
Costs_SC5	.0147882	.0188792	0.78	0.434	-.0222299	.0518062
Deterr_SD_Likely_SC2	.0054177	.0120638	0.45	0.653	-.0182368	.0290723
Deterr_SD_Severe	-.0035386	.0106361	-0.33	0.739	-.0243938	.0173166
MA_MoralBelief	.2464488	.0194737	12.66	0.000	.2082649	.2846327
MA_Authority_SC2	-.0084912	.0112729	-0.75	0.451	-.030595	.0136127
NOO_SC3	.0168347	.0246747	0.68	0.495	-.0315472	.0652167
NNOO_SC3	.0203413	.0210967	0.96	0.335	-.0210249	.0617074
OOL_SC12	.0158517	.014211	1.12	0.265	-.012013	.0437164
PJE_SC4	-.0096642	.0121635	-0.79	0.427	-.0335143	.0141859
Trust_Science_SC4	.0372607	.0220471	1.69	0.091	-.005969	.0804904
Trust_in_media	-.0130709	.0157575	-0.83	0.407	-.043968	.0178263
Impulsivity_SC4	-.0857041	.0190524	-4.50	0.000	-.1230619	-.0483463
NegEmo_SC6	.0176664	.0126347	1.40	0.162	-.0071076	.0424404
SN_SC7	.0306467	.0146208	2.10	0.036	.0019785	.0593149
CTC_SC7	.5150173	.0216555	23.78	0.000	.4725554	.5574792
OTC_SC7	-.0256669	.0102176	-2.51	0.012	-.0457014	-.0056324
Costs_SC5	0	(omitted)				
wave_d1#c.Costs_SC5						
1	.0037746	.0248756	0.15	0.879	-.0450011	.0525503
Costs_SC5	0	(omitted)				
wave_d2#c.Costs_SC5						
1	.0325257	.0254628	1.28	0.202	-.0174016	.0824529
_cons	-.0444975	.2082533	-0.21	0.831	-.4528381	.3638432

```
.
. *12.a.3 Check hettest: Run this right after your regression to apply the Breusch-Pagan / Cook-Weisberg test for hete
. *if significant, then you need to run the regression with vce(ro) at the end
. estat hettest

Breusch-Pagan / Cook-Weisberg test for heteroskedasticity
         Ho: Constant variance
         Variables: fitted values of DV_Compliance_SC7

         chi2(1)      =   365.48
         Prob > chi2  =   0.0000

.
. *12.a.4. check vif, to check for for multicollinearity (VIFs >10 are problematic)
. vif
```

Variable	VIF	1/VIF
1.wave_d1	10.11	0.098947
1.wave_d2	10.30	0.097086
Age	1.27	0.790256
1.Gender_F~e	1.11	0.901656
1.Minority	1.15	0.867725
Education	1.28	0.778872
1.Employed	1.27	0.788768
1.Corona_c~e	1.22	0.818921
1.Insuranc~c	2.54	0.394146
1.Insuran~te	2.34	0.426478
SES_before	1.37	0.728185
SES_change	1.24	0.808956
2.Health_s~f	1.35	0.738218
2.Health_o~r	1.33	0.754180
1.Conserv~01	1.44	0.693544
1.Conserva~r	1.26	0.791111
1.GeoCensu~1	1.66	0.603404
1.GeoCensu~2	1.86	0.538840
1.GeoCensu~3	1.54	0.649837
1.Current_~s	1.14	0.878233
Measures_c~r	1.42	0.705666
MA_Perc_Th~3	2.40	0.416969
Costs_SC5	3.42	0.292077
Deterr_SD_~2	1.62	0.619026
Deterr_SD_~e	1.19	0.838150
MA_MoralBe~f	2.27	0.439744
MA_Authori~2	1.62	0.616279
NOO_SC3	1.77	0.566183
NNOO_SC3	1.58	0.631644
OOL_SC12	1.59	0.630602
PJE_SC4	1.38	0.726735
Trust_Scie~4	1.69	0.591699
Trust_in_m~a	1.53	0.655448
Impulsivi~C4	1.64	0.610368
NegEmo_SC6	1.40	0.712989
SN_SC7	1.49	0.672003
CTC_SC7	1.64	0.609350
OTC_SC7	1.15	0.872699
wave_d1# c.Costs_SC5 1	10.32	0.096894
wave_d2# c.Costs_SC5 1	10.57	0.094593
Mean VIF	2.46	

```
338 .
339 . *12.a.5. Effect size
340 . estat esize

    Effect sizes for linear models
```

Source	Eta-Squared	df	[95% Conf.	Interval]
Model	.5202369	40	.4910197	.5350237
wave_d1	.0002973	1	.	.0028798
wave_d2	.0020102	1	.0000561	.0065826
Age	.0019542	1	.0000437	.0064815
Gender_Female	.0039762	1	.0007069	.0098449
Minority	9.07e-06	1	.	.0011446
Education	.0026982	1	.000237	.007778
Employed	.0000425	1	.	.001726
Corona_care	.0001638	1	.	.0024127
Insurance_Public	.0009756	1	.	.0045713
Insurance_Private	.0019289	1	.0000382	.0064356
SES_before	.0001075	1	.	.0021605
SES_change	1.63e-10	1	.	.
Health_self	.0002366	1	.	.0026813
Health_other	.0000885	1	.	.0020588
Conservative_01	.0005043	1	.	.0034662
Conservative_other	.0011639	1	.	.004967
GeoCensus_d1	.0011698	1	.	.0049792
GeoCensus_d2	.0025847	1	.0002034	.0075859
GeoCensus_d3	9.24e-08	1	.	.
Current_measures	.0041463	1	.0007799	.0101093
Measures_clear	.001028	1	.	.0046834
MA_Perc_Threat_SC3	.01831	1	.0098657	.0291255
Costs_SC5	.0018356	1	.0000185	.0062655
Deterr_SD_Likely_SC2	.0000701	1	.	.0019454
Deterr_SD_Severe	.0000385	1	.	.0016854
MA_MoralBelief	.0527162	1	.0380093	.0692777
MA_Authority_SC2	.0001971	1	.	.002541
NOO_SC3	.0001617	1	.	.0024042
NNOO_SC3	.0003229	1	.	.0029586
OOL_SC12	.0004321	1	.	.0032731
PJE_SC4	.0002193	1	.	.0026212
Trust_Science_SC4	.0009915	1	.	.0046055
Trust_in_media	.000239	1	.	.0026897
Impulsivity_SC4	.0069818	1	.0022255	.014289
NegEmo_SC6	.0006789	1	.	.003901
SN_SC7	.0015243	1	.	.0056815
CTC_SC7	.1642456	1	.1408204	.1881409
OTC_SC7	.0021878	1	.0000976	.0068988
Costs_SC5	.0018356	1	.0000185	.0062655
wave_d1#c.Costs_SC5	8.00e-06	1	.	.0011
Costs_SC5	.0018356	1	.0000185	.0062655
wave_d2#c.Costs_SC5	.0005666	1	.	.0036261

```
    Note: Eta-Squared values for individual model terms are partial.

341 .
342 . *12.a.6 Regression with vce(ro)
343 . reg DV_Compliance_SC7 i.wave_d1 i.wave_d2 Age i.Gender_Female i.Minority Education i.Employed i.Corona_care i.Insura
    > rvative_01 i.Conservative_other i.GeoCensus_d1 i.GeoCensus_d2 i.GeoCensus_d3 i.Current_measures Measures_clear MA_Pe
    > _SC2 NOO_SC3 NNOO_SC3 OOL_SC12 PJE_SC4 Trust_Science_SC4 Trust_in_media Impulsivity_SC4 NegEmo_SC6 SN_SC7 CTC_SC7 OT
    note: Costs_SC5 omitted because of collinearity
    note: Costs_SC5 omitted because of collinearity

    Linear regression                               Number of obs     =      2,919
                                                    F(40, 2878)       =      67.25
                                                    Prob > F          =     0.0000
                                                    R-squared         =     0.5202
                                                    Root MSE          =     .90384
```

DV_Compliance_SC7	Coef.	Robust Std. Err.	t	P>|t|	[95% Conf.	Interval]
1.wave_d1	-.1040376	.1141543	-0.91	0.362	-.3278699	.1197948
1.wave_d2	-.2781679	.1267192	-2.20	0.028	-.5266375	-.0296983
Age	.0034226	.0014377	2.38	0.017	.0006037	.0062416
1.Gender_Female	.1199283	.0354441	3.38	0.001	.0504298	.1894268
1.Minority	-.0061128	.0389387	-0.16	0.875	-.0824634	.0702379
Education	.0342892	.0120492	2.85	0.004	.0106633	.0579151
1.Employed	.0137097	.0404344	0.34	0.735	-.0655736	.0929931
1.Corona_care	-.0448786	.0701843	-0.64	0.523	-.1824951	.0927378
1.Insurance_Public	.0901805	.0600688	1.50	0.133	-.0276016	.2079626
1.Insurance_Private	.1326146	.0625643	2.12	0.034	.0099391	.25529
SES_before	.0053195	.0103374	0.51	0.607	-.0149501	.025589
SES_change	-7.70e-06	.0124285	-0.00	1.000	-.0243774	.024362
2.Health_self	.0339325	.0369258	0.92	0.358	-.0384712	.1063363
2.Health_other	-.0197246	.0377849	-0.52	0.602	-.0938127	.0543635
1.Conservative_01	.0486348	.0408591	1.19	0.234	-.0314814	.1287509
1.Conservative_other	.1060277	.0616157	1.72	0.085	-.0147876	.2268431
1.GeoCensus_d1	-.0974832	.0518057	-1.88	0.060	-.1990632	.0040968
1.GeoCensus_d2	-.1257921	.0441717	-2.85	0.004	-.2124035	-.0391808
1.GeoCensus_d3	-.0009223	.0515363	-0.02	0.986	-.1019742	.1001296
Current_measures						
Yes	.1812307	.0637931	2.84	0.005	.056146	.3063153
Measures_clear	-.0198403	.0122224	-1.62	0.105	-.0438059	.0041253
MA_Perc_Threat_SC3	.1260082	.0196725	6.41	0.000	.0874345	.1645819
Costs_SC5	.0147882	.0184044	0.80	0.422	-.0212989	.0508753
Deterr_SD_Likely_SC2	.0054177	.0122471	0.44	0.658	-.0185962	.0294317
Deterr_SD_Severe	-.0035386	.0110987	-0.32	0.750	-.0253008	.0182236
MA_MoralBelief	.2464488	.0258779	9.52	0.000	.1957077	.2971899
MA_Authority_SC2	-.0084912	.0108854	-0.78	0.435	-.0298352	.0128529
NOO_SC3	.0168347	.0257035	0.65	0.513	-.0335644	.0672338
NNOO_SC3	.0203413	.0215244	0.95	0.345	-.0218636	.0625461
OOL_SC12	.0158517	.014738	1.08	0.282	-.0130464	.0447498
PJE_SC4	-.0096642	.0119943	-0.81	0.420	-.0331825	.0138541
Trust_Science_SC4	.0372607	.023521	1.58	0.113	-.0088591	.0833805
Trust_in_media	-.0130709	.0150956	-0.87	0.387	-.0426702	.0165285
Impulsivity_SC4	-.0857041	.0187599	-4.57	0.000	-.1224883	-.0489198
NegEmo_SC6	.0176664	.0128979	1.37	0.171	-.0076236	.0429564
SN_SC7	.0306467	.0146577	2.09	0.037	.0019061	.0593873
CTC_SC7	.5150173	.0319796	16.10	0.000	.4523121	.5777225
OTC_SC7	-.0256669	.0097274	-2.64	0.008	-.0447403	-.0065935
Costs_SC5	0	(omitted)				
wave_d1#c.Costs_SC5						
1	.0037746	.0250355	0.15	0.880	-.0453146	.0528639
Costs_SC5	0	(omitted)				
wave_d2#c.Costs_SC5						
1	.0325257	.0275612	1.18	0.238	-.0215161	.0865674
_cons	-.0444975	.2249071	-0.20	0.843	-.4854927	.3964978

```
.
.
. *****************
.
. *13. Model 13: Punish certainty x Survey Wave
```

```
349 .
350 . *13.a.2 Regression
351 . reg DV_Compliance_SC7 i.wave_d1 i.wave_d2 Age i.Gender_Female i.Minority Education i.Employed i.Corona_care i.Insura
    > rvative_01 i.Conservative_other i.GeoCensus_d1 i.GeoCensus_d2 i.GeoCensus_d3 i.Current_measures Measures_clear MA_Pe
    > _SC2 NOO_SC3 NNOO_SC3 OOL_SC12 PJE_SC4 Trust_Science_SC4 Trust_in_media Impulsivity_SC4 NegEmo_SC6 SN_SC7 CTC_SC7 OT
    > _reqs == 1
    note: Deterr_SD_Likely_SC2 omitted because of collinearity
    note: Deterr_SD_Likely_SC2 omitted because of collinearity
```

Source	SS	df	MS			
				Number of obs	=	2,919
				F(40, 2878)	=	77.94
Model	2548.23862	40	63.7059656	Prob > F	=	0.0000
Residual	2352.29188	2,878	.817335607	R-squared	=	0.5200
				Adj R-squared	=	0.5133
Total	4900.5305	2,918	1.67941415	Root MSE	=	.90407

DV_Compliance_SC7	Coef.	Std. Err.	t	P>|t|	[95% Conf.	Interval]
1.wave_d1	-.1247179	.0855461	-1.46	0.145	-.2924558	.0430199
1.wave_d2	-.1920308	.0892434	-2.15	0.031	-.3670182	-.0170435
Age	.0034862	.0014426	2.42	0.016	.0006575	.0063149
1.Gender_Female	.1206423	.0354148	3.41	0.001	.0512014	.1900833
1.Minority	-.0047805	.0378134	-0.13	0.899	-.0789245	.0693635
Education	.0346381	.0122898	2.82	0.005	.0105405	.0587357
1.Employed	.0145099	.0392384	0.37	0.712	-.0624283	.0914481
1.Corona_care	-.0470788	.0653531	-0.72	0.471	-.1752224	.0810647
1.Insurance_Public	.0910317	.0537948	1.69	0.091	-.0144484	.1965119
1.Insurance_Private	.133931	.056241	2.38	0.017	.0236543	.2442078
SES_before	.0049826	.0095649	0.52	0.602	-.0137722	.0237374
SES_change	.0000753	.0112442	0.01	0.995	-.0219723	.0221229
2.Health_self	.0344477	.0411231	0.84	0.402	-.046186	.1150814
2.Health_other	-.0189494	.0390881	-0.48	0.628	-.0955928	.057694
1.Conservative_01	.0498282	.0403658	1.23	0.217	-.0293207	.128977
1.Conservative_other	.1073127	.0579132	1.85	0.064	-.0062428	.2208682
1.GeoCensus_d1	-.0983342	.0531138	-1.85	0.064	-.2024791	.0058106
1.GeoCensus_d2	-.126239	.046109	-2.74	0.006	-.216649	-.035829
1.GeoCensus_d3	.0002285	.0565757	0.00	0.997	-.1107046	.1111615
Current_measures						
Yes	.1791705	.0523531	3.42	0.001	.0765171	.2818239
Measures_clear	-.0197812	.0115278	-1.72	0.086	-.0423848	.0028224
MA_Perc_Threat_SC3	.1258122	.0171999	7.31	0.000	.0920869	.1595375
Costs_SC5	.0261836	.0122722	2.13	0.033	.0021205	.0502467
Deterr_SD_Likely_SC2	-.0025376	.0174832	-0.15	0.885	-.0368185	.0317434
Deterr_SD_Severe	-.0034529	.0106322	-0.32	0.745	-.0243004	.0173946
MA_MoralBelief	.2462256	.0194846	12.64	0.000	.2080204	.2844307
MA_Authority_SC2	-.0087185	.011326	-0.77	0.441	-.0309263	.0134894
NOO_SC3	.0171408	.0246779	0.69	0.487	-.0312474	.065529
NNOO_SC3	.0204139	.0211378	0.97	0.334	-.0210327	.0618606
OOL_SC12	.0154178	.014212	1.08	0.278	-.0124488	.0432844
PJE_SC4	-.0100455	.0121682	-0.83	0.409	-.0339047	.0138138
Trust_Science_SC4	.0382885	.0220533	1.74	0.083	-.0049535	.0815304
Trust_in_media	-.0126222	.0157711	-0.80	0.424	-.043546	.0183016
Impulsivity_SC4	-.0856429	.0190576	-4.49	0.000	-.1230109	-.0482749
NegEmo_SC6	.0173942	.0126424	1.38	0.169	-.0073949	.0421834
SN_SC7	.0294218	.0146054	2.01	0.044	.0007837	.0580599
CTC_SC7	.5161303	.0216623	23.83	0.000	.4736552	.5586055
OTC_SC7	-.0261436	.0102177	-2.56	0.011	-.0461784	-.0061088
Deterr_SD_Likely_SC2	0	(omitted)				
wave_d1#c.Deterr_SD_Likely_SC2						
1	.0116428	.0229466	0.51	0.612	-.0333507	.0566364
Deterr_SD_Likely_SC2	0	(omitted)				
wave_d2#c.Deterr_SD_Likely_SC2						
1	.0152672	.0237821	0.64	0.521	-.0313644	.0618989
_cons	-.0670316	.2045381	-0.33	0.743	-.4680876	.3340244

```
352 .
353 . *13.a.3 Check hettest: Run this right after your regression to apply the Breusch-Pagan / Cook-Weisberg test for hete
354 . *if significant, then you need to run the regression with vce(ro) at the end
355 . estat hettest

    Breusch-Pagan / Cook-Weisberg test for heteroskedasticity
             Ho: Constant variance
             Variables: fitted values of DV_Compliance_SC7

             chi2(1)      =   367.77
             Prob > chi2  =   0.0000

356 .
357 . *13.a.4. check vif, to check for for multicollinearity (VIFs >10 are problematic)
358 . vif
```

Variable	VIF	1/VIF
1.wave_d1	5.85	0.171051
1.wave_d2	6.14	0.162790
Age	1.27	0.789749
1.Gender_F~e	1.11	0.900431
1.Minority	1.15	0.869083
Education	1.28	0.779074
1.Employed	1.27	0.788302
1.Corona_c~e	1.22	0.819392
1.Insuranc~c	2.54	0.394325
1.Insuran~te	2.34	0.426543
SES_before	1.37	0.728491
SES_change	1.24	0.809398
2.Health_s~f	1.35	0.738384
2.Health_o~r	1.33	0.754224
1.Conserv~01	1.44	0.693720
1.Conserva~r	1.26	0.791129
1.GeoCensu~1	1.66	0.603354
1.GeoCensu~2	1.86	0.538003
1.GeoCensu~3	1.54	0.649808
1.Current_~s	1.14	0.878735
Measures_c~r	1.42	0.706138
MA_Perc_Th~3	2.40	0.417127
Costs_SC5	1.45	0.691579
Deterr_SD_~2	3.39	0.294885
Deterr_SD_~e	1.19	0.839197
MA_MoralBe~f	2.28	0.439479
MA_Authori~2	1.64	0.610830
NOO_SC3	1.77	0.566325
NNOO_SC3	1.59	0.629511
OOL_SC12	1.59	0.630836
PJE_SC4	1.38	0.726550
Trust_Scie~4	1.69	0.591666
Trust_in_m~a	1.53	0.654653
Impulsivi~C4	1.64	0.610347
NegEmo_SC6	1.40	0.712480
SN_SC7	1.48	0.673760
CTC_SC7	1.64	0.609282
OTC_SC7	1.15	0.873115
wave_d1# c. Deterr_SD_~2 1	6.29	0.158934
wave_d2# c. Deterr_SD_~2 1	6.50	0.153920
Mean VIF	2.04	

```
359 .
360 . *13.a.5. Effect size
361 . estat esize

    Effect sizes for linear models
```

Source	Eta-Squared	df	[95% Conf.	Interval]
Model	.5199924	40	.4907642	.5347837
wave_d1	.000738	1	.	.0040403
wave_d2	.0016062	1	.	.0058377
Age	.002025	1	.0000594	.0066091
Gender_Female	.004016	1	.0007238	.0099069
Minority	5.55e-06	1	.	.000971
Education	.0027525	1	.0002537	.0078695
Employed	.0000475	1	.	.001773
Corona_care	.0001803	1	.	.0024776
Insurance_Public	.000994	1	.	.0046109
Insurance_Private	.0019666	1	.0000464	.0065039
SES_before	.0000943	1	.	.0020911
SES_change	1.56e-08	1	.	.
Health_self	.0002438	1	.	.0027057
Health_other	.0000817	1	.	.0020187
Conservative_01	.0005292	1	.	.0035307
Conservative_other	.0011916	1	.	.0050239
GeoCensus_d1	.0011896	1	.	.0050197
GeoCensus_d2	.0025977	1	.0002072	.007608
GeoCensus_d3	5.67e-09	1	.	.
Current_measures	.0040532	1	.0007397	.0099648
Measures_clear	.0010221	1	.	.0046708
MA_Perc_Threat_SC3	.0182518	1	.0098225	.0290532
Costs_SC5	.0015792	1	.	.0057864
Deterr_SD_Likely_SC2	.0002056	1	.	.0025722
Deterr_SD_Severe	.0000366	1	.	.0016659
MA_MoralBelief	.0525703	1	.0378831	.0691142
MA_Authority_SC2	.0002058	1	.	.0025731
NOO_SC3	.0001676	1	.	.0024279
NNOO_SC3	.000324	1	.	.0029618
OOL_SC12	.0004088	1	.	.0032083
PJE_SC4	.0002368	1	.	.0026819
Trust_Science_SC4	.0010463	1	.	.0047221
Trust_in_media	.0002225	1	.	.0026326
Impulsivity_SC4	.0069681	1	.0022178	.0142696
NegEmo_SC6	.0006573	1	.	.0038493
SN_SC7	.001408	1	.	.0054562
CTC_SC7	.1647534	1	.1413066	.1886651
OTC_SC7	.0022696	1	.0001179	.0070424
Deterr_SD_Likely_SC2	.0002056	1	.	.0025722
wave_d1#c.Deterr_SD_Likely_SC2	.0000894	1	.	.0020641
Deterr_SD_Likely_SC2	.0002056	1	.	.0025722
wave_d2#c.Deterr_SD_Likely_SC2	.0001432	1	.	.0023266

```
    Note: Eta-Squared values for individual model terms are partial.

362 .
363 . *13.a.6 Regression with vce(ro)
364 . reg DV_Compliance_SC7 i.wave_d1 i.wave_d2 Age i.Gender_Female i.Minority Education i.Employed i.Corona_care i.Insura
    > rvative_01 i.Conservative_other i.GeoCensus_d1 i.GeoCensus_d2 i.GeoCensus_d3 i.Current_measures Measures_clear MA_Pe
    > _SC2 NOO_SC3 NNOO_SC3 OOL_SC12 PJE_SC4 Trust_Science_SC4 Trust_in_media Impulsivity_SC4 NegEmo_SC6 SN_SC7 CTC_SC7 OT
    > _reqs == 1, vce(ro)
    note: Deterr_SD_Likely_SC2 omitted because of collinearity
    note: Deterr_SD_Likely_SC2 omitted because of collinearity

    Linear regression                               Number of obs     =      2,919
                                                    F(40, 2878)       =      67.37
                                                    Prob > F          =     0.0000
                                                    R-squared         =     0.5200
                                                    Root MSE          =     .90407
```

DV_Compliance_SC7	Coef.	Robust Std. Err.	t	P>|t|	[95% Conf.	Interval]
1.wave_d1	-.1247179	.0782742	-1.59	0.111	-.2781971	.0287613
1.wave_d2	-.1920308	.090234	-2.13	0.033	-.3689605	-.0151011
Age	.0034862	.001444	2.41	0.016	.0006549	.0063175
1.Gender_Female	.1206423	.0355159	3.40	0.001	.0510032	.1902815
1.Minority	-.0047805	.038873	-0.12	0.902	-.0810022	.0714412
Education	.0346381	.0120714	2.87	0.004	.0109687	.0583076
1.Employed	.0145099	.0404093	0.36	0.720	-.0647241	.0937439
1.Corona_care	-.0470788	.0702247	-0.67	0.503	-.1847746	.0906169
1.Insurance_Public	.0910317	.0601431	1.51	0.130	-.0268962	.2089597
1.Insurance_Private	.133931	.0626317	2.14	0.033	.0111235	.2567386
SES_before	.0049826	.010326	0.48	0.629	-.0152645	.0252298
SES_change	.0000753	.0124203	0.01	0.995	-.0242783	.0244288
2.Health_self	.0344477	.036958	0.93	0.351	-.0380191	.1069145
2.Health_other	-.0189494	.0378039	-0.50	0.616	-.0930749	.0551761
1.Conservative_01	.0498282	.0408114	1.22	0.222	-.0301944	.1298508
1.Conservative_other	.1073127	.0615706	1.74	0.081	-.0134143	.2280396
1.GeoCensus_d1	-.0983342	.0519269	-1.89	0.058	-.2001519	.0034835
1.GeoCensus_d2	-.126239	.0442246	-2.85	0.004	-.2129541	-.0395239
1.GeoCensus_d3	.0002285	.0514529	0.00	0.996	-.1006598	.1011167
Current_measures						
Yes	.1791705	.0637085	2.81	0.005	.0542516	.3040895
Measures_clear	-.0197812	.0121594	-1.63	0.104	-.0436232	.0040608
MA_Perc_Threat_SC3	.1258122	.0197453	6.37	0.000	.0870958	.1645286
Costs_SC5	.0261836	.0126375	2.07	0.038	.0014041	.0509631
Deterr_SD_Likely_SC2	-.0025376	.0168046	-0.15	0.880	-.0354879	.0304127
Deterr_SD_Severe	-.0034529	.0111321	-0.31	0.756	-.0252805	.0183747
MA_MoralBelief	.2462256	.0259401	9.49	0.000	.1953625	.2970886
MA_Authority_SC2	-.0087185	.0109596	-0.80	0.426	-.0302079	.012771
NOO_SC3	.0171408	.0257647	0.67	0.506	-.0333783	.0676599
NNOO_SC3	.0204139	.0214646	0.95	0.342	-.0216736	.0625014
OOL_SC12	.0154178	.0147071	1.05	0.295	-.0134197	.0442553
PJE_SC4	-.0100455	.0120265	-0.84	0.404	-.0336268	.0135359
Trust_Science_SC4	.0382885	.0234575	1.63	0.103	-.0077066	.0842836
Trust_in_media	-.0126222	.0151	-0.84	0.403	-.0422301	.0169857
Impulsivity_SC4	-.0856429	.0187764	-4.56	0.000	-.1224595	-.0488262
NegEmo_SC6	.0173942	.0129157	1.35	0.178	-.0079308	.0427193
SN_SC7	.0294218	.0146776	2.00	0.045	.0006422	.0582014
CTC_SC7	.5161303	.0319426	16.16	0.000	.4534976	.578763
OTC_SC7	-.0261436	.0097695	-2.68	0.007	-.0452996	-.0069876
Deterr_SD_Likely_SC2	0	(omitted)				
wave_d1#c.Deterr_SD_Likely_SC2						
1	.0116428	.0226576	0.51	0.607	-.032784	.0560696
Deterr_SD_Likely_SC2	0	(omitted)				
wave_d2#c.Deterr_SD_Likely_SC2						
1	.0152672	.0257742	0.59	0.554	-.0352705	.0658049
_cons	-.0670316	.219219	-0.31	0.760	-.4968736	.3628105

```
.
.
. *****************
.
. *14. Model 14: Punish severity x Survey Wave
```

```
370 .
371 . *14.a.2 Regression
372 . reg DV_Compliance_SC7 i.wave_d1 i.wave_d2 Age i.Gender_Female i.Minority Education i.Employed i.Corona_care i.Insura
    > rvative_01 i.Conservative_other i.GeoCensus_d1 i.GeoCensus_d2 i.GeoCensus_d3 i.Current_measures Measures_clear MA_Pe
    > _SC2 NOO_SC3 NNOO_SC3 OOL_SC12 PJE_SC4 Trust_Science_SC4 Trust_in_media Impulsivity_SC4 NegEmo_SC6 SN_SC7 CTC_SC7 OT
    >  1
    note: Deterr_SD_Severe omitted because of collinearity
    note: Deterr_SD_Severe omitted because of collinearity
```

Source	SS	df	MS
Model	2548.07591	40	63.7018977
Residual	2352.45459	2,878	.817392145
Total	4900.5305	2,918	1.67941415

Number of obs	=	2,919
F(40, 2878)	=	77.93
Prob > F	=	0.0000
R-squared	=	0.5200
Adj R-squared	=	0.5133
Root MSE	=	.9041

DV_Compliance_SC7	Coef.	Std. Err.	t	P>|t|	[95% Conf.	Interval]
1.wave_d1	-.0404348	.0994838	-0.41	0.684	-.2355016	.1546319
1.wave_d2	-.1110186	.1022693	-1.09	0.278	-.311547	.0895098
Age	.0035148	.0014423	2.44	0.015	.0006868	.0063428
1.Gender_Female	.1201508	.0354178	3.39	0.001	.0507041	.1895976
1.Minority	-.0045478	.0378196	-0.12	0.904	-.078704	.0696084
Education	.0349449	.0122848	2.84	0.004	.0108571	.0590327
1.Employed	.013867	.0392279	0.35	0.724	-.0630507	.0907847
1.Corona_care	-.0455711	.065366	-0.70	0.486	-.1737399	.0825978
1.Insurance_Public	.0907621	.0538035	1.69	0.092	-.0147352	.1962594
1.Insurance_Private	.1334902	.0562457	2.37	0.018	.0232043	.2437761
SES_before	.005095	.0095605	0.53	0.594	-.0136512	.0238411
SES_change	.0000276	.0112431	0.00	0.998	-.0220178	.022073
2.Health_self	.0343519	.0411356	0.84	0.404	-.0463062	.1150101
2.Health_other	-.0194687	.0391063	-0.50	0.619	-.0961479	.0572105
1.Conservative_01	.0495817	.0403689	1.23	0.219	-.0295732	.1287365
1.Conservative_other	.1072794	.0579199	1.85	0.064	-.0062892	.2208481
1.GeoCensus_d1	-.0982881	.0531054	-1.85	0.064	-.2024166	.0058404
1.GeoCensus_d2	-.1251484	.0460766	-2.72	0.007	-.2154948	-.0348019
1.GeoCensus_d3	.0008312	.0565612	0.01	0.988	-.1100733	.1117357
Current_measures						
Yes	.1800586	.0523754	3.44	0.001	.0773616	.2827556
Measures_clear	-.0198023	.0115291	-1.72	0.086	-.0424085	.0028039
MA_Perc_Threat_SC3	.125903	.0171986	7.32	0.000	.0921802	.1596259
Costs_SC5	.0261897	.0122686	2.13	0.033	.0021336	.0502458
Deterr_SD_Likely_SC2	.0057284	.0120657	0.47	0.635	-.01793	.0293868
Deterr_SD_Severe	.0031168	.0173196	0.18	0.857	-.0308433	.0370769
MA_MoralBelief	.2461577	.0194933	12.63	0.000	.2079356	.2843799
MA_Authority_SC2	-.0082426	.0112806	-0.73	0.465	-.0303616	.0138763
NOO_SC3	.016953	.024684	0.69	0.492	-.0314471	.0653531
NNOO_SC3	.0202423	.0211123	0.96	0.338	-.0211543	.061639
OOL_SC12	.0154149	.0142116	1.08	0.278	-.0124511	.0432808
PJE_SC4	-.0097947	.0121723	-0.80	0.421	-.033662	.0140725
Trust_Science_SC4	.0379062	.0220487	1.72	0.086	-.0053266	.0811391
Trust_in_media	-.0123467	.0157546	-0.78	0.433	-.0432381	.0185447
Impulsivity_SC4	-.0854301	.019058	-4.48	0.000	-.1227987	-.0480615
NegEmo_SC6	.017582	.012638	1.39	0.164	-.0071986	.0423625
SN_SC7	.0298499	.0146116	2.04	0.041	.0011996	.0585001
CTC_SC7	.5161259	.0216554	23.83	0.000	.4736643	.5585876
OTC_SC7	-.0263942	.01021	-2.59	0.010	-.0464139	-.0063746
Deterr_SD_Severe	0	(omitted)				
wave_d1#c.Deterr_SD_Severe						
1	-.0120453	.0237789	-0.51	0.613	-.0586707	.03458
Deterr_SD_Severe	0	(omitted)				
wave_d2#c.Deterr_SD_Severe						
1	-.0079267	.0241631	-0.33	0.743	-.0553054	.039452
_cons	-.1267688	.2074418	-0.61	0.541	-.5335183	.2799807

```
.
. *14.a.3 Check hettest: Run this right after your regression to apply the Breusch-Pagan / Cook-Weisberg test for hete
. *if significant, then you need to run the regression with vce(ro) at the end
. estat hettest

Breusch-Pagan / Cook-Weisberg test for heteroskedasticity
         Ho: Constant variance
         Variables: fitted values of DV_Compliance_SC7

         chi2(1)      =   367.60
         Prob > chi2  =   0.0000

.
. *14.a.4. check vif, to check for for multicollinearity (VIFs >10 are problematic)
. vif
```

Variable	VIF	1/VIF
1.wave_d1	7.91	0.126488
1.wave_d2	8.07	0.123971
Age	1.27	0.790191
1.Gender_F~e	1.11	0.900344
1.Minority	1.15	0.868857
Education	1.28	0.779763
1.Employed	1.27	0.788777
1.Corona_c~e	1.22	0.819125
1.Insuranc~c	2.54	0.394224
1.Insuran~te	2.34	0.426502
SES_before	1.37	0.729215
SES_change	1.24	0.809613
2.Health_s~f	1.36	0.737987
2.Health_o~r	1.33	0.753573
1.Conserv~01	1.44	0.693663
1.Conserva~r	1.26	0.791001
1.GeoCensu~1	1.66	0.603586
1.GeoCensu~2	1.86	0.538797
1.GeoCensu~3	1.54	0.650188
1.Current_~s	1.14	0.878050
Measures_c~r	1.42	0.706025
MA_Perc_Th~3	2.40	0.417216
Costs_SC5	1.45	0.692030
Deterr_SD_~2	1.62	0.619183
Deterr_SD_~e	3.16	0.316274
MA_MoralBe~f	2.28	0.439118
MA_Authori~2	1.62	0.615794
NOO_SC3	1.77	0.566085
NNOO_SC3	1.58	0.631077
OOL_SC12	1.59	0.630911
PJE_SC4	1.38	0.726113
Trust_Scie~4	1.69	0.591956
Trust_in_m~a	1.52	0.656072
Impulsivi~C4	1.64	0.610368
NegEmo_SC6	1.40	0.713026
SN_SC7	1.49	0.673238
CTC_SC7	1.64	0.609712
OTC_SC7	1.14	0.874496
wave_d1# c. Deterr_SD_~e 1	8.57	0.116667
wave_d2# c. Deterr_SD_~e 1	8.78	0.113886
Mean VIF	2.26	

```
380 .
381 . *14.a.5. Effect size
382 . estat esize

    Effect sizes for linear models
```

Source	Eta-Squared	df	[95% Conf.	Interval]
Model	.5199592	40	.4907295	.5347511
wave_d1	.0000574	1	.	.0018547
wave_d2	.0004093	1	.	.0032098
Age	.0020593	1	.0000672	.0066705
Gender_Female	.0039828	1	.0007097	.0098552
Minority	5.02e-06	1	.	.0009359
Education	.0028037	1	.0002696	.0079551
Employed	.0000434	1	.	.0017352
Corona_care	.0001689	1	.	.0024328
Insurance_Public	.0009878	1	.	.0045976
Insurance_Private	.0019534	1	.0000435	.00648
SES_before	.0000987	1	.	.0021148
SES_change	2.10e-09	1	.	.
Health_self	.0002423	1	.	.0027007
Health_other	.0000861	1	.	.002045
Conservative_01	.0005239	1	.	.0035171
Conservative_other	.0011906	1	.	.0050218
GeoCensus_d1	.0011888	1	.	.0050182
GeoCensus_d2	.0025567	1	.0001953	.0075383
GeoCensus_d3	7.50e-08	1	.	.
Current_measures	.0040898	1	.0007554	.0100218
Measures_clear	.001024	1	.	.0046749
MA_Perc_Threat_SC3	.0182803	1	.0098436	.0290886
Costs_SC5	.0015809	1	.	.0057896
Deterr_SD_Likely_SC2	.0000783	1	.	.0019983
Deterr_SD_Severe	.0001009	1	.	.0021264
MA_MoralBelief	.0524985	1	.0378211	.0690338
MA_Authority_SC2	.0001855	1	.	.0024974
NOO_SC3	.0001639	1	.	.0024129
NNOO_SC3	.0003193	1	.	.0029476
OOL_SC12	.0004086	1	.	.0032079
PJE_SC4	.0002249	1	.	.002641
Trust_Science_SC4	.0010259	1	.	.004679
Trust_in_media	.0002134	1	.	.0026001
Impulsivity_SC4	.0069336	1	.0021982	.0142206
NegEmo_SC6	.000672	1	.	.0038847
SN_SC7	.001448	1	.	.0055342
CTC_SC7	.1648386	1	.1413882	.188753
OTC_SC7	.0023167	1	.0001299	.0071246
Deterr_SD_Severe	.0001009	1	.	.0021264
wave_d1#c.Deterr_SD_Severe	.0000892	1	.	.0020625
Deterr_SD_Severe	.0001009	1	.	.0021264
wave_d2#c.Deterr_SD_Severe	.0000374	1	.	.001674

```
    Note: Eta-Squared values for individual model terms are partial.

383 .
384 . *14.a.6 Regression with vce(ro)
385 . reg DV_Compliance_SC7 i.wave_d1 i.wave_d2 Age i.Gender_Female i.Minority Education i.Employed i.Corona_care i.Insura
    > rvative_01 i.Conservative_other i.GeoCensus_d1 i.GeoCensus_d2 i.GeoCensus_d3 i.Current_measures Measures_clear MA_Pe
    > _SC2 NOO_SC3 NNOO_SC3 OOL_SC12 PJE_SC4 Trust_Science_SC4 Trust_in_media Impulsivity_SC4 NegEmo_SC6 SN_SC7 CTC_SC7 OT
    >  1, vce(ro)
    note: Deterr_SD_Severe omitted because of collinearity
    note: Deterr_SD_Severe omitted because of collinearity

    Linear regression                               Number of obs     =      2,919
                                                    F(40, 2878)       =      67.25
                                                    Prob > F          =     0.0000
                                                    R-squared         =     0.5200
                                                    Root MSE          =      .9041
```

DV_Compliance_SC7	Coef.	Robust Std. Err.	t	P>|t|	[95% Conf.	Interval]
1.wave_d1	-.0404348	.0973736	-0.42	0.678	-.2313638	.1504942
1.wave_d2	-.1110186	.1071793	-1.04	0.300	-.3211746	.0991374
Age	.0035148	.0014418	2.44	0.015	.0006876	.0063419
1.Gender_Female	.1201508	.0354426	3.39	0.001	.0506555	.1896462
1.Minority	-.0045478	.0389222	-0.12	0.907	-.0808661	.0717705
Education	.0349449	.0120827	2.89	0.004	.0112532	.0586365
1.Employed	.013867	.0404381	0.34	0.732	-.0654236	.0931577
1.Corona_care	-.0455711	.0702193	-0.65	0.516	-.1832563	.0921141
1.Insurance_Public	.0907621	.060121	1.51	0.131	-.0271224	.2086466
1.Insurance_Private	.1334902	.0626097	2.13	0.033	.0107259	.2562546
SES_before	.005095	.010365	0.49	0.623	-.0152286	.0254185
SES_change	.0000276	.012416	0.00	0.998	-.0243176	.0243728
2.Health_self	.0343519	.0369233	0.93	0.352	-.0380468	.1067507
2.Health_other	-.0194687	.0377998	-0.52	0.607	-.0935861	.0546488
1.Conservative_01	.0495817	.0408046	1.22	0.224	-.0304274	.1295908
1.Conservative_other	.1072794	.0616799	1.74	0.082	-.0136618	.2282207
1.GeoCensus_d1	-.0982881	.0518929	-1.89	0.058	-.2000391	.0034629
1.GeoCensus_d2	-.1251484	.0441707	-2.83	0.005	-.2117577	-.038539
1.GeoCensus_d3	.0008312	.0513989	0.02	0.987	-.0999512	.1016136
Current_measures						
Yes	.1800586	.0638627	2.82	0.005	.0548373	.3052799
Measures_clear	-.0198023	.0122093	-1.62	0.105	-.0437421	.0041375
MA_Perc_Threat_SC3	.125903	.0197021	6.39	0.000	.0872714	.1645347
Costs_SC5	.0261897	.0126039	2.08	0.038	.0014761	.0509033
Deterr_SD_Likely_SC2	.0057284	.012286	0.47	0.641	-.0183618	.0298186
Deterr_SD_Severe	.0031168	.0168136	0.19	0.853	-.0298511	.0360848
MA_MoralBelief	.2461577	.0258952	9.51	0.000	.1953827	.2969327
MA_Authority_SC2	-.0082426	.0108882	-0.76	0.449	-.0295921	.0131068
NOO_SC3	.016953	.0257853	0.66	0.511	-.0336065	.0675124
NNOO_SC3	.0202423	.0215243	0.94	0.347	-.0219623	.0624469
OOL_SC12	.0154149	.0147338	1.05	0.296	-.013475	.0443047
PJE_SC4	-.0097947	.0120409	-0.81	0.416	-.0334043	.0138149
Trust_Science_SC4	.0379062	.0235064	1.61	0.107	-.0081849	.0839974
Trust_in_media	-.0123467	.015077	-0.82	0.413	-.0419095	.017216
Impulsivity_SC4	-.0854301	.0187631	-4.55	0.000	-.1222205	-.0486397
NegEmo_SC6	.017582	.0129025	1.36	0.173	-.007717	.0428809
SN_SC7	.0298499	.0146964	2.03	0.042	.0010332	.0586665
CTC_SC7	.5161259	.0319893	16.13	0.000	.4534017	.5788502
OTC_SC7	-.0263942	.0097474	-2.71	0.007	-.0455069	-.0072815
Deterr_SD_Severe	0	(omitted)				
wave_d1#c.Deterr_SD_Severe						
1	-.0120453	.0234087	-0.51	0.607	-.057945	.0338543
Deterr_SD_Severe	0	(omitted)				
wave_d2#c.Deterr_SD_Severe						
1	-.0079267	.0253567	-0.31	0.755	-.0576458	.0417924
_cons	-.1267688	.2175715	-0.58	0.560	-.5533805	.299843

```
.
.
. *****************
.
. *15. Model 15: Moral alignment x Survey Wave
```

```
391 .
392 . *15.a.2 Regression
393 . reg DV_Compliance_SC7 i.wave_d1 i.wave_d2 Age i.Gender_Female i.Minority Education i.Employed i.Corona_care i.Insura
    > rvative_01 i.Conservative_other i.GeoCensus_d1 i.GeoCensus_d2 i.GeoCensus_d3 i.Current_measures Measures_clear MA_Pe
    > _SC2 NOO_SC3 NNOO_SC3 OOL_SC12 PJE_SC4 Trust_Science_SC4 Trust_in_media Impulsivity_SC4 NegEmo_SC6 SN_SC7 CTC_SC7 OT
    note: MA_MoralBelief omitted because of collinearity
    note: MA_MoralBelief omitted because of collinearity
```

Source	SS	df	MS			
				Number of obs	=	2,919
				F(40, 2878)	=	77.96
Model	2548.49837	40	63.7124593	Prob > F	=	0.0000
Residual	2352.03213	2,878	.817245353	R-squared	=	0.5200
				Adj R-squared	=	0.5134
Total	4900.5305	2,918	1.67941415	Root MSE	=	.90402

DV_Compliance_SC7	Coef.	Std. Err.	t	P>|t|	[95% Conf.	Interval]
1.wave_d1	-.2474079	.2038861	-1.21	0.225	-.6471854	.1523696
1.wave_d2	-.2936598	.2072069	-1.42	0.157	-.6999489	.1126292
Age	.0034755	.0014413	2.41	0.016	.0006495	.0063015
1.Gender_Female	.1194305	.0353906	3.37	0.001	.0500371	.1888239
1.Minority	-.0050241	.0378083	-0.13	0.894	-.0791581	.06911
Education	.0347323	.0122851	2.83	0.005	.0106439	.0588207
1.Employed	.0130387	.0392389	0.33	0.740	-.0639005	.0899779
1.Corona_care	-.0459303	.0653439	-0.70	0.482	-.1740558	.0821953
1.Insurance_Public	.0920749	.0538104	1.71	0.087	-.013436	.1975857
1.Insurance_Private	.1350767	.056265	2.40	0.016	.0247529	.2454005
SES_before	.0050846	.0095612	0.53	0.595	-.0136629	.0238322
SES_change	-.0000336	.0112421	-0.00	0.998	-.0220771	.0220098
2.Health_self	.0329663	.0411709	0.80	0.423	-.0477612	.1136938
2.Health_other	-.0180485	.0391098	-0.46	0.644	-.0947345	.0586374
1.Conservative_01	.0491432	.0403668	1.22	0.224	-.0300075	.1282939
1.Conservative_other	.1067033	.0579242	1.84	0.066	-.0068738	.2202803
1.GeoCensus_d1	-.0975117	.0531181	-1.84	0.066	-.201665	.0066416
1.GeoCensus_d2	-.1259298	.046072	-2.73	0.006	-.2162673	-.0355923
1.GeoCensus_d3	-.0007113	.0565907	-0.01	0.990	-.1116737	.1102512
Current_measures						
Yes	.1792752	.052405	3.42	0.001	.0765201	.2820302
Measures_clear	-.0199913	.0115233	-1.73	0.083	-.0425862	.0026035
MA_Perc_Threat_SC3	.126415	.0172162	7.34	0.000	.0926576	.1601724
Costs_SC5	.0266691	.0122673	2.17	0.030	.0026154	.0507227
Deterr_SD_Likely_SC2	.0056789	.0120722	0.47	0.638	-.0179921	.0293499
Deterr_SD_Severe	-.0036498	.0106323	-0.34	0.731	-.0244975	.0171979
MA_MoralBelief	.2276538	.0287852	7.91	0.000	.1712122	.2840955
MA_Authority_SC2	-.0081079	.0112833	-0.72	0.472	-.030232	.0140163
NOO_SC3	.0178063	.0246864	0.72	0.471	-.0305985	.0662112
NNOO_SC3	.0202033	.0211003	0.96	0.338	-.02117	.0615766
OOL_SC12	.0157216	.0142156	1.11	0.269	-.0121521	.0435954
PJE_SC4	-.0096343	.0121645	-0.79	0.428	-.0334863	.0142177
Trust_Science_SC4	.0374877	.0220528	1.70	0.089	-.0057531	.0807286
Trust_in_media	-.0126382	.0157521	-0.80	0.422	-.0435248	.0182484
Impulsivity_SC4	-.0852335	.0190587	-4.47	0.000	-.1226036	-.0478635
NegEmo_SC6	.0171873	.0126523	1.36	0.174	-.0076212	.0419958
SN_SC7	.0295092	.0145944	2.02	0.043	.0008928	.0581257
CTC_SC7	.5164652	.0216713	23.83	0.000	.4739724	.5589581
OTC_SC7	-.0264046	.0102086	-2.59	0.010	-.0464216	-.0063876
MA_MoralBelief	0	(omitted)				
wave_d1#c.MA_MoralBelief						
1	.0261125	.0324295	0.81	0.421	-.0374749	.0896999
MA_MoralBelief	0	(omitted)				
wave_d2#c.MA_MoralBelief						
1	.0245781	.0328211	0.75	0.454	-.0397772	.0889334
_cons	.0111258	.2347544	0.05	0.962	-.449178	.4714295

```
394 .
395 . *15.a.3 Check hettest: Run this right after your regression to apply the Breusch-Pagan / Cook-Weisberg test for hete
396 . *if significant, then you need to run the regression with vce(ro) at the end
397 . estat hettest

    Breusch-Pagan / Cook-Weisberg test for heteroskedasticity
             Ho: Constant variance
             Variables: fitted values of DV_Compliance_SC7

             chi2(1)      =   367.03
             Prob > chi2  =   0.0000

398 .
399 . *15.a.4. check vif, to check for for multicollinearity (VIFs >10 are problematic)
400 . vif
```

Variable	VIF	1/VIF
1.wave_d1	33.21	0.030109
1.wave_d2	33.12	0.030194
Age	1.26	0.791174
1.Gender_F~e	1.11	0.901566
1.Minority	1.15	0.869221
Education	1.28	0.779584
1.Employed	1.27	0.788194
1.Corona_c~e	1.22	0.819531
1.Insuranc~c	2.54	0.394053
1.Insuran~te	2.35	0.426132
SES_before	1.37	0.728974
SES_change	1.24	0.809612
2.Health_s~f	1.36	0.736587
2.Health_o~r	1.33	0.753304
1.Conserv~01	1.44	0.693611
1.Conserva~r	1.26	0.790742
1.GeoCensu~1	1.66	0.603189
1.GeoCensu~2	1.86	0.538807
1.GeoCensu~3	1.54	0.649392
1.Current_~s	1.14	0.876901
Measures_c~r	1.42	0.706607
MA_Perc_Th~3	2.40	0.416288
Costs_SC5	1.44	0.692046
Deterr_SD_~2	1.62	0.618412
Deterr_SD_~e	1.19	0.839085
MA_MoralBe~f	4.97	0.201342
MA_Authori~2	1.62	0.615394
NOO_SC3	1.77	0.565872
NNOO_SC3	1.58	0.631676
OOL_SC12	1.59	0.630444
PJE_SC4	1.38	0.726912
Trust_Scie~4	1.69	0.591631
Trust_in_m~a	1.52	0.656159
Impulsivi~C4	1.64	0.610213
NegEmo_SC6	1.41	0.711289
SN_SC7	1.48	0.674705
CTC_SC7	1.64	0.608706
OTC_SC7	1.14	0.874574
wave_d1# c. MA_MoralBe~f 1	33.57	0.029793
wave_d2# c. MA_MoralBe~f 1	33.68	0.029692
Mean VIF	4.79	

```
401 .
402 . *15.a.5. Effect size
403 . estat esize
```

Effect sizes for linear models

Source	Eta-Squared	df	[95% Conf.	Interval]
Model	.5200454	40	.4908196	.5348357
wave_d1	.0005114	1	.	.0034847
wave_d2	.0006974	1	.	.0039451
Age	.0020165	1	.0000575	.0065938
Gender_Female	.0039414	1	.0006922	.0097905
Minority	6.14e-06	1	.	.0010061
Education	.0027696	1	.0002589	.0078981
Employed	.0000384	1	.	.0016844
Corona_care	.0001716	1	.	.0024439
Insurance_Public	.0010163	1	.	.0046585
Insurance_Private	.0019986	1	.0000535	.0065616
SES_before	.0000983	1	.	.0021126
SES_change	3.11e-09	1	.	.
Health_self	.0002227	1	.	.0026333
Health_other	.000074	1	.	.0019711
Conservative_01	.0005147	1	.	.0034933
Conservative_other	.0011777	1	.	.0049954
GeoCensus_d1	.0011696	1	.	.0049788
GeoCensus_d2	.0025892	1	.0002047	.0075935
GeoCensus_d3	5.49e-08	1	.	.
Current_measures	.0040499	1	.0007383	.0099597
Measures_clear	.0010447	1	.	.0047187
MA_Perc_Threat_SC3	.0183895	1	.0099246	.0292243
Costs_SC5	.0016395	1	.	.0059006
Deterr_SD_Likely_SC2	.0000769	1	.	.0019894
Deterr_SD_Severe	.0000409	1	.	.001711
MA_MoralBelief	.0485136	1	.034391	.0645517
MA_Authority_SC2	.0001794	1	.	.0024741
NOO_SC3	.0001807	1	.	.0024793
NNOO_SC3	.0003184	1	.	.002945
OOL_SC12	.0004248	1	.	.0032529
PJE_SC4	.0002179	1	.	.0026163
Trust_Science_SC4	.0010031	1	.	.0046303
Trust_in_media	.0002236	1	.	.0026364
Impulsivity_SC4	.0069014	1	.00218	.014175
NegEmo_SC6	.0006408	1	.	.0038093
SN_SC7	.0014185	1	.	.0054767
CTC_SC7	.164817	1	.1413675	.1887307
OTC_SC7	.0023191	1	.0001305	.0071289
MA_MoralBelief	.0485136	1	.034391	.0645517
wave_d1#c.MA_MoralBelief	.0002252	1	.	.0026421
MA_MoralBelief	.0485136	1	.034391	.0645517
wave_d2#c.MA_MoralBelief	.0001948	1	.	.0025325

Note: Eta-Squared values for individual model terms are partial.

```
404 .
405 . *15.a.6 Regression with vce(ro)
406 . reg DV_Compliance_SC7 i.wave_d1 i.wave_d2 Age i.Gender_Female i.Minority Education i.Employed i.Corona_care i.Insura
    > rvative_01 i.Conservative_other i.GeoCensus_d1 i.GeoCensus_d2 i.GeoCensus_d3 i.Current_measures Measures_clear MA_Pe
    > _SC2 NOO_SC3 NNOO_SC3 OOL_SC12 PJE_SC4 Trust_Science_SC4 Trust_in_media Impulsivity_SC4 NegEmo_SC6 SN_SC7 CTC_SC7 OT
    > vce(ro)
note: MA_MoralBelief omitted because of collinearity
note: MA_MoralBelief omitted because of collinearity

Linear regression                               Number of obs     =      2,919
                                                F(40, 2878)       =      68.61
                                                Prob > F          =     0.0000
                                                R-squared         =     0.5200
                                                Root MSE          =     .90402
```

DV_Compliance_SC7	Coef.	Robust Std. Err.	t	P>|t|	[95% Conf.	Interval]
1.wave_d1	-.2474079	.2733976	-0.90	0.366	-.7834828	.2886669
1.wave_d2	-.2936598	.2514918	-1.17	0.243	-.7867821	.1994624
Age	.0034755	.0014363	2.42	0.016	.0006592	.0062918
1.Gender_Female	.1194305	.0354419	3.37	0.001	.0499364	.1889245
1.Minority	-.0050241	.038908	-0.13	0.897	-.0813145	.0712664
Education	.0347323	.0121131	2.87	0.004	.010981	.0584836
1.Employed	.0130387	.0404004	0.32	0.747	-.0661778	.0922553
1.Corona_care	-.0459303	.0702236	-0.65	0.513	-.183624	.0917634
1.Insurance_Public	.0920749	.0601435	1.53	0.126	-.0258539	.2100036
1.Insurance_Private	.1350767	.0625967	2.16	0.031	.0123379	.2578155
SES_before	.0050846	.0103707	0.49	0.624	-.0152501	.0254194
SES_change	-.0000336	.0124131	-0.00	0.998	-.0243731	.0243059
2.Health_self	.0329663	.0369407	0.89	0.372	-.0394666	.1053992
2.Health_other	-.0180485	.0377737	-0.48	0.633	-.0921148	.0560177
1.Conservative_01	.0491432	.040885	1.20	0.229	-.0310237	.1293101
1.Conservative_other	.1067033	.0616994	1.73	0.084	-.0142763	.2276828
1.GeoCensus_d1	-.0975117	.0518186	-1.88	0.060	-.199117	.0040936
1.GeoCensus_d2	-.1259298	.0441783	-2.85	0.004	-.2125542	-.0393055
1.GeoCensus_d3	-.0007113	.0515208	-0.01	0.989	-.1017326	.1003101
Current_measures						
Yes	.1792752	.0638681	2.81	0.005	.0540434	.3045069
Measures_clear	-.0199913	.0121908	-1.64	0.101	-.043895	.0039123
MA_Perc_Threat_SC3	.126415	.0197482	6.40	0.000	.0876929	.1651371
Costs_SC5	.0266691	.0126518	2.11	0.035	.0018617	.0514765
Deterr_SD_Likely_SC2	.0056789	.0122537	0.46	0.643	-.0183481	.0297059
Deterr_SD_Severe	-.0036498	.0111408	-0.33	0.743	-.0254945	.0181949
MA_MoralBelief	.2276538	.0334713	6.80	0.000	.1620237	.2932839
MA_Authority_SC2	-.0081079	.0108836	-0.74	0.456	-.0294483	.0132325
NOO_SC3	.0178063	.0258067	0.69	0.490	-.0327952	.0684078
NNOO_SC3	.0202033	.0214914	0.94	0.347	-.0219368	.0623434
OOL_SC12	.0157216	.014705	1.07	0.285	-.0131117	.0445549
PJE_SC4	-.0096343	.0119946	-0.80	0.422	-.0331533	.0138846
Trust_Science_SC4	.0374877	.0234918	1.60	0.111	-.0085747	.0835501
Trust_in_media	-.0126382	.0150323	-0.84	0.401	-.0421133	.016837
Impulsivity_SC4	-.0852335	.0187456	-4.55	0.000	-.1219898	-.0484773
NegEmo_SC6	.0171873	.0129609	1.33	0.185	-.0082263	.0426008
SN_SC7	.0295092	.0146637	2.01	0.044	.0007568	.0582617
CTC_SC7	.5164652	.0319109	16.18	0.000	.4538948	.5790357
OTC_SC7	-.0264046	.009771	-2.70	0.007	-.0455635	-.0072457
MA_MoralBelief	0	(omitted)				
wave_d1#c.MA_MoralBelief						
1	.0261125	.0421292	0.62	0.535	-.0564939	.108719
MA_MoralBelief	0	(omitted)				
wave_d2#c.MA_MoralBelief						
1	.0245781	.0386676	0.64	0.525	-.051241	.1003972
_cons	.0111258	.2635978	0.04	0.966	-.5057337	.5279852

```
. 
. 
. *****************
. 
. *16. Model 16: Authority evaluation x Survey Wave
```

```
412 .
413 . *16.a.2 Regression
414 . reg DV_Compliance_SC7 i.wave_d1 i.wave_d2 Age i.Gender_Female i.Minority Education i.Employed i.Corona_care i.Insura
    > rvative_01 i.Conservative_other i.GeoCensus_d1 i.GeoCensus_d2 i.GeoCensus_d3 i.Current_measures Measures_clear MA_Pe
    > _SC2 NOO_SC3 NNOO_SC3 OOL_SC12 PJE_SC4 Trust_Science_SC4 Trust_in_media Impulsivity_SC4 NegEmo_SC6 SN_SC7 CTC_SC7 OT
    >  1
    note: MA_Authority_SC2 omitted because of collinearity
    note: MA_Authority_SC2 omitted because of collinearity
```

Source	SS	df	MS			
				Number of obs	=	2,919
				F(40, 2878)	=	78.00
Model	2549.1498	40	63.728745	Prob > F	=	0.0000
Residual	2351.3807	2,878	.817019007	R-squared	=	0.5202
				Adj R-squared	=	0.5135
Total	4900.5305	2,918	1.67941415	Root MSE	=	.90389

DV_Compliance_SC7	Coef.	Std. Err.	t	P>|t|	[95% Conf.	Interval]
1.wave_d1	-.1926047	.1038665	-1.85	0.064	-.3962649	.0110555
1.wave_d2	-.2367451	.0982703	-2.41	0.016	-.4294323	-.0440578
Age	.0034651	.0014413	2.40	0.016	.0006389	.0062912
1.Gender_Female	.1198485	.0353839	3.39	0.001	.0504682	.1892289
1.Minority	-.0057352	.0378138	-0.15	0.879	-.0798802	.0684097
Education	.034249	.0122942	2.79	0.005	.0101427	.0583554
1.Employed	.0149822	.0392368	0.38	0.703	-.061953	.0919173
1.Corona_care	-.0481469	.0653522	-0.74	0.461	-.1762887	.0799949
1.Insurance_Public	.0918636	.0538127	1.71	0.088	-.0136517	.197379
1.Insurance_Private	.1335882	.0562301	2.38	0.018	.0233329	.2438435
SES_before	.004527	.0095675	0.47	0.636	-.0142329	.023287
SES_change	-.0003457	.0112457	-0.03	0.975	-.0223962	.0217047
2.Health_self	.0338442	.0411249	0.82	0.411	-.046793	.1144814
2.Health_other	-.0182828	.0390845	-0.47	0.640	-.0949193	.0583537
1.Conservative_01	.0505417	.0403674	1.25	0.211	-.0286104	.1296937
1.Conservative_other	.1070555	.0579003	1.85	0.065	-.0064748	.2205859
1.GeoCensus_d1	-.0973646	.0531179	-1.83	0.067	-.2015175	.0067883
1.GeoCensus_d2	-.1246376	.0460655	-2.71	0.007	-.2149623	-.0343129
1.GeoCensus_d3	.0005391	.0565703	0.01	0.992	-.1103832	.1114615
Current_measures						
Yes	.1798524	.0523334	3.44	0.001	.0772377	.2824671
Measures_clear	-.0199542	.0115336	-1.73	0.084	-.0425692	.0026607
MA_Perc_Threat_SC3	.1258028	.017192	7.32	0.000	.0920929	.1595127
Costs_SC5	.02643	.0122624	2.16	0.031	.0023862	.0504739
Deterr_SD_Likely_SC2	.0055384	.0120758	0.46	0.647	-.0181397	.0292164
Deterr_SD_Severe	-.0039524	.0106394	-0.37	0.710	-.0248141	.0169093
MA_MoralBelief	.2469968	.0194803	12.68	0.000	.2088	.2851936
MA_Authority_SC2	-.0238296	.0167419	-1.42	0.155	-.0566569	.0089977
NOO_SC3	.0173373	.0246751	0.70	0.482	-.0310454	.06572
NNOO_SC3	.0200903	.0211092	0.95	0.341	-.0213004	.061481
OOL_SC12	.0149438	.0142105	1.05	0.293	-.0129199	.0428076
PJE_SC4	-.0099588	.0121619	-0.82	0.413	-.0338057	.0138881
Trust_Science_SC4	.0381769	.0220461	1.73	0.083	-.0050508	.0814046
Trust_in_media	-.0129323	.0157552	-0.82	0.412	-.043825	.0179604
Impulsivity_SC4	-.0857263	.0190537	-4.50	0.000	-.1230866	-.048366
NegEmo_SC6	.01722	.0126418	1.36	0.173	-.0075679	.0420079
SN_SC7	.0291024	.0145968	1.99	0.046	.0004811	.0577237
CTC_SC7	.5163182	.021651	23.85	0.000	.4738652	.5587712
OTC_SC7	-.026256	.0102063	-2.57	0.010	-.0462685	-.0062435
MA_Authority_SC2	0	(omitted)				
wave_d1#c.MA_Authority_SC2						
1	.024699	.0220773	1.12	0.263	-.0185899	.067988
MA_Authority_SC2	0	(omitted)				
wave_d2#c.MA_Authority_SC2						
1	.0230431	.0219042	1.05	0.293	-.0199064	.0659926
_cons	-.023921	.2078167	-0.12	0.908	-.4314056	.3835635

```
. 
. *16.a.3 Check hettest: Run this right after your regression to apply the Breusch-Pagan / Cook-Weisberg test for hete
. *if significant, then you need to run the regression with vce(ro) at the end
. estat hettest

Breusch-Pagan / Cook-Weisberg test for heteroskedasticity 
         Ho: Constant variance
         Variables: fitted values of DV_Compliance_SC7

         chi2(1)      =   366.09
         Prob > chi2  =   0.0000

. 
. *16.a.4. check vif, to check for for multicollinearity (VIFs >10 are problematic)
. vif
```

Variable	VIF	1/VIF
1.wave_d1	8.62	0.115986
1.wave_d2	7.45	0.134204
Age	1.26	0.790869
1.Gender_F~e	1.11	0.901655
1.Minority	1.15	0.868725
Education	1.29	0.778209
1.Employed	1.27	0.788059
1.Corona_c~e	1.22	0.819096
1.Insuranc~c	2.54	0.393910
1.Insuran~te	2.34	0.426543
SES_before	1.37	0.727809
SES_change	1.24	0.808873
2.Health_s~f	1.35	0.738034
2.Health_o~r	1.33	0.754068
1.Conserv~01	1.44	0.693396
1.Conserva~r	1.26	0.791174
1.GeoCensu~1	1.66	0.603027
1.GeoCensu~2	1.86	0.538810
1.GeoCensu~3	1.54	0.649682
1.Current_~s	1.14	0.879057
Measures_c~r	1.42	0.705157
MA_Perc_Th~3	2.40	0.417345
Costs_SC5	1.44	0.692417
Deterr_SD_~2	1.62	0.617870
Deterr_SD_~e	1.19	0.837727
MA_MoralBe~f	2.28	0.439500
MA_Authori~2	3.58	0.279445
NOO_SC3	1.77	0.566234
NNOO_SC3	1.58	0.630970
OOL_SC12	1.59	0.630723
PJE_SC4	1.38	0.727021
Trust_Scie~4	1.69	0.591826
Trust_in_m~a	1.53	0.655720
Impulsivi~C4	1.64	0.610363
NegEmo_SC6	1.40	0.712278
SN_SC7	1.48	0.674290
CTC_SC7	1.64	0.609680
OTC_SC7	1.14	0.874724
wave_d1# c. MA_Authori~2 1	9.38	0.106651
wave_d2# c. MA_Authori~2 1	7.43	0.134569
Mean VIF	2.25	

```
422 .
423 . *16.a.5. Effect size
424 . estat esize
```

Effect sizes for linear models

Source	Eta-Squared	df	[95% Conf.	Interval]
Model	.5201783	40	.4909585	.5349662
wave_d1	.0011934	1	.	.0050275
wave_d2	.0020126	1	.0000566	.0065868
Age	.0020042	1	.0000548	.0065717
Gender_Female	.0039704	1	.0007045	.0098359
Minority	7.99e-06	1	.	.0010996
Education	.0026893	1	.0002343	.007763
Employed	.0000507	1	.	.0018003
Corona_care	.0001886	1	.	.0025091
Insurance_Public	.0010115	1	.	.0046484
Insurance_Private	.0019573	1	.0000444	.0064871
SES_before	.0000778	1	.	.001995
SES_change	3.28e-07	1	.	.
Health_self	.0002353	1	.	.0026768
Health_other	.000076	1	.	.001984
Conservative_01	.0005444	1	.	.0035697
Conservative_other	.0011865	1	.	.0050133
GeoCensus_d1	.0011661	1	.	.0049716
GeoCensus_d2	.0025372	1	.0001897	.0075049
GeoCensus_d3	3.16e-08	1	.	.
Current_measures	.004087	1	.0007542	.0100174
Measures_clear	.001039	1	.	.0047066
MA_Perc_Threat_SC3	.0182654	1	.0098326	.0290701
Costs_SC5	.0016116	1	.	.0058479
Deterr_SD_Likely_SC2	.0000731	1	.	.0019652
Deterr_SD_Severe	.0000479	1	.	.0017769
MA_MoralBelief	.0529046	1	.0381723	.0694889
MA_Authority_SC2	3.49e-09	1	.	.
NOO_SC3	.0001715	1	.	.0024433
NNOO_SC3	.0003146	1	.	.0029333
OOL_SC12	.0003841	1	.	.0031386
PJE_SC4	.0002329	1	.	.0026688
Trust_Science_SC4	.0010409	1	.	.0047107
Trust_in_media	.0002341	1	.	.0026727
Impulsivity_SC4	.0069845	1	.0022271	.0142928
NegEmo_SC6	.0006443	1	.	.0038179
SN_SC7	.0013793	1	.	.0053998
CTC_SC7	.1649971	1	.1415399	.1889165
OTC_SC7	.0022942	1	.0001241	.0070854
MA_Authority_SC2	3.49e-09	1	.	.
wave_d1#c.MA_Authority_SC2	.0004347	1	.	.0032801
MA_Authority_SC2	3.49e-09	1	.	.
wave_d2#c.MA_Authority_SC2	.0003844	1	.	.0031394

Note: Eta-Squared values for individual model terms are partial.

```
425 .
426 . *16.a.6 Regression with vce(ro)
427 . reg DV_Compliance_SC7 i.wave_d1 i.wave_d2 Age i.Gender_Female i.Minority Education i.Employed i.Corona_care i.Insura
    > rvative_01 i.Conservative_other i.GeoCensus_d1 i.GeoCensus_d2 i.GeoCensus_d3 i.Current_measures Measures_clear MA_Pe
    > _SC2 NOO_SC3 NNOO_SC3 OOL_SC12 PJE_SC4 Trust_Science_SC4 Trust_in_media Impulsivity_SC4 NegEmo_SC6 SN_SC7 CTC_SC7 OT
    >  1, vce(ro)
    note: MA_Authority_SC2 omitted because of collinearity
    note: MA_Authority_SC2 omitted because of collinearity

    Linear regression                               Number of obs     =      2,919
                                                    F(40, 2878)       =      67.16
                                                    Prob > F          =     0.0000
                                                    R-squared         =     0.5202
                                                    Root MSE          =     .90389
```

DV_Compliance_SC7	Coef.	Robust Std. Err.	t	P>|t|	[95% Conf.	Interval]
1.wave_d1	-.1926047	.0940103	-2.05	0.041	-.376939	-.0082704
1.wave_d2	-.2367451	.0893634	-2.65	0.008	-.4119678	-.0615223
Age	.0034651	.0014367	2.41	0.016	.0006481	.0062821
1.Gender_Female	.1198485	.03544	3.38	0.001	.0503582	.1893389
1.Minority	-.0057352	.0389142	-0.15	0.883	-.0820377	.0705672
Education	.034249	.0120815	2.83	0.005	.0105597	.0579383
1.Employed	.0149822	.0404197	0.37	0.711	-.0642723	.0942366
1.Corona_care	-.0481469	.070178	-0.69	0.493	-.1857512	.0894574
1.Insurance_Public	.0918636	.0600756	1.53	0.126	-.025932	.2096593
1.Insurance_Private	.1335882	.0625658	2.14	0.033	.01091	.2562664
SES_before	.004527	.0103329	0.44	0.661	-.0157335	.0247876
SES_change	-.0003457	.0124014	-0.03	0.978	-.0246623	.0239708
2.Health_self	.0338442	.0369438	0.92	0.360	-.0385948	.1062831
2.Health_other	-.0182828	.037808	-0.48	0.629	-.0924163	.0558506
1.Conservative_01	.0505417	.0408	1.24	0.216	-.0294585	.1305418
1.Conservative_other	.1070555	.061623	1.74	0.082	-.0137741	.2278852
1.GeoCensus_d1	-.0973646	.0519254	-1.88	0.061	-.1991793	.0044501
1.GeoCensus_d2	-.1246376	.0441832	-2.82	0.005	-.2112715	-.0380037
1.GeoCensus_d3	.0005391	.0515269	0.01	0.992	-.1004942	.1015724
Current_measures						
Yes	.1798524	.063852	2.82	0.005	.0546521	.3050526
Measures_clear	-.0199542	.0121316	-1.64	0.100	-.0437418	.0038333
MA_Perc_Threat_SC3	.1258028	.019733	6.38	0.000	.0871106	.164495
Costs_SC5	.02643	.0126289	2.09	0.036	.0016674	.0511927
Deterr_SD_Likely_SC2	.0055384	.0123047	0.45	0.653	-.0185886	.0296654
Deterr_SD_Severe	-.0039524	.0111611	-0.35	0.723	-.0258369	.0179321
MA_MoralBelief	.2469968	.0259754	9.51	0.000	.1960645	.2979291
MA_Authority_SC2	-.0238296	.0145363	-1.64	0.101	-.0523321	.004673
NOO_SC3	.0173373	.0257723	0.67	0.501	-.0331968	.0678713
NNOO_SC3	.0200903	.0215165	0.93	0.351	-.022099	.0622796
OOL_SC12	.0149438	.0147531	1.01	0.311	-.0139839	.0438716
PJE_SC4	-.0099588	.0119972	-0.83	0.407	-.0334827	.0135651
Trust_Science_SC4	.0381769	.0235164	1.62	0.105	-.0079337	.0842875
Trust_in_media	-.0129323	.0150114	-0.86	0.389	-.0423665	.0165019
Impulsivity_SC4	-.0857263	.018762	-4.57	0.000	-.1225146	-.0489381
NegEmo_SC6	.01722	.0129186	1.33	0.183	-.0081107	.0425507
SN_SC7	.0291024	.0146236	1.99	0.047	.0004287	.0577762
CTC_SC7	.5163182	.0319354	16.17	0.000	.4536997	.5789367
OTC_SC7	-.026256	.0097682	-2.69	0.007	-.0454093	-.0071027
MA_Authority_SC2	0	(omitted)				
wave_d1#c.MA_Authority_SC2						
1	.024699	.0204717	1.21	0.228	-.0154416	.0648396
MA_Authority_SC2	0	(omitted)				
wave_d2#c.MA_Authority_SC2						
1	.0230431	.0209358	1.10	0.271	-.0180075	.0640938
_cons	-.023921	.2200873	-0.11	0.913	-.4554658	.4076237

```
.
.
. *****************
.
. *17. Model 17: Normative obligation x Survey Wave
```

```
433 .
434 . *17.a.2 Regression
435 . reg DV_Compliance_SC7 i.wave_d1 i.wave_d2 Age i.Gender_Female i.Minority Education i.Employed i.Corona_care i.Insura
    > rvative_01 i.Conservative_other i.GeoCensus_d1 i.GeoCensus_d2 i.GeoCensus_d3 i.Current_measures Measures_clear MA_Pe
    > _SC2 NOO_SC3 NNOO_SC3 OOL_SC12 PJE_SC4 Trust_Science_SC4 Trust_in_media Impulsivity_SC4 NegEmo_SC6 SN_SC7 CTC_SC7 OT
note: NOO_SC3 omitted because of collinearity
note: NOO_SC3 omitted because of collinearity
```

Source	SS	df	MS			
				Number of obs	=	2,919
				F(40, 2878)	=	78.37
Model	2554.91485	40	63.8728714	Prob > F	=	0.0000
Residual	2345.61565	2,878	.81501586	R-squared	=	0.5214
				Adj R-squared	=	0.5147
Total	4900.5305	2,918	1.67941415	Root MSE	=	.90278

DV_Compliance_SC7	Coef.	Std. Err.	t	P>|t|	[95% Conf.	Interval]
1.wave_d1	-.4272575	.1849262	-2.31	0.021	-.7898587	-.0646563
1.wave_d2	-.6747006	.1875535	-3.60	0.000	-1.042453	-.3069478
Age	.0034074	.0014394	2.37	0.018	.0005851	.0062298
1.Gender_Female	.1181739	.0353571	3.34	0.001	.0488461	.1875016
1.Minority	-.00795	.0377782	-0.21	0.833	-.082025	.066125
Education	.0337292	.0122728	2.75	0.006	.0096648	.0577937
1.Employed	.0125576	.0391736	0.32	0.749	-.0642536	.0893688
1.Corona_care	-.0516802	.0652769	-0.79	0.429	-.1796744	.0763141
1.Insurance_Public	.0974178	.0537705	1.81	0.070	-.0080148	.2028504
1.Insurance_Private	.138714	.0561941	2.47	0.014	.0285292	.2488988
SES_before	.0047463	.0095464	0.50	0.619	-.0139722	.0234649
SES_change	-.0000636	.0112267	-0.01	0.995	-.0220767	.0219495
2.Health_self	.0293585	.0411059	0.71	0.475	-.0512415	.1099584
2.Health_other	-.0164704	.0390405	-0.42	0.673	-.0930206	.0600798
1.Conservative_01	.0473948	.0403199	1.18	0.240	-.0316639	.1264535
1.Conservative_other	.1075377	.0578337	1.86	0.063	-.0058619	.2209373
1.GeoCensus_d1	-.0994982	.0530289	-1.88	0.061	-.2034766	.0044801
1.GeoCensus_d2	-.1268284	.0460116	-2.76	0.006	-.2170474	-.0366093
1.GeoCensus_d3	-.0048226	.0565189	-0.09	0.932	-.1156442	.1059991
Current_measures						
Yes	.1783088	.0523219	3.41	0.001	.0757166	.2809009
Measures_clear	-.0195489	.0115087	-1.70	0.090	-.0421151	.0030173
MA_Perc_Threat_SC3	.1280031	.0171849	7.45	0.000	.0943072	.1616991
Costs_SC5	.026903	.0122502	2.20	0.028	.0028829	.0509232
Deterr_SD_Likely_SC2	.0048946	.0120501	0.41	0.685	-.018733	.0285223
Deterr_SD_Severe	-.0036063	.0106181	-0.34	0.734	-.0244261	.0172136
MA_MoralBelief	.2459066	.01945	12.64	0.000	.2077692	.284044
MA_Authority_SC2	-.0073349	.0112649	-0.65	0.515	-.0294231	.0147532
NOO_SC3	-.0598282	.0375508	-1.59	0.111	-.1334574	.0138009
NNOO_SC3	.0216432	.021078	1.03	0.305	-.0196864	.0629727
OOL_SC12	.0165182	.0142006	1.16	0.245	-.0113261	.0443625
PJE_SC4	-.0100221	.0121514	-0.82	0.410	-.0338484	.0138042
Trust_Science_SC4	.035609	.0220342	1.62	0.106	-.0075954	.0788135
Trust_in_media	-.0119528	.0157276	-0.76	0.447	-.0427913	.0188858
Impulsivity_SC4	-.0839002	.019039	-4.41	0.000	-.1212317	-.0465687
NegEmo_SC6	.0164111	.012626	1.30	0.194	-.0083458	.041168
SN_SC7	.0296339	.0145744	2.03	0.042	.0010567	.0582112
CTC_SC7	.5161626	.0216334	23.86	0.000	.4737442	.558581
OTC_SC7	-.0268345	.0101982	-2.63	0.009	-.0468309	-.006838
NOO_SC3	0	(omitted)				
wave_d1#c.NOO_SC3						
1	.0863083	.046122	1.87	0.061	-.0041273	.1767439
NOO_SC3	0	(omitted)				
wave_d2#c.NOO_SC3						
1	.1354807	.0464662	2.92	0.004	.0443704	.2265911
_cons	.2058956	.2277304	0.90	0.366	-.2406355	.6524267

```
436 .
437 . *17.a.3 Check hettest: Run this right after your regression to apply the Breusch-Pagan / Cook-Weisberg test for hete
438 . *if significant, then you need to run the regression with vce(ro) at the end
439 . estat hettest

    Breusch-Pagan / Cook-Weisberg test for heteroskedasticity
             Ho: Constant variance
             Variables: fitted values of DV_Compliance_SC7

             chi2(1)      =   363.05
             Prob > chi2  =   0.0000

440 .
441 . *17.a.4. check vif, to check for for multicollinearity (VIFs >10 are problematic)
442 . vif
```

Variable	VIF	1/VIF
1.wave_d1	27.40	0.036500
1.wave_d2	27.21	0.036753
Age	1.26	0.791054
1.Gender_F~e	1.11	0.900810
1.Minority	1.15	0.868233
Education	1.28	0.779006
1.Employed	1.27	0.788665
1.Corona_c~e	1.22	0.818973
1.Insuranc~c	2.54	0.393561
1.Insuran~te	2.35	0.426043
SES_before	1.37	0.729242
SES_change	1.24	0.809631
2.Health_s~f	1.36	0.736905
2.Health_o~r	1.33	0.753916
1.Conserv~01	1.44	0.693330
1.Conserva~r	1.26	0.791055
1.GeoCensu~1	1.66	0.603569
1.GeoCensu~2	1.86	0.538749
1.GeoCensu~3	1.54	0.649267
1.Current_~s	1.14	0.877288
Measures_c~r	1.42	0.706469
MA_Perc_Th~3	2.40	0.416667
Costs_SC5	1.44	0.692087
Deterr_SD_~2	1.62	0.618991
Deterr_SD_~e	1.19	0.839038
MA_MoralBe~f	2.27	0.439789
MA_Authori~2	1.62	0.615717
NOO_SC3	4.10	0.243899
NNOO_SC3	1.58	0.631287
OOL_SC12	1.59	0.630054
PJE_SC4	1.38	0.726491
Trust_Scie~4	1.69	0.591011
Trust_in_m~a	1.52	0.656411
Impulsivi~C4	1.64	0.609806
NegEmo_SC6	1.40	0.712312
SN_SC7	1.48	0.674713
CTC_SC7	1.64	0.609178
OTC_SC7	1.14	0.873976
wave_d1# c.NOO_SC3 1	27.26	0.036683
wave_d2# c.NOO_SC3 1	27.51	0.036344
Mean VIF	4.17	

```
443 .
444 . *17.a.5. Effect size
445 . estat esize
```

Effect sizes for linear models

Source	Eta-Squared	df	[95% Conf.	Interval]
Model	.5213548	40	.4921878	.5361206
wave_d1	.0018513	1	.0000218	.0062942
wave_d2	.0044764	1	.0009272	.010617
Age	.0019434	1	.0000414	.0064619
Gender_Female	.0038665	1	.0006609	.0096732
Minority	.0000154	1	.	.0013352
Education	.0026175	1	.0002129	.0076417
Employed	.0000357	1	.	.0016554
Corona_care	.0002177	1	.	.0026157
Insurance_Public	.0011392	1	.	.0049162
Insurance_Private	.0021128	1	.0000796	.0067659
SES_before	.0000859	1	.	.0020437
SES_change	1.11e-08	1	.	.
Health_self	.0001772	1	.	.0024657
Health_other	.0000618	1	.	.001888
Conservative_01	.0004799	1	.	.0034019
Conservative_other	.0011999	1	.	.0050408
GeoCensus_d1	.0012218	1	.	.0050852
GeoCensus_d2	.0026331	1	.0002175	.007668
GeoCensus_d3	2.53e-06	1	.	.000696
Current_measures	.0040192	1	.0007252	.009912
Measures_clear	.0010015	1	.	.004627
MA_Perc_Threat_SC3	.0189131	1	.0103142	.0298738
Costs_SC5	.001673	1	.	.0059636
Deterr_SD_Likely_SC2	.0000573	1	.	.0018542
Deterr_SD_Severe	.0000401	1	.	.0017022
MA_MoralBelief	.0526179	1	.0379243	.0691676
MA_Authority_SC2	.0001473	1	.	.0023443
NOO_SC3	.0011995	1	.	.00504
NNOO_SC3	.0003662	1	.	.0030871
OOL_SC12	.0004699	1	.	.0033754
PJE_SC4	.0002363	1	.	.0026804
Trust_Science_SC4	.0009067	1	.	.0044214
Trust_in_media	.0002006	1	.	.0025541
Impulsivity_SC4	.0067023	1	.0020682	.0138919
NegEmo_SC6	.0005867	1	.	.0036763
SN_SC7	.0014344	1	.	.0055078
CTC_SC7	.1651387	1	.1416755	.1890626
OTC_SC7	.0024	1	.0001518	.0072691
NOO_SC3	.0011995	1	.	.00504
wave_d1#c.NOO_SC3	.0012153	1	.	.005072
NOO_SC3	.0011995	1	.	.00504
wave_d2#c.NOO_SC3	.0029452	1	.0003153	.0081906

Note: Eta-Squared values for individual model terms are partial.

```
446 .
447 . *17.a.6 Regression with vce(ro)
448 . reg DV_Compliance_SC7 i.wave_d1 i.wave_d2 Age i.Gender_Female i.Minority Education i.Employed i.Corona_care i.Insura
    > rvative_01 i.Conservative_other i.GeoCensus_d1 i.GeoCensus_d2 i.GeoCensus_d3 i.Current_measures Measures_clear MA_Pe
    > _SC2 NOO_SC3 NNOO_SC3 OOL_SC12 PJE_SC4 Trust_Science_SC4 Trust_in_media Impulsivity_SC4 NegEmo_SC6 SN_SC7 CTC_SC7 OT
    note: NOO_SC3 omitted because of collinearity
    note: NOO_SC3 omitted because of collinearity

    Linear regression                               Number of obs     =      2,919
                                                    F(40, 2878)       =      68.02
                                                    Prob > F          =     0.0000
                                                    R-squared         =     0.5214
                                                    Root MSE          =     .90278
```

DV_Compliance_SC7	Coef.	Robust Std. Err.	t	P>|t|	[95% Conf.	Interval]
1.wave_d1	-.4272575	.2053206	-2.08	0.038	-.8298477	-.0246673
1.wave_d2	-.6747006	.2047551	-3.30	0.001	-1.076182	-.2732192
Age	.0034074	.0014369	2.37	0.018	.00059	.0062248
1.Gender_Female	.1181739	.0353999	3.34	0.001	.0487622	.1875856
1.Minority	-.00795	.0388277	-0.20	0.838	-.084083	.068183
Education	.0337292	.0120922	2.79	0.005	.010019	.0574395
1.Employed	.0125576	.0403102	0.31	0.755	-.0664822	.0915974
1.Corona_care	-.0516802	.0700872	-0.74	0.461	-.1891063	.085746
1.Insurance_Public	.0974178	.0599376	1.63	0.104	-.0201072	.2149428
1.Insurance_Private	.138714	.062497	2.22	0.027	.0161706	.2612573
SES_before	.0047463	.0103236	0.46	0.646	-.0154961	.0249888
SES_change	-.0000636	.0123637	-0.01	0.996	-.0243062	.024179
2.Health_self	.0293585	.0369759	0.79	0.427	-.0431435	.1018605
2.Health_other	-.0164704	.0377738	-0.44	0.663	-.0905368	.0575959
1.Conservative_01	.0473948	.0407608	1.16	0.245	-.0325286	.1273182
1.Conservative_other	.1075377	.0614289	1.75	0.080	-.0129114	.2279868
1.GeoCensus_d1	-.0994982	.0517711	-1.92	0.055	-.2010105	.002014
1.GeoCensus_d2	-.1268284	.0441953	-2.87	0.004	-.2134861	-.0401707
1.GeoCensus_d3	-.0048226	.051533	-0.09	0.925	-.1058679	.0962228
Current_measures						
Yes	.1783088	.0638972	2.79	0.005	.0530199	.3035976
Measures_clear	-.0195489	.0121691	-1.61	0.108	-.0434098	.004312
MA_Perc_Threat_SC3	.1280031	.019734	6.49	0.000	.0893089	.1666974
Costs_SC5	.026903	.0126221	2.13	0.033	.0021537	.0516524
Deterr_SD_Likely_SC2	.0048946	.0122736	0.40	0.690	-.0191713	.0289606
Deterr_SD_Severe	-.0036063	.0111047	-0.32	0.745	-.0253801	.0181676
MA_MoralBelief	.2459066	.0258004	9.53	0.000	.1953176	.2964957
MA_Authority_SC2	-.0073349	.0108804	-0.67	0.500	-.0286692	.0139993
NOO_SC3	-.0598282	.0361155	-1.66	0.098	-.130643	.0109866
NNOO_SC3	.0216432	.0214597	1.01	0.313	-.0204347	.0637211
OOL_SC12	.0165182	.0146431	1.13	0.259	-.0121938	.0452302
PJE_SC4	-.0100221	.0119599	-0.84	0.402	-.0334729	.0134287
Trust_Science_SC4	.035609	.0235491	1.51	0.131	-.0105659	.0817839
Trust_in_media	-.0119528	.0149912	-0.80	0.425	-.0413473	.0174417
Impulsivity_SC4	-.0839002	.01862	-4.51	0.000	-.1204102	-.0473903
NegEmo_SC6	.0164111	.0128594	1.28	0.202	-.0088035	.0416257
SN_SC7	.0296339	.0145853	2.03	0.042	.0010351	.0582327
CTC_SC7	.5161626	.0317088	16.28	0.000	.4539884	.5783368
OTC_SC7	-.0268345	.0097423	-2.75	0.006	-.0459371	-.0077318
NOO_SC3	0	(omitted)				
wave_d1#c.NOO_SC3						
1	.0863083	.0497918	1.73	0.083	-.011323	.1839396
NOO_SC3	0	(omitted)				
wave_d2#c.NOO_SC3						
1	.1354807	.0496971	2.73	0.006	.0380352	.2329262
_cons	.2058956	.2427935	0.85	0.396	-.2701712	.6819623

```
449 .
450 . *17.a.7 Simple main effects
451 . reg DV_Compliance_SC7 i.WAVE Age i.Gender_Female i.Minority Education i.Employed i.Corona_care i.Insurance_Public i.
    > Conservative_other i.GeoCensus_d1 i.GeoCensus_d2 i.GeoCensus_d3 i.Current_measures Measures_clear MA_Perc_Threat_SC3
    > NNOO_SC3 OOL_SC12 PJE_SC4 Trust_Science_SC4 Trust_in_media Impulsivity_SC4 NegEmo_SC6 SN_SC7 CTC_SC7 OTC_SC7 c.NOO_S
    note: NOO_SC3 omitted because of collinearity

    Linear regression                               Number of obs     =      2,919
                                                    F(40, 2878)       =      68.02
                                                    Prob > F          =     0.0000
                                                    R-squared         =     0.5214
                                                    Root MSE          =     .90278
```

DV_Compliance_SC7	Coef.	Robust Std. Err.	t	P>|t|	[95% Conf.	Interval]
WAVE						
2	-.4272575	.2053206	-2.08	0.038	-.8298477	-.0246673
3	-.6747006	.2047551	-3.30	0.001	-1.076182	-.2732192
Age	.0034074	.0014369	2.37	0.018	.00059	.0062248
1.Gender_Female	.1181739	.0353999	3.34	0.001	.0487622	.1875856
1.Minority	-.00795	.0388277	-0.20	0.838	-.084083	.068183
Education	.0337292	.0120922	2.79	0.005	.010019	.0574395
1.Employed	.0125576	.0403102	0.31	0.755	-.0664822	.0915974
1.Corona_care	-.0516802	.0700872	-0.74	0.461	-.1891063	.085746
1.Insurance_Public	.0974178	.0599376	1.63	0.104	-.0201072	.2149428
1.Insurance_Private	.138714	.062497	2.22	0.027	.0161706	.2612573
SES_before	.0047463	.0103236	0.46	0.646	-.0154961	.0249888
SES_change	-.0000636	.0123637	-0.01	0.996	-.0243062	.024179
2.Health_self	.0293585	.0369759	0.79	0.427	-.0431435	.1018605
2.Health_other	-.0164704	.0377738	-0.44	0.663	-.0905368	.0575959
1.Conservative_01	.0473948	.0407608	1.16	0.245	-.0325286	.1273182
1.Conservative_other	.1075377	.0614289	1.75	0.080	-.0129114	.2279868
1.GeoCensus_d1	-.0994982	.0517711	-1.92	0.055	-.2010105	.002014
1.GeoCensus_d2	-.1268284	.0441953	-2.87	0.004	-.2134861	-.0401707
1.GeoCensus_d3	-.0048226	.051533	-0.09	0.925	-.1058679	.0962228
Current_measures						
Yes	.1783088	.0638972	2.79	0.005	.0530199	.3035976
Measures_clear	-.0195489	.0121691	-1.61	0.108	-.0434098	.004312
MA_Perc_Threat_SC3	.1280031	.019734	6.49	0.000	.0893089	.1666974
Costs_SC5	.026903	.0126221	2.13	0.033	.0021537	.0516524
Deterr_SD_Likely_SC2	.0048946	.0122736	0.40	0.690	-.0191713	.0289606
Deterr_SD_Severe	-.0036063	.0111047	-0.32	0.745	-.0253801	.0181676
MA_MoralBelief	.2459066	.0258004	9.53	0.000	.1953176	.2964957
MA_Authority_SC2	-.0073349	.0108804	-0.67	0.500	-.0286692	.0139993
NOO_SC3	-.0598282	.0361155	-1.66	0.098	-.130643	.0109866
NNOO_SC3	.0216432	.0214597	1.01	0.313	-.0204347	.0637211
OOL_SC12	.0165182	.0146431	1.13	0.259	-.0121938	.0452302
PJE_SC4	-.0100221	.0119599	-0.84	0.402	-.0334729	.0134287
Trust_Science_SC4	.035609	.0235491	1.51	0.131	-.0105659	.0817839
Trust_in_media	-.0119528	.0149912	-0.80	0.425	-.0413473	.0174417
Impulsivity_SC4	-.0839002	.01862	-4.51	0.000	-.1204102	-.0473903
NegEmo_SC6	.0164111	.0128594	1.28	0.202	-.0088035	.0416257
SN_SC7	.0296339	.0145853	2.03	0.042	.0010351	.0582327
CTC_SC7	.5161626	.0317088	16.28	0.000	.4539884	.5783368
OTC_SC7	-.0268345	.0097423	-2.75	0.006	-.0459371	-.0077318
NOO_SC3	0	(omitted)				
WAVE#c.NOO_SC3						
2	.0863083	.0497918	1.73	0.083	-.011323	.1839396
3	.1354807	.0496971	2.73	0.006	.0380352	.2329262
_cons	.2058956	.2427935	0.85	0.396	-.2701712	.6819623

```
452 .
453 . margins WAVE, dydx(NOO_SC3)

    Average marginal effects                    Number of obs     =      2,919
    Model VCE    : Robust

    Expression   : Linear prediction, predict()
    dy/dx w.r.t. : NOO_SC3
```

```
------------------------------------------------------------------------------
             |            Delta-method
             |      dy/dx   Std. Err.      t    P>|t|     [95% Conf. Interval]
-------------+----------------------------------------------------------------
NOO_SC3      |
        WAVE |
          1  |  -.0598282   .0361155    -1.66   0.098     -.130643    .0109866
          2  |   .0264801   .0388486     0.68   0.496    -.0496937    .1026539
          3  |   .0756525   .0410928     1.84   0.066    -.0049218    .1562268
------------------------------------------------------------------------------

454 . margins WAVE, dydx(NOO_SC3) pwcompare(effects)

    Pairwise comparisons of average marginal effects

    Model VCE    : Robust                          Number of obs     =      2,919

    Expression   : Linear prediction, predict()
    dy/dx w.r.t. : NOO_SC3

------------------------------------------------------------------------------
             |   Contrast Delta-method    Unadjusted           Unadjusted
             |      dy/dx   Std. Err.      t    P>|t|     [95% Conf. Interval]
-------------+----------------------------------------------------------------
NOO_SC3      |
        WAVE |
     2 vs 1  |   .0863083   .0497918     1.73   0.083     -.011323    .1839396
     3 vs 1  |   .1354807   .0496971     2.73   0.006     .0380352    .2329262
     3 vs 2  |   .0491724    .052326     0.94   0.347    -.0534278    .1517726
------------------------------------------------------------------------------

455 .
456 .
457 . *****************
458 .
459 . *18. Model 18: Non-normative obligation x Survey Wave
460 .
461 . *18.a.2 Regression
462 . reg DV_Compliance_SC7 i.wave_d1 i.wave_d2 Age i.Gender_Female i.Minority Education i.Employed i.Corona_care i.Insura
    > rvative_01 i.Conservative_other i.GeoCensus_d1 i.GeoCensus_d2 i.GeoCensus_d3 i.Current_measures Measures_clear MA_Pe
    > _SC2 NOO_SC3 NNOO_SC3 OOL_SC12 PJE_SC4 Trust_Science_SC4 Trust_in_media Impulsivity_SC4 NegEmo_SC6 SN_SC7 CTC_SC7 OT
    note: NNOO_SC3 omitted because of collinearity
    note: NNOO_SC3 omitted because of collinearity

          Source |       SS           df       MS      Number of obs   =     2,919
    -------------+----------------------------------   F(40, 2878)     =     78.79
           Model |  2561.46646        40  64.0366614   Prob > F        =    0.0000
        Residual |  2339.06404     2,878  .812739417   R-squared       =    0.5227
    -------------+----------------------------------   Adj R-squared   =    0.5161
           Total |   4900.5305     2,918  1.67941415   Root MSE        =    .90152

    ------------------------------------------------------------------------------
    DV_Compliance_SC7 |      Coef.   Std. Err.      t    P>|t|     [95% Conf. Interval]
    ------------------+----------------------------------------------------------------
            1.wave_d1 |  -.3619409   .1264868    -2.86   0.004    -.6099548    -.113927
            1.wave_d2 |  -.6449025   .1305204    -4.94   0.000    -.9008254   -.3889797
                  Age |   .0034343   .0014382     2.39   0.017     .0006143    .0062543
      1.Gender_Female |   .1233372   .0353095     3.49   0.000     .0541027    .1925717
           1.Minority |  -.0039841   .0377053    -0.11   0.916    -.0779163    .0699481
            Education |   .0343665   .0122519     2.80   0.005     .0103432    .0583898
           1.Employed |   .0149332    .039141     0.38   0.703    -.0618141    .0916805
        1.Corona_care |  -.0517896   .0651754    -0.79   0.427    -.1795848    .0760056
   1.Insurance_Public |   .0893862   .0536432     1.67   0.096    -.0157967    .1945692
  1.Insurance_Private |   .1290411   .0560991     2.30   0.022     .0190426    .2390396
           SES_before |   .0042924   .0095361     0.45   0.653    -.0144058    .0229906
           SES_change |   .0004289   .0112168     0.04   0.970    -.0215648    .0224225
        2.Health_self |   .0314617   .0410144     0.77   0.443    -.0489589    .1118822
       2.Health_other |  -.0219358   .0389852    -0.56   0.574    -.0983775    .0545059
     1.Conservative_01 |   .0534097   .0402671     1.33   0.185    -.0255457     .132365
 1.Conservative_other |   .1095205   .0577489     1.90   0.058    -.0037128    .2227538
       1.GeoCensus_d1 |  -.0952879   .0529771    -1.80   0.072    -.1991649     .008589
       1.GeoCensus_d2 |  -.1254428   .0459402    -2.73   0.006    -.2155219   -.0353637
       1.GeoCensus_d3 |   -.002088   .0564029    -0.04   0.970    -.1126823    .1085062
```

```
    Current_measures |
                 Yes |     .18125   .0521942     3.47   0.001     .0789082    .2835918
      Measures_clear |  -.0180895   .0115007    -1.57   0.116    -.0406398    .0044609
    MA_Perc_Threat_SC3 |   .1282729   .0171558     7.48   0.000      .094634    .1619118
           Costs_SC5 |    .026148   .0122293     2.14   0.033     .0021689    .0501271
  Deterr_SD_Likely_SC2 |   .0069372   .0120503     0.58   0.565    -.0166908    .0305652
    Deterr_SD_Severe |  -.0043552   .0106025    -0.41   0.681    -.0251446    .0164341
      MA_MoralBelief |   .2443852   .0194291    12.58   0.000     .2062888    .2824815
    MA_Authority_SC2 |  -.0102796   .0112579    -0.91   0.361     -.032354    .0117948
             NOO_SC3 |   .0176349   .0246087     0.72   0.474    -.0306175    .0658873
            NNOO_SC3 |  -.0657479    .031414    -2.09   0.036    -.1273441   -.0041517
            OOL_SC12 |   .0148701   .0141737     1.05   0.294    -.0129215    .0426617
             PJE_SC4 |  -.0109844   .0121338    -0.91   0.365    -.0347762    .0128075
   Trust_Science_SC4 |   .0393511   .0219938     1.79   0.074    -.0037741    .0824762
      Trust_in_media |  -.0149686   .0157182    -0.95   0.341    -.0457888    .0158516
     Impulsivity_SC4 |  -.0846042   .0190052    -4.45   0.000    -.1218693    -.047339
          NegEmo_SC6 |   .0164024   .0126056     1.30   0.193    -.0083145    .0411193
             SN_SC7 |   .0307633   .0145691     2.11   0.035     .0021963    .0593303
             CTC_SC7 |   .5166562    .021592    23.93   0.000     .4743188    .5589936
             OTC_SC7 |   -.025201    .010184    -2.47   0.013    -.0451697   -.0052323
            NNOO_SC3 |          0  (omitted)
                     |
   wave_d1#c.NNOO_SC3 |
                   1 |   .0935625    .040387     2.32   0.021     .0143722    .1727529
                     |
            NNOO_SC3 |          0  (omitted)
                     |
   wave_d2#c.NNOO_SC3 |
                   1 |   .1709001   .0419428     4.07   0.000     .0886591    .2531412
                     |
               _cons |   .1563584   .2102512     0.74   0.457    -.2558998    .5686166
-------------------------------------------------------------------------------------

463 .
464 . *18.a.3 Check hettest: Run this right after your regression to apply the Breusch-Pagan / Cook-Weisberg test for hete
465 . *if significant, then you need to run the regression with vce(ro) at the end
466 . estat hettest

    Breusch-Pagan / Cook-Weisberg test for heteroskedasticity
             Ho: Constant variance
             Variables: fitted values of DV_Compliance_SC7

             chi2(1)      =   366.16
             Prob > chi2  =   0.0000

467 .
468 . *18.a.4. check vif, to check for for multicollinearity (VIFs >10 are problematic)
469 . vif

        Variable |       VIF       1/VIF
    -------------+----------------------
       1.wave_d1 |     12.85    0.077801
       1.wave_d2 |     13.21    0.075679
             Age |      1.27    0.790138
    1.Gender_F~e |      1.11    0.900716
      1.Minority |      1.15    0.869155
       Education |      1.28    0.779492
      1.Employed |      1.27    0.787773
    1.Corona_c~e |      1.22    0.819232
    1.Insuranc~c |      2.54    0.394327
    1.Insuran~te |      2.35    0.426293
      SES_before |      1.37    0.728784
      SES_change |      1.24    0.808795
    2.Health_s~f |      1.35    0.738128
    2.Health_o~r |      1.33    0.753947
    1.Conserv~01 |      1.44    0.693205
    1.Conserva~r |      1.26    0.791164
    1.GeoCensu~1 |      1.66    0.603060
    1.GeoCensu~2 |      1.86    0.538915
    1.GeoCensu~3 |      1.54    0.650119
    1.Current_~s |      1.14    0.879123
    Measures_c~r |      1.42    0.705485
    MA_Perc_Th~3 |      2.40    0.416914
       Costs_SC5 |      1.44    0.692515
```

Deterr_SD_~2	1.62	0.617241
Deterr_SD_~e	1.19	0.839151
MA_MoralBe~f	2.28	0.439507
MA_Authori~2	1.63	0.614764
NOO_SC3	1.77	0.566315
NNOO_SC3	3.53	0.283417
OOL_SC12	1.59	0.630682
PJE_SC4	1.38	0.726563
Trust_Scie~4	1.69	0.591530
Trust_in_m~a	1.53	0.655358
Impulsivi~C4	1.64	0.610268
NegEmo_SC6	1.40	0.712622
SN_SC7	1.49	0.673311
CTC_SC7	1.64	0.609803
OTC_SC7	1.14	0.873961
wave_d1#		
c.NNOO_SC3		
1	13.62	0.073402
wave_d2#		
c.NNOO_SC3		
1	13.71	0.072931
Mean VIF	2.76	

```
470 .
471 . *18.a.5. Effect size
472 . estat esize
```

Effect sizes for linear models

Source	Eta-Squared	df	[95% Conf.	Interval]
Model	.5226917	40	.4935849	.5374323
wave_d1	.002837	1	.0002802	.0080108
wave_d2	.0084115	1	.0030667	.0162842
Age	.0019773	1	.0000488	.0065233
Gender_Female	.0042216	1	.0008129	.0102258
Minority	3.88e-06	1	.	.0008452
Education	.0027264	1	.0002456	.0078255
Employed	.0000506	1	.	.0017996
Corona_care	.0002193	1	.	.0026214
Insurance_Public	.0009638	1	.	.004546
Insurance_Private	.0018351	1	.0000184	.0062644
SES_before	.0000704	1	.	.0019476
SES_change	5.08e-07	1	.	.000138
Health_self	.0002044	1	.	.0025679
Health_other	.00011	1	.	.0021732
Conservative_01	.0006109	1	.	.0037364
Conservative_other	.0012482	1	.	.0051386
GeoCensus_d1	.0011228	1	.	.0048824
GeoCensus_d2	.002584	1	.0002032	.0075846
GeoCensus_d3	4.76e-07	1	.	.0001156
Current_measures	.0041726	1	.0007914	.01015
Measures_clear	.0008589	1	.	.0043157
MA_Perc_Threat_SC3	.0190547	1	.0104199	.030049
Costs_SC5	.001586	1	.	.0057993
Deterr_SD_Likely_SC2	.0001151	1	.	.0021985
Deterr_SD_Severe	.0000586	1	.	.0018641
MA_MoralBelief	.052109	1	.0374844	.0685969
MA_Authority_SC2	.0002896	1	.	.0028555
NOO_SC3	.0001784	1	.	.0024703
NNOO_SC3	.0025883	1	.0002044	.007592
OOL_SC12	.0003823	1	.	.0031335
PJE_SC4	.0002847	1	.	.0028398
Trust_Science_SC4	.0011111	1	.	.0048579
Trust_in_media	.000315	1	.	.0029345
Impulsivity_SC4	.0068386	1	.0021446	.0140858
NegEmo_SC6	.000588	1	.	.0036795
SN_SC7	.0015468	1	.	.0057246
CTC_SC7	.1659309	1	.1424343	.1898799
OTC_SC7	.0021232	1	.0000821	.0067844
NNOO_SC3	.0025883	1	.0002044	.007592

```
   wave_d1#c.NNOO_SC3 |   .0018613       1     .0000239    .0063125
             NNOO_SC3 |   .0025883       1     .0002044     .007592
   wave_d2#c.NNOO_SC3 |   .0057356       1     .0015456    .0124966
-------------------------------------------------------------------

Note: Eta-Squared values for individual model terms are partial.

473 .
474 . *18.a.6 Regression with vce(ro)
475 . reg DV_Compliance_SC7 i.wave_d1 i.wave_d2 Age i.Gender_Female i.Minority Education i.Employed i.Corona_care i.Insura
    > rvative_01 i.Conservative_other i.GeoCensus_d1 i.GeoCensus_d2 i.GeoCensus_d3 i.Current_measures Measures_clear MA_Pe
    > _SC2 NOO_SC3 NNOO_SC3 OOL_SC12 PJE_SC4 Trust_Science_SC4 Trust_in_media Impulsivity_SC4 NegEmo_SC6 SN_SC7 CTC_SC7 OT
note: NNOO_SC3 omitted because of collinearity
note: NNOO_SC3 omitted because of collinearity

Linear regression                               Number of obs     =      2,919
                                                F(40, 2878)       =      68.53
                                                Prob > F          =     0.0000
                                                R-squared         =     0.5227
                                                Root MSE          =     .90152
```

DV_Compliance_SC7	Coef.	Robust Std. Err.	t	P>|t|	[95% Conf.	Interval]
1.wave_d1	-.3619409	.1258065	-2.88	0.004	-.6086208	-.115261
1.wave_d2	-.6449025	.1268506	-5.08	0.000	-.8936298	-.3961753
Age	.0034343	.0014406	2.38	0.017	.0006096	.006259
1.Gender_Female	.1233372	.0354146	3.48	0.001	.0538967	.1927776
1.Minority	-.0039841	.0387694	-0.10	0.918	-.0800027	.0720345
Education	.0343665	.0120038	2.86	0.004	.0108295	.0579035
1.Employed	.0149332	.0403554	0.37	0.711	-.0641953	.0940617
1.Corona_care	-.0517896	.0699138	-0.74	0.459	-.1888757	.0852966
1.Insurance_Public	.0893862	.0599382	1.49	0.136	-.0281399	.2069123
1.Insurance_Private	.1290411	.0624228	2.07	0.039	.0066433	.2514389
SES_before	.0042924	.0102514	0.42	0.675	-.0158085	.0243933
SES_change	.0004289	.0123959	0.03	0.972	-.0238768	.0247345
2.Health_self	.0314617	.0368278	0.85	0.393	-.0407499	.1036732
2.Health_other	-.0219358	.0376471	-0.58	0.560	-.0957538	.0518822
1.Conservative_01	.0534097	.0406229	1.31	0.189	-.0262432	.1330626
1.Conservative_other	.1095205	.0611387	1.79	0.073	-.0103596	.2294006
1.GeoCensus_d1	-.0952879	.0518179	-1.84	0.066	-.1968918	.0063159
1.GeoCensus_d2	-.1254428	.044026	-2.85	0.004	-.2117684	-.0391171
1.GeoCensus_d3	-.002088	.0512972	-0.04	0.968	-.1026709	.0984949
Current_measures						
Yes	.18125	.0635842	2.85	0.004	.0565749	.3059251
Measures_clear	-.0180895	.0121168	-1.49	0.136	-.0418479	.0056689
MA_Perc_Threat_SC3	.1282729	.0196787	6.52	0.000	.0896871	.1668587
Costs_SC5	.026148	.0126028	2.07	0.038	.0014367	.0508594
Deterr_SD_Likely_SC2	.0069372	.0122585	0.57	0.571	-.017099	.0309735
Deterr_SD_Severe	-.0043552	.0110998	-0.39	0.695	-.0261197	.0174092
MA_MoralBelief	.2443852	.0257064	9.51	0.000	.1939804	.2947899
MA_Authority_SC2	-.0102796	.0108811	-0.94	0.345	-.0316152	.011056
NOO_SC3	.0176349	.0256189	0.69	0.491	-.0325984	.0678682
NNOO_SC3	-.0657479	.0325468	-2.02	0.043	-.1295653	-.0019305
OOL_SC12	.0148701	.0146676	1.01	0.311	-.0138899	.0436302
PJE_SC4	-.0109844	.0119369	-0.92	0.358	-.0343901	.0124213
Trust_Science_SC4	.0393511	.0233999	1.68	0.093	-.0065313	.0852334
Trust_in_media	-.0149686	.0149682	-1.00	0.317	-.044318	.0143808
Impulsivity_SC4	-.0846042	.0186586	-4.53	0.000	-.1211898	-.0480185
NegEmo_SC6	.0164024	.012874	1.27	0.203	-.0088408	.0416456
SN_SC7	.0307633	.0146311	2.10	0.036	.0020748	.0594517
CTC_SC7	.5166562	.0317097	16.29	0.000	.4544802	.5788322
OTC_SC7	-.025201	.009754	-2.58	0.010	-.0443266	-.0060754
NNOO_SC3	0	(omitted)				
wave_d1#c.NNOO_SC3						
1	.0935625	.0421005	2.22	0.026	.0110123	.1761128
NNOO_SC3	0	(omitted)				
wave_d2#c.NNOO_SC3						
1	.1709001	.0424806	4.02	0.000	.0876046	.2541956

_cons	.1563584	.2241886	0.70	0.486	-.2832281	.5959449

```
476 .
477 . *18.a.7 Simple main effects
478 . reg DV_Compliance_SC7 i.WAVE Age i.Gender_Female i.Minority Education i.Employed i.Corona_care i.Insurance_Public i.
    > Conservative_other i.GeoCensus_d1 i.GeoCensus_d2 i.GeoCensus_d3 i.Current_measures Measures_clear MA_Perc_Threat_SC3
    > NNOO_SC3 OOL_SC12 PJE_SC4 Trust_Science_SC4 Trust_in_media Impulsivity_SC4 NegEmo_SC6 SN_SC7 CTC_SC7 OTC_SC7 c.NNOO_
    note: NNOO_SC3 omitted because of collinearity
```

Linear regression

Number of obs	=	2,919
F(40, 2878)	=	68.53
Prob > F	=	0.0000
R-squared	=	0.5227
Root MSE	=	.90152

DV_Compliance_SC7	Coef.	Robust Std. Err.	t	P>|t|	[95% Conf.	Interval]
WAVE						
2	-.3619409	.1258065	-2.88	0.004	-.6086208	-.115261
3	-.6449025	.1268506	-5.08	0.000	-.8936298	-.3961753
Age	.0034343	.0014406	2.38	0.017	.0006096	.006259
1.Gender_Female	.1233372	.0354146	3.48	0.001	.0538967	.1927776
1.Minority	-.0039841	.0387694	-0.10	0.918	-.0800027	.0720345
Education	.0343665	.0120038	2.86	0.004	.0108295	.0579035
1.Employed	.0149332	.0403554	0.37	0.711	-.0641953	.0940617
1.Corona_care	-.0517896	.0699138	-0.74	0.459	-.1888757	.0852966
1.Insurance_Public	.0893862	.0599382	1.49	0.136	-.0281399	.2069123
1.Insurance_Private	.1290411	.0624228	2.07	0.039	.0066433	.2514389
SES_before	.0042924	.0102514	0.42	0.675	-.0158085	.0243933
SES_change	.0004289	.0123959	0.03	0.972	-.0238768	.0247345
2.Health_self	.0314617	.0368278	0.85	0.393	-.0407499	.1036732
2.Health_other	-.0219358	.0376471	-0.58	0.560	-.0957538	.0518822
1.Conservative_01	.0534097	.0406229	1.31	0.189	-.0262432	.1330626
1.Conservative_other	.1095205	.0611387	1.79	0.073	-.0103596	.2294006
1.GeoCensus_d1	-.0952879	.0518179	-1.84	0.066	-.1968918	.0063159
1.GeoCensus_d2	-.1254428	.044026	-2.85	0.004	-.2117684	-.0391171
1.GeoCensus_d3	-.002088	.0512972	-0.04	0.968	-.1026709	.0984949
Current_measures						
Yes	.18125	.0635842	2.85	0.004	.0565749	.3059251
Measures_clear	-.0180895	.0121168	-1.49	0.136	-.0418479	.0056689
MA_Perc_Threat_SC3	.1282729	.0196787	6.52	0.000	.0896871	.1668587
Costs_SC5	.026148	.0126028	2.07	0.038	.0014367	.0508594
Deterr_SD_Likely_SC2	.0069372	.0122585	0.57	0.571	-.017099	.0309735
Deterr_SD_Severe	-.0043552	.0110998	-0.39	0.695	-.0261197	.0174092
MA_MoralBelief	.2443852	.0257064	9.51	0.000	.1939804	.2947899
MA_Authority_SC2	-.0102796	.0108811	-0.94	0.345	-.0316152	.011056
NOO_SC3	.0176349	.0256189	0.69	0.491	-.0325984	.0678682
NNOO_SC3	-.0657479	.0325468	-2.02	0.043	-.1295653	-.0019305
OOL_SC12	.0148701	.0146676	1.01	0.311	-.0138899	.0436302
PJE_SC4	-.0109844	.0119369	-0.92	0.358	-.0343901	.0124213
Trust_Science_SC4	.0393511	.0233999	1.68	0.093	-.0065313	.0852334
Trust_in_media	-.0149686	.0149682	-1.00	0.317	-.044318	.0143808
Impulsivity_SC4	-.0846042	.0186586	-4.53	0.000	-.1211898	-.0480185
NegEmo_SC6	.0164024	.012874	1.27	0.203	-.0088408	.0416456
SN_SC7	.0307633	.0146311	2.10	0.036	.0020748	.0594517
CTC_SC7	.5166562	.0317097	16.29	0.000	.4544802	.5788322
OTC_SC7	-.025201	.009754	-2.58	0.010	-.0443266	-.0060754
NNOO_SC3	0	(omitted)				
WAVE#c.NNOO_SC3						
2	.0935625	.0421005	2.22	0.026	.0110123	.1761128
3	.1709001	.0424806	4.02	0.000	.0876046	.2541956
_cons	.1563584	.2241886	0.70	0.486	-.2832281	.5959449

```
479 .
480 . margins WAVE, dydx(NNOO_SC3)

    Average marginal effects                        Number of obs     =      2,919
    Model VCE    : Robust

    Expression   : Linear prediction, predict()
    dy/dx w.r.t. : NNOO_SC3
```

	dy/dx	Delta-method Std. Err.	t	P>|t|	[95% Conf.	Interval]
**NNOO_SC3**						
WAVE						
1	-.0657479	.0325468	-2.02	0.043	-.1295653	-.0019305
2	.0278147	.0333354	0.83	0.404	-.0375491	.0931784
3	.1051523	.0321576	3.27	0.001	.042098	.1682065

```
481 . margins WAVE, dydx(NNOO_SC3) pwcompare(effects)

    Pairwise comparisons of average marginal effects

    Model VCE    : Robust                           Number of obs     =      2,919

    Expression   : Linear prediction, predict()
    dy/dx w.r.t. : NNOO_SC3
```

	Contrast dy/dx	Delta-method Std. Err.	Unadjusted t	Unadjusted P>|t|	Unadjusted [95% Conf.	Interval]
**NNOO_SC3**						
WAVE						
2 vs 1	.0935625	.0421005	2.22	0.026	.0110123	.1761128
3 vs 1	.1709001	.0424806	4.02	0.000	.0876046	.2541956
3 vs 2	.0773376	.0437623	1.77	0.077	-.0084709	.1631462

```
482 .
483 .
484 . *****************
485 .
486 . *19. Model 19: PJ x Survey Wave
487 .
488 . *19.a.2 Regression
489 . reg DV_Compliance_SC7 i.wave_d1 i.wave_d2 Age i.Gender_Female i.Minority Education i.Employed i.Corona_care i.Insura
    > rvative_01 i.Conservative_other i.GeoCensus_d1 i.GeoCensus_d2 i.GeoCensus_d3 i.Current_measures Measures_clear MA_Pe
    > _SC2 NOO_SC3 NNOO_SC3 OOL_SC12 PJE_SC4 Trust_Science_SC4 Trust_in_media Impulsivity_SC4 NegEmo_SC6 SN_SC7 CTC_SC7 OT
    note: PJE_SC4 omitted because of collinearity
    note: PJE_SC4 omitted because of collinearity
```

Source	SS	df	MS			
				Number of obs	=	2,919
				F(40, 2878)	=	78.11
Model	2550.91397	40	63.7728493	Prob > F	=	0.0000
Residual	2349.61653	2,878	.816406021	R-squared	=	0.5205
				Adj R-squared	=	0.5139
Total	4900.5305	2,918	1.67941415	Root MSE	=	.90355

DV_Compliance_SC7	Coef.	Std. Err.	t	P>|t|	[95% Conf.	Interval]
1.wave_d1	-.3297074	.1387078	-2.38	0.018	-.601684	-.0577308
1.wave_d2	-.3458197	.1420066	-2.44	0.015	-.6242646	-.0673748
Age	.0034924	.0014403	2.42	0.015	.0006682	.0063165
1.Gender_Female	.1210695	.0353802	3.42	0.001	.0516965	.1904425
1.Minority	-.0065321	.0377984	-0.17	0.863	-.0806467	.0675825
Education	.0340323	.0122936	2.77	0.006	.0099272	.0581375
1.Employed	.0156585	.0392286	0.40	0.690	-.0612605	.0925776
1.Corona_care	-.0476789	.0653145	-0.73	0.465	-.175747	.0803891
1.Insurance_Public	.091314	.0537979	1.70	0.090	-.0141722	.1968003
1.Insurance_Private	.1336775	.0562168	2.38	0.017	.0234483	.2439068
SES_before	.0047448	.0095557	0.50	0.620	-.0139918	.0234815

```
          SES_change |   .0003296    .011239     0.03   0.977    -.0217077    .0223668
       2.Health_self |   .0338468    .041101     0.82   0.410    -.0467436    .1144373
      2.Health_other |  -.0171302   .0390765    -0.44   0.661     -.093751    .0594906
    1.Conservative_01 |   .0473594   .0403749     1.17   0.241    -.0318073    .1265262
1.Conservative_other |   .1055709   .0579159     1.82   0.068      -.00799    .2191317
      1.GeoCensus_d1 |  -.1002147    .053099    -1.89   0.059    -.2043307    .0039012
      1.GeoCensus_d2 |   -.128038   .0460703    -2.78   0.005     -.218372   -.0377039
      1.GeoCensus_d3 |  -.0039842   .0565883    -0.07   0.944    -.1149419    .1069735
                     |
    Current_measures |
                 Yes |   .1765439    .052342     3.37   0.001     .0739122    .2791755
      Measures_clear |  -.0191393   .0115261    -1.66   0.097    -.0417394    .0034609
    MA_Perc_Threat_SC3 |   .1250531   .0171866     7.28   0.000     .0913538    .1587525
           Costs_SC5 |   .0262159   .0122591     2.14   0.033     .0021784    .0502534
 Deterr_SD_Likely_SC2 |   .0054171   .0120605     0.45   0.653    -.0182309    .0290651
    Deterr_SD_Severe |  -.0039681   .0106414    -0.37   0.709    -.0248336    .0168974
      MA_MoralBelief |   .2471516   .0194703    12.69   0.000     .2089745    .2853288
    MA_Authority_SC2 |  -.0079224   .0112701    -0.70   0.482    -.0300207    .0141759
            NOO_SC3 |    .017214   .0246697     0.70   0.485    -.0311581     .065586
           NNOO_SC3 |   .0205654   .0210905     0.98   0.330    -.0207886    .0619193
           OOL_SC12 |   .0142039   .0142184     1.00   0.318    -.0136754    .0420832
            PJE_SC4 |   -.039949   .0199012    -2.01   0.045    -.0789711    -.000927
   Trust_Science_SC4 |   .0374717   .0220415     1.70   0.089    -.0057471    .0806906
      Trust_in_media |  -.0121274   .0157394    -0.77   0.441     -.042989    .0187343
     Impulsivity_SC4 |  -.0866642   .0190584    -4.55   0.000    -.1240336   -.0492948
          NegEmo_SC6 |   .0175939   .0126303     1.39   0.164    -.0071715    .0423592
             SN_SC7 |   .0285339   .0145973     1.95   0.051    -.0000883     .057156
            CTC_SC7 |   .5181427   .0216699    23.91   0.000     .4756527    .5606327
            OTC_SC7 |  -.0265291   .0102049    -2.60   0.009    -.0465388   -.0065194
            PJE_SC4 |          0  (omitted)
                     |
   wave_d1#c.PJE_SC4 |
                   1 |   .0470452   .0256748     1.83   0.067    -.0032976     .097388
                     |
            PJE_SC4 |          0  (omitted)
                     |
   wave_d2#c.PJE_SC4 |
                   1 |   .0393588   .0262741     1.50   0.134    -.0121592    .0908768
                     |
               _cons |   .0666328   .2168225     0.31   0.759    -.3585103     .491776
------------------------------------------------------------------------------------

490 .
491 . *19.a.3 Check hettest: Run this right after your regression to apply the Breusch-Pagan / Cook-Weisberg test for hete
492 . *if significant, then you need to run the regression with vce(ro) at the end
493 . estat hettest

Breusch-Pagan / Cook-Weisberg test for heteroskedasticity
         Ho: Constant variance
         Variables: fitted values of DV_Compliance_SC7

         chi2(1)      =   367.86
         Prob > chi2  =   0.0000

494 .
495 . *19.a.4. check vif, to check for for multicollinearity (VIFs >10 are problematic)
496 . vif

    Variable |       VIF       1/VIF
-------------+----------------------
   1.wave_d1 |     15.39    0.064988
   1.wave_d2 |     15.57    0.064220
         Age |      1.26    0.791402
1.Gender_F~e |      1.11    0.901170
  1.Minority |      1.15    0.868784
   Education |      1.29    0.777701
  1.Employed |      1.27    0.787797
1.Corona_c~e |      1.22    0.819425
1.Insuranc~c |      2.54    0.393832
1.Insuran~te |      2.35    0.426425
  SES_before |      1.37    0.729072
  SES_change |      1.24    0.809235
2.Health_s~f |      1.35    0.738336
2.Health_o~r |      1.33    0.753811
```

1.Conserv~01	1.44	0.692618
1.Conserva~r	1.27	0.790156
1.GeoCensu~1	1.66	0.603002
1.GeoCensu~2	1.86	0.538295
1.GeoCensu~3	1.54	0.648780
1.Current_~s	1.14	0.878107
Measures_c~r	1.42	0.705547
MA_Perc_Th~3	2.40	0.417294
Costs_SC5	1.44	0.692266
Deterr_SD_~2	1.62	0.618979
Deterr_SD_~e	1.20	0.836796
MA_MoralBe~f	2.27	0.439622
MA_Authori~2	1.62	0.616203
NOO_SC3	1.77	0.566059
NNOO_SC3	1.58	0.631618
OOL_SC12	1.59	0.629545
PJE_SC4	3.69	0.271309
Trust_Scie~4	1.69	0.591626
Trust_in_m~a	1.52	0.656547
Impulsivi~C4	1.64	0.609607
NegEmo_SC6	1.40	0.713037
SN_SC7	1.48	0.673746
CTC_SC7	1.64	0.608162
OTC_SC7	1.14	0.874313
wave_d1#		
c.PJE_SC4		
1	15.72	0.063629
wave_d2#		
c.PJE_SC4		
1	15.86	0.063044
Mean VIF	3.00	

```
497 .
498 . *19.a.5. Effect size
499 . estat esize
```

Effect sizes for linear models

Source	Eta-Squared	df	[95% Conf.	Interval]
Model	.5205383	40	.4913346	.5353194
wave_d1	.0019594	1	.0000448	.0064908
wave_d2	.0020564	1	.0000665	.0066653
Age	.0020387	1	.0000625	.0066337
Gender_Female	.0040522	1	.0007393	.0099634
Minority	.0000104	1	.	.0011927
Education	.0026557	1	.0002242	.0077063
Employed	.0000554	1	.	.0018388
Corona_care	.0001851	1	.	.0024961
Insurance_Public	.001	1	.	.0046238
Insurance_Private	.0019608	1	.0000452	.0064935
SES_before	.0000857	1	.	.0020424
SES_change	2.99e-07	1	.	.
Health_self	.0002356	1	.	.0026779
Health_other	.0000668	1	.	.001923
Conservative_01	.0004778	1	.	.0033965
Conservative_other	.0011532	1	.	.0049451
GeoCensus_d1	.0012361	1	.	.0051143
GeoCensus_d2	.0026766	1	.0002305	.0077416
GeoCensus_d3	1.72e-06	1	.	.0005622
Current_measures	.0039373	1	.0006905	.0097842
Measures_clear	.0009572	1	.	.0045315
MA_Perc_Threat_SC3	.0180634	1	.0096831	.0288188
Costs_SC5	.0015865	1	.	.0058002
Deterr_SD_Likely_SC2	.0000701	1	.	.0019456
Deterr_SD_Severe	.0000483	1	.	.0017801
MA_MoralBelief	.053019	1	.0382713	.069617
MA_Authority_SC2	.0001717	1	.	.002444
NOO_SC3	.0001691	1	.	.002434
NNOO_SC3	.0003303	1	.	.0029808
OOL_SC12	.0003466	1	.	.0030297

PJE_SC4	.0000187	1	.	.0014065
Trust_Science_SC4	.0010032	1	.	.0046306
Trust_in_media	.0002062	1	.	.0025745
Impulsivity_SC4	.0071336	1	.002312	.0145036
NegEmo_SC6	.0006738	1	.	.0038888
SN_SC7	.0013259	1	.	.0052943
CTC_SC7	.1657302	1	.1422421	.189673
OTC_SC7	.0023427	1	.0001367	.0071699
PJE_SC4	.0000187	1	.	.0014065
wave_d1#c.PJE_SC4	.0011653	1	.	.0049699
PJE_SC4	.0000187	1	.	.0014065
wave_d2#c.PJE_SC4	.0007791	1	.	.0041353

Note: Eta-Squared values for individual model terms are partial.

```
500 .
501 . *19.a.6 Regression with vce(ro)
502 . reg DV_Compliance_SC7 i.wave_d1 i.wave_d2 Age i.Gender_Female i.Minority Education i.Employed i.Corona_care i.Insura
    > rvative_01 i.Conservative_other i.GeoCensus_d1 i.GeoCensus_d2 i.GeoCensus_d3 i.Current_measures Measures_clear MA_Pe
    > _SC2 NOO_SC3 NNOO_SC3 OOL_SC12 PJE_SC4 Trust_Science_SC4 Trust_in_media Impulsivity_SC4 NegEmo_SC6 SN_SC7 CTC_SC7 OT
    note: PJE_SC4 omitted because of collinearity
    note: PJE_SC4 omitted because of collinearity
```

Linear regression

Number of obs	=	2,919
F(40, 2878)	=	67.38
Prob > F	=	0.0000
R-squared	=	0.5205
Root MSE	=	.90355

DV_Compliance_SC7	Coef.	Robust Std. Err.	t	P>|t|	[95% Conf.	Interval]
1.wave_d1	-.3297074	.1401202	-2.35	0.019	-.6044534	-.0549613
1.wave_d2	-.3458197	.1454985	-2.38	0.018	-.6311114	-.060528
Age	.0034924	.0014359	2.43	0.015	.000677	.0063078
1.Gender_Female	.1210695	.0353895	3.42	0.001	.0516782	.1904608
1.Minority	-.0065321	.0389762	-0.17	0.867	-.0829562	.0698919
Education	.0340323	.0120955	2.81	0.005	.0103157	.057749
1.Employed	.0156585	.040402	0.39	0.698	-.0635612	.0948783
1.Corona_care	-.0476789	.0701964	-0.68	0.497	-.1853193	.0899614
1.Insurance_Public	.091314	.0600377	1.52	0.128	-.0264072	.2090353
1.Insurance_Private	.1336775	.0624209	2.14	0.032	.0112834	.2560717
SES_before	.0047448	.0103472	0.46	0.647	-.0155438	.0250335
SES_change	.0003296	.0124108	0.03	0.979	-.0240054	.0246646
2.Health_self	.0338468	.0369106	0.92	0.359	-.038527	.1062207
2.Health_other	-.0171302	.0377524	-0.45	0.650	-.0911548	.0568943
1.Conservative_01	.0473594	.0407619	1.16	0.245	-.0325661	.1272849
1.Conservative_other	.1055709	.0616988	1.71	0.087	-.0154075	.2265492
1.GeoCensus_d1	-.1002147	.0519205	-1.93	0.054	-.2020199	.0015905
1.GeoCensus_d2	-.128038	.0442074	-2.90	0.004	-.2147194	-.0413565
1.GeoCensus_d3	-.0039842	.0514727	-0.08	0.938	-.1049113	.0969429
Current_measures						
Yes	.1765439	.063658	2.77	0.006	.0517241	.3013637
Measures_clear	-.0191393	.0122171	-1.57	0.117	-.0430945	.004816
MA_Perc_Threat_SC3	.1250531	.019723	6.34	0.000	.0863804	.1637259
Costs_SC5	.0262159	.0126183	2.08	0.038	.0014741	.0509576
Deterr_SD_Likely_SC2	.0054171	.0122616	0.44	0.659	-.0186252	.0294595
Deterr_SD_Severe	-.0039681	.0111844	-0.35	0.723	-.0258983	.0179621
MA_MoralBelief	.2471516	.0259499	9.52	0.000	.1962694	.2980338
MA_Authority_SC2	-.0079224	.0108822	-0.73	0.467	-.0292601	.0134153
NOO_SC3	.017214	.0257386	0.67	0.504	-.0332539	.0676819
NNOO_SC3	.0205654	.0215356	0.95	0.340	-.0216614	.0627922
OOL_SC12	.0142039	.014753	0.96	0.336	-.0147235	.0431313
PJE_SC4	-.039949	.0191452	-2.09	0.037	-.0774887	-.0024093
Trust_Science_SC4	.0374717	.0234571	1.60	0.110	-.0085227	.0834662
Trust_in_media	-.0121274	.0150245	-0.81	0.420	-.0415871	.0173324
Impulsivity_SC4	-.0866642	.0187587	-4.62	0.000	-.1234461	-.0498823
NegEmo_SC6	.0175939	.0129036	1.36	0.173	-.0077073	.042895
SN_SC7	.0285339	.0146334	1.95	0.051	-.0001591	.0572269
CTC_SC7	.5181427	.0319517	16.22	0.000	.4554922	.5807932
OTC_SC7	-.0265291	.0097841	-2.71	0.007	-.0457137	-.0073445
PJE_SC4	0	(omitted)				

wave_d1#c.PJE_SC4						
1	.0470452	.025725	1.83	0.068	-.003396	.0974864
PJE_SC4	0	(omitted)				
wave_d2#c.PJE_SC4						
1	.0393588	.0270023	1.46	0.145	-.013587	.0923046
_cons	.0666328	.2262581	0.29	0.768	-.3770115	.5102771

```
503 .
504 .
505 . *****************
506 .
507 . *20. Model 20: Trust in science x Survey Wave
508 .
509 . *20.a.2 Regression
510 . reg DV_Compliance_SC7 i.wave_d1 i.wave_d2 Age i.Gender_Female i.Minority Education i.Employed i.Corona_care i.Insura
    > rvative_01 i.Conservative_other i.GeoCensus_d1 i.GeoCensus_d2 i.GeoCensus_d3 i.Current_measures Measures_clear MA_Pe
    > _SC2 NOO_SC3 NNOO_SC3 OOL_SC12 PJE_SC4 Trust_Science_SC4 Trust_in_media Impulsivity_SC4 NegEmo_SC6 SN_SC7 CTC_SC7 OT
    > == 1
    note: Trust_Science_SC4 omitted because of collinearity
    note: Trust_Science_SC4 omitted because of collinearity
```

Source	SS	df	MS
Model	2557.73187	40	63.9432968
Residual	2342.79863	2,878	.81403705
Total	4900.5305	2,918	1.67941415

Number of obs	=	2,919
F(40, 2878)	=	78.55
Prob > F	=	0.0000
R-squared	=	0.5219
Adj R-squared	=	0.5153
Root MSE	=	.90224

DV_Compliance_SC7	Coef.	Std. Err.	t	P>|t|	[95% Conf.	Interval]
1.wave_d1	-.4737815	.1655356	-2.86	0.004	-.7983618	-.1492013
1.wave_d2	-.6918409	.1678638	-4.12	0.000	-1.020986	-.3626955
Age	.0034062	.0014386	2.37	0.018	.0005855	.0062269
1.Gender_Female	.1175005	.0353331	3.33	0.001	.0482198	.1867812
1.Minority	-.0092273	.0377567	-0.24	0.807	-.0832602	.0648055
Education	.0342652	.0122589	2.80	0.005	.010228	.0583024
1.Employed	.0122939	.0391673	0.31	0.754	-.0645049	.0890927
1.Corona_care	-.0484301	.0652396	-0.74	0.458	-.1763511	.079491
1.Insurance_Public	.0949552	.0536987	1.77	0.077	-.0103365	.200247
1.Insurance_Private	.1359978	.0561314	2.42	0.015	.025936	.2460595
SES_before	.0058918	.0095442	0.62	0.537	-.0128224	.024606
SES_change	.0011368	.0112251	0.10	0.919	-.0208733	.023147
2.Health_self	.032454	.0410481	0.79	0.429	-.0480327	.1129406
2.Health_other	-.0190819	.0390369	-0.49	0.625	-.095625	.0574612
1.Conservative_01	.0497943	.0402832	1.24	0.217	-.0291926	.1287811
1.Conservative_other	.1068847	.0577958	1.85	0.065	-.0064406	.22021
1.GeoCensus_d1	-.0968137	.0529986	-1.83	0.068	-.2007329	.0071054
1.GeoCensus_d2	-.1260077	.0459874	-2.74	0.006	-.2161791	-.0358362
1.GeoCensus_d3	.0002152	.0564473	0.00	0.997	-.1104659	.1108964
Current_measures						
Yes	.1798028	.0522636	3.44	0.001	.077325	.2822806
Measures_clear	-.0204267	.0115044	-1.78	0.076	-.0429845	.0021311
MA_Perc_Threat_SC3	.1259061	.0171653	7.33	0.000	.0922486	.1595635
Costs_SC5	.0267351	.012241	2.18	0.029	.002733	.0507372
Deterr_SD_Likely_SC2	.0060321	.0120403	0.50	0.616	-.0175764	.0296406
Deterr_SD_Severe	-.0035961	.0106111	-0.34	0.735	-.0244022	.01721
MA_MoralBelief	.2450733	.0194444	12.60	0.000	.206947	.2831996
MA_Authority_SC2	-.0079703	.0112555	-0.71	0.479	-.0300399	.0140992
NOO_SC3	.0154857	.0246327	0.63	0.530	-.0328138	.0637852
NNOO_SC3	.0213354	.0210669	1.01	0.311	-.0199725	.0626432
OOL_SC12	.0165013	.0141896	1.16	0.245	-.0113215	.0443242
PJE_SC4	-.0094732	.0121385	-0.78	0.435	-.0332741	.0143278
Trust_Science_SC4	-.0413865	.0325109	-1.27	0.203	-.1051334	.0223605
Trust_in_media	-.0110316	.0157236	-0.70	0.483	-.0418622	.019799
Impulsivity_SC4	-.0857353	.0190182	-4.51	0.000	-.1230261	-.0484446
NegEmo_SC6	.0164741	.0126238	1.31	0.192	-.0082785	.0412267
SN_SC7	.0271199	.0145834	1.86	0.063	-.001475	.0557149

```
               CTC_SC7 |    .515832   .0216313    23.85   0.000     .4734175    .5582464
               OTC_SC7 |  -.0261077   .0101894    -2.56   0.010    -.0460869   -.0061284
     Trust_Science_SC4 |          0  (omitted)
                       |
wave_d1#c.Trust_Science_SC4 |
                     1 |   .0997106    .041488     2.40   0.016     .0183615    .1810598
                       |
     Trust_Science_SC4 |          0  (omitted)
                       |
wave_d2#c.Trust_Science_SC4 |
                     1 |   .1422876   .0420539     3.38   0.001     .0598287    .2247464
                       |
                 _cons |   .2307195   .2213733     1.04   0.297    -.2033467    .6647857
------------------------------------------------------------------------------------------

. 
. *20.a.3 Check hettest: Run this right after your regression to apply the Breusch-Pagan / Cook-Weisberg test for hete
. *if significant, then you need to run the regression with vce(ro) at the end
. estat hettest

Breusch-Pagan / Cook-Weisberg test for heteroskedasticity
         Ho: Constant variance
         Variables: fitted values of DV_Compliance_SC7

         chi2(1)      =   382.00
         Prob > chi2  =   0.0000

. 
. *20.a.4. check vif, to check for for multicollinearity (VIFs >10 are problematic)
. vif

    Variable |       VIF       1/VIF  
-------------+----------------------
   1.wave_d1 |     21.98    0.045497
   1.wave_d2 |     21.82    0.045826
         Age |      1.26    0.791022
1.Gender_F~e |      1.11    0.900951
  1.Minority |      1.15    0.868176
   Education |      1.28    0.779838
  1.Employed |      1.27    0.787974
1.Corona_c~e |      1.22    0.818926
1.Insuranc~c |      2.54    0.394141
1.Insuran~te |      2.34    0.426483
  SES_before |      1.37    0.728703
  SES_change |      1.24    0.808876
2.Health_s~f |      1.35    0.738094
2.Health_o~r |      1.33    0.753150
1.Conserv~01 |      1.44    0.693758
1.Conserva~r |      1.26    0.791141
1.GeoCensu~1 |      1.66    0.603532
1.GeoCensu~2 |      1.86    0.538670
1.GeoCensu~3 |      1.54    0.650134
1.Current_~s |      1.14    0.878191
Measures_c~r |      1.42    0.706148
MA_Perc_Th~3 |      2.40    0.417120
   Costs_SC5 |      1.44    0.692295
Deterr_SD_~2 |      1.61    0.619250
Deterr_SD_~e |      1.19    0.839135
MA_MoralBe~f |      2.28    0.439517
MA_Authori~2 |      1.62    0.616015
     NOO_SC3 |      1.77    0.566113
    NNOO_SC3 |      1.58    0.631193
    OOL_SC12 |      1.59    0.630269
     PJE_SC4 |      1.38    0.727165
Trust_Scie~4 |      3.69    0.271152
Trust_in_m~a |      1.52    0.655958
Impulsivi~C4 |      1.64    0.610405
  NegEmo_SC6 |      1.41    0.711702
      SN_SC7 |      1.49    0.673070
     CTC_SC7 |      1.64    0.608561
     OTC_SC7 |      1.14    0.874433
    wave_d1# |
          c. |
Trust_Scie~4 |
```

1	22.35	0.044746
wave_d2# c. Trust_Scie~4 1	22.07	0.045310
Mean VIF	3.63	

518 .
519 . *20.a.5. Effect size
520 . estat esize

Effect sizes for linear models

Source	Eta-Squared	df	[95% Conf.	Interval]
Model	.5219296	40	.4927885	.5366846
wave_d1	.0028382	1	.0002806	.0080129
wave_d2	.0058675	1	.0016147	.0126891
Age	.0019442	1	.0000415	.0064634
Gender_Female	.0038279	1	.000645	.0096126
Minority	.0000208	1	.	.0014458
Education	.0027073	1	.0002398	.0077934
Employed	.0000342	1	.	.0016386
Corona_care	.0001914	1	.	.00252
Insurance_Public	.0010853	1	.	.0048042
Insurance_Private	.0020355	1	.0000618	.006628
SES_before	.0001324	1	.	.0022791
SES_change	3.56e-06	1	.	.0008156
Health_self	.0002172	1	.	.0026137
Health_other	.000083	1	.	.0020268
Conservative_01	.0005306	1	.	.0035344
Conservative_other	.0011869	1	.	.0050143
GeoCensus_d1	.0011581	1	.	.0049552
GeoCensus_d2	.0026019	1	.0002084	.0076151
GeoCensus_d3	5.05e-09	1	.	.
Current_measures	.0040956	1	.000758	.0100308
Measures_clear	.0010942	1	.	.0048228
MA_Perc_Threat_SC3	.0183509	1	.009896	.0291764
Costs_SC5	.0016547	1	.	.0059292
Deterr_SD_Likely_SC2	.0000872	1	.	.0020513
Deterr_SD_Severe	.0000399	1	.	.0017004
MA_MoralBelief	.0523094	1	.0376576	.0688217
MA_Authority_SC2	.0001742	1	.	.0024539
NOO_SC3	.0001373	1	.	.002301
NNOO_SC3	.0003562	1	.	.003058
OOL_SC12	.0004697	1	.	.0033748
PJE_SC4	.0002116	1	.	.0025938
Trust_Science_SC4	.0034587	1	.0004985	.0090267
Trust_in_media	.000171	1	.	.0024414
Impulsivity_SC4	.0070118	1	.0022426	.0143315
NegEmo_SC6	.0005914	1	.	.003688
SN_SC7	.0012002	1	.	.0050414
CTC_SC7	.1649879	1	.1415312	.1889071
OTC_SC7	.0022759	1	.0001195	.0070535
Trust_Science_SC4	.0034587	1	.0004985	.0090267
wave_d1#c.Trust_Science_SC4	.002003	1	.0000545	.0065695
Trust_Science_SC4	.0034587	1	.0004985	.0090267
wave_d2#c.Trust_Science_SC4	.0039619	1	.0007009	.0098226

Note: Eta-Squared values for individual model terms are partial.

```
521 .
522 . *20.a.6 Regression with vce(ro)
523 . reg DV_Compliance_SC7 i.wave_d1 i.wave_d2 Age i.Gender_Female i.Minority Education i.Employed i.Corona_care i.Insura
    > rvative_01 i.Conservative_other i.GeoCensus_d1 i.GeoCensus_d2 i.GeoCensus_d3 i.Current_measures Measures_clear MA_Pe
    > _SC2 NOO_SC3 NNOO_SC3 OOL_SC12 PJE_SC4 Trust_Science_SC4 Trust_in_media Impulsivity_SC4 NegEmo_SC6 SN_SC7 CTC_SC7 OT
    > == 1, vce(ro)
    note: Trust_Science_SC4 omitted because of collinearity
    note: Trust_Science_SC4 omitted because of collinearity

    Linear regression                               Number of obs     =      2,919
                                                    F(40, 2878)       =      67.56
                                                    Prob > F          =     0.0000
                                                    R-squared         =     0.5219
                                                    Root MSE          =     .90224
```

DV_Compliance_SC7	Coef.	Robust Std. Err.	t	P>|t|	[95% Conf.	Interval]
1.wave_d1	-.4737815	.184317	-2.57	0.010	-.8351882	-.1123749
1.wave_d2	-.6918409	.182044	-3.80	0.000	-1.048791	-.3348911
Age	.0034062	.0014317	2.38	0.017	.000599	.0062134
1.Gender_Female	.1175005	.0354095	3.32	0.001	.0480701	.186931
1.Minority	-.0092273	.0389392	-0.24	0.813	-.0855789	.0671242
Education	.0342652	.0120949	2.83	0.005	.0105497	.0579807
1.Employed	.0122939	.0403632	0.30	0.761	-.0668499	.0914376
1.Corona_care	-.0484301	.0702614	-0.69	0.491	-.1861979	.0893378
1.Insurance_Public	.0949552	.0599473	1.58	0.113	-.0225888	.2124992
1.Insurance_Private	.1359978	.0623697	2.18	0.029	.0137039	.2582917
SES_before	.0058918	.010345	0.57	0.569	-.0143925	.0261761
SES_change	.0011368	.0123931	0.09	0.927	-.0231633	.025437
2.Health_self	.032454	.0368426	0.88	0.378	-.0397866	.1046946
2.Health_other	-.0190819	.0375576	-0.51	0.611	-.0927244	.0545606
1.Conservative_01	.0497943	.0407764	1.22	0.222	-.0301595	.1297481
1.Conservative_other	.1068847	.0614113	1.74	0.082	-.0135299	.2272993
1.GeoCensus_d1	-.0968137	.0517969	-1.87	0.062	-.1983765	.004749
1.GeoCensus_d2	-.1260077	.0441308	-2.86	0.004	-.2125388	-.0394765
1.GeoCensus_d3	.0002152	.0514919	0.00	0.997	-.1007495	.10118
Current_measures						
Yes	.1798028	.0636663	2.82	0.005	.0549667	.3046389
Measures_clear	-.0204267	.0122241	-1.67	0.095	-.0443956	.0035422
MA_Perc_Threat_SC3	.1259061	.0197157	6.39	0.000	.0872477	.1645645
Costs_SC5	.0267351	.0126161	2.12	0.034	.0019976	.0514726
Deterr_SD_Likely_SC2	.0060321	.0122256	0.49	0.622	-.0179398	.0300039
Deterr_SD_Severe	-.0035961	.0110827	-0.32	0.746	-.0253269	.0181348
MA_MoralBelief	.2450733	.0258061	9.50	0.000	.1944731	.2956735
MA_Authority_SC2	-.0079703	.0109081	-0.73	0.465	-.0293588	.0134181
NOO_SC3	.0154857	.0256981	0.60	0.547	-.0349028	.0658742
NNOO_SC3	.0213354	.0213792	1.00	0.318	-.0205847	.0632554
OOL_SC12	.0165013	.014691	1.12	0.261	-.0123047	.0453073
PJE_SC4	-.0094732	.0120007	-0.79	0.430	-.033004	.0140577
Trust_Science_SC4	-.0413865	.0309003	-1.34	0.181	-.1019753	.0192024
Trust_in_media	-.0110316	.0150414	-0.73	0.463	-.0405246	.0184614
Impulsivity_SC4	-.0857353	.0187155	-4.58	0.000	-.1224325	-.0490381
NegEmo_SC6	.0164741	.0129213	1.27	0.202	-.0088618	.04181
SN_SC7	.0271199	.0146482	1.85	0.064	-.0016022	.055842
CTC_SC7	.515832	.0318152	16.21	0.000	.453449	.5782149
OTC_SC7	-.0261077	.0097431	-2.68	0.007	-.0452119	-.0070035
Trust_Science_SC4	0	(omitted)				
wave_d1#c.Trust_Science_SC4						
1	.0997106	.0450264	2.21	0.027	.0114233	.1879979
Trust_Science_SC4	0	(omitted)				
wave_d2#c.Trust_Science_SC4						
1	.1422876	.0444969	3.20	0.001	.0550386	.2295365
_cons	.2307195	.2310363	1.00	0.318	-.2222938	.6837328

```
524 .
525 . *20.a.7 Simple main effects
526 . reg DV_Compliance_SC7 i.WAVE Age i.Gender_Female i.Minority Education i.Employed i.Corona_care i.Insurance_Public i.
    > Conservative_other i.GeoCensus_d1 i.GeoCensus_d2 i.GeoCensus_d3 i.Current_measures Measures_clear MA_Perc_Threat_SC3
    > NNOO_SC3 OOL_SC12 PJE_SC4 Trust_Science_SC4 Trust_in_media Impulsivity_SC4 NegEmo_SC6 SN_SC7 CTC_SC7 OTC_SC7 c.Trust
note: Trust_Science_SC4 omitted because of collinearity

Linear regression                               Number of obs     =      2,919
                                                F(40, 2878)       =      67.56
                                                Prob > F          =     0.0000
                                                R-squared         =     0.5219
                                                Root MSE          =     .90224
```

DV_Compliance_SC7	Coef.	Robust Std. Err.	t	P>|t|	[95% Conf.	Interval]
WAVE						
2	-.4737815	.184317	-2.57	0.010	-.8351882	-.1123749
3	-.6918409	.182044	-3.80	0.000	-1.048791	-.3348911
Age	.0034062	.0014317	2.38	0.017	.000599	.0062134
1.Gender_Female	.1175005	.0354095	3.32	0.001	.0480701	.186931
1.Minority	-.0092273	.0389392	-0.24	0.813	-.0855789	.0671242
Education	.0342652	.0120949	2.83	0.005	.0105497	.0579807
1.Employed	.0122939	.0403632	0.30	0.761	-.0668499	.0914376
1.Corona_care	-.0484301	.0702614	-0.69	0.491	-.1861979	.0893378
1.Insurance_Public	.0949552	.0599473	1.58	0.113	-.0225888	.2124992
1.Insurance_Private	.1359978	.0623697	2.18	0.029	.0137039	.2582917
SES_before	.0058918	.010345	0.57	0.569	-.0143925	.0261761
SES_change	.0011368	.0123931	0.09	0.927	-.0231633	.025437
2.Health_self	.032454	.0368426	0.88	0.378	-.0397866	.1046946
2.Health_other	-.0190819	.0375576	-0.51	0.611	-.0927244	.0545606
1.Conservative_01	.0497943	.0407764	1.22	0.222	-.0301595	.1297481
1.Conservative_other	.1068847	.0614113	1.74	0.082	-.0135299	.2272993
1.GeoCensus_d1	-.0968137	.0517969	-1.87	0.062	-.1983765	.004749
1.GeoCensus_d2	-.1260077	.0441308	-2.86	0.004	-.2125388	-.0394765
1.GeoCensus_d3	.0002152	.0514919	0.00	0.997	-.1007495	.10118
Current_measures						
Yes	.1798028	.0636663	2.82	0.005	.0549667	.3046389
Measures_clear	-.0204267	.0122241	-1.67	0.095	-.0443956	.0035422
MA_Perc_Threat_SC3	.1259061	.0197157	6.39	0.000	.0872477	.1645645
Costs_SC5	.0267351	.0126161	2.12	0.034	.0019976	.0514726
Deterr_SD_Likely_SC2	.0060321	.0122256	0.49	0.622	-.0179398	.0300039
Deterr_SD_Severe	-.0035961	.0110827	-0.32	0.746	-.0253269	.0181348
MA_MoralBelief	.2450733	.0258061	9.50	0.000	.1944731	.2956735
MA_Authority_SC2	-.0079703	.0109081	-0.73	0.465	-.0293588	.0134181
NOO_SC3	.0154857	.0256981	0.60	0.547	-.0349028	.0658742
NNOO_SC3	.0213354	.0213792	1.00	0.318	-.0205847	.0632554
OOL_SC12	.0165013	.014691	1.12	0.261	-.0123047	.0453073
PJE_SC4	-.0094732	.0120007	-0.79	0.430	-.033004	.0140577
Trust_Science_SC4	-.0413865	.0309003	-1.34	0.181	-.1019753	.0192024
Trust_in_media	-.0110316	.0150414	-0.73	0.463	-.0405246	.0184614
Impulsivity_SC4	-.0857353	.0187155	-4.58	0.000	-.1224325	-.0490381
NegEmo_SC6	.0164741	.0129213	1.27	0.202	-.0088618	.04181
SN_SC7	.0271199	.0146482	1.85	0.064	-.0016022	.055842
CTC_SC7	.515832	.0318152	16.21	0.000	.453449	.5782149
OTC_SC7	-.0261077	.0097431	-2.68	0.007	-.0452119	-.0070035
Trust_Science_SC4	0	(omitted)				
WAVE#c.Trust_Science_SC4						
2	.0997106	.0450264	2.21	0.027	.0114233	.1879979
3	.1422876	.0444969	3.20	0.001	.0550386	.2295365
_cons	.2307195	.2310363	1.00	0.318	-.2222938	.6837328

```
527 .
528 . margins WAVE, dydx(Trust_Science_SC4)

    Average marginal effects                        Number of obs     =      2,919
    Model VCE    : Robust

    Expression   : Linear prediction, predict()
    dy/dx w.r.t. : Trust_Science_SC4
```

	dy/dx	Delta-method Std. Err.	t	P>|t|	[95% Conf.	Interval]
**Trust_Science_SC4**						
WAVE						
1	-.0413865	.0309003	-1.34	0.181	-.1019753	.0192024
2	.0583241	.0367714	1.59	0.113	-.0137768	.1304251
3	.1009011	.0380744	2.65	0.008	.0262454	.1755569

```
529 . margins WAVE, dydx(Trust_Science_SC4) pwcompare(effects)

    Pairwise comparisons of average marginal effects

    Model VCE    : Robust                           Number of obs     =      2,919

    Expression   : Linear prediction, predict()
    dy/dx w.r.t. : Trust_Science_SC4
```

	Contrast dy/dx	Delta-method Std. Err.	Unadjusted t	P>|t|	Unadjusted [95% Conf.	Interval]
**Trust_Science_SC4**						
WAVE						
2 vs 1	.0997106	.0450264	2.21	0.027	.0114233	.1879979
3 vs 1	.1422876	.0444969	3.20	0.001	.0550386	.2295365
3 vs 2	.042577	.048291	0.88	0.378	-.0521114	.1372654

```
530 .
531 . *****************
532 .
533 . *21. Model 21: Trust in media x Survey Wave
534 .
535 . *21.a.2 Regression
536 . reg DV_Compliance_SC7 i.wave_d1 i.wave_d2 Age i.Gender_Female i.Minority Education i.Employed i.Corona_care i.Insura
    > rvative_01 i.Conservative_other i.GeoCensus_d1 i.GeoCensus_d2 i.GeoCensus_d3 i.Current_measures Measures_clear MA_Pe
    > _SC2 NOO_SC3 NNOO_SC3 OOL_SC12 PJE_SC4 Trust_Science_SC4 Trust_in_media Impulsivity_SC4 NegEmo_SC6 SN_SC7 CTC_SC7 OT
    note: Trust_in_media omitted because of collinearity
    note: Trust_in_media omitted because of collinearity
```

Source	SS	df	MS			
				Number of obs	=	2,919
				F(40, 2878)	=	78.25
Model	2553.03992	40	63.825998	Prob > F	=	0.0000
Residual	2347.49058	2,878	.815667332	R-squared	=	0.5210
				Adj R-squared	=	0.5143
Total	4900.5305	2,918	1.67941415	Root MSE	=	.90314

DV_Compliance_SC7	Coef.	Std. Err.	t	P>|t|	[95% Conf.	Interval]
1.wave_d1	-.2678575	.1008345	-2.66	0.008	-.4655726	-.0701423
1.wave_d2	-.3540862	.0999806	-3.54	0.000	-.550127	-.1580454
Age	.0034937	.0014398	2.43	0.015	.0006707	.0063168
1.Gender_Female	.1173511	.0353711	3.32	0.001	.0479958	.1867064
1.Minority	-.0049115	.0377706	-0.13	0.897	-.0789717	.0691488
Education	.0343222	.0122811	2.79	0.005	.0102417	.0584028
1.Employed	.0152492	.039191	0.39	0.697	-.0615961	.0920944
1.Corona_care	-.0504238	.0652979	-0.77	0.440	-.1784591	.0776115
1.Insurance_Public	.0925798	.0537436	1.72	0.085	-.0128	.1979597
1.Insurance_Private	.1373108	.0562011	2.44	0.015	.0271122	.2475093
SES_before	.0051956	.0095494	0.54	0.586	-.0135289	.02392
SES_change	-.0002978	.0112352	-0.03	0.979	-.0223277	.0217321

2.Health_self	.0342198	.0410836	0.83	0.405	-.0463365	.1147761
2.Health_other	-.0195124	.0390488	-0.50	0.617	-.0960789	.0570541
1.Conservative_01	.0481533	.0403332	1.19	0.233	-.0309317	.1272382
1.Conservative_other	.106634	.0578494	1.84	0.065	-.0067965	.2200644
1.GeoCensus_d1	-.0980745	.0530472	-1.85	0.065	-.2020889	.0059399
1.GeoCensus_d2	-.1236893	.0460304	-2.69	0.007	-.2139451	-.0334335
1.GeoCensus_d3	.0031566	.0565133	0.06	0.955	-.1076541	.1139673
Current_measures						
Yes	.178237	.0523417	3.41	0.001	.075606	.280868
Measures_clear	-.0209473	.0115184	-1.82	0.069	-.0435324	.0016379
MA_Perc_Threat_SC3	.1264016	.0171799	7.36	0.000	.0927154	.1600877
Costs_SC5	.0261549	.0122575	2.13	0.033	.0021206	.0501892
Deterr_SD_Likely_SC2	.0056986	.0120564	0.47	0.636	-.0179415	.0293386
Deterr_SD_Severe	-.0038055	.0106235	-0.36	0.720	-.024636	.0170249
MA_MoralBelief	.2441076	.0194785	12.53	0.000	.2059143	.2823008
MA_Authority_SC2	-.0091985	.0112678	-0.82	0.414	-.0312923	.0128953
NOO_SC3	.018519	.0246594	0.75	0.453	-.0298329	.066871
NNOO_SC3	.0189386	.021089	0.90	0.369	-.0224126	.0602897
OOL_SC12	.0158105	.0141974	1.11	0.266	-.0120275	.0436485
PJE_SC4	-.0096977	.0121506	-0.80	0.425	-.0335225	.014127
Trust_Science_SC4	.0389943	.0220285	1.77	0.077	-.004199	.0821876
Trust_in_media	-.0565865	.0237282	-2.38	0.017	-.1031125	-.0100606
Impulsivity_SC4	-.0843843	.0190476	-4.43	0.000	-.1217327	-.0470359
NegEmo_SC6	.0175076	.0126324	1.39	0.166	-.0072618	.042277
SN_SC7	.0285585	.0145857	1.96	0.050	-.000041	.057158
CTC_SC7	.5168281	.0216334	23.89	0.000	.4744096	.5592467
OTC_SC7	-.0259826	.0102023	-2.55	0.011	-.0459871	-.0059781
Trust_in_media	0	(omitted)				
wave_d1#c.Trust_in_media						
1	.0619608	.0314065	1.97	0.049	.0003792	.1235424
Trust_in_media	0	(omitted)				
wave_d2#c.Trust_in_media						
1	.0733104	.0313684	2.34	0.020	.0118037	.1348172
_cons	.0419671	.2067913	0.20	0.839	-.3635069	.447441

```
537 .
538 . *21.a.3 Check hettest: Run this right after your regression to apply the Breusch-Pagan / Cook-Weisberg test for hete
539 . *if significant, then you need to run the regression with vce(ro) at the end
540 . estat hettest

    Breusch-Pagan / Cook-Weisberg test for heteroskedasticity
             Ho: Constant variance
             Variables: fitted values of DV_Compliance_SC7

             chi2(1)      =   371.69
             Prob > chi2  =   0.0000

541 .
542 . *21.a.4. check vif, to check for for multicollinearity (VIFs >10 are problematic)
543 . vif
```

Variable	VIF	1/VIF
1.wave_d1	8.14	0.122863
1.wave_d2	7.73	0.129438
Age	1.26	0.791280
1.Gender_F~e	1.11	0.900816
1.Minority	1.15	0.869272
Education	1.28	0.778587
1.Employed	1.27	0.788597
1.Corona_c~e	1.22	0.819102
1.Insuranc~c	2.54	0.394270
1.Insuran~te	2.35	0.426277
SES_before	1.37	0.729363
SES_change	1.24	0.809040
2.Health_s~f	1.35	0.738294
2.Health_o~r	1.33	0.754197
1.Conserv~01	1.44	0.693423

1.Conserva~r	1.26	0.791257
1.GeoCensu~1	1.66	0.603634
1.GeoCensu~2	1.86	0.538740
1.GeoCensu~3	1.54	0.649914
1.Current_~s	1.14	0.877325
Measures_c~r	1.42	0.705850
MA_Perc_Th~3	2.40	0.417243
Costs_SC5	1.45	0.691823
Deterr_SD_~2	1.62	0.618835
Deterr_SD_~e	1.19	0.838854
MA_MoralBe~f	2.28	0.438855
MA_Authori~2	1.62	0.615893
NOO_SC3	1.77	0.566017
NNOO_SC3	1.58	0.631132
OOL_SC12	1.59	0.630844
PJE_SC4	1.38	0.727167
Trust_Scie~4	1.69	0.591789
Trust_in_m~a	3.46	0.288616
Impulsivi~C4	1.64	0.609740
NegEmo_SC6	1.40	0.712159
SN_SC7	1.48	0.674203
CTC_SC7	1.64	0.609662
OTC_SC7	1.14	0.873974
wave_d1# c. Trust_in_m~a 1	8.81	0.113500
wave_d2# c. Trust_in_m~a 1	8.09	0.123624
Mean VIF	2.25	

544 .
545 . *21.a.5. Effect size
546 . estat esize

Effect sizes for linear models

Source	Eta-Squared	df	[95% Conf.	Interval]
Model	.5209722	40	.491788	.5357451
wave_d1	.0024459	1	.0001643	.0073483
wave_d2	.0043392	1	.0008651	.0104068
Age	.0020418	1	.0000632	.0066393
Gender_Female	.00381	1	.0006376	.0095845
Minority	5.88e-06	1	.	.0009909
Education	.0027065	1	.0002395	.0077921
Employed	.0000526	1	.	.0018165
Corona_care	.0002072	1	.	.0025778
Insurance_Public	.00103	1	.	.0046877
Insurance_Private	.0020698	1	.0000696	.0066893
SES_before	.0001028	1	.	.0021368
SES_change	2.44e-07	1	.	.
Health_self	.000241	1	.	.0026964
Health_other	.0000868	1	.	.0020487
Conservative_01	.000495	1	.	.0034419
Conservative_other	.0011792	1	.	.0049985
GeoCensus_d1	.0011863	1	.	.0050129
GeoCensus_d2	.0025026	1	.00018	.0074458
GeoCensus_d3	1.08e-06	1	.	.0004013
Current_measures	.0040129	1	.0007225	.0099022
Measures_clear	.0011478	1	.	.0049341
MA_Perc_Threat_SC3	.018462	1	.0099785	.0293144
Costs_SC5	.0015795	1	.	.005787
Deterr_SD_Likely_SC2	.0000776	1	.	.001994
Deterr_SD_Severe	.0000446	1	.	.0017463
MA_MoralBelief	.0517469	1	.0371718	.0681906
MA_Authority_SC2	.0002315	1	.	.0026639
NOO_SC3	.0001959	1	.	.0025367
NNOO_SC3	.0002801	1	.	.0028253

OOL_SC12	.0004307	1	.	.0032692
PJE_SC4	.0002213	1	.	.0026283
Trust_Science_SC4	.0010876	1	.	.004809
Trust_in_media	.0001266	1	.	.0022527
Impulsivity_SC4	.0067733	1	.0021079	.0139929
NegEmo_SC6	.000667	1	.	.0038725
SN_SC7	.0013303	1	.	.005303
CTC_SC7	.1654936	1	.1420155	.1894288
OTC_SC7	.0022485	1	.0001126	.0070056
Trust_in_media	.0001266	1	.	.0022527
wave_d1#c.Trust_in_media	.0013506	1	.	.0053432
Trust_in_media	.0001266	1	.	.0022527
wave_d2#c.Trust_in_media	.0018942	1	.0000308	.0063726

Note: Eta-Squared values for individual model terms are partial.

```
547 .
548 . *21.a.6 Regression with vce(ro)
549 . reg DV_Compliance_SC7 i.wave_d1 i.wave_d2 Age i.Gender_Female i.Minority Education i.Employed i.Corona_care i.Insura
    > rvative_01 i.Conservative_other i.GeoCensus_d1 i.GeoCensus_d2 i.GeoCensus_d3 i.Current_measures Measures_clear MA_Pe
    > _SC2 NOO_SC3 NNOO_SC3 OOL_SC12 PJE_SC4 Trust_Science_SC4 Trust_in_media Impulsivity_SC4 NegEmo_SC6 SN_SC7 CTC_SC7 OT
    > vce(ro)
note: Trust_in_media omitted because of collinearity
note: Trust_in_media omitted because of collinearity
```

Linear regression

Number of obs	=	2,919
F(40, 2878)	=	67.85
Prob > F	=	0.0000
R-squared	=	0.5210
Root MSE	=	.90314

DV_Compliance_SC7	Coef.	Robust Std. Err.	t	P>|t|	[95% Conf.	Interval]
1.wave_d1	-.2678575	.1000881	-2.68	0.007	-.4641092	-.0716058
1.wave_d2	-.3540862	.0989617	-3.58	0.000	-.5481291	-.1600433
Age	.0034937	.0014373	2.43	0.015	.0006754	.0063121
1.Gender_Female	.1173511	.0353677	3.32	0.001	.0480026	.1866996
1.Minority	-.0049115	.0388643	-0.13	0.899	-.0811161	.0712931
Education	.0343222	.0120941	2.84	0.005	.0106083	.0580362
1.Employed	.0152492	.0403803	0.38	0.706	-.0639281	.0944265
1.Corona_care	-.0504238	.0702704	-0.72	0.473	-.1882092	.0873615
1.Insurance_Public	.0925798	.0600018	1.54	0.123	-.025071	.2102306
1.Insurance_Private	.1373108	.0624958	2.20	0.028	.0147697	.2598518
SES_before	.0051956	.0103582	0.50	0.616	-.0151147	.0255058
SES_change	-.0002978	.0123957	-0.02	0.981	-.0246032	.0240076
2.Health_self	.0342198	.0369934	0.93	0.355	-.0383164	.1067559
2.Health_other	-.0195124	.0377406	-0.52	0.605	-.0935137	.0544889
1.Conservative_01	.0481533	.0407507	1.18	0.237	-.0317502	.1280567
1.Conservative_other	.106634	.061576	1.73	0.083	-.0141036	.2273716
1.GeoCensus_d1	-.0980745	.0518345	-1.89	0.059	-.199711	.003562
1.GeoCensus_d2	-.1236893	.0440893	-2.81	0.005	-.2101391	-.0372396
1.GeoCensus_d3	.0031566	.0514918	0.06	0.951	-.097808	.1041212
Current_measures						
Yes	.178237	.0638701	2.79	0.005	.0530013	.3034727
Measures_clear	-.0209473	.0122403	-1.71	0.087	-.0449479	.0030533
MA_Perc_Threat_SC3	.1264016	.0197015	6.42	0.000	.087771	.1650321
Costs_SC5	.0261549	.0126387	2.07	0.039	.0013731	.0509367
Deterr_SD_Likely_SC2	.0056986	.0122622	0.46	0.642	-.018345	.0297421
Deterr_SD_Severe	-.0038055	.0111234	-0.34	0.732	-.0256161	.0180051
MA_MoralBelief	.2441076	.0258368	9.45	0.000	.1934471	.2947681
MA_Authority_SC2	-.0091985	.0108411	-0.85	0.396	-.0304557	.0120587
NOO_SC3	.018519	.0257038	0.72	0.471	-.0318806	.0689187
NNOO_SC3	.0189386	.0214827	0.88	0.378	-.0231846	.0610617
OOL_SC12	.0158105	.0146774	1.08	0.281	-.0129688	.0445898
PJE_SC4	-.0096977	.0120002	-0.81	0.419	-.0332276	.0138321
Trust_Science_SC4	.0389943	.0234192	1.67	0.096	-.0069259	.0849144
Trust_in_media	-.0565865	.0222702	-2.54	0.011	-.1002538	-.0129193
Impulsivity_SC4	-.0843843	.0186926	-4.51	0.000	-.1210365	-.0477321
NegEmo_SC6	.0175076	.0129122	1.36	0.175	-.0078105	.0428257
SN_SC7	.0285585	.0146284	1.95	0.051	-.0001246	.0572417
CTC_SC7	.5168281	.0319338	16.18	0.000	.4542127	.5794436

```
              OTC_SC7 │  -.0259826   .0097533    -2.66   0.008    -.0451067   -.0068585
       Trust_in_media │          0  (omitted)
                      │
wave_d1#c.Trust_in_media │
                    1 │   .0619608   .0314096     1.97   0.049     .0003732    .1235483
                      │
       Trust_in_media │          0  (omitted)
                      │
wave_d2#c.Trust_in_media │
                    1 │   .0733104   .0317626     2.31   0.021     .0110308    .1355901
                      │
                _cons │   .0419671   .2164123     0.19   0.846    -.3823716    .4663058
──────────────────────────────────────────────────────────────────────────────────────────
```

```
550 .
551 . *21.a.7 Simple main effects
552 . reg DV_Compliance_SC7 i.WAVE Age i.Gender_Female i.Minority Education i.Employed i.Corona_care i.Insurance_Public i.
    > Conservative_other i.GeoCensus_d1 i.GeoCensus_d2 i.GeoCensus_d3 i.Current_measures Measures_clear MA_Perc_Threat_SC3
    > NNOO_SC3 OOL_SC12 PJE_SC4 Trust_Science_SC4 Trust_in_media Impulsivity_SC4 NegEmo_SC6 SN_SC7 CTC_SC7 OTC_SC7 c.Trust
    note: Trust_in_media omitted because of collinearity

    Linear regression                               Number of obs     =      2,919
                                                    F(40, 2878)       =      67.85
                                                    Prob > F          =     0.0000
                                                    R-squared         =     0.5210
                                                    Root MSE          =     .90314
```

```
─────────────────────────────────────────────────────────────────────────────────────
                      │               Robust
    DV_Compliance_SC7 │      Coef.   Std. Err.      t    P>|t|     [95% Conf. Interval]
──────────────────────┼──────────────────────────────────────────────────────────────
                 WAVE │
                    2 │  -.2678575   .1000881    -2.68   0.007    -.4641092   -.0716058
                    3 │  -.3540862   .0989617    -3.58   0.000    -.5481291   -.1600433
                      │
                  Age │   .0034937   .0014373     2.43   0.015     .0006754    .0063121
      1.Gender_Female │   .1173511   .0353677     3.32   0.001     .0480026    .1866996
           1.Minority │  -.0049115   .0388643    -0.13   0.899    -.0811161    .0712931
            Education │   .0343222   .0120941     2.84   0.005     .0106083    .0580362
           1.Employed │   .0152492   .0403803     0.38   0.706    -.0639281    .0944265
        1.Corona_care │  -.0504238   .0702704    -0.72   0.473    -.1882092    .0873615
   1.Insurance_Public │   .0925798   .0600018     1.54   0.123     -.025071    .2102306
  1.Insurance_Private │   .1373108   .0624958     2.20   0.028     .0147697    .2598518
           SES_before │   .0051956   .0103582     0.50   0.616    -.0151147    .0255058
           SES_change │  -.0002978   .0123957    -0.02   0.981    -.0246032    .0240076
        2.Health_self │   .0342198   .0369934     0.93   0.355    -.0383164    .1067559
       2.Health_other │  -.0195124   .0377406    -0.52   0.605    -.0935137    .0544889
    1.Conservative_01 │   .0481533   .0407507     1.18   0.237    -.0317502    .1280567
 1.Conservative_other │    .106634    .061576     1.73   0.083    -.0141036    .2273716
       1.GeoCensus_d1 │  -.0980745   .0518345    -1.89   0.059     -.199711     .003562
       1.GeoCensus_d2 │  -.1236893   .0440893    -2.81   0.005    -.2101391   -.0372396
       1.GeoCensus_d3 │   .0031566   .0514918     0.06   0.951     -.097808    .1041212
                      │
     Current_measures │
                  Yes │    .178237   .0638701     2.79   0.005     .0530013    .3034727
       Measures_clear │  -.0209473   .0122403    -1.71   0.087    -.0449479    .0030533
    MA_Perc_Threat_SC3 │   .1264016   .0197015     6.42   0.000      .087771    .1650321
            Costs_SC5 │   .0261549   .0126387     2.07   0.039     .0013731    .0509367
  Deterr_SD_Likely_SC2 │   .0056986   .0122622     0.46   0.642     -.018345    .0297421
    Deterr_SD_Severe │  -.0038055   .0111234    -0.34   0.732    -.0256161    .0180051
      MA_MoralBelief │   .2441076   .0258368     9.45   0.000     .1934471    .2947681
     MA_Authority_SC2 │  -.0091985   .0108411    -0.85   0.396    -.0304557    .0120587
              NOO_SC3 │    .018519   .0257038     0.72   0.471    -.0318806    .0689187
             NNOO_SC3 │   .0189386   .0214827     0.88   0.378    -.0231846    .0610617
             OOL_SC12 │   .0158105   .0146774     1.08   0.281    -.0129688    .0445898
              PJE_SC4 │  -.0096977   .0120002    -0.81   0.419    -.0332276    .0138321
    Trust_Science_SC4 │   .0389943   .0234192     1.67   0.096    -.0069259    .0849144
       Trust_in_media │  -.0565865   .0222702    -2.54   0.011    -.1002538   -.0129193
      Impulsivity_SC4 │  -.0843843   .0186926    -4.51   0.000    -.1210365   -.0477321
           NegEmo_SC6 │   .0175076   .0129122     1.36   0.175    -.0078105    .0428257
               SN_SC7 │   .0285585   .0146284     1.95   0.051    -.0001246    .0572417
              CTC_SC7 │   .5168281   .0319338    16.18   0.000     .4542127    .5794436
              OTC_SC7 │  -.0259826   .0097533    -2.66   0.008    -.0451067   -.0068585
       Trust_in_media │          0  (omitted)
```

```
WAVE#c.Trust_in_media
                  2     .0619608   .0314096     1.97   0.049     .0003732    .1235483
                  3     .0733104   .0317626     2.31   0.021     .0110308    .1355901

              _cons     .0419671   .2164123     0.19   0.846    -.3823716    .4663058

553 .
554 . margins WAVE, dydx(Trust_in_media)

    Average marginal effects                        Number of obs     =      2,919
    Model VCE    : Robust

    Expression   : Linear prediction, predict()
    dy/dx w.r.t. : Trust_in_media

                              Delta-method
                        dy/dx   Std. Err.      t    P>|t|     [95% Conf. Interval]

    Trust_in_media
              WAVE
                 1   -.0565865   .0222702    -2.54   0.011    -.1002538   -.0129193
                 2    .0053742   .0239508     0.22   0.822    -.0415883    .0523367
                 3    .0167239   .0254208     0.66   0.511     -.033121    .0665688

555 . margins WAVE, dydx(Trust_in_media) pwcompare(effects)

    Pairwise comparisons of average marginal effects

    Model VCE    : Robust                           Number of obs     =      2,919

    Expression   : Linear prediction, predict()
    dy/dx w.r.t. : Trust_in_media

                     Contrast Delta-method    Unadjusted           Unadjusted
                        dy/dx   Std. Err.      t    P>|t|     [95% Conf. Interval]

    Trust_in_media
              WAVE
            2 vs 1    .0619608   .0314096     1.97   0.049     .0003732    .1235483
            3 vs 1    .0733104   .0317626     2.31   0.021     .0110308    .1355901
            3 vs 2    .0113497   .0331527     0.34   0.732    -.0536558    .0763552

556 .
557 . *****************
558 .
559 . *22. Model 22: Obligation to obey the law x Survey Wave
560 .
561 . *22.a.2 Regression
562 . reg DV_Compliance_SC7 i.wave_d1 i.wave_d2 Age i.Gender_Female i.Minority Education i.Employed i.Corona_care i.Insura
    > rvative_01 i.Conservative_other i.GeoCensus_d1 i.GeoCensus_d2 i.GeoCensus_d3 i.Current_measures Measures_clear MA_Pe
    > _SC2 NOO_SC3 NNOO_SC3 OOL_SC12 PJE_SC4 Trust_Science_SC4 Trust_in_media Impulsivity_SC4 NegEmo_SC6 SN_SC7 CTC_SC7 OT
    note: OOL_SC12 omitted because of collinearity
    note: OOL_SC12 omitted because of collinearity

          Source         SS           df       MS      Number of obs   =     2,919
                                                       F(40, 2878)     =     77.97
           Model   2548.72806          40  63.7182014   Prob > F        =    0.0000
        Residual   2351.80244       2,878  .817165546   R-squared       =    0.5201
                                                       Adj R-squared   =    0.5134
           Total    4900.5305       2,918  1.67941415   Root MSE        =    .90397
```

DV_Compliance_SC7	Coef.	Std. Err.	t	P>|t|	[95% Conf.	Interval]
1.wave_d1	.000731	.1261452	0.01	0.995	-.246613	.2480751
1.wave_d2	-.018933	.1300132	-0.15	0.884	-.2738615	.2359955
Age	.0034755	.0014414	2.41	0.016	.0006492	.0063017
1.Gender_Female	.1204174	.0353936	3.40	0.001	.051018	.1898167
1.Minority	-.0043943	.0378097	-0.12	0.907	-.0785312	.0697426
Education	.0346126	.0122894	2.82	0.005	.0105157	.0587094
1.Employed	.0145368	.0392287	0.37	0.711	-.0623825	.091456
1.Corona_care	-.0474544	.0653465	-0.73	0.468	-.175585	.0806762
1.Insurance_Public	.0904358	.0537916	1.68	0.093	-.0150382	.1959098
1.Insurance_Private	.1334448	.0562361	2.37	0.018	.0231776	.2437119
SES_before	.0052551	.0095596	0.55	0.583	-.0134892	.0239993
SES_change	.0005362	.0112505	0.05	0.962	-.0215235	.022596
2.Health_self	.0355943	.0411373	0.87	0.387	-.0450674	.1162559
2.Health_other	-.019319	.0390912	-0.49	0.621	-.0959686	.0573306
1.Conservative_01	.0506929	.0403786	1.26	0.209	-.028481	.1298669
1.Conservative_other	.1070714	.0579024	1.85	0.065	-.006463	.2206058
1.GeoCensus_d1	-.0969104	.0531349	-1.82	0.068	-.2010967	.0072759
1.GeoCensus_d2	-.1242771	.0460767	-2.70	0.007	-.2146237	-.0339305
1.GeoCensus_d3	.0026457	.0565782	0.05	0.963	-.1082922	.1135837
Current_measures						
Yes	.1790783	.0523365	3.42	0.001	.0764575	.2816992
Measures_clear	-.0194745	.0115356	-1.69	0.091	-.0420934	.0031443
MA_Perc_Threat_SC3	.125755	.0171912	7.32	0.000	.0920466	.1594634
Costs_SC5	.0262925	.012263	2.14	0.032	.0022475	.0503376
Deterr_SD_Likely_SC2	.00566	.0120632	0.47	0.639	-.0179934	.0293134
Deterr_SD_Severe	-.0035113	.0106316	-0.33	0.741	-.0243576	.017335
MA_MoralBelief	.2464393	.0194799	12.65	0.000	.2082433	.2846353
MA_Authority_SC2	-.0086972	.011296	-0.77	0.441	-.0308463	.0134519
NOO_SC3	.016753	.0246825	0.68	0.497	-.0316442	.0651502
NNOO_SC3	.0205173	.0211071	0.97	0.331	-.0208693	.0619039
OOL_SC12	.0311493	.0214056	1.46	0.146	-.0108225	.073121
PJE_SC4	-.0094774	.01217	-0.78	0.436	-.0333402	.0143854
Trust_Science_SC4	.037992	.0220469	1.72	0.085	-.0052373	.0812212
Trust_in_media	-.0123858	.0157469	-0.79	0.432	-.0432621	.0184906
Impulsivity_SC4	-.0856688	.0190569	-4.50	0.000	-.1230354	-.0483022
NegEmo_SC6	.0175586	.012636	1.39	0.165	-.0072179	.0423351
SN_SC7	.029495	.0145946	2.02	0.043	.0008782	.0581119
CTC_SC7	.5154816	.0216532	23.81	0.000	.4730242	.557939
OTC_SC7	-.02599	.0102123	-2.54	0.011	-.0460141	-.0059659
OOL_SC12	0	(omitted)				
wave_d1#c.OOL_SC12						
1	-.0199147	.0274602	-0.73	0.468	-.0737585	.033929
OOL_SC12	0	(omitted)				
wave_d2#c.OOL_SC12						
1	-.027988	.0280971	-1.00	0.319	-.0830804	.0271044
_cons	-.1680853	.2118504	-0.79	0.428	-.5834791	.2473085

```
. 
. *22.a.3 Check hettest: Run this right after your regression to apply the Breusch-Pagan / Cook-Weisberg test for hete
. *if significant, then you need to run the regression with vce(ro) at the end
. estat hettest

Breusch-Pagan / Cook-Weisberg test for heteroskedasticity
         Ho: Constant variance
         Variables: fitted values of DV_Compliance_SC7

         chi2(1)      =   366.85
         Prob > chi2  =   0.0000
```

```
567 .
568 . *22.a.4. check vif, to check for for multicollinearity (VIFs >10 are problematic)
569 . vif
```

Variable	VIF	1/VIF
1.wave_d1	12.71	0.078649
1.wave_d2	13.04	0.076686
Age	1.26	0.790951
1.Gender_F~e	1.11	0.901323
1.Minority	1.15	0.869068
Education	1.28	0.778961
1.Employed	1.27	0.788527
1.Corona_c~e	1.22	0.819386
1.Insuranc~c	2.54	0.394290
1.Insuran~te	2.34	0.426529
SES_before	1.37	0.729158
SES_change	1.24	0.808336
2.Health_s~f	1.36	0.737719
2.Health_o~r	1.33	0.753946
1.Conserv~01	1.44	0.693136
1.Conserva~r	1.26	0.791259
1.GeoCensu~1	1.66	0.602749
1.GeoCensu~2	1.86	0.538646
1.GeoCensu~3	1.54	0.649615
1.Current_~s	1.14	0.879110
Measures_c~r	1.42	0.705039
MA_Perc_Th~3	2.40	0.417459
Costs_SC5	1.44	0.692473
Deterr_SD_~2	1.61	0.619274
Deterr_SD_~e	1.19	0.839118
MA_MoralBe~f	2.27	0.439598
MA_Authori~2	1.63	0.613947
NOO_SC3	1.77	0.565996
NNOO_SC3	1.58	0.631209
OOL_SC12	3.60	0.278023
PJE_SC4	1.38	0.726181
Trust_Scie~4	1.69	0.591889
Trust_in_m~a	1.52	0.656531
Impulsivi~C4	1.64	0.610264
NegEmo_SC6	1.40	0.713058
SN_SC7	1.48	0.674622
CTC_SC7	1.64	0.609665
OTC_SC7	1.14	0.873864
wave_d1# c.OOL_SC12		
1	13.16	0.076011
wave_d2# c.OOL_SC12		
1	13.63	0.073345
Mean VIF	2.74	

```
570 .
571 . *22.a.5. Effect size
572 . estat esize
```

Effect sizes for linear models

Source	Eta-Squared	df	[95% Conf.	Interval]
Model	.5200923	40	.4908686	.5348817
wave_d1	1.17e-08	1	.	.
wave_d2	7.37e-06	1	.	.0010708
Age	.0020161	1	.0000574	.0065931
Gender_Female	.0040058	1	.0007195	.0098912
Minority	4.69e-06	1	.	.0009119
Education	.0027487	1	.0002525	.007863
Employed	.0000477	1	.	.0017747
Corona_care	.0001832	1	.	.0024888
Insurance_Public	.0009811	1	.	.0045833
Insurance_Private	.0019527	1	.0000434	.0064788

SES_before	.000105	1	.	.0021478
SES_change	7.89e-07	1	.	.0002911
Health_self	.0002601	1	.	.0027602
Health_other	.0000849	1	.	.0020377
Conservative_01	.0005473	1	.	.0035772
Conservative_other	.0011867	1	.	.0050139
GeoCensus_d1	.0011545	1	.	.0049477
GeoCensus_d2	.0025213	1	.0001852	.0074778
GeoCensus_d3	7.60e-07	1	.	.0002779
Current_measures	.0040516	1	.000739	.0099623
Measures_clear	.0009893	1	.	.0046008
MA_Perc_Threat_SC3	.0182535	1	.0098238	.0290552
Costs_SC5	.0015947	1	.	.0058159
Deterr_SD_Likely_SC2	.0000765	1	.	.0019869
Deterr_SD_Severe	.0000379	1	.	.0016795
MA_MoralBelief	.0526808	1	.0379787	.069238
MA_Authority_SC2	.0002059	1	.	.0025734
NOO_SC3	.00016	1	.	.0023974
NNOO_SC3	.0003282	1	.	.0029746
OOL_SC12	.0000675	1	.	.0019282
PJE_SC4	.0002107	1	.	.0025905
Trust_Science_SC4	.0010307	1	.	.0046892
Trust_in_media	.0002149	1	.	.0026057
Impulsivity_SC4	.0069728	1	.0022204	.0142763
NegEmo_SC6	.0006705	1	.	.0038809
SN_SC7	.0014171	1	.	.005474
CTC_SC7	.1645226	1	.1410856	.1884269
OTC_SC7	.0022454	1	.0001118	.0070001
OOL_SC12	.0000675	1	.	.0019282
wave_d1#c.OOL_SC12	.0001827	1	.	.0024869
OOL_SC12	.0000675	1	.	.0019282
wave_d2#c.OOL_SC12	.0003447	1	.	.0030238

Note: Eta-Squared values for individual model terms are partial.

```
573 .
574 . *22.a.6 Regression with vce(ro)
575 . reg DV_Compliance_SC7 i.wave_d1 i.wave_d2 Age i.Gender_Female i.Minority Education i.Employed i.Corona_care i.Insura
    > rvative_01 i.Conservative_other i.GeoCensus_d1 i.GeoCensus_d2 i.GeoCensus_d3 i.Current_measures Measures_clear MA_Pe
    > _SC2 NOO_SC3 NNOO_SC3 OOL_SC12 PJE_SC4 Trust_Science_SC4 Trust_in_media Impulsivity_SC4 NegEmo_SC6 SN_SC7 CTC_SC7 OT
note: OOL_SC12 omitted because of collinearity
note: OOL_SC12 omitted because of collinearity
```

Linear regression

Number of obs	=	2,919
F(40, 2878)	=	67.21
Prob > F	=	0.0000
R-squared	=	0.5201
Root MSE	=	.90397

DV_Compliance_SC7	Coef.	Robust Std. Err.	t	P>|t|	[95% Conf.	Interval]
1.wave_d1	.000731	.1349734	0.01	0.996	-.2639233	.2653854
1.wave_d2	-.018933	.1453026	-0.13	0.896	-.3038406	.2659746
Age	.0034755	.0014385	2.42	0.016	.0006548	.0062962
1.Gender_Female	.1204174	.035398	3.40	0.001	.0510094	.1898253
1.Minority	-.0043943	.038771	-0.11	0.910	-.080416	.0716274
Education	.0346126	.0120414	2.87	0.004	.0110019	.0582233
1.Employed	.0145368	.0403859	0.36	0.719	-.0646514	.093725
1.Corona_care	-.0474544	.0702595	-0.68	0.499	-.1852184	.0903096
1.Insurance_Public	.0904358	.0601413	1.50	0.133	-.0274885	.2083601
1.Insurance_Private	.1334448	.0626072	2.13	0.033	.0106853	.2562042
SES_before	.0052551	.0103843	0.51	0.613	-.0151063	.0256165
SES_change	.0005362	.0124955	0.04	0.966	-.0239648	.0250373
2.Health_self	.0355943	.0370287	0.96	0.337	-.0370111	.1081997
2.Health_other	-.019319	.0377314	-0.51	0.609	-.0933023	.0546643
1.Conservative_01	.0506929	.0408699	1.24	0.215	-.0294443	.1308302
1.Conservative_other	.1070714	.0615698	1.74	0.082	-.0136539	.2277967
1.GeoCensus_d1	-.0969104	.0520545	-1.86	0.063	-.1989782	.0051575
1.GeoCensus_d2	-.1242771	.0442264	-2.81	0.005	-.2109957	-.0375585
1.GeoCensus_d3	.0026457	.0514646	0.05	0.959	-.0982655	.1035569
Current_measures						

	Coef.	Std. Err.	t	P>|t|	[95% Conf.	Interval]
Yes	.1790783	.0636524	2.81	0.005	.0542695	.3038872
Measures_clear	-.0194745	.0120978	-1.61	0.108	-.0431958	.0042468
MA_Perc_Threat_SC3	.125755	.0197036	6.38	0.000	.0871205	.1643896
Costs_SC5	.0262925	.0126431	2.08	0.038	.001502	.0510831
Deterr_SD_Likely_SC2	.00566	.0122786	0.46	0.645	-.0184157	.0297357
Deterr_SD_Severe	-.0035113	.0111107	-0.32	0.752	-.0252971	.0182745
MA_MoralBelief	.2464393	.0259282	9.50	0.000	.1955996	.297279
MA_Authority_SC2	-.0086972	.0108479	-0.80	0.423	-.0299677	.0125733
NOO_SC3	.016753	.0257555	0.65	0.515	-.033748	.067254
NNOO_SC3	.0205173	.0215591	0.95	0.341	-.0217554	.06279
OOL_SC12	.0311493	.0224478	1.39	0.165	-.0128662	.0751647
PJE_SC4	-.0094774	.0120114	-0.79	0.430	-.0330293	.0140745
Trust_Science_SC4	.037992	.023513	1.62	0.106	-.0081121	.084096
Trust_in_media	-.0123858	.0150455	-0.82	0.410	-.0418868	.0171153
Impulsivity_SC4	-.0856688	.0187646	-4.57	0.000	-.1224621	-.0488754
NegEmo_SC6	.0175586	.0129002	1.36	0.174	-.007736	.0428532
SN_SC7	.029495	.0146908	2.01	0.045	.0006895	.0583006
CTC_SC7	.5154816	.0320183	16.10	0.000	.4527005	.5782628
OTC_SC7	-.02599	.009763	-2.66	0.008	-.0451331	-.0068469
OOL_SC12	0	(omitted)				
wave_d1#c.OOL_SC12						
1	-.0199147	.0282196	-0.71	0.480	-.0752474	.0354179
OOL_SC12	0	(omitted)				
wave_d2#c.OOL_SC12						
1	-.027988	.0304759	-0.92	0.359	-.0877449	.0317688
_cons	-.1680853	.2230682	-0.75	0.451	-.6054749	.2693042

```
576 .
577 .
578 . *****************
579 .
580 . *23. Model 23: Impulsivity x Survey Wave
581 .
582 . *23.a.2 Regression
583 . reg DV_Compliance_SC7 i.wave_d1 i.wave_d2 Age i.Gender_Female i.Minority Education i.Employed i.Corona_care i.Insura
    > rvative_01 i.Conservative_other i.GeoCensus_d1 i.GeoCensus_d2 i.GeoCensus_d3 i.Current_measures Measures_clear MA_Pe
    > _SC2 NOO_SC3 NNOO_SC3 OOL_SC12 PJE_SC4 Trust_Science_SC4 Trust_in_media Impulsivity_SC4 NegEmo_SC6 SN_SC7 CTC_SC7 OT
note: Impulsivity_SC4 omitted because of collinearity
note: Impulsivity_SC4 omitted because of collinearity
```

Source	SS	df	MS			
				Number of obs	=	2,919
				F(40, 2878)	=	78.18
Model	2551.87826	40	63.7969564	Prob > F	=	0.0000
Residual	2348.65224	2,878	.816070968	R-squared	=	0.5207
				Adj R-squared	=	0.5141
Total	4900.5305	2,918	1.67941415	Root MSE	=	.90337

DV_Compliance_SC7	Coef.	Std. Err.	t	P>|t|	[95% Conf.	Interval]
1.wave_d1	-.2493367	.0984993	-2.53	0.011	-.442473	-.0562004
1.wave_d2	-.3213424	.099921	-3.22	0.001	-.5172664	-.1254184
Age	.0034484	.0014402	2.39	0.017	.0006244	.0062723
1.Gender_Female	.1210843	.0353738	3.42	0.001	.0517236	.1904449
1.Minority	-.004012	.0377827	-0.11	0.915	-.078096	.070072
Education	.0340654	.0122864	2.77	0.006	.0099744	.0581563
1.Employed	.0152542	.0392009	0.39	0.697	-.0616104	.0921189
1.Corona_care	-.0481244	.0653035	-0.74	0.461	-.1761707	.079922
1.Insurance_Public	.0907293	.053792	1.69	0.092	-.0147454	.1962041
1.Insurance_Private	.1359536	.056223	2.42	0.016	.0257122	.246195
SES_before	.0050526	.0095551	0.53	0.597	-.013683	.0237882
SES_change	.0007932	.0112403	0.07	0.944	-.0212466	.022833
2.Health_self	.0351505	.0410926	0.86	0.392	-.0454233	.1157243
2.Health_other	-.0227079	.0390904	-0.58	0.561	-.0993559	.0539401
1.Conservative_01	.0501462	.0403374	1.24	0.214	-.028947	.1292394
1.Conservative_other	.1070946	.0578675	1.85	0.064	-.0063712	.2205605
1.GeoCensus_d1	-.0944785	.0530956	-1.78	0.075	-.1985877	.0096307
1.GeoCensus_d2	-.1221704	.0460563	-2.65	0.008	-.2124771	-.0318637
1.GeoCensus_d3	.0049626	.0565435	0.09	0.930	-.1059073	.1158326

```
         Current_measures |
                      Yes |   .1790847   .0523002     3.42   0.001      .076535    .2816343
           Measures_clear |  -.0192451     .01152    -1.67   0.095    -.0418333    .0033431
       MA_Perc_Threat_SC3 |   .1258567   .0171797     7.33   0.000     .0921708    .1595425
                Costs_SC5 |   .0258779    .012256     2.11   0.035     .0018465    .0499092
     Deterr_SD_Likely_SC2 |   .0051938   .0120571     0.43   0.667    -.0184477    .0288352
         Deterr_SD_Severe |  -.0035678   .0106225    -0.34   0.737    -.0243963    .0172607
           MA_MoralBelief |   .2464025   .0194664    12.66   0.000      .208233     .284572
         MA_Authority_SC2 |  -.0089638    .011287    -0.79   0.427    -.0310953    .0131676
                  NOO_SC3 |   .0186552   .0246668     0.76   0.450    -.0297112    .0670215
                 NNOO_SC3 |   .0199101   .0210856     0.94   0.345    -.0214344    .0612545
                 OOL_SC12 |   .0154067   .0141987     1.09   0.278    -.0124339    .0432473
                  PJE_SC4 |  -.0107593   .0121642    -0.88   0.376    -.0346107    .0130921
       Trust_Science_SC4 |    .037316   .0220335     1.69   0.090     -.005887     .080519
           Trust_in_media |  -.0118596   .0157369    -0.75   0.451    -.0427163     .018997
          Impulsivity_SC4 |  -.1320818   .0283882    -4.65   0.000    -.1877449   -.0764186
               NegEmo_SC6 |   .0189945   .0126424     1.50   0.133    -.0057947    .0437836
                   SN_SC7 |   .0293867   .0145964     2.01   0.044     .0007662    .0580072
                  CTC_SC7 |    .515759   .0216346    23.84   0.000     .4733381    .5581799
                  OTC_SC7 |  -.0255994   .0102091    -2.51   0.012    -.0456172   -.0055816
          Impulsivity_SC4 |          0  (omitted)
                          |
wave_d1#c.Impulsivity_SC4 |
                        1 |   .0668242   .0362858     1.84   0.066    -.0043245    .1379729
                          |
          Impulsivity_SC4 |          0  (omitted)
                          |
wave_d2#c.Impulsivity_SC4 |
                        1 |    .074012   .0371563     1.99   0.046     .0011564    .1468677
                          |
                    _cons |   .0115326    .205298     0.06   0.955    -.3910135    .4140787
------------------------------------------------------------------------------------------
```

```
584 .
585 . *23.a.3 Check hettest: Run this right after your regression to apply the Breusch-Pagan / Cook-Weisberg test for hete
586 . *if significant, then you need to run the regression with vce(ro) at the end
587 . estat hettest

Breusch-Pagan / Cook-Weisberg test for heteroskedasticity
         Ho: Constant variance
         Variables: fitted values of DV_Compliance_SC7

         chi2(1)      =   368.48
         Prob > chi2  =   0.0000

588 .
589 . *23.a.4. check vif, to check for for multicollinearity (VIFs >10 are problematic)
590 . vif
```

Variable	VIF	1/VIF
1.wave_d1	7.76	0.128821
1.wave_d2	7.71	0.129656
Age	1.26	0.791191
1.Gender_F~e	1.11	0.901122
1.Minority	1.15	0.869146
Education	1.28	0.778297
1.Employed	1.27	0.788589
1.Corona_c~e	1.22	0.819366
1.Insuranc~c	2.54	0.393756
1.Insuran~te	2.35	0.426156
SES_before	1.37	0.728853
SES_change	1.24	0.808717
2.Health_s~f	1.35	0.738338
2.Health_o~r	1.33	0.752967
1.Conserv~01	1.44	0.693622
1.Conserva~r	1.26	0.791154
1.GeoCensu~1	1.66	0.602833
1.GeoCensu~2	1.86	0.538400
1.GeoCensu~3	1.54	0.649541
1.Current_~s	1.14	0.879152
Measures_c~r	1.42	0.706005
MA_Perc_Th~3	2.40	0.417457

Costs_SC5	1.44	0.692334
Deterr_SD_~2	1.62	0.619067
Deterr_SD_~e	1.19	0.839425
MA_MoralBe~f	2.27	0.439618
MA_Authori~2	1.63	0.614106
NOO_SC3	1.77	0.565959
NNOO_SC3	1.58	0.631649
OOL_SC12	1.58	0.631040
PJE_SC4	1.38	0.725901
Trust_Scie~4	1.69	0.591816
Trust_in_m~a	1.52	0.656488
Impulsivi~C4	3.64	0.274643
NegEmo_SC6	1.41	0.711379
SN_SC7	1.48	0.673548
CTC_SC7	1.64	0.609895
OTC_SC7	1.15	0.873245
wave_d1# c. Impulsivi~C4 1	8.73	0.114486
wave_d2# c. Impulsivi~C4 1	8.43	0.118636
Mean VIF	2.25	

591 .
592 . *23.a.5. Effect size
593 . estat esize

Effect sizes for linear models

Source	Eta-Squared	df	[95% Conf.	Interval]
Model	.5207351	40	.4915403	.5355125
wave_d1	.0022215	1	.0001058	.0069581
wave_d2	.0035807	1	.0005456	.0092217
Age	.001988	1	.0000512	.0065426
Gender_Female	.0040547	1	.0007403	.0099672
Minority	3.92e-06	1	.	.0008487
Education	.002664	1	.0002267	.0077203
Employed	.0000526	1	.	.0018166
Corona_care	.0001887	1	.	.0025095
Insurance_Public	.0009875	1	.	.004597
Insurance_Private	.0020276	1	.00006	.0066138
SES_before	.0000971	1	.	.0021066
SES_change	1.73e-06	1	.	.0005638
Health_self	.0002542	1	.	.0027407
Health_other	.0001172	1	.	.0022087
Conservative_01	.0005367	1	.	.00355
Conservative_other	.0011887	1	.	.0050178
GeoCensus_d1	.001099	1	.	.0048327
GeoCensus_d2	.0024389	1	.0001624	.0073364
GeoCensus_d3	2.68e-06	1	.	.0007157
Current_measures	.0040575	1	.0007415	.0099715
Measures_clear	.0009688	1	.	.0045567
MA_Perc_Threat_SC3	.0183065	1	.009863	.0291211
Costs_SC5	.0015467	1	.	.0057244
Deterr_SD_Likely_SC2	.0000645	1	.	.0019069
Deterr_SD_Severe	.0000392	1	.	.0016931
MA_MoralBelief	.052735	1	.0380256	.0692988
MA_Authority_SC2	.0002191	1	.	.0026206
NOO_SC3	.0001987	1	.	.0025469
NNOO_SC3	.0003097	1	.	.0029182
OOL_SC12	.0004089	1	.	.0032088
PJE_SC4	.0002718	1	.	.0027984
Trust_Science_SC4	.0009956	1	.	.0046144
Trust_in_media	.0001973	1	.	.0025418
Impulsivity_SC4	.0027558	1	.0002547	.007875
NegEmo_SC6	.0007837	1	.	.0041459
SN_SC7	.0014064	1	.	.005453

```
                 CTC_SC7 │    .164907       1      .1414536    .1888235
                 OTC_SC7 │     .00218       1      .0000957    .0068849
         Impulsivity_SC4 │   .0027558       1      .0002547     .007875
wave_d1#c.Impulsivity_SC4 │    .001177       1             .    .0049941
         Impulsivity_SC4 │   .0027558       1      .0002547     .007875
wave_d2#c.Impulsivity_SC4 │   .0013767       1             .    .0053948
─────────────────────────────────────────────────────────────────────────

Note: Eta-Squared values for individual model terms are partial.

594 .
595 . *23.a.6 Regression with vce(ro)
596 . reg DV_Compliance_SC7 i.wave_d1 i.wave_d2 Age i.Gender_Female i.Minority Education i.Employed i.Corona_care i.Insura
    > rvative_01 i.Conservative_other i.GeoCensus_d1 i.GeoCensus_d2 i.GeoCensus_d3 i.Current_measures Measures_clear MA_Pe
    > _SC2 NOO_SC3 NNOO_SC3 OOL_SC12 PJE_SC4 Trust_Science_SC4 Trust_in_media Impulsivity_SC4 NegEmo_SC6 SN_SC7 CTC_SC7 OT
    > , vce(ro)
note: Impulsivity_SC4 omitted because of collinearity
note: Impulsivity_SC4 omitted because of collinearity

Linear regression                               Number of obs     =      2,919
                                                F(40, 2878)       =      67.60
                                                Prob > F          =     0.0000
                                                R-squared         =     0.5207
                                                Root MSE          =     .90337

────────────────────────────────────────────────────────────────────────────────────
                      │               Robust
    DV_Compliance_SC7 │      Coef.   Std. Err.      t    P>|t|     [95% Conf. Interval]
──────────────────────┼─────────────────────────────────────────────────────────────
            1.wave_d1 │  -.2493367   .0918657    -2.71   0.007    -.4294659   -.0692075
            1.wave_d2 │  -.3213424   .0966754    -3.32   0.001    -.5109024   -.1317824
                  Age │   .0034484   .0014397     2.40   0.017     .0006254    .0062714
      1.Gender_Female │   .1210843   .0354189     3.42   0.001     .0516354    .1905332
           1.Minority │   -.004012    .038782    -0.10   0.918    -.0800553    .0720313
            Education │   .0340654   .0120331     2.83   0.005     .0104709    .0576598
           1.Employed │   .0152542   .0404047     0.38   0.706    -.0639709    .0944793
        1.Corona_care │  -.0481244   .0700368    -0.69   0.492    -.1854517     .089203
   1.Insurance_Public │   .0907293   .0601796     1.51   0.132    -.0272701    .2087288
  1.Insurance_Private │   .1359536   .0626329     2.17   0.030     .0131437    .2587635
           SES_before │   .0050526   .0103187     0.49   0.624    -.0151801    .0252853
           SES_change │   .0007932   .0124354     0.06   0.949    -.0235901    .0251764
        2.Health_self │   .0351505   .0369446     0.95   0.341      -.03729    .1075911
       2.Health_other │  -.0227079   .0377613    -0.60   0.548    -.0967498     .051334
    1.Conservative_01 │   .0501462   .0407834     1.23   0.219    -.0298215    .1301138
 1.Conservative_other │   .1070946   .0615475     1.74   0.082    -.0135871    .2277763
       1.GeoCensus_d1 │  -.0944785   .0520244    -1.82   0.069    -.1964873    .0075303
       1.GeoCensus_d2 │  -.1221704   .0442694    -2.76   0.006    -.2089733   -.0353674
       1.GeoCensus_d3 │   .0049626   .0515025     0.10   0.923    -.0960229    .1059482
                      │
     Current_measures │
                 Yes  │   .1790847   .0635963     2.82   0.005     .0543858    .3037836
       Measures_clear │  -.0192451   .0121976    -1.58   0.115     -.043162    .0046718
     MA_Perc_Threat_SC3 │   .1258567   .0197189     6.38   0.000     .0871921    .1645212
            Costs_SC5 │   .0258779    .012605     2.05   0.040     .0011621    .0505936
  Deterr_SD_Likely_SC2 │   .0051938   .0122522     0.42   0.672    -.0188302    .0292177
     Deterr_SD_Severe │  -.0035678   .0111156    -0.32   0.748    -.0253632    .0182276
       MA_MoralBelief │   .2464025    .025942     9.50   0.000     .1955358    .2972692
     MA_Authority_SC2 │  -.0089638   .0108841    -0.82   0.410    -.0303052    .0123775
              NOO_SC3 │   .0186552   .0257073     0.73   0.468    -.0317513    .0690617
             NNOO_SC3 │   .0199101   .0215387     0.92   0.355    -.0223228     .062143
             OOL_SC12 │   .0154067   .0147199     1.05   0.295    -.0134559    .0442693
              PJE_SC4 │  -.0107593   .0120172    -0.90   0.371    -.0343226    .0128039
    Trust_Science_SC4 │    .037316   .0235138     1.59   0.113    -.0087897    .0834216
       Trust_in_media │  -.0118596   .0150637    -0.79   0.431    -.0413963     .017677
      Impulsivity_SC4 │  -.1320818   .0273067    -4.84   0.000    -.1856244   -.0785391
           NegEmo_SC6 │   .0189945   .0128422     1.48   0.139    -.0061863    .0441753
               SN_SC7 │   .0293867   .0147075     2.00   0.046     .0005484     .058225
              CTC_SC7 │    .515759   .0318749    16.18   0.000      .453259     .578259
              OTC_SC7 │  -.0255994   .0097591    -2.62   0.009     -.044735   -.0064638
      Impulsivity_SC4 │          0  (omitted)
                      │
wave_d1#c.Impulsivity_SC4 │
                    1 │   .0668242    .036712     1.82   0.069    -.0051602    .1388087
                      │
      Impulsivity_SC4 │          0  (omitted)
```

wave_d2#c.Impulsivity_SC4						
1	.074012	.0376206	1.97	0.049	.0002461	.147778
_cons	.0115326	.2178408	0.05	0.958	-.4156072	.4386724

```
597 .
598 . *21.a.7 Simple main effects
599 . reg DV_Compliance_SC7 i.WAVE Age i.Gender_Female i.Minority Education i.Employed i.Corona_care i.Insurance_Public i.
    > Conservative_other i.GeoCensus_d1 i.GeoCensus_d2 i.GeoCensus_d3 i.Current_measures Measures_clear MA_Perc_Threat_SC3
    > NNOO_SC3 OOL_SC12 PJE_SC4 Trust_Science_SC4 Trust_in_media Impulsivity_SC4 NegEmo_SC6 SN_SC7 CTC_SC7 OTC_SC7 c.Impul
    note: Impulsivity_SC4 omitted because of collinearity
```

Linear regression

Number of obs	=	2,919
F(40, 2878)	=	67.60
Prob > F	=	0.0000
R-squared	=	0.5207
Root MSE	=	.90337

DV_Compliance_SC7	Coef.	Robust Std. Err.	t	P>|t|	[95% Conf.	Interval]
WAVE						
2	-.2493367	.0918657	-2.71	0.007	-.4294659	-.0692075
3	-.3213424	.0966754	-3.32	0.001	-.5109024	-.1317824
Age	.0034484	.0014397	2.40	0.017	.0006254	.0062714
1.Gender_Female	.1210843	.0354189	3.42	0.001	.0516354	.1905332
1.Minority	-.004012	.038782	-0.10	0.918	-.0800553	.0720313
Education	.0340654	.0120331	2.83	0.005	.0104709	.0576598
1.Employed	.0152542	.0404047	0.38	0.706	-.0639709	.0944793
1.Corona_care	-.0481244	.0700368	-0.69	0.492	-.1854517	.089203
1.Insurance_Public	.0907293	.0601796	1.51	0.132	-.0272701	.2087288
1.Insurance_Private	.1359536	.0626329	2.17	0.030	.0131437	.2587635
SES_before	.0050526	.0103187	0.49	0.624	-.0151801	.0252853
SES_change	.0007932	.0124354	0.06	0.949	-.0235901	.0251764
2.Health_self	.0351505	.0369446	0.95	0.341	-.03729	.1075911
2.Health_other	-.0227079	.0377613	-0.60	0.548	-.0967498	.051334
1.Conservative_01	.0501462	.0407834	1.23	0.219	-.0298215	.1301138
1.Conservative_other	.1070946	.0615475	1.74	0.082	-.0135871	.2277763
1.GeoCensus_d1	-.0944785	.0520244	-1.82	0.069	-.1964873	.0075303
1.GeoCensus_d2	-.1221704	.0442694	-2.76	0.006	-.2089733	-.0353674
1.GeoCensus_d3	.0049626	.0515025	0.10	0.923	-.0960229	.1059482
Current_measures						
Yes	.1790847	.0635963	2.82	0.005	.0543858	.3037836
Measures_clear	-.0192451	.0121976	-1.58	0.115	-.043162	.0046718
MA_Perc_Threat_SC3	.1258567	.0197189	6.38	0.000	.0871921	.1645212
Costs_SC5	.0258779	.012605	2.05	0.040	.0011621	.0505936
Deterr_SD_Likely_SC2	.0051938	.0122522	0.42	0.672	-.0188302	.0292177
Deterr_SD_Severe	-.0035678	.0111156	-0.32	0.748	-.0253632	.0182276
MA_MoralBelief	.2464025	.025942	9.50	0.000	.1955358	.2972692
MA_Authority_SC2	-.0089638	.0108841	-0.82	0.410	-.0303052	.0123775
NOO_SC3	.0186552	.0257073	0.73	0.468	-.0317513	.0690617
NNOO_SC3	.0199101	.0215387	0.92	0.355	-.0223228	.062143
OOL_SC12	.0154067	.0147199	1.05	0.295	-.0134559	.0442693
PJE_SC4	-.0107593	.0120172	-0.90	0.371	-.0343226	.0128039
Trust_Science_SC4	.037316	.0235138	1.59	0.113	-.0087897	.0834216
Trust_in_media	-.0118596	.0150637	-0.79	0.431	-.0413963	.017677
Impulsivity_SC4	-.1320818	.0273067	-4.84	0.000	-.1856244	-.0785391
NegEmo_SC6	.0189945	.0128422	1.48	0.139	-.0061863	.0441753
SN_SC7	.0293867	.0147075	2.00	0.046	.0005484	.058225
CTC_SC7	.515759	.0318749	16.18	0.000	.453259	.578259
OTC_SC7	-.0255994	.0097591	-2.62	0.009	-.044735	-.0064638
Impulsivity_SC4	0	(omitted)				
WAVE#c.Impulsivity_SC4						
2	.0668242	.036712	1.82	0.069	-.0051602	.1388087
3	.074012	.0376206	1.97	0.049	.0002461	.147778
_cons	.0115326	.2178408	0.05	0.958	-.4156072	.4386724

```
600 .
601 . margins WAVE, dydx(Impulsivity_SC4)

    Average marginal effects                        Number of obs     =      2,919
    Model VCE    : Robust

    Expression   : Linear prediction, predict()
    dy/dx w.r.t. : Impulsivity_SC4
```

	dy/dx	Delta-method Std. Err.	t	P>|t|	[95% Conf.	Interval]
**Impulsivity_SC4**						
WAVE						
1	-.1320818	.0273067	-4.84	0.000	-.1856244	-.0785391
2	-.0652575	.0294572	-2.22	0.027	-.1230169	-.0074982
3	-.0580697	.029492	-1.97	0.049	-.1158974	-.0002421

```
602 . margins WAVE, dydx(Impulsivity_SC4) pwcompare(effects)

    Pairwise comparisons of average marginal effects

    Model VCE    : Robust                           Number of obs     =      2,919

    Expression   : Linear prediction, predict()
    dy/dx w.r.t. : Impulsivity_SC4
```

	Contrast dy/dx	Delta-method Std. Err.	Unadjusted t	Unadjusted P>|t|	Unadjusted [95% Conf.	Interval]
**Impulsivity_SC4**						
WAVE						
2 vs 1	.0668242	.036712	1.82	0.069	-.0051602	.1388087
3 vs 1	.074012	.0376206	1.97	0.049	.0002461	.147778
3 vs 2	.0071878	.0389258	0.18	0.854	-.0691375	.0835131

```
603 .
604 . *****************
605 .
606 . *24. Model 24: Neg emotions x Survey Wave
607 .
608 . *24.a.2 Regression
609 . reg DV_Compliance_SC7 i.wave_d1 i.wave_d2 Age i.Gender_Female i.Minority Education i.Employed i.Corona_care i.Insura
    > rvative_01 i.Conservative_other i.GeoCensus_d1 i.GeoCensus_d2 i.GeoCensus_d3 i.Current_measures Measures_clear MA_Pe
    > _SC2 NOO_SC3 NNOO_SC3 OOL_SC12 PJE_SC4 Trust_Science_SC4 Trust_in_media Impulsivity_SC4 NegEmo_SC6 SN_SC7 CTC_SC7 OT
    note: NegEmo_SC6 omitted because of collinearity
    note: NegEmo_SC6 omitted because of collinearity
```

Source	SS	df	MS
Model	2550.04959	40	63.7512398
Residual	2350.48091	2,878	.816706361
Total	4900.5305	2,918	1.67941415

Number of obs	=	2,919
F(40, 2878)	=	78.06
Prob > F	=	0.0000
R-squared	=	0.5204
Adj R-squared	=	0.5137
Root MSE	=	.90372

DV_Compliance_SC7	Coef.	Std. Err.	t	P>|t|	[95% Conf.	Interval]
1.wave_d1	-.2807761	.1257672	-2.23	0.026	-.527379	-.0341731
1.wave_d2	-.2417592	.1312625	-1.84	0.066	-.4991372	.0156188
Age	.0034879	.0014421	2.42	0.016	.0006602	.0063156
1.Gender_Female	.1199995	.0353772	3.39	0.001	.0506322	.1893668
1.Minority	-.0051115	.0377953	-0.14	0.892	-.0792202	.0689972
Education	.0352238	.0122835	2.87	0.004	.0111385	.0593092
1.Employed	.0118649	.0392312	0.30	0.762	-.0650592	.0887889
1.Corona_care	-.049496	.065353	-0.76	0.449	-.1776394	.0786473
1.Insurance_Public	.0915883	.0537939	1.70	0.089	-.0138902	.1970668
1.Insurance_Private	.1331409	.0562226	2.37	0.018	.0229003	.2433816
SES_before	.0055175	.0095629	0.58	0.564	-.0132333	.0242683

```
             SES_change |   .0005768   .0112594     0.05   0.959    -.0215005    .0226541
          2.Health_self |   .0337449   .0411102     0.82   0.412    -.0468635    .1143532
         2.Health_other |  -.0179965    .039079    -0.46   0.645    -.0946221    .0586291
     1.Conservative_01 |   .0491522   .0403535     1.22   0.223    -.0299725    .1282769
  1.Conservative_other |   .1072428   .0578859     1.85   0.064    -.0062592    .2207447
        1.GeoCensus_d1 |  -.0970849    .053098    -1.83   0.068    -.2011989    .0070291
        1.GeoCensus_d2 |  -.1238931   .0460623    -2.69   0.007    -.2142115   -.0335747
        1.GeoCensus_d3 |   .0017552   .0565507     0.03   0.975    -.1091288    .1126392
                       |
      Current_measures |
                   Yes |   .1806646   .0523302     3.45   0.001     .0780561    .2832731
        Measures_clear |  -.0193493   .0115269    -1.68   0.093    -.0419511    .0032525
     MA_Perc_Threat_SC3 |   .1269199   .0172117     7.37   0.000     .0931715    .1606684
             Costs_SC5 |   .0265708   .0122596     2.17   0.030     .0025324    .0506092
   Deterr_SD_Likely_SC2 |    .005195   .0120651     0.43   0.667    -.0184622    .0288522
      Deterr_SD_Severe |  -.0032235   .0106283    -0.30   0.762    -.0240632    .0176163
        MA_MoralBelief |   .2451576   .0195134    12.56   0.000     .2068959    .2834192
      MA_Authority_SC2 |  -.0082631   .0112807    -0.73   0.464    -.0303822     .013856
               NOO_SC3 |   .0163638   .0246735     0.66   0.507    -.0320157    .0647432
              NNOO_SC3 |   .0200303   .0210943     0.95   0.342    -.0213312    .0613919
              OOL_SC12 |   .0159722   .0142164     1.12   0.261    -.0119032    .0438476
               PJE_SC4 |  -.0097745    .012158    -0.80   0.421    -.0336138    .0140648
     Trust_Science_SC4 |   .0371883   .0220556     1.69   0.092    -.0060581    .0804348
        Trust_in_media |  -.0117104   .0157548    -0.74   0.457    -.0426023    .0191815
       Impulsivity_SC4 |  -.0837779   .0190862    -4.39   0.000     -.121202   -.0463538
            NegEmo_SC6 |  -.0043919   .0199187    -0.22   0.826    -.0434484    .0346645
                SN_SC7 |   .0292104   .0146222     2.00   0.046     .0005394    .0578815
               CTC_SC7 |    .516496   .0216465    23.86   0.000     .4740519    .5589402
               OTC_SC7 |  -.0264063   .0102064    -2.59   0.010     -.046419   -.0063937
            NegEmo_SC6 |          0  (omitted)
                       |
   wave_d1#c.NegEmo_SC6 |
                     1 |    .042564   .0259936     1.64   0.102     -.008404     .093532
                       |
            NegEmo_SC6 |          0  (omitted)
                       |
   wave_d2#c.NegEmo_SC6 |
                     1 |   .0217765   .0268753     0.81   0.418    -.0309202    .0744733
                       |
                 _cons |  -.0073503   .2103713    -0.03   0.972    -.4198439    .4051433
------------------------------------------------------------------------------------------
```

```
610 .
611 . *24.a.3 Check hettest: Run this right after your regression to apply the Breusch-Pagan / Cook-Weisberg test for hete
612 . *if significant, then you need to run the regression with vce(ro) at the end
613 . estat hettest

    Breusch-Pagan / Cook-Weisberg test for heteroskedasticity
             Ho: Constant variance
             Variables: fitted values of DV_Compliance_SC7

             chi2(1)      =   367.74
             Prob > chi2  =   0.0000

614 .
615 . *24.a.4. check vif, to check for for multicollinearity (VIFs >10 are problematic)
616 . vif
```

```
    Variable |       VIF       1/VIF
-------------+----------------------
   1.wave_d1 |     12.65    0.079078
   1.wave_d2 |     13.30    0.075191
         Age |      1.27    0.789700
1.Gender_F~e |      1.11    0.901651
  1.Minority |      1.15    0.869242
   Education |      1.28    0.779270
  1.Employed |      1.27    0.787985
1.Corona_c~e |      1.22    0.818763
1.Insuranc~c |      2.54    0.394034
1.Insuran~te |      2.34    0.426494
  SES_before |      1.37    0.728242
  SES_change |      1.24    0.806597
2.Health_s~f |      1.35    0.738279
2.Health_o~r |      1.33    0.753994
```

1.Conserv~01	1.44	0.693610
1.Conserva~r	1.26	0.791267
1.GeoCensu~1	1.66	0.603247
1.GeoCensu~2	1.86	0.538679
1.GeoCensu~3	1.54	0.649882
1.Current_~s	1.14	0.878827
Measures_c~r	1.42	0.705706
MA_Perc_Th~3	2.40	0.416233
Costs_SC5	1.44	0.692468
Deterr_SD_~2	1.62	0.618726
Deterr_SD_~e	1.19	0.839172
MA_MoralBe~f	2.28	0.437844
MA_Authori~2	1.63	0.615269
NOO_SC3	1.77	0.566093
NNOO_SC3	1.58	0.631619
OOL_SC12	1.59	0.629953
PJE_SC4	1.38	0.727207
Trust_Scie~4	1.69	0.591088
Trust_in_m~a	1.53	0.655502
Impulsivi~C4	1.64	0.608051
NegEmo_SC6	3.49	0.286798
SN_SC7	1.49	0.671695
CTC_SC7	1.64	0.609702
OTC_SC7	1.14	0.874376
wave_d1# c.NegEmo_SC6 1	13.17	0.075910
wave_d2# c.NegEmo_SC6 1	13.94	0.071756
Mean VIF	2.76	

617 .
618 . *24.a.5. Effect size
619 . estat esize

Effect sizes for linear models

Source	Eta-Squared	df	[95% Conf.	Interval]
Model	.5203619	40	.4911503	.5351464
wave_d1	.0017288	1	.	.0060679
wave_d2	.0011773	1	.	.0049946
Age	.0020284	1	.0000602	.0066153
Gender_Female	.0039819	1	.0007093	.0098538
Minority	6.36e-06	1	.	.0010185
Education	.0028491	1	.000284	.0080309
Employed	.0000318	1	.	.0016092
Corona_care	.0001993	1	.	.002549
Insurance_Public	.0010062	1	.	.004637
Insurance_Private	.0019448	1	.0000417	.0064644
SES_before	.0001157	1	.	.002201
SES_change	9.12e-07	1	.	.0003413
Health_self	.0002341	1	.	.0026727
Health_other	.0000737	1	.	.0019691
Conservative_01	.0005152	1	.	.0034947
Conservative_other	.0011912	1	.	.005023
GeoCensus_d1	.0011602	1	.	.0049596
GeoCensus_d2	.0025074	1	.0001813	.0074539
GeoCensus_d3	3.35e-07	1	.	.
Current_measures	.0041243	1	.0007704	.0100753
Measures_clear	.0009781	1	.	.0045768
MA_Perc_Threat_SC3	.0185436	1	.010039	.0294156
Costs_SC5	.0016295	1	.	.0058818
Deterr_SD_Likely_SC2	.0000644	1	.	.0019065
Deterr_SD_Severe	.000032	1	.	.0016114
MA_MoralBelief	.0519931	1	.0373843	.0684669
MA_Authority_SC2	.0001864	1	.	.0025009
NOO_SC3	.0001528	1	.	.0023675
NNOO_SC3	.0003132	1	.	.0029289
OOL_SC12	.0004384	1	.	.0032902

PJE_SC4	.0002245	1	.	.0026396
Trust_Science_SC4	.0009869	1	.	.0045956
Trust_in_media	.0001919	1	.	.0025218
Impulsivity_SC4	.0066501	1	.0020391	.0138174
NegEmo_SC6	.0012361	1	.	.0051142
SN_SC7	.0013847	1	.	.0054105
CTC_SC7	.1651495	1	.1416859	.1890738
OTC_SC7	.0023204	1	.0001309	.0071312
NegEmo_SC6	.0012361	1	.	.0051142
wave_d1#c.NegEmo_SC6	.0009308	1	.	.0044743
NegEmo_SC6	.0012361	1	.	.0051142
wave_d2#c.NegEmo_SC6	.0002281	1	.	.002652

Note: Eta-Squared values for individual model terms are partial.

```
620 .
621 . *24.a.6 Regression with vce(ro)
622 . reg DV_Compliance_SC7 i.wave_d1 i.wave_d2 Age i.Gender_Female i.Minority Education i.Employed i.Corona_care i.Insura
    > rvative_01 i.Conservative_other i.GeoCensus_d1 i.GeoCensus_d2 i.GeoCensus_d3 i.Current_measures Measures_clear MA_Pe
    > _SC2 NOO_SC3 NNOO_SC3 OOL_SC12 PJE_SC4 Trust_Science_SC4 Trust_in_media Impulsivity_SC4 NegEmo_SC6 SN_SC7 CTC_SC7 OT
    note: NegEmo_SC6 omitted because of collinearity
    note: NegEmo_SC6 omitted because of collinearity
```

Linear regression

Number of obs	=	2,919
F(40, 2878)	=	67.24
Prob > F	=	0.0000
R-squared	=	0.5204
Root MSE	=	.90372

DV_Compliance_SC7	Coef.	Robust Std. Err.	t	P>|t|	[95% Conf.	Interval]
1.wave_d1	-.2807761	.1287354	-2.18	0.029	-.533199	-.0283531
1.wave_d2	-.2417592	.1420104	-1.70	0.089	-.5202115	.0366931
Age	.0034879	.0014368	2.43	0.015	.0006707	.0063051
1.Gender_Female	.1199995	.0354319	3.39	0.001	.050525	.189474
1.Minority	-.0051115	.0388945	-0.13	0.895	-.0813755	.0711525
Education	.0352238	.0120625	2.92	0.004	.0115718	.0588758
1.Employed	.0118649	.0403	0.29	0.768	-.0671549	.0908847
1.Corona_care	-.049496	.0703621	-0.70	0.482	-.1874612	.0884691
1.Insurance_Public	.0915883	.0600888	1.52	0.128	-.0262331	.2094097
1.Insurance_Private	.1331409	.0625193	2.13	0.033	.0105537	.2557281
SES_before	.0055175	.0103929	0.53	0.596	-.0148607	.0258958
SES_change	.0005768	.012484	0.05	0.963	-.0239017	.0250554
2.Health_self	.0337449	.0369281	0.91	0.361	-.0386633	.106153
2.Health_other	-.0179965	.0377467	-0.48	0.634	-.0920099	.0560169
1.Conservative_01	.0491522	.0408203	1.20	0.229	-.0308878	.1291922
1.Conservative_other	.1072428	.0615915	1.74	0.082	-.0135253	.2280108
1.GeoCensus_d1	-.0970849	.0518497	-1.87	0.061	-.1987513	.0045815
1.GeoCensus_d2	-.1238931	.0442393	-2.80	0.005	-.2106369	-.0371492
1.GeoCensus_d3	.0017552	.0513025	0.03	0.973	-.0988381	.1023486
Current_measures						
Yes	.1806646	.064002	2.82	0.005	.0551703	.306159
Measures_clear	-.0193493	.0121812	-1.59	0.112	-.0432341	.0045355
MA_Perc_Threat_SC3	.1269199	.0197577	6.42	0.000	.0881792	.1656607
Costs_SC5	.0265708	.0126383	2.10	0.036	.0017898	.0513518
Deterr_SD_Likely_SC2	.005195	.0122616	0.42	0.672	-.0188475	.0292375
Deterr_SD_Severe	-.0032235	.0111337	-0.29	0.772	-.0250544	.0186074
MA_MoralBelief	.2451576	.0260356	9.42	0.000	.1941074	.2962078
MA_Authority_SC2	-.0082631	.0109262	-0.76	0.450	-.029687	.0131608
NOO_SC3	.0163638	.0257852	0.63	0.526	-.0341955	.066923
NNOO_SC3	.0200303	.0215783	0.93	0.353	-.0222801	.0623407
OOL_SC12	.0159722	.0147926	1.08	0.280	-.0130329	.0449773
PJE_SC4	-.0097745	.0120005	-0.81	0.415	-.0333049	.0137559
Trust_Science_SC4	.0371883	.0235817	1.58	0.115	-.0090504	.0834271
Trust_in_media	-.0117104	.0150682	-0.78	0.437	-.041256	.0178352
Impulsivity_SC4	-.0837779	.0188338	-4.45	0.000	-.1207071	-.0468487
NegEmo_SC6	-.0043919	.0196102	-0.22	0.823	-.0428434	.0340595
SN_SC7	.0292104	.0146365	2.00	0.046	.0005113	.0579096
CTC_SC7	.516496	.0319549	16.16	0.000	.4538393	.5791528
OTC_SC7	-.0264063	.0097641	-2.70	0.007	-.0455517	-.007261
NegEmo_SC6	0	(omitted)				

```
wave_d1#c.NegEmo_SC6
                   1 |    .042564   .0257904     1.65   0.099    -.0080055    .0931335
                     |
          NegEmo_SC6 |          0  (omitted)
                     |
wave_d2#c.NegEmo_SC6
                   1 |   .0217765   .0281588     0.77   0.439    -.0334369    .0769899
                     |
               _cons |  -.0073503   .2212357    -0.03   0.973    -.4411467    .4264461
------------------------------------------------------------------------------------

623 .
624 .
625 . *****************
626 .
627 . *25. Model 25: Social norms x Survey Wave
628 .
629 . *25.a.2 Regression
630 . reg DV_Compliance_SC7 i.wave_d1 i.wave_d2 Age i.Gender_Female i.Minority Education i.Employed i.Corona_care i.Insura
    > rvative_01 i.Conservative_other i.GeoCensus_d1 i.GeoCensus_d2 i.GeoCensus_d3 i.Current_measures Measures_clear MA_Pe
    > _SC2 NOO_SC3 NNOO_SC3 OOL_SC12 PJE_SC4 Trust_Science_SC4 Trust_in_media Impulsivity_SC4 NegEmo_SC6 SN_SC7 CTC_SC7 OT
note: SN_SC7 omitted because of collinearity
note: SN_SC7 omitted because of collinearity

      Source |       SS           df       MS      Number of obs   =     2,919
-------------+----------------------------------   F(40, 2878)     =     78.10
       Model |  2550.72046        40  63.7680114   Prob > F        =    0.0000
    Residual |  2349.81005     2,878  .816473261   R-squared       =    0.5205
-------------+----------------------------------   Adj R-squared   =    0.5138
       Total |   4900.5305     2,918  1.67941415   Root MSE        =    .90359

------------------------------------------------------------------------------------
   DV_Compliance_SC7 |      Coef.   Std. Err.      t    P>|t|     [95% Conf. Interval]
---------------------+--------------------------------------------------------------
           1.wave_d1 |  -.1861208   .1667137    -1.12   0.264    -.5130112    .1407696
           1.wave_d2 |  -.4297011   .1647561    -2.61   0.009    -.7527529   -.1066493
                 Age |   .0034079   .0014414     2.36   0.018     .0005817    .0062342
     1.Gender_Female |   .1191131   .0353736     3.37   0.001     .0497531    .1884732
          1.Minority |  -.0062236   .0378015    -0.16   0.869    -.0803444    .0678971
           Education |     .03476   .0122769     2.83   0.005     .0106875    .0588324
          1.Employed |   .0122547   .0392291     0.31   0.755    -.0646654    .0891747
       1.Corona_care |  -.0493676   .0653308    -0.76   0.450    -.1774676    .0787323
  1.Insurance_Public |   .0908149   .0537653     1.69   0.091    -.0146075    .1962373
 1.Insurance_Private |   .1335474   .0562124     2.38   0.018     .0233267    .2437681
          SES_before |   .0047498   .0095573     0.50   0.619    -.0139901    .0234897
          SES_change |  -7.57e-06   .0112366    -0.00   0.999    -.0220401     .022025
       2.Health_self |    .032449   .0411272     0.79   0.430    -.0481929    .1130908
      2.Health_other |  -.0174538   .0390783    -0.45   0.655     -.094078    .0591704
    1.Conservative_01 |   .0488903   .0403462     1.21   0.226    -.0302201    .1280007
1.Conservative_other |   .1036304   .0579096     1.79   0.074     -.009918    .2171788
      1.GeoCensus_d1 |  -.0980638   .0530776    -1.85   0.065    -.2021377    .0060102
      1.GeoCensus_d2 |  -.1282112   .0460692    -2.78   0.005     -.218543   -.0378793
      1.GeoCensus_d3 |  -.0056823   .0566375    -0.10   0.920    -.1167364    .1053719
                     |
    Current_measures |
                Yes  |   .1807226   .0523273     3.45   0.001     .0781198    .2833253
      Measures_clear |  -.0199137   .0115179    -1.73   0.084    -.0424979    .0026706
   MA_Perc_Threat_SC3 |   .1260397   .0171844     7.33   0.000     .0923448    .1597346
           Costs_SC5 |   .0266773   .0122599     2.18   0.030     .0026382    .0507163
Deterr_SD_Likely_SC2 |   .0057532   .0120577     0.48   0.633    -.0178894    .0293959
    Deterr_SD_Severe |  -.0041066   .0106602    -0.39   0.700    -.0250091    .0167959
      MA_MoralBelief |   .2464849   .0194673    12.66   0.000     .2083136    .2846562
    MA_Authority_SC2 |  -.0085187   .0112684    -0.76   0.450    -.0306137    .0135763
             NOO_SC3 |   .0179772   .0246711     0.73   0.466    -.0303977    .0663521
            NNOO_SC3 |   .0217215   .0211137     1.03   0.304     -.019678    .0631209
            OOL_SC12 |     .01584   .0142064     1.11   0.265    -.0120157    .0436957
             PJE_SC4 |  -.0099332   .0121573    -0.82   0.414    -.0337711    .0139047
   Trust_Science_SC4 |   .0357131   .0220669     1.62   0.106    -.0075553    .0789816
      Trust_in_media |  -.0125232   .0157401    -0.80   0.426    -.0433862    .0183399
     Impulsivity_SC4 |  -.0848468   .0190561    -4.45   0.000    -.1222117   -.0474819
          NegEmo_SC6 |   .0177601   .0126321     1.41   0.160    -.0070088     .042529
              SN_SC7 |   .0041627   .0235054     0.18   0.859    -.0419265    .0502518
             CTC_SC7 |   .5158264   .0216508    23.82   0.000     .4733739     .558279
```

OTC_SC7	-.0268449	.0102069	-2.63	0.009	-.0468585	-.0068314
SN_SC7	0	(omitted)				
wave_d1#c.SN_SC7						
1	.0180586	.0302373	0.60	0.550	-.0412303	.0773476
SN_SC7	0	(omitted)				
wave_d2#c.SN_SC7						
1	.0548762	.0301681	1.82	0.069	-.0042772	.1140295
_cons	.0500781	.2265679	0.22	0.825	-.3941737	.4943299

```
631 .
632 . *25.a.3 Check hettest: Run this right after your regression to apply the Breusch-Pagan / Cook-Weisberg test for hete
633 . *if significant, then you need to run the regression with vce(ro) at the end
634 . estat hettest

    Breusch-Pagan / Cook-Weisberg test for heteroskedasticity
             Ho: Constant variance
             Variables: fitted values of DV_Compliance_SC7

             chi2(1)      =   368.83
             Prob > chi2  =   0.0000

635 .
636 . *25.a.4. check vif, to check for for multicollinearity (VIFs >10 are problematic)
637 . vif
```

Variable	VIF	1/VIF
1.wave_d1	22.23	0.044991
1.wave_d2	20.96	0.047713
Age	1.27	0.790278
1.Gender_F~e	1.11	0.901581
1.Minority	1.15	0.868712
Education	1.28	0.779878
1.Employed	1.27	0.787842
1.Corona_c~e	1.22	0.819084
1.Insuranc~c	2.54	0.394341
1.Insuran~te	2.34	0.426526
SES_before	1.37	0.728880
SES_change	1.24	0.809646
2.Health_s~f	1.36	0.737456
2.Health_o~r	1.33	0.753806
1.Conserv~01	1.44	0.693663
1.Conserva~r	1.27	0.790394
1.GeoCensu~1	1.66	0.603538
1.GeoCensu~2	1.86	0.538365
1.GeoCensu~3	1.54	0.647707
1.Current_~s	1.14	0.878675
Measures_c~r	1.42	0.706601
MA_Perc_Th~3	2.40	0.417439
Costs_SC5	1.44	0.692232
Deterr_SD_~2	1.61	0.619309
Deterr_SD_~e	1.20	0.833904
MA_MoralBe~f	2.27	0.439794
MA_Authori~2	1.62	0.616435
NOO_SC3	1.77	0.566039
NNOO_SC3	1.59	0.630282
OOL_SC12	1.59	0.630666
PJE_SC4	1.38	0.727085
Trust_Scie~4	1.69	0.590318
Trust_in_m~a	1.52	0.656539
Impulsivi~C4	1.64	0.609804
NegEmo_SC6	1.40	0.712893
SN_SC7	3.85	0.259859
CTC_SC7	1.64	0.609286
OTC_SC7	1.14	0.874048
wave_d1#		
c.SN_SC7		
1	22.03	0.045383
wave_d2#		

c.SN_SC7		
1	20.32	0.049202
Mean VIF	3.58	

638 .
639 . *25.a.5. Effect size
640 . estat esize

Effect sizes for linear models

Source	Eta-Squared	df	[95% Conf.	Interval]
Model	.5204988	40	.4912934	.5352807
wave_d1	.0004329	1	.	.0032751
wave_d2	.0023579	1	.0001407	.0071964
Age	.0019386	1	.0000403	.0064533
Gender_Female	.0039243	1	.000685	.0097638
Minority	9.42e-06	1	.	.001158
Education	.0027777	1	.0002615	.0079116
Employed	.0000339	1	.	.0016348
Corona_care	.0001984	1	.	.0025457
Insurance_Public	.0009903	1	.	.0046031
Insurance_Private	.0019573	1	.0000444	.0064872
SES_before	.0000858	1	.	.0020433
SES_change	1.58e-10	1	.	.
Health_self	.0002163	1	.	.0026105
Health_other	.0000693	1	.	.0019403
Conservative_01	.00051	1	.	.003481
Conservative_other	.0011115	1	.	.0048588
GeoCensus_d1	.0011846	1	.	.0050096
GeoCensus_d2	.0026839	1	.0002327	.007754
GeoCensus_d3	3.50e-06	1	.	.000809
Current_measures	.0041274	1	.0007717	.0100802
Measures_clear	.0010376	1	.	.0047037
MA_Perc_Threat_SC3	.0183491	1	.0098947	.0291741
Costs_SC5	.0016425	1	.	.0059063
Deterr_SD_Likely_SC2	.0000791	1	.	.0020031
Deterr_SD_Severe	.0000516	1	.	.0018079
MA_MoralBelief	.0527638	1	.0380505	.0693311
MA_Authority_SC2	.0001985	1	.	.0025464
NOO_SC3	.0001845	1	.	.0024936
NNOO_SC3	.0003676	1	.	.0030912
OOL_SC12	.0004318	1	.	.0032721
PJE_SC4	.0002319	1	.	.0026653
Trust_Science_SC4	.0009093	1	.	.0044272
Trust_in_media	.0002199	1	.	.0026234
Impulsivity_SC4	.0068412	1	.0021461	.0140896
NegEmo_SC6	.0006864	1	.	.0039189
SN_SC7	.0020659	1	.0000687	.0066823
CTC_SC7	.1647377	1	.1412915	.1886488
OTC_SC7	.0023978	1	.0001512	.0072653
SN_SC7	.0020659	1	.0000687	.0066823
wave_d1#c.SN_SC7	.0001239	1	.	.0022403
SN_SC7	.0020659	1	.0000687	.0066823
wave_d2#c.SN_SC7	.0011484	1	.	.0049351

Note: Eta-Squared values for individual model terms are partial.

641 .

```
642 . *25.a.6 Regression with vce(ro)
643 . reg DV_Compliance_SC7 i.wave_d1 i.wave_d2 Age i.Gender_Female i.Minority Education i.Employed i.Corona_care i.Insura
    > rvative_01 i.Conservative_other i.GeoCensus_d1 i.GeoCensus_d2 i.GeoCensus_d3 i.Current_measures Measures_clear MA_Pe
    > _SC2 NOO_SC3 NNOO_SC3 OOL_SC12 PJE_SC4 Trust_Science_SC4 Trust_in_media Impulsivity_SC4 NegEmo_SC6 SN_SC7 CTC_SC7 OT
    note: SN_SC7 omitted because of collinearity
    note: SN_SC7 omitted because of collinearity

    Linear regression                               Number of obs     =      2,919
                                                    F(40, 2878)       =      67.37
                                                    Prob > F          =     0.0000
                                                    R-squared         =     0.5205
                                                    Root MSE          =     .90359
```

DV_Compliance_SC7	Coef.	Robust Std. Err.	t	P>|t|	[95% Conf.	Interval]
1.wave_d1	-.1861208	.1599931	-1.16	0.245	-.4998335	.1275919
1.wave_d2	-.4297011	.1721038	-2.50	0.013	-.7671603	-.0922419
Age	.0034079	.0014387	2.37	0.018	.0005869	.006229
1.Gender_Female	.1191131	.0354127	3.36	0.001	.0496763	.18855
1.Minority	-.0062236	.0389472	-0.16	0.873	-.0825908	.0701435
Education	.03476	.0120888	2.88	0.004	.0110564	.0584635
1.Employed	.0122547	.0405159	0.30	0.762	-.0671885	.0916978
1.Corona_care	-.0493676	.0702693	-0.70	0.482	-.1871508	.0884155
1.Insurance_Public	.0908149	.0600695	1.51	0.131	-.0269688	.2085986
1.Insurance_Private	.1335474	.0626018	2.13	0.033	.0107985	.2562963
SES_before	.0047498	.0103121	0.46	0.645	-.0154701	.0249697
SES_change	-7.57e-06	.0124401	-0.00	1.000	-.0244	.0243849
2.Health_self	.032449	.0370299	0.88	0.381	-.0401589	.1050569
2.Health_other	-.0174538	.0379331	-0.46	0.645	-.0918326	.0569251
1.Conservative_01	.0488903	.040856	1.20	0.232	-.0312197	.1290004
1.Conservative_other	.1036304	.0616554	1.68	0.093	-.0172629	.2245236
1.GeoCensus_d1	-.0980638	.0518387	-1.89	0.059	-.1997085	.003581
1.GeoCensus_d2	-.1282112	.0441203	-2.91	0.004	-.2147217	-.0417006
1.GeoCensus_d3	-.0056823	.0512287	-0.11	0.912	-.1061309	.0947664
Current_measures						
Yes	.1807226	.0639137	2.83	0.005	.0554013	.3060438
Measures_clear	-.0199137	.0121989	-1.63	0.103	-.0438332	.0040058
MA_Perc_Threat_SC3	.1260397	.0197165	6.39	0.000	.0873798	.1646997
Costs_SC5	.0266773	.012619	2.11	0.035	.001934	.0514205
Deterr_SD_Likely_SC2	.0057532	.0122649	0.47	0.639	-.0182956	.0298021
Deterr_SD_Severe	-.0041066	.0112041	-0.37	0.714	-.0260755	.0178623
MA_MoralBelief	.2464849	.0258844	9.52	0.000	.195731	.2972388
MA_Authority_SC2	-.0085187	.0108723	-0.78	0.433	-.029837	.0127995
NOO_SC3	.0179772	.0257581	0.70	0.485	-.0325289	.0684833
NNOO_SC3	.0217215	.0215887	1.01	0.314	-.0206095	.0640524
OOL_SC12	.01584	.0147137	1.08	0.282	-.0130105	.0446905
PJE_SC4	-.0099332	.012021	-0.83	0.409	-.0335037	.0136374
Trust_Science_SC4	.0357131	.0235319	1.52	0.129	-.0104278	.0818541
Trust_in_media	-.0125232	.0150111	-0.83	0.404	-.0419568	.0169105
Impulsivity_SC4	-.0848468	.0187679	-4.52	0.000	-.1216467	-.0480469
NegEmo_SC6	.0177601	.0128913	1.38	0.168	-.007517	.0430372
SN_SC7	.0041627	.0206997	0.20	0.841	-.0364252	.0447505
CTC_SC7	.5158264	.0320061	16.12	0.000	.4530693	.5785836
OTC_SC7	-.0268449	.0097342	-2.76	0.006	-.0459316	-.0077582
SN_SC7	0	(omitted)				
wave_d1#c.SN_SC7						
1	.0180586	.0283986	0.64	0.525	-.0376251	.0737424
SN_SC7	0	(omitted)				
wave_d2#c.SN_SC7						
1	.0548762	.0309624	1.77	0.076	-.0058346	.1155869
_cons	.0500781	.2325638	0.22	0.830	-.4059304	.5060865

```
644 .
645 .
646 . *****************
647 .
648 . *26. Model 26: Capacity x Survey Wave
649 .
650 . *26.a.2 Regression
651 . reg DV_Compliance_SC7 i.wave_d1 i.wave_d2 Age i.Gender_Female i.Minority Education i.Employed i.Corona_care i.Insura
    > rvative_01 i.Conservative_other i.GeoCensus_d1 i.GeoCensus_d2 i.GeoCensus_d3 i.Current_measures Measures_clear MA_Pe
    > _SC2 NOO_SC3 NNOO_SC3 OOL_SC12 PJE_SC4 Trust_Science_SC4 Trust_in_media Impulsivity_SC4 NegEmo_SC6 SN_SC7 CTC_SC7 OT
    note: CTC_SC7 omitted because of collinearity
    note: CTC_SC7 omitted because of collinearity

          Source |       SS           df       MS      Number of obs   =     2,919
    -------------+----------------------------------   F(40, 2878)     =     78.00
           Model |  2549.11246        40  63.7278115   Prob > F        =    0.0000
        Residual |  2351.41804     2,878  .817031981   R-squared       =    0.5202
    -------------+----------------------------------   Adj R-squared   =    0.5135
           Total |   4900.5305     2,918  1.67941415   Root MSE        =     .9039

    ------------------------------------------------------------------------------------
       DV_Compliance_SC7 |      Coef.   Std. Err.      t    P>|t|     [95% Conf. Interval]
    ---------------------+--------------------------------------------------------------
               1.wave_d1 |  -.1124156   .2630061    -0.43   0.669     -.628115    .4032839
               1.wave_d2 |  -.4130977   .2504969    -1.65   0.099    -.9042693    .0780738
                     Age |   .0035202   .0014411     2.44   0.015     .0006945    .0063459
         1.Gender_Female |   .1192628   .0353881     3.37   0.001     .0498743    .1886513
              1.Minority |  -.0049434   .0378112    -0.13   0.896    -.0790832    .0691964
               Education |   .0349201    .012281     2.84   0.004     .0108397    .0590005
              1.Employed |   .0126258   .0392356     0.32   0.748    -.0643069    .0895584
           1.Corona_care |  -.0462978    .065339    -0.71   0.479    -.1744137    .0818181
      1.Insurance_Public |   .0900233   .0538159     1.67   0.094    -.0154984     .195545
     1.Insurance_Private |   .1330321     .05624     2.37   0.018     .0227573    .2433069
              SES_before |   .0052247   .0095625     0.55   0.585    -.0135253    .0239748
              SES_change |   .0003906   .0112447     0.03   0.972    -.0216578     .022439
           2.Health_self |   .0330246   .0411409     0.80   0.422    -.0476439    .1136932
          2.Health_other |  -.0187895   .0390839    -0.48   0.631    -.0954248    .0578458
      1.Conservative_01 |   .0482703   .0403715     1.20   0.232    -.0308897    .1274303
    1.Conservative_other |   .1081655   .0579128     1.87   0.062    -.0053893    .2217204
          1.GeoCensus_d1 |  -.0989202   .0530974    -1.86   0.063     -.203033    .0051927
          1.GeoCensus_d2 |  -.1274823    .046098    -2.77   0.006    -.2178707   -.0370938
          1.GeoCensus_d3 |  -.0025811   .0566355    -0.05   0.964    -.1136313    .1084691
                         |
        Current_measures |
                     Yes |   .1807219   .0523752     3.45   0.001     .0780251    .2834186
          Measures_clear |  -.0201253   .0115222    -1.75   0.081    -.0427179    .0024674
       MA_Perc_Threat_SC3 |   .1263152    .017201     7.34   0.000     .0925877    .1600427
               Costs_SC5 |   .0263084   .0122613     2.15   0.032     .0022666    .0503501
     Deterr_SD_Likely_SC2 |   .0054826   .0120669     0.45   0.650     -.018178    .0291432
        Deterr_SD_Severe |  -.0037037   .0106399    -0.35   0.728    -.0245664    .0171589
          MA_MoralBelief |   .2464074   .0194746    12.65   0.000     .2082218    .2845931
        MA_Authority_SC2 |  -.0080531   .0112736    -0.71   0.475    -.0301582     .014052
                 NOO_SC3 |   .0170697   .0246737     0.69   0.489    -.0313102    .0654496
                NNOO_SC3 |   .0209647   .0211095     0.99   0.321    -.0204265    .0623558
                OOL_SC12 |   .0158463   .0142161     1.11   0.265    -.0120284    .0437209
                 PJE_SC4 |  -.0092359   .0121749    -0.76   0.448    -.0331083    .0146364
       Trust_Science_SC4 |   .0369025   .0220584     1.67   0.094    -.0063493    .0801543
          Trust_in_media |  -.0124136   .0157451    -0.79   0.431    -.0432864    .0184593
         Impulsivity_SC4 |  -.0854554   .0190532    -4.49   0.000    -.1228147   -.0480961
              NegEmo_SC6 |   .0175758   .0126348     1.39   0.164    -.0071985      .04235
                  SN_SC7 |   .0295426   .0145923     2.02   0.043     .0009301    .0581551
                 CTC_SC7 |   .4972113    .033284    14.94   0.000     .4319485    .5624741
                 OTC_SC7 |  -.0264541   .0102097    -2.59   0.010    -.0464733    -.006435
                 CTC_SC7 |          0  (omitted)
                         |
       wave_d1#c.CTC_SC7 |
                       1 |   .0041025   .0431928     0.09   0.924    -.0805894    .0887945
                         |
                 CTC_SC7 |          0  (omitted)
                         |
       wave_d2#c.CTC_SC7 |
                       1 |   .0454873   .0412196     1.10   0.270    -.0353355    .1263101
                         |
                   _cons |   .0127907   .2523328     0.05   0.960    -.4819807    .5075621
```

```
. 
. *26.a.3 Check hettest: Run this right after your regression to apply the Breusch-Pagan / Cook-Weisberg test for hete
. *if significant, then you need to run the regression with vce(ro) at the end
. estat hettest

Breusch-Pagan / Cook-Weisberg test for heteroskedasticity
         Ho: Constant variance
         Variables: fitted values of DV_Compliance_SC7

         chi2(1)      =   369.67
         Prob > chi2  =   0.0000

. 
. *26.a.4. check vif, to check for for multicollinearity (VIFs >10 are problematic)
. vif
```

Variable	VIF	1/VIF
1.wave_d1	55.28	0.018090
1.wave_d2	48.42	0.020654
Age	1.26	0.791130
1.Gender_F~e	1.11	0.901459
1.Minority	1.15	0.868859
Education	1.28	0.779898
1.Employed	1.27	0.788122
1.Corona_c~e	1.22	0.819440
1.Insuranc~c	2.54	0.393869
1.Insuran~te	2.35	0.426400
SES_before	1.37	0.728588
SES_change	1.24	0.809036
2.Health_s~f	1.36	0.737472
2.Health_o~r	1.33	0.754104
1.Conserv~01	1.44	0.693268
1.Conserva~r	1.26	0.790845
1.GeoCensu~1	1.66	0.603501
1.GeoCensu~2	1.86	0.538059
1.GeoCensu~3	1.54	0.648197
1.Current_~s	1.14	0.877667
Measures_c~r	1.42	0.706561
MA_Perc_Th~3	2.40	0.416916
Costs_SC5	1.44	0.692552
Deterr_SD_~2	1.62	0.618793
Deterr_SD_~e	1.19	0.837665
MA_MoralBe~f	2.27	0.439764
MA_Authori~2	1.62	0.616296
NOO_SC3	1.77	0.566309
NNOO_SC3	1.58	0.630966
OOL_SC12	1.59	0.630238
PJE_SC4	1.38	0.725484
Trust_Scie~4	1.69	0.591177
Trust_in_m~a	1.52	0.656573
Impulsivi~C4	1.64	0.610404
NegEmo_SC6	1.40	0.713072
SN_SC7	1.48	0.674719
CTC_SC7	3.88	0.257985
OTC_SC7	1.14	0.874157
wave_d1#		
c.CTC_SC7		
1	55.19	0.018121
wave_d2#		
c.CTC_SC7		
1	48.00	0.020833
Mean VIF	6.61	

```
659 .
660 . *26.a.5. Effect size
661 . estat esize
```

Effect sizes for linear models

Source	Eta-Squared	df	[95% Conf.	Interval]
Model	.5201707	40	.4909505	.5349587
wave_d1	.0000635	1	.	.0018998
wave_d2	.0009441	1	.	.0045031
Age	.002069	1	.0000694	.0066878
Gender_Female	.0039309	1	.0006878	.0097742
Minority	5.94e-06	1	.	.0009947
Education	.0028014	1	.0002689	.0079513
Employed	.000036	1	.	.0016585
Corona_care	.0001744	1	.	.0024548
Insurance_Public	.0009713	1	.	.0045622
Insurance_Private	.0019404	1	.0000407	.0064565
SES_before	.0001037	1	.	.0021413
SES_change	4.19e-07	1	.	.0000715
Health_self	.0002238	1	.	.0026372
Health_other	.0000803	1	.	.0020105
Conservative_01	.0004965	1	.	.0034457
Conservative_other	.0012106	1	.	.0050626
GeoCensus_d1	.0012045	1	.	.0050502
GeoCensus_d2	.0026503	1	.0002226	.0076971
GeoCensus_d3	7.22e-07	1	.	.00026
Current_measures	.0041199	1	.0007684	.0100684
Measures_clear	.0010589	1	.	.0047488
MA_Perc_Threat_SC3	.0183929	1	.0099272	.0292286
Costs_SC5	.0015971	1	.	.0058204
Deterr_SD_Likely_SC2	.0000717	1	.	.0019563
Deterr_SD_Severe	.0000421	1	.	.0017225
MA_MoralBelief	.0526948	1	.0379908	.0692538
MA_Authority_SC2	.0001773	1	.	.0024659
NOO_SC3	.0001663	1	.	.0024225
NNOO_SC3	.0003426	1	.	.0030177
OOL_SC12	.0004315	1	.	.0032714
PJE_SC4	.0001999	1	.	.0025514
Trust_Science_SC4	.0009715	1	.	.0045626
Trust_in_media	.0002159	1	.	.0026093
Impulsivity_SC4	.0069411	1	.0022025	.0142313
NegEmo_SC6	.0006719	1	.	.0038844
SN_SC7	.0014221	1	.	.0054838
CTC_SC7	.1352377	1	.1132476	.1580168
OTC_SC7	.0023273	1	.0001327	.0071431
CTC_SC7	.1352377	1	.1132476	.1580168
wave_d1#c.CTC_SC7	3.13e-06	1	.	.0007708
CTC_SC7	.1352377	1	.1132476	.1580168
wave_d2#c.CTC_SC7	.000423	1	.	.0032478

Note: Eta-Squared values for individual model terms are partial.

```
662 .
663 . *26.a.6 Regression with vce(ro)
664 . reg DV_Compliance_SC7 i.wave_d1 i.wave_d2 Age i.Gender_Female i.Minority Education i.Employed i.Corona_care i.Insura
    > rvative_01 i.Conservative_other i.GeoCensus_d1 i.GeoCensus_d2 i.GeoCensus_d3 i.Current_measures Measures_clear MA_Pe
    > _SC2 NOO_SC3 NNOO_SC3 OOL_SC12 PJE_SC4 Trust_Science_SC4 Trust_in_media Impulsivity_SC4 NegEmo_SC6 SN_SC7 CTC_SC7 OT
    note: CTC_SC7 omitted because of collinearity
    note: CTC_SC7 omitted because of collinearity
```

Linear regression

Number of obs	=	2,919
F(40, 2878)	=	67.75
Prob > F	=	0.0000
R-squared	=	0.5202
Root MSE	=	.9039

DV_Compliance_SC7	Coef.	Robust Std. Err.	t	P>|t|	[95% Conf.	Interval]
1.wave_d1	-.1124156	.3240086	-0.35	0.729	-.747728	.5228969
1.wave_d2	-.4130977	.3434171	-1.20	0.229	-1.086466	.2602706
Age	.0035202	.0014371	2.45	0.014	.0007023	.0063381
1.Gender_Female	.1192628	.0354244	3.37	0.001	.049803	.1887226
1.Minority	-.0049434	.0389414	-0.13	0.899	-.0812992	.0714124
Education	.0349201	.0120984	2.89	0.004	.0111977	.0586424
1.Employed	.0126258	.0404056	0.31	0.755	-.0666012	.0918527
1.Corona_care	-.0462978	.0700518	-0.66	0.509	-.1836546	.091059
1.Insurance_Public	.0900233	.0600425	1.50	0.134	-.0277074	.207754
1.Insurance_Private	.1330321	.0625481	2.13	0.034	.0103885	.2556757
SES_before	.0052247	.010338	0.51	0.613	-.0150458	.0254953
SES_change	.0003906	.0123789	0.03	0.975	-.0238817	.024663
2.Health_self	.0330246	.0369704	0.89	0.372	-.0394666	.1055158
2.Health_other	-.0187895	.0377739	-0.50	0.619	-.0928561	.0552771
1.Conservative_01	.0482703	.0408503	1.18	0.237	-.0318285	.1283691
1.Conservative_other	.1081655	.0616302	1.76	0.079	-.0126782	.2290093
1.GeoCensus_d1	-.0989202	.0518448	-1.91	0.056	-.2005769	.0027365
1.GeoCensus_d2	-.1274823	.0442335	-2.88	0.004	-.2142148	-.0407497
1.GeoCensus_d3	-.0025811	.0515979	-0.05	0.960	-.1037536	.0985914
Current_measures						
Yes	.1807219	.0638287	2.83	0.005	.0555672	.3058765
Measures_clear	-.0201253	.0122182	-1.65	0.100	-.0440826	.0038321
MA_Perc_Threat_SC3	.1263152	.0197434	6.40	0.000	.0876026	.1650278
Costs_SC5	.0263084	.0126414	2.08	0.038	.0015212	.0510955
Deterr_SD_Likely_SC2	.0054826	.0122315	0.45	0.654	-.0185008	.029466
Deterr_SD_Severe	-.0037037	.0111433	-0.33	0.740	-.0255533	.0181458
MA_MoralBelief	.2464074	.0258997	9.51	0.000	.1956236	.2971913
MA_Authority_SC2	-.0080531	.0109035	-0.74	0.460	-.0294325	.0133263
NOO_SC3	.0170697	.0257641	0.66	0.508	-.0334482	.0675876
NNOO_SC3	.0209647	.0215291	0.97	0.330	-.0212493	.0631786
OOL_SC12	.0158463	.0147132	1.08	0.282	-.0130033	.0446958
PJE_SC4	-.0092359	.0120197	-0.77	0.442	-.0328041	.0143322
Trust_Science_SC4	.0369025	.0234029	1.58	0.115	-.0089856	.0827907
Trust_in_media	-.0124136	.0150429	-0.83	0.409	-.0419095	.0170824
Impulsivity_SC4	-.0854554	.0187343	-4.56	0.000	-.1221893	-.0487215
NegEmo_SC6	.0175758	.0129184	1.36	0.174	-.0077545	.042906
SN_SC7	.0295426	.0146575	2.02	0.044	.0008023	.0582829
CTC_SC7	.4972113	.0426826	11.65	0.000	.4135197	.5809029
OTC_SC7	-.0264541	.0097584	-2.71	0.007	-.0455884	-.0073199
CTC_SC7	0	(omitted)				
wave_d1#c.CTC_SC7						
1	.0041025	.0514265	0.08	0.936	-.096734	.1049391
CTC_SC7	0	(omitted)				
wave_d2#c.CTC_SC7						
1	.0454873	.0547294	0.83	0.406	-.0618255	.1528001
_cons	.0127907	.2920793	0.04	0.965	-.5599151	.5854965

```
.
.
. *****************
.
. *27. Model 27: Opportunity x Survey Wave
```

```
670 .
671 . *27.a.2 Regression
672 . reg DV_Compliance_SC7 i.wave_d1 i.wave_d2 Age i.Gender_Female i.Minority Education i.Employed i.Corona_care i.Insura
    > rvative_01 i.Conservative_other i.GeoCensus_d1 i.GeoCensus_d2 i.GeoCensus_d3 i.Current_measures Measures_clear MA_Pe
    > _SC2 NOO_SC3 NNOO_SC3 OOL_SC12 PJE_SC4 Trust_Science_SC4 Trust_in_media Impulsivity_SC4 NegEmo_SC6 SN_SC7 CTC_SC7 OT
    note: OTC_SC7 omitted because of collinearity
    note: OTC_SC7 omitted because of collinearity
```

Source	SS	df	MS
Model	2548.03481	40	63.7008701
Residual	2352.4957	2,878	.817406426
Total	4900.5305	2,918	1.67941415

Number of obs	=	2,919
F(40, 2878)	=	77.93
Prob > F	=	0.0000
R-squared	=	0.5200
Adj R-squared	=	0.5133
Root MSE	=	.90411

DV_Compliance_SC7	Coef.	Std. Err.	t	P>|t|	[95% Conf.	Interval]
1.wave_d1	-.0995046	.1133485	-0.88	0.380	-.3217572	.1227479
1.wave_d2	-.1905606	.1158166	-1.65	0.100	-.4176525	.0365313
Age	.0034894	.0014416	2.42	0.016	.0006626	.0063161
1.Gender_Female	.1196381	.0353981	3.38	0.001	.0502299	.1890464
1.Minority	-.005021	.0378112	-0.13	0.894	-.0791608	.0691188
Education	.034862	.0122833	2.84	0.005	.0107771	.0589469
1.Employed	.0140315	.0392324	0.36	0.721	-.0628949	.0909578
1.Corona_care	-.0468549	.0653569	-0.72	0.473	-.1750059	.0812962
1.Insurance_Public	.0903969	.0538053	1.68	0.093	-.0151039	.1958978
1.Insurance_Private	.1335282	.056254	2.37	0.018	.023226	.2438303
SES_before	.0050818	.0095606	0.53	0.595	-.0136645	.0238282
SES_change	.0001743	.0112473	0.02	0.988	-.0218793	.0222279
2.Health_self	.0346388	.0411259	0.84	0.400	-.0460004	.115278
2.Health_other	-.0192907	.0390903	-0.49	0.622	-.0959385	.0573572
1.Conservative_01	.049781	.0403722	1.23	0.218	-.0293803	.1289422
1.Conservative_other	.1067835	.0579267	1.84	0.065	-.0067985	.2203655
1.GeoCensus_d1	-.098128	.053127	-1.85	0.065	-.2022989	.0060429
1.GeoCensus_d2	-.1251091	.0460746	-2.72	0.007	-.2154516	-.0347666
1.GeoCensus_d3	.0006936	.0565644	0.01	0.990	-.1102173	.1116045
Current_measures						
Yes	.1793378	.0523631	3.42	0.001	.0766648	.2820107
Measures_clear	-.019833	.0115285	-1.72	0.085	-.0424381	.002772
MA_Perc_Threat_SC3	.1257557	.0171945	7.31	0.000	.0920409	.1594705
Costs_SC5	.0265533	.012271	2.16	0.031	.0024925	.0506141
Deterr_SD_Likely_SC2	.0059805	.012074	0.50	0.620	-.017694	.029655
Deterr_SD_Severe	-.003438	.0106332	-0.32	0.746	-.0242874	.0174115
MA_MoralBelief	.2464083	.0194796	12.65	0.000	.2082128	.2846037
MA_Authority_SC2	-.0084464	.0112811	-0.75	0.454	-.0305663	.0136734
NOO_SC3	.0170899	.0246844	0.69	0.489	-.031311	.0654908
NNOO_SC3	.0201291	.0211114	0.95	0.340	-.0212659	.061524
OOL_SC12	.0153953	.0142127	1.08	0.279	-.0124728	.0432633
PJE_SC4	-.0097915	.0121667	-0.80	0.421	-.0336478	.0140647
Trust_Science_SC4	.0379691	.0220479	1.72	0.085	-.0052621	.0812003
Trust_in_media	-.0122386	.0157494	-0.78	0.437	-.0431197	.0186426
Impulsivity_SC4	-.0853201	.0190647	-4.48	0.000	-.1227021	-.0479382
NegEmo_SC6	.0176431	.0126385	1.40	0.163	-.0071382	.0424245
SN_SC7	.0292504	.0146076	2.00	0.045	.000608	.0578929
CTC_SC7	.5159537	.0216535	23.83	0.000	.4734958	.5584116
OTC_SC7	-.030609	.0163942	-1.87	0.062	-.0627546	.0015366
OTC_SC7	0	(omitted)				
wave_d1#c.OTC_SC7						
1	.0030303	.0230157	0.13	0.895	-.0420986	.0481593
OTC_SC7	0	(omitted)				
wave_d2#c.OTC_SC7						
1	.0107579	.0237007	0.45	0.650	-.0357141	.0572298
_cons	-.0795751	.2074505	-0.38	0.701	-.4863416	.3271915

```
673 .
674 . *27.a.3 Check hettest: Run this right after your regression to apply the Breusch-Pagan / Cook-Weisberg test for hete
675 . *if significant, then you need to run the regression with vce(ro) at the end
676 . estat hettest

    Breusch-Pagan / Cook-Weisberg test for heteroskedasticity
             Ho: Constant variance
             Variables: fitted values of DV_Compliance_SC7

             chi2(1)      =   367.73
             Prob > chi2  =   0.0000

677 .
678 . *27.a.4. check vif, to check for for multicollinearity (VIFs >10 are problematic)
679 . vif

        Variable |       VIF       1/VIF
    -------------+----------------------
       1.wave_d1 |     10.26    0.097439
       1.wave_d2 |     10.34    0.096666
             Age |      1.26    0.790892
    1.Gender_F~e |      1.11    0.901359
      1.Minority |      1.15    0.869257
       Education |      1.28    0.779966
      1.Employed |      1.27    0.788613
    1.Corona_c~e |      1.22    0.819367
    1.Insuranc~c |      2.54    0.394205
    1.Insuran~te |      2.35    0.426384
      SES_before |      1.37    0.729211
      SES_change |      1.24    0.809023
    2.Health_s~f |      1.35    0.738347
    2.Health_o~r |      1.33    0.754203
    1.Conserv~01 |      1.44    0.693563
    1.Conserva~r |      1.26    0.790829
    1.GeoCensu~1 |      1.66    0.603105
    1.GeoCensu~2 |      1.86    0.538853
    1.GeoCensu~3 |      1.54    0.650124
    1.Current_~s |      1.14    0.878477
    Measures_c~r |      1.42    0.706109
    MA_Perc_Th~3 |      2.40    0.417422
       Costs_SC5 |      1.45    0.691772
    Deterr_SD_~2 |      1.62    0.618352
    Deterr_SD_~e |      1.19    0.839112
    MA_MoralBe~f |      2.27    0.439740
    MA_Authori~2 |      1.62    0.615755
         NOO_SC3 |      1.77    0.566076
        NNOO_SC3 |      1.58    0.631140
        OOL_SC12 |      1.59    0.630826
         PJE_SC4 |      1.38    0.726796
    Trust_Scie~4 |      1.69    0.592010
    Trust_in_m~a |      1.52    0.656519
    Impulsivi~C4 |      1.64    0.609945
      NegEmo_SC6 |      1.40    0.712991
          SN_SC7 |      1.48    0.673614
         CTC_SC7 |      1.64    0.609830
         OTC_SC7 |      2.95    0.339184
         wave_d1#|
       c.OTC_SC7 |
              1  |     11.30    0.088508
         wave_d2#|
       c.OTC_SC7 |
              1  |     11.07    0.090337
    -------------+----------------------
        Mean VIF |      2.50
```

680 .
681 . *27.a.5. Effect size
682 . estat esize

Effect sizes for linear models

Source	Eta-Squared	df	[95% Conf.	Interval]
Model	.5199508	40	.4907208	.5347429
wave_d1	.0002677	1	.	.0027852
wave_d2	.0009398	1	.	.0044938
Age	.0020314	1	.0000609	.0066206
Gender_Female	.0039534	1	.0006973	.0098093
Minority	6.13e-06	1	.	.0010056
Education	.0027911	1	.0002657	.0079341
Employed	.0000444	1	.	.0017449
Corona_care	.0001785	1	.	.0024709
Insurance_Public	.0009798	1	.	.0045804
Insurance_Private	.0019539	1	.0000437	.0064809
SES_before	.0000982	1	.	.0021121
SES_change	8.35e-08	1	.	.
Health_self	.0002464	1	.	.0027148
Health_other	.0000846	1	.	.0020362
Conservative_01	.000528	1	.	.0035277
Conservative_other	.0011794	1	.	.0049988
GeoCensus_d1	.001184	1	.	.0050083
GeoCensus_d2	.0025554	1	.0001949	.0075359
GeoCensus_d3	5.22e-08	1	.	.
Current_measures	.0040592	1	.0007423	.0099741
Measures_clear	.0010273	1	.	.0046819
MA_Perc_Threat_SC3	.0182468	1	.0098188	.029047
Costs_SC5	.0016244	1	.	.005872
Deterr_SD_Likely_SC2	.0000852	1	.	.0020399
Deterr_SD_Severe	.0000363	1	.	.0016623
MA_MoralBelief	.0526695	1	.037969	.0692255
MA_Authority_SC2	.0001947	1	.	.0025323
NOO_SC3	.0001665	1	.	.0024235
NNOO_SC3	.0003158	1	.	.0029368
OOL_SC12	.0004075	1	.	.0032048
PJE_SC4	.000225	1	.	.0026413
Trust_Science_SC4	.0010294	1	.	.0046864
Trust_in_media	.0002098	1	.	.0025873
Impulsivity_SC4	.006911	1	.0021854	.0141886
NegEmo_SC6	.0006767	1	.	.0038958
SN_SC7	.0013913	1	.	.0054234
CTC_SC7	.1647711	1	.1413235	.1886833
OTC_SC7	.0012495	1	.	.0051414
wave_d1#c.OTC_SC7	6.02e-06	1	.	.0009996
OTC_SC7	.0012495	1	.	.0051414
wave_d2#c.OTC_SC7	.0000716	1	.	.0019554

Note: Eta-Squared values for individual model terms are partial.

683 .
684 . *27.a.6 Regression with vce(ro)
685 . reg DV_Compliance_SC7 i.wave_d1 i.wave_d2 Age i.Gender_Female i.Minority Education i.Employed i.Corona_care i.Insura
> rvative_01 i.Conservative_other i.GeoCensus_d1 i.GeoCensus_d2 i.GeoCensus_d3 i.Current_measures Measures_clear MA_Pe
> _SC2 NOO_SC3 NNOO_SC3 OOL_SC12 PJE_SC4 Trust_Science_SC4 Trust_in_media Impulsivity_SC4 NegEmo_SC6 SN_SC7 CTC_SC7 OT
note: OTC_SC7 omitted because of collinearity
note: OTC_SC7 omitted because of collinearity

Linear regression

Number of obs	=	2,919
F(40, 2878)	=	67.18
Prob > F	=	0.0000
R-squared	=	0.5200
Root MSE	=	.90411

DV_Compliance_SC7	Coef.	Robust Std. Err.	t	P>|t|	[95% Conf.	Interval]
1.wave_d1	-.0995046	.1031722	-0.96	0.335	-.3018035	.1027942
1.wave_d2	-.1905606	.1054569	-1.81	0.071	-.3973392	.0162181
Age	.0034894	.0014375	2.43	0.015	.0006708	.0063079
1.Gender_Female	.1196381	.035454	3.37	0.001	.0501202	.189156
1.Minority	-.005021	.0389087	-0.13	0.897	-.0813128	.0712708
Education	.034862	.0120955	2.88	0.004	.0111452	.0585788
1.Employed	.0140315	.0404214	0.35	0.729	-.0652264	.0932893
1.Corona_care	-.0468549	.0702452	-0.67	0.505	-.1845908	.0908811
1.Insurance_Public	.0903969	.060138	1.50	0.133	-.0275209	.2083148
1.Insurance_Private	.1335282	.0626435	2.13	0.033	.0106976	.2563588
SES_before	.0050818	.0103735	0.49	0.624	-.0152584	.025422
SES_change	.0001743	.0124337	0.01	0.989	-.0242055	.0245541
2.Health_self	.0346388	.0369397	0.94	0.348	-.0377922	.1070699
2.Health_other	-.0192907	.0378043	-0.51	0.610	-.0934169	.0548355
1.Conservative_01	.049781	.0408379	1.22	0.223	-.0302935	.1298554
1.Conservative_other	.1067835	.0616813	1.73	0.084	-.0141605	.2277275
1.GeoCensus_d1	-.098128	.0518379	-1.89	0.058	-.1997712	.0035152
1.GeoCensus_d2	-.1251091	.0442617	-2.83	0.005	-.211897	-.0383213
1.GeoCensus_d3	.0006936	.0514095	0.01	0.989	-.1001095	.1014967
Current_measures						
Yes	.1793378	.0639205	2.81	0.005	.0540032	.3046724
Measures_clear	-.019833	.0121791	-1.63	0.104	-.0437136	.0040475
MA_Perc_Threat_SC3	.1257557	.0197093	6.38	0.000	.08711	.1644014
Costs_SC5	.0265533	.0126086	2.11	0.035	.0018304	.0512761
Deterr_SD_Likely_SC2	.0059805	.0122744	0.49	0.626	-.018087	.030048
Deterr_SD_Severe	-.003438	.0111273	-0.31	0.757	-.0252563	.0183804
MA_MoralBelief	.2464083	.0259497	9.50	0.000	.1955263	.2972902
MA_Authority_SC2	-.0084464	.010867	-0.78	0.437	-.0297543	.0128614
NOO_SC3	.0170899	.0257661	0.66	0.507	-.0334319	.0676117
NNOO_SC3	.0201291	.0215549	0.93	0.350	-.0221356	.0623937
OOL_SC12	.0153953	.0147503	1.04	0.297	-.013527	.0443175
PJE_SC4	-.0097915	.0120273	-0.81	0.416	-.0333746	.0137916
Trust_Science_SC4	.0379691	.0234993	1.62	0.106	-.0081081	.0840463
Trust_in_media	-.0122386	.015045	-0.81	0.416	-.0417387	.0172616
Impulsivity_SC4	-.0853201	.018749	-4.55	0.000	-.122083	-.0485573
NegEmo_SC6	.0176431	.0129214	1.37	0.172	-.007693	.0429793
SN_SC7	.0292504	.0146146	2.00	0.045	.0005943	.0579066
CTC_SC7	.5159537	.0319967	16.13	0.000	.453215	.5786924
OTC_SC7	-.030609	.0148324	-2.06	0.039	-.0596921	-.0015259
OTC_SC7	0	(omitted)				
wave_d1#c.OTC_SC7						
1	.0030303	.0212881	0.14	0.887	-.0387112	.0447719
OTC_SC7	0	(omitted)				
wave_d2#c.OTC_SC7						
1	.0107579	.0223509	0.48	0.630	-.0330675	.0545832
_cons	-.0795751	.2176484	-0.37	0.715	-.5063376	.3471875

```
.
.
.
. *****************
.
```

```
691 . *28. Mediation model
692 .
693 . *28.a.0 Install paramed package
694 . ssc install paramed
    checking paramed consistency and verifying not already installed...
    all files already exist and are up to date.

695 . help paramed

696 .
697 . *28.a.1 Mediation model, wave (1-2) on compliance mediated by knowledge
698 . paramed DV_Compliance_SC7, avar(wave_d1) mvar(Current_measures) cvars(wave_d2 Age Gender_Female Minority Education E
    > Health_other Conservative_01 Conservative_other GeoCensus_d1 GeoCensus_d2 GeoCensus_d3 Measures_clear MA_Perc_Threat
    > SC3 NNOO_SC3 OOL_SC12 PJE_SC4 Trust_Science_SC4 Trust_in_media Impulsivity_SC4 NegEmo_SC6 SN_SC7 CTC_SC7 OTC_SC7) a0
```

Source	SS	df	MS
Model	5783.88315	38	152.207451
Residual	4685.07115	4,309	1.08727574
Total	10468.9543	4,347	2.40831707

Number of obs	=	4,348
F(38, 4309)	=	139.99
Prob > F	=	0.0000
R-squared	=	0.5525
Adj R-squared	=	0.5485
Root MSE	=	1.0427

DV_Compliance_SC7	Coef.	Std. Err.	t	P>|t|	[95% Conf.	Interval]
wave_d1	-.1008503	.0393389	-2.56	0.010	-.1779747	-.0237259
Current_measures	.2499748	.0438322	5.70	0.000	.1640412	.3359085
wave_d2	-.1057489	.0396427	-2.67	0.008	-.1834691	-.0280287
Age	.0033071	.0012878	2.57	0.010	.0007823	.0058319
Gender_Female	.1356169	.0331373	4.09	0.000	.0706507	.2005831
Minority	-.0115737	.0342592	-0.34	0.736	-.0787394	.055592
Education	.0269047	.0111445	2.41	0.016	.0050558	.0487536
Employed	.0456376	.0366804	1.24	0.213	-.0262749	.1175501
Corona_care	.0163155	.053475	0.31	0.760	-.088523	.1211539
Insurance_Public	.1594496	.0483136	3.30	0.001	.06473	.2541691
Insurance_Private	.1641472	.050507	3.25	0.001	.0651275	.2631669
SES_before	.0073995	.008939	0.83	0.408	-.0101256	.0249246
SES_change	-.0057423	.0103144	-0.56	0.578	-.0259637	.0144792
Health_self	-.0606208	.0384104	-1.58	0.115	-.135925	.0146833
Health_other	.0013416	.0370705	0.04	0.971	-.0713357	.0740189
Conservative_01	.0104904	.0367129	0.29	0.775	-.0614859	.0824667
Conservative_other	.0324492	.0536565	0.60	0.545	-.0727451	.1376435
GeoCensus_d1	-.0347888	.0503101	-0.69	0.489	-.1334224	.0638449
GeoCensus_d2	-.0517626	.043166	-1.20	0.231	-.1363901	.0328649
GeoCensus_d3	.016889	.0530092	0.32	0.750	-.0870362	.1208143
Measures_clear	.0045911	.0107412	0.43	0.669	-.0164671	.0256494
MA_Perc_Threat_SC3	.2237231	.0156131	14.33	0.000	.1931133	.2543328
Costs_SC5	.0190175	.0119631	1.59	0.112	-.0044364	.0424713
Deterr_SD_Likely_SC2	.0035187	.0115967	0.30	0.762	-.0192168	.0262542
Deterr_SD_Severe	-.0029309	.0101497	-0.29	0.773	-.0228295	.0169678
MA_MoralBelief	.2074829	.0163511	12.69	0.000	.1754264	.2395394
MA_Authority_SC2	-.005595	.0109528	-0.51	0.609	-.0270681	.0158781
NOO_SC3	.0002182	.0238173	0.01	0.993	-.046476	.0469123
NNOO_SC3	-.0291422	.0208513	-1.40	0.162	-.0700214	.0117371
OOL_SC12	.0295314	.013935	2.12	0.034	.0022117	.0568512
PJE_SC4	-.0267302	.0123098	-2.17	0.030	-.0508637	-.0025966
Trust_Science_SC4	.0090169	.0210033	0.43	0.668	-.0321604	.0501941
Trust_in_media	-.0415326	.0149385	-2.78	0.005	-.0708198	-.0122454
Impulsivity_SC4	-.084699	.0184327	-4.60	0.000	-.1208366	-.0485615
NegEmo_SC6	.0156761	.012208	1.28	0.199	-.0082577	.03961
SN_SC7	.0632409	.0147381	4.29	0.000	.0343466	.0921352
CTC_SC7	.4347295	.018896	23.01	0.000	.3976837	.4717753
OTC_SC7	-.0312296	.0104532	-2.99	0.003	-.0517233	-.010736
_cons	.1463905	.1804394	0.81	0.417	-.2073636	.5001446

```
Iteration 0:   log likelihood =  -2187.344
Iteration 1:   log likelihood = -1797.1313
Iteration 2:   log likelihood =  -1758.911
Iteration 3:   log likelihood =  -1758.007
Iteration 4:   log likelihood = -1758.0053
```

Logistic regression

Number of obs	=	4348
LR chi2(37)	=	858.68
Prob > chi2	=	0.0000
Pseudo R2	=	0.1963

Log likelihood = -1758.0053

Current_me~s	Coef.	Std. Err.	z	P>|z|	[95% Conf.	Interval]
wave_d1	-.3940417	.1072898	-3.67	0.000	-.6043259	-.1837575
wave_d2	-.2888808	.1099154	-2.63	0.009	-.5043111	-.0734505
Age	.0108263	.0036012	3.01	0.003	.003768	.0178846
Gender_Fem~e	-.035776	.0888065	-0.40	0.687	-.2098336	.1382815
Minority	.1639175	.0923877	1.77	0.076	-.0171591	.3449941
Education	-.107286	.0291958	-3.67	0.000	-.1645087	-.0500634
Employed	.1570713	.0977511	1.61	0.108	-.0345174	.3486599
Corona_care	.3103666	.1371132	2.26	0.024	.0416297	.5791035
Insurance_~c	.2259258	.1213882	1.86	0.063	-.0119907	.4638423
Insurance_~e	.2294008	.1274999	1.80	0.072	-.0204944	.479296
SES_before	.06895	.0243863	2.83	0.005	.0211537	.1167462
SES_change	.006826	.0281308	0.24	0.808	-.0483094	.0619614
Health_self	.0411643	.1063612	0.39	0.699	-.1672999	.2496284
Health_other	.3612378	.0993309	3.64	0.000	.1665529	.5559228
Conservat~01	.0466672	.0980686	0.48	0.634	-.1455437	.2388781
Conservati~r	.0007955	.1418518	0.01	0.996	-.277229	.27882
GeoCensus_d1	-.1919587	.138598	-1.39	0.166	-.4636058	.0796884
GeoCensus_d2	-.3636337	.120066	-3.03	0.002	-.5989586	-.1283087
GeoCensus_d3	-.0797235	.1499844	-0.53	0.595	-.3736876	.2142406
Measures_c~r	.2305441	.0272235	8.47	0.000	.1771871	.2839011
MA_Perc_Th~3	.0713604	.0393279	1.81	0.070	-.0057208	.1484417
Costs_SC5	.0132211	.0339535	0.39	0.697	-.0533265	.0797687
Deterr_SD_~2	-.0173926	.0332483	-0.52	0.601	-.082558	.0477727
Deterr_SD_~e	-.0798677	.0281588	-2.84	0.005	-.135058	-.0246773
MA_MoralBe~f	.1335988	.0382075	3.50	0.000	.0587135	.2084841
MA_Authori~2	-.0923422	.0322966	-2.86	0.004	-.1556423	-.0290421
NOO_SC3	.1065076	.0637474	1.67	0.095	-.0184351	.2314502
NNOO_SC3	-.1184427	.061006	-1.94	0.052	-.2380123	.0011269
OOL_SC12	.0500958	.0404223	1.24	0.215	-.0291305	.129322
PJE_SC4	.0624386	.0342831	1.82	0.069	-.004755	.1296323
Trust_Scie~4	.0428913	.0575242	0.75	0.456	-.069854	.1556366
Trust_in_m~a	-.0719575	.0419782	-1.71	0.086	-.1542332	.0103183
Impulsivi~C4	-.2261238	.0514913	-4.39	0.000	-.3270449	-.1252027
NegEmo_SC6	.0148764	.034801	0.43	0.669	-.0533323	.0830851
SN_SC7	.0839816	.0426377	1.97	0.049	.0004133	.1675498
CTC_SC7	.3460265	.0466464	7.42	0.000	.2546012	.4374518
OTC_SC7	-.0553518	.0343515	-1.61	0.107	-.1226794	.0119758
_cons	-2.977365	.4876234	-6.11	0.000	-3.933089	-2.02164

	Estimate	Std Err	P>|z|	[95% Conf	Interval]
cde	-.10085033	.03933886	0.010	-.1779545	-.02374617
nde	-.10085033	.03933886	0.010	-.1779545	-.02374617
nie	-.01286545	.00429513	0.003	-.02128391	-.00444699
mte	-.11371578	.0395288	0.004	-.19119222	-.03623934

cde:controlled direct effect, nde:natural direct effect, nie:natural indirect effect, mte:marginal total effect

	Observed Coef.	Bias	Bootstrap Std. Err.	[95% Conf.	Interval]	
cde	-.10085033	-.0006079	.03946667	-.1861741	-.0287204	(BC)
nde	-.10085033	-.0006079	.03946667	-.1861741	-.0287204	(BC)
nie	-.01286545	.0002637	.00434595	-.0244482	-.0065816	(BC)
mte	-.11371578	-.0003442	.03935632	-.1960464	-.0403347	(BC)

(BC)   bias-corrected confidence interval

```
699 .
700 . *28.a.2 Mediation model, wave (1-3) on compliance mediated by knowledge
701 . paramed DV_Compliance_SC7, avar(wave_d2) mvar(Current_measures) cvars(wave_d1 Age Gender_Female Minority Education E
    > Health_other Conservative_01 Conservative_other GeoCensus_d1 GeoCensus_d2 GeoCensus_d3 Measures_clear MA_Perc_Threat
    > SC3 NNOO_SC3 OOL_SC12 PJE_SC4 Trust_Science_SC4 Trust_in_media Impulsivity_SC4 NegEmo_SC6 SN_SC7 CTC_SC7 OTC_SC7) a0
```

Source	SS	df	MS
Model	5783.88315	38	152.207451
Residual	4685.07115	4,309	1.08727574
Total	10468.9543	4,347	2.40831707

Number of obs	=	4,348
F(38, 4309)	=	139.99
Prob > F	=	0.0000
R-squared	=	0.5525
Adj R-squared	=	0.5485
Root MSE	=	1.0427

DV_Compliance_SC7	Coef.	Std. Err.	t	P>|t|	[95% Conf.	Interval]
wave_d2	-.1057489	.0396427	-2.67	0.008	-.1834691	-.0280287
Current_measures	.2499748	.0438322	5.70	0.000	.1640412	.3359085
wave_d1	-.1008503	.0393389	-2.56	0.010	-.1779747	-.0237259
Age	.0033071	.0012878	2.57	0.010	.0007823	.0058319
Gender_Female	.1356169	.0331373	4.09	0.000	.0706507	.2005831
Minority	-.0115737	.0342592	-0.34	0.736	-.0787394	.055592
Education	.0269047	.0111445	2.41	0.016	.0050558	.0487536
Employed	.0456376	.0366804	1.24	0.213	-.0262749	.1175501
Corona_care	.0163155	.053475	0.31	0.760	-.088523	.1211539
Insurance_Public	.1594496	.0483136	3.30	0.001	.06473	.2541691
Insurance_Private	.1641472	.050507	3.25	0.001	.0651275	.2631669
SES_before	.0073995	.008939	0.83	0.408	-.0101256	.0249246
SES_change	-.0057423	.0103144	-0.56	0.578	-.0259637	.0144792
Health_self	-.0606208	.0384104	-1.58	0.115	-.135925	.0146833
Health_other	.0013416	.0370705	0.04	0.971	-.0713357	.0740189
Conservative_01	.0104904	.0367129	0.29	0.775	-.0614859	.0824667
Conservative_other	.0324492	.0536565	0.60	0.545	-.0727451	.1376435
GeoCensus_d1	-.0347888	.0503101	-0.69	0.489	-.1334224	.0638449
GeoCensus_d2	-.0517626	.043166	-1.20	0.231	-.1363901	.0328649
GeoCensus_d3	.016889	.0530092	0.32	0.750	-.0870362	.1208143
Measures_clear	.0045911	.0107412	0.43	0.669	-.0164671	.0256494
MA_Perc_Threat_SC3	.2237231	.0156131	14.33	0.000	.1931133	.2543328
Costs_SC5	.0190175	.0119631	1.59	0.112	-.0044364	.0424713
Deterr_SD_Likely_SC2	.0035187	.0115967	0.30	0.762	-.0192168	.0262542
Deterr_SD_Severe	-.0029309	.0101497	-0.29	0.773	-.0228295	.0169678
MA_MoralBelief	.2074829	.0163511	12.69	0.000	.1754264	.2395394
MA_Authority_SC2	-.005595	.0109528	-0.51	0.609	-.0270681	.0158781
NOO_SC3	.0002182	.0238173	0.01	0.993	-.046476	.0469123
NNOO_SC3	-.0291422	.0208513	-1.40	0.162	-.0700214	.0117371
OOL_SC12	.0295314	.013935	2.12	0.034	.0022117	.0568512
PJE_SC4	-.0267302	.0123098	-2.17	0.030	-.0508637	-.0025966
Trust_Science_SC4	.0090169	.0210033	0.43	0.668	-.0321604	.0501941
Trust_in_media	-.0415326	.0149385	-2.78	0.005	-.0708198	-.0122454
Impulsivity_SC4	-.084699	.0184327	-4.60	0.000	-.1208366	-.0485615
NegEmo_SC6	.0156761	.012208	1.28	0.199	-.0082577	.03961
SN_SC7	.0632409	.0147381	4.29	0.000	.0343466	.0921352
CTC_SC7	.4347295	.018896	23.01	0.000	.3976837	.4717753
OTC_SC7	-.0312296	.0104532	-2.99	0.003	-.0517233	-.010736
_cons	.1463905	.1804394	0.81	0.417	-.2073636	.5001446

```
Iteration 0:   log likelihood =  -2187.344
Iteration 1:   log likelihood = -1797.1313
Iteration 2:   log likelihood =  -1758.911
Iteration 3:   log likelihood =  -1758.007
Iteration 4:   log likelihood = -1758.0053
```

Logistic regression

Number of obs	=	4348
LR chi2(37)	=	858.68
Prob > chi2	=	0.0000
Pseudo R2	=	0.1963

Log likelihood = -1758.0053

Current_me~s	Coef.	Std. Err.	z	P>|z|	[95% Conf.	Interval]
wave_d2	-.2888808	.1099154	-2.63	0.009	-.5043111	-.0734505
wave_d1	-.3940417	.1072898	-3.67	0.000	-.6043259	-.1837575
Age	.0108263	.0036012	3.01	0.003	.003768	.0178846
Gender_Fem~e	-.035776	.0888065	-0.40	0.687	-.2098336	.1382815
Minority	.1639175	.0923877	1.77	0.076	-.0171591	.3449941
Education	-.107286	.0291958	-3.67	0.000	-.1645087	-.0500634
Employed	.1570713	.0977511	1.61	0.108	-.0345174	.3486599
Corona_care	.3103666	.1371132	2.26	0.024	.0416297	.5791035
Insurance_~c	.2259258	.1213882	1.86	0.063	-.0119907	.4638423
Insurance_~e	.2294008	.1274999	1.80	0.072	-.0204944	.479296
SES_before	.06895	.0243863	2.83	0.005	.0211537	.1167462
SES_change	.006826	.0281308	0.24	0.808	-.0483094	.0619614
Health_self	.0411643	.1063612	0.39	0.699	-.1672999	.2496284
Health_other	.3612378	.0993309	3.64	0.000	.1665529	.5559228
Conservat~01	.0466672	.0980686	0.48	0.634	-.1455437	.2388781
Conservati~r	.0007955	.1418518	0.01	0.996	-.277229	.27882
GeoCensus_d1	-.1919587	.138598	-1.39	0.166	-.4636058	.0796884
GeoCensus_d2	-.3636337	.120066	-3.03	0.002	-.5989586	-.1283087
GeoCensus_d3	-.0797235	.1499844	-0.53	0.595	-.3736876	.2142406
Measures_c~r	.2305441	.0272235	8.47	0.000	.1771871	.2839011
MA_Perc_Th~3	.0713604	.0393279	1.81	0.070	-.0057208	.1484417
Costs_SC5	.0132211	.0339535	0.39	0.697	-.0533265	.0797687
Deterr_SD_~2	-.0173926	.0332483	-0.52	0.601	-.082558	.0477727
Deterr_SD_~e	-.0798677	.0281588	-2.84	0.005	-.135058	-.0246773
MA_MoralBe~f	.1335988	.0382075	3.50	0.000	.0587135	.2084841
MA_Authori~2	-.0923422	.0322966	-2.86	0.004	-.1556423	-.0290421
NOO_SC3	.1065076	.0637474	1.67	0.095	-.0184351	.2314502
NNOO_SC3	-.1184427	.061006	-1.94	0.052	-.2380123	.0011269
OOL_SC12	.0500958	.0404223	1.24	0.215	-.0291305	.129322
PJE_SC4	.0624386	.0342831	1.82	0.069	-.004755	.1296323
Trust_Scie~4	.0428913	.0575242	0.75	0.456	-.069854	.1556366
Trust_in_m~a	-.0719575	.0419782	-1.71	0.086	-.1542332	.0103183
Impulsivi~C4	-.2261238	.0514913	-4.39	0.000	-.3270449	-.1252027
NegEmo_SC6	.0148764	.034801	0.43	0.669	-.0533323	.0830851
SN_SC7	.0839816	.0426377	1.97	0.049	.0004133	.1675498
CTC_SC7	.3460265	.0466464	7.42	0.000	.2546012	.4374518
OTC_SC7	-.0553518	.0343515	-1.61	0.107	-.1226794	.0119758
_cons	-2.977365	.4876234	-6.11	0.000	-3.933089	-2.02164

	Estimate	Std Err	P>|z|	[95% Conf	Interval]
cde	-.1057489	.03964275	0.008	-.18344868	-.02804911
nde	-.1057489	.03964275	0.008	-.18344868	-.02804911
nie	-.00932425	.00401563	0.020	-.01719488	-.00145361
mte	-.11507314	.03985471	0.004	-.19318838	-.03695791

cde:controlled direct effect, nde:natural direct effect, nie:natural indirect effect, mte:marginal total effect

	Observed Coef.	Bias	Bootstrap Std. Err.	[95% Conf.	Interval]	
cde	-.1057489	-.0013102	.03856689	-.1794706	-.0272605	(BC)
nde	-.1057489	-.0013102	.03856689	-.1794706	-.0272605	(BC)
nie	-.00932425	.0002571	.003958	-.0189364	-.003127	(BC)
mte	-.11507314	-.001053	.0384153	-.188351	-.0368643	(BC)

(BC)   bias-corrected confidence interval

```
702 .
703 . *28.b.1 Mediation model, wave (1-2) on compliance mediated by perceived threat
704 . paramed DV_Compliance_SC7, avar(wave_d1) mvar(MA_Perc_Threat_SC3) cvars(wave_d2 Age Gender_Female Minority Educatior
    > f Health_other Conservative_01 Conservative_other GeoCensus_d1 GeoCensus_d2 GeoCensus_d3 Current_measures Measures_c
    > SC3 NNOO_SC3 OOL_SC12 PJE_SC4 Trust_Science_SC4 Trust_in_media Impulsivity_SC4 NegEmo_SC6 SN_SC7 CTC_SC7 OTC_SC7) a0
```

Source	SS	df	MS			
				Number of obs	=	4,348
				F(38, 4309)	=	139.99
Model	5783.88315	38	152.207451	Prob > F	=	0.0000
Residual	4685.07115	4,309	1.08727574	R-squared	=	0.5525
				Adj R-squared	=	0.5485
Total	10468.9543	4,347	2.40831707	Root MSE	=	1.0427

DV_Compliance_SC7	Coef.	Std. Err.	t	P>|t|	[95% Conf.	Interval]
wave_d1	-.1008503	.0393389	-2.56	0.010	-.1779747	-.0237259
MA_Perc_Threat_SC3	.2237231	.0156131	14.33	0.000	.1931133	.2543328
wave_d2	-.1057489	.0396427	-2.67	0.008	-.1834691	-.0280287
Age	.0033071	.0012878	2.57	0.010	.0007823	.0058319
Gender_Female	.1356169	.0331373	4.09	0.000	.0706507	.2005831
Minority	-.0115737	.0342592	-0.34	0.736	-.0787394	.055592
Education	.0269047	.0111445	2.41	0.016	.0050558	.0487536
Employed	.0456376	.0366804	1.24	0.213	-.0262749	.1175501
Corona_care	.0163155	.053475	0.31	0.760	-.088523	.1211539
Insurance_Public	.1594496	.0483136	3.30	0.001	.06473	.2541691
Insurance_Private	.1641472	.050507	3.25	0.001	.0651275	.2631669
SES_before	.0073995	.008939	0.83	0.408	-.0101256	.0249246
SES_change	-.0057423	.0103144	-0.56	0.578	-.0259637	.0144792
Health_self	-.0606208	.0384104	-1.58	0.115	-.135925	.0146833
Health_other	.0013416	.0370705	0.04	0.971	-.0713357	.0740189
Conservative_01	.0104904	.0367129	0.29	0.775	-.0614859	.0824667
Conservative_other	.0324492	.0536565	0.60	0.545	-.0727451	.1376435
GeoCensus_d1	-.0347888	.0503101	-0.69	0.489	-.1334224	.0638449
GeoCensus_d2	-.0517626	.043166	-1.20	0.231	-.1363901	.0328649
GeoCensus_d3	.016889	.0530092	0.32	0.750	-.0870362	.1208143
Current_measures	.2499748	.0438322	5.70	0.000	.1640412	.3359085
Measures_clear	.0045911	.0107412	0.43	0.669	-.0164671	.0256494
Costs_SC5	.0190175	.0119631	1.59	0.112	-.0044364	.0424713
Deterr_SD_Likely_SC2	.0035187	.0115967	0.30	0.762	-.0192168	.0262542
Deterr_SD_Severe	-.0029309	.0101497	-0.29	0.773	-.0228295	.0169678
MA_MoralBelief	.2074829	.0163511	12.69	0.000	.1754264	.2395394
MA_Authority_SC2	-.005595	.0109528	-0.51	0.609	-.0270681	.0158781
NOO_SC3	.0002182	.0238173	0.01	0.993	-.046476	.0469123
NNOO_SC3	-.0291422	.0208513	-1.40	0.162	-.0700214	.0117371
OOL_SC12	.0295314	.013935	2.12	0.034	.0022117	.0568512
PJE_SC4	-.0267302	.0123098	-2.17	0.030	-.0508637	-.0025966
Trust_Science_SC4	.0090169	.0210033	0.43	0.668	-.0321604	.0501941
Trust_in_media	-.0415326	.0149385	-2.78	0.005	-.0708198	-.0122454
Impulsivity_SC4	-.084699	.0184327	-4.60	0.000	-.1208366	-.0485615
NegEmo_SC6	.0156761	.012208	1.28	0.199	-.0082577	.03961
SN_SC7	.0632409	.0147381	4.29	0.000	.0343466	.0921352
CTC_SC7	.4347295	.018896	23.01	0.000	.3976837	.4717753
OTC_SC7	-.0312296	.0104532	-2.99	0.003	-.0517233	-.010736
_cons	.1463905	.1804394	0.81	0.417	-.2073636	.5001446

Source	SS	df	MS			
				Number of obs	=	4,348
				F(37, 4310)	=	169.15
Model	6476.56015	37	175.042166	Prob > F	=	0.0000
Residual	4460.25933	4,310	1.03486295	R-squared	=	0.5922
				Adj R-squared	=	0.5887
Total	10936.8195	4,347	2.51594651	Root MSE	=	1.0173

MA_Perc_Threat_SC3	Coef.	Std. Err.	t	P>|t|	[95% Conf.	Interval]
wave_d1	.0499007	.0383714	1.30	0.194	-.0253271	.1251285
wave_d2	.1445349	.0386127	3.74	0.000	.0688341	.2202357
Age	.0040212	.0012549	3.20	0.001	.001561	.0064815
Gender_Female	-.0047292	.0323287	-0.15	0.884	-.0681101	.0586516
Minority	.1504549	.0333446	4.51	0.000	.0850823	.2158275
Education	-.010407	.0108714	-0.96	0.338	-.0317205	.0109066
Employed	.0064063	.0357853	0.18	0.858	-.0637512	.0765639
Corona_care	.0310366	.052168	0.59	0.552	-.0712395	.1333127
Insurance_Public	.039788	.0471308	0.84	0.399	-.0526127	.1321887
Insurance_Private	.0163995	.0492739	0.33	0.739	-.0802028	.1130018
SES_before	-.0278167	.0087106	-3.19	0.001	-.044894	-.0107395
SES_change	-.0330253	.0100501	-3.29	0.001	-.0527287	-.0133219
Health_self	.2221508	.0373201	5.95	0.000	.1489843	.2953174
Health_other	.0654124	.0361523	1.81	0.070	-.0054646	.1362894
Conservative_01	-.0749305	.0357989	-2.09	0.036	-.1451149	-.0047462
Conservative_other	.0088442	.0523471	0.17	0.866	-.093783	.1114714
GeoCensus_d1	-.0655274	.0490723	-1.34	0.182	-.1617345	.0306796
GeoCensus_d2	-.0076459	.0421125	-0.18	0.856	-.0902082	.0749163
GeoCensus_d3	.0173004	.051715	0.33	0.738	-.0840877	.1186885
Current_measures	.0510167	.0427556	1.19	0.233	-.0328062	.1348397
Measures_clear	.0184918	.0104753	1.77	0.078	-.0020452	.0390288
Costs_SC5	.0654781	.0116285	5.63	0.000	.0426803	.088276
Deterr_SD_Likely_SC2	.0479841	.0112901	4.25	0.000	.0258497	.0701184
Deterr_SD_Severe	-.0158318	.0098991	-1.60	0.110	-.0352391	.0035756
MA_MoralBelief	.5626535	.0134543	41.82	0.000	.5362762	.5890308
MA_Authority_SC2	.0124647	.0106839	1.17	0.243	-.0084812	.0334105
NOO_SC3	.1564466	.0231136	6.77	0.000	.1111321	.2017612
NNOO_SC3	.0046167	.0203424	0.23	0.820	-.0352649	.0444982
OOL_SC12	.0444453	.0135781	3.27	0.001	.0178253	.0710654
PJE_SC4	-.0121052	.012008	-1.01	0.313	-.0356471	.0114367
Trust_Science_SC4	.1054682	.0204277	5.16	0.000	.0654193	.145517
Trust_in_media	.1075392	.0144817	7.43	0.000	.0791477	.1359307
Impulsivity_SC4	.0193341	.0179805	1.08	0.282	-.0159169	.0545852
NegEmo_SC6	.0838068	.0118415	7.08	0.000	.0605914	.1070222
SN_SC7	-.0182896	.0143758	-1.27	0.203	-.0464735	.0098944
CTC_SC7	.0546752	.0184161	2.97	0.003	.0185702	.0907802
OTC_SC7	.0261766	.0101903	2.57	0.010	.0061983	.0461549
_cons	-1.128955	.1751946	-6.44	0.000	-1.472427	-.7854833

	Estimate	Std Err	P>|z|	[95% Conf	Interval]
cde	-.10085033	.03933886	0.010	-.1779545	-.02374617
nie	.01116393	.00861986	0.195	-.00573099	.02805885
te	-.0896864	.0402571	0.026	-.16859031	-.01078249

cde:controlled direct effect, nie:natural indirect effect, te:total effect

	Observed Coef.	Bias	Bootstrap Std. Err.	[95% Conf.	Interval]	
cde	-.10085033	-.0006079	.03946667	-.1861741	-.0287204	(BC)
nie	.01116393	.0005842	.00877216	-.00799	.0281221	(BC)
te	-.0896864	-.0000237	.04034447	-.1743738	-.0148648	(BC)

(BC) bias-corrected confidence interval

705 .

```
706 . *28.b.2 Mediation model, wave (1-3) on compliance mediated by perceived threat
707 . paramed DV_Compliance_SC7, avar(wave_d2) mvar(MA_Perc_Threat_SC3) cvars(wave_d1 Age Gender_Female Minority Educatio
    > f Health_other Conservative_01 Conservative_other GeoCensus_d1 GeoCensus_d2 GeoCensus_d3 Current_measures Measures_c
    > SC3 NNOO_SC3 OOL_SC12 PJE_SC4 Trust_Science_SC4 Trust_in_media Impulsivity_SC4 NegEmo_SC6 SN_SC7 CTC_SC7 OTC_SC7) a0
```

Source	SS	df	MS			
				Number of obs	=	4,348
				F(38, 4309)	=	139.99
Model	5783.88315	38	152.207451	Prob > F	=	0.0000
Residual	4685.07115	4,309	1.08727574	R-squared	=	0.5525
				Adj R-squared	=	0.5485
Total	10468.9543	4,347	2.40831707	Root MSE	=	1.0427

DV_Compliance_SC7	Coef.	Std. Err.	t	P>|t|	[95% Conf.	Interval]
wave_d2	-.1057489	.0396427	-2.67	0.008	-.1834691	-.0280287
MA_Perc_Threat_SC3	.2237231	.0156131	14.33	0.000	.1931133	.2543328
wave_d1	-.1008503	.0393389	-2.56	0.010	-.1779747	-.0237259
Age	.0033071	.0012878	2.57	0.010	.0007823	.0058319
Gender_Female	.1356169	.0331373	4.09	0.000	.0706507	.2005831
Minority	-.0115737	.0342592	-0.34	0.736	-.0787394	.055592
Education	.0269047	.0111445	2.41	0.016	.0050558	.0487536
Employed	.0456376	.0366804	1.24	0.213	-.0262749	.1175501
Corona_care	.0163155	.053475	0.31	0.760	-.088523	.1211539
Insurance_Public	.1594496	.0483136	3.30	0.001	.06473	.2541691
Insurance_Private	.1641472	.050507	3.25	0.001	.0651275	.2631669
SES_before	.0073995	.008939	0.83	0.408	-.0101256	.0249246
SES_change	-.0057423	.0103144	-0.56	0.578	-.0259637	.0144792
Health_self	-.0606208	.0384104	-1.58	0.115	-.135925	.0146833
Health_other	.0013416	.0370705	0.04	0.971	-.0713357	.0740189
Conservative_01	.0104904	.0367129	0.29	0.775	-.0614859	.0824667
Conservative_other	.0324492	.0536565	0.60	0.545	-.0727451	.1376435
GeoCensus_d1	-.0347888	.0503101	-0.69	0.489	-.1334224	.0638449
GeoCensus_d2	-.0517626	.043166	-1.20	0.231	-.1363901	.0328649
GeoCensus_d3	.016889	.0530092	0.32	0.750	-.0870362	.1208143
Current_measures	.2499748	.0438322	5.70	0.000	.1640412	.3359085
Measures_clear	.0045911	.0107412	0.43	0.669	-.0164671	.0256494
Costs_SC5	.0190175	.0119631	1.59	0.112	-.0044364	.0424713
Deterr_SD_Likely_SC2	.0035187	.0115967	0.30	0.762	-.0192168	.0262542
Deterr_SD_Severe	-.0029309	.0101497	-0.29	0.773	-.0228295	.0169678
MA_MoralBelief	.2074829	.0163511	12.69	0.000	.1754264	.2395394
MA_Authority_SC2	-.005595	.0109528	-0.51	0.609	-.0270681	.0158781
NOO_SC3	.0002182	.0238173	0.01	0.993	-.046476	.0469123
NNOO_SC3	-.0291422	.0208513	-1.40	0.162	-.0700214	.0117371
OOL_SC12	.0295314	.013935	2.12	0.034	.0022117	.0568512
PJE_SC4	-.0267302	.0123098	-2.17	0.030	-.0508637	-.0025966
Trust_Science_SC4	.0090169	.0210033	0.43	0.668	-.0321604	.0501941
Trust_in_media	-.0415326	.0149385	-2.78	0.005	-.0708198	-.0122454
Impulsivity_SC4	-.084699	.0184327	-4.60	0.000	-.1208366	-.0485615
NegEmo_SC6	.0156761	.012208	1.28	0.199	-.0082577	.03961
SN_SC7	.0632409	.0147381	4.29	0.000	.0343466	.0921352
CTC_SC7	.4347295	.018896	23.01	0.000	.3976837	.4717753
OTC_SC7	-.0312296	.0104532	-2.99	0.003	-.0517233	-.010736
_cons	.1463905	.1804394	0.81	0.417	-.2073636	.5001446

Source	SS	df	MS			
				Number of obs	=	4,348
				F(37, 4310)	=	169.15
Model	6476.56015	37	175.042166	Prob > F	=	0.0000
Residual	4460.25933	4,310	1.03486295	R-squared	=	0.5922
				Adj R-squared	=	0.5887
Total	10936.8195	4,347	2.51594651	Root MSE	=	1.0173

MA_Perc_Threat_SC3	Coef.	Std. Err.	t	P>|t|	[95% Conf.	Interval]
wave_d2	.1445349	.0386127	3.74	0.000	.0688341	.2202357
wave_d1	.0499007	.0383714	1.30	0.194	-.0253271	.1251285
Age	.0040212	.0012549	3.20	0.001	.001561	.0064815
Gender_Female	-.0047292	.0323287	-0.15	0.884	-.0681101	.0586516
Minority	.1504549	.0333446	4.51	0.000	.0850823	.2158275
Education	-.010407	.0108714	-0.96	0.338	-.0317205	.0109066
Employed	.0064063	.0357853	0.18	0.858	-.0637512	.0765639
Corona_care	.0310366	.052168	0.59	0.552	-.0712395	.1333127
Insurance_Public	.039788	.0471308	0.84	0.399	-.0526127	.1321887
Insurance_Private	.0163995	.0492739	0.33	0.739	-.0802028	.1130018
SES_before	-.0278167	.0087106	-3.19	0.001	-.044894	-.0107395
SES_change	-.0330253	.0100501	-3.29	0.001	-.0527287	-.0133219
Health_self	.2221508	.0373201	5.95	0.000	.1489843	.2953174
Health_other	.0654124	.0361523	1.81	0.070	-.0054646	.1362894
Conservative_01	-.0749305	.0357989	-2.09	0.036	-.1451149	-.0047462
Conservative_other	.0088442	.0523471	0.17	0.866	-.093783	.1114714
GeoCensus_d1	-.0655274	.0490723	-1.34	0.182	-.1617345	.0306796
GeoCensus_d2	-.0076459	.0421125	-0.18	0.856	-.0902082	.0749163
GeoCensus_d3	.0173004	.051715	0.33	0.738	-.0840877	.1186885
Current_measures	.0510167	.0427556	1.19	0.233	-.0328062	.1348397
Measures_clear	.0184918	.0104753	1.77	0.078	-.0020452	.0390288
Costs_SC5	.0654781	.0116285	5.63	0.000	.0426803	.088276
Deterr_SD_Likely_SC2	.0479841	.0112901	4.25	0.000	.0258497	.0701184
Deterr_SD_Severe	-.0158318	.0098991	-1.60	0.110	-.0352391	.0035756
MA_MoralBelief	.5626535	.0134543	41.82	0.000	.5362762	.5890308
MA_Authority_SC2	.0124647	.0106839	1.17	0.243	-.0084812	.0334105
NOO_SC3	.1564466	.0231136	6.77	0.000	.1111321	.2017612
NNOO_SC3	.0046167	.0203424	0.23	0.820	-.0352649	.0444982
OOL_SC12	.0444453	.0135781	3.27	0.001	.0178253	.0710654
PJE_SC4	-.0121052	.012008	-1.01	0.313	-.0356471	.0114367
Trust_Science_SC4	.1054682	.0204277	5.16	0.000	.0654193	.145517
Trust_in_media	.1075392	.0144817	7.43	0.000	.0791477	.1359307
Impulsivity_SC4	.0193341	.0179805	1.08	0.282	-.0159169	.0545852
NegEmo_SC6	.0838068	.0118415	7.08	0.000	.0605914	.1070222
SN_SC7	-.0182896	.0143758	-1.27	0.203	-.0464735	.0098944
CTC_SC7	.0546752	.0184161	2.97	0.003	.0185702	.0907802
OTC_SC7	.0261766	.0101903	2.57	0.010	.0061983	.0461549
_cons	-1.128955	.1751946	-6.44	0.000	-1.472427	-.7854833

	Estimate	Std Err	P>|z|	[95% Conf	Interval]
cde	-.1057489	.03964275	0.008	-.18344868	-.02804911
nie	.03233579	.00892844	0.000	.01483604	.04983554
te	-.0734131	.04051024	0.070	-.15281318	.00598697

cde:controlled direct effect, nie:natural indirect effect, te:total effect

	Observed Coef.	Bias	Bootstrap Std. Err.	[95% Conf.	Interval]	
cde	-.1057489	-.0013102	.03856689	-.1794706	-.0272605	(BC)
nie	.03233579	-.0000671	.00918282	.0160366	.0513678	(BC)
te	-.0734131	-.0013773	.03925837	-.1478188	.0067907	(BC)

(BC)   bias-corrected confidence interval

708 .

```
709 . *28.c.1 Mediation model, wave (1-2) on compliance mediated by costs
710 . paramed DV_Compliance_SC7, avar(wave_d1) mvar(Costs_SC5) cvars(wave_d2 Age Gender_Female Minority Education Employed
    > other Conservative_01 Conservative_other GeoCensus_d1 GeoCensus_d2 GeoCensus_d3 Current_measures Measures_clear MA_P
    > SC3 NNOO_SC3 OOL_SC12 PJE_SC4 Trust_Science_SC4 Trust_in_media Impulsivity_SC4 NegEmo_SC6 SN_SC7 CTC_SC7 OTC_SC7) a0
```

Source	SS	df	MS			
				Number of obs	=	4,348
				F(38, 4309)	=	139.99
Model	5783.88315	38	152.207451	Prob > F	=	0.0000
Residual	4685.07115	4,309	1.08727574	R-squared	=	0.5525
				Adj R-squared	=	0.5485
Total	10468.9543	4,347	2.40831707	Root MSE	=	1.0427

DV_Compliance_SC7	Coef.	Std. Err.	t	P>|t|	[95% Conf.	Interval]
wave_d1	-.1008503	.0393389	-2.56	0.010	-.1779747	-.0237259
Costs_SC5	.0190175	.0119631	1.59	0.112	-.0044364	.0424713
wave_d2	-.1057489	.0396427	-2.67	0.008	-.1834691	-.0280287
Age	.0033071	.0012878	2.57	0.010	.0007823	.0058319
Gender_Female	.1356169	.0331373	4.09	0.000	.0706507	.2005831
Minority	-.0115737	.0342592	-0.34	0.736	-.0787394	.055592
Education	.0269047	.0111445	2.41	0.016	.0050558	.0487536
Employed	.0456376	.0366804	1.24	0.213	-.0262749	.1175501
Corona_care	.0163155	.053475	0.31	0.760	-.088523	.1211539
Insurance_Public	.1594496	.0483136	3.30	0.001	.06473	.2541691
Insurance_Private	.1641472	.050507	3.25	0.001	.0651275	.2631669
SES_before	.0073995	.008939	0.83	0.408	-.0101256	.0249246
SES_change	-.0057423	.0103144	-0.56	0.578	-.0259637	.0144792
Health_self	-.0606208	.0384104	-1.58	0.115	-.135925	.0146833
Health_other	.0013416	.0370705	0.04	0.971	-.0713357	.0740189
Conservative_01	.0104904	.0367129	0.29	0.775	-.0614859	.0824667
Conservative_other	.0324492	.0536565	0.60	0.545	-.0727451	.1376435
GeoCensus_d1	-.0347888	.0503101	-0.69	0.489	-.1334224	.0638449
GeoCensus_d2	-.0517626	.043166	-1.20	0.231	-.1363901	.0328649
GeoCensus_d3	.016889	.0530092	0.32	0.750	-.0870362	.1208143
Current_measures	.2499748	.0438322	5.70	0.000	.1640412	.3359085
Measures_clear	.0045911	.0107412	0.43	0.669	-.0164671	.0256494
MA_Perc_Threat_SC3	.2237231	.0156131	14.33	0.000	.1931133	.2543328
Deterr_SD_Likely_SC2	.0035187	.0115967	0.30	0.762	-.0192168	.0262542
Deterr_SD_Severe	-.0029309	.0101497	-0.29	0.773	-.0228295	.0169678
MA_MoralBelief	.2074829	.0163511	12.69	0.000	.1754264	.2395394
MA_Authority_SC2	-.005595	.0109528	-0.51	0.609	-.0270681	.0158781
NOO_SC3	.0002182	.0238173	0.01	0.993	-.046476	.0469123
NNOO_SC3	-.0291422	.0208513	-1.40	0.162	-.0700214	.0117371
OOL_SC12	.0295314	.013935	2.12	0.034	.0022117	.0568512
PJE_SC4	-.0267302	.0123098	-2.17	0.030	-.0508637	-.0025966
Trust_Science_SC4	.0090169	.0210033	0.43	0.668	-.0321604	.0501941
Trust_in_media	-.0415326	.0149385	-2.78	0.005	-.0708198	-.0122454
Impulsivity_SC4	-.084699	.0184327	-4.60	0.000	-.1208366	-.0485615
NegEmo_SC6	.0156761	.012208	1.28	0.199	-.0082577	.03961
SN_SC7	.0632409	.0147381	4.29	0.000	.0343466	.0921352
CTC_SC7	.4347295	.018896	23.01	0.000	.3976837	.4717753
OTC_SC7	-.0312296	.0104532	-2.99	0.003	-.0517233	-.010736
_cons	.1463905	.1804394	0.81	0.417	-.2073636	.5001446

Source	SS	df	MS			
				Number of obs	=	4,348
				F(37, 4310)	=	52.02
Model	3392.48689	37	91.6888349	Prob > F	=	0.0000
Residual	7597.16583	4,310	1.76268349	R-squared	=	0.3087
				Adj R-squared	=	0.3028
Total	10989.6527	4,347	2.52810047	Root MSE	=	1.3277

Costs_SC5	Coef.	Std. Err.	t	P>|t|	[95% Conf.	Interval]
wave_d1	-.1422493	.0500417	-2.84	0.004	-.2403568	-.0441417
wave_d2	-.1396685	.0504307	-2.77	0.006	-.2385386	-.0407984
Age	-.0081206	.0016351	-4.97	0.000	-.0113262	-.004915
Gender_Female	.0570207	.0421835	1.35	0.177	-.0256807	.1397221
Minority	.0467253	.0436151	1.07	0.284	-.0387828	.1322333
Education	.0272727	.0141837	1.92	0.055	-.0005348	.0550801
Employed	.0572418	.0466956	1.23	0.220	-.0343056	.1487891
Corona_care	-.0481651	.0680836	-0.71	0.479	-.181644	.0853138
Insurance_Public	-.353385	.0612799	-5.77	0.000	-.4735251	-.2332449
Insurance_Private	-.2939127	.0641525	-4.58	0.000	-.4196846	-.1681407
SES_before	-.0556327	.0113501	-4.90	0.000	-.0778848	-.0333807
SES_change	-.1347294	.0129715	-10.39	0.000	-.1601603	-.1092985
Health_self	.0058985	.0489064	0.12	0.904	-.0899831	.1017802
Health_other	.1647816	.0471337	3.50	0.000	.0723754	.2571879
Conservative_01	-.1685595	.0466746	-3.61	0.000	-.2600657	-.0770533
Conservative_other	-.0843625	.0683066	-1.24	0.217	-.2182785	.0495536
GeoCensus_d1	-.0403149	.0640549	-0.63	0.529	-.1658955	.0852656
GeoCensus_d2	.0104202	.0549613	0.19	0.850	-.0973323	.1181726
GeoCensus_d3	.0208909	.0674937	0.31	0.757	-.1114316	.1532133
Current_measures	.0181059	.0558091	0.32	0.746	-.0913086	.1275205
Measures_clear	-.0243493	.0136713	-1.78	0.075	-.0511521	.0024535
MA_Perc_Threat_SC3	.111529	.0198069	5.63	0.000	.0726974	.1503606
Deterr_SD_Likely_SC2	.2041545	.0144344	14.14	0.000	.1758556	.2324534
Deterr_SD_Severe	-.0589906	.012892	-4.58	0.000	-.0842655	-.0337157
MA_MoralBelief	-.0003532	.0208192	-0.02	0.986	-.0411695	.0404631
MA_Authority_SC2	-.023528	.0139412	-1.69	0.092	-.0508598	.0038039
NOO_SC3	.0556341	.0303137	1.84	0.067	-.0037965	.1150646
NNOO_SC3	.069556	.026528	2.62	0.009	.0175475	.1215645
OOL_SC12	-.1246906	.0176409	-7.07	0.000	-.1592759	-.0901054
PJE_SC4	.0086806	.015673	0.55	0.580	-.0220466	.0394078
Trust_Science_SC4	-.0071862	.0267424	-0.27	0.788	-.0596151	.0452427
Trust_in_media	-.0473993	.0190069	-2.49	0.013	-.0846626	-.010136
Impulsivity_SC4	.0257691	.0234663	1.10	0.272	-.020237	.0717752
NegEmo_SC6	.2416428	.0151018	16.00	0.000	.2120355	.2712502
SN_SC7	-.0062461	.0187652	-0.33	0.739	-.0430356	.0305434
CTC_SC7	.0443716	.02405	1.84	0.065	-.0027788	.0915219
OTC_SC7	.0316717	.0133009	2.38	0.017	.0055951	.0577483
_cons	2.676337	.2261007	11.84	0.000	2.233064	3.119611

	Estimate	Std Err	P>|z|	[95% Conf	Interval]
cde	-.10085033	.03933886	0.010	-.1779545	-.02374617
nie	-.00270522	.00194977	0.165	-.00652677	.00111633
te	-.10355556	.03931356	0.008	-.18061013	-.02650099

cde:controlled direct effect, nie:natural indirect effect, te:total effect

	Observed Coef.	Bias	Bootstrap Std. Err.	[95% Conf.	Interval]	
cde	-.10085033	-.0006079	.03946667	-.1861741	-.0287204	(BC)
nie	-.00270522	.0000906	.00207903	-.0092814	.0000736	(BC)
te	-.10355556	-.0005173	.03928631	-.1883164	-.0287757	(BC)

(BC) bias-corrected confidence interval

711 .

```
712 . *28.c.2 Mediation model, wave (1-3) on compliance mediated by costs
713 . paramed DV_Compliance_SC7, avar(wave_d2) mvar(Costs_SC5) cvars(wave_d1 Age Gender_Female Minority Education Employed
    > other Conservative_01 Conservative_other GeoCensus_d1 GeoCensus_d2 GeoCensus_d3 Current_measures Measures_clear MA_P
    > SC3 NNOO_SC3 OOL_SC12 PJE_SC4 Trust_Science_SC4 Trust_in_media Impulsivity_SC4 NegEmo_SC6 SN_SC7 CTC_SC7 OTC_SC7) a0
```

Source	SS	df	MS
Model	5783.88315	38	152.207451
Residual	4685.07115	4,309	1.08727574
Total	10468.9543	4,347	2.40831707

Number of obs	=	4,348
F(38, 4309)	=	139.99
Prob > F	=	0.0000
R-squared	=	0.5525
Adj R-squared	=	0.5485
Root MSE	=	1.0427

DV_Compliance_SC7	Coef.	Std. Err.	t	P>|t|	[95% Conf.	Interval]
wave_d2	-.1057489	.0396427	-2.67	0.008	-.1834691	-.0280287
Costs_SC5	.0190175	.0119631	1.59	0.112	-.0044364	.0424713
wave_d1	-.1008503	.0393389	-2.56	0.010	-.1779747	-.0237259
Age	.0033071	.0012878	2.57	0.010	.0007823	.0058319
Gender_Female	.1356169	.0331373	4.09	0.000	.0706507	.2005831
Minority	-.0115737	.0342592	-0.34	0.736	-.0787394	.055592
Education	.0269047	.0111445	2.41	0.016	.0050558	.0487536
Employed	.0456376	.0366804	1.24	0.213	-.0262749	.1175501
Corona_care	.0163155	.053475	0.31	0.760	-.088523	.1211539
Insurance_Public	.1594496	.0483136	3.30	0.001	.06473	.2541691
Insurance_Private	.1641472	.050507	3.25	0.001	.0651275	.2631669
SES_before	.0073995	.008939	0.83	0.408	-.0101256	.0249246
SES_change	-.0057423	.0103144	-0.56	0.578	-.0259637	.0144792
Health_self	-.0606208	.0384104	-1.58	0.115	-.135925	.0146833
Health_other	.0013416	.0370705	0.04	0.971	-.0713357	.0740189
Conservative_01	.0104904	.0367129	0.29	0.775	-.0614859	.0824667
Conservative_other	.0324492	.0536565	0.60	0.545	-.0727451	.1376435
GeoCensus_d1	-.0347888	.0503101	-0.69	0.489	-.1334224	.0638449
GeoCensus_d2	-.0517626	.043166	-1.20	0.231	-.1363901	.0328649
GeoCensus_d3	.016889	.0530092	0.32	0.750	-.0870362	.1208143
Current_measures	.2499748	.0438322	5.70	0.000	.1640412	.3359085
Measures_clear	.0045911	.0107412	0.43	0.669	-.0164671	.0256494
MA_Perc_Threat_SC3	.2237231	.0156131	14.33	0.000	.1931133	.2543328
Deterr_SD_Likely_SC2	.0035187	.0115967	0.30	0.762	-.0192168	.0262542
Deterr_SD_Severe	-.0029309	.0101497	-0.29	0.773	-.0228295	.0169678
MA_MoralBelief	.2074829	.0163511	12.69	0.000	.1754264	.2395394
MA_Authority_SC2	-.005595	.0109528	-0.51	0.609	-.0270681	.0158781
NOO_SC3	.0002182	.0238173	0.01	0.993	-.046476	.0469123
NNOO_SC3	-.0291422	.0208513	-1.40	0.162	-.0700214	.0117371
OOL_SC12	.0295314	.013935	2.12	0.034	.0022117	.0568512
PJE_SC4	-.0267302	.0123098	-2.17	0.030	-.0508637	-.0025966
Trust_Science_SC4	.0090169	.0210033	0.43	0.668	-.0321604	.0501941
Trust_in_media	-.0415326	.0149385	-2.78	0.005	-.0708198	-.0122454
Impulsivity_SC4	-.084699	.0184327	-4.60	0.000	-.1208366	-.0485615
NegEmo_SC6	.0156761	.012208	1.28	0.199	-.0082577	.03961
SN_SC7	.0632409	.0147381	4.29	0.000	.0343466	.0921352
CTC_SC7	.4347295	.018896	23.01	0.000	.3976837	.4717753
OTC_SC7	-.0312296	.0104532	-2.99	0.003	-.0517233	-.010736
_cons	.1463905	.1804394	0.81	0.417	-.2073636	.5001446

Source	SS	df	MS
Model	3392.48689	37	91.6888349
Residual	7597.16583	4,310	1.76268349
Total	10989.6527	4,347	2.52810047

Number of obs	=	4,348
F(37, 4310)	=	52.02
Prob > F	=	0.0000
R-squared	=	0.3087
Adj R-squared	=	0.3028
Root MSE	=	1.3277

Costs_SC5	Coef.	Std. Err.	t	P>|t|	[95% Conf.	Interval]
wave_d2	-.1396685	.0504307	-2.77	0.006	-.2385386	-.0407984
wave_d1	-.1422493	.0500417	-2.84	0.004	-.2403568	-.0441417
Age	-.0081206	.0016351	-4.97	0.000	-.0113262	-.004915
Gender_Female	.0570207	.0421835	1.35	0.177	-.0256807	.1397221
Minority	.0467253	.0436151	1.07	0.284	-.0387828	.1322333
Education	.0272727	.0141837	1.92	0.055	-.0005348	.0550801
Employed	.0572418	.0466956	1.23	0.220	-.0343056	.1487891
Corona_care	-.0481651	.0680836	-0.71	0.479	-.181644	.0853138
Insurance_Public	-.353385	.0612799	-5.77	0.000	-.4735251	-.2332449
Insurance_Private	-.2939127	.0641525	-4.58	0.000	-.4196846	-.1681407
SES_before	-.0556327	.0113501	-4.90	0.000	-.0778848	-.0333807
SES_change	-.1347294	.0129715	-10.39	0.000	-.1601603	-.1092985
Health_self	.0058985	.0489064	0.12	0.904	-.0899831	.1017802
Health_other	.1647816	.0471337	3.50	0.000	.0723754	.2571879
Conservative_01	-.1685595	.0466746	-3.61	0.000	-.2600657	-.0770533
Conservative_other	-.0843625	.0683066	-1.24	0.217	-.2182785	.0495536
GeoCensus_d1	-.0403149	.0640549	-0.63	0.529	-.1658955	.0852656
GeoCensus_d2	.0104202	.0549613	0.19	0.850	-.0973323	.1181726
GeoCensus_d3	.0208909	.0674937	0.31	0.757	-.1114316	.1532133
Current_measures	.0181059	.0558091	0.32	0.746	-.0913086	.1275205
Measures_clear	-.0243493	.0136713	-1.78	0.075	-.0511521	.0024535
MA_Perc_Threat_SC3	.111529	.0198069	5.63	0.000	.0726974	.1503606
Deterr_SD_Likely_SC2	.2041545	.0144344	14.14	0.000	.1758556	.2324534
Deterr_SD_Severe	-.0589906	.012892	-4.58	0.000	-.0842655	-.0337157
MA_MoralBelief	-.0003532	.0208192	-0.02	0.986	-.0411695	.0404631
MA_Authority_SC2	-.023528	.0139412	-1.69	0.092	-.0508598	.0038039
NOO_SC3	.0556341	.0303137	1.84	0.067	-.0037965	.1150646
NNOO_SC3	.069556	.026528	2.62	0.009	.0175475	.1215645
OOL_SC12	-.1246906	.0176409	-7.07	0.000	-.1592759	-.0901054
PJE_SC4	.0086806	.015673	0.55	0.580	-.0220466	.0394078
Trust_Science_SC4	-.0071862	.0267424	-0.27	0.788	-.0596151	.0452427
Trust_in_media	-.0473993	.0190069	-2.49	0.013	-.0846626	-.010136
Impulsivity_SC4	.0257691	.0234663	1.10	0.272	-.020237	.0717752
NegEmo_SC6	.2416428	.0151018	16.00	0.000	.2120355	.2712502
SN_SC7	-.0062461	.0187652	-0.33	0.739	-.0430356	.0305434
CTC_SC7	.0443716	.02405	1.84	0.065	-.0027788	.0915219
OTC_SC7	.0316717	.0133009	2.38	0.017	.0055951	.0577483
_cons	2.676337	.2261007	11.84	0.000	2.233064	3.119611

	Estimate	Std Err	P>|z|	[95% Conf	Interval]
cde	-.1057489	.03964275	0.008	-.18344868	-.02804911
nie	-.00265614	.00192655	0.168	-.00643219	.0011199
te	-.10840504	.03961913	0.006	-.18605853	-.03075155

cde:controlled direct effect, nie:natural indirect effect, te:total effect

	Observed Coef.	Bias	Bootstrap Std. Err.	[95% Conf.	Interval]	
cde	-.1057489	-.0013102	.03856689	-.1794706	-.0272605	(BC)
nie	-.00265614	.0000332	.00212173	-.0089759	.0001961	(BC)
te	-.10840504	-.0012769	.0385272	-.1830288	-.0307288	(BC)

(BC)   bias-corrected confidence interval

714 .

```
715 . *28.d.1 Mediation model, wave (1-2) on compliance mediated by moral alignment
716 . paramed DV_Compliance_SC7, avar(wave_d1) mvar(MA_MoralBelief) cvars(wave_d2 Age Gender_Female Minority Education Emp
    > alth_other Conservative_01 Conservative_other GeoCensus_d1 GeoCensus_d2 GeoCensus_d3 Current_measures Measures_clear
    > _SC3 NNOO_SC3 OOL_SC12 PJE_SC4 Trust_Science_SC4 Trust_in_media Impulsivity_SC4 NegEmo_SC6 SN_SC7 CTC_SC7 OTC_SC7) a
```

Source	SS	df	MS			
Model	5783.88315	38	152.207451	Number of obs	=	4,348
Residual	4685.07115	4,309	1.08727574	F(38, 4309)	=	139.99
				Prob > F	=	0.0000
Total	10468.9543	4,347	2.40831707	R-squared	=	0.5525
				Adj R-squared	=	0.5485
				Root MSE	=	1.0427

DV_Compliance_SC7	Coef.	Std. Err.	t	P>|t|	[95% Conf.	Interval]
wave_d1	-.1008503	.0393389	-2.56	0.010	-.1779747	-.0237259
MA_MoralBelief	.2074829	.0163511	12.69	0.000	.1754264	.2395394
wave_d2	-.1057489	.0396427	-2.67	0.008	-.1834691	-.0280287
Age	.0033071	.0012878	2.57	0.010	.0007823	.0058319
Gender_Female	.1356169	.0331373	4.09	0.000	.0706507	.2005831
Minority	-.0115737	.0342592	-0.34	0.736	-.0787394	.055592
Education	.0269047	.0111445	2.41	0.016	.0050558	.0487536
Employed	.0456376	.0366804	1.24	0.213	-.0262749	.1175501
Corona_care	.0163155	.053475	0.31	0.760	-.088523	.1211539
Insurance_Public	.1594496	.0483136	3.30	0.001	.06473	.2541691
Insurance_Private	.1641472	.050507	3.25	0.001	.0651275	.2631669
SES_before	.0073995	.008939	0.83	0.408	-.0101256	.0249246
SES_change	-.0057423	.0103144	-0.56	0.578	-.0259637	.0144792
Health_self	-.0606208	.0384104	-1.58	0.115	-.135925	.0146833
Health_other	.0013416	.0370705	0.04	0.971	-.0713357	.0740189
Conservative_01	.0104904	.0367129	0.29	0.775	-.0614859	.0824667
Conservative_other	.0324492	.0536565	0.60	0.545	-.0727451	.1376435
GeoCensus_d1	-.0347888	.0503101	-0.69	0.489	-.1334224	.0638449
GeoCensus_d2	-.0517626	.043166	-1.20	0.231	-.1363901	.0328649
GeoCensus_d3	.016889	.0530092	0.32	0.750	-.0870362	.1208143
Current_measures	.2499748	.0438322	5.70	0.000	.1640412	.3359085
Measures_clear	.0045911	.0107412	0.43	0.669	-.0164671	.0256494
MA_Perc_Threat_SC3	.2237231	.0156131	14.33	0.000	.1931133	.2543328
Costs_SC5	.0190175	.0119631	1.59	0.112	-.0044364	.0424713
Deterr_SD_Likely_SC2	.0035187	.0115967	0.30	0.762	-.0192168	.0262542
Deterr_SD_Severe	-.0029309	.0101497	-0.29	0.773	-.0228295	.0169678
MA_Authority_SC2	-.005595	.0109528	-0.51	0.609	-.0270681	.0158781
NOO_SC3	.0002182	.0238173	0.01	0.993	-.046476	.0469123
NNOO_SC3	-.0291422	.0208513	-1.40	0.162	-.0700214	.0117371
OOL_SC12	.0295314	.013935	2.12	0.034	.0022117	.0568512
PJE_SC4	-.0267302	.0123098	-2.17	0.030	-.0508637	-.0025966
Trust_Science_SC4	.0090169	.0210033	0.43	0.668	-.0321604	.0501941
Trust_in_media	-.0415326	.0149385	-2.78	0.005	-.0708198	-.0122454
Impulsivity_SC4	-.084699	.0184327	-4.60	0.000	-.1208366	-.0485615
NegEmo_SC6	.0156761	.012208	1.28	0.199	-.0082577	.03961
SN_SC7	.0632409	.0147381	4.29	0.000	.0343466	.0921352
CTC_SC7	.4347295	.018896	23.01	0.000	.3976837	.4717753
OTC_SC7	-.0312296	.0104532	-2.99	0.003	-.0517233	-.010736
_cons	.1463905	.1804394	0.81	0.417	-.2073636	.5001446

Source	SS	df	MS			
Model	6348.4721	37	171.580327	Number of obs	=	4,348
Residual	4066.74662	4,310	.9435607	F(37, 4310)	=	181.84
				Prob > F	=	0.0000
Total	10415.2187	4,347	2.39595554	R-squared	=	0.6095
				Adj R-squared	=	0.6062
				Root MSE	=	.97137

MA_MoralBelief	Coef.	Std. Err.	t	P>|t|	[95% Conf.	Interval]
wave_d1	-.0411294	.0366415	-1.12	0.262	-.1129656	.0307068
wave_d2	-.0770566	.0369113	-2.09	0.037	-.1494217	-.0046914
Age	.0000484	.0011997	0.04	0.968	-.0023036	.0024004
Gender_Female	.0607295	.0308559	1.97	0.049	.0002361	.1212229
Minority	.0392302	.0319092	1.23	0.219	-.0233283	.1017887
Education	.0229413	.010376	2.21	0.027	.002599	.0432835
Employed	-.0331151	.0341666	-0.97	0.332	-.1000992	.0338691
Corona_care	-.2061973	.0497165	-4.15	0.000	-.3036673	-.1087274
Insurance_Public	.0485239	.0450014	1.08	0.281	-.039702	.1367498
Insurance_Private	.08934	.0470311	1.90	0.058	-.0028651	.1815451
SES_before	.0124557	.0083252	1.50	0.135	-.0038659	.0287772
SES_change	.0005885	.0096085	0.06	0.951	-.0182491	.0194262
Health_self	-.1350556	.0357228	-3.78	0.000	-.2050906	-.0650206
Health_other	.0705972	.034517	2.05	0.041	.0029261	.1382683
Conservative_01	-.0849681	.0341762	-2.49	0.013	-.151971	-.0179653
Conservative_other	-.0327616	.0499822	-0.66	0.512	-.1307525	.0652293
GeoCensus_d1	-.0470031	.0468618	-1.00	0.316	-.1388764	.0448703
GeoCensus_d2	.0077291	.0402119	0.19	0.848	-.071107	.0865651
GeoCensus_d3	.0032096	.0493817	0.06	0.948	-.0936039	.1000231
Current_measures	.2102342	.0407069	5.16	0.000	.1304276	.2900407
Measures_clear	.0225115	.0100003	2.25	0.024	.0029058	.0421172
MA_Perc_Threat_SC3	.5130126	.0122672	41.82	0.000	.4889625	.5370627
Costs_SC5	-.000189	.0111445	-0.02	0.986	-.0220379	.0216598
Deterr_SD_Likely_SC2	-.0185241	.0107994	-1.72	0.086	-.0396965	.0026483
Deterr_SD_Severe	.0190097	.0094507	2.01	0.044	.0004814	.037538
MA_Authority_SC2	.0240184	.0101967	2.36	0.019	.0040275	.0440092
NOO_SC3	.1234731	.0221076	5.59	0.000	.0801309	.1668154
NNOO_SC3	-.0351246	.019417	-1.81	0.071	-.073192	.0029428
OOL_SC12	.0150524	.0129794	1.16	0.246	-.0103939	.0404987
PJE_SC4	-.0103115	.0114664	-0.90	0.369	-.0327915	.0121685
Trust_Science_SC4	.1223615	.019477	6.28	0.000	.0841765	.1605465
Trust_in_media	-.0389841	.0139036	-2.80	0.005	-.0662423	-.0117259
Impulsivity_SC4	-.0260899	.0171667	-1.52	0.129	-.0597455	.0075657
NegEmo_SC6	-.0078914	.0113719	-0.69	0.488	-.0301862	.0144034
SN_SC7	.017698	.0137269	1.29	0.197	-.0092139	.0446098
CTC_SC7	.2732926	.0171036	15.98	0.000	.2397608	.3068245
OTC_SC7	-.0135574	.0097357	-1.39	0.164	-.0326443	.0055296
_cons	.419817	.1679701	2.50	0.012	.0905092	.7491248

	Estimate	Std Err	P>|z|	[95% Conf	Interval]
cde	-.10085033	.03933886	0.010	-.1779545	-.02374617
nie	-.00853365	.00763217	0.264	-.02349271	.00642541
te	-.10938398	.0400611	0.006	-.18790373	-.03086423

cde:controlled direct effect, nie:natural indirect effect, te:total effect

	Observed Coef.	Bias	Bootstrap Std. Err.	[95% Conf.	Interval]	
cde	-.10085033	-.0006079	.03946667	-.1861741	-.0287204	(BC)
nie	-.00853365	-.0004181	.00751539	-.0223451	.0074709	(BC)
te	-.10938398	-.001026	.04032185	-.1937245	-.0354406	(BC)

(BC)   bias-corrected confidence interval

717 .

```
718 . *28.d.2 Mediation model, wave (1-3) on compliance mediated by moral alignment
719 . paramed DV_Compliance_SC7, avar(wave_d2) mvar(MA_MoralBelief) cvars(wave_d1 Age Gender_Female Minority Education Emp
    > alth_other Conservative_01 Conservative_other GeoCensus_d1 GeoCensus_d2 GeoCensus_d3 Current_measures Measures_clear
    > _SC3 NNOO_SC3 OOL_SC12 PJE_SC4 Trust_Science_SC4 Trust_in_media Impulsivity_SC4 NegEmo_SC6 SN_SC7 CTC_SC7 OTC_SC7) a
```

Source	SS	df	MS
Model	5783.88315	38	152.207451
Residual	4685.07115	4,309	1.08727574
Total	10468.9543	4,347	2.40831707

Number of obs	=	4,348
F(38, 4309)	=	139.99
Prob > F	=	0.0000
R-squared	=	0.5525
Adj R-squared	=	0.5485
Root MSE	=	1.0427

DV_Compliance_SC7	Coef.	Std. Err.	t	P>|t|	[95% Conf.	Interval]
wave_d2	-.1057489	.0396427	-2.67	0.008	-.1834691	-.0280287
MA_MoralBelief	.2074829	.0163511	12.69	0.000	.1754264	.2395394
wave_d1	-.1008503	.0393389	-2.56	0.010	-.1779747	-.0237259
Age	.0033071	.0012878	2.57	0.010	.0007823	.0058319
Gender_Female	.1356169	.0331373	4.09	0.000	.0706507	.2005831
Minority	-.0115737	.0342592	-0.34	0.736	-.0787394	.055592
Education	.0269047	.0111445	2.41	0.016	.0050558	.0487536
Employed	.0456376	.0366804	1.24	0.213	-.0262749	.1175501
Corona_care	.0163155	.053475	0.31	0.760	-.088523	.1211539
Insurance_Public	.1594496	.0483136	3.30	0.001	.06473	.2541691
Insurance_Private	.1641472	.050507	3.25	0.001	.0651275	.2631669
SES_before	.0073995	.008939	0.83	0.408	-.0101256	.0249246
SES_change	-.0057423	.0103144	-0.56	0.578	-.0259637	.0144792
Health_self	-.0606208	.0384104	-1.58	0.115	-.135925	.0146833
Health_other	.0013416	.0370705	0.04	0.971	-.0713357	.0740189
Conservative_01	.0104904	.0367129	0.29	0.775	-.0614859	.0824667
Conservative_other	.0324492	.0536565	0.60	0.545	-.0727451	.1376435
GeoCensus_d1	-.0347888	.0503101	-0.69	0.489	-.1334224	.0638449
GeoCensus_d2	-.0517626	.043166	-1.20	0.231	-.1363901	.0328649
GeoCensus_d3	.016889	.0530092	0.32	0.750	-.0870362	.1208143
Current_measures	.2499748	.0438322	5.70	0.000	.1640412	.3359085
Measures_clear	.0045911	.0107412	0.43	0.669	-.0164671	.0256494
MA_Perc_Threat_SC3	.2237231	.0156131	14.33	0.000	.1931133	.2543328
Costs_SC5	.0190175	.0119631	1.59	0.112	-.0044364	.0424713
Deterr_SD_Likely_SC2	.0035187	.0115967	0.30	0.762	-.0192168	.0262542
Deterr_SD_Severe	-.0029309	.0101497	-0.29	0.773	-.0228295	.0169678
MA_Authority_SC2	-.005595	.0109528	-0.51	0.609	-.0270681	.0158781
NOO_SC3	.0002182	.0238173	0.01	0.993	-.046476	.0469123
NNOO_SC3	-.0291422	.0208513	-1.40	0.162	-.0700214	.0117371
OOL_SC12	.0295314	.013935	2.12	0.034	.0022117	.0568512
PJE_SC4	-.0267302	.0123098	-2.17	0.030	-.0508637	-.0025966
Trust_Science_SC4	.0090169	.0210033	0.43	0.668	-.0321604	.0501941
Trust_in_media	-.0415326	.0149385	-2.78	0.005	-.0708198	-.0122454
Impulsivity_SC4	-.084699	.0184327	-4.60	0.000	-.1208366	-.0485615
NegEmo_SC6	.0156761	.012208	1.28	0.199	-.0082577	.03961
SN_SC7	.0632409	.0147381	4.29	0.000	.0343466	.0921352
CTC_SC7	.4347295	.018896	23.01	0.000	.3976837	.4717753
OTC_SC7	-.0312296	.0104532	-2.99	0.003	-.0517233	-.010736
_cons	.1463905	.1804394	0.81	0.417	-.2073636	.5001446

Source	SS	df	MS
Model	6348.4721	37	171.580327
Residual	4066.74662	4,310	.9435607
Total	10415.2187	4,347	2.39595554

Number of obs	=	4,348
F(37, 4310)	=	181.84
Prob > F	=	0.0000
R-squared	=	0.6095
Adj R-squared	=	0.6062
Root MSE	=	.97137

MA_MoralBelief	Coef.	Std. Err.	t	P>|t|	[95% Conf.	Interval]
wave_d2	-.0770566	.0369113	-2.09	0.037	-.1494217	-.0046914
wave_d1	-.0411294	.0366415	-1.12	0.262	-.1129656	.0307068
Age	.0000484	.0011997	0.04	0.968	-.0023036	.0024004
Gender_Female	.0607295	.0308559	1.97	0.049	.0002361	.1212229
Minority	.0392302	.0319092	1.23	0.219	-.0233283	.1017887
Education	.0229413	.010376	2.21	0.027	.002599	.0432835
Employed	-.0331151	.0341666	-0.97	0.332	-.1000992	.0338691
Corona_care	-.2061973	.0497165	-4.15	0.000	-.3036673	-.1087274
Insurance_Public	.0485239	.0450014	1.08	0.281	-.039702	.1367498
Insurance_Private	.08934	.0470311	1.90	0.058	-.0028651	.1815451
SES_before	.0124557	.0083252	1.50	0.135	-.0038659	.0287772
SES_change	.0005885	.0096085	0.06	0.951	-.0182491	.0194262
Health_self	-.1350556	.0357228	-3.78	0.000	-.2050906	-.0650206
Health_other	.0705972	.034517	2.05	0.041	.0029261	.1382683
Conservative_01	-.0849681	.0341762	-2.49	0.013	-.151971	-.0179653
Conservative_other	-.0327616	.0499822	-0.66	0.512	-.1307525	.0652293
GeoCensus_d1	-.0470031	.0468618	-1.00	0.316	-.1388764	.0448703
GeoCensus_d2	.0077291	.0402119	0.19	0.848	-.071107	.0865651
GeoCensus_d3	.0032096	.0493817	0.06	0.948	-.0936039	.1000231
Current_measures	.2102342	.0407069	5.16	0.000	.1304276	.2900407
Measures_clear	.0225115	.0100003	2.25	0.024	.0029058	.0421172
MA_Perc_Threat_SC3	.5130126	.0122672	41.82	0.000	.4889625	.5370627
Costs_SC5	-.000189	.0111445	-0.02	0.986	-.0220379	.0216598
Deterr_SD_Likely_SC2	-.0185241	.0107994	-1.72	0.086	-.0396965	.0026483
Deterr_SD_Severe	.0190097	.0094507	2.01	0.044	.0004814	.037538
MA_Authority_SC2	.0240184	.0101967	2.36	0.019	.0040275	.0440092
NOO_SC3	.1234731	.0221076	5.59	0.000	.0801309	.1668154
NNOO_SC3	-.0351246	.019417	-1.81	0.071	-.073192	.0029428
OOL_SC12	.0150524	.0129794	1.16	0.246	-.0103939	.0404987
PJE_SC4	-.0103115	.0114664	-0.90	0.369	-.0327915	.0121685
Trust_Science_SC4	.1223615	.019477	6.28	0.000	.0841765	.1605465
Trust_in_media	-.0389841	.0139036	-2.80	0.005	-.0662423	-.0117259
Impulsivity_SC4	-.0260899	.0171667	-1.52	0.129	-.0597455	.0075657
NegEmo_SC6	-.0078914	.0113719	-0.69	0.488	-.0301862	.0144034
SN_SC7	.017698	.0137269	1.29	0.197	-.0092139	.0446098
CTC_SC7	.2732926	.0171036	15.98	0.000	.2397608	.3068245
OTC_SC7	-.0135574	.0097357	-1.39	0.164	-.0326443	.0055296
_cons	.419817	.1679701	2.50	0.012	.0905092	.7491248

	Estimate	Std Err	P>|z|	[95% Conf	Interval]
cde	-.1057489	.03964275	0.008	-.18344868	-.02804911
nie	-.01598792	.00776141	0.039	-.0312003	-.00077555
te	-.12173682	.04035606	0.003	-.2008347	-.04263893

cde:controlled direct effect, nie:natural indirect effect, te:total effect

	Observed Coef.	Bias	Bootstrap Std. Err.	[95% Conf.	Interval]	
cde	-.1057489	-.0013102	.03856689	-.1794706	-.0272605	(BC)
nie	-.01598792	-.0001459	.00763295	-.0307487	-.0012128	(BC)
te	-.12173682	-.0014561	.03925233	-.1988589	-.0387043	(BC)

(BC)   bias-corrected confidence interval

720 .

```
721 . *28.e.1 Mediation model, wave (1-2) on compliance mediated by social norms
722 . paramed DV_Compliance_SC7, avar(wave_d1) mvar(SN_SC7) cvars(wave_d2 Age Gender_Female Minority Education Employed Co
    > er Conservative_01 Conservative_other GeoCensus_d1 GeoCensus_d2 GeoCensus_d3 Current_measures Measures_clear MA_Perc
    > C2 NOO_SC3 NNOO_SC3 OOL_SC12 PJE_SC4 Trust_Science_SC4 Trust_in_media Impulsivity_SC4 NegEmo_SC6  CTC_SC7 OTC_SC7) a
```

Source	SS	df	MS		
				Number of obs =	4,348
				F(38, 4309) =	139.99
Model	5783.88315	38	152.207451	Prob > F =	0.0000
Residual	4685.07115	4,309	1.08727574	R-squared =	0.5525
				Adj R-squared =	0.5485
Total	10468.9543	4,347	2.40831707	Root MSE =	1.0427

DV_Compliance_SC7	Coef.	Std. Err.	t	P>|t|	[95% Conf.	Interval]
wave_d1	-.1008503	.0393389	-2.56	0.010	-.1779747	-.0237259
SN_SC7	.0632409	.0147381	4.29	0.000	.0343466	.0921352
wave_d2	-.1057489	.0396427	-2.67	0.008	-.1834691	-.0280287
Age	.0033071	.0012878	2.57	0.010	.0007823	.0058319
Gender_Female	.1356169	.0331373	4.09	0.000	.0706507	.2005831
Minority	-.0115737	.0342592	-0.34	0.736	-.0787394	.055592
Education	.0269047	.0111445	2.41	0.016	.0050558	.0487536
Employed	.0456376	.0366804	1.24	0.213	-.0262749	.1175501
Corona_care	.0163155	.053475	0.31	0.760	-.088523	.1211539
Insurance_Public	.1594496	.0483136	3.30	0.001	.06473	.2541691
Insurance_Private	.1641472	.050507	3.25	0.001	.0651275	.2631669
SES_before	.0073995	.008939	0.83	0.408	-.0101256	.0249246
SES_change	-.0057423	.0103144	-0.56	0.578	-.0259637	.0144792
Health_self	-.0606208	.0384104	-1.58	0.115	-.135925	.0146833
Health_other	.0013416	.0370705	0.04	0.971	-.0713357	.0740189
Conservative_01	.0104904	.0367129	0.29	0.775	-.0614859	.0824667
Conservative_other	.0324492	.0536565	0.60	0.545	-.0727451	.1376435
GeoCensus_d1	-.0347888	.0503101	-0.69	0.489	-.1334224	.0638449
GeoCensus_d2	-.0517626	.043166	-1.20	0.231	-.1363901	.0328649
GeoCensus_d3	.016889	.0530092	0.32	0.750	-.0870362	.1208143
Current_measures	.2499748	.0438322	5.70	0.000	.1640412	.3359085
Measures_clear	.0045911	.0107412	0.43	0.669	-.0164671	.0256494
MA_Perc_Threat_SC3	.2237231	.0156131	14.33	0.000	.1931133	.2543328
Costs_SC5	.0190175	.0119631	1.59	0.112	-.0044364	.0424713
Deterr_SD_Likely_SC2	.0035187	.0115967	0.30	0.762	-.0192168	.0262542
Deterr_SD_Severe	-.0029309	.0101497	-0.29	0.773	-.0228295	.0169678
MA_MoralBelief	.2074829	.0163511	12.69	0.000	.1754264	.2395394
MA_Authority_SC2	-.005595	.0109528	-0.51	0.609	-.0270681	.0158781
NOO_SC3	.0002182	.0238173	0.01	0.993	-.046476	.0469123
NNOO_SC3	-.0291422	.0208513	-1.40	0.162	-.0700214	.0117371
OOL_SC12	.0295314	.013935	2.12	0.034	.0022117	.0568512
PJE_SC4	-.0267302	.0123098	-2.17	0.030	-.0508637	-.0025966
Trust_Science_SC4	.0090169	.0210033	0.43	0.668	-.0321604	.0501941
Trust_in_media	-.0415326	.0149385	-2.78	0.005	-.0708198	-.0122454
Impulsivity_SC4	-.084699	.0184327	-4.60	0.000	-.1208366	-.0485615
NegEmo_SC6	.0156761	.012208	1.28	0.199	-.0082577	.03961
CTC_SC7	.4347295	.018896	23.01	0.000	.3976837	.4717753
OTC_SC7	-.0312296	.0104532	-2.99	0.003	-.0517233	-.010736
_cons	.1463905	.1804394	0.81	0.417	-.2073636	.5001446

Source	SS	df	MS		
				Number of obs =	4,348
				F(37, 4310) =	77.95
Model	3349.52095	37	90.5275931	Prob > F =	0.0000
Residual	5005.59493	4,310	1.16139093	R-squared =	0.4009
				Adj R-squared =	0.3958
Total	8355.11587	4,347	1.92204184	Root MSE =	1.0777

SN_SC7	Coef.	Std. Err.	t	P>|t|	[95% Conf.	Interval]
wave_d1	-.1547429	.0405892	-3.81	0.000	-.2343185	-.0751673
wave_d2	-.1859291	.0408736	-4.55	0.000	-.2660624	-.1057957
Age	.0030905	.0013302	2.32	0.020	.0004827	.0056984
Gender_Female	.0045254	.0342481	0.13	0.895	-.0626185	.0716692
Minority	.0805618	.0353864	2.28	0.023	.0111864	.1499373
Education	.0029336	.011518	0.25	0.799	-.0196476	.0255147
Employed	.050142	.0379023	1.32	0.186	-.024166	.12445
Corona_care	-.0065109	.0552674	-0.12	0.906	-.1148635	.1018417
Insurance_Public	.0241826	.0499318	0.48	0.628	-.0737094	.1220746
Insurance_Private	-.0058846	.0521999	-0.11	0.910	-.1082234	.0964541
SES_before	.0224741	.0092323	2.43	0.015	.004374	.0405742
SES_change	.0150019	.0106577	1.41	0.159	-.0058926	.0358965
Health_self	.0155697	.0396973	0.39	0.695	-.0622573	.0933968
Health_other	-.085427	.0382911	-2.23	0.026	-.1604972	-.0103568
Conservative_01	.0419022	.0379382	1.10	0.269	-.0324762	.1162807
Conservative_other	-.0724871	.0554441	-1.31	0.191	-.1811861	.0362119
GeoCensus_d1	-.1611059	.0519386	-3.10	0.002	-.2629322	-.0592795
GeoCensus_d2	-.1721117	.0445358	-3.86	0.000	-.2594249	-.0847986
GeoCensus_d3	-.060224	.0547784	-1.10	0.272	-.1676179	.0471699
Current_measures	.0620301	.0452916	1.37	0.171	-.0267648	.150825
Measures_clear	.0197972	.0110972	1.78	0.074	-.0019589	.0415534
MA_Perc_Threat_SC3	-.0205258	.0161335	-1.27	0.203	-.0521556	.0111041
Costs_SC5	-.0041154	.012364	-0.33	0.739	-.0283552	.0201243
Deterr_SD_Likely_SC2	.0541372	.011957	4.53	0.000	.0306953	.0775791
Deterr_SD_Severe	.013751	.0104879	1.31	0.190	-.0068106	.0343126
MA_MoralBelief	.0217837	.0168959	1.29	0.197	-.011341	.0549084
MA_Authority_SC2	.0695098	.0112703	6.17	0.000	.0474141	.0916054
NOO_SC3	.0429835	.0246069	1.75	0.081	-.0052588	.0912257
NNOO_SC3	.1157517	.021478	5.39	0.000	.0736438	.1578596
OOL_SC12	.0149808	.0144003	1.04	0.298	-.0132512	.0432127
PJE_SC4	.0591404	.0126905	4.66	0.000	.0342605	.0840203
Trust_Science_SC4	.0492717	.0216944	2.27	0.023	.0067396	.0918038
Trust_in_media	.0346443	.0154302	2.25	0.025	.0043931	.0648956
Impulsivity_SC4	.0598401	.0190288	3.14	0.002	.0225339	.0971462
NegEmo_SC6	.0120565	.0126158	0.96	0.339	-.0126771	.0367901
CTC_SC7	.4803255	.0181071	26.53	0.000	.4448263	.5158247
OTC_SC7	.073839	.0107449	6.87	0.000	.0527735	.0949046
_cons	.0036409	.1864879	0.02	0.984	-.3619714	.3692532

	Estimate	Std Err	P>|z|	[95% Conf	Interval]
cde	-.10085033	.03933886	0.010	-.1779545	-.02374617
nie	-.00978608	.00343368	0.004	-.0165161	-.00305607
te	-.11063642	.0393565	0.005	-.18777515	-.03349769

cde:controlled direct effect, nie:natural indirect effect, te:total effect

	Observed Coef.	Bias	Bootstrap Std. Err.	[95% Conf.	Interval]	
cde	-.10085033	-.0006079	.03946667	-.1861741	-.0287204	(BC)
nie	-.00978608	.0001245	.00344157	-.0180546	-.0042588	(BC)
te	-.11063642	-.0004834	.03937346	-.1948502	-.0390008	(BC)

(BC)   bias-corrected confidence interval

723 .

```
724 . *28.e.2 Mediation model, wave (1-3) on compliance mediated by social norms
725 . paramed DV_Compliance_SC7, avar(wave_d2) mvar(SN_SC7) cvars(wave_d1 Age Gender_Female Minority Education Employed Co
    > er Conservative_01 Conservative_other GeoCensus_d1 GeoCensus_d2 GeoCensus_d3 Current_measures Measures_clear MA_Perc
    > C2 NOO_SC3 NNOO_SC3 OOL_SC12 PJE_SC4 Trust_Science_SC4 Trust_in_media Impulsivity_SC4 NegEmo_SC6  CTC_SC7 OTC_SC7) a
```

Source	SS	df	MS
Model	5783.88315	38	152.207451
Residual	4685.07115	4,309	1.08727574
Total	10468.9543	4,347	2.40831707

Number of obs	=	4,348
F(38, 4309)	=	139.99
Prob > F	=	0.0000
R-squared	=	0.5525
Adj R-squared	=	0.5485
Root MSE	=	1.0427

DV_Compliance_SC7	Coef.	Std. Err.	t	P>|t|	[95% Conf.	Interval]
wave_d2	-.1057489	.0396427	-2.67	0.008	-.1834691	-.0280287
SN_SC7	.0632409	.0147381	4.29	0.000	.0343466	.0921352
wave_d1	-.1008503	.0393389	-2.56	0.010	-.1779747	-.0237259
Age	.0033071	.0012878	2.57	0.010	.0007823	.0058319
Gender_Female	.1356169	.0331373	4.09	0.000	.0706507	.2005831
Minority	-.0115737	.0342592	-0.34	0.736	-.0787394	.055592
Education	.0269047	.0111445	2.41	0.016	.0050558	.0487536
Employed	.0456376	.0366804	1.24	0.213	-.0262749	.1175501
Corona_care	.0163155	.053475	0.31	0.760	-.088523	.1211539
Insurance_Public	.1594496	.0483136	3.30	0.001	.06473	.2541691
Insurance_Private	.1641472	.050507	3.25	0.001	.0651275	.2631669
SES_before	.0073995	.008939	0.83	0.408	-.0101256	.0249246
SES_change	-.0057423	.0103144	-0.56	0.578	-.0259637	.0144792
Health_self	-.0606208	.0384104	-1.58	0.115	-.135925	.0146833
Health_other	.0013416	.0370705	0.04	0.971	-.0713357	.0740189
Conservative_01	.0104904	.0367129	0.29	0.775	-.0614859	.0824667
Conservative_other	.0324492	.0536565	0.60	0.545	-.0727451	.1376435
GeoCensus_d1	-.0347888	.0503101	-0.69	0.489	-.1334224	.0638449
GeoCensus_d2	-.0517626	.043166	-1.20	0.231	-.1363901	.0328649
GeoCensus_d3	.016889	.0530092	0.32	0.750	-.0870362	.1208143
Current_measures	.2499748	.0438322	5.70	0.000	.1640412	.3359085
Measures_clear	.0045911	.0107412	0.43	0.669	-.0164671	.0256494
MA_Perc_Threat_SC3	.2237231	.0156131	14.33	0.000	.1931133	.2543328
Costs_SC5	.0190175	.0119631	1.59	0.112	-.0044364	.0424713
Deterr_SD_Likely_SC2	.0035187	.0115967	0.30	0.762	-.0192168	.0262542
Deterr_SD_Severe	-.0029309	.0101497	-0.29	0.773	-.0228295	.0169678
MA_MoralBelief	.2074829	.0163511	12.69	0.000	.1754264	.2395394
MA_Authority_SC2	-.005595	.0109528	-0.51	0.609	-.0270681	.0158781
NOO_SC3	.0002182	.0238173	0.01	0.993	-.046476	.0469123
NNOO_SC3	-.0291422	.0208513	-1.40	0.162	-.0700214	.0117371
OOL_SC12	.0295314	.013935	2.12	0.034	.0022117	.0568512
PJE_SC4	-.0267302	.0123098	-2.17	0.030	-.0508637	-.0025966
Trust_Science_SC4	.0090169	.0210033	0.43	0.668	-.0321604	.0501941
Trust_in_media	-.0415326	.0149385	-2.78	0.005	-.0708198	-.0122454
Impulsivity_SC4	-.084699	.0184327	-4.60	0.000	-.1208366	-.0485615
NegEmo_SC6	.0156761	.012208	1.28	0.199	-.0082577	.03961
CTC_SC7	.4347295	.018896	23.01	0.000	.3976837	.4717753
OTC_SC7	-.0312296	.0104532	-2.99	0.003	-.0517233	-.010736
_cons	.1463905	.1804394	0.81	0.417	-.2073636	.5001446

Source	SS	df	MS
Model	3349.52095	37	90.5275931
Residual	5005.59493	4,310	1.16139093
Total	8355.11587	4,347	1.92204184

Number of obs	=	4,348
F(37, 4310)	=	77.95
Prob > F	=	0.0000
R-squared	=	0.4009
Adj R-squared	=	0.3958
Root MSE	=	1.0777

SN_SC7	Coef.	Std. Err.	t	P>|t|	[95% Conf.	Interval]
wave_d2	-.1859291	.0408736	-4.55	0.000	-.2660624	-.1057957
wave_d1	-.1547429	.0405892	-3.81	0.000	-.2343185	-.0751673
Age	.0030905	.0013302	2.32	0.020	.0004827	.0056984
Gender_Female	.0045254	.0342481	0.13	0.895	-.0626185	.0716692
Minority	.0805618	.0353864	2.28	0.023	.0111864	.1499373
Education	.0029336	.011518	0.25	0.799	-.0196476	.0255147
Employed	.050142	.0379023	1.32	0.186	-.024166	.12445
Corona_care	-.0065109	.0552674	-0.12	0.906	-.1148635	.1018417
Insurance_Public	.0241826	.0499318	0.48	0.628	-.0737094	.1220746
Insurance_Private	-.0058846	.0521999	-0.11	0.910	-.1082234	.0964541
SES_before	.0224741	.0092323	2.43	0.015	.004374	.0405742
SES_change	.0150019	.0106577	1.41	0.159	-.0058926	.0358965
Health_self	.0155697	.0396973	0.39	0.695	-.0622573	.0933968
Health_other	-.085427	.0382911	-2.23	0.026	-.1604972	-.0103568
Conservative_01	.0419022	.0379382	1.10	0.269	-.0324762	.1162807
Conservative_other	-.0724871	.0554441	-1.31	0.191	-.1811861	.0362119
GeoCensus_d1	-.1611059	.0519386	-3.10	0.002	-.2629322	-.0592795
GeoCensus_d2	-.1721117	.0445358	-3.86	0.000	-.2594249	-.0847986
GeoCensus_d3	-.060224	.0547784	-1.10	0.272	-.1676179	.0471699
Current_measures	.0620301	.0452916	1.37	0.171	-.0267648	.150825
Measures_clear	.0197972	.0110972	1.78	0.074	-.0019589	.0415534
MA_Perc_Threat_SC3	-.0205258	.0161335	-1.27	0.203	-.0521556	.0111041
Costs_SC5	-.0041154	.012364	-0.33	0.739	-.0283552	.0201243
Deterr_SD_Likely_SC2	.0541372	.011957	4.53	0.000	.0306953	.0775791
Deterr_SD_Severe	.013751	.0104879	1.31	0.190	-.0068106	.0343126
MA_MoralBelief	.0217837	.0168959	1.29	0.197	-.011341	.0549084
MA_Authority_SC2	.0695098	.0112703	6.17	0.000	.0474141	.0916054
NOO_SC3	.0429835	.0246069	1.75	0.081	-.0052588	.0912257
NNOO_SC3	.1157517	.021478	5.39	0.000	.0736438	.1578596
OOL_SC12	.0149808	.0144003	1.04	0.298	-.0132512	.0432127
PJE_SC4	.0591404	.0126905	4.66	0.000	.0342605	.0840203
Trust_Science_SC4	.0492717	.0216944	2.27	0.023	.0067396	.0918038
Trust_in_media	.0346443	.0154302	2.25	0.025	.0043931	.0648956
Impulsivity_SC4	.0598401	.0190288	3.14	0.002	.0225339	.0971462
NegEmo_SC6	.0120565	.0126158	0.96	0.339	-.0126771	.0367901
CTC_SC7	.4803255	.0181071	26.53	0.000	.4448263	.5158247
OTC_SC7	.073839	.0107449	6.87	0.000	.0527735	.0949046
_cons	.0036409	.1864879	0.02	0.984	-.3619714	.3692532

	Estimate	Std Err	P>|z|	[95% Conf	Interval]
cde	-.1057489	.03964275	0.008	-.18344868	-.02804911
nie	-.01175833	.00376704	0.002	-.01914172	-.00437493
te	-.11750722	.03963231	0.003	-.19518655	-.03982789

cde:controlled direct effect, nie:natural indirect effect, te:total effect

	Observed Coef.	Bias	Bootstrap Std. Err.	[95% Conf.	Interval]	
cde	-.1057489	-.0013102	.03856689	-.1794706	-.0272605	(BC)
nie	-.01175833	.0001904	.00388145	-.0211834	-.0055514	(BC)
te	-.11750722	-.0011198	.03844894	-.1913872	-.0421069	(BC)

(BC) bias-corrected confidence interval

726 .

```
727 . *28.f.1 Mediation model, wave (1-2) on compliance mediated by capacity
728 . paramed DV_Compliance_SC7, avar(wave_d1) mvar(CTC_SC7) cvars(wave_d2 Age Gender_Female Minority Education Employed C
    > her Conservative_01 Conservative_other GeoCensus_d1 GeoCensus_d2 GeoCensus_d3 Current_measures Measures_clear MA_Per
    > SC2 NOO_SC3 NNOO_SC3 OOL_SC12 PJE_SC4 Trust_Science_SC4 Trust_in_media Impulsivity_SC4 NegEmo_SC6 SN_SC7  OTC_SC7) a
```

Source	SS	df	MS			
				Number of obs	=	4,348
				F(38, 4309)	=	139.99
Model	5783.88315	38	152.207451	Prob > F	=	0.0000
Residual	4685.07115	4,309	1.08727574	R-squared	=	0.5525
				Adj R-squared	=	0.5485
Total	10468.9543	4,347	2.40831707	Root MSE	=	1.0427

DV_Compliance_SC7	Coef.	Std. Err.	t	P>|t|	[95% Conf.	Interval]
wave_d1	-.1008503	.0393389	-2.56	0.010	-.1779747	-.0237259
CTC_SC7	.4347295	.018896	23.01	0.000	.3976837	.4717753
wave_d2	-.1057489	.0396427	-2.67	0.008	-.1834691	-.0280287
Age	.0033071	.0012878	2.57	0.010	.0007823	.0058319
Gender_Female	.1356169	.0331373	4.09	0.000	.0706507	.2005831
Minority	-.0115737	.0342592	-0.34	0.736	-.0787394	.055592
Education	.0269047	.0111445	2.41	0.016	.0050558	.0487536
Employed	.0456376	.0366804	1.24	0.213	-.0262749	.1175501
Corona_care	.0163155	.053475	0.31	0.760	-.088523	.1211539
Insurance_Public	.1594496	.0483136	3.30	0.001	.06473	.2541691
Insurance_Private	.1641472	.050507	3.25	0.001	.0651275	.2631669
SES_before	.0073995	.008939	0.83	0.408	-.0101256	.0249246
SES_change	-.0057423	.0103144	-0.56	0.578	-.0259637	.0144792
Health_self	-.0606208	.0384104	-1.58	0.115	-.135925	.0146833
Health_other	.0013416	.0370705	0.04	0.971	-.0713357	.0740189
Conservative_01	.0104904	.0367129	0.29	0.775	-.0614859	.0824667
Conservative_other	.0324492	.0536565	0.60	0.545	-.0727451	.1376435
GeoCensus_d1	-.0347888	.0503101	-0.69	0.489	-.1334224	.0638449
GeoCensus_d2	-.0517626	.043166	-1.20	0.231	-.1363901	.0328649
GeoCensus_d3	.016889	.0530092	0.32	0.750	-.0870362	.1208143
Current_measures	.2499748	.0438322	5.70	0.000	.1640412	.3359085
Measures_clear	.0045911	.0107412	0.43	0.669	-.0164671	.0256494
MA_Perc_Threat_SC3	.2237231	.0156131	14.33	0.000	.1931133	.2543328
Costs_SC5	.0190175	.0119631	1.59	0.112	-.0044364	.0424713
Deterr_SD_Likely_SC2	.0035187	.0115967	0.30	0.762	-.0192168	.0262542
Deterr_SD_Severe	-.0029309	.0101497	-0.29	0.773	-.0228295	.0169678
MA_MoralBelief	.2074829	.0163511	12.69	0.000	.1754264	.2395394
MA_Authority_SC2	-.005595	.0109528	-0.51	0.609	-.0270681	.0158781
NOO_SC3	.0002182	.0238173	0.01	0.993	-.046476	.0469123
NNOO_SC3	-.0291422	.0208513	-1.40	0.162	-.0700214	.0117371
OOL_SC12	.0295314	.013935	2.12	0.034	.0022117	.0568512
PJE_SC4	-.0267302	.0123098	-2.17	0.030	-.0508637	-.0025966
Trust_Science_SC4	.0090169	.0210033	0.43	0.668	-.0321604	.0501941
Trust_in_media	-.0415326	.0149385	-2.78	0.005	-.0708198	-.0122454
Impulsivity_SC4	-.084699	.0184327	-4.60	0.000	-.1208366	-.0485615
NegEmo_SC6	.0156761	.012208	1.28	0.199	-.0082577	.03961
SN_SC7	.0632409	.0147381	4.29	0.000	.0343466	.0921352
OTC_SC7	-.0312296	.0104532	-2.99	0.003	-.0517233	-.010736
_cons	.1463905	.1804394	0.81	0.417	-.2073636	.5001446

Source	SS	df	MS			
				Number of obs	=	4,348
				F(37, 4310)	=	131.03
Model	3425.21643	37	92.5734169	Prob > F	=	0.0000
Residual	3045.10302	4,310	.706520422	R-squared	=	0.5294
				Adj R-squared	=	0.5253
Total	6470.31945	4,347	1.48845628	Root MSE	=	.84055

CTC_SC7	Coef.	Std. Err.	t	P>|t|	[95% Conf.	Interval]
wave_d1	.0516528	.0317016	1.63	0.103	-.0104985	.1138042
wave_d2	.0215345	.0319546	0.67	0.500	-.041113	.0841819
Age	.0040801	.0010363	3.94	0.000	.0020485	.0061117
Gender_Female	.0191166	.0267106	0.72	0.474	-.03325	.0714832
Minority	-.1024543	.0275725	-3.72	0.000	-.1565105	-.0483981
Education	.0189979	.008979	2.12	0.034	.0013945	.0366013
Employed	-.0179901	.0295671	-0.61	0.543	-.0759568	.0399765
Corona_care	-.2170716	.0429795	-5.05	0.000	-.3013336	-.1328097
Insurance_Public	-.0103793	.0389456	-0.27	0.790	-.0867327	.0659742
Insurance_Private	-.0349385	.0407105	-0.86	0.391	-.114752	.0448751
SES_before	.0048919	.0072054	0.68	0.497	-.0092344	.0190182
SES_change	-.0011446	.0083145	-0.14	0.891	-.0174452	.015156
Health_self	-.0488828	.0309539	-1.58	0.114	-.1095685	.0118028
Health_other	.00548	.0298827	0.18	0.855	-.0531054	.0640655
Conservative_01	.0053701	.0295944	0.18	0.856	-.0526503	.0633904
Conservative_other	.1216868	.0432131	2.82	0.005	.0369669	.2064067
GeoCensus_d1	.071537	.0405406	1.76	0.078	-.0079435	.1510176
GeoCensus_d2	.0577308	.0347853	1.66	0.097	-.0104662	.1259279
GeoCensus_d3	.0173717	.0427302	0.41	0.684	-.0664016	.1011449
Current_measures	.3046613	.0350273	8.70	0.000	.2359896	.3733329
Measures_clear	.0437297	.0086329	5.07	0.000	.0268048	.0606546
MA_Perc_Threat_SC3	.0373278	.012573	2.97	0.003	.0126783	.0619773
Costs_SC5	.0177851	.0096397	1.84	0.065	-.0011138	.0366839
Deterr_SD_Likely_SC2	-.0127522	.0093461	-1.36	0.173	-.0310755	.005571
Deterr_SD_Severe	.0113476	.0081799	1.39	0.165	-.0046893	.0273844
MA_MoralBelief	.2046364	.0128068	15.98	0.000	.1795284	.2297444
MA_Authority_SC2	-.0072999	.0088284	-0.83	0.408	-.0246081	.0100084
NOO_SC3	.1608835	.0190422	8.45	0.000	.1235509	.198216
NNOO_SC3	-.0347924	.0168	-2.07	0.038	-.067729	-.0018557
OOL_SC12	-.016829	.0112302	-1.50	0.134	-.0388459	.0051879
PJE_SC4	.0277232	.009914	2.80	0.005	.0082866	.0471598
Trust_Science_SC4	.0723447	.016895	4.28	0.000	.0392218	.1054675
Trust_in_media	-.0531898	.0120147	-4.43	0.000	-.0767449	-.0296347
Impulsivity_SC4	-.0881053	.014798	-5.95	0.000	-.117117	-.0590937
NegEmo_SC6	.0196166	.0098364	1.99	0.046	.0003323	.038901
SN_SC7	.2922011	.0110153	26.53	0.000	.2706056	.3137967
OTC_SC7	.0341247	.0084104	4.06	0.000	.0176361	.0506134
_cons	1.385399	.1439145	9.63	0.000	1.103252	1.667545

	Estimate	Std Err	P>|z|	[95% Conf	Interval]
cde	-.10085033	.03933886	0.010	-.1779545	-.02374617
nie	.02245501	.01381612	0.104	-.00462458	.0495346
te	-.07839532	.04167164	0.060	-.16007174	.0032811

cde:controlled direct effect, nie:natural indirect effect, te:total effect

	Observed Coef.	Bias	Bootstrap Std. Err.	[95% Conf.	Interval]	
cde	-.10085033	-.0006079	.03946667	-.1861741	-.0287204	(BC)
nie	.02245501	-.0005461	.01375692	-.0013496	.0516582	(BC)
te	-.07839532	-.001154	.04206874	-.162769	.0026251	(BC)

(BC)   bias-corrected confidence interval

729 .

```
730 . *28.f.2 Mediation model, wave (1-3) on compliance mediated by capacity
731 . paramed DV_Compliance_SC7, avar(wave_d2) mvar(CTC_SC7) cvars(wave_d1 Age Gender_Female Minority Education Employed C
    > her Conservative_01 Conservative_other GeoCensus_d1 GeoCensus_d2 GeoCensus_d3 Current_measures Measures_clear MA_Per
    > SC2 NOO_SC3 NNOO_SC3 OOL_SC12 PJE_SC4 Trust_Science_SC4 Trust_in_media Impulsivity_SC4 NegEmo_SC6 SN_SC7  OTC_SC7) a
```

Source	SS	df	MS
Model	5783.88315	38	152.207451
Residual	4685.07115	4,309	1.08727574
Total	10468.9543	4,347	2.40831707

Number of obs	=	4,348
F(38, 4309)	=	139.99
Prob > F	=	0.0000
R-squared	=	0.5525
Adj R-squared	=	0.5485
Root MSE	=	1.0427

DV_Compliance_SC7	Coef.	Std. Err.	t	P>|t|	[95% Conf.	Interval]
wave_d2	-.1057489	.0396427	-2.67	0.008	-.1834691	-.0280287
CTC_SC7	.4347295	.018896	23.01	0.000	.3976837	.4717753
wave_d1	-.1008503	.0393389	-2.56	0.010	-.1779747	-.0237259
Age	.0033071	.0012878	2.57	0.010	.0007823	.0058319
Gender_Female	.1356169	.0331373	4.09	0.000	.0706507	.2005831
Minority	-.0115737	.0342592	-0.34	0.736	-.0787394	.055592
Education	.0269047	.0111445	2.41	0.016	.0050558	.0487536
Employed	.0456376	.0366804	1.24	0.213	-.0262749	.1175501
Corona_care	.0163155	.053475	0.31	0.760	-.088523	.1211539
Insurance_Public	.1594496	.0483136	3.30	0.001	.06473	.2541691
Insurance_Private	.1641472	.050507	3.25	0.001	.0651275	.2631669
SES_before	.0073995	.008939	0.83	0.408	-.0101256	.0249246
SES_change	-.0057423	.0103144	-0.56	0.578	-.0259637	.0144792
Health_self	-.0606208	.0384104	-1.58	0.115	-.135925	.0146833
Health_other	.0013416	.0370705	0.04	0.971	-.0713357	.0740189
Conservative_01	.0104904	.0367129	0.29	0.775	-.0614859	.0824667
Conservative_other	.0324492	.0536565	0.60	0.545	-.0727451	.1376435
GeoCensus_d1	-.0347888	.0503101	-0.69	0.489	-.1334224	.0638449
GeoCensus_d2	-.0517626	.043166	-1.20	0.231	-.1363901	.0328649
GeoCensus_d3	.016889	.0530092	0.32	0.750	-.0870362	.1208143
Current_measures	.2499748	.0438322	5.70	0.000	.1640412	.3359085
Measures_clear	.0045911	.0107412	0.43	0.669	-.0164671	.0256494
MA_Perc_Threat_SC3	.2237231	.0156131	14.33	0.000	.1931133	.2543328
Costs_SC5	.0190175	.0119631	1.59	0.112	-.0044364	.0424713
Deterr_SD_Likely_SC2	.0035187	.0115967	0.30	0.762	-.0192168	.0262542
Deterr_SD_Severe	-.0029309	.0101497	-0.29	0.773	-.0228295	.0169678
MA_MoralBelief	.2074829	.0163511	12.69	0.000	.1754264	.2395394
MA_Authority_SC2	-.005595	.0109528	-0.51	0.609	-.0270681	.0158781
NOO_SC3	.0002182	.0238173	0.01	0.993	-.046476	.0469123
NNOO_SC3	-.0291422	.0208513	-1.40	0.162	-.0700214	.0117371
OOL_SC12	.0295314	.013935	2.12	0.034	.0022117	.0568512
PJE_SC4	-.0267302	.0123098	-2.17	0.030	-.0508637	-.0025966
Trust_Science_SC4	.0090169	.0210033	0.43	0.668	-.0321604	.0501941
Trust_in_media	-.0415326	.0149385	-2.78	0.005	-.0708198	-.0122454
Impulsivity_SC4	-.084699	.0184327	-4.60	0.000	-.1208366	-.0485615
NegEmo_SC6	.0156761	.012208	1.28	0.199	-.0082577	.03961
SN_SC7	.0632409	.0147381	4.29	0.000	.0343466	.0921352
OTC_SC7	-.0312296	.0104532	-2.99	0.003	-.0517233	-.010736
_cons	.1463905	.1804394	0.81	0.417	-.2073636	.5001446

Source	SS	df	MS
Model	3425.21643	37	92.5734169
Residual	3045.10302	4,310	.706520422
Total	6470.31945	4,347	1.48845628

Number of obs	=	4,348
F(37, 4310)	=	131.03
Prob > F	=	0.0000
R-squared	=	0.5294
Adj R-squared	=	0.5253
Root MSE	=	.84055

CTC_SC7	Coef.	Std. Err.	t	P>|t|	[95% Conf.	Interval]
wave_d2	.0215345	.0319546	0.67	0.500	-.041113	.0841819
wave_d1	.0516528	.0317016	1.63	0.103	-.0104985	.1138042
Age	.0040801	.0010363	3.94	0.000	.0020485	.0061117
Gender_Female	.0191166	.0267106	0.72	0.474	-.03325	.0714832
Minority	-.1024543	.0275725	-3.72	0.000	-.1565105	-.0483981
Education	.0189979	.008979	2.12	0.034	.0013945	.0366013
Employed	-.0179901	.0295671	-0.61	0.543	-.0759568	.0399765
Corona_care	-.2170716	.0429795	-5.05	0.000	-.3013336	-.1328097
Insurance_Public	-.0103793	.0389456	-0.27	0.790	-.0867327	.0659742
Insurance_Private	-.0349385	.0407105	-0.86	0.391	-.114752	.0448751
SES_before	.0048919	.0072054	0.68	0.497	-.0092344	.0190182
SES_change	-.0011446	.0083145	-0.14	0.891	-.0174452	.015156
Health_self	-.0488828	.0309539	-1.58	0.114	-.1095685	.0118028
Health_other	.00548	.0298827	0.18	0.855	-.0531054	.0640655
Conservative_01	.0053701	.0295944	0.18	0.856	-.0526503	.0633904
Conservative_other	.1216868	.0432131	2.82	0.005	.0369669	.2064067
GeoCensus_d1	.071537	.0405406	1.76	0.078	-.0079435	.1510176
GeoCensus_d2	.0577308	.0347853	1.66	0.097	-.0104662	.1259279
GeoCensus_d3	.0173717	.0427302	0.41	0.684	-.0664016	.1011449
Current_measures	.3046613	.0350273	8.70	0.000	.2359896	.3733329
Measures_clear	.0437297	.0086329	5.07	0.000	.0268048	.0606546
MA_Perc_Threat_SC3	.0373278	.012573	2.97	0.003	.0126783	.0619773
Costs_SC5	.0177851	.0096397	1.84	0.065	-.0011138	.0366839
Deterr_SD_Likely_SC2	-.0127522	.0093461	-1.36	0.173	-.0310755	.005571
Deterr_SD_Severe	.0113476	.0081799	1.39	0.165	-.0046893	.0273844
MA_MoralBelief	.2046364	.0128068	15.98	0.000	.1795284	.2297444
MA_Authority_SC2	-.0072999	.0088284	-0.83	0.408	-.0246081	.0100084
NOO_SC3	.1608835	.0190422	8.45	0.000	.1235509	.198216
NNOO_SC3	-.0347924	.0168	-2.07	0.038	-.067729	-.0018557
OOL_SC12	-.016829	.0112302	-1.50	0.134	-.0388459	.0051879
PJE_SC4	.0277232	.009914	2.80	0.005	.0082866	.0471598
Trust_Science_SC4	.0723447	.016895	4.28	0.000	.0392218	.1054675
Trust_in_media	-.0531898	.0120147	-4.43	0.000	-.0767449	-.0296347
Impulsivity_SC4	-.0881053	.014798	-5.95	0.000	-.117117	-.0590937
NegEmo_SC6	.0196166	.0098364	1.99	0.046	.0003323	.038901
SN_SC7	.2922011	.0110153	26.53	0.000	.2706056	.3137967
OTC_SC7	.0341247	.0084104	4.06	0.000	.0176361	.0506134
_cons	1.385399	.1439145	9.63	0.000	1.103252	1.667545

	Estimate	Std Err	P>|z|	[95% Conf	Interval]
cde	-.1057489	.03964275	0.008	-.18344868	-.02804911
nie	.00936167	.01389756	0.501	-.01787755	.0366009
te	-.09638722	.04200427	0.022	-.17871558	-.01405886

cde:controlled direct effect, nie:natural indirect effect, te:total effect

	Observed Coef.	Bias	Bootstrap Std. Err.	[95% Conf.	Interval]	
cde	-.1057489	-.0013102	.03856689	-.1794706	-.0272605	(BC)
nie	.00936167	-.0006835	.01383204	-.0167966	.0362545	(BC)
te	-.09638722	-.0019937	.04153775	-.1767589	-.0162383	(BC)

(BC) bias-corrected confidence interval

732 .

```
733 . *28.g.1 Mediation model, wave (1-2) on compliance mediated by opportunity
734 . paramed DV_Compliance_SC7, avar(wave_d1) mvar(OTC_SC7) cvars(wave_d2 Age Gender_Female Minority Education Employed C
    > her Conservative_01 Conservative_other GeoCensus_d1 GeoCensus_d2 GeoCensus_d3 Current_measures Measures_clear MA_Per
    > SC2 NOO_SC3 NNOO_SC3 OOL_SC12 PJE_SC4 Trust_Science_SC4 Trust_in_media Impulsivity_SC4 NegEmo_SC6 SN_SC7 CTC_SC7 ) a
```

Source	SS	df	MS			
				Number of obs	=	4,348
				F(38, 4309)	=	139.99
Model	5783.88315	38	152.207451	Prob > F	=	0.0000
Residual	4685.07115	4,309	1.08727574	R-squared	=	0.5525
				Adj R-squared	=	0.5485
Total	10468.9543	4,347	2.40831707	Root MSE	=	1.0427

DV_Compliance_SC7	Coef.	Std. Err.	t	P>|t|	[95% Conf.	Interval]
wave_d1	-.1008503	.0393389	-2.56	0.010	-.1779747	-.0237259
OTC_SC7	-.0312296	.0104532	-2.99	0.003	-.0517233	-.010736
wave_d2	-.1057489	.0396427	-2.67	0.008	-.1834691	-.0280287
Age	.0033071	.0012878	2.57	0.010	.0007823	.0058319
Gender_Female	.1356169	.0331373	4.09	0.000	.0706507	.2005831
Minority	-.0115737	.0342592	-0.34	0.736	-.0787394	.055592
Education	.0269047	.0111445	2.41	0.016	.0050558	.0487536
Employed	.0456376	.0366804	1.24	0.213	-.0262749	.1175501
Corona_care	.0163155	.053475	0.31	0.760	-.088523	.1211539
Insurance_Public	.1594496	.0483136	3.30	0.001	.06473	.2541691
Insurance_Private	.1641472	.050507	3.25	0.001	.0651275	.2631669
SES_before	.0073995	.008939	0.83	0.408	-.0101256	.0249246
SES_change	-.0057423	.0103144	-0.56	0.578	-.0259637	.0144792
Health_self	-.0606208	.0384104	-1.58	0.115	-.135925	.0146833
Health_other	.0013416	.0370705	0.04	0.971	-.0713357	.0740189
Conservative_01	.0104904	.0367129	0.29	0.775	-.0614859	.0824667
Conservative_other	.0324492	.0536565	0.60	0.545	-.0727451	.1376435
GeoCensus_d1	-.0347888	.0503101	-0.69	0.489	-.1334224	.0638449
GeoCensus_d2	-.0517626	.043166	-1.20	0.231	-.1363901	.0328649
GeoCensus_d3	.016889	.0530092	0.32	0.750	-.0870362	.1208143
Current_measures	.2499748	.0438322	5.70	0.000	.1640412	.3359085
Measures_clear	.0045911	.0107412	0.43	0.669	-.0164671	.0256494
MA_Perc_Threat_SC3	.2237231	.0156131	14.33	0.000	.1931133	.2543328
Costs_SC5	.0190175	.0119631	1.59	0.112	-.0044364	.0424713
Deterr_SD_Likely_SC2	.0035187	.0115967	0.30	0.762	-.0192168	.0262542
Deterr_SD_Severe	-.0029309	.0101497	-0.29	0.773	-.0228295	.0169678
MA_MoralBelief	.2074829	.0163511	12.69	0.000	.1754264	.2395394
MA_Authority_SC2	-.005595	.0109528	-0.51	0.609	-.0270681	.0158781
NOO_SC3	.0002182	.0238173	0.01	0.993	-.046476	.0469123
NNOO_SC3	-.0291422	.0208513	-1.40	0.162	-.0700214	.0117371
OOL_SC12	.0295314	.013935	2.12	0.034	.0022117	.0568512
PJE_SC4	-.0267302	.0123098	-2.17	0.030	-.0508637	-.0025966
Trust_Science_SC4	.0090169	.0210033	0.43	0.668	-.0321604	.0501941
Trust_in_media	-.0415326	.0149385	-2.78	0.005	-.0708198	-.0122454
Impulsivity_SC4	-.084699	.0184327	-4.60	0.000	-.1208366	-.0485615
NegEmo_SC6	.0156761	.012208	1.28	0.199	-.0082577	.03961
SN_SC7	.0632409	.0147381	4.29	0.000	.0343466	.0921352
CTC_SC7	.4347295	.018896	23.01	0.000	.3976837	.4717753
_cons	.1463905	.1804394	0.81	0.417	-.2073636	.5001446

Source	SS	df	MS			
				Number of obs	=	4,348
				F(37, 4310)	=	24.39
Model	2083.12677	37	56.3007234	Prob > F	=	0.0000
Residual	9950.40681	4,310	2.30867907	R-squared	=	0.1731
				Adj R-squared	=	0.1660
Total	12033.5336	4,347	2.76823869	Root MSE	=	1.5194

OTC_SC7	Coef.	Std. Err.	t	P>|t|	[95% Conf.	Interval]
wave_d1	.2257791	.0572204	3.95	0.000	.1135977	.3379605
wave_d2	.1674023	.0577101	2.90	0.004	.0542607	.2805439
Age	.0006582	.0018766	0.35	0.726	-.0030209	.0043372
Gender_Female	-.0509601	.0482807	-1.06	0.291	-.1456151	.0436949
Minority	-.0755572	.0499084	-1.51	0.130	-.1734034	.022289
Education	-.0032079	.0162394	-0.20	0.843	-.0350455	.0286296
Employed	.1309476	.0534126	2.45	0.014	.0262314	.2356637
Corona_care	.080811	.0779127	1.04	0.300	-.071938	.2335599
Insurance_Public	.1698764	.0703539	2.41	0.016	.0319466	.3078061
Insurance_Private	.1042166	.0735804	1.42	0.157	-.0400389	.2484721
SES_before	.0016401	.0130257	0.13	0.900	-.023897	.0271771
SES_change	.0262153	.0150245	1.74	0.081	-.0032405	.0556712
Health_self	-.0548446	.0559645	-0.98	0.327	-.1645638	.0548745
Health_other	-.0417742	.0540145	-0.77	0.439	-.1476704	.0641221
Conservative_01	-.0726499	.0534858	-1.36	0.174	-.1775095	.0322098
Conservative_other	-.0168345	.0781865	-0.22	0.830	-.1701203	.1364513
GeoCensus_d1	.0593805	.073305	0.81	0.418	-.0843351	.2030961
GeoCensus_d2	.0705251	.0628912	1.12	0.262	-.052774	.1938243
GeoCensus_d3	.0179361	.0772432	0.23	0.816	-.1335003	.1693725
Current_measures	-.019078	.0638705	-0.30	0.765	-.1442971	.1061411
Measures_clear	-.0249943	.0156472	-1.60	0.110	-.0556709	.0056822
MA_Perc_Threat_SC3	.0583975	.0227337	2.57	0.010	.0138278	.1029672
Costs_SC5	.0414821	.0174209	2.38	0.017	.0073282	.075636
Deterr_SD_Likely_SC2	.0143769	.016897	0.85	0.395	-.0187499	.0475037
Deterr_SD_Severe	.0181328	.0147873	1.23	0.220	-.010858	.0471236
MA_MoralBelief	-.0331718	.023821	-1.39	0.164	-.0798733	.0135296
MA_Authority_SC2	.0434023	.0159465	2.72	0.007	.0121391	.0746656
NOO_SC3	-.0426525	.0346999	-1.23	0.219	-.1106821	.0253771
NNOO_SC3	.192977	.0302415	6.38	0.000	.1336882	.2522658
OOL_SC12	-.1662705	.0201472	-8.25	0.000	-.2057693	-.1267717
PJE_SC4	.071567	.0179044	4.00	0.000	.0364651	.1066688
Trust_Science_SC4	.0103758	.0306051	0.34	0.735	-.0496259	.0703774
Trust_in_media	-.0139425	.021767	-0.64	0.522	-.056617	.028732
Impulsivity_SC4	.1204285	.0267969	4.49	0.000	.0678927	.1729643
NegEmo_SC6	.0040677	.017789	0.23	0.819	-.030808	.0389433
SN_SC7	.1467814	.0213593	6.87	0.000	.1049061	.1886567
CTC_SC7	.1115086	.0274823	4.06	0.000	.0576292	.165388
_cons	2.13848	.2609064	8.20	0.000	1.626969	2.649991

	Estimate	Std Err	P>|z|	[95% Conf	Interval]
cde	-.10085033	.03933886	0.010	-.1779545	-.02374617
nie	-.00705099	.0029603	0.017	-.01285319	-.0012488
te	-.10790133	.03930864	0.006	-.18494626	-.03085639

cde:controlled direct effect, nie:natural indirect effect, te:total effect

	Observed Coef.	Bias	Bootstrap Std. Err.	[95% Conf.	Interval]	
cde	-.10085033	-.0006079	.03946667	-.1861741	-.0287204	(BC)
nie	-.00705099	.0001597	.00283956	-.0151104	-.0029908	(BC)
te	-.10790133	-.0004482	.03932867	-.1902632	-.0337228	(BC)

(BC) bias-corrected confidence interval

735 .

```
736 . *28.g.2 Mediation model, wave (1-3) on compliance mediated by opportunity
737 . paramed DV_Compliance_SC7, avar(wave_d2) mvar(OTC_SC7) cvars(wave_d1 Age Gender_Female Minority Education Employed C
    > her Conservative_01 Conservative_other GeoCensus_d1 GeoCensus_d2 GeoCensus_d3 Current_measures Measures_clear MA_Per
    > SC2 NOO_SC3 NNOO_SC3 OOL_SC12 PJE_SC4 Trust_Science_SC4 Trust_in_media Impulsivity_SC4 NegEmo_SC6 SN_SC7 CTC_SC7 ) a
```

Source	SS	df	MS			
				Number of obs	=	4,348
				F(38, 4309)	=	139.99
Model	5783.88315	38	152.207451	Prob > F	=	0.0000
Residual	4685.07115	4,309	1.08727574	R-squared	=	0.5525
				Adj R-squared	=	0.5485
Total	10468.9543	4,347	2.40831707	Root MSE	=	1.0427

DV_Compliance_SC7	Coef.	Std. Err.	t	P>|t|	[95% Conf.	Interval]
wave_d2	-.1057489	.0396427	-2.67	0.008	-.1834691	-.0280287
OTC_SC7	-.0312296	.0104532	-2.99	0.003	-.0517233	-.010736
wave_d1	-.1008503	.0393389	-2.56	0.010	-.1779747	-.0237259
Age	.0033071	.0012878	2.57	0.010	.0007823	.0058319
Gender_Female	.1356169	.0331373	4.09	0.000	.0706507	.2005831
Minority	-.0115737	.0342592	-0.34	0.736	-.0787394	.055592
Education	.0269047	.0111445	2.41	0.016	.0050558	.0487536
Employed	.0456376	.0366804	1.24	0.213	-.0262749	.1175501
Corona_care	.0163155	.053475	0.31	0.760	-.088523	.1211539
Insurance_Public	.1594496	.0483136	3.30	0.001	.06473	.2541691
Insurance_Private	.1641472	.050507	3.25	0.001	.0651275	.2631669
SES_before	.0073995	.008939	0.83	0.408	-.0101256	.0249246
SES_change	-.0057423	.0103144	-0.56	0.578	-.0259637	.0144792
Health_self	-.0606208	.0384104	-1.58	0.115	-.135925	.0146833
Health_other	.0013416	.0370705	0.04	0.971	-.0713357	.0740189
Conservative_01	.0104904	.0367129	0.29	0.775	-.0614859	.0824667
Conservative_other	.0324492	.0536565	0.60	0.545	-.0727451	.1376435
GeoCensus_d1	-.0347888	.0503101	-0.69	0.489	-.1334224	.0638449
GeoCensus_d2	-.0517626	.043166	-1.20	0.231	-.1363901	.0328649
GeoCensus_d3	.016889	.0530092	0.32	0.750	-.0870362	.1208143
Current_measures	.2499748	.0438322	5.70	0.000	.1640412	.3359085
Measures_clear	.0045911	.0107412	0.43	0.669	-.0164671	.0256494
MA_Perc_Threat_SC3	.2237231	.0156131	14.33	0.000	.1931133	.2543328
Costs_SC5	.0190175	.0119631	1.59	0.112	-.0044364	.0424713
Deterr_SD_Likely_SC2	.0035187	.0115967	0.30	0.762	-.0192168	.0262542
Deterr_SD_Severe	-.0029309	.0101497	-0.29	0.773	-.0228295	.0169678
MA_MoralBelief	.2074829	.0163511	12.69	0.000	.1754264	.2395394
MA_Authority_SC2	-.005595	.0109528	-0.51	0.609	-.0270681	.0158781
NOO_SC3	.0002182	.0238173	0.01	0.993	-.046476	.0469123
NNOO_SC3	-.0291422	.0208513	-1.40	0.162	-.0700214	.0117371
OOL_SC12	.0295314	.013935	2.12	0.034	.0022117	.0568512
PJE_SC4	-.0267302	.0123098	-2.17	0.030	-.0508637	-.0025966
Trust_Science_SC4	.0090169	.0210033	0.43	0.668	-.0321604	.0501941
Trust_in_media	-.0415326	.0149385	-2.78	0.005	-.0708198	-.0122454
Impulsivity_SC4	-.084699	.0184327	-4.60	0.000	-.1208366	-.0485615
NegEmo_SC6	.0156761	.012208	1.28	0.199	-.0082577	.03961
SN_SC7	.0632409	.0147381	4.29	0.000	.0343466	.0921352
CTC_SC7	.4347295	.018896	23.01	0.000	.3976837	.4717753
_cons	.1463905	.1804394	0.81	0.417	-.2073636	.5001446

Source	SS	df	MS			
				Number of obs	=	4,348
				F(37, 4310)	=	24.39
Model	2083.12677	37	56.3007234	Prob > F	=	0.0000
Residual	9950.40681	4,310	2.30867907	R-squared	=	0.1731
				Adj R-squared	=	0.1660
Total	12033.5336	4,347	2.76823869	Root MSE	=	1.5194

OTC_SC7	Coef.	Std. Err.	t	P>|t|	[95% Conf.	Interval]
wave_d2	.1674023	.0577101	2.90	0.004	.0542607	.2805439
wave_d1	.2257791	.0572204	3.95	0.000	.1135977	.3379605
Age	.0006582	.0018766	0.35	0.726	-.0030209	.0043372
Gender_Female	-.0509601	.0482807	-1.06	0.291	-.1456151	.0436949
Minority	-.0755572	.0499084	-1.51	0.130	-.1734034	.022289
Education	-.0032079	.0162394	-0.20	0.843	-.0350455	.0286296
Employed	.1309476	.0534126	2.45	0.014	.0262314	.2356637
Corona_care	.080811	.0779127	1.04	0.300	-.071938	.2335599
Insurance_Public	.1698764	.0703539	2.41	0.016	.0319466	.3078061
Insurance_Private	.1042166	.0735804	1.42	0.157	-.0400389	.2484721
SES_before	.0016401	.0130257	0.13	0.900	-.023897	.0271771
SES_change	.0262153	.0150245	1.74	0.081	-.0032405	.0556712
Health_self	-.0548446	.0559645	-0.98	0.327	-.1645638	.0548745
Health_other	-.0417742	.0540145	-0.77	0.439	-.1476704	.0641221
Conservative_01	-.0726499	.0534858	-1.36	0.174	-.1775095	.0322098
Conservative_other	-.0168345	.0781865	-0.22	0.830	-.1701203	.1364513
GeoCensus_d1	.0593805	.073305	0.81	0.418	-.0843351	.2030961
GeoCensus_d2	.0705251	.0628912	1.12	0.262	-.052774	.1938243
GeoCensus_d3	.0179361	.0772432	0.23	0.816	-.1335003	.1693725
Current_measures	-.019078	.0638705	-0.30	0.765	-.1442971	.1061411
Measures_clear	-.0249943	.0156472	-1.60	0.110	-.0556709	.0056822
MA_Perc_Threat_SC3	.0583975	.0227337	2.57	0.010	.0138278	.1029672
Costs_SC5	.0414821	.0174209	2.38	0.017	.0073282	.075636
Deterr_SD_Likely_SC2	.0143769	.016897	0.85	0.395	-.0187499	.0475037
Deterr_SD_Severe	.0181328	.0147873	1.23	0.220	-.010858	.0471236
MA_MoralBelief	-.0331718	.023821	-1.39	0.164	-.0798733	.0135296
MA_Authority_SC2	.0434023	.0159465	2.72	0.007	.0121391	.0746656
NOO_SC3	-.0426525	.0346999	-1.23	0.219	-.1106821	.0253771
NNOO_SC3	.192977	.0302415	6.38	0.000	.1336882	.2522658
OOL_SC12	-.1662705	.0201472	-8.25	0.000	-.2057693	-.1267717
PJE_SC4	.071567	.0179044	4.00	0.000	.0364651	.1066688
Trust_Science_SC4	.0103758	.0306051	0.34	0.735	-.0496259	.0703774
Trust_in_media	-.0139425	.021767	-0.64	0.522	-.056617	.028732
Impulsivity_SC4	.1204285	.0267969	4.49	0.000	.0678927	.1729643
NegEmo_SC6	.0040677	.017789	0.23	0.819	-.030808	.0389433
SN_SC7	.1467814	.0213593	6.87	0.000	.1049061	.1886567
CTC_SC7	.1115086	.0274823	4.06	0.000	.0576292	.165388
_cons	2.13848	.2609064	8.20	0.000	1.626969	2.649991

	Estimate	Std Err	P>|z|	[95% Conf	Interval]
cde	-.1057489	.03964275	0.008	-.18344868	-.02804911
nie	-.00522791	.00251203	0.037	-.01015148	-.00030434
te	-.11097681	.03964509	0.005	-.18868119	-.03327242

cde:controlled direct effect, nie:natural indirect effect, te:total effect

	Observed Coef.	Bias	Bootstrap Std. Err.	[95% Conf.	Interval]	
cde	-.1057489	-.0013102	.03856689	-.1794706	-.0272605	(BC)
nie	-.00522791	.0001342	.00244023	-.012375	-.001787	(BC)
te	-.11097681	-.001176	.03874464	-.1852203	-.0315987	(BC)

(BC)   bias-corrected confidence interval

```
. 
. 
. 
. *****************
. 
. log close
      name:  <unnamed>
       log:  C:\Users\creinde\OneDrive - UvA\RESEARCH\2020\20 03 Coronavirus-measures compliance survey\Data\US\NWO US
  log type:  smcl
 closed on:  17 Jun 2021, 22:47:05
```
