# Supplementary material for: Social distancing in America: Understanding long-term adherence to COVID-19 mitigation recommendations
Source: PLoS One. 2021 Sep 24;16(9):e0257945. doi: 10.1371/journal.pone.0257945 (PMC8462713; doi:10.1371/journal.pone.0257945)
Supplement: Attachment — Submitted filename: Response to Reviewers.docx [file pone.0257945.s013.docx]

Christopher Reinders Folmer

Amsterdam Law School, University of Amsterdam

Department of Jurisprudence

Center of Law and Behavior

P.O. Box 15544

1001 NA Amsterdam

e-mail: c.p.reindersfolmer@uva.nl

Professor Ali B. Mahmoud

Editor of PLOS ONE

Peter J. Tobin College of Business

St. John's University

8000 Utopia Parkway, Queens, NY 11439

United States of America

Dear Professor Mahmoud,

With this letter, we send you the revised version of manuscript “Social Distancing in America:

Understanding Long-term Adherence to COVID-19 Mitigation Recommendations” (PONE-D-20-39307R1).

We were happy to hear the Reviewers’ favorable evaluations of our revised manuscript, and grateful to receive their suggestions for further minor additions.

We respond to all comments in detail below. We thank you for considering our manuscript for publication in your journal.

Yours sincerely,

The authors

**Editor’s comments**

1. Age as a linear predictor

In line with Reviewer 1’s comment, you note that age may have nonlinearities that would be interesting to investigate. As suggested by Reviewer 1, you suggest to present the findings for age by category, using the typical categories for this variable.

We thank you and Reviewer 1 for this suggestion. We agree that age could have interesting nonlinearities that are obscured by treating this covariate as a linear variable. In response, we have carefully considered this suggestion, and conducted additional analyses to assess it. However, this has lead us to conclude that this change will not yield additional insight beyond the original analyses.

A first important consideration is that reducing age to a categorical variable will result in a loss of information by aggregating individuals with different ages into the same category. We also note that in similar articles in your journal, both categorical and continuous measures of age have been used.

More importantly, when dividing participants into age categories (15-17 | 18-20 | 21-44 | 45-64 | 65+, in line with the US Census), we found no differences between age groups – neither when using the lowest age group as the reference category, nor when using the oldest age group. Moreover, using age categories did not change the results of the predictor variables (please see S5 Output). In sum: we found no indications of nonlinearities in terms of the effect of age (probably because such differences are better explained by the predictor variables, eg threat perceptions, than by the unique effect of age). Furthermore, to implement age categories in the manuscript, all analyses presented in the Results section (p. 23-46) would need to be revised (because all coefficients are slightly altered by the loss in degrees of freedom). Given that age is only a control variable in our model, and that the Reviewer presents this as “only a suggestion”, we were hesitant to make substantial changes that do not yield greater informativeness. As such, we have retained the original analyses (with age as a linear covariate) in the final manuscript. However, in case that you or the Reviewers believe that separating age by category nonetheless can be informative, we will of course be happy to revise the results accordingly.

We mention generational differences as a possible avenue for future research in the Discussion (p. 50), in line with your suggestion:

*“Future research could also expand on these findings by zooming in further on specific variables that may directly or indirectly shape compliance (e.g., (…) by separating individuals from different generations [101])”*

2. Distinction between essential and nonessential work

Following Reviewer 2’s suggestion, you suggest that it would be a good idea to include the distinction between essential and nonessential work in the Discussion as a research implication.

We agree that this distinction would be interesting to explore, and have mentioned this as a possible avenue for future research in the Discussion (p. 50):

*“Future research could also expand on these findings by zooming in further on specific variables that may directly or indirectly shape compliance (e.g., by distinguishing essential and nonessential work; by separating individuals from different generations [101]), or by identifying further variables with which our model could be expanded.”*

3. Reference list

As per journal policy, you ask that we review our reference list to ensure that it is complete and correct, and to include the rationale in the case that retracted papers are cited.

We have reviewed all references and made changes wherever appropriate (please see p. 53-64). This chiefly involved adding DOIs where these were not yet provided [19, 24-26, 32, 35, 37, 44, 55, 56, 58, 59, 61-69, 71-73, 75, 77, 79, 84, 96, 11]. Page numbers were added for [25] and [66]. A missing issue number was added for [72].

In addition, the previous version of the manuscript mentioned an incorrect reference for [33]; this has been replaced with the correct reference. An additional relevant reference was added to the discussion [93]. A typo was corrected for [97], and for [99] and [112] the citations were updated (from preprints to published papers). No retracted papers are cited.

**Reviewer 1’s comments**

1. Age as a linear predictor

As a final suggestion, the Reviewer suggests to present the findings for age by category, using the typical categories for this variable.

We thank the Reviewer for this suggestion. We have responded to it in detail in our response to the Editor’s point 1 (please see above).

**Reviewer 2’s comments**

1. Distinction between essential and nonessential work

The Reviewer notes that because we mentioned that the distinction between essential and nonessential work is important (in our rebuttal letter for the previous revision round), it might be prudent to add this to the Discussion, as a direction or suggestion for future research.

We thank the Reviewer for this suggestion. As detailed in our response to the Editor’s point 2, we now mention this in the Discussion (please see p. 50).
